# Decoding drug tolerance: insights into the *Rv0274* gene's role in isoniazid tolerance

**DOI:** 10.3389/fmicb.2025.1697416

**Published:** 2025-11-27

**Authors:** Min Li, Yun Wang, Xiaohong Xiang, Lingling Dong, Qiming Li, Zhou Liu, Wenwen Chu, Ye Naifang, Zhen Gong, Qiang Zhou

**Affiliations:** 1 Department of Clinical Laboratory, The Second Affiliated Hospital of Anhui Medical University, Hefei, Anhui, China; 2 School of Pharmacy, Chongqing Medical and Pharmaceutical College, Chongqing, China; 3 Department of Clinical Laboratory, The First Affiliated Hospital of Henan University, Henan University, Kaifeng, China; 4 Department of Clinical Laboratory Center, Anhui Chest Hospital, Hefei, China; 5 Department of Microbiology, Tumor and Cell Biology, Karolinska Institutet, Stockholm, Sweden

**Keywords:** *Mycobacterium tuberculosis*, glyoxalase, isoniazid, *Rv0274*, drug tolerance

## Abstract

**Introduction:**

Isoniazid is widely used in the treatment of pulmonary tuberculosis, yet the mechanisms underlying its tolerance remain incompletely understood. The *Rv0274* gene of Mycobacterium tuberculosis, presumed to belong to the aldehyde dehydrogenase family, has been hypothesized to contribute to isoniazid tolerance. *Mycobacterium smegmatis* was used in this study as a surrogate model to investigate this possibility.

**Methods:**

We generated an MSMEG_0608 knockout strain and performed drug susceptibility testing, growth curve analysis, biofilm formation assays, transcriptomic profiling, and RT-qPCR validation. Complementation with *Rv0274* and knockdown of *MSMEG_0606* were further conducted to substantiate the regulatory relationships observed.

**Results:**

The deletion of *MSMEG_0608* significantly impaired isoniazid tolerance. Complementation and gene knockdown experiments supported the involvement of *Rv0274/MSMEG_0608* in modulating the expression of genes associated with *Rv0273c/MSMEG_0606*, ultimately influencing inhA expression. These findings consistently demonstrated that *MSMEG_0608* is integral to the isoniazid tolerance phenotype.

**Discussion:**

Our results suggest that *Rv0274* (*MSMEG_0608*) negatively regulates genes linked to *Rv0273c* (*MSMEG_0606*), thereby contributing to alterations in inhA expression and influencing isoniazid tolerance. This work provides preliminary mechanistic insight into INH tolerance in mycobacteria and establishes a foundation for further investigations into drug tolerance in *Mycobacterium tuberculosis*.

## 1 Introduction

Tuberculosis has become the leading cause of mortality attributable to a single infectious pathogen worldwide, and the global spread of drug-resistant strains poses a formidable public health challenge (WHO, 2024). The widespread administration of anti-tuberculosis drugs has accelerated the emergence and dissemination of multidrug-resistant *Mycobacterium tuberculosis* (*M. tuberculosis*), thereby complicating disease management. Elucidating the molecular mechanisms underlying drug tolerance is thus crucial for optimizing therapeutic strategies and curbing the global tuberculosis epidemic.

The *Rv0274* gene of *Mycobacterium tuberculosis* remains functionally uncharacterized. Structurally, it encodes a soluble protein of 193 amino acids with a predicted molecular weight of approximately 21 kDa and features an extracellular loop cleavage near its C-terminus. *Rv0274* is highly conserved across various mycobacterial species, and sequence homology analysis suggests that it belongs to the aldehyde dehydrogenase family. Previous studies have shown that mutations within the promoter region of *Rv0274* enhance bacterial sensitivity to prothionamide, an industrial precursor for the synthesis of isoniazid (Rawat et al., 2003). Based on these findings, we hypothesize that *Rv0274* may play a critical role in the development of isoniazid tolerance.

Recent studies have highlighted that aldehyde metabolism and redox homeostasis are tightly linked to antibiotic stress adaptation and survival in mycobacteria (Limon et al., 2023; Darwin and Stanley, 2022; Chen et al., 2024). Aldehyde-detoxifying enzymes, such as the formaldehyde-degrading MscR in *M. smegmatis*, and redox-balancing systems in *M. tuberculosis* have been shown to influence drug tolerance and oxidative stress responses. These findings provide a mechanistic framework supporting the potential involvement of aldehyde dehydrogenase family members, including *Rv0274*, in INH responsiveness through redox-linked pathways.

Isoniazid (INH) remains a cornerstone in the frontline treatment of tuberculosis (Tiberi et al., 2018). Within host cells, INH is activated by the catalase-peroxidase enzyme KatG to generate an isonicotinyl intermediate, which subsequently conjugates with nicotinamide adenine dinucleotide (NAD) to form an isonicotinyl-NAD adduct. This adduct specifically inhibits InhA, an essential enoyl-acyl carrier protein reductase required for mycolic acid biosynthesis, thereby compromising the integrity of the mycobacterial cell wall and ultimately inducing bacterial death (Van Scoy and Wilkowske, 1992). The predominant mechanism of INH tolerance involves the remodeling of lipid metabolism pathways: under INH-induced selective pressure, *M. tuberculosis* can downregulate its metabolic activity and lipid synthesis demands, reducing its dependence on lipid precursors (Martinot et al., 2016). Additionally, increasing evidence implicates the regulation of intracellular redox homeostasis in conferring INH tolerance (Jeeves et al., 2015). Nevertheless, beyond these known mechanisms, many molecular determinants and regulatory pathways underlying INH tolerance remain to be fully elucidated.

To elucidate the functional role of *Rv0274* in isoniazid tolerance, we employed *Mycobacterium smegmatis* (*M. smegmatis*) as a surrogate model. Our findings indicate that *Rv0274* may contribute to INH tolerance by negatively regulating *Rv0273c*, thereby relieving its inhibitory effect on the *inhA* gene and facilitating its upregulation. *M. smegmatis* is a well-established surrogate for studying mycobacterial genetics because of its rapid growth, non-pathogenic nature, and high genomic conservation with M. tuberculosis. While this model enables preliminary mechanistic insights, findings require subsequent validation in *M. tuberculosis* to confirm their translational relevance.

## 2 Materials and methods

### 2.1 Bioinformatics analysis

Structural modeling and molecular interaction analyses of *Rv0274* were performed using Swiss-model, AutoDockTools, and PyMOL (Choudhary et al., 2023). Data processing and figure preparation were conducted with GraphPad Prism 7, Photoshop, and DNAMAN. Sequence alignment and conservation analyses were performed using MEGA 7, ClustalX, and Espript 3.0 (https://espript.ibcp.fr/ESPript/cgi-bin/ESPript.cgi). Transcriptomic data were analyzed in Rstudio 4.4.1, and statistical significance was determined using Excel's *t*-test, with thresholds set at ^*^p < 0.05, ^**^p < 0.01, ^***^p < 0.001, and ^****^p < 0.0001.

### 2.2 Bacterial strains, plasmids, primers, and culture conditions

*Escherichia coli* DH5α was used as the host strain for plasmid construction. *M. smegmatis* was cultured in Middlebrook 7H9 medium supplemented with 0.05% Tween-80, with selective antibiotics (hygromycin, acetamide, and tetracycline) added as required. The primer sequences used in this study are provided in Table 1.

**Table 1:** Primers used in this study.

**Primer name**	**Sequence (5^′^–3^′^)**
*MSMEG_0608* primer validation -F	ATGATCAGACCCGACAACCCCAACT
*MSMEG_0608* primer validation -R	CTACTTCGTCGAGGCGACCAAACCG
*MSMEG_0606*-F	GGTGATCTCCCGAAAAGGCA
*MSMEG_0606*-R	CCCAGTACTGCGCAAAATCG
*MSMEG_0608*-F	TCAAATCACTCGACCTGCCC
*MSMEG_0608*-R	ATCTCGCTGTTGTCGTGGTT
SigA-F	TCAACGCCGAAGAAGAGGTG
SigA-R	TGGCGTAGGTCGAGAACTTG
*KatG*-F	CTACGAGCAGATCACCCGTC
*KatG*-R	GGACATGTCGAGCAGGTTGA
*InhA*-F	AGATCGGTGAGGGCAACAAG
*InhA*-R	ACGGTCATCCAGTTGTAGGC
*Rv0274* complementation -F	CGCGGATCCATGATCAAGCCGCACAAC ACCAACA
*Rv0274* complementation -R	CCCAAGCTTGGGCTAACGATCCGCAGC CACCGGA
*MSMEG_0606* knockdown -F	TGCGGCGCTTTTTTTTTTGAATTC
*MSMEG_0606* knockdown -R	CTGCGTTATCCCCTGATTCTG

### 2.3 Construction of knockout strains

The *MSMEG_0608* knockout strain was constructed using a double-crossover homologous recombination strategy (Zhang et al., 2015). The *MSMEG_0608* deletion mutant of *M. smegmatis* mc^2^155 was generated using a homologous recombination strategy. Genomic DNA was extracted from freshly cultured bacteria and used as the template to amplify the upstream (834 bp) and downstream (850 bp) homologous arms of *MSMEG_0608*. The two fragments were fused by overlap-extension PCR and inserted into the pMD19-T vector. After verification by restriction digestion with *Bam*HI and *Hind*III, the hygromycin resistance cassette (*Hyg*^*R*^) excised from pAL75 with *Bgl*II was ligated into the construct to obtain the recombinant Up-Hyg-Down fragment. The resulting fragment was electroporated into *M. smegmatis* harboring pJV53, and transformants were selected on 7H9 agar containing kanamycin (25 μg/mL) and hygromycin (50 μg/mL). Positive colonies were verified by PCR using *MSMEG_0608* specific primers. To remove the selection marker, the hygromycin-resistant clones were serially passaged in antibiotic-free medium (10–12 passages) and then screened on plates with and without hygromycin. Colonies that grew only on antibiotic-free plates were confirmed by PCR and designated as MSMEG_KO0608.

### 2.4 Antibiotic susceptibility testing (MIC determination)

Minimum inhibitory concentrations (MICs) were determined using both a 96-well microdilution method (Wiegand et al., 2008) and the Kirby-Bauer disk diffusion assay. Log-phase cultures grown in 7H9 medium supplemented with 0.05% Tween-80 were inoculated into fresh medium and incubated at 37 °C with shaking. For the microdilution method, serial dilutions of the tested antibiotics were prepared in 96-well plates and incubated for 5–7 days, with the lowest concentration showing no visible growth recorded as the MIC (Garbe et al., 1993). In the Kirby-Bauer assay, bacterial suspensions were evenly spread onto blood agar plates, antibiotic-impregnated disks were applied, and inhibition zone diameters were measured. All experiments were performed in triplicate (Brulle et al., 2013).

### 2.5 Bacterial growth curve

Log-phase cultures were adjusted to an equivalent OD_600_ (Initial inoculation volume 4.5 × 105 CFU/mL) and inoculated into fresh 7H9 medium. Cultures were incubated at 37 °C with shaking at 170 rpm, and OD600 values were recorded at designated time points. All experiments were performed in triplicate to generate growth kinetic curves (Huang et al., 2024).

### 2.6 Sliding motility and biofilm assays

For sliding motility assays, log-phase cultures adjusted to an OD600 of 1.5 were spotted onto 7H9 solid medium. Biofilm formation was assessed using 96-well plates: equal volumes of 7H9 medium supplemented with 2% (v/v) glucose were inoculated with 10% of the adjusted bacterial suspension and incubated at 37 °C for 2–3 days. The resulting biofilms were stained with crystal violet and quantified using a microplate reader. All experiments were performed in triplicate (Chakraborty et al., 2021).

### 2.7 CFU Determination

Bacterial cultures grown in 50 mL of 7H9 medium to an OD600 of 1.5 were serially diluted 10-fold and plated onto 7H9 solid medium containing isoniazid. Colony-forming units (CFUs) were enumerated after incubation for 5 days at 37 °C. All experiments were conducted in triplicate.

### 2.8 Sample preparation and transcriptomic sequencing

Wild-type (WT) and MSMEG_KO0608 strains were cultured in 7H9 medium. Two experimental conditions were established: an untreated group and a control group supplemented with 8 μg/mL isoniazid. After 2–3 days of incubation, bacterial cells were harvested by low-temperature centrifugation, rapidly frozen in liquid nitrogen, and submitted to Sangon Biotech (Shanghai) Co., Ltd. for high-throughput transcriptomic sequencing and downstream bioinformatics analysis (Deng et al., 2022).

### 2.9 RNA extraction and RT-qPCR

Total RNA was extracted using the Trizol reagent, and complementary DNA (cDNA) was synthesized by reverse transcription for quantitative real-time PCR (RT-qPCR) analysis, with sigA used as the internal reference gene. Primer sequences are listed in Table 1. PCR amplification was performed under the following conditions: initial denaturation at 95 °C for 5 min; 40 cycles of 95 °C for 15 s and 60 °C for 30 s; followed by melting curve analysis to verify specificity. Relative gene expression levels were calculated using the 2^∧^−ΔΔCt method, and all experiments were performed in triplicate to ensure reproducibility.

### 2.10 Complementation and knockdown strain construction

The homologous *Rv0274* gene was cloned into the pALACE vector to construct the recombinant plasmid pALACE_*Rv0274*, which was subsequently transformed into the corresponding *M. smegmatis* mutant strain. Complemented strains were selected on 7H9 medium supplemented with 0.2% glycerol and 50 μg/mL hygromycin and verified by Western blot analysis. In addition, CRISPR interference (CRISPRi) (Wong and Rock, 2021) was employed to transcriptionally knock down *MSMEG_0606* expression, and positive clones were selected for downstream experiments.

### 2.11 Statistical analysis

All statistical analyses were performed using GraphPad Prism 10.0. Statistical significance was assessed using one-way and two-way ANOVA, with thresholds defined as ^*^p < 0.05, ^**^p < 0.01, ^***^p < 0.001, and ^****^p < 0.0001. Error bars represent the standard deviation (SD).

## 3 Results

### 3.1 Bioinformatics analysis and structural features of *Rv0274*

**Figure 1:**
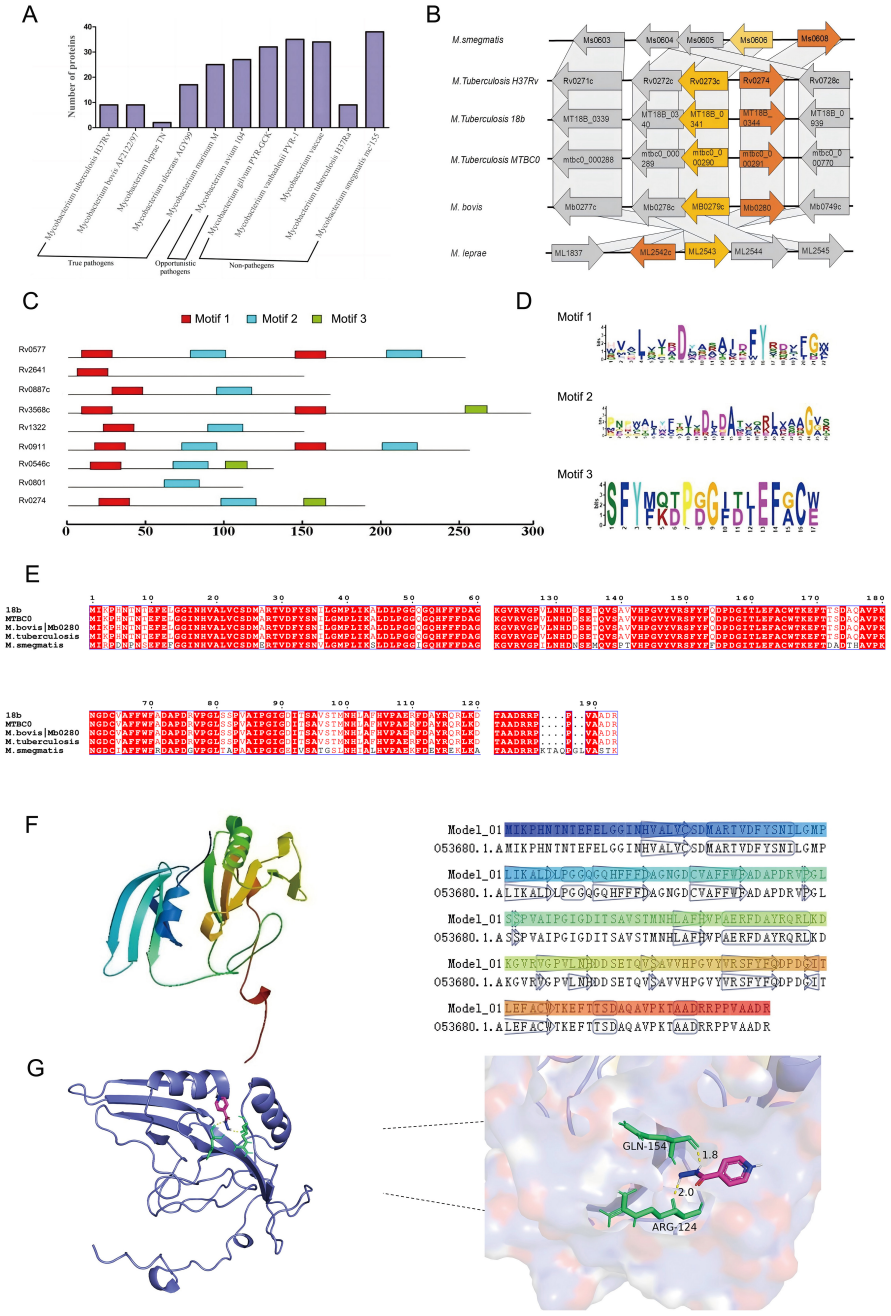
Bioinformatics analysis and structural features of *Rv0274*. **(A, B)** Comparative genomic analysis showing conservation of *Rv0274* and Rv0273c across mycobacterial species with opposite transcriptional orientations. **(C)** Homology analysis revealed that *Rv0274* possesses the characteristic three conserved domains typical of the glyoxalase enzyme family. **(D)** Conserved domain prediction identifying three aldehyde dehydrogenase–specific domains. **(E)** Multiple sequence alignment of *Rv0274* and its homologs in different mycobacterial species. **(F)** Swiss-Model 3D structure of *Rv0274* showing high similarity to reference protein O53680 (TM-score > 0.8). **(G)** Molecular docking showing interaction between *Rv0274* and isoniazid (INH) with −4.72 kcal/mol binding energy and hydrogen bonds at Arg124 (2.0 Å) and Gln154 (1.8 Å).

Bioinformatics analysis revealed that the *Rv0274* gene, a putative member of the aldehyde dehydrogenase family, is highly conserved among *M. tuberculosis, M. bovis*, and *M. smegmatis*, suggesting an important role in mycobacterial physiology. Comparative genomic analyses demonstrated that, although *Rv0274* and its adjacent gene *Rv0273c* are transcribed in opposite directions, their genomic positions and orientations are highly conserved across diverse mycobacterial species (Figures 1A,B), implying potential functional synergy. Homology analysis revealed that *Rv0274* possesses the characteristic three conserved domains typical of the glyoxalase enzyme family (Figure 1C), thereby validating the selection of *MSMEG_0608* as a surrogate model for downstream functional studies. Conserved domain prediction revealed that aldehyde dehydrogenase family proteins typically harbor three characteristic domains, although their precise biological roles remain undefined (Figure 1D). To evaluate the evolutionary conservation of *Rv0274*, multiple sequence alignment was performed among *Mycobacterium tuberculosis* strains (*H37*Rv, 18b, MTBC0, *M. bovis*) and *M. smegmatis MSMEG_0608*. The analysis revealed a high degree of sequence conservation, with *MSMEG_0608* sharing ~74.3% amino acid identity similarity with *Rv0274* of *M. tuberculosis* (Figure 1E). A tertiary structure model of *Rv0274* constructed using Swiss-Model (Figure 1F) showed high structural similarity (TM-score > 0.8) to a reference protein (PDB: O53680), supporting its classification as a bona fide aldehyde dehydrogenase. Furthermore, molecular docking analysis suggested a potential interaction between *Rv0274* and the frontline anti-tuberculosis drug isoniazid (INH), with a predicted binding energy of −4.72 kcal/mol (Figure 1G). Detailed inspection of the docking interface revealed hydrogen bonds between INH and two key residues, Arg124 (bond length: 2.0 Å) and Gln154 (bond length: 1.8 Å). Although the docking energy indicates only a weak interaction, this result should be interpreted as supportive rather than conclusive. The predicted binding pattern provides preliminary structural insight consistent with the proposed regulatory role of *MSMEG_0608*/*Rv0274*, but further biochemical validation is required to establish its physiological relevance. Collectively, these findings imply that *Rv0274* may participate in INH metabolism or tolerance mechanisms (Ferreira et al., 2015), warranting further experimental validation.

### 3.2 Construction and phenotypic analysis of the *MSMEG_0608* knockout strain

**Figure 2:**
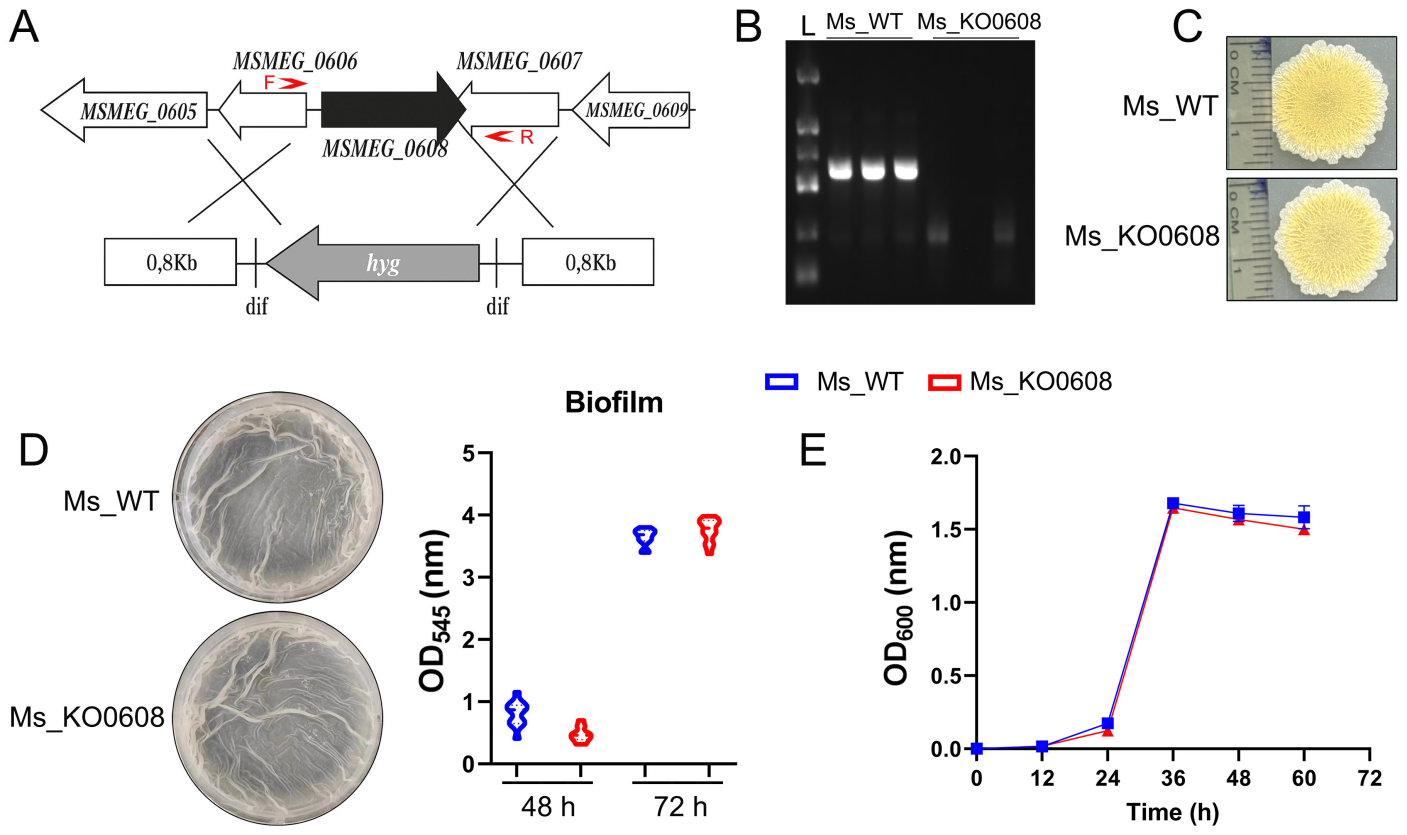
Construction and phenotypic analysis of the MSMEG_0608 knockout strain. **(A, B)** Schematic representation of the MSMEG_0608 gene deletion strategy. The MSMEG_0608 coding sequence was replaced using a two-step homologous recombination approach with approximately 1 kb upstream and downstream flanking regions. The primer pairs used for PCR verification (F and R) are indicated by red arrows, showing the amplification regions for confirming both the successful recombination and the absence of the MSMEG_0608 gene in the mutant strain. PCR verification showing absence of the 943 bp fragment in MSMEG_KO0608 compared with wild-type (WT). **(C)** Sliding motility assays showing no differences between WT and MSMEG_KO0608. **(D)** Biofilm formation assays showing comparable biofilm production in WT and MSMEG_KO0608. **(E)** Growth curves showing similar growth rates of WT and MSMEG_KO0608 in nutrient-rich medium.

An *MSMEG_0608* deletion strain (MSMEG_KO0608) of *M. smegmatis* was successfully generated using a double-crossover homologous recombination strategy. PCR verification confirmed the deletion, as the wild-type mc^2^155 strain produced the expected 943 bp fragment encompassing *MSMEG_0608*, whereas this fragment was absent in the knockout strain (Figures 2A,B). To elucidate the functional role of *MSMEG_0608*, we systematically evaluated the phenotype of the deletion mutant. Sliding motility assays on 7H9 agar plates demonstrated no significant differences in surface migration between MSMEG_KO0608 and the wild-type strain (Figure 2C). Similarly, biofilm formation assays revealed no marked differences (Figure 2D), and growth curve analysis showed comparable growth rates under nutrient-rich conditions (Figure 2E). Collectively, these results indicate that *MSMEG_0608* is not essential for growth, motility, or biofilm formation under standard culture conditions.

### 3.3 Impact of *MSMEG_0608* deletion on prothionamide tolerance and isoniazid sensitivity

**Figure 3:**
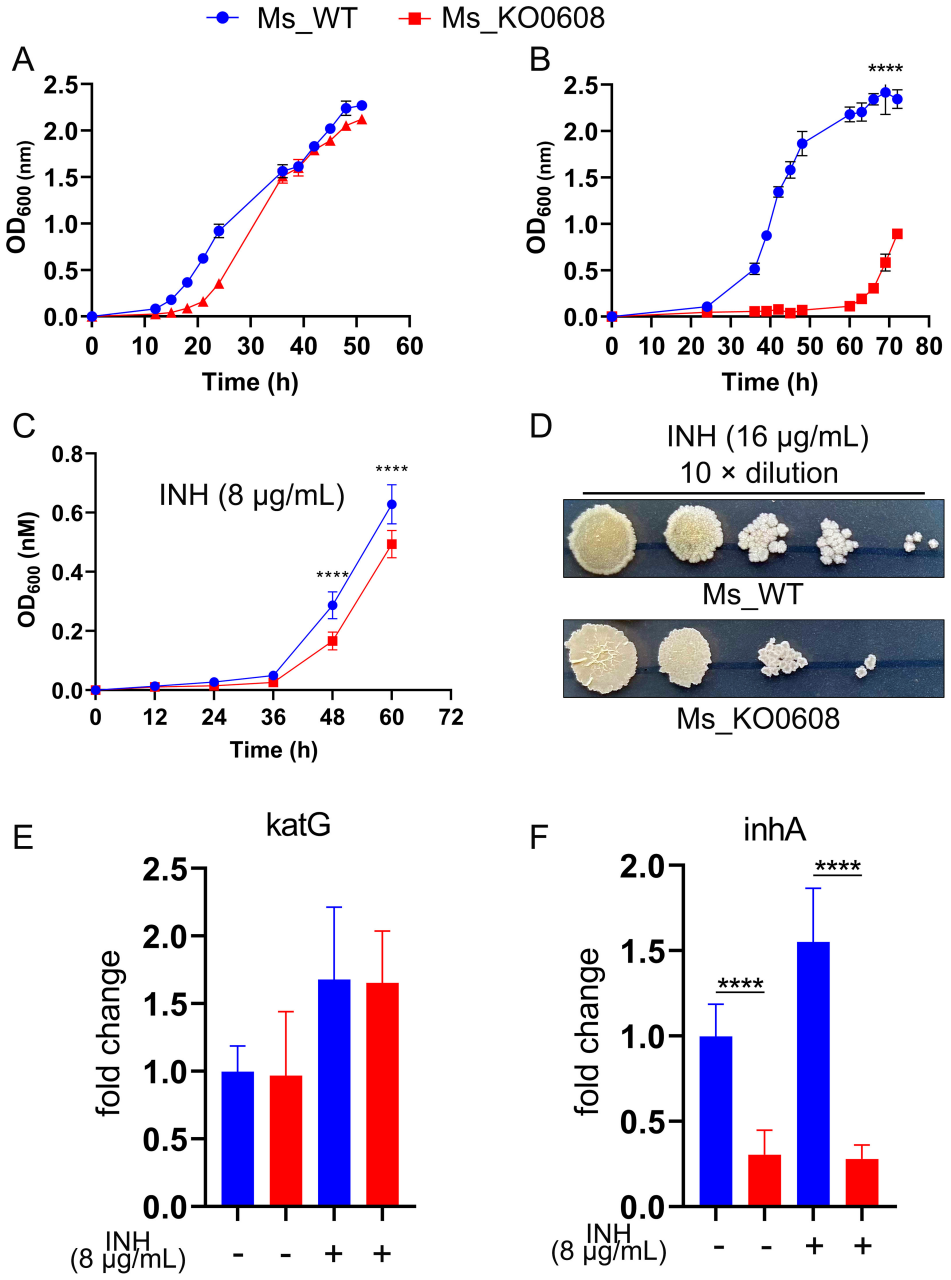
Effect of MSMEG_0608 deletion on prothionamide tolerance and INH susceptibility. **(A)** Growth curves showing similar proliferation of WT and MSMEG_KO0608 at 1.75 mM prothionamide. **(B)** Growth curves showing delayed growth of MSMEG_KO0608 compared with WT at 2.625 mM prothionamide. **(C)** Growth inhibition of MSMEG_KO0608 compared with WT under low INH concentrations. **(D)** Colony numbers showing reduced survival of MSMEG_KO0608 on plates containing 16 μg/mL INH. **(E, F)** RT-qPCR showing unchanged katG expression and reduced inhA expression in MSMEG_KO0608 after INH exposure.

In medium containing 1.75 mM prothionamide, the growth rate of MSMEG_KO0608 was comparable to that of the wild-type mc^2^155 strain (Figure 3A). However, at a higher concentration of 2.625 mM, the wild-type strain resumed exponential growth at ~30 h, whereas MSMEG_KO0608 exhibited a significant growth delay until around 60 h (Figure 3B), indicating that *MSMEG_0608* deletion reduces tolerance to elevated prothionamide levels.

**Figure 4:**
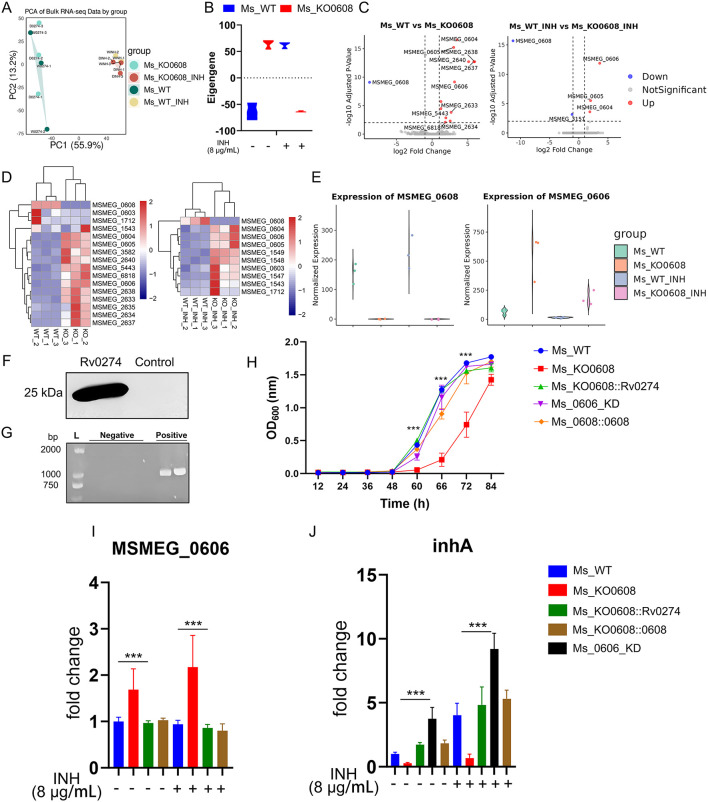
MSMEG_0608 regulates INH tolerance via MSMEG_0606 and inhA. **(A)** PCA showing distinct clustering under different treatments. **(B)** Weighted expression analysis showing global upregulation of module genes in MSMEG_KO0608 without INH and overall downregulation under INH. **(C)** Transcriptomic analysis showing increased MSMEG_0606 expression in MSMEG_KO0608, further elevated by INH. **(D)** Differential expression analysis identifying multiple genes within the MSMEG_0603–MSMEG_1712 locus. **(E)** MSMEG_0606 expression upregulated in MSMEG_KO0608 and suppressed in WT. **(F)** Complementation of *Rv0274* in the MSMEG_KO0608 strain successfully restored gene expression. **(G)** PCR verification of MSMEG_0606 knockdown efficiency. **(H)** Growth curve analysis showing that, under INH treatment, both complemented and MSMEG_0606 knockdown strains exhibited superior growth during the logarithmic phase compared with MSMEG_KO0608, although the late-stage growth of MSMEG_0606 KD was slightly affected by tetracycline supplementation. **(I, J)** RT-qPCR showing MSMEG_0608 represses MSMEG_0606 and upregulates inhA.

Antibiotic susceptibility testing (Table 2) revealed no significant differences in sensitivity to most tested antibiotics (e.g., TE, CIP, TZP) between MSMEG_KO0608 and the wild-type strain. Notably, MIC determination using the microdilution method demonstrated that the MIC of INH for MSMEG_KO0608 was 16 μg/mL—representing a 50% reduction compared with the wild-type value of 32 μg/mL—while the MIC for rifampicin (RIF) remained similar between the two strains (Table 3). Further INH treatment experiments confirmed these findings: under low INH concentrations, logarithmic-phase growth of MSMEG_KO0608 was markedly impaired (Figure 3C), and on 7H9 agar plates containing 16 μg/mL INH, the number of colonies formed by MSMEG_KO0608 was significantly lower than that of the wild-type (Figure 3D). Expression analysis of tolerance-associated genes revealed no significant change in *katG* expression before and after INH exposure, whereas *inhA* expression was significantly downregulated following INH treatment (Figures 4E,F). Collectively, these results suggest that *MSMEG_0608* may modulate INH sensitivity by regulating *inhA* expression.

**Table 2:** Results of *Mycobacterium smegmatis* drug sensitivity to antibiotics on paper tablets.

**Diameter/mm**	**TE**	**CIP**	**TZP**	**C**	**E**	**LEV**	**AZM**
WT	42	30	6	16	19	38	20
MSMEG_KO0608	40	30	6	16	20	39	12

**Table 3:** MIC testing results for *Mycobacterium smegmatis*.

**Antibiotic (μg/mL)**	**WT**	**MSMEG_KO0608**
INH	32	16
RIF	8	8

### 3.4 *MSMEG_0608*-mediated regulation of *MSMEG_0606* and inhA in INH tolerance

Principal component analysis (PCA) revealed distinct clustering of samples under different treatment conditions, indicating substantial transcriptional variation between groups (Figure 4A). Weighted average analysis showed that, under untreated conditions, global gene expression levels were elevated in the MSMEG_KO0608 strain, whereas overall expression was markedly reduced following INH exposure (Figure 4B).

Transcriptomic profiling demonstrated that deletion of *MSMEG_0608* led to significant upregulation of several genes, including MSMEG_0604, MSMEG_0605, and *MSMEG_0606*; notably, *MSMEG_0606* expression was further enhanced under INH treatment (Figure 4C), highlighting a central regulatory role for *MSMEG_0608* in INH-associated metabolic pathways. Comparative transcriptomic analysis identified numerous differentially expressed genes between experimental and control groups, including MSMEG_0603, MSMEG_0604, MSMEG_0605, *MSMEG_0606*, and MSMEG_1712 (Figure 4D). Interestingly, in wild-type strains under INH treatment, *MSMEG_0608* was markedly upregulated while *MSMEG_0606* expression was repressed; conversely, in the MSMEG_KO0608 strain, *MSMEG_0606* expression was strongly upregulated and further enhanced by INH exposure (Figure 4E).

Previous studies reported that the homologous gene of *MSMEG_0606, Rv0273c*, encodes a putative transcriptional regulator, EtbR, which specifically binds to the upstream regulatory motif of the *inhA* gene, repressing its expression; under prothionamide induction, enhanced *inhA* repression potentiates the bactericidal effect of INH on *M. tuberculosis* (Zhu et al., 2018). To further investigate the role of *Rv0274* in INH sensitivity and elucidate the regulatory relationship between *MSMEG_0608* and *MSMEG_0606*, we constructed three strains: complementation strain MSMEG_KO0608::Rv0274, MSMEG_KO0608::0608 and an *MSMEG_0606* knockdown strain (MSMEG_0606_KD) (Figures 4F, G).

MIC determination revealed that both MSMEG_KO0608::Rv0274 and MSMEG_0606_KD exhibited an MIC for INH of 64 μg/mL, substantially higher than those of the wild-type (32 μg/mL) and MSMEG_KO0608 (16 μg/mL) strains (Table 4), confirming that both strains displayed markedly enhanced tolerance. Growth curve analysis further demonstrated that, under INH treatment, both strains exhibited superior growth during the logarithmic phase compared to MSMEG_KO0608, although later-stage growth of MSMEG_0606_KD was slightly affected by tetracycline supplementation (Figure 4H).

**Table 4:** MIC testing results for isoniazid.

**Antibiotic (μg/ml)**	**WT**	**MSMEG_KO0608**	**MSMEG_KO0608::Rv0274**	**MSMEG_0606_KD**
INH	32	16	64	64

Gene expression analysis normalized to sigA revealed that *MSMEG_0606* expression was significantly upregulated in MSMEG_KO0608 compared to the wild-type strain, but was strongly suppressed upon complementation with *Rv0274* and MSMEG_0608 (Figure 4I), confirming the negative regulatory role of *MSMEG_0608* on *MSMEG_0606*. In contrast, *inhA* expression displayed the opposite pattern: it was significantly downregulated in MSMEG_KO0608 but markedly elevated in both MSMEG_KO0608::Rv0274, MSMEG_KO0608::0608 and MSMEG_0606_KD strains (Figure 4J).

Taken together, these findings indicate that *MSMEG_0608* indirectly enhances bacterial tolerance to INH by negatively regulating *MSMEG_0606* and relieving its repression of *inhA*, thereby positively modulating *inhA* expression.

## 4 Discussion

Tuberculosis remains one of the most formidable infectious diseases worldwide, with persistently high incidence and mortality rates posing a substantial public health burden. The widespread use of antibiotics has accelerated the emergence of drug-resistant tuberculosis, further complicating disease management. Chronic infections caused by *M. tuberculosis* are sustained by a complex genetic regulatory network involving secondary metabolism, cell wall synthesis, stress responses, and signal transduction pathways (Clark-Curtiss and Haydel, 2003). Moreover, transcriptional regulation and biofilm formation are increasingly recognized as key contributors to antibiotic tolerance; however, their precise roles in tuberculosis pathogenesis remain to be fully elucidated.

Bioinformatics analyses identified *Rv0274* as a putative member of the aldehyde dehydrogenase family, which is highly conserved across mycobacterial species. Interestingly, aldehyde dehydrogenase family genes in *M. tuberculosis* share three conserved motifs, suggesting their potential involvement in the metabolism of methylglyoxal or related compounds. In this study, we successfully constructed an *MSMEG_0608* knockout strain in *M. smegmatis* using a homologous recombination strategy. Under nutrient-rich conditions, the growth rate and sliding motility of MSMEG_KO0608 were comparable to those of the wild-type strain, indicating that *MSMEG_0608* is non-essential for bacterial survival under such conditions. Biofilm formation, a phenotype closely associated with both mycobacterial growth and tolerance to environmental stress (Richards and Ojha, 2014), was similarly unaffected by the deletion of *MSMEG_0608*. Although biofilm formation in *M. smegmatis* is influenced by multiple factors, including genetic changes affecting lipid components such as free mycolic acids (FMA), mycolic acid diesters (MDAG), monothiolated mycolic acid diesters (MMDAG), mycolic acid wax esters (MWE), and glycopeptidolipids (GPL) (Nisbett et al., 2023), single-gene deletion of *MSMEG_0608* did not significantly alter biofilm development under standard culture conditions. Although the biofilm and motility assays did not reveal significant differences between the wild-type and MSMEG_KO0608 strains, these negative results provide valuable insight into the functional specificity of *MSMEG_0608*. The absence of phenotypic changes under non-stress conditions suggests that *MSMEG_0608* does not function as a broad stress-response or surface-regulation factor. Instead, its effects appear to be selectively activated under INH-induced stress, consistent with its proposed role in modulating inhA expression and INH tolerance. This specificity further supports the conclusion that *MSMEG_0608* participates in targeted redox- and drug-related regulatory pathways rather than in general physiological adaptation.

Our findings further revealed that the *Rv0274* gene in *M. tuberculosis* provides protection against hydrazine-induced stress but exhibits limited responsiveness to other forms of oxidative stress, such as redox-cycling radicals and organic peroxides. Given that isoniazid (INH) is a derivative of hydrazine, we hypothesized that *Rv0274* might also contribute to INH tolerance. Integrating bioinformatics predictions with drug susceptibility assays, our results confirmed that *Rv0274* functions as an INH tolerance-associated gene.

It is well established that *katG* and *inhA* are key genes involved in the regulation of INH activity (Unissa et al., 2016). The *inhA* gene encodes InhA, a pivotal enzyme in mycolic acid biosynthesis and the direct target of activated INH. *In vitro* studies have demonstrated that, under low INH concentrations, promoter mutations in *inhA* (e.g., fabG1–15C>T) represent the predominant tolerance mechanism by increasing InhA protein levels to neutralize active INH. At higher INH concentrations, mutations in *katG* (e.g., *katG* S315N, *katG* N138S, and *katG* K414N) become the dominant mechanism, leading to reduced or abolished *KatG* activity and thereby impairing INH activation. Adaptive shifts in tolerance strategies have also been observed: at elevated INH concentrations, the frequency of *inhA* promoter mutations decreases, whereas *katG* mutations become more prevalent. Furthermore, compensatory mutations in the *ahpC* gene may arise to offset the functional loss of *KatG* (Dokrungkoon et al., 2023).

Our study demonstrates a positive regulatory relationship between *MSMEG_0608* and the inhA gene. In the absence of drug treatment, deletion of *MSMEG_0608* led to widespread upregulation of numerous genes, suggesting that this transcriptional activation may represent a compensatory response to partial physiological deficits caused by the loss of *MSMEG_0608*. Under INH treatment, *MSMEG_0606* expression was markedly increased, and a negative regulatory interaction between *MSMEG_0608* and *MSMEG_0606* was observed; elevated *MSMEG_0606* levels may suppress the expression or function of *MSMEG_0608*, thereby attenuating its positive regulatory effect on inhA. Previous studies have reported that *Rv0273c*, the homolog of *MSMEG_0606*, encodes the transcriptional regulator EtbR, which specifically binds to an upstream motif of inhA and represses its transcription (Zhu et al., 2018). Consistent with this model, complementation of *Rv0274* in the MSMEG_KO0608 strain and knockdown of *MSMEG_0606* both confirmed that *MSMEG_0608* functions as a key regulatory factor: by indirectly inhibiting *MSMEG_0606* expression, it relieves the repression of inhA, thereby upregulating inhA expression and enhancing bacterial tolerance to INH.

It should be noted that the deletion of *MSMEG_0608* may exert a certain degree of compensatory transcriptional influence on adjacent genes within the same genomic region, particularly *MSMEG_0606*, even under basal conditions. However, the difference becomes more pronounced under INH treatment, suggesting that the observed transcriptional changes are primarily stress-induced rather than due to a simple polar effect. This implies that *MSMEG_0608* and *MSMEG_0606* may participate in a coordinated regulatory network that responds to drug-induced stress, rather than being functionally linked through an operonic structure. To further exclude a classical polar effect caused by gene deletion, we performed transcriptional-strand and operon-prediction analysis (Operon-mapper). The results showed that *MSMEG_0608* is located on the opposite strand and is not predicted to form a single operon with *MSMEG_0604-0606*, supporting a regulatory mechanism rather than operon disruption. These data are provided in Supplementary Table.

Aldehyde dehydrogenases are essential for cellular and bacterial survival, yet their roles in mycobacteria remain poorly understood. As a predicted member of the aldehyde dehydrogenase family, *Rv0274* shares conserved structural domains with orthologous genes across mycobacterial species. For example, the putative glyoxalase II enzyme MSMEG_2975 has been reported to influence bacterial growth, biofilm formation, transcriptomic profiles, and antibiotic susceptibility (Haris et al., 2021). Similarly, our findings demonstrate that *MSMEG_0608* modulates global transcriptional patterns, regulates INH sensitivity, and affects the expression of tolerance-related genes. These results suggest that aldehyde dehydrogenase family genes may represent promising therapeutic targets for combating mycobacterial pathogens. Despite recent advances linking specific mutations in tolerance-associated genes to INH tolerance, the underlying molecular mechanisms remain incompletely understood. Our experiments, conducted using an *M. smegmatis* model, require validation of *Rv0274*′s function in *M. tuberculosis*. Future studies should elucidate how *Rv0274* regulates the expression of *Rv0273c* and *inhA*, and assess the impact of altered mycolic acid content on cell wall integrity among the different strains. A key limitation of this study is the use of *M. smegmatis* as a surrogate model; the results have not yet been validated in *M. tuberculosis*. Although *M. smegmatis* exhibits higher intrinsic tolerance to isoniazid than *M. tuberculosis*, largely due to *KatG* variants with reduced INH-activating efficiency (Reingewertz et al., 2020), it remains a reliable and mechanistically informative surrogate for dissecting *inhA*-mediated resistance pathways. The *inhA* gene and its regulatory network—including *Rv0273c/MSMEG_0606*-mediated transcriptional repression—are highly conserved across mycobacterial species (Zhu et al., 2018). Therefore, the relative changes in INH susceptibility observed in the MSMEG_KO0608 and complemented strains provide mechanistic insights relevant to the *Rv0274–Rv0273c–inhA* regulatory axis in *M. tuberculosis*. Future studies will construct *Rv0274* knockout and overexpression strains in *M. tuberculosis* H37Rv to confirm its role in INH tolerance and its regulatory interactions with *Rv0273c* and inhA.

## Data Availability

The processed RNA-seq datasets generated and analyzed in this study are publicly available in Zenodo at: https://doi.org/10.5281/zenodo.17636367.
