# Supplementary figures and images for: Decoding drug tolerance: insights into the Rv0274 gene's role in isoniazid tolerance
Source: Front Microbiol. 2025 Nov 27;16:1697416. doi: 10.3389/fmicb.2025.1697416 (PMC12695796; doi:10.3389/fmicb.2025.1697416)

**R : 0.6204 Significance : 0.006**

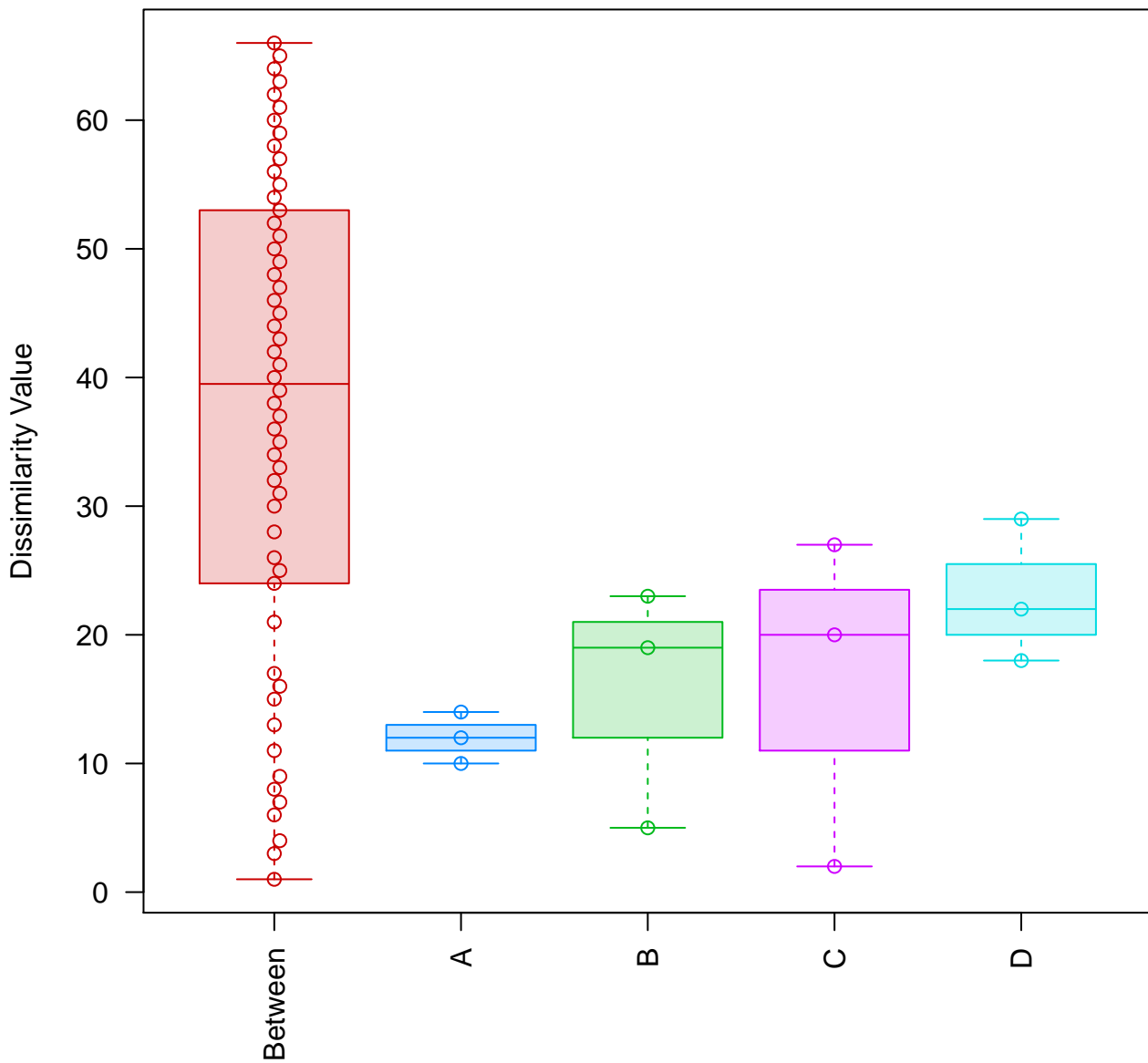

Supplement: Supplementary file 1 [file Supplementary_file_1.zip › RNA_seq_expression/anosim/gene_tpm_anosim_boxplot.pdf]

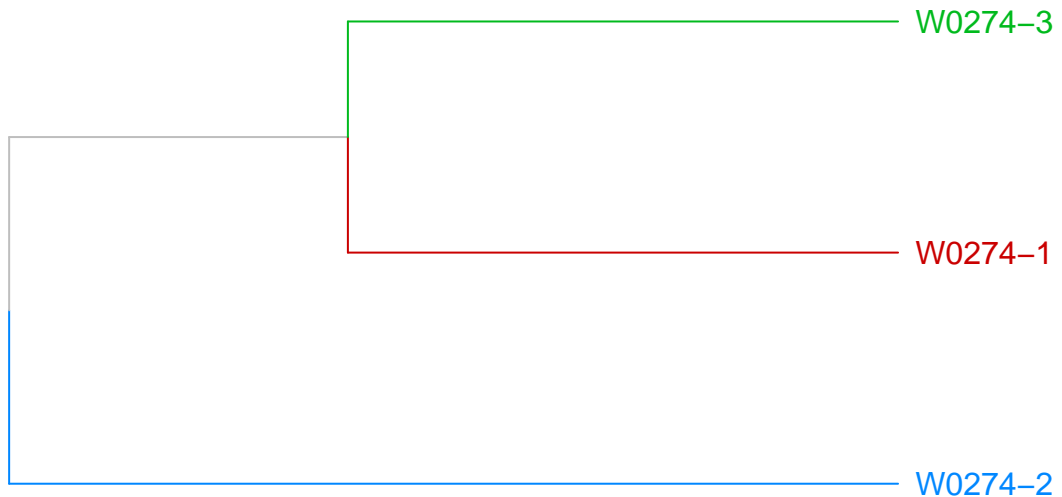

0.1

Supplement: Supplementary file 1 [file Supplementary_file_1.zip › RNA_seq_expression/bray_tree/A.gene_tpm_bray_tree.pdf]

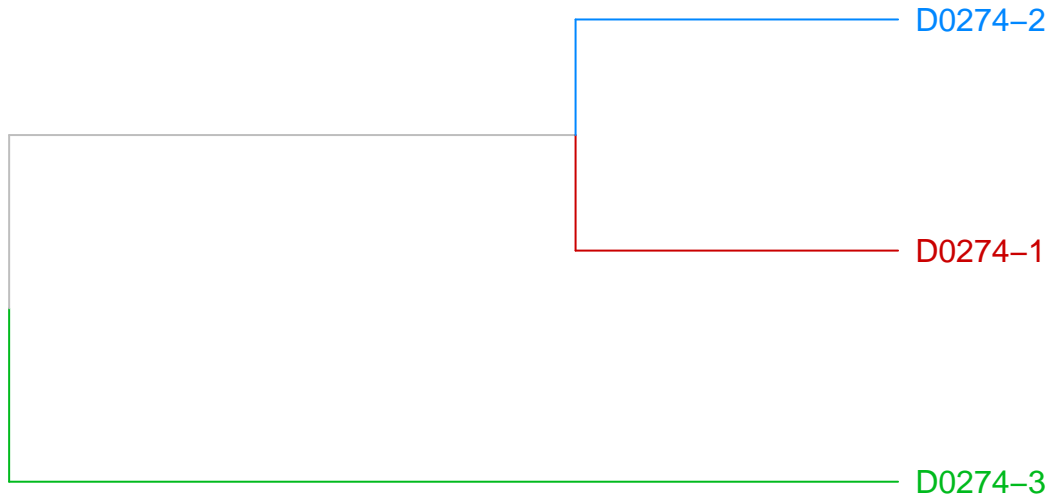

0.1

Supplement: Supplementary file 1 [file Supplementary_file_1.zip › RNA_seq_expression/bray_tree/B.gene_tpm_bray_tree.pdf]

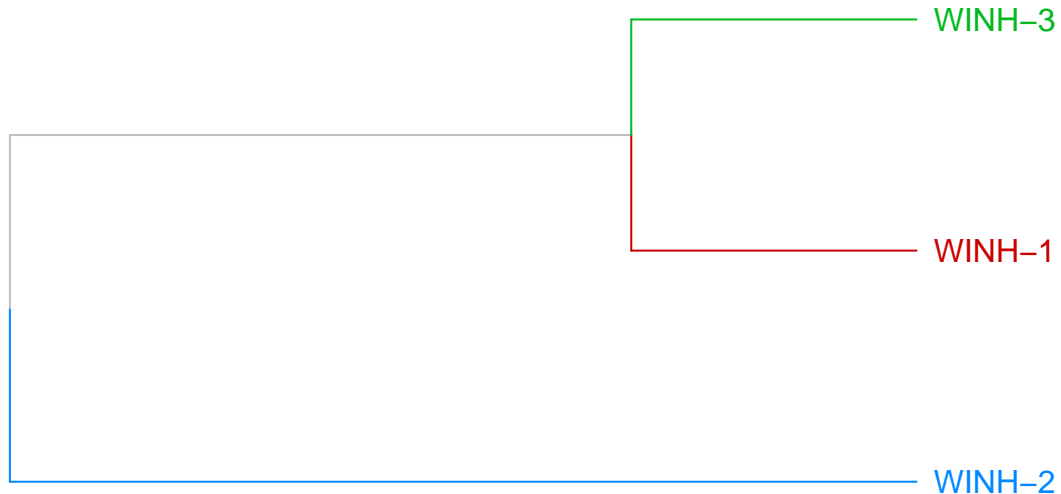

0.1

Supplement: Supplementary file 1 [file Supplementary_file_1.zip › RNA_seq_expression/bray_tree/C.gene_tpm_bray_tree.pdf]

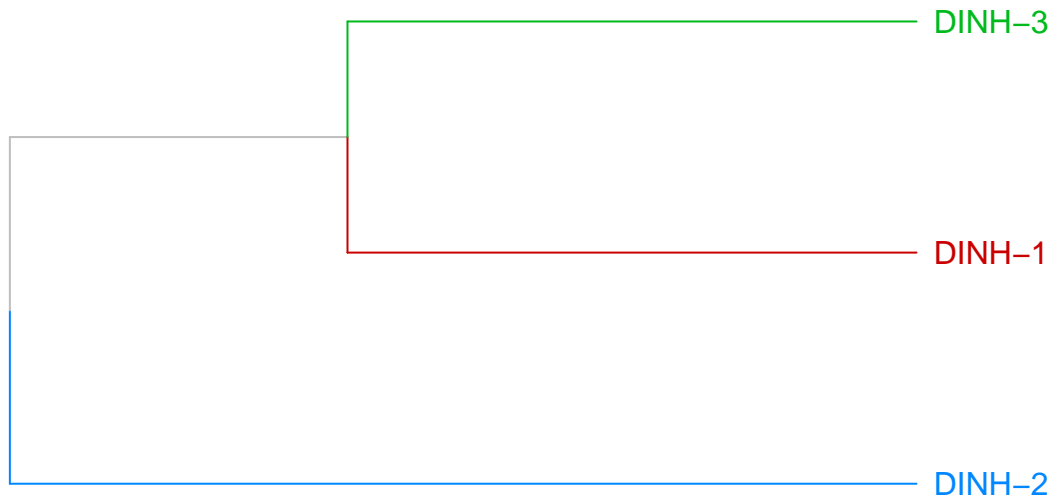

0.1

Supplement: Supplementary file 1 [file Supplementary_file_1.zip › RNA_seq_expression/bray_tree/D.gene_tpm_bray_tree.pdf]

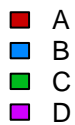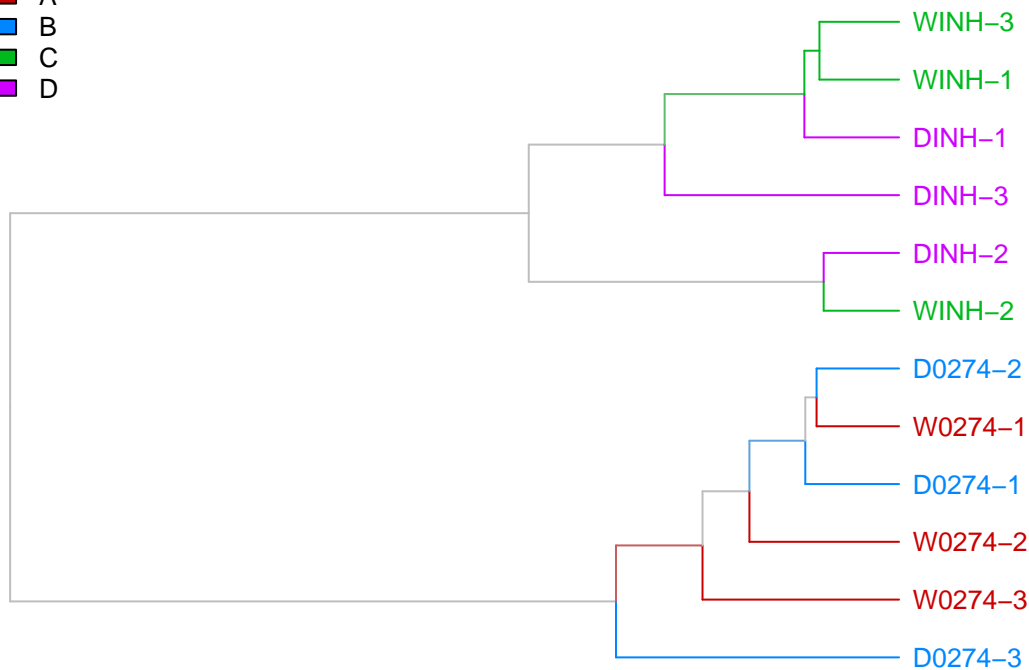

0.1

Supplement: Supplementary file 1 [file Supplementary_file_1.zip › RNA_seq_expression/bray_tree/gene_tpm_bray_tree.pdf]

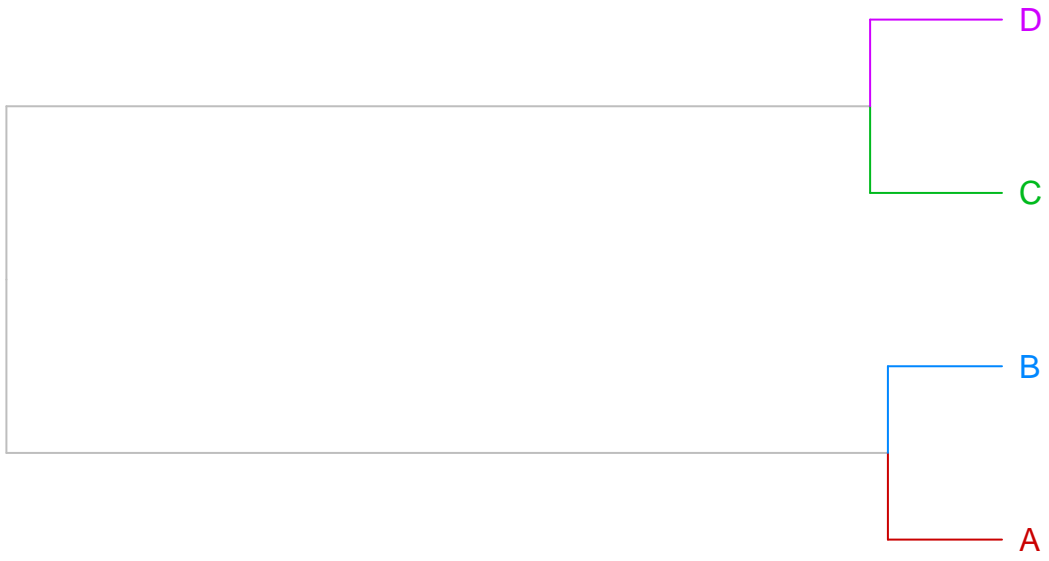

0.1

Supplement: Supplementary file 1 [file Supplementary_file_1.zip › RNA_seq_expression/bray_tree/groupmerge.gene_tpm_bray_tree.pdf]

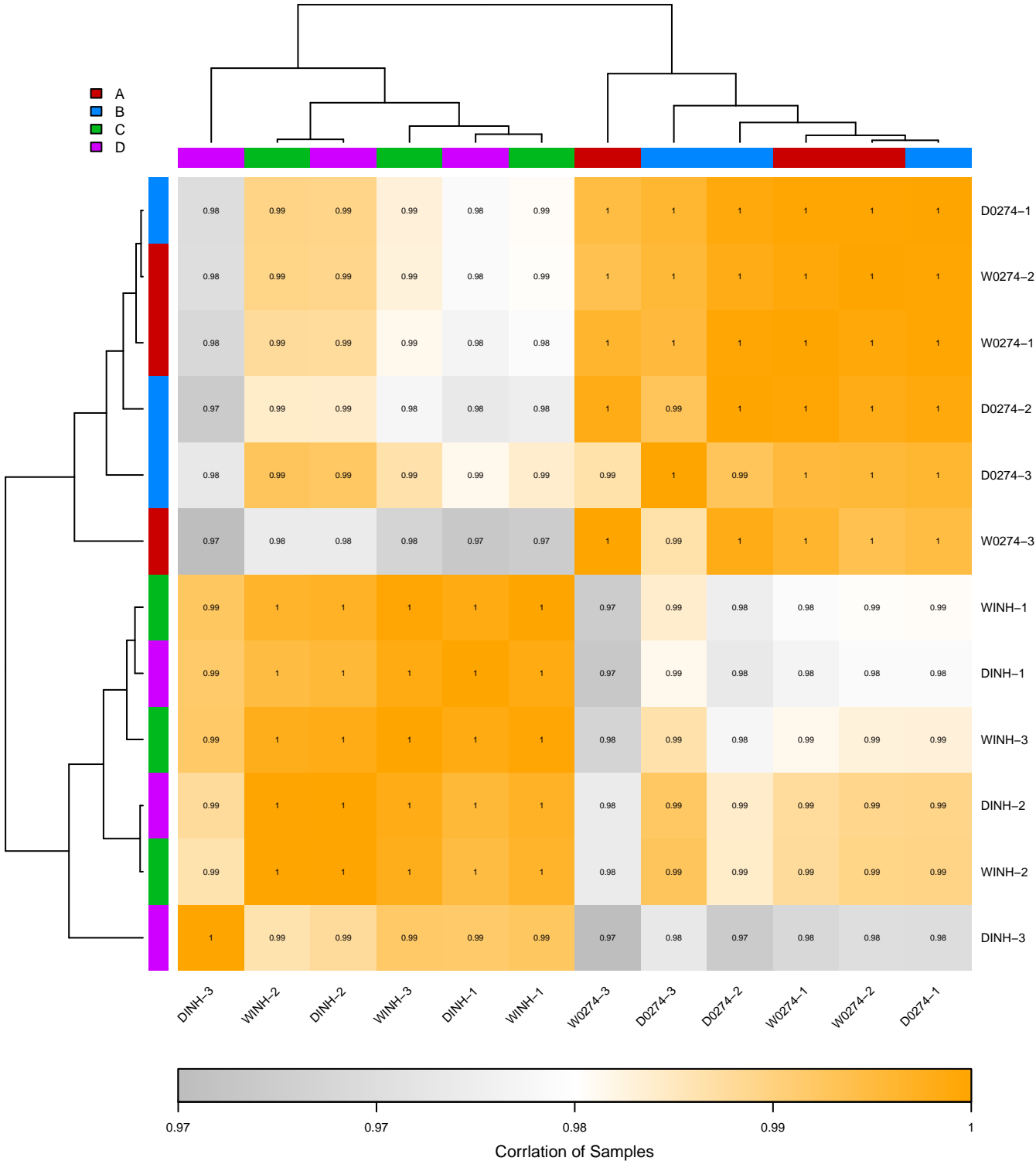

Supplement: Supplementary file 1 [file Supplementary_file_1.zip › RNA_seq_expression/corrlation/gene_tpm_corrlation_heatmap.pdf]

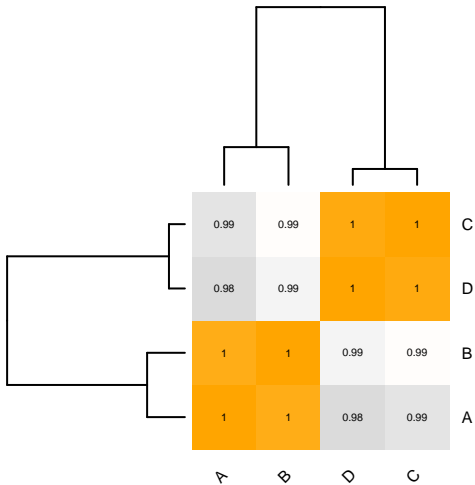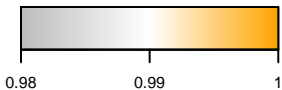

Correlation of Groups

Supplement: Supplementary file 1 [file Supplementary_file_1.zip › RNA_seq_expression/corrlation/groupmerge.gene_tpm_corrlation_heatmap.pdf]

TPM density distribution

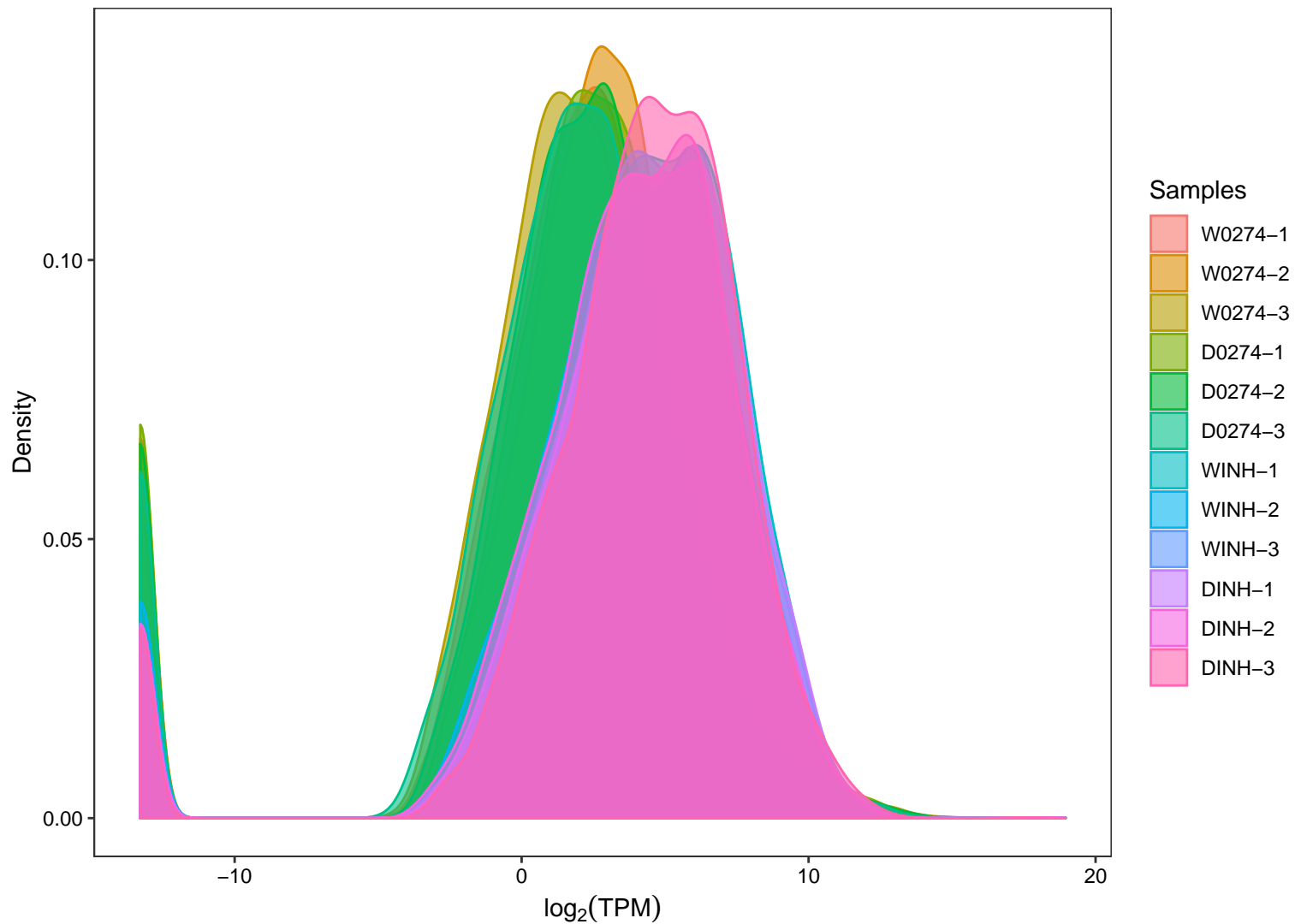

Supplement: Supplementary file 1 [file Supplementary_file_1.zip › RNA_seq_expression/density/gene_tpm_density.pdf]

**Sample Distance boxplot**

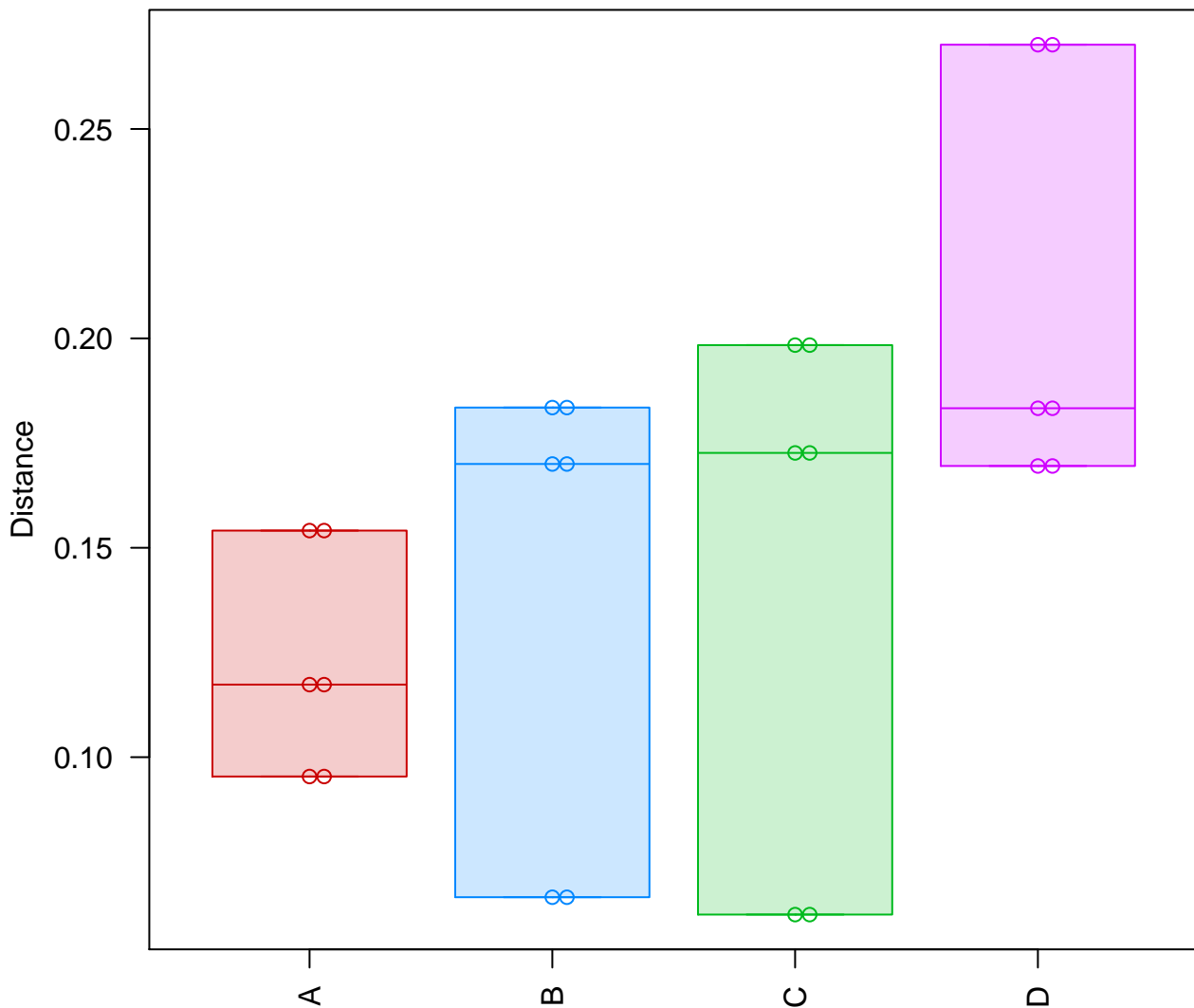

Supplement: Supplementary file 1 [file Supplementary_file_1.zip › RNA_seq_expression/dist_boxplot/gene_tpm_distance_boxplot.pdf]

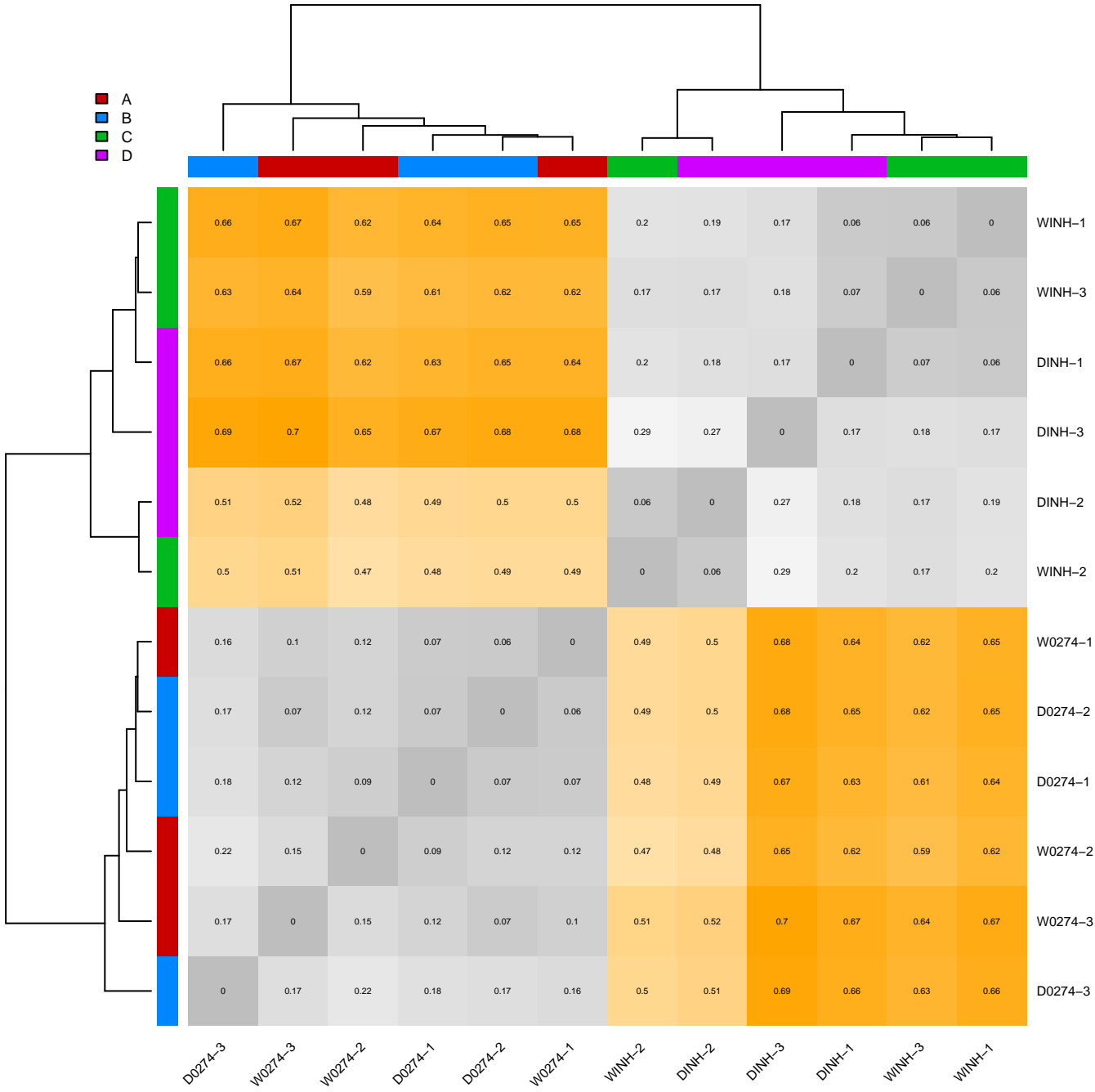

Supplement: Supplementary file 1 [file Supplementary_file_1.zip › RNA_seq_expression/dist_heatmap/gene_tpm_distance_heatmap.pdf]

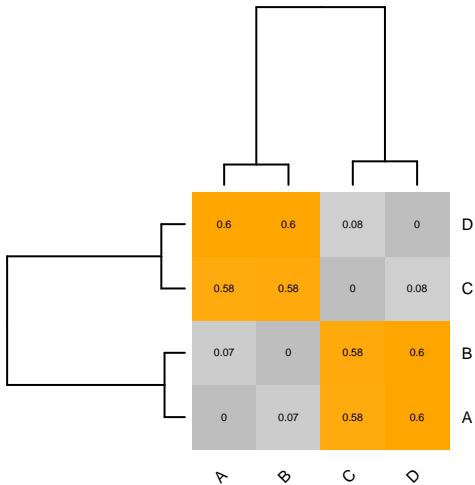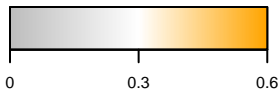

Distance between groups

Supplement: Supplementary file 1 [file Supplementary_file_1.zip › RNA_seq_expression/dist_heatmap/groupmerge.gene_tpm_distance_heatmap.pdf]

Gene Saturation (D0274-1)

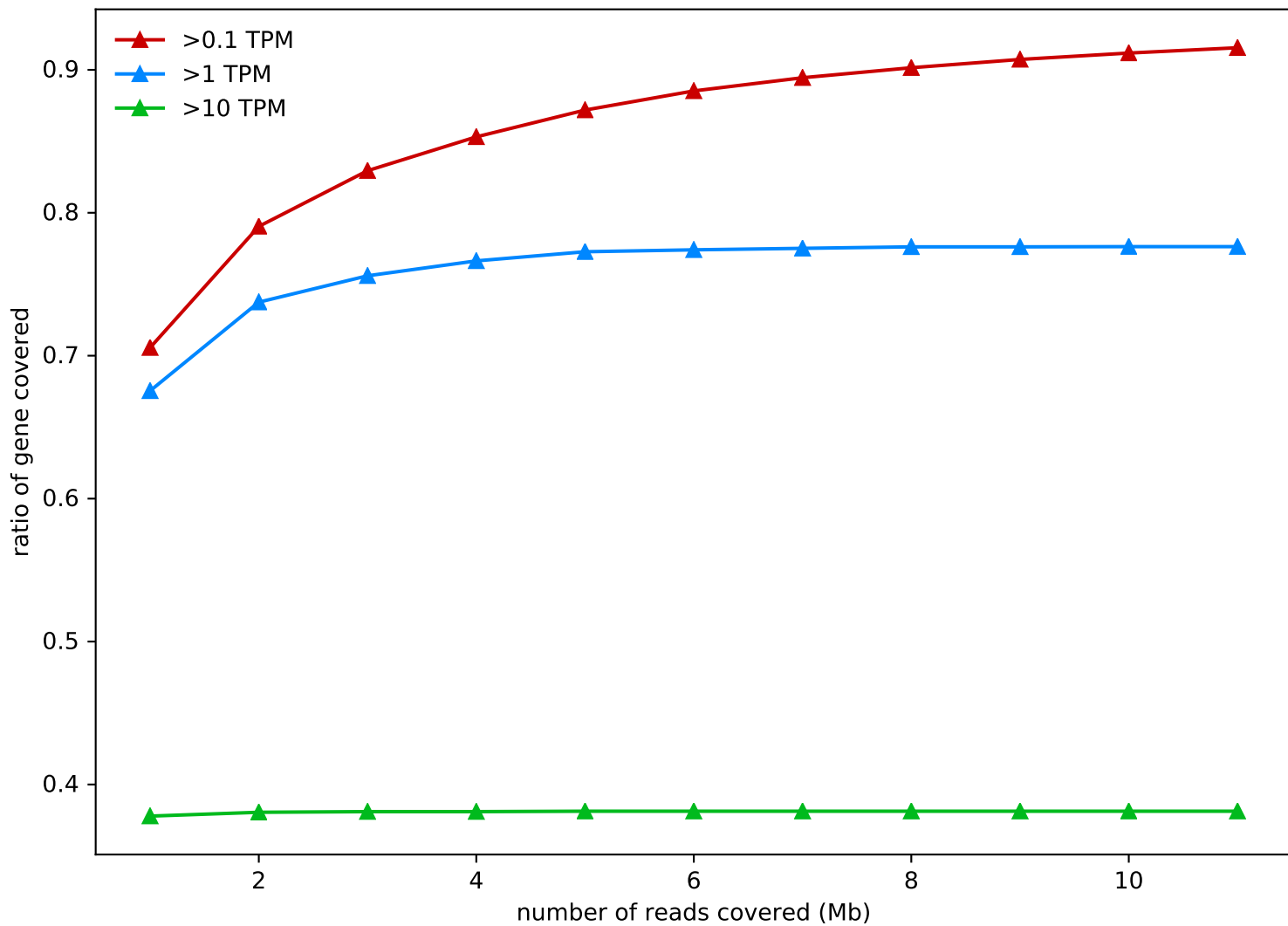

Supplement: Supplementary file 1 [file Supplementary_file_1.zip › RNA_seq_expression/gene_saturation/D0274-1.gene_saturation.pdf]

Gene Saturation (D0274-2)

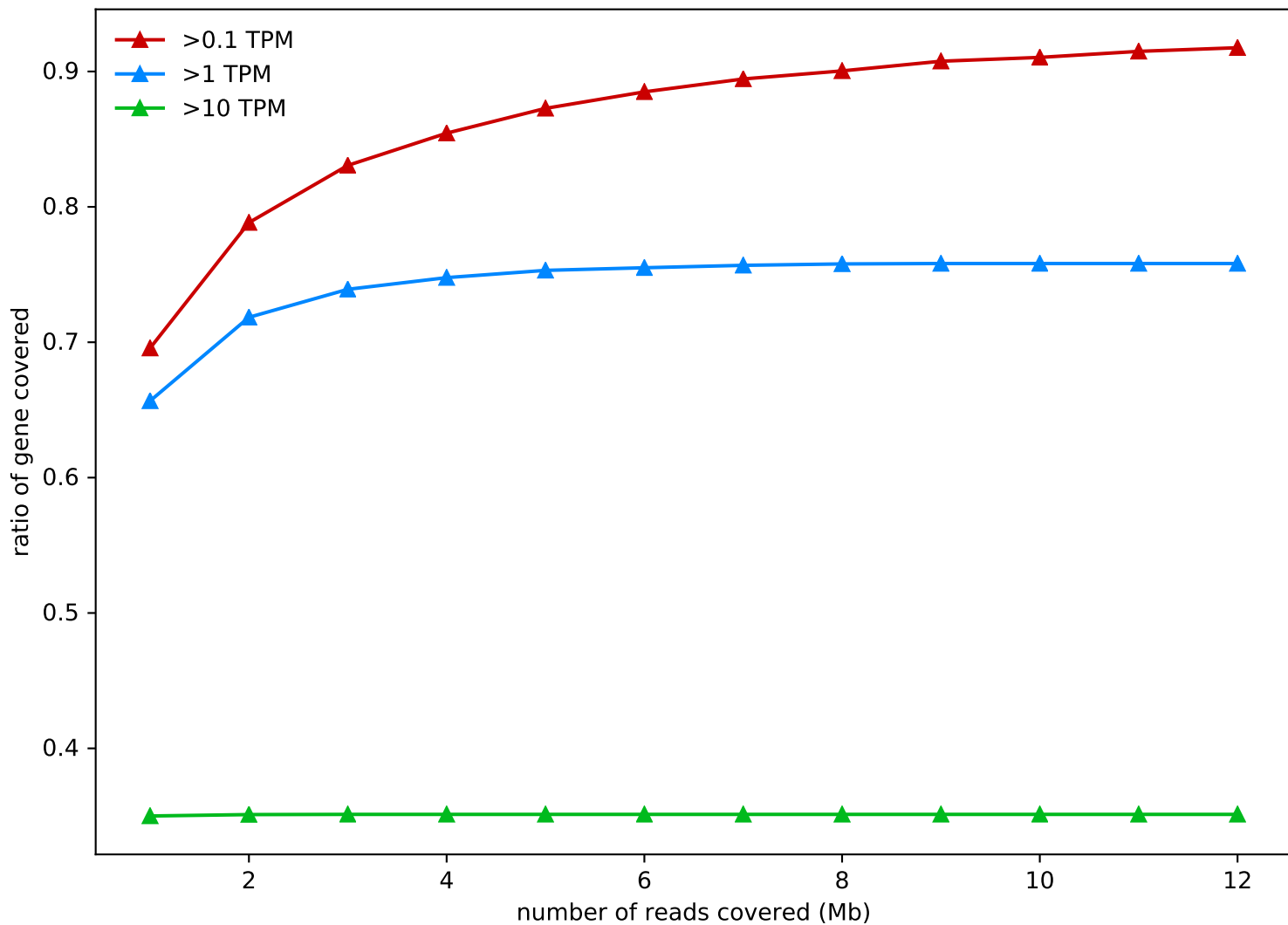

Supplement: Supplementary file 1 [file Supplementary_file_1.zip › RNA_seq_expression/gene_saturation/D0274-2.gene_saturation.pdf]

Gene Saturation (D0274-3)

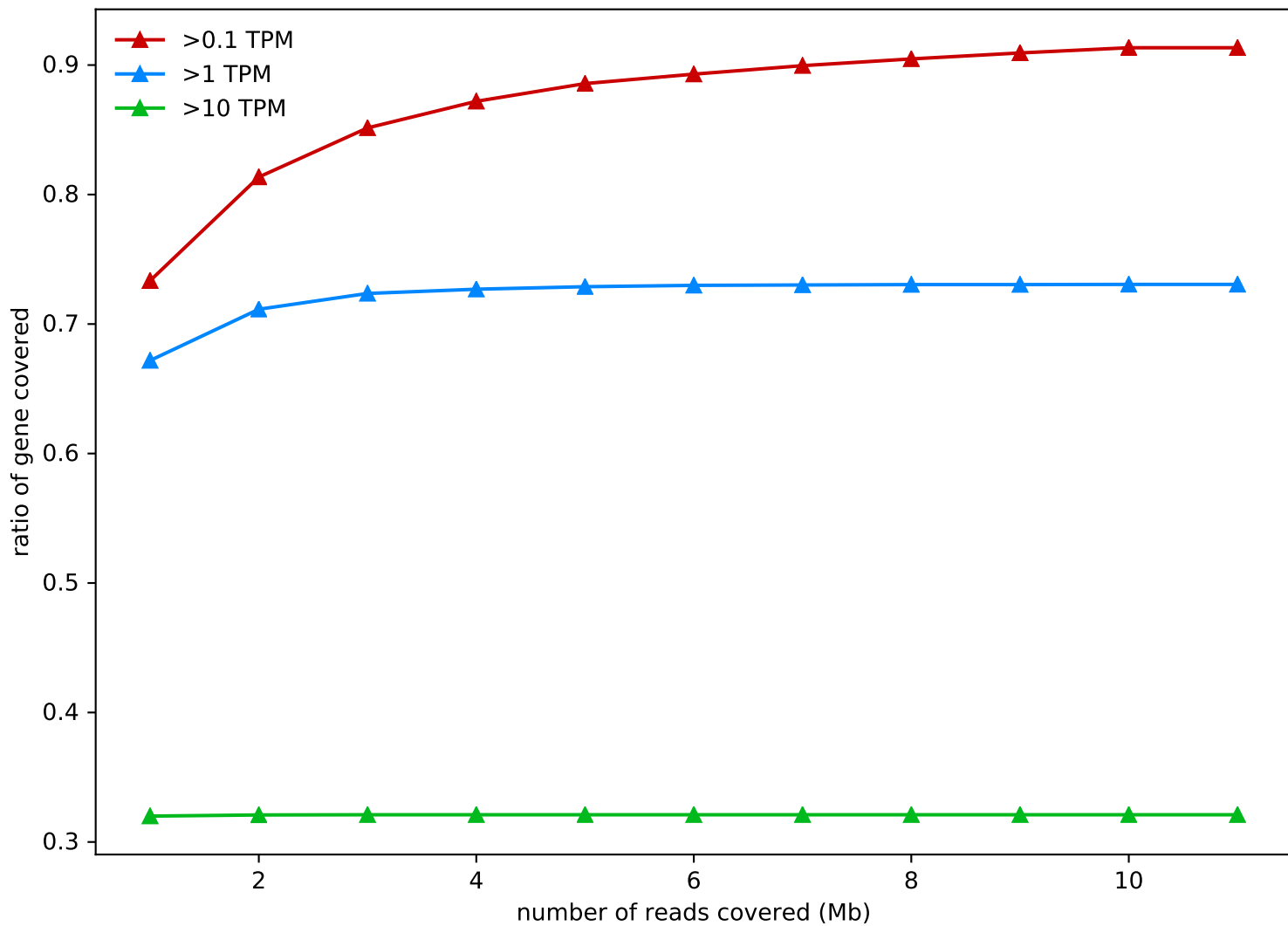

Supplement: Supplementary file 1 [file Supplementary_file_1.zip › RNA_seq_expression/gene_saturation/D0274-3.gene_saturation.pdf]

Gene Saturation (DINH-1)

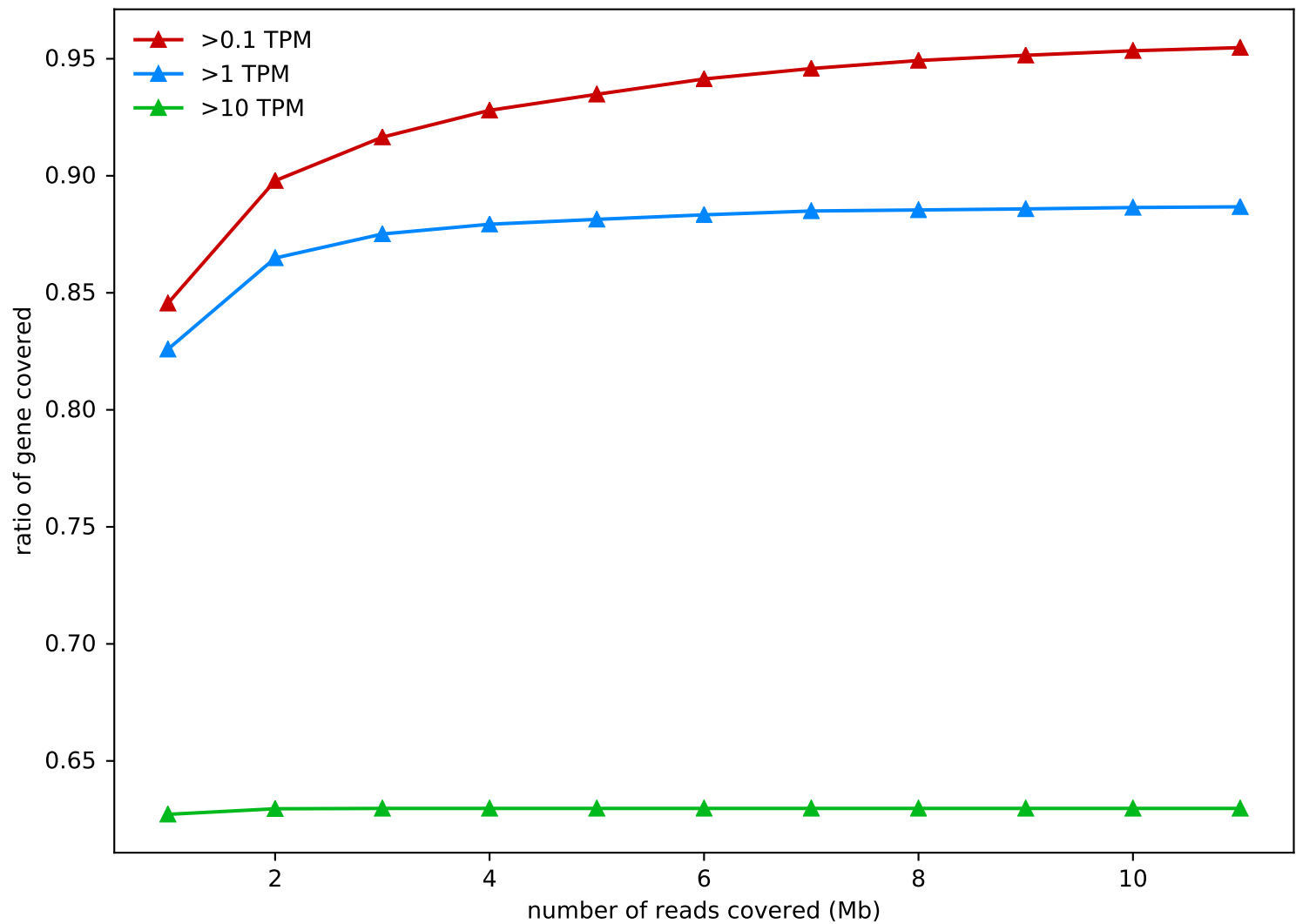

Supplement: Supplementary file 1 [file Supplementary_file_1.zip › RNA_seq_expression/gene_saturation/DINH-1.gene_saturation.pdf]

Gene Saturation (DINH-2)

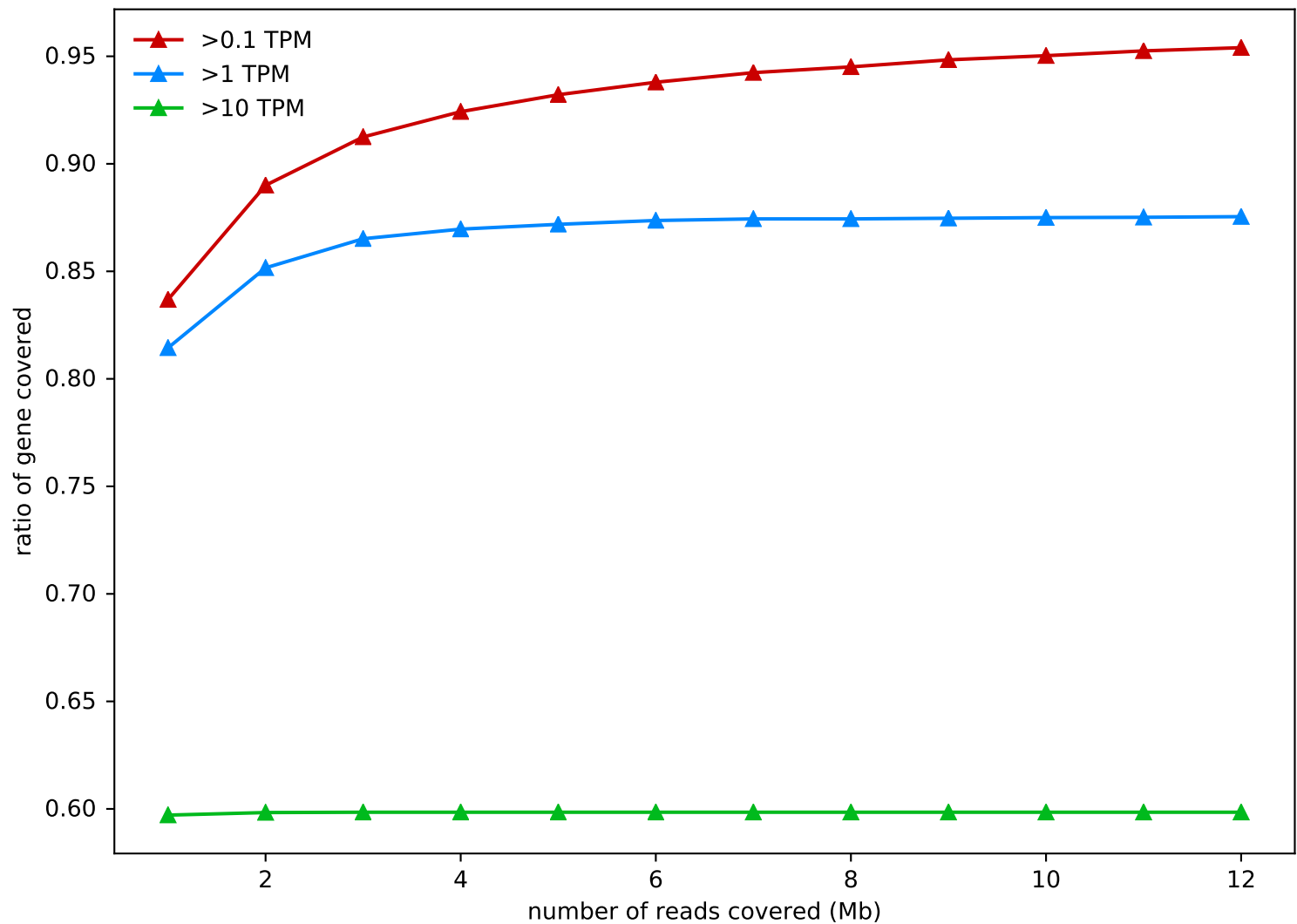

Supplement: Supplementary file 1 [file Supplementary_file_1.zip › RNA_seq_expression/gene_saturation/DINH-2.gene_saturation.pdf]

Gene Saturation (DINH-3)

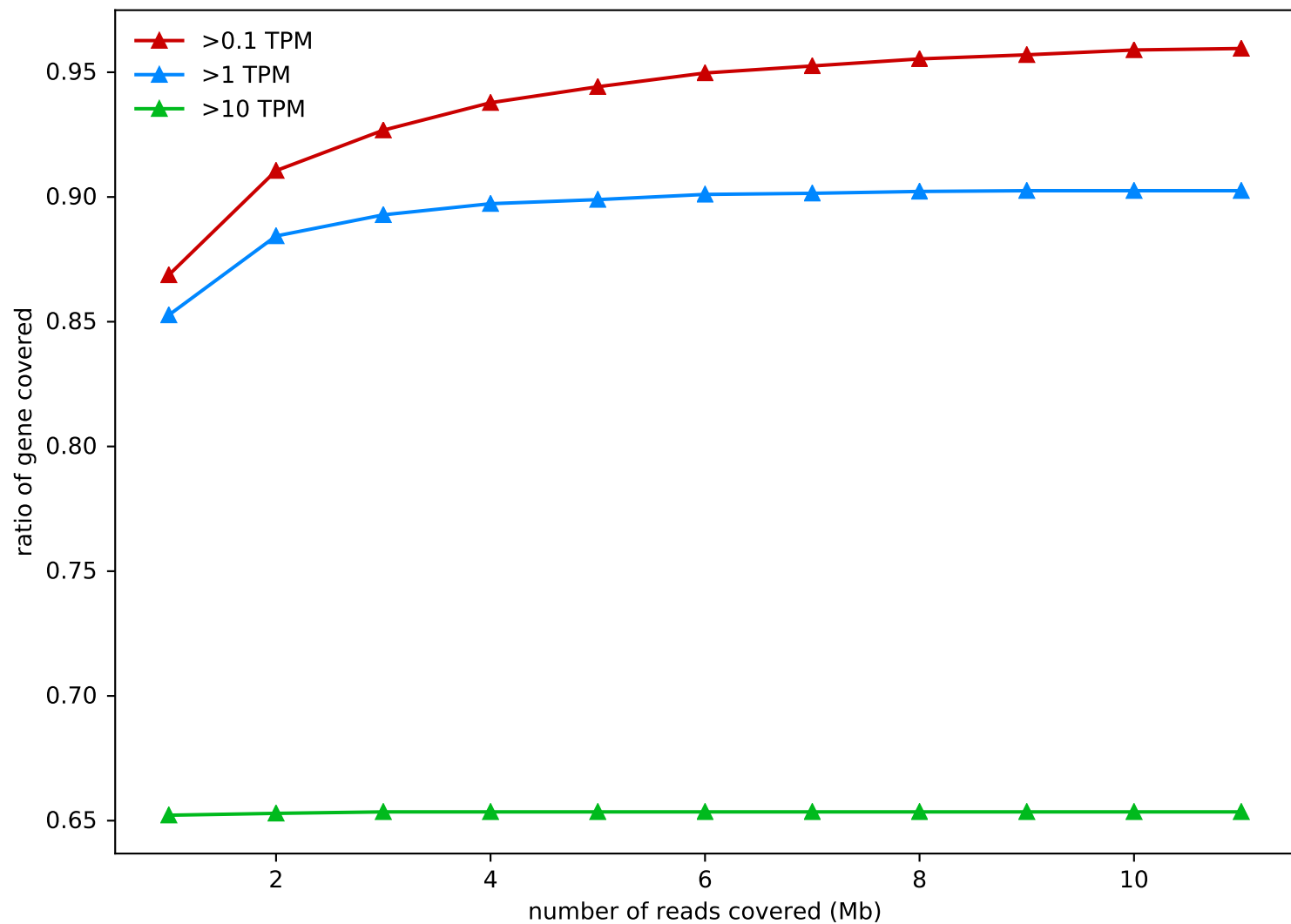

Supplement: Supplementary file 1 [file Supplementary_file_1.zip › RNA_seq_expression/gene_saturation/DINH-3.gene_saturation.pdf]

Gene Saturation (W0274-1)

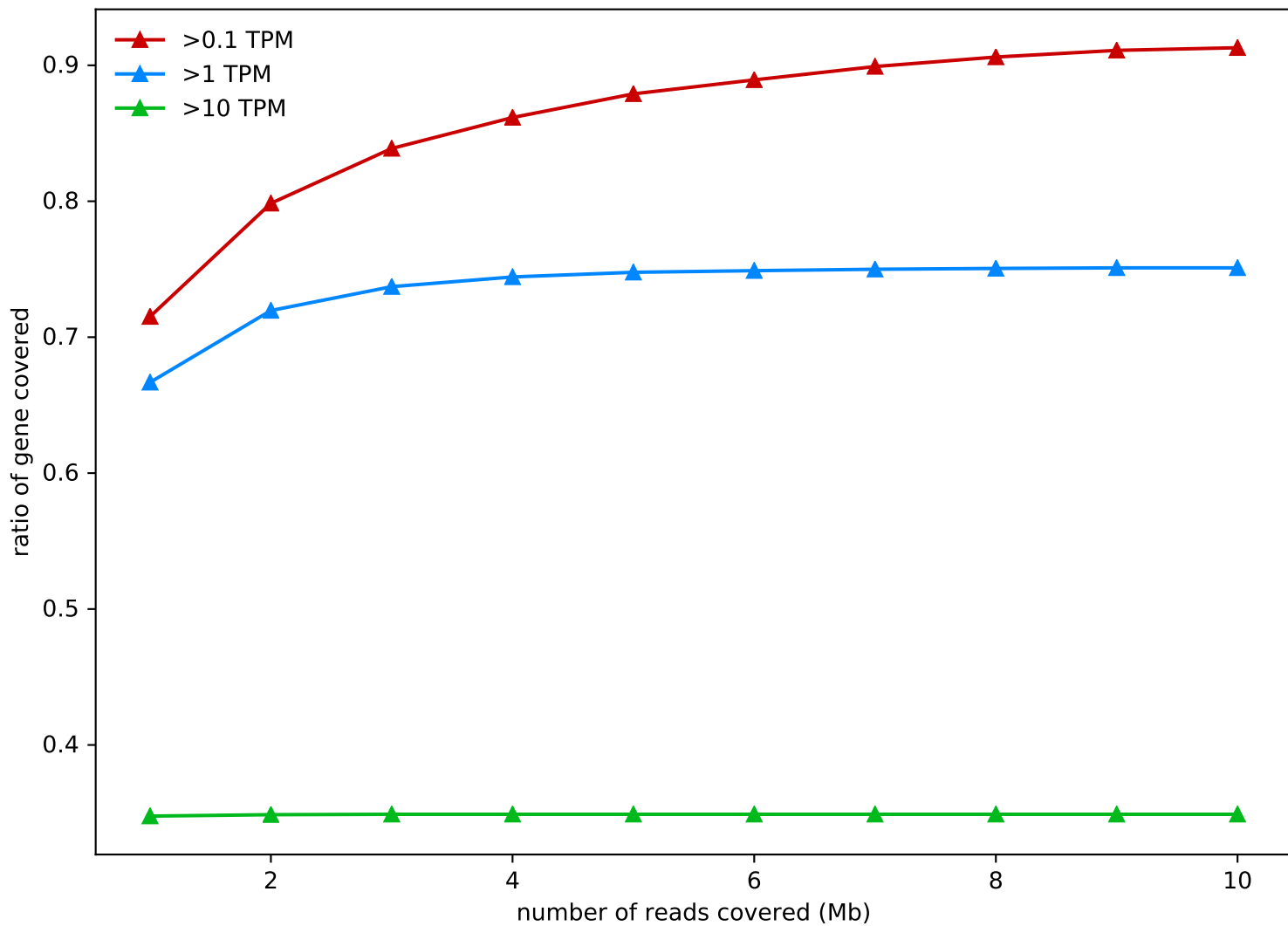

Supplement: Supplementary file 1 [file Supplementary_file_1.zip › RNA_seq_expression/gene_saturation/W0274-1.gene_saturation.pdf]

Gene Saturation (W0274-2)

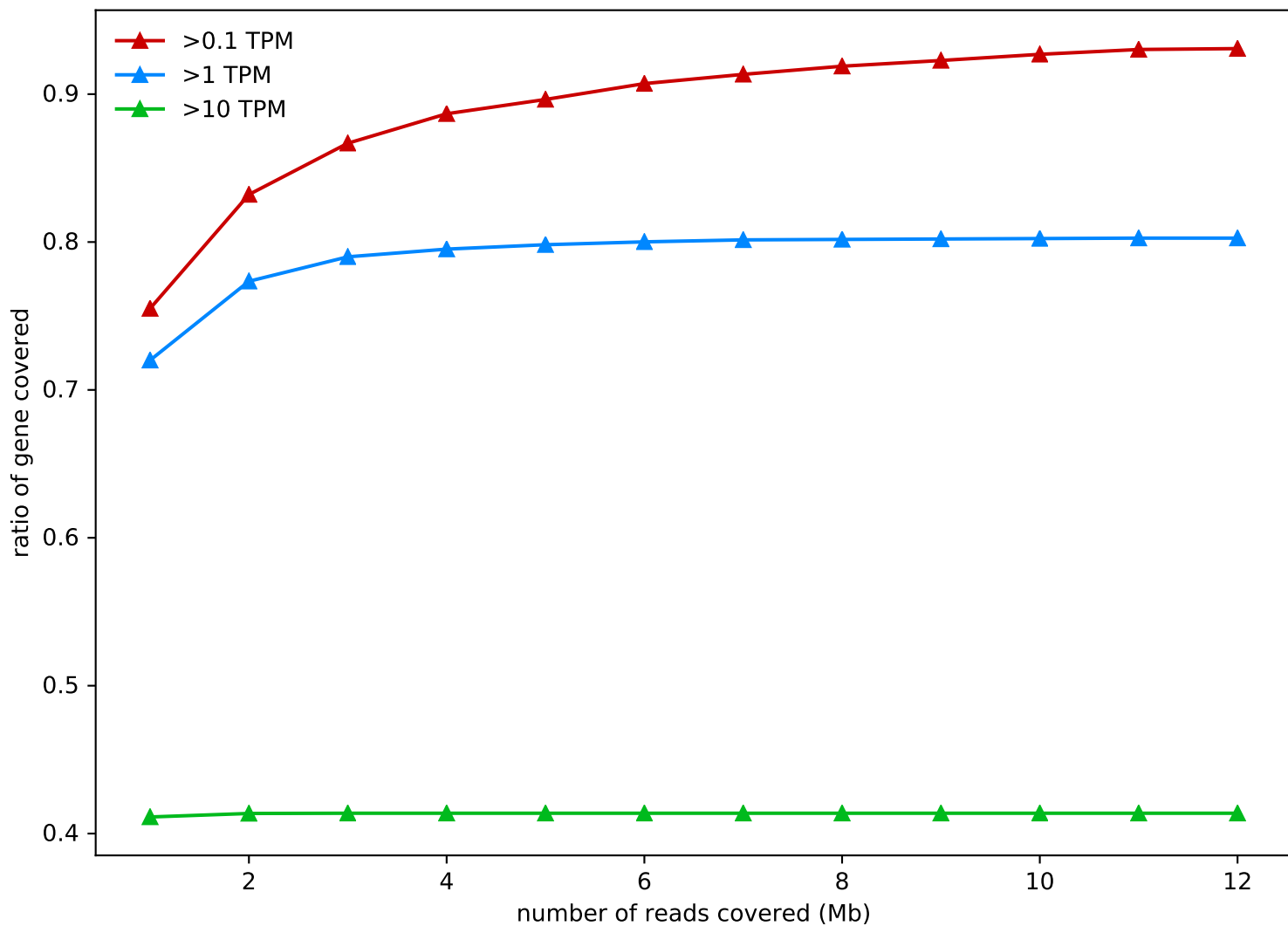

Supplement: Supplementary file 1 [file Supplementary_file_1.zip › RNA_seq_expression/gene_saturation/W0274-2.gene_saturation.pdf]

Gene Saturation (W0274-3)

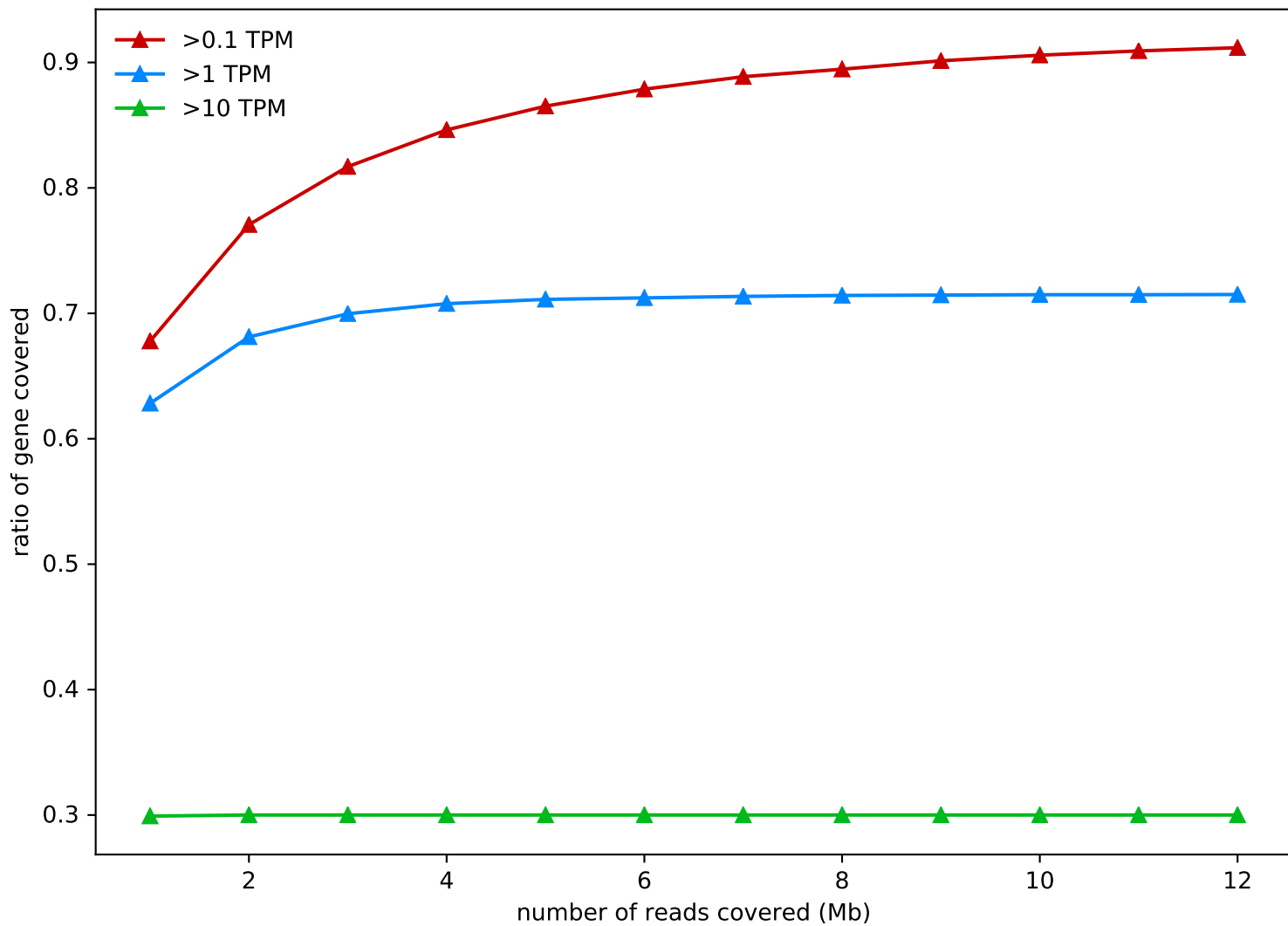

Supplement: Supplementary file 1 [file Supplementary_file_1.zip › RNA_seq_expression/gene_saturation/W0274-3.gene_saturation.pdf]

Gene Saturation (WINH-1)

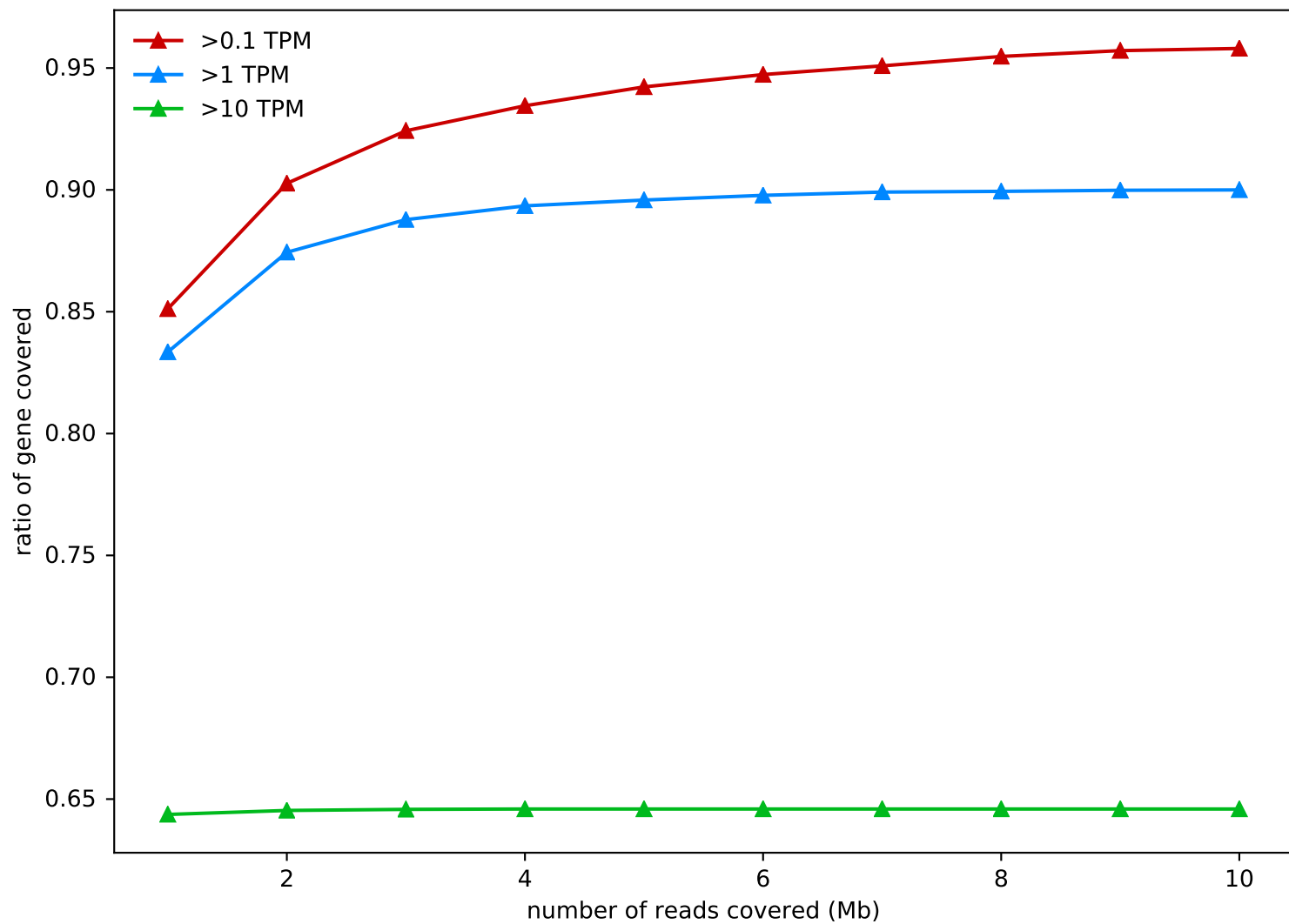

Supplement: Supplementary file 1 [file Supplementary_file_1.zip › RNA_seq_expression/gene_saturation/WINH-1.gene_saturation.pdf]

Gene Saturation (WINH-2)

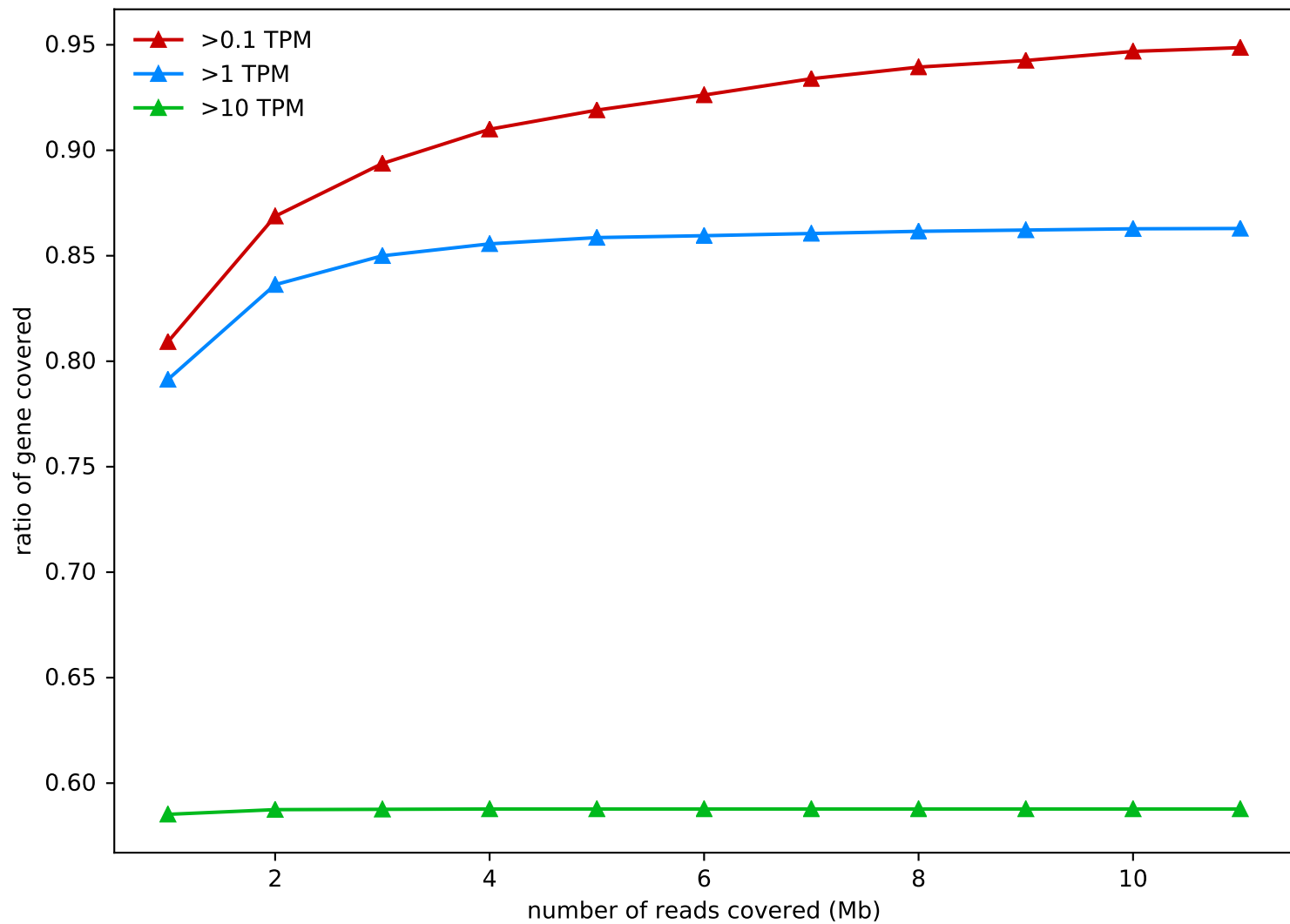

Supplement: Supplementary file 1 [file Supplementary_file_1.zip › RNA_seq_expression/gene_saturation/WINH-2.gene_saturation.pdf]

Gene Saturation (WINH-3)

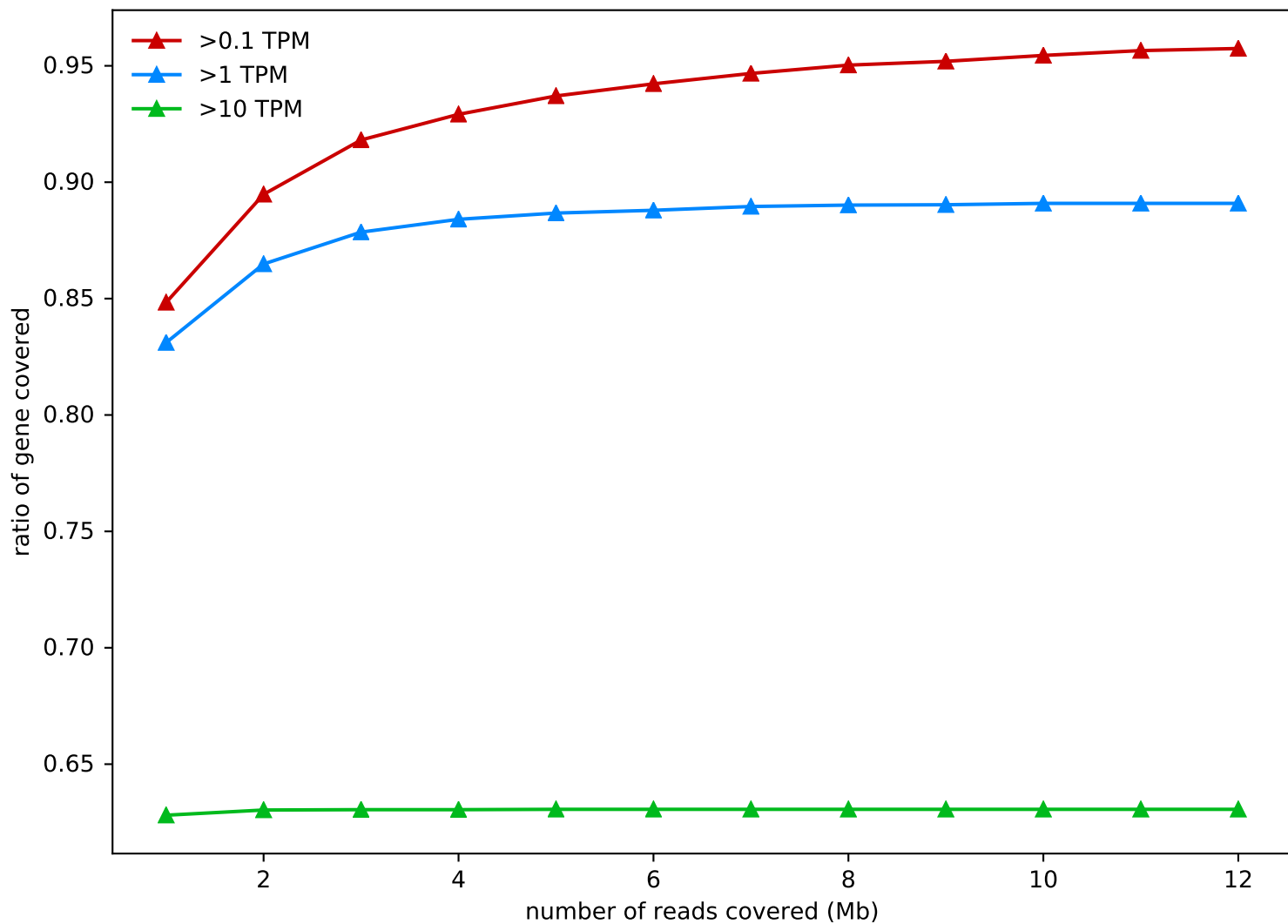

Supplement: Supplementary file 1 [file Supplementary_file_1.zip › RNA_seq_expression/gene_saturation/WINH-3.gene_saturation.pdf]

# NMDS 3D

- A
- B
- ▲ C
- ◆ D

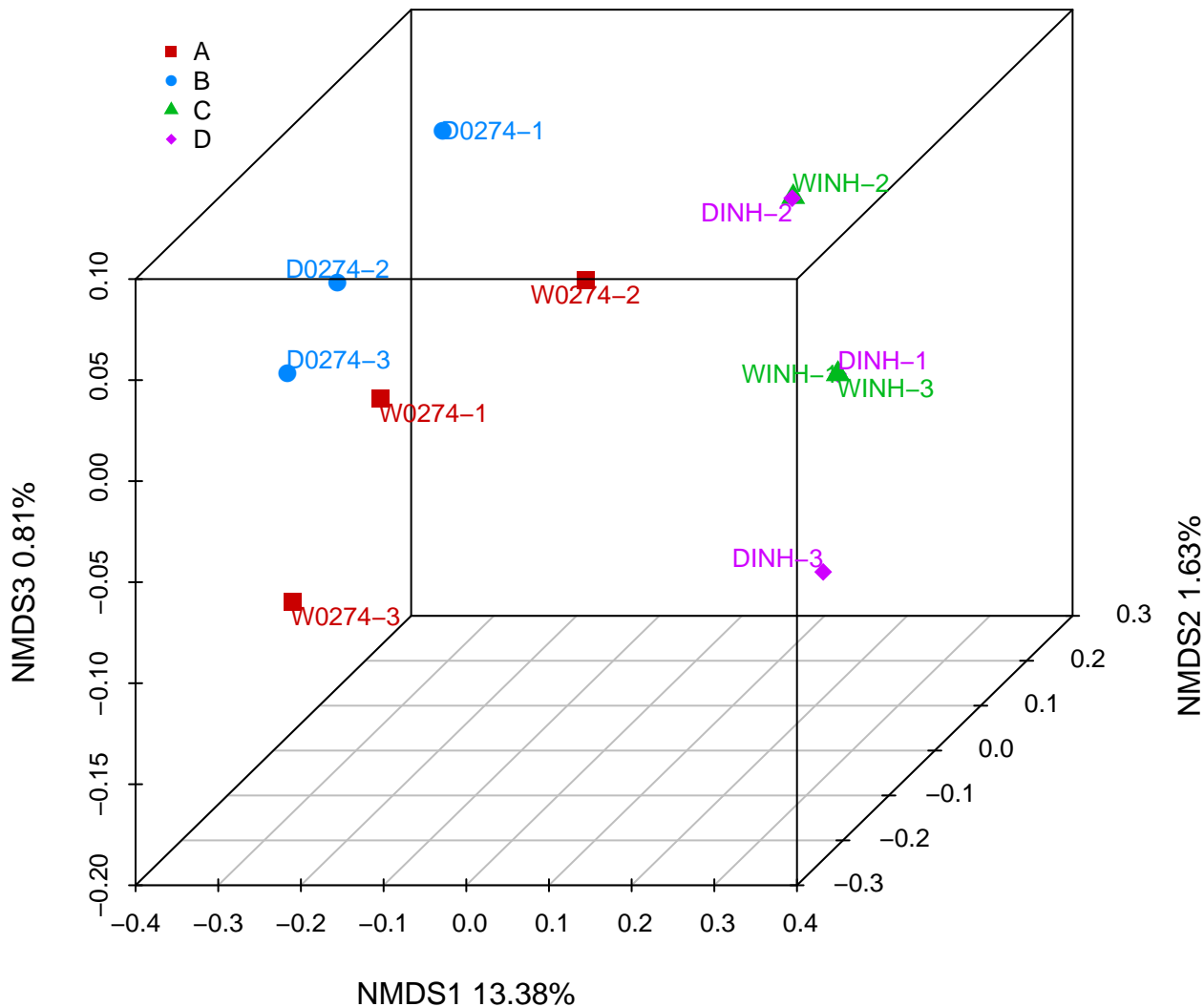

# NMDS – MDS1 vs MDS2

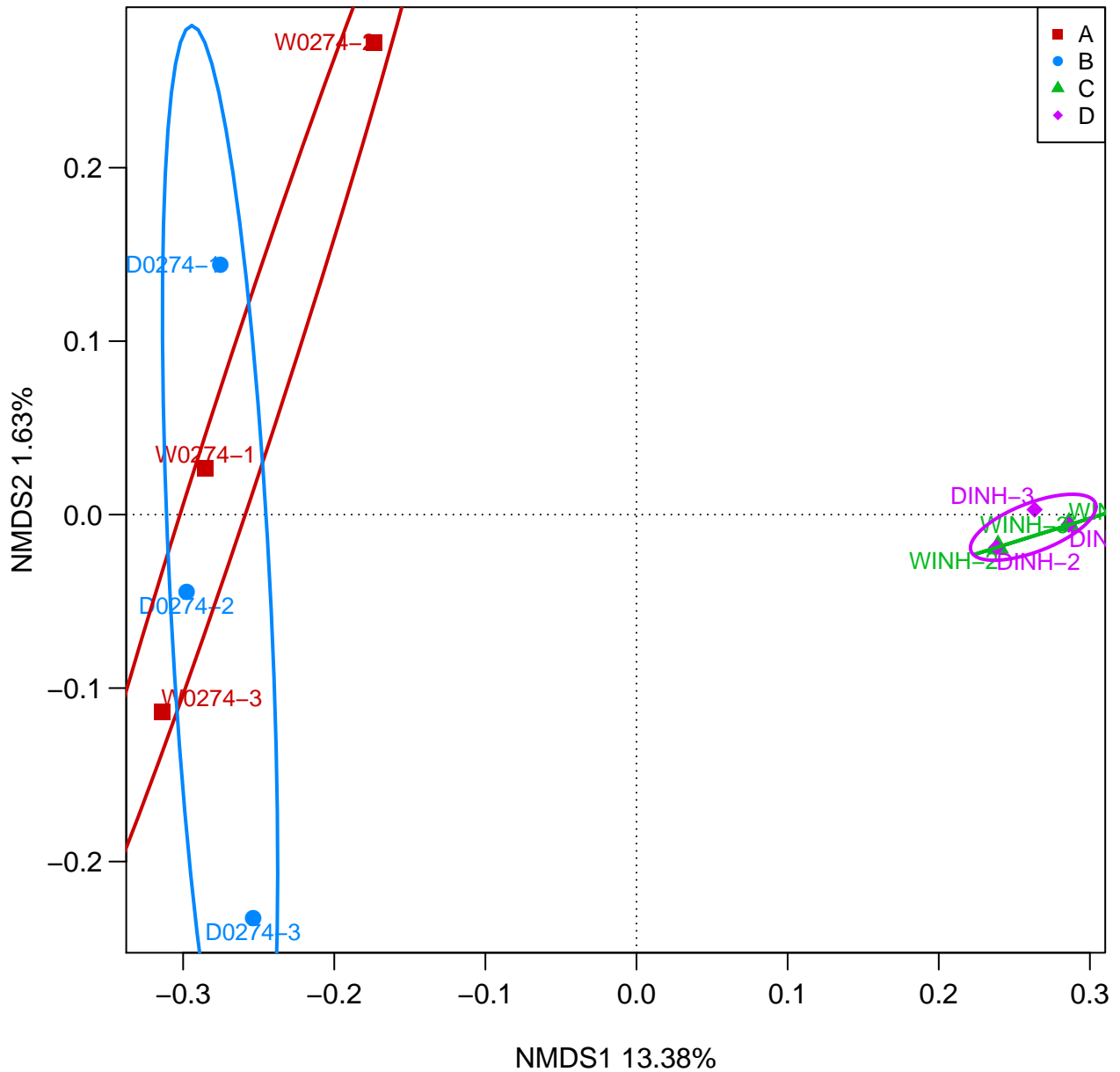

# NMDS – MDS1 vs MDS3

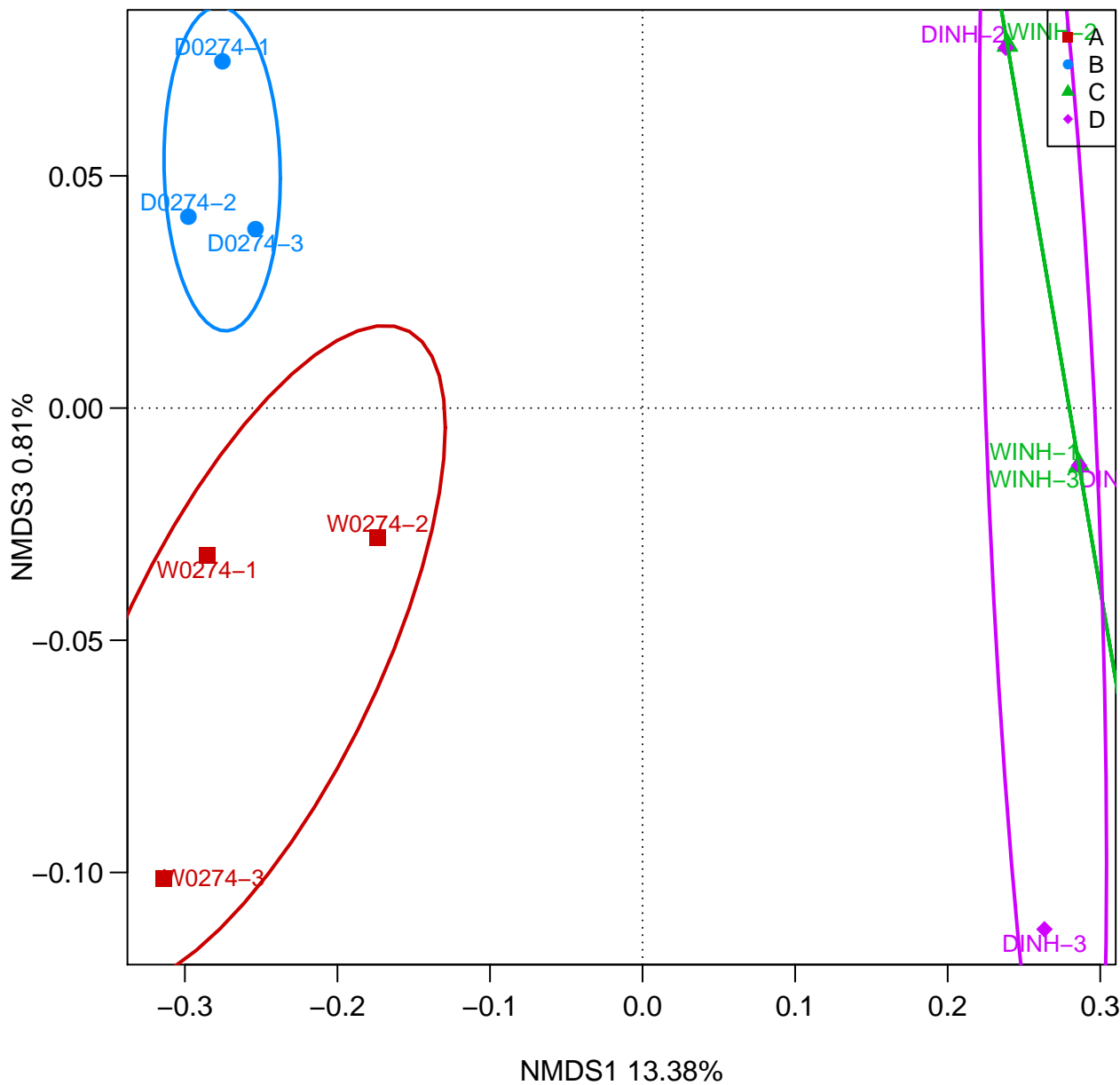

# NMDS – MDS2 vs MDS3

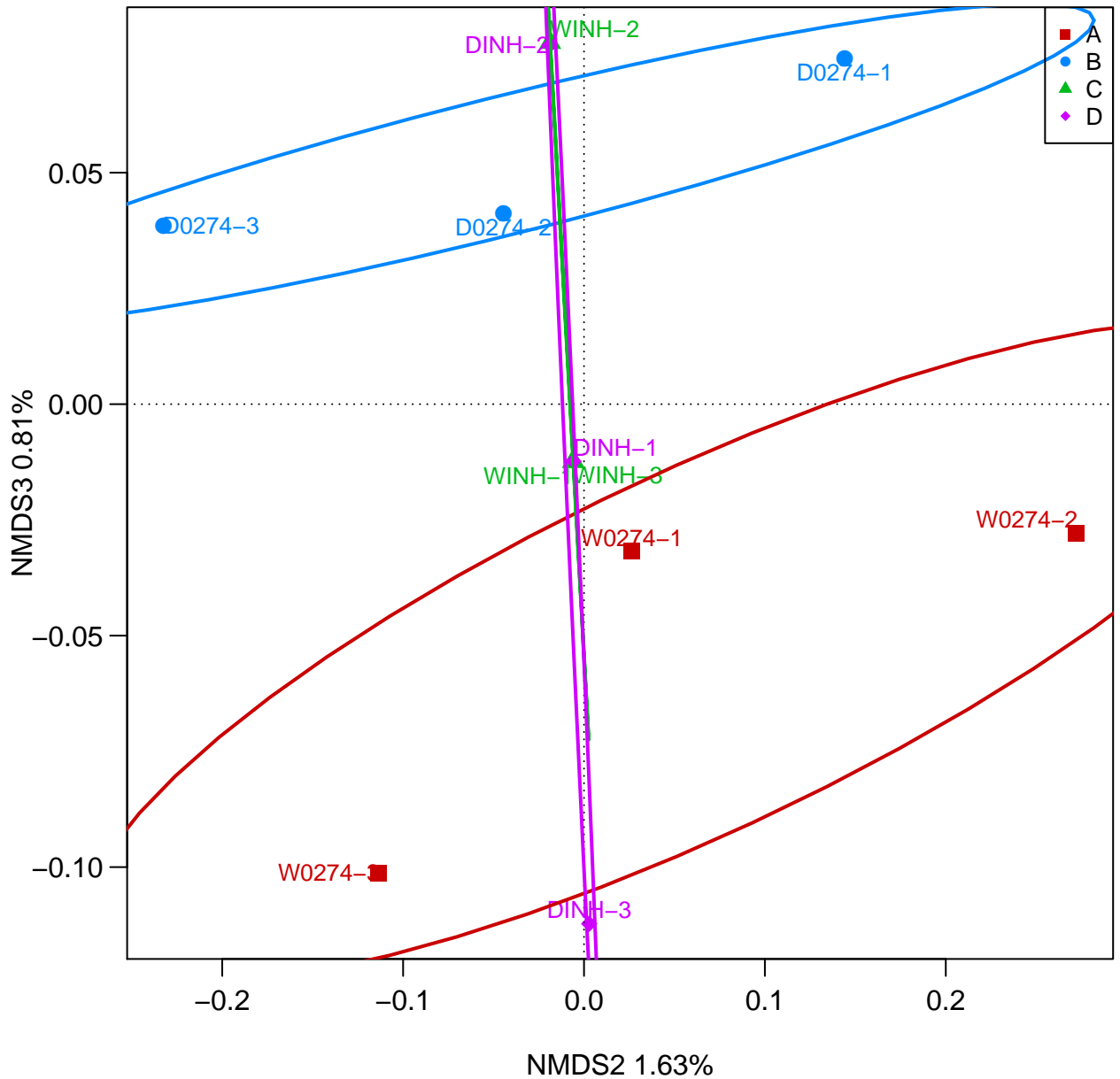

Supplement: Supplementary file 1 [file Supplementary_file_1.zip › RNA_seq_expression/nmds/gene_tpm_nmds.pdf]

# NMDS 3D

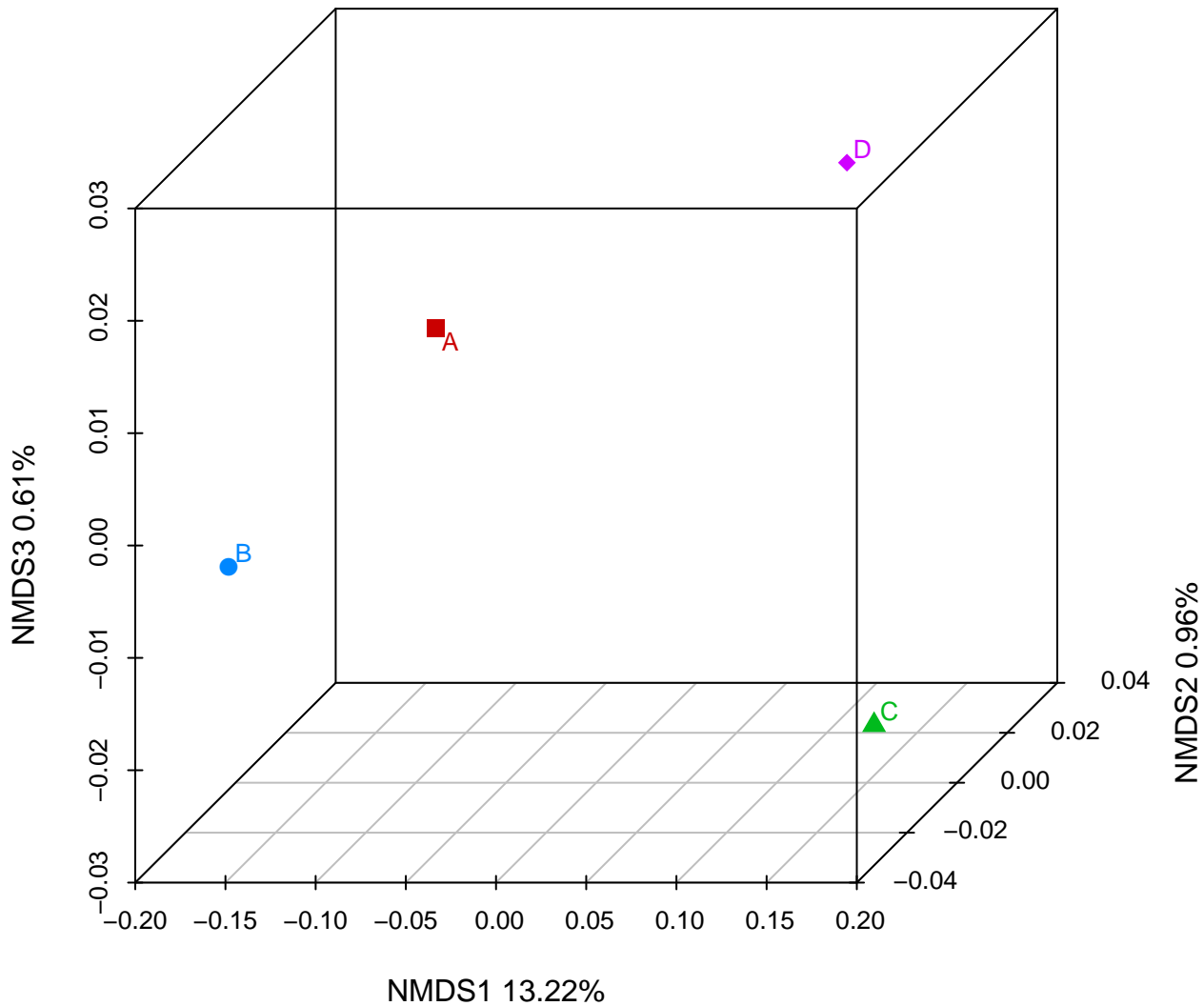

# NMDS – MDS1 vs MDS2

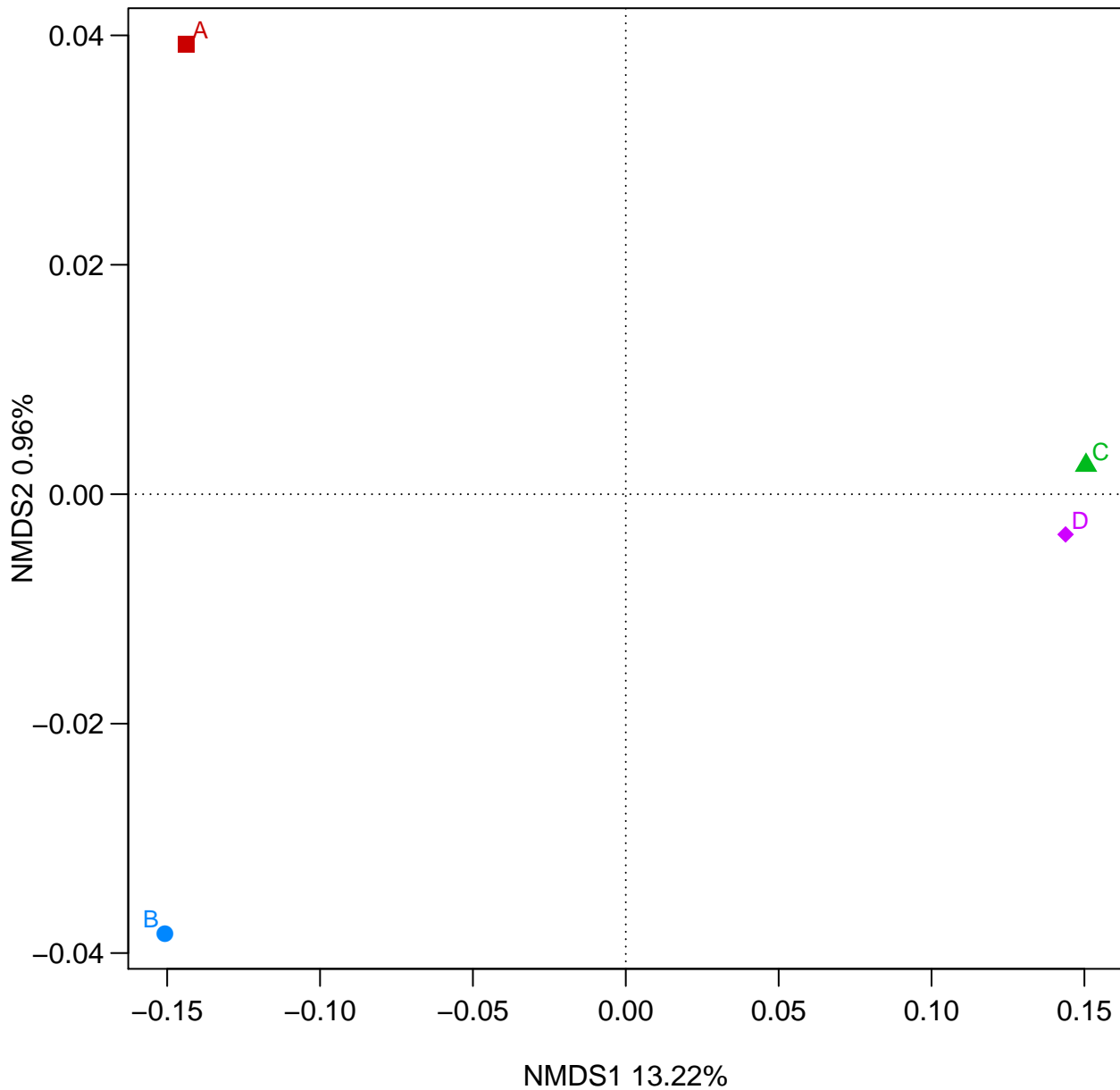

# NMDS – MDS1 vs MDS3

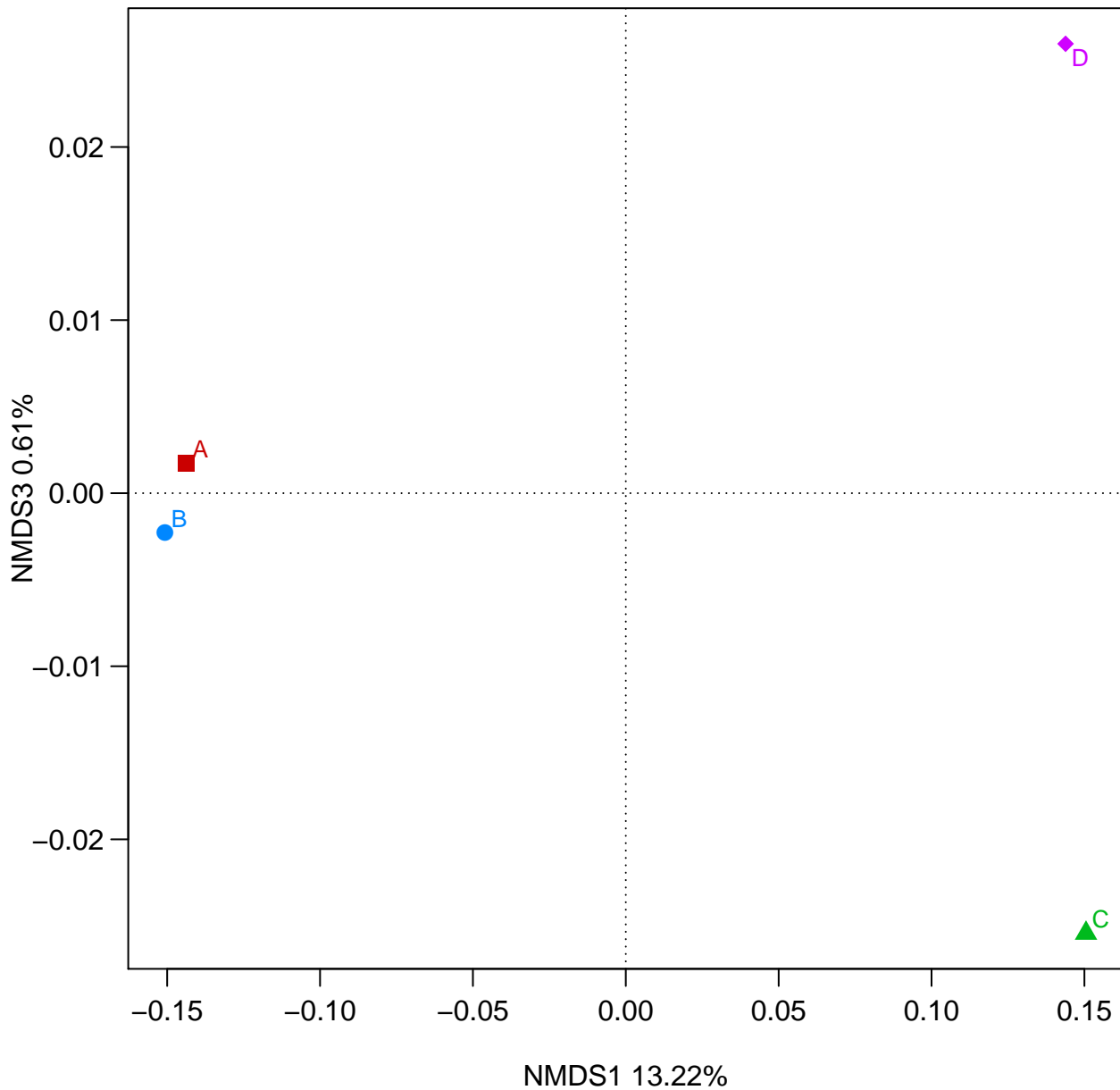

# NMDS – MDS2 vs MDS3

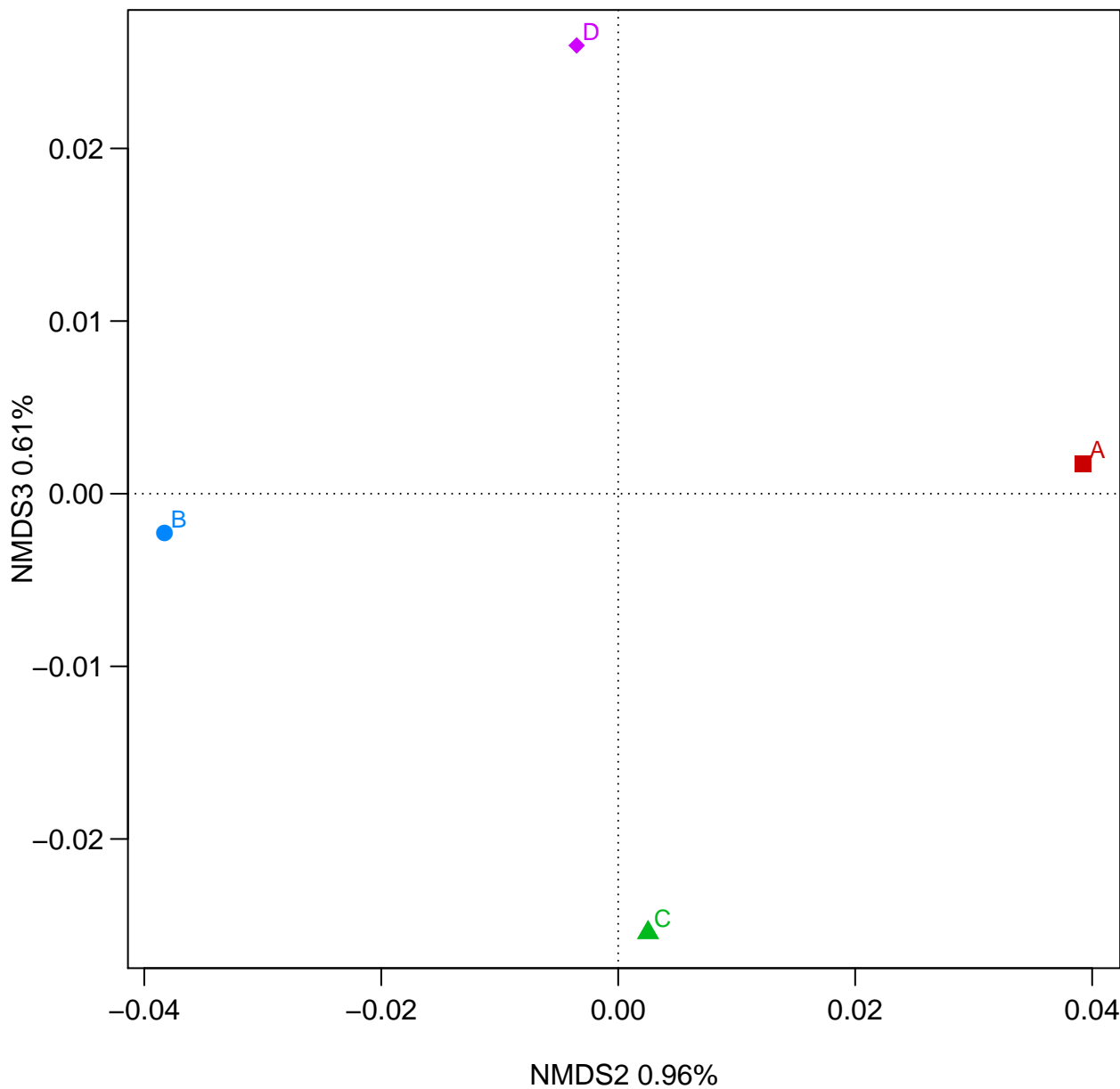

Supplement: Supplementary file 1 [file Supplementary_file_1.zip › RNA_seq_expression/nmds/groupmerge.gene_tpm_nmds.pdf]

# PCA 3D

- A
- B
- ▲ C
- ◆ D

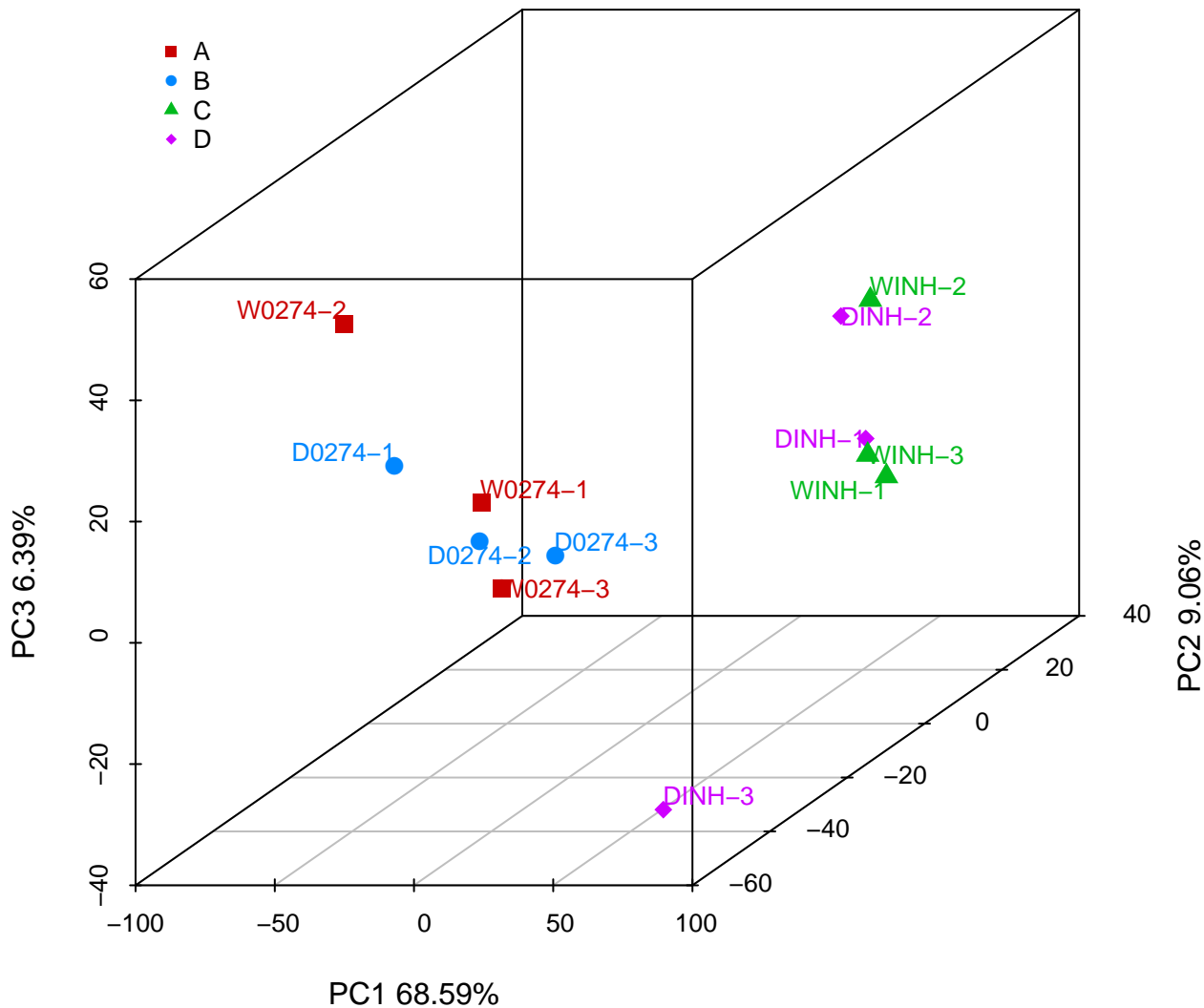

# PCA – PC1 vs PC2

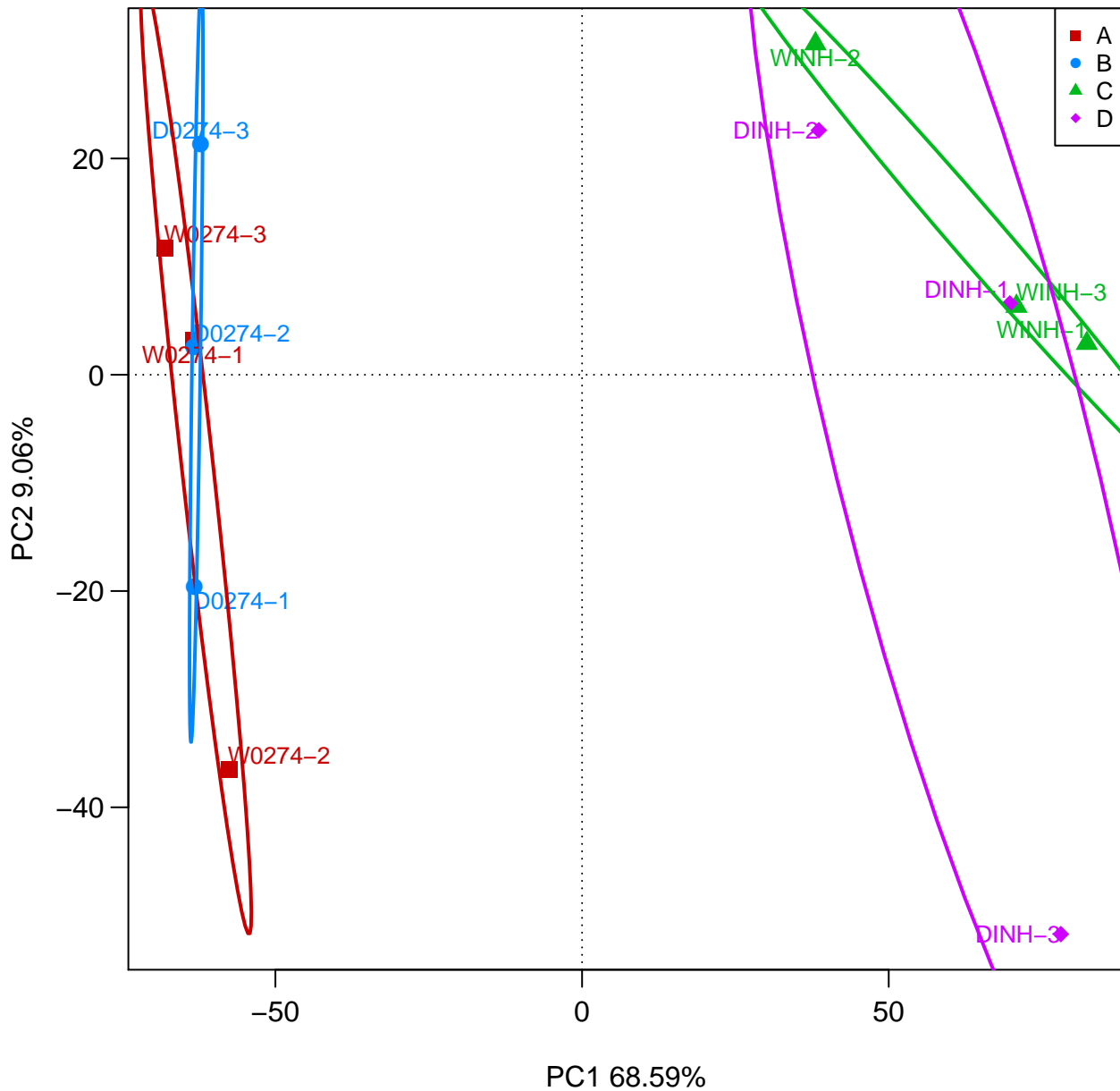

# PCA – PC1 vs PC3

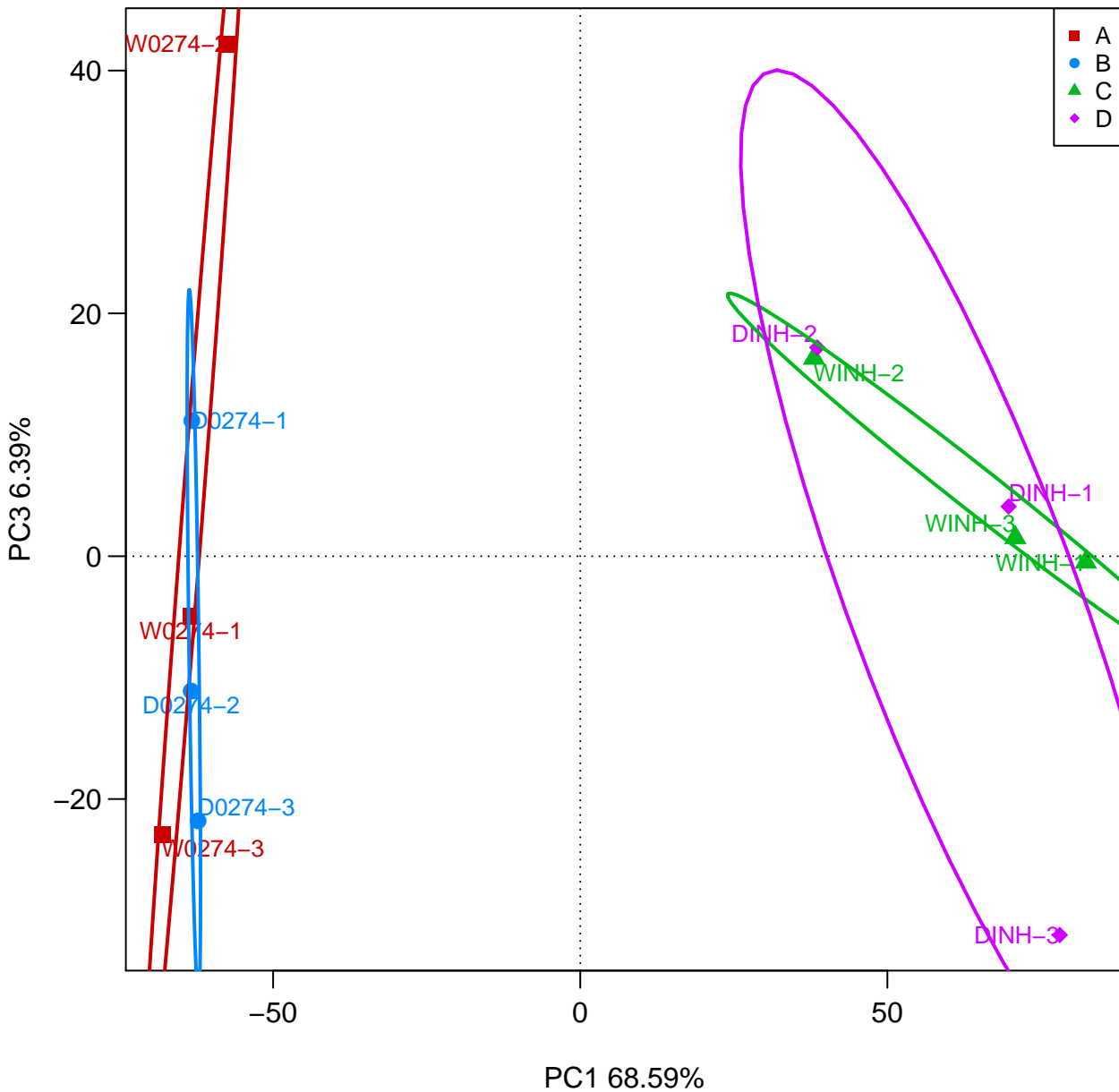

# PCA – PC2 vs PC3

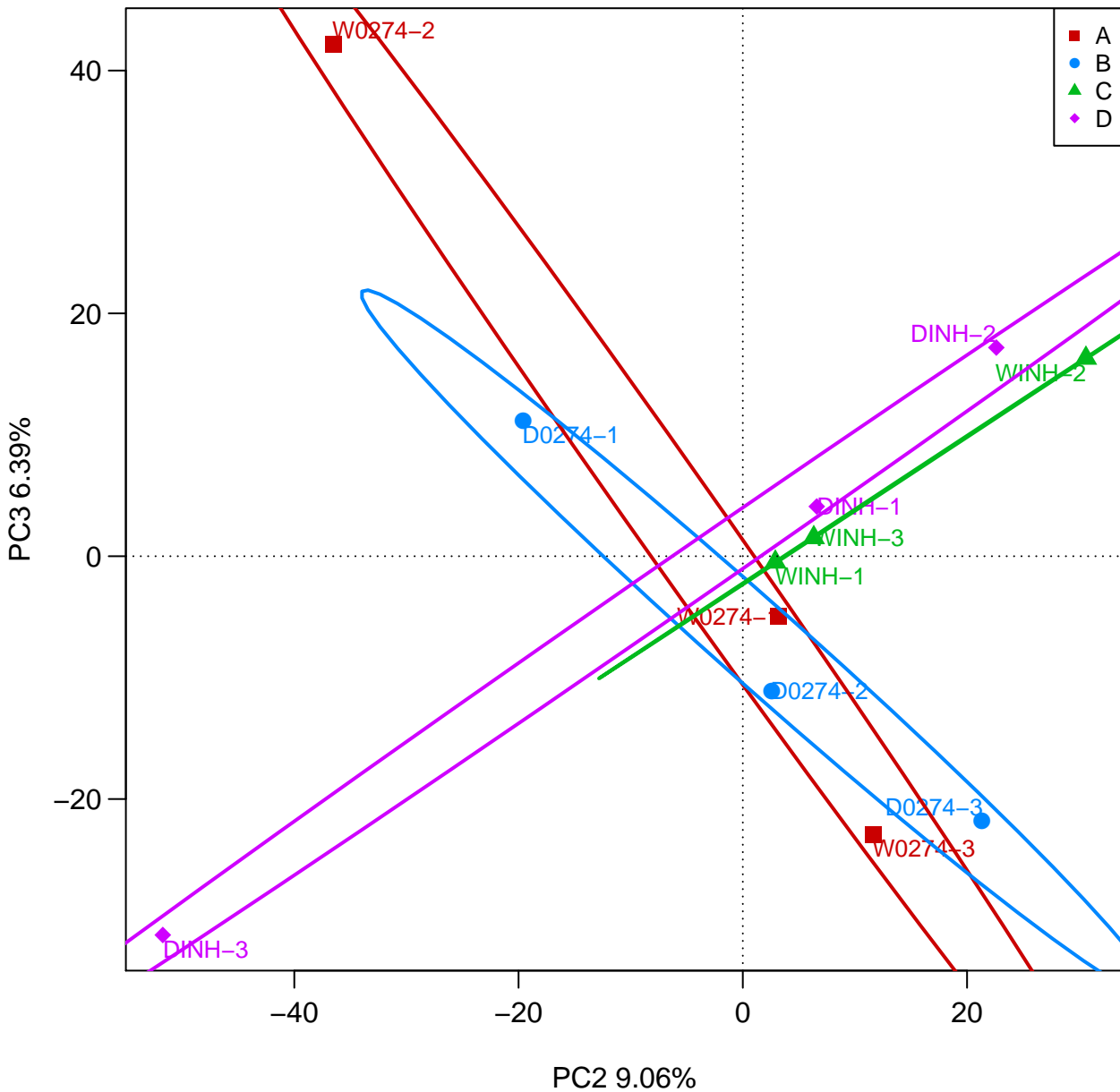

Supplement: Supplementary file 1 [file Supplementary_file_1.zip › RNA_seq_expression/pca/gene_tpm_pca.pdf]

# PCA 3D

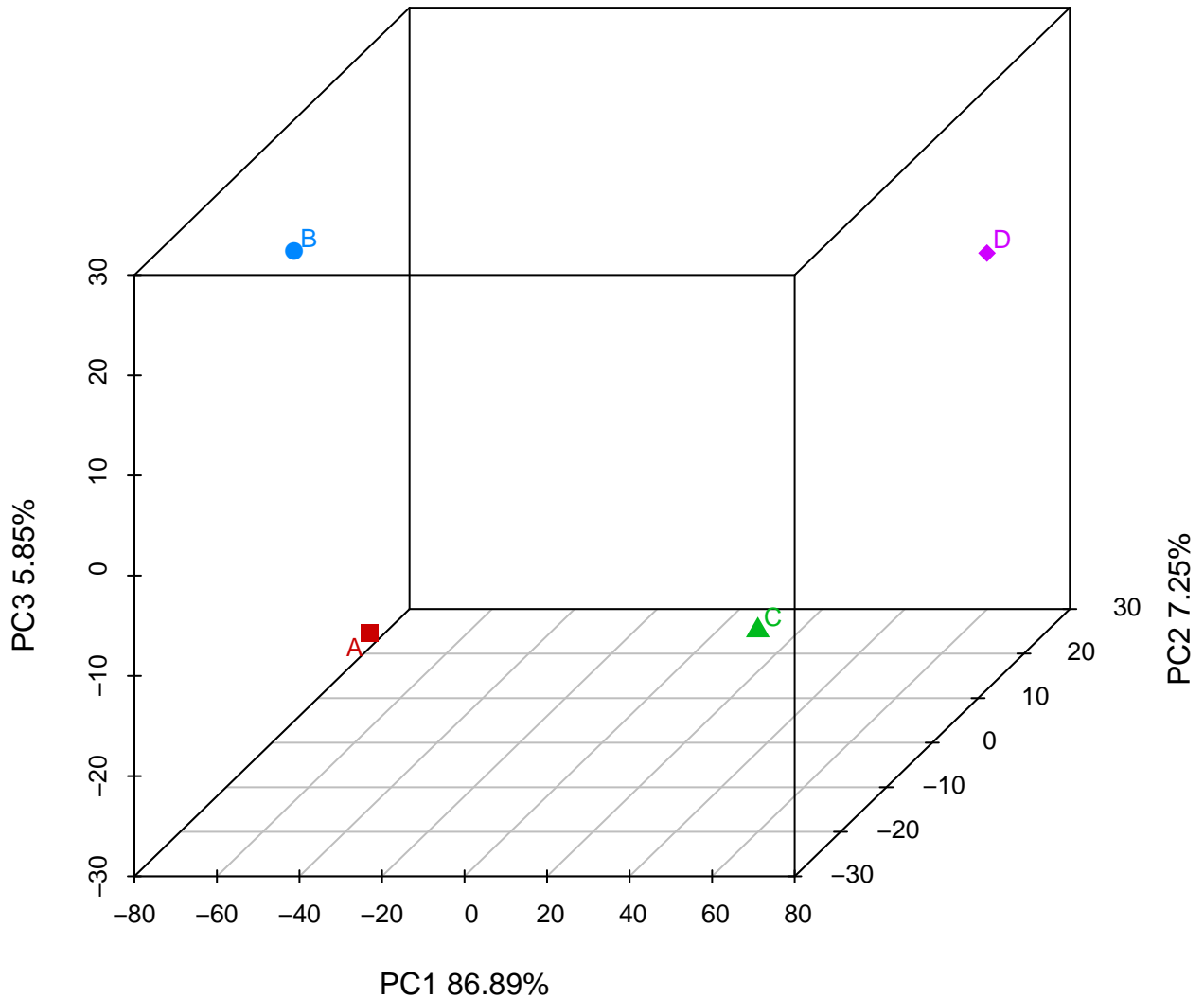

# PCA – PC1 vs PC2

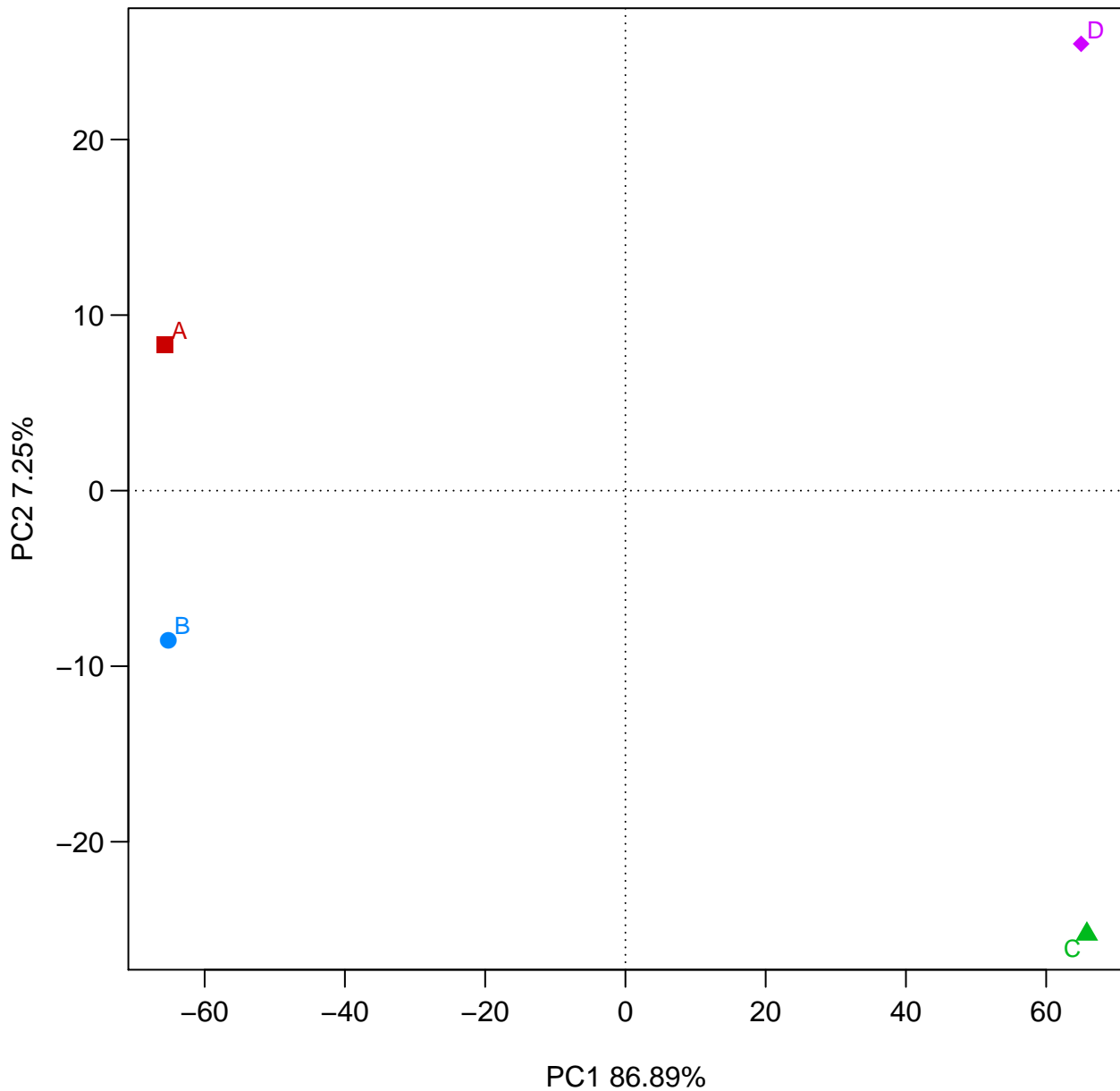

# PCA – PC1 vs PC3

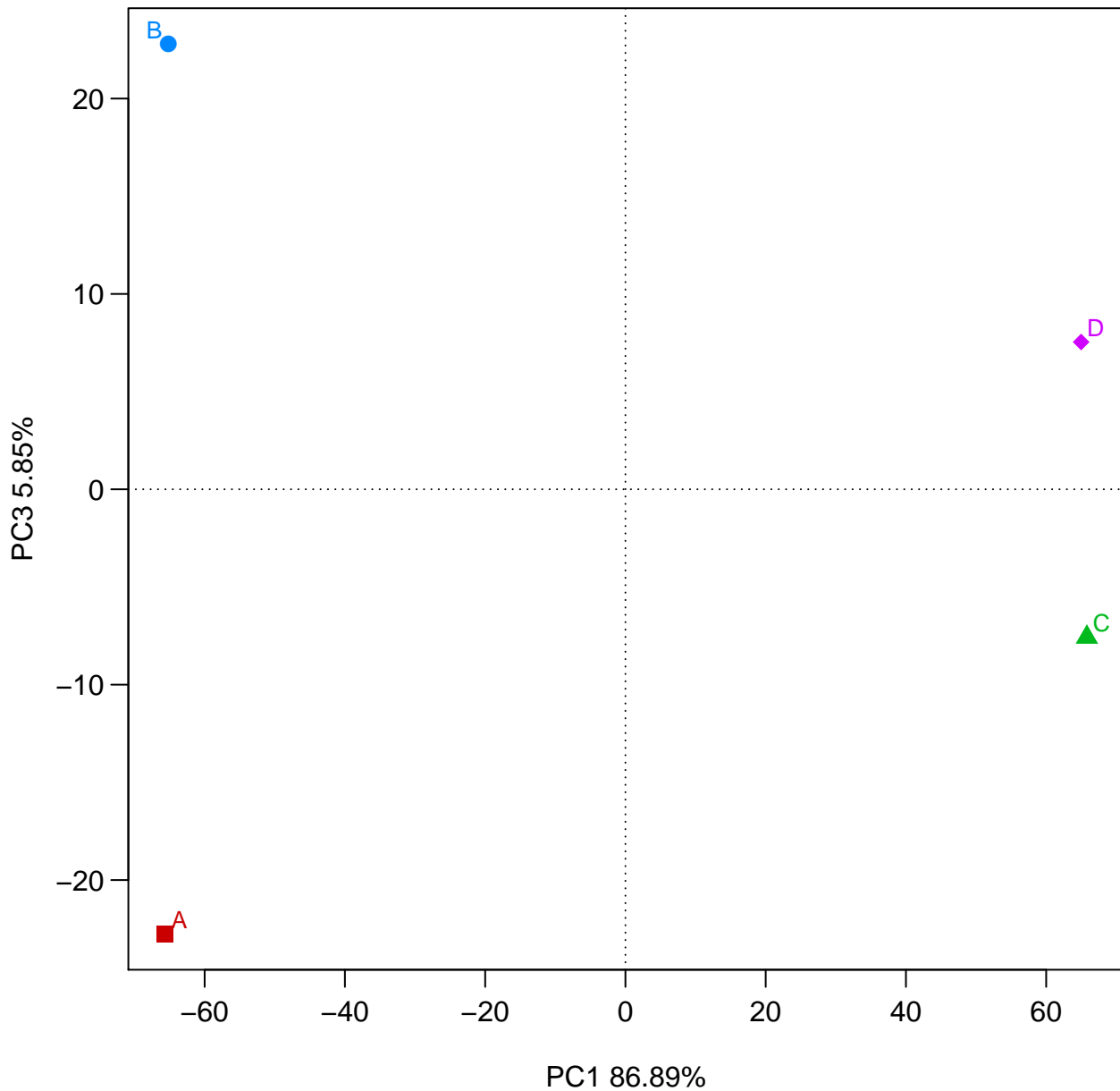

PCA – PC2 vs PC3

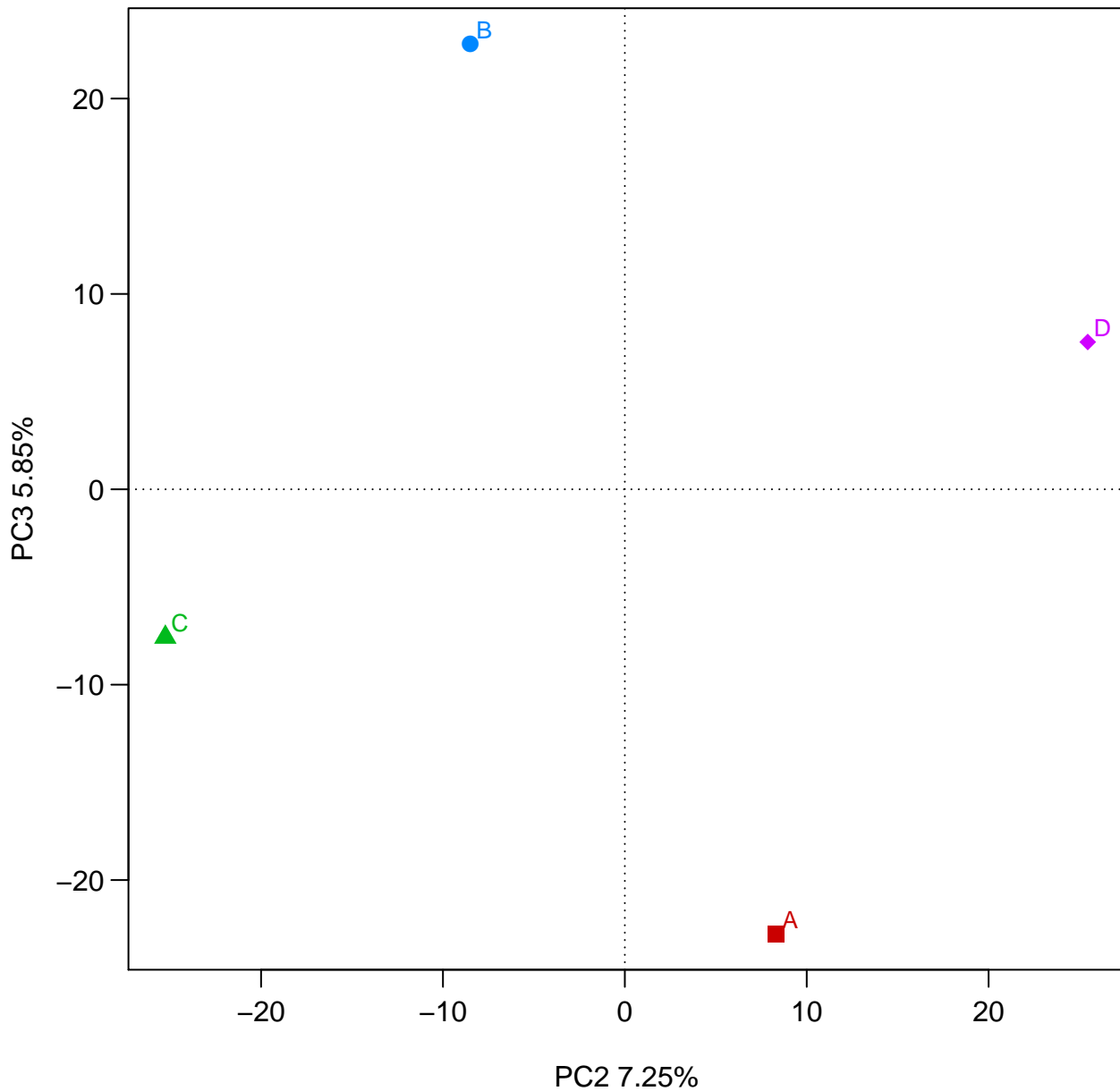

Supplement: Supplementary file 1 [file Supplementary_file_1.zip › RNA_seq_expression/pca/groupmerge.gene_tpm_pca.pdf]

# PCoA 3D

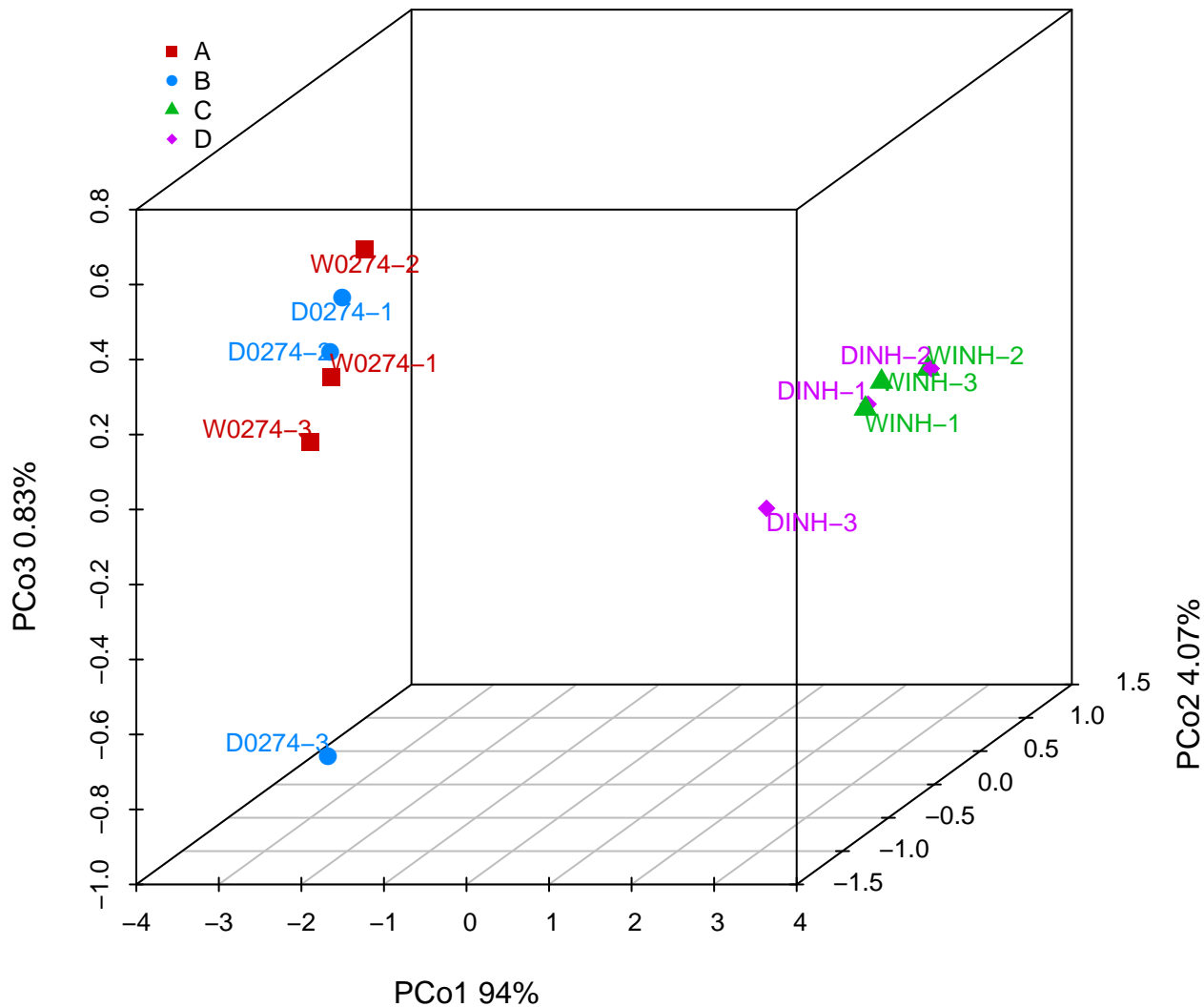

# PCoA – PCo1 vs PCo2

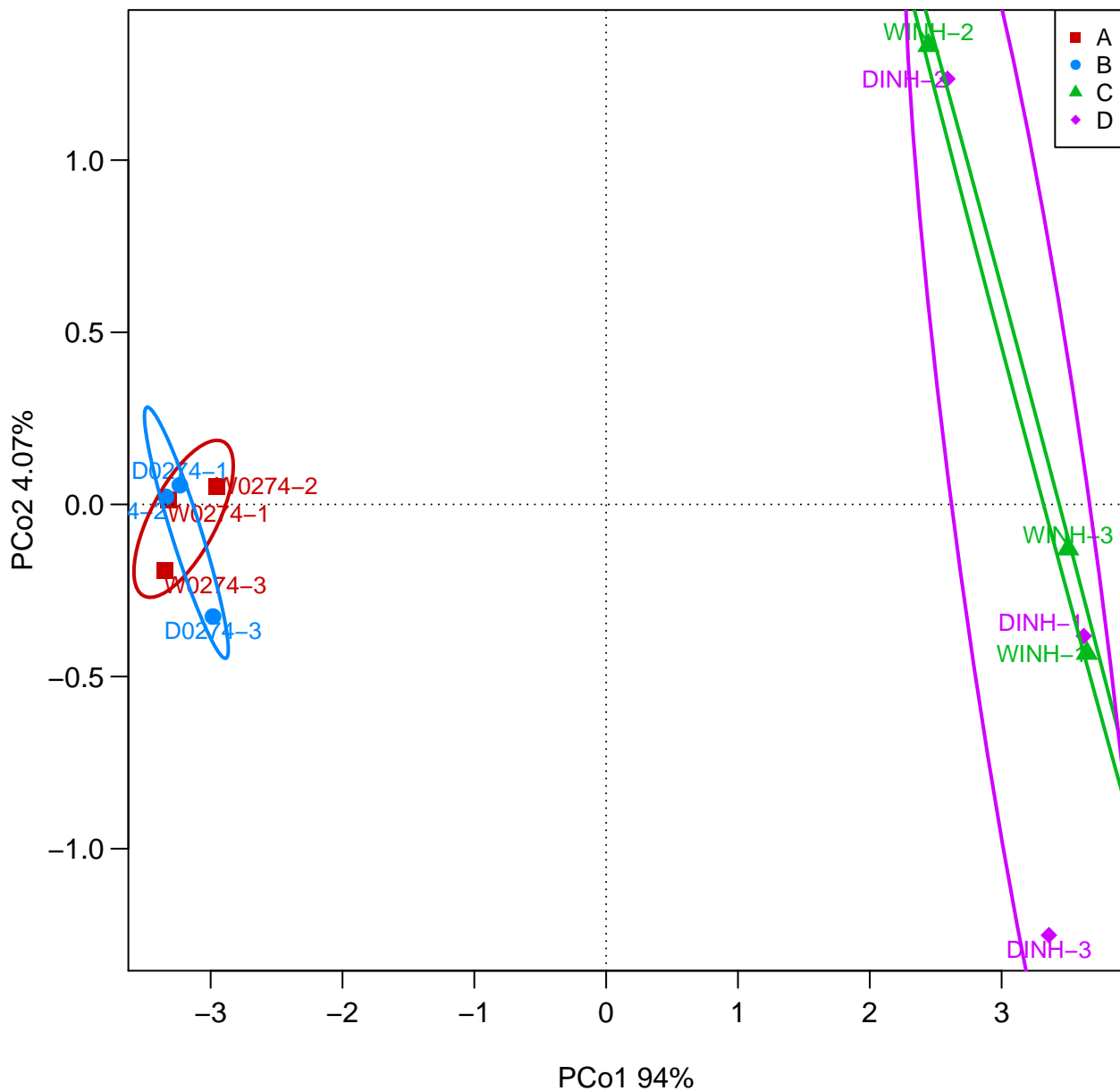

# PCoA – PCo1 vs PCo3

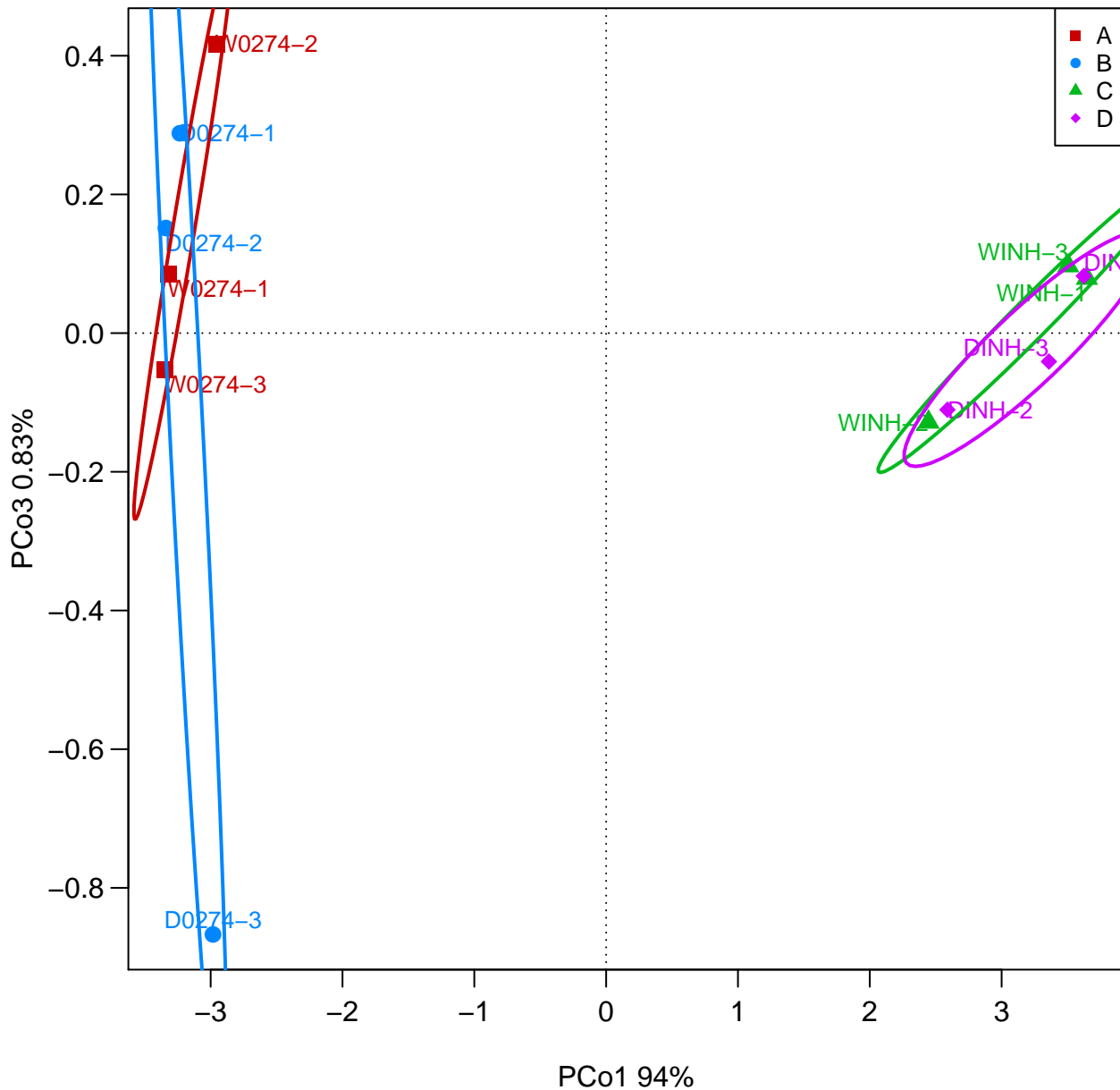

# PCoA – PCo2 vs PCo3

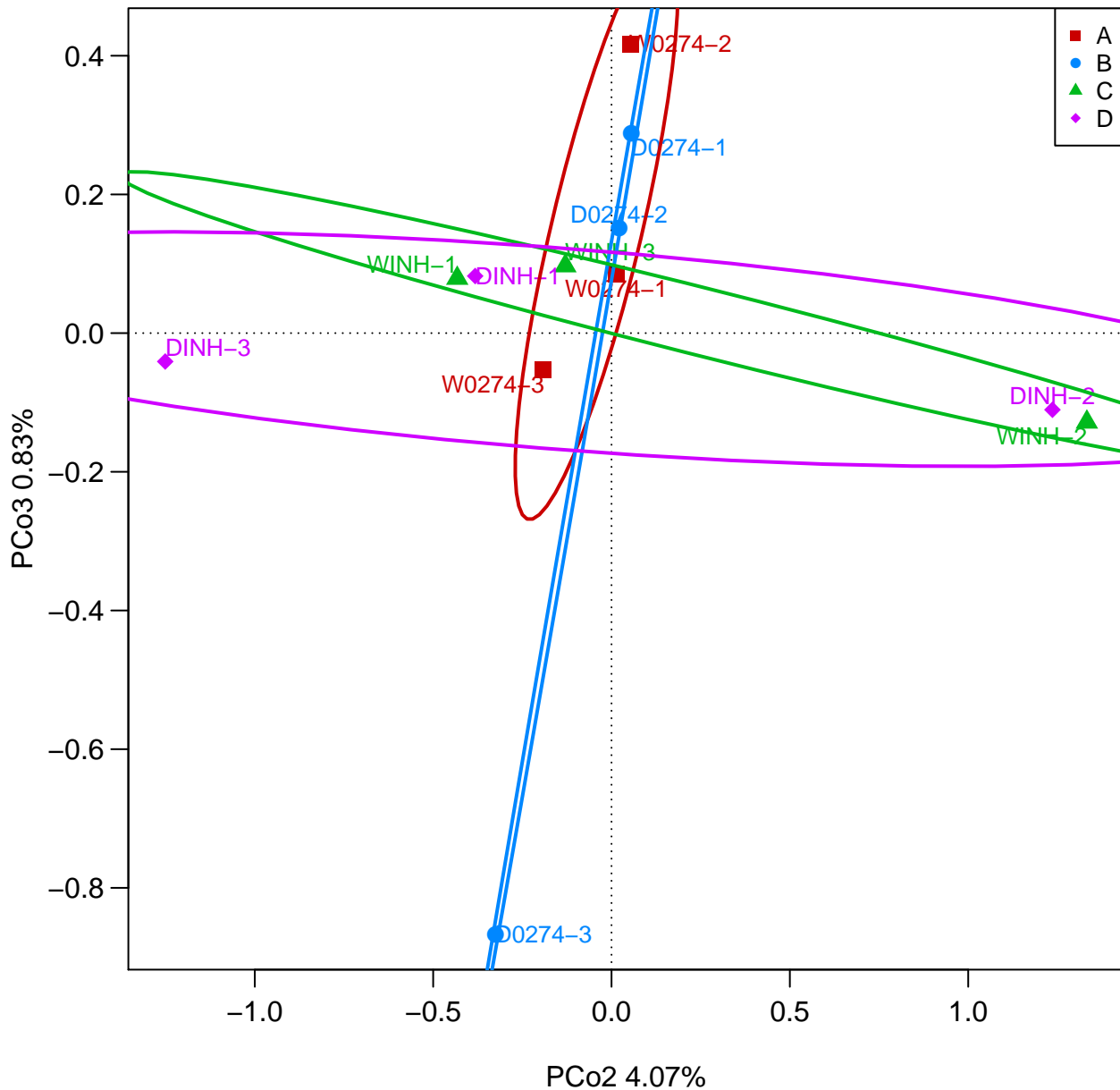

Supplement: Supplementary file 1 [file Supplementary_file_1.zip › RNA_seq_expression/pcoa/gene_tpm_pcoa.pdf]

# PCoA 3D

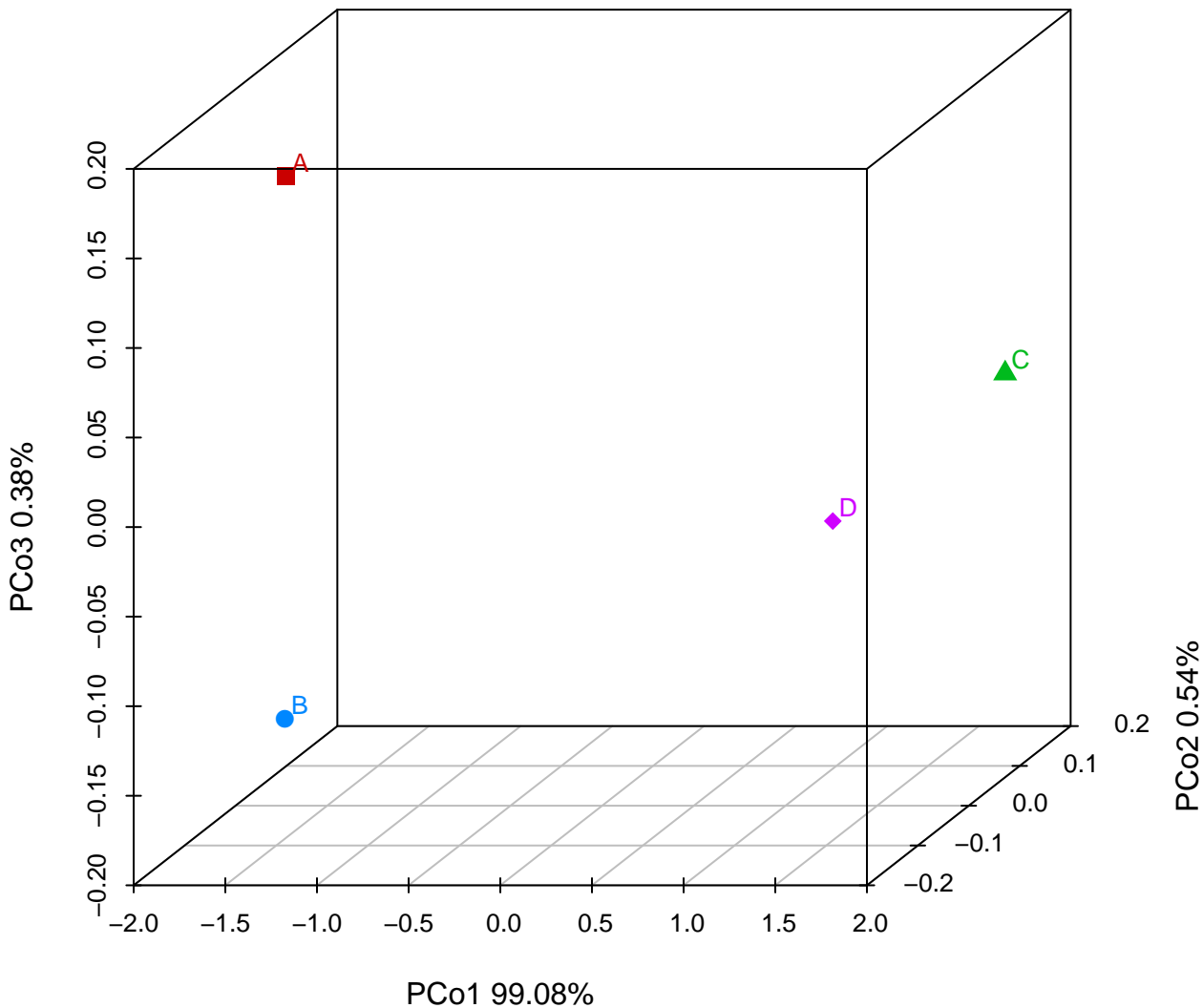

PCoA – PCo1 vs PCo2

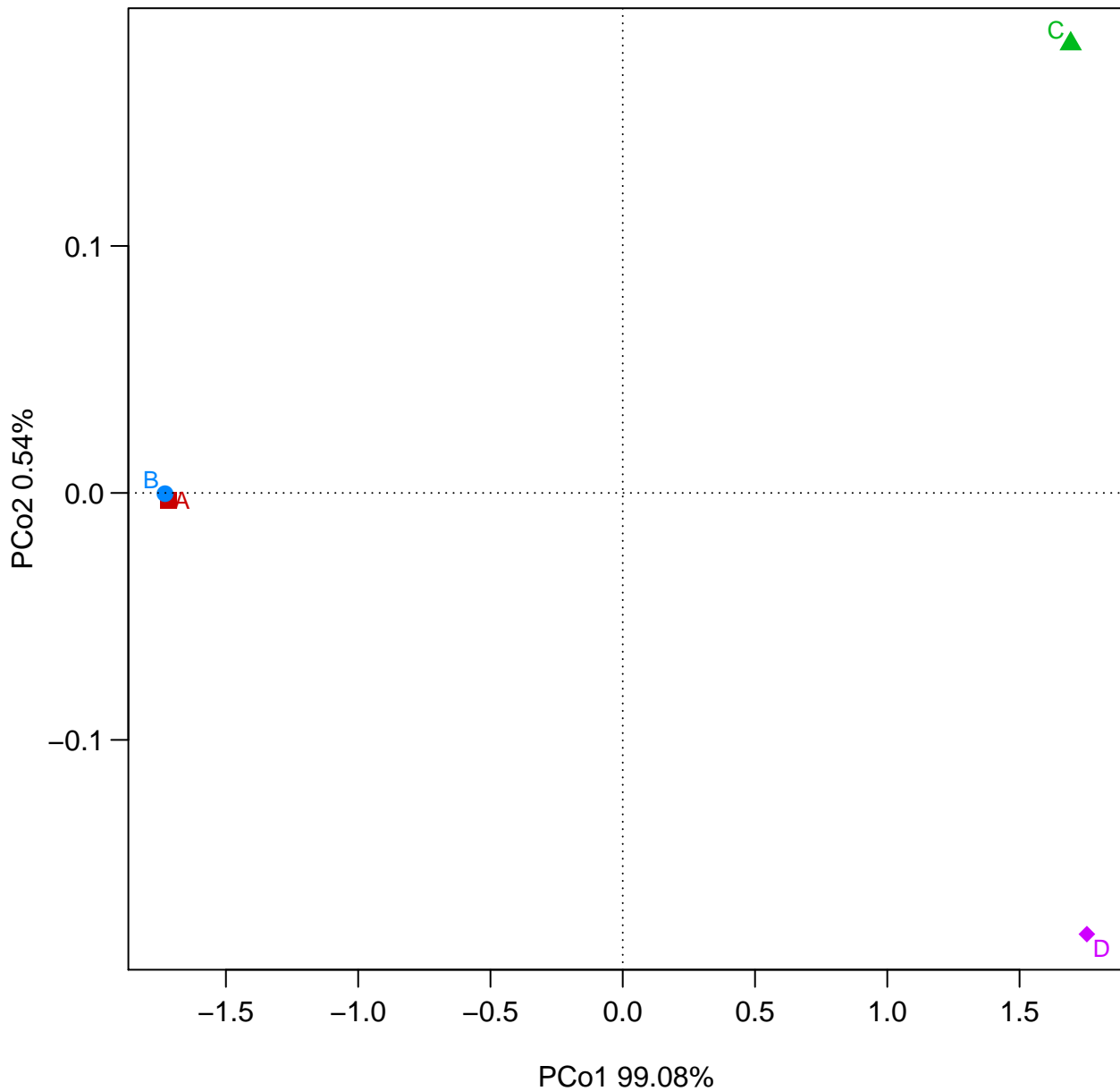

# PCoA – PCo1 vs PCo3

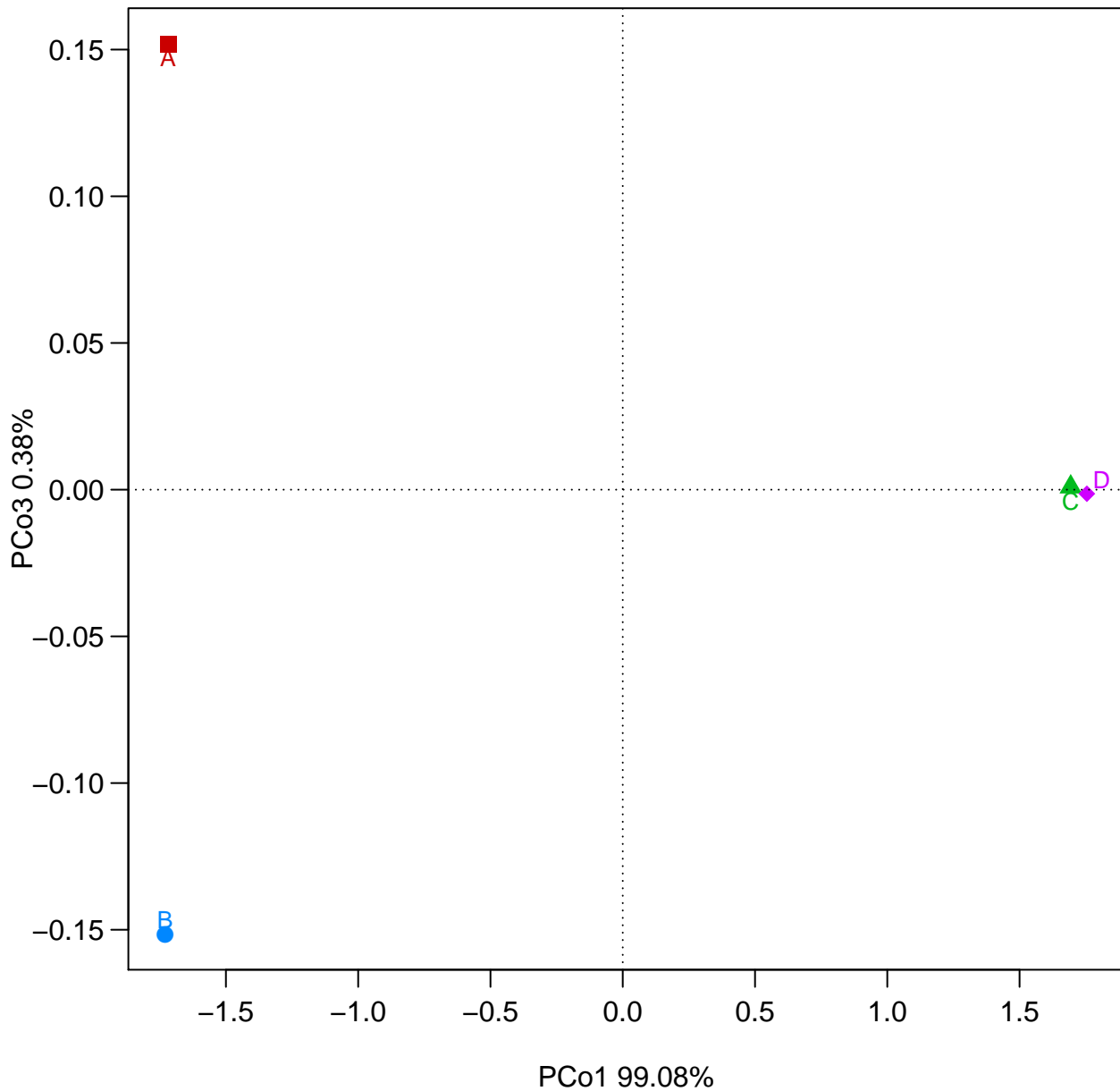

# PCoA – PCo2 vs PCo3

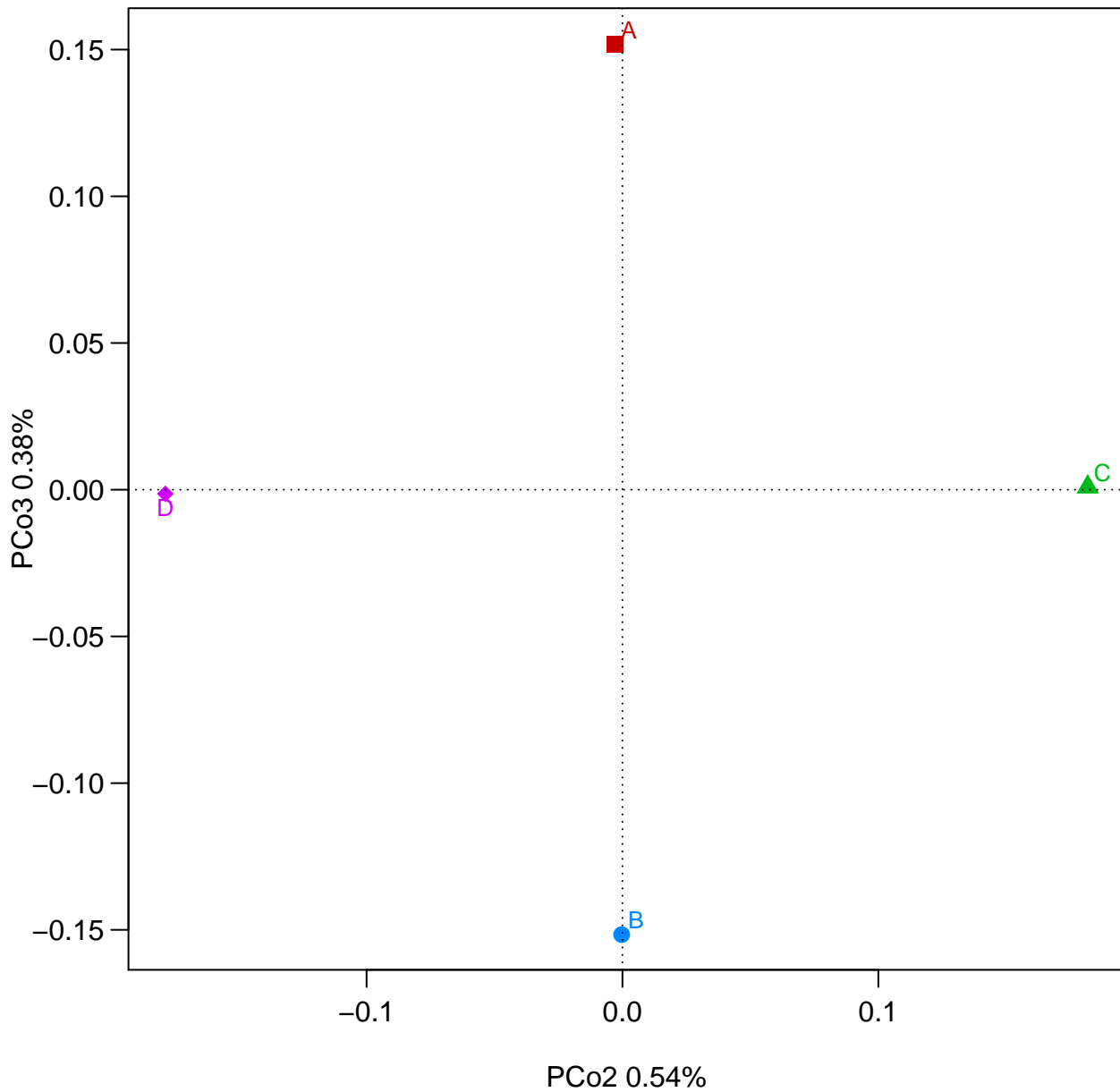

Supplement: Supplementary file 1 [file Supplementary_file_1.zip › RNA_seq_expression/pcoa/groupmerge.gene_tpm_pcoa.pdf]

# D0274-2 VS D0274-1

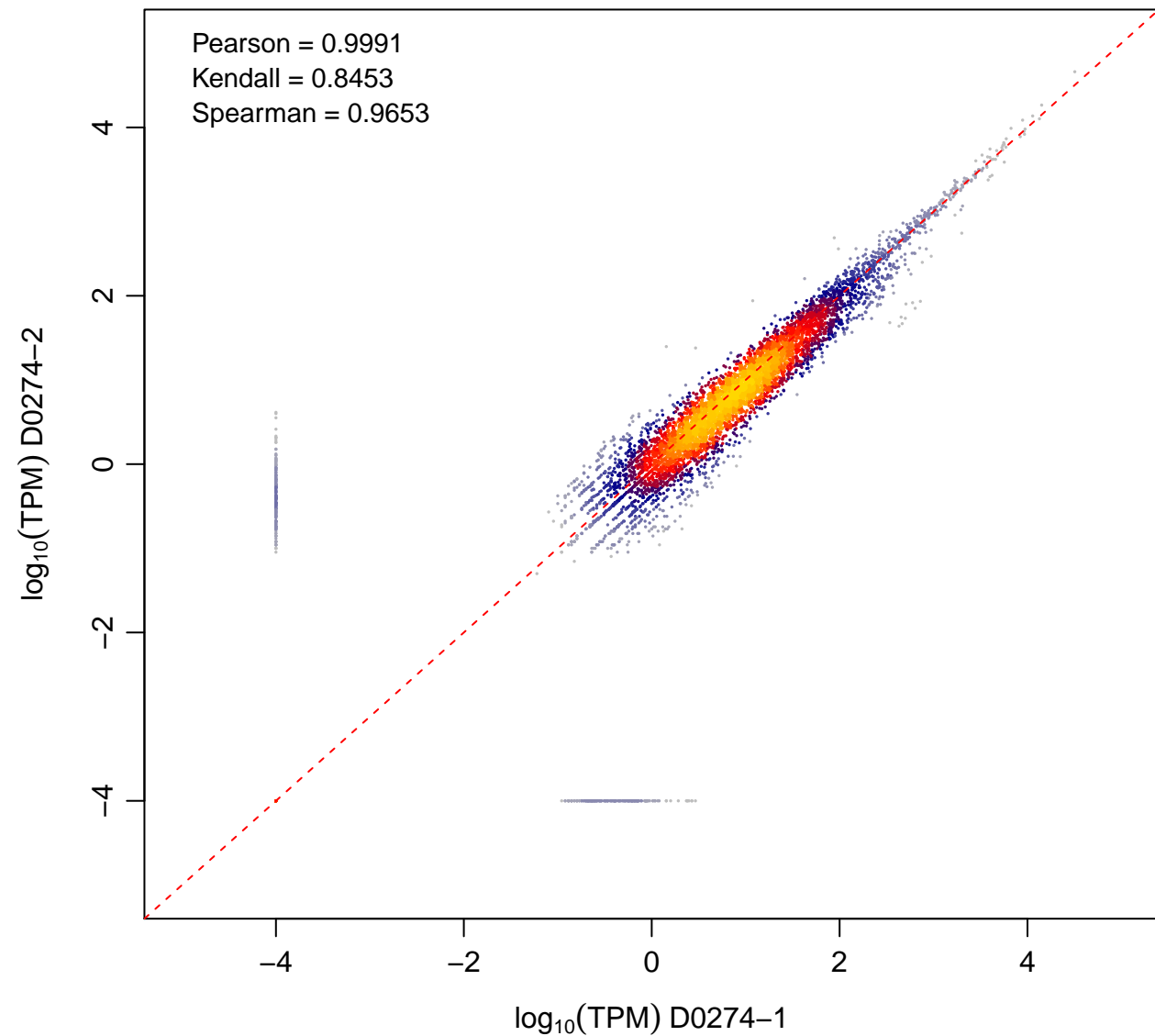

Supplement: Supplementary file 1 [file Supplementary_file_1.zip › RNA_seq_expression/replicate/D0274-2_vs_D0274-1.gene_tpm_replicate_correlation.pdf]

# D0274-3 VS D0274-1

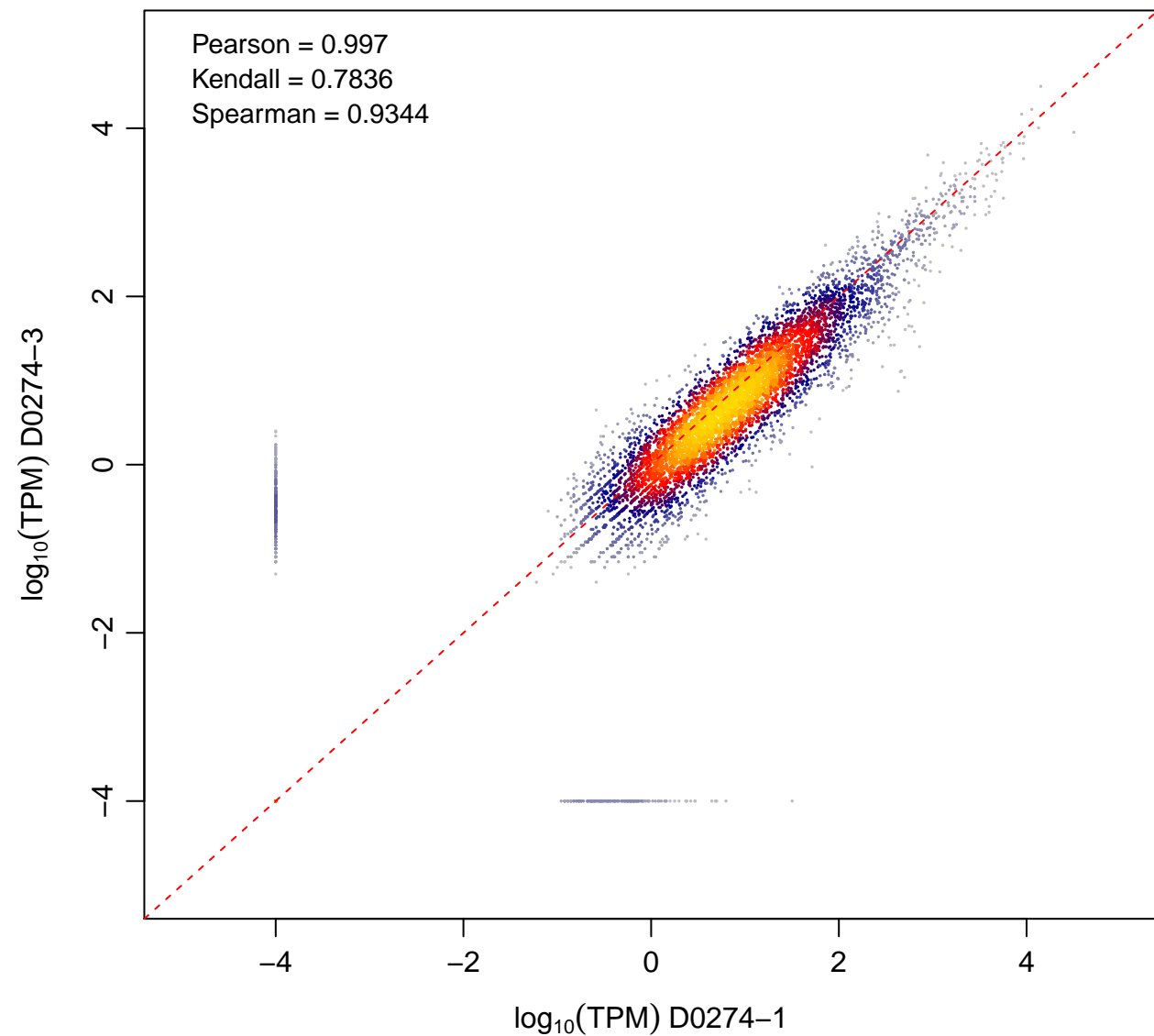

Supplement: Supplementary file 1 [file Supplementary_file_1.zip › RNA_seq_expression/replicate/D0274-3_vs_D0274-1.gene_tpm_replicate_correlation.pdf]

# D0274-3 VS D0274-2

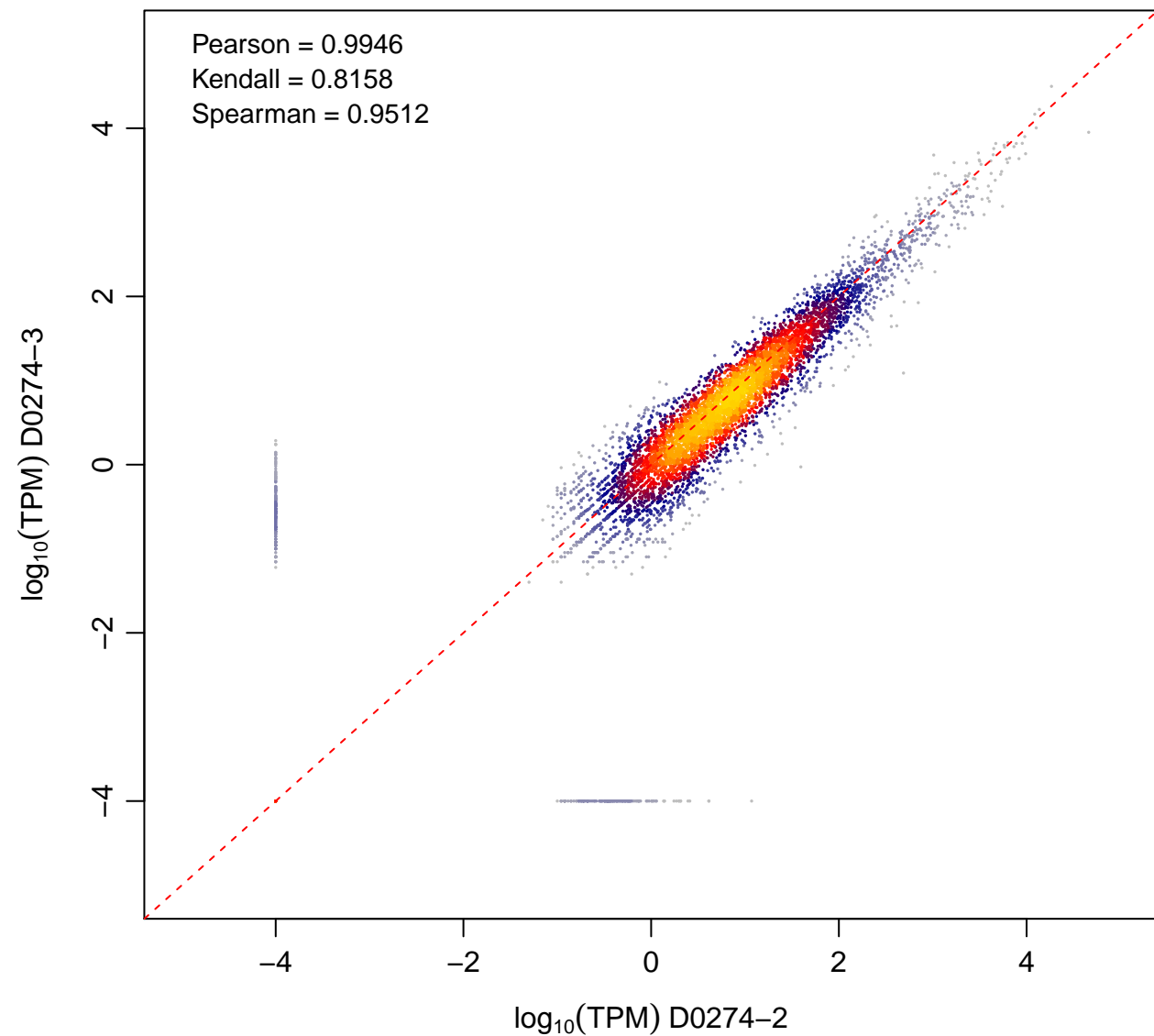

Supplement: Supplementary file 1 [file Supplementary_file_1.zip › RNA_seq_expression/replicate/D0274-3_vs_D0274-2.gene_tpm_replicate_correlation.pdf]

# DINH-2 VS DINH-1

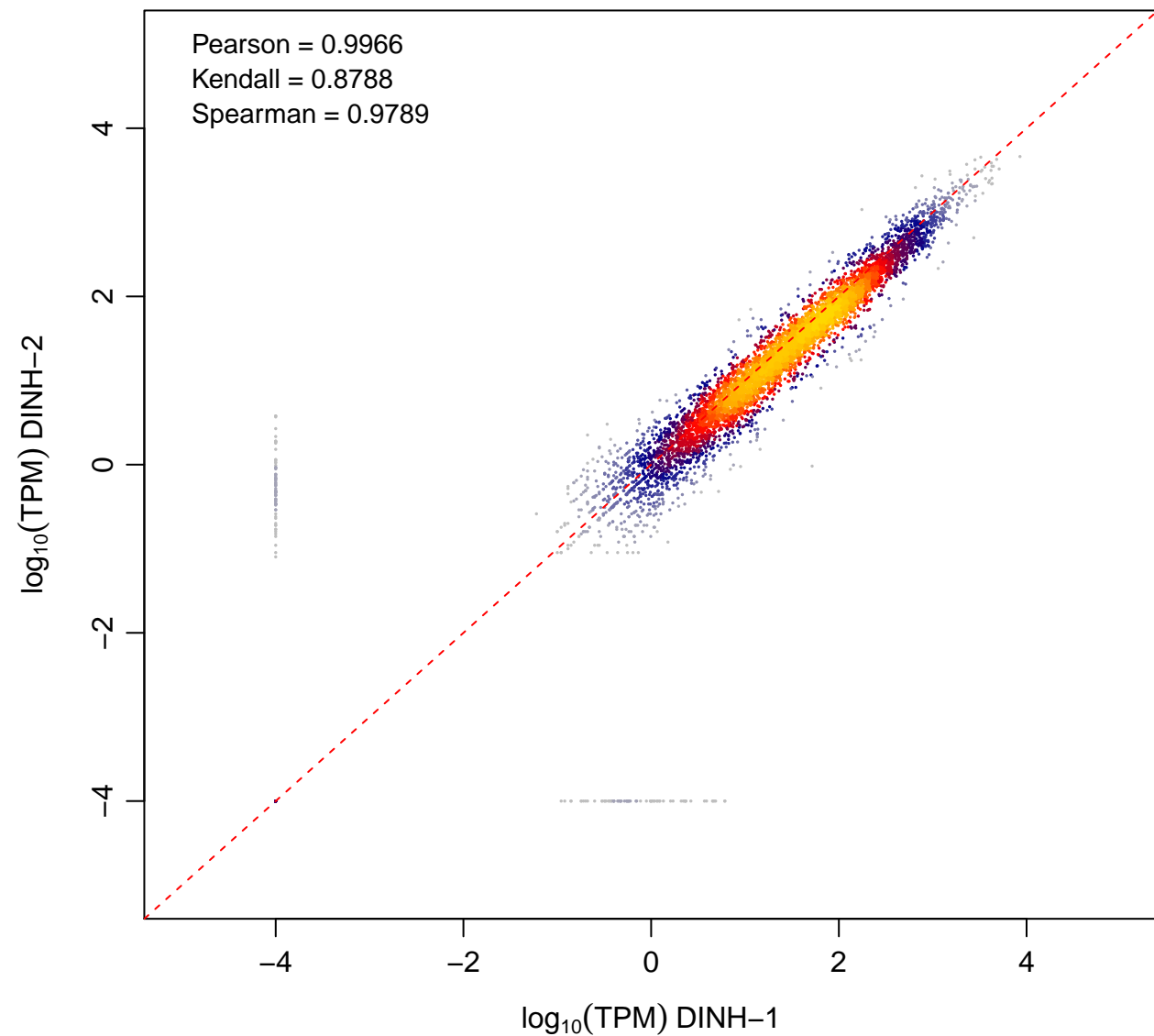

Supplement: Supplementary file 1 [file Supplementary_file_1.zip › RNA_seq_expression/replicate/DINH-2_vs_DINH-1.gene_tpm_replicate_correlation.pdf]

# DINH-3 VS DINH-1

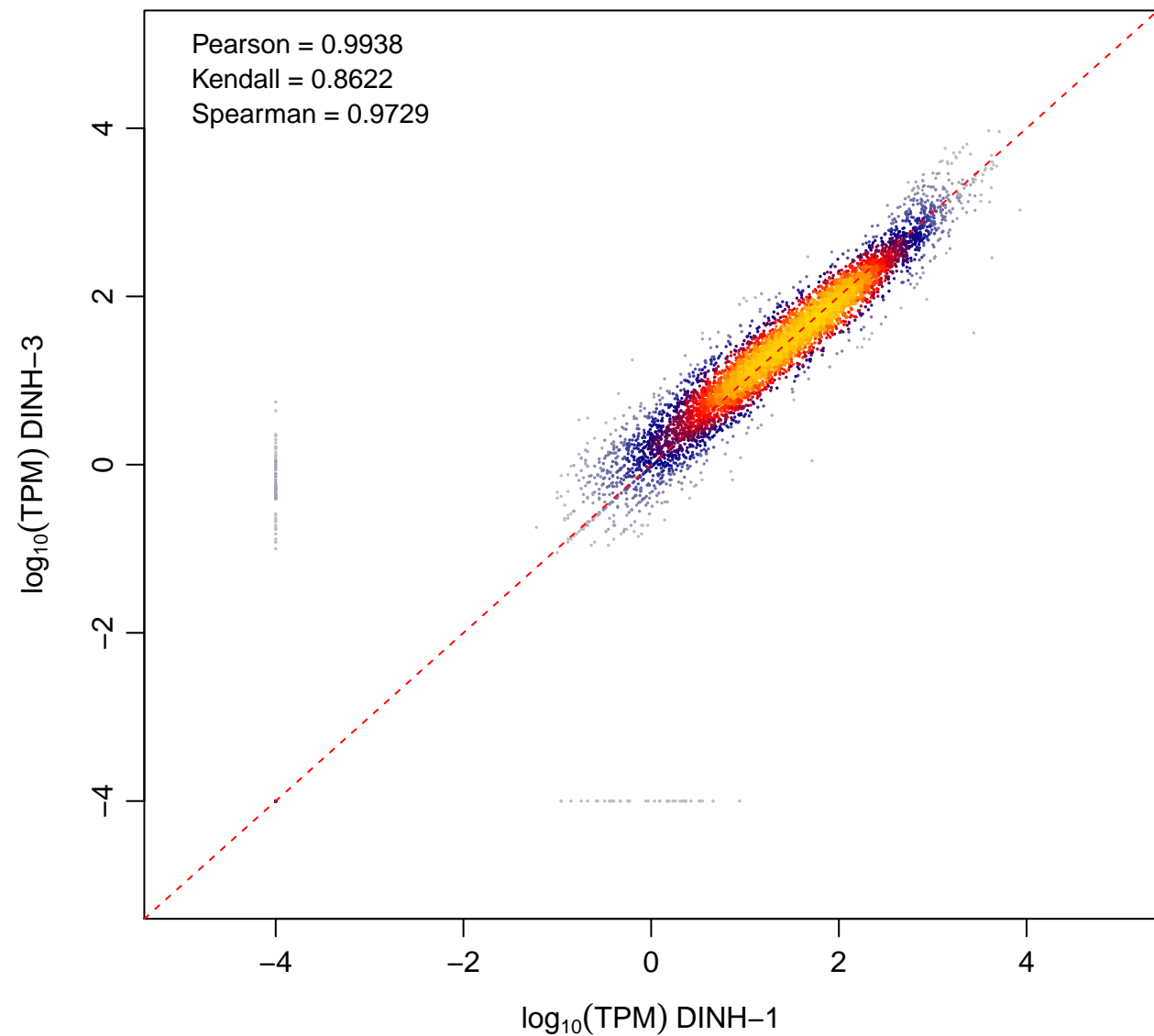

Supplement: Supplementary file 1 [file Supplementary_file_1.zip › RNA_seq_expression/replicate/DINH-3_vs_DINH-1.gene_tpm_replicate_correlation.pdf]

# DINH-3 VS DINH-2

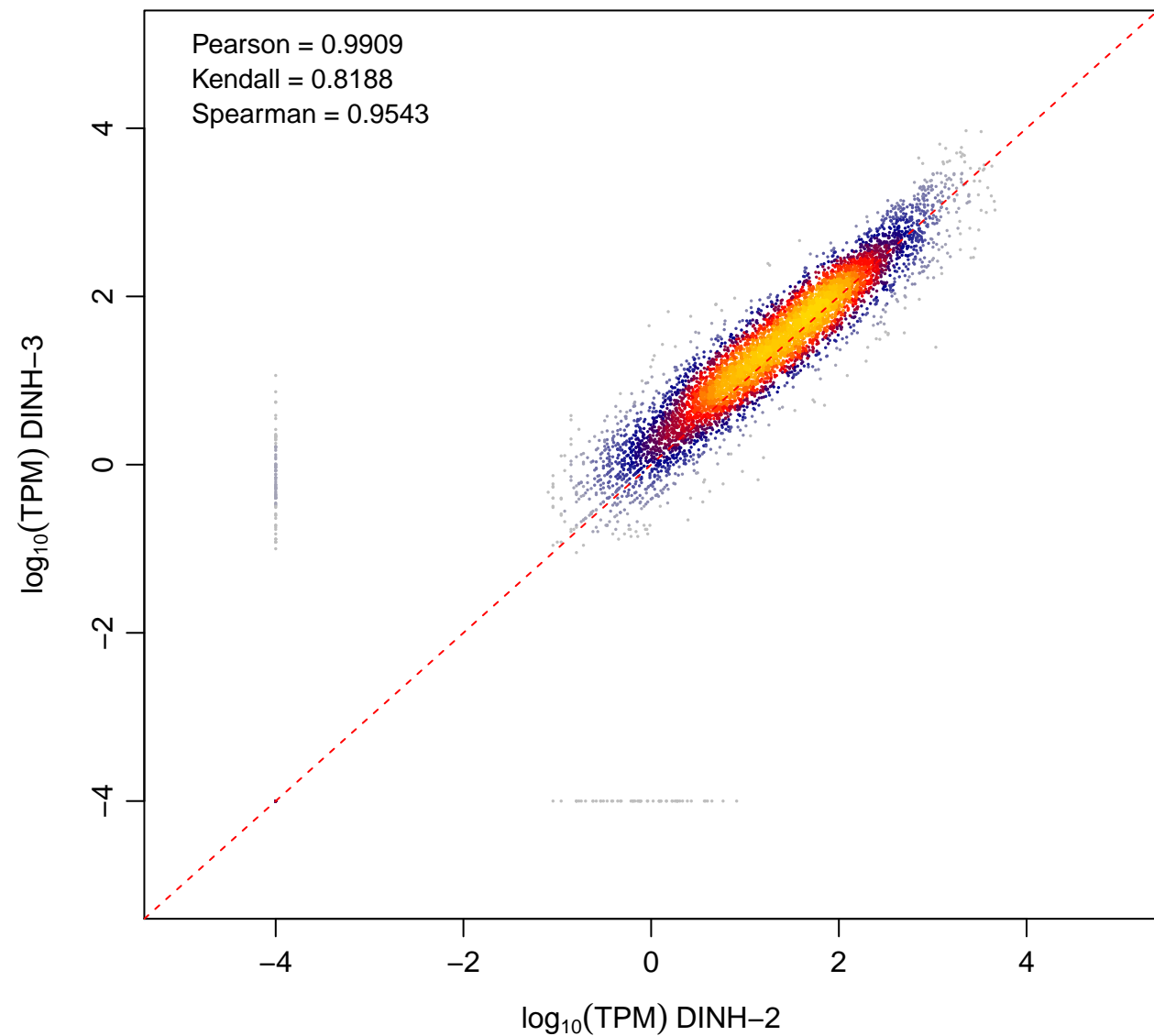

Supplement: Supplementary file 1 [file Supplementary_file_1.zip › RNA_seq_expression/replicate/DINH-3_vs_DINH-2.gene_tpm_replicate_correlation.pdf]

# W0274-2 VS W0274-1

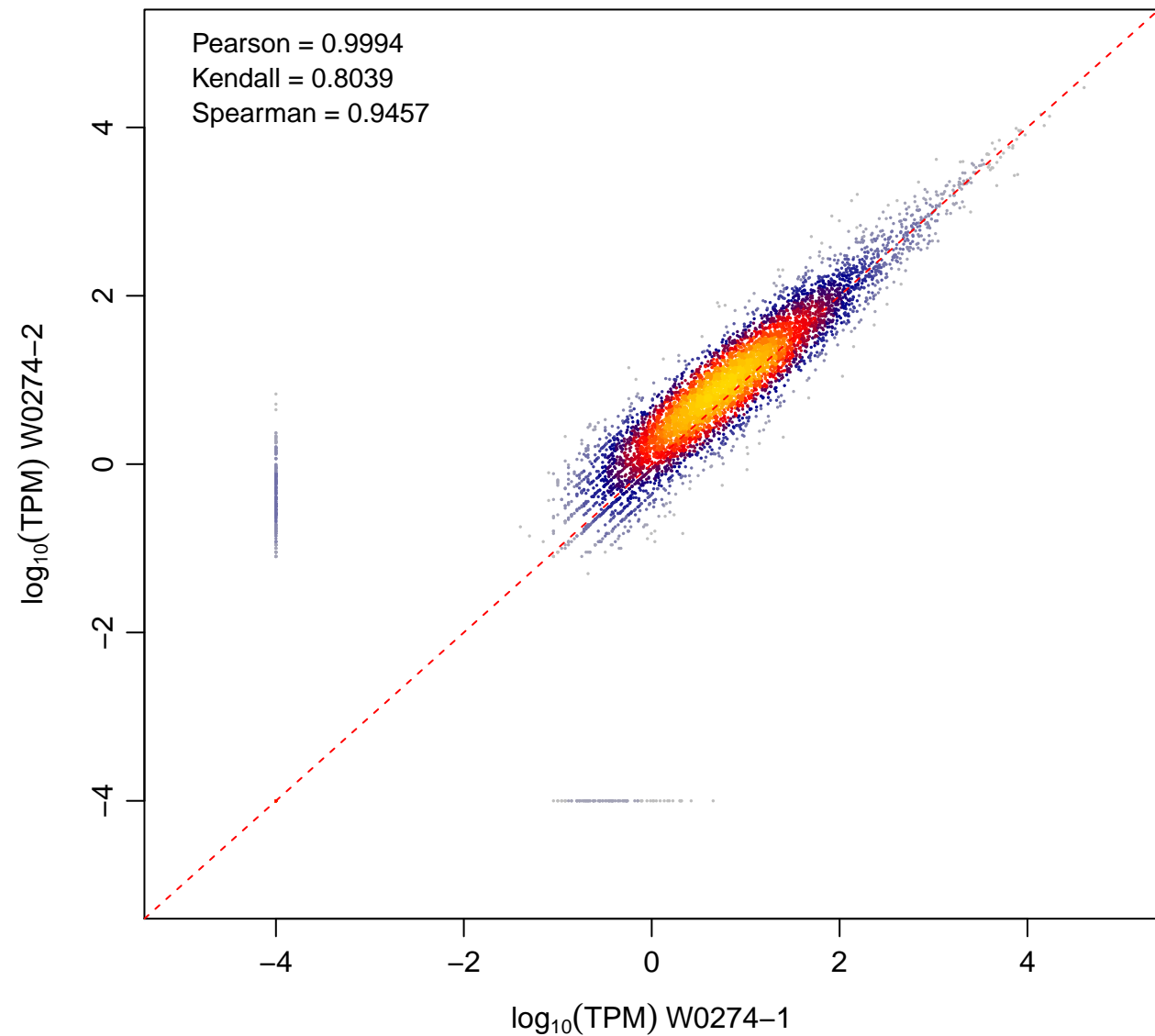

Supplement: Supplementary file 1 [file Supplementary_file_1.zip › RNA_seq_expression/replicate/W0274-2_vs_W0274-1.gene_tpm_replicate_correlation.pdf]

# W0274-3 VS W0274-1

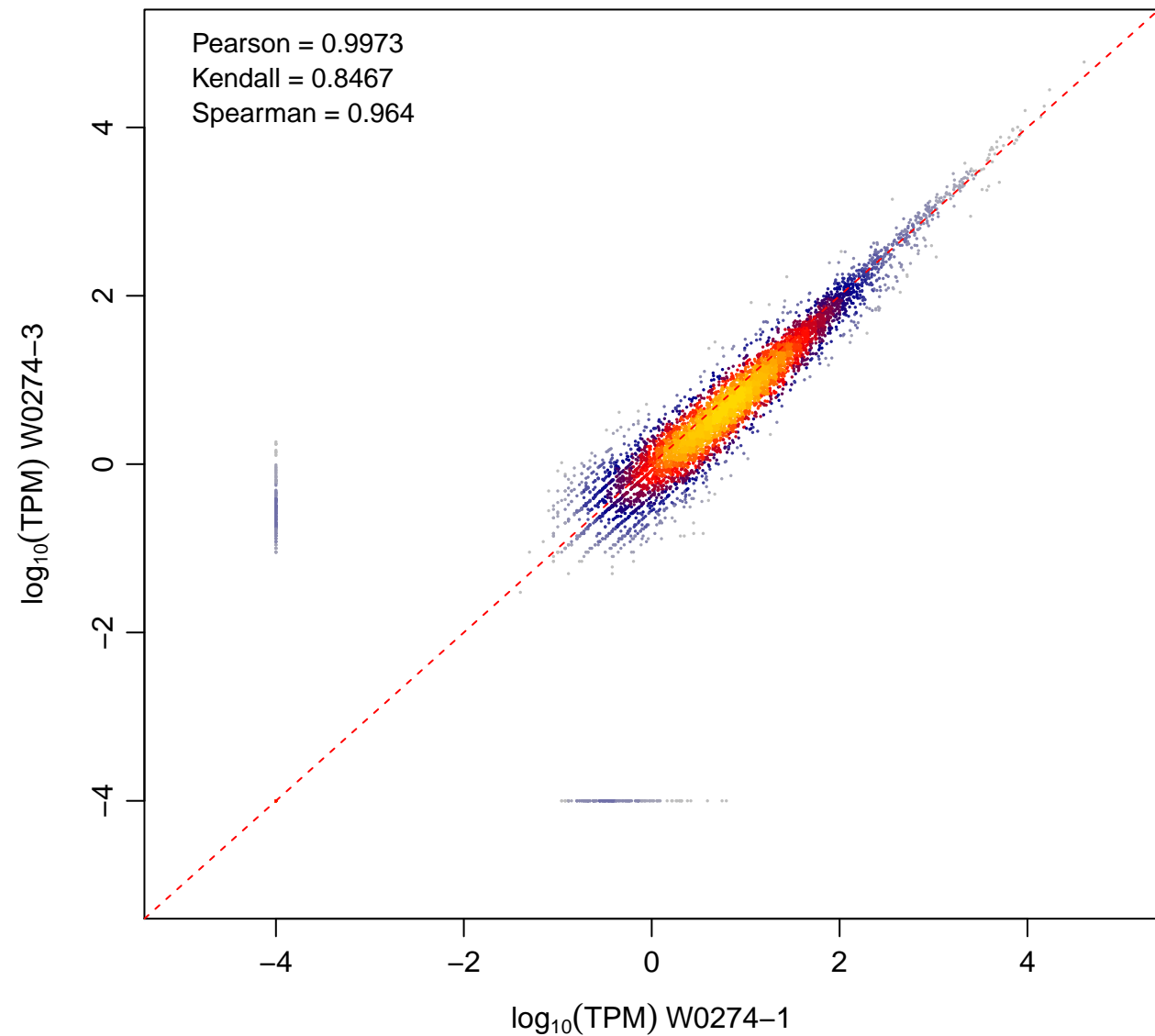

Supplement: Supplementary file 1 [file Supplementary_file_1.zip › RNA_seq_expression/replicate/W0274-3_vs_W0274-1.gene_tpm_replicate_correlation.pdf]

# W0274-3 VS W0274-2

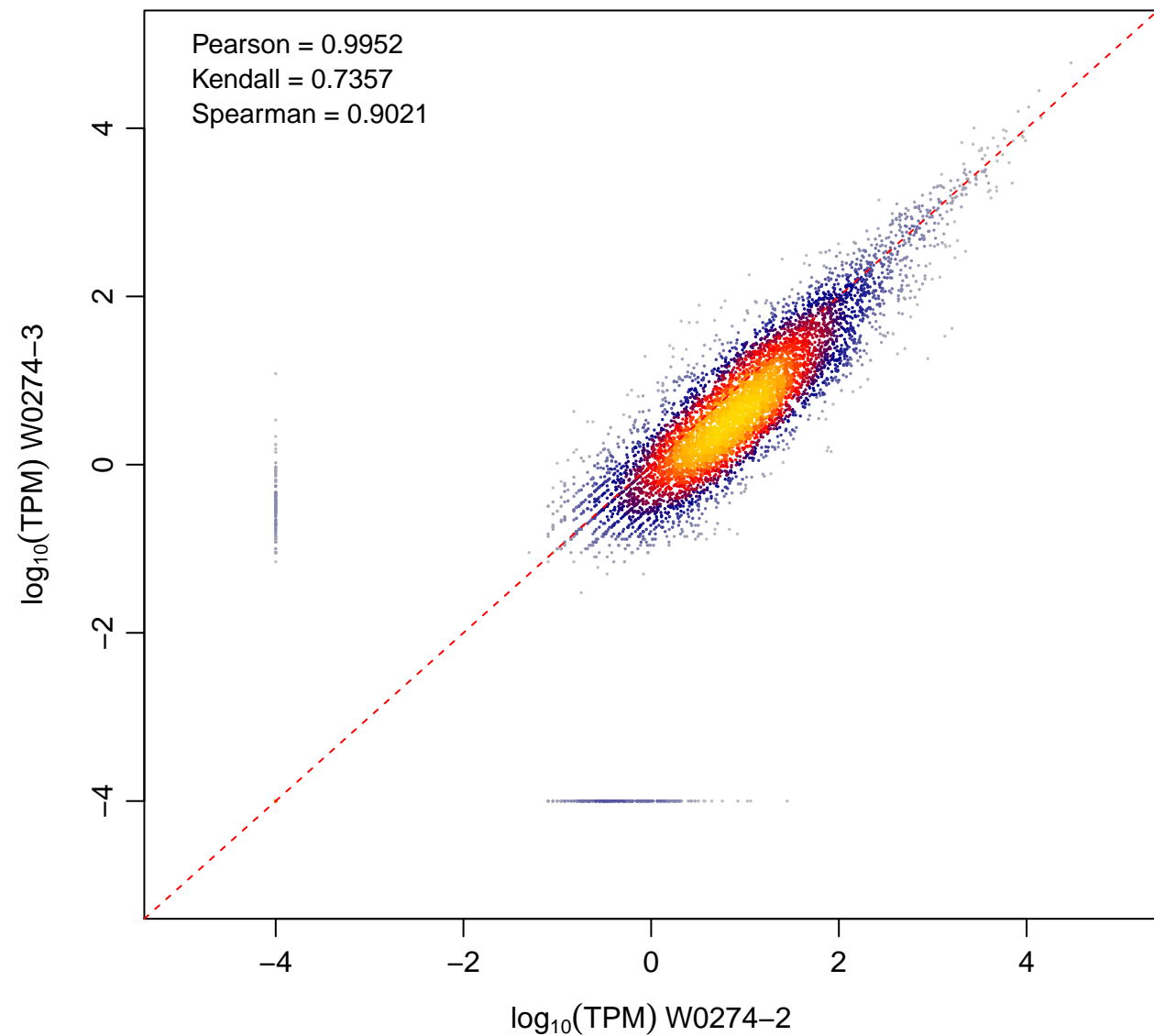

Supplement: Supplementary file 1 [file Supplementary_file_1.zip › RNA_seq_expression/replicate/W0274-3_vs_W0274-2.gene_tpm_replicate_correlation.pdf]

# WINH-2 VS WINH-1

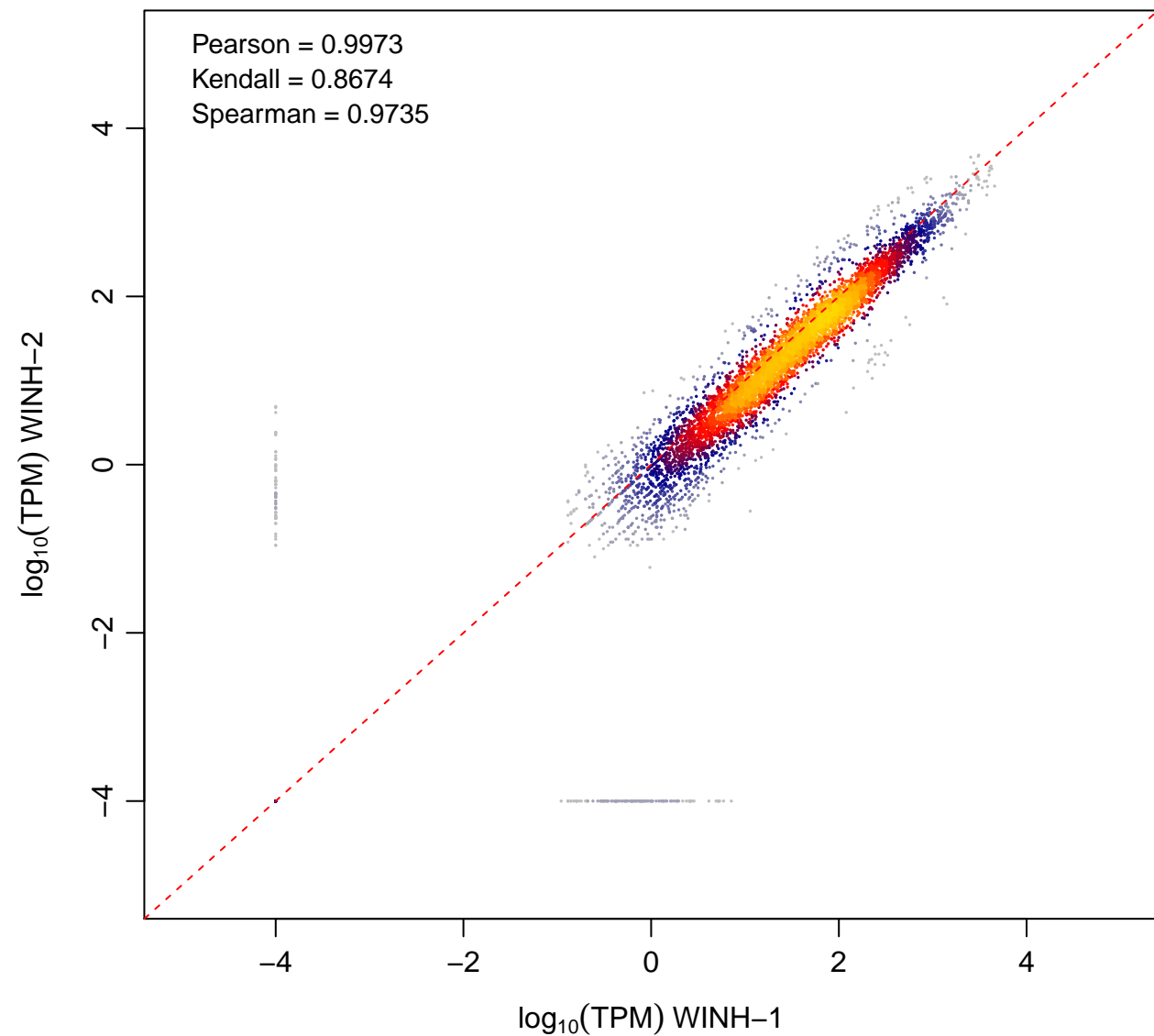

Supplement: Supplementary file 1 [file Supplementary_file_1.zip › RNA_seq_expression/replicate/WINH-2_vs_WINH-1.gene_tpm_replicate_correlation.pdf]

# WINH-3 VS WINH-1

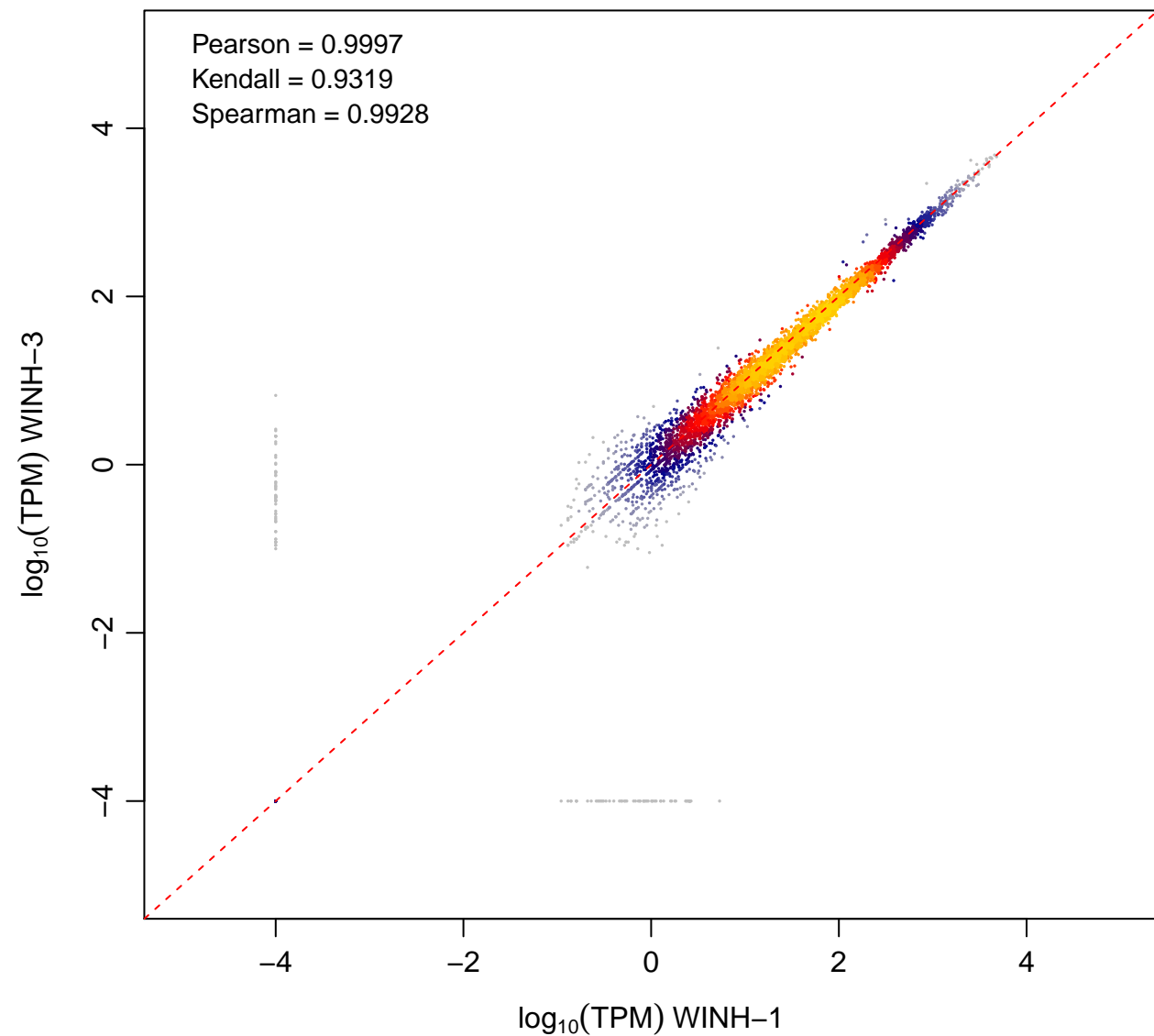

Supplement: Supplementary file 1 [file Supplementary_file_1.zip › RNA_seq_expression/replicate/WINH-3_vs_WINH-1.gene_tpm_replicate_correlation.pdf]

# WINH-3 VS WINH-2

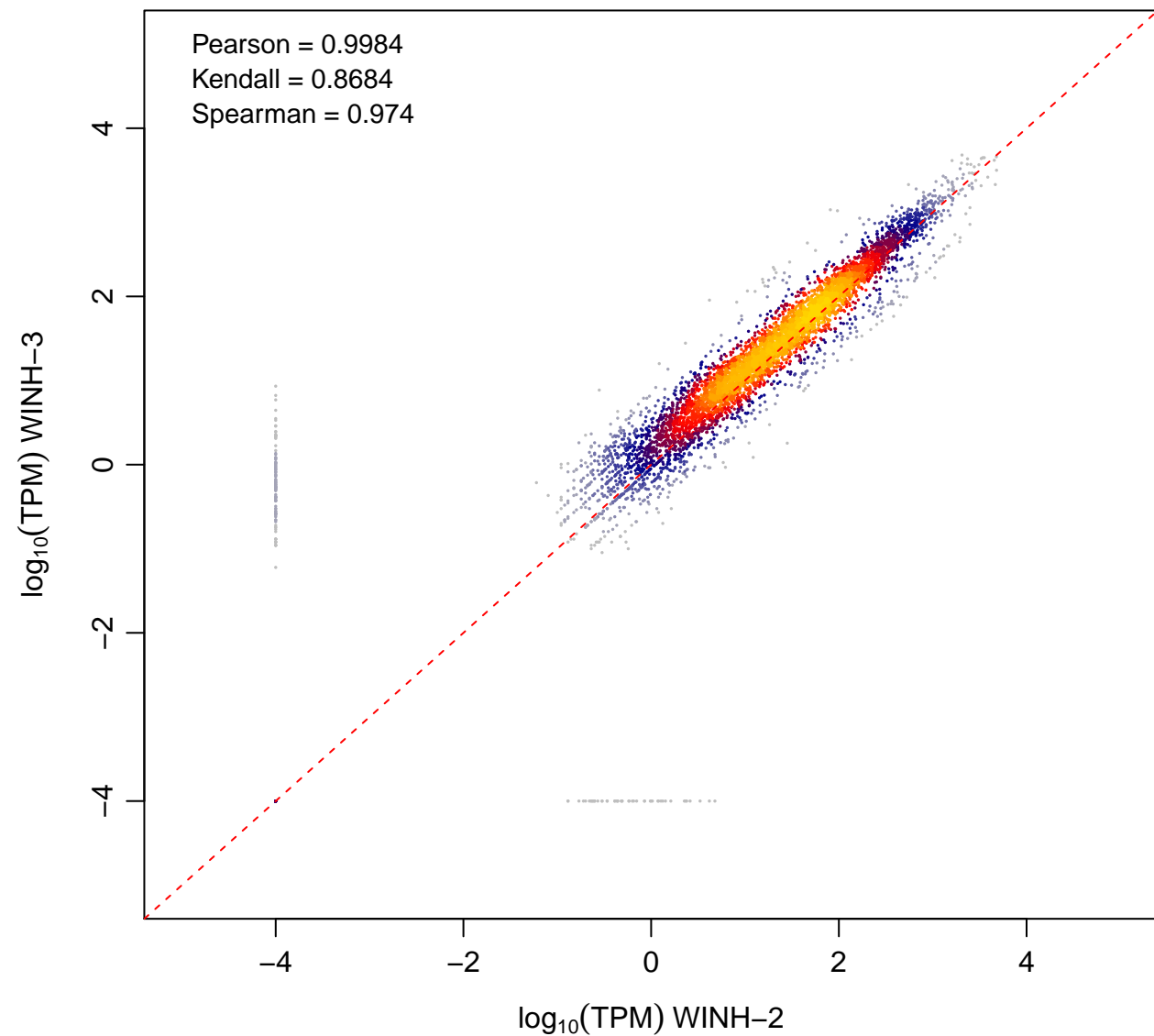

Supplement: Supplementary file 1 [file Supplementary_file_1.zip › RNA_seq_expression/replicate/WINH-3_vs_WINH-2.gene_tpm_replicate_correlation.pdf]

### Saturation curves (D0274-1)

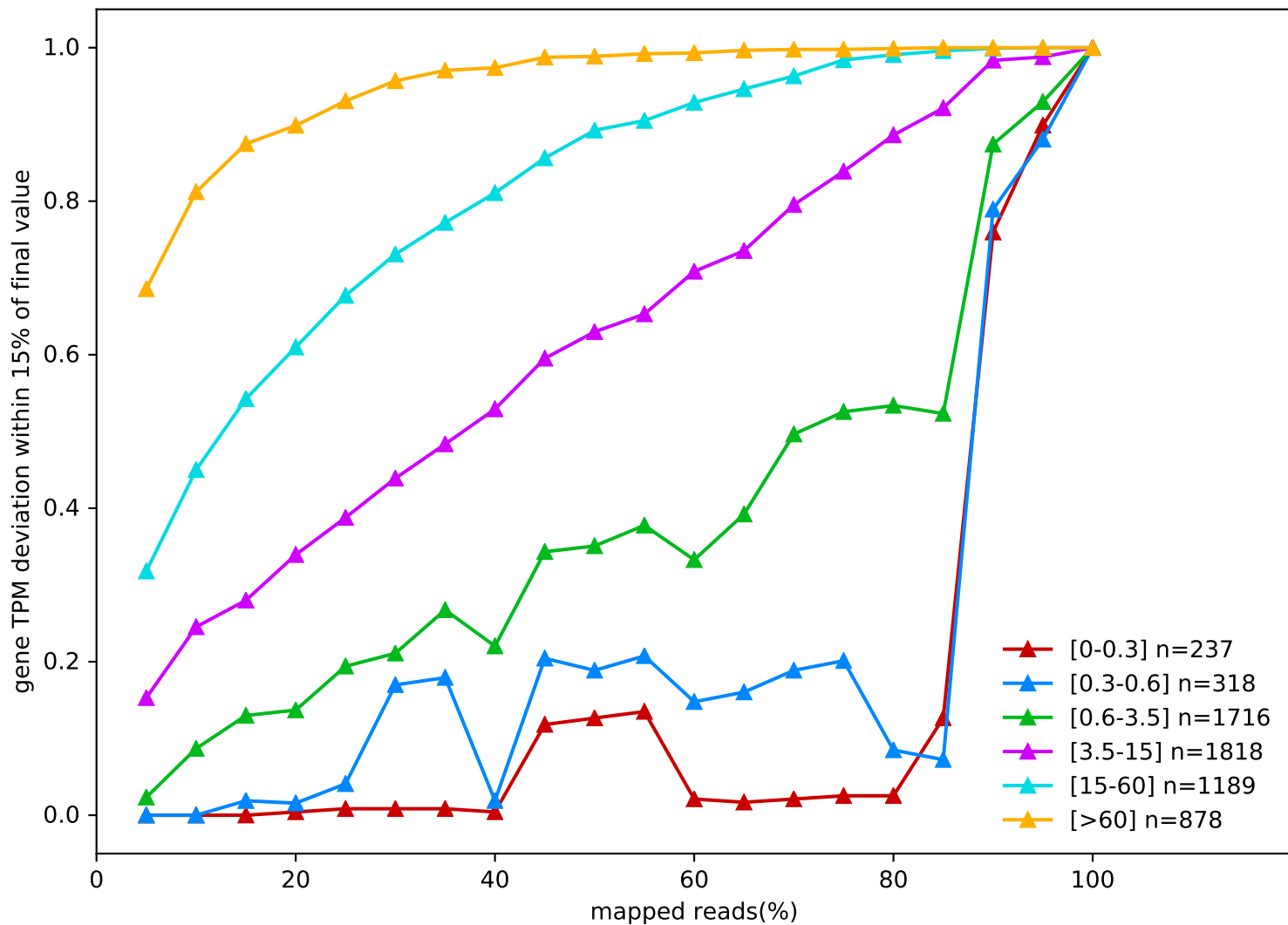

Supplement: Supplementary file 1 [file Supplementary_file_1.zip › RNA_seq_expression/tpm_saturation/D0274-1.tpm_saturation.pdf]

### Saturation curves (D0274-2)

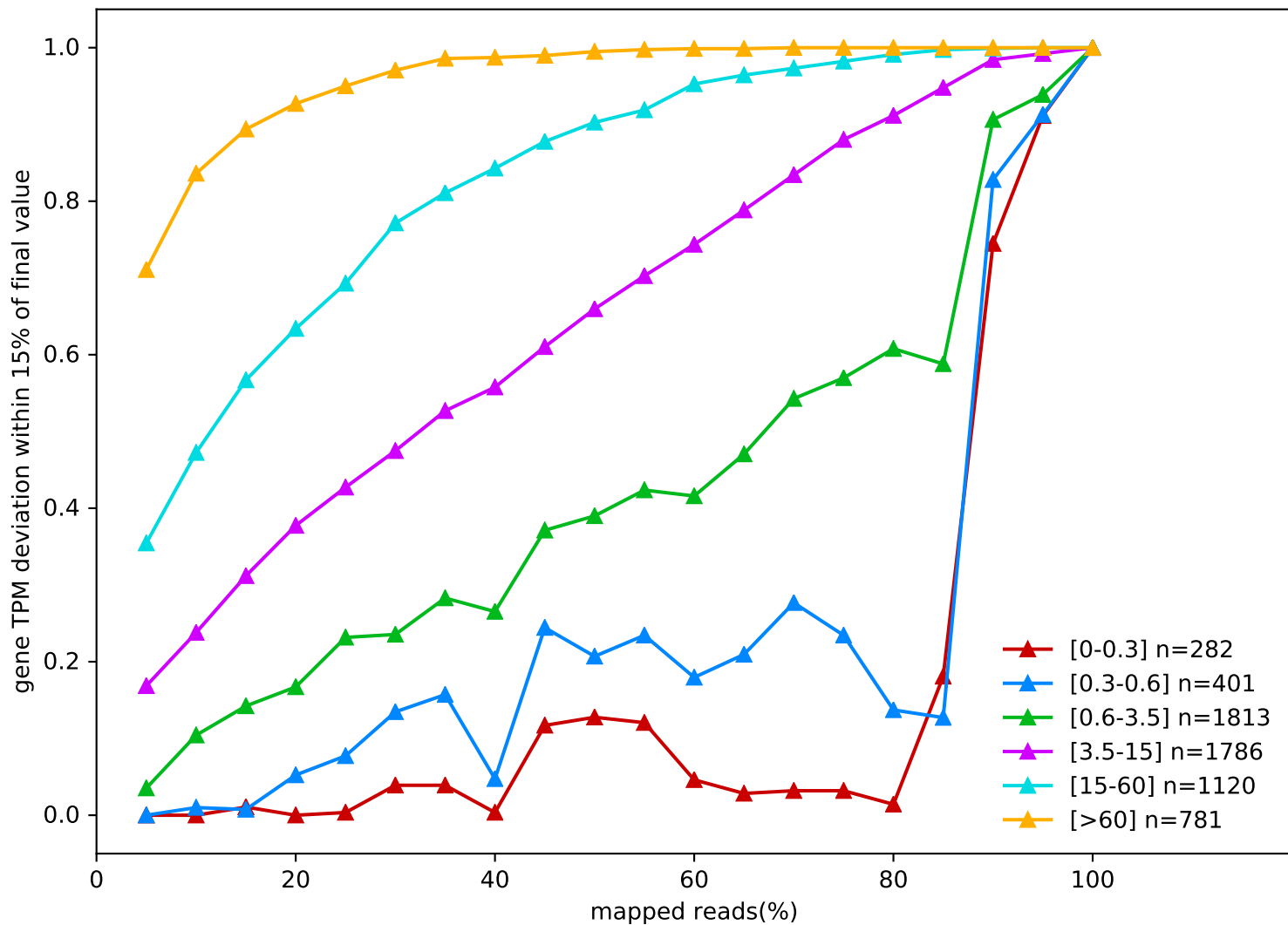

Supplement: Supplementary file 1 [file Supplementary_file_1.zip › RNA_seq_expression/tpm_saturation/D0274-2.tpm_saturation.pdf]

### Saturation curves (D0274-3)

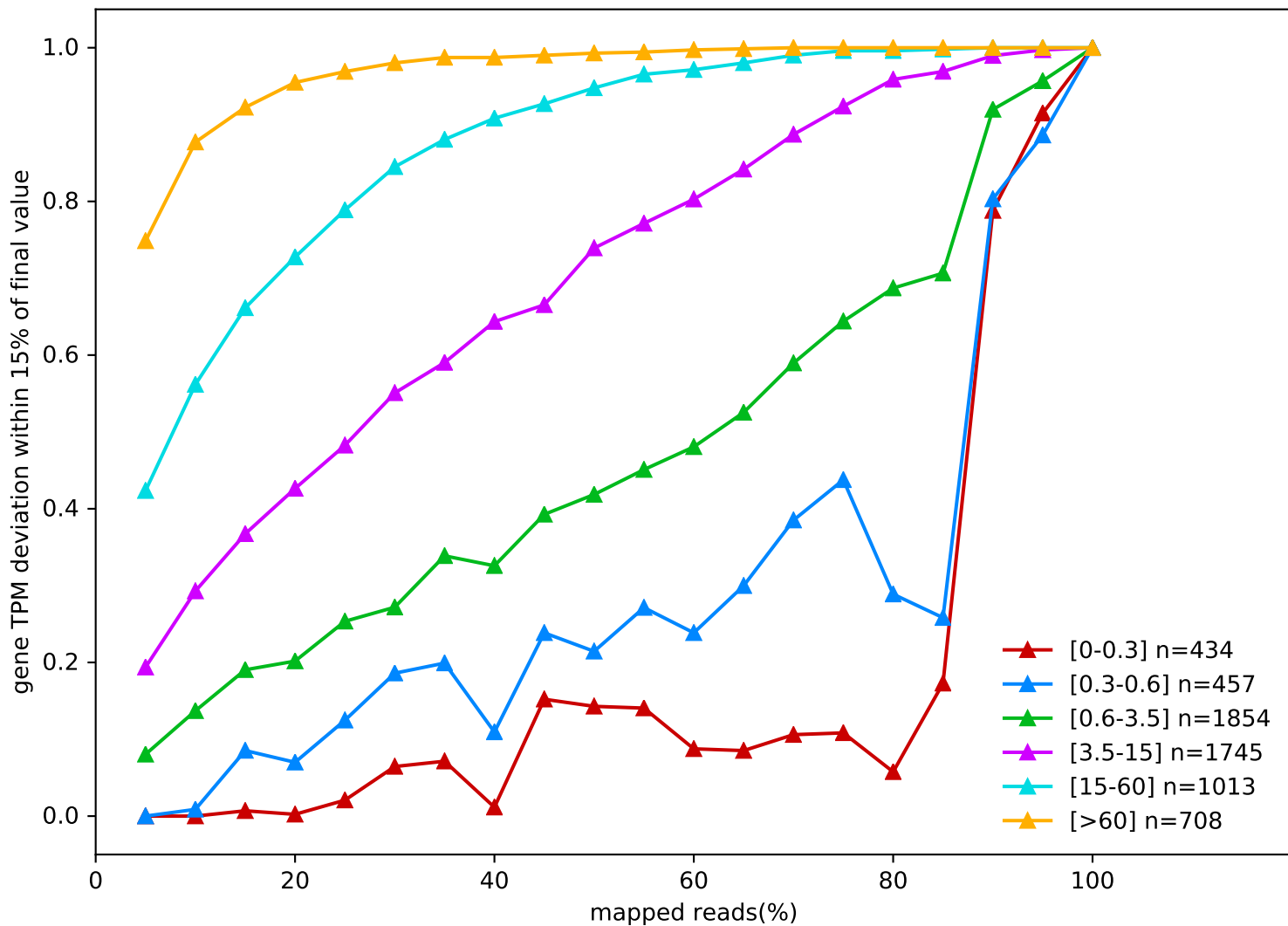

Supplement: Supplementary file 1 [file Supplementary_file_1.zip › RNA_seq_expression/tpm_saturation/D0274-3.tpm_saturation.pdf]

### Saturation curves (DINH-1)

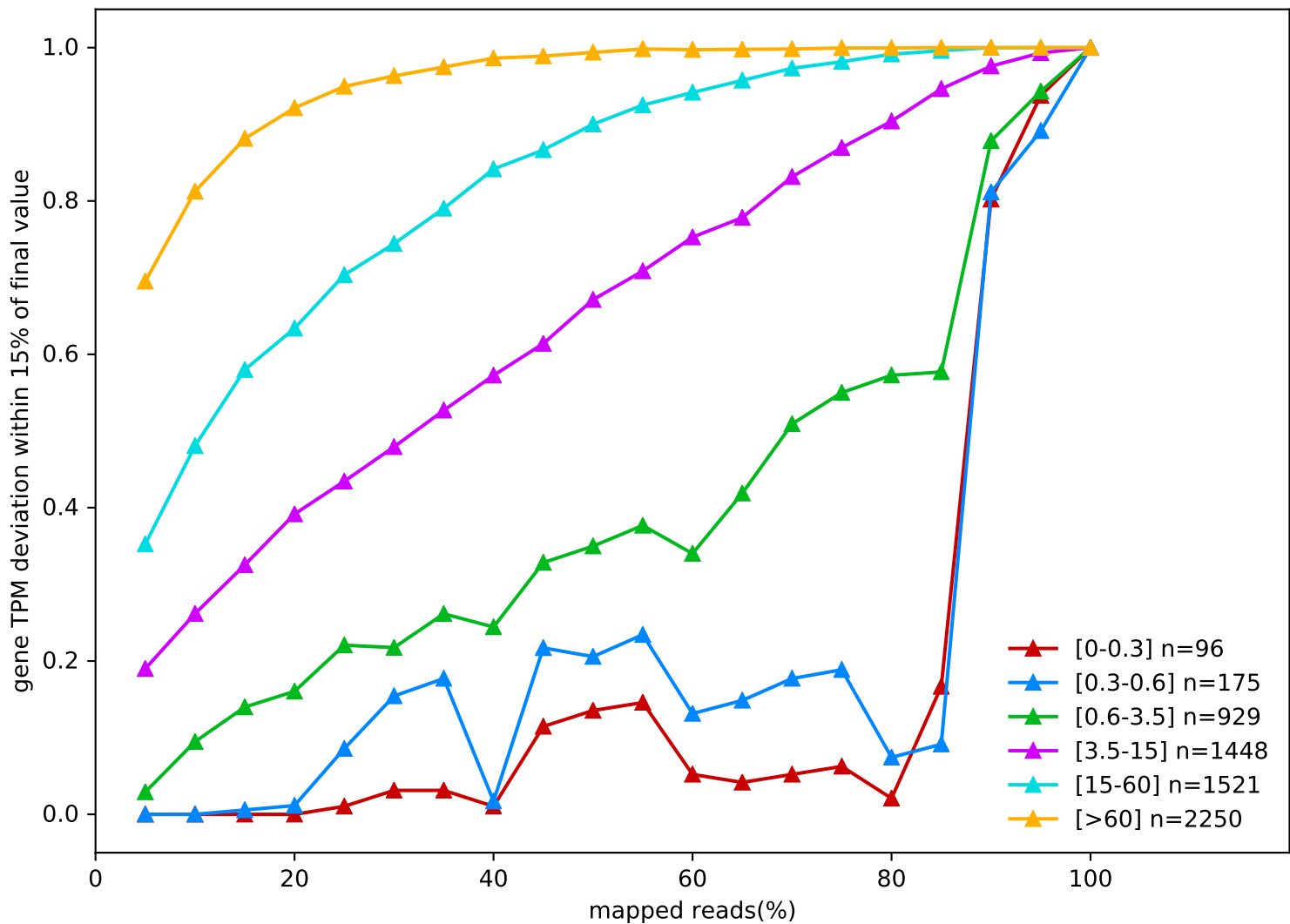

Supplement: Supplementary file 1 [file Supplementary_file_1.zip › RNA_seq_expression/tpm_saturation/DINH-1.tpm_saturation.pdf]

## Saturation curves (DINH-2)

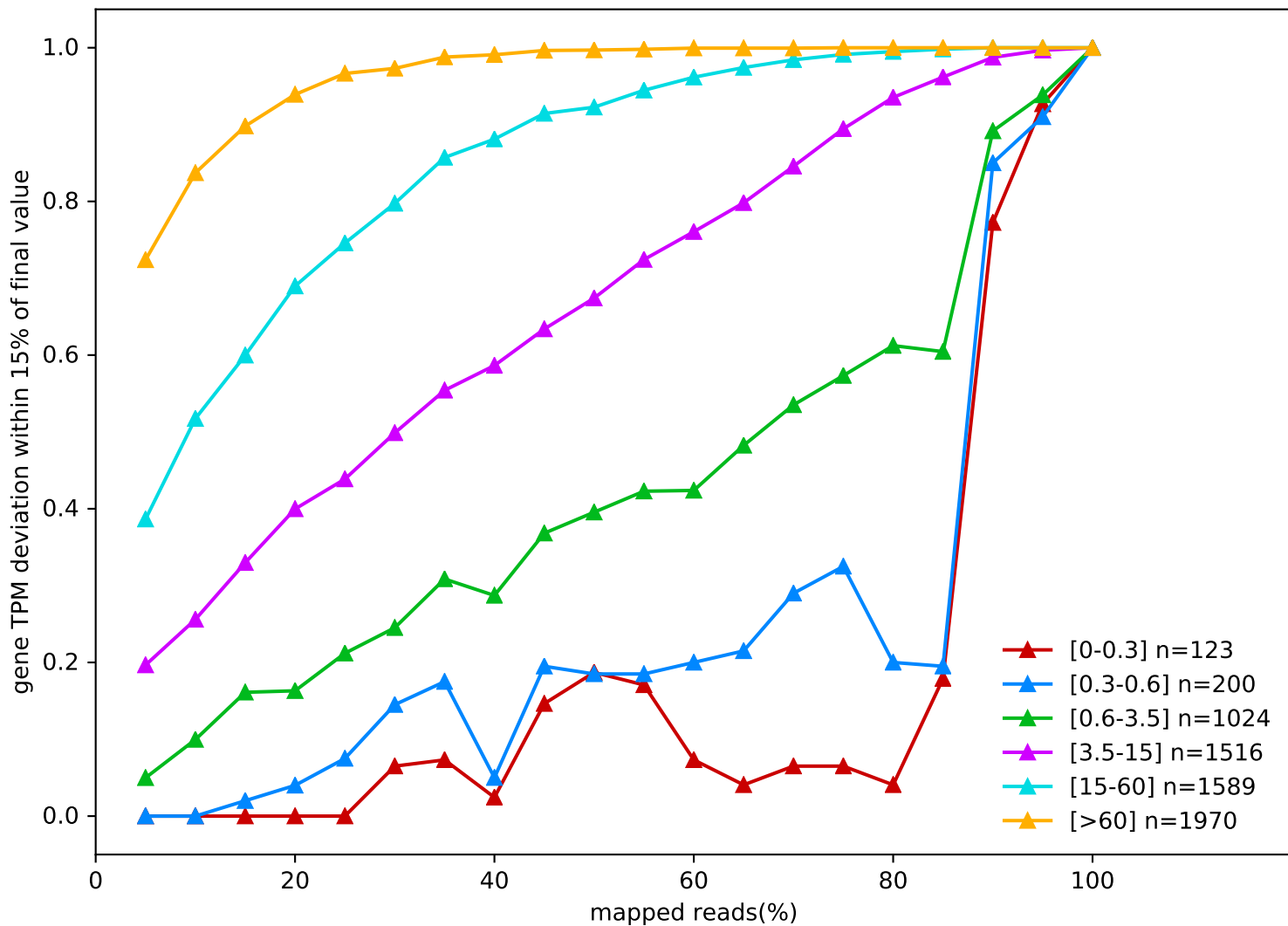

Supplement: Supplementary file 1 [file Supplementary_file_1.zip › RNA_seq_expression/tpm_saturation/DINH-2.tpm_saturation.pdf]

### Saturation curves (DINH-3)

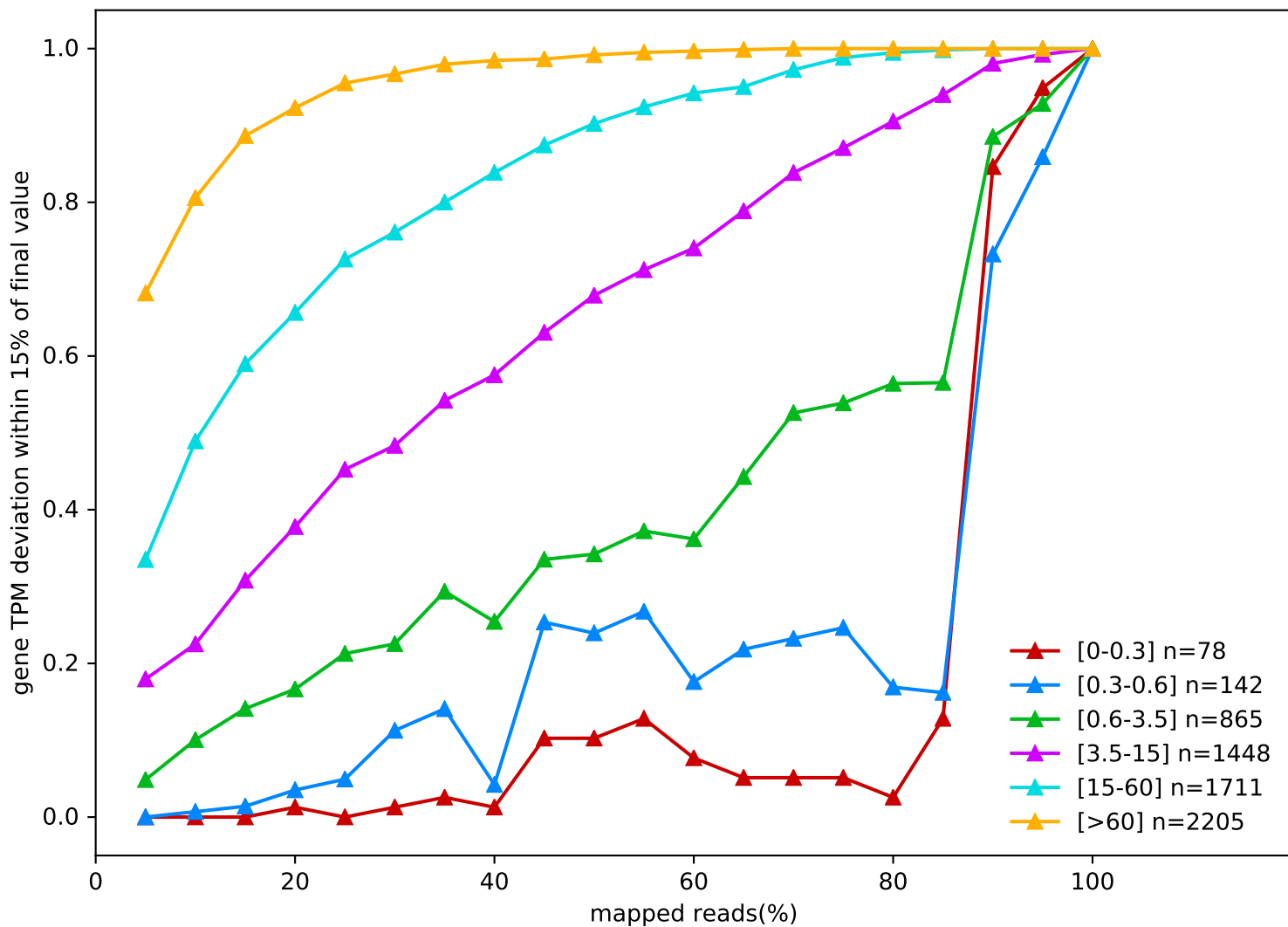

Supplement: Supplementary file 1 [file Supplementary_file_1.zip › RNA_seq_expression/tpm_saturation/DINH-3.tpm_saturation.pdf]

### Saturation curves (W0274-1)

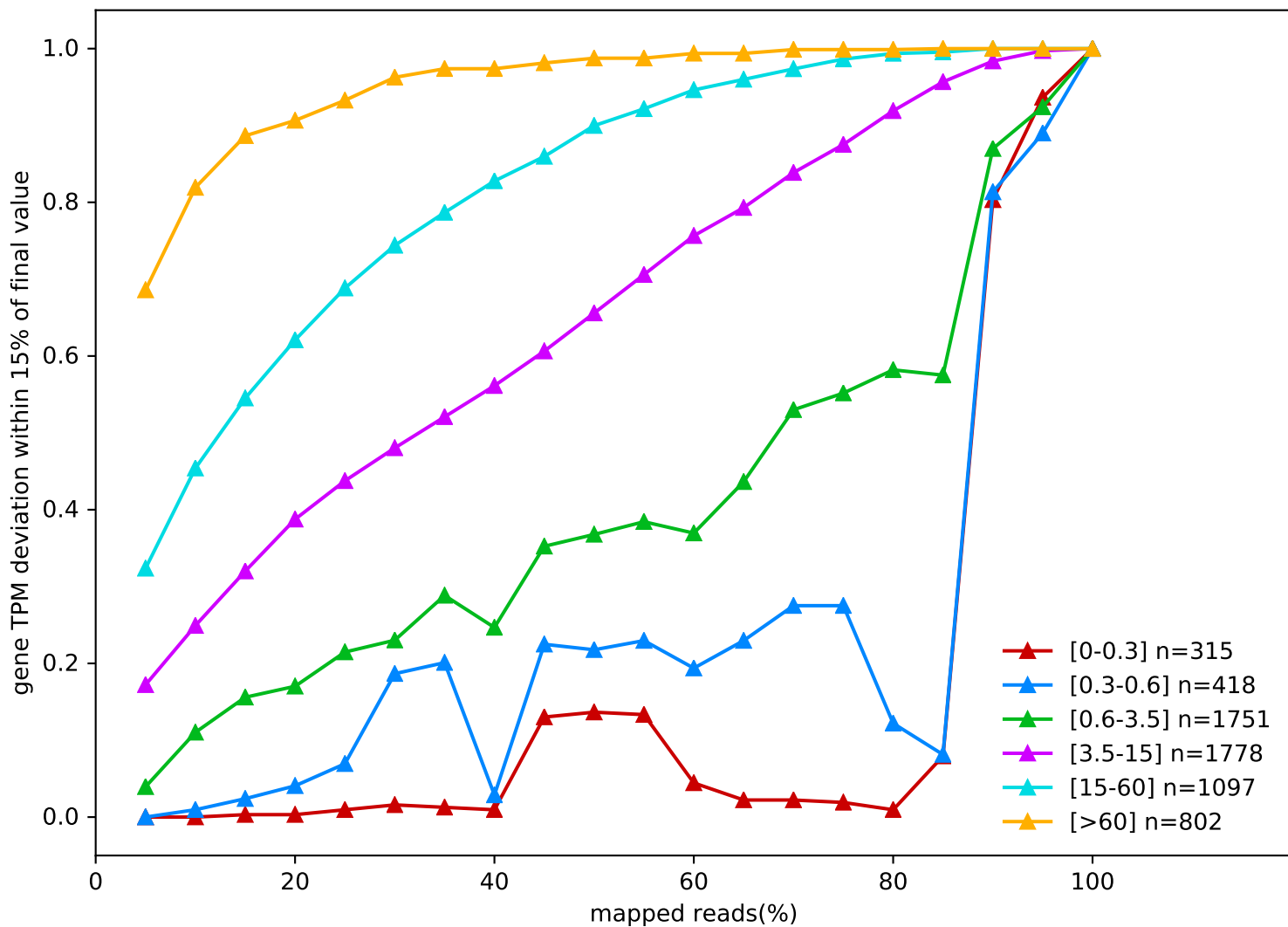

Supplement: Supplementary file 1 [file Supplementary_file_1.zip › RNA_seq_expression/tpm_saturation/W0274-1.tpm_saturation.pdf]

### Saturation curves (W0274-2)

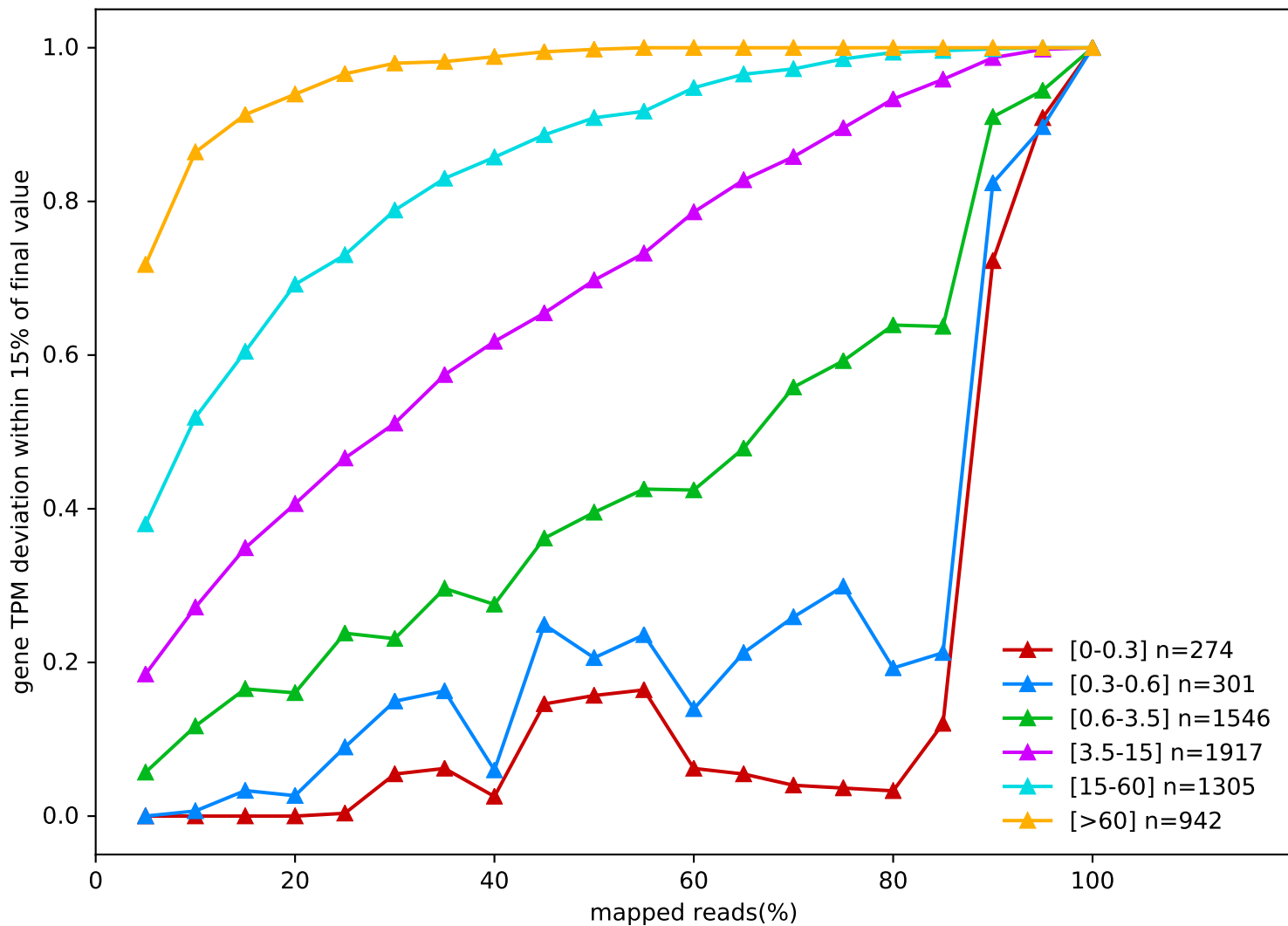

Supplement: Supplementary file 1 [file Supplementary_file_1.zip › RNA_seq_expression/tpm_saturation/W0274-2.tpm_saturation.pdf]

### Saturation curves (W0274-3)

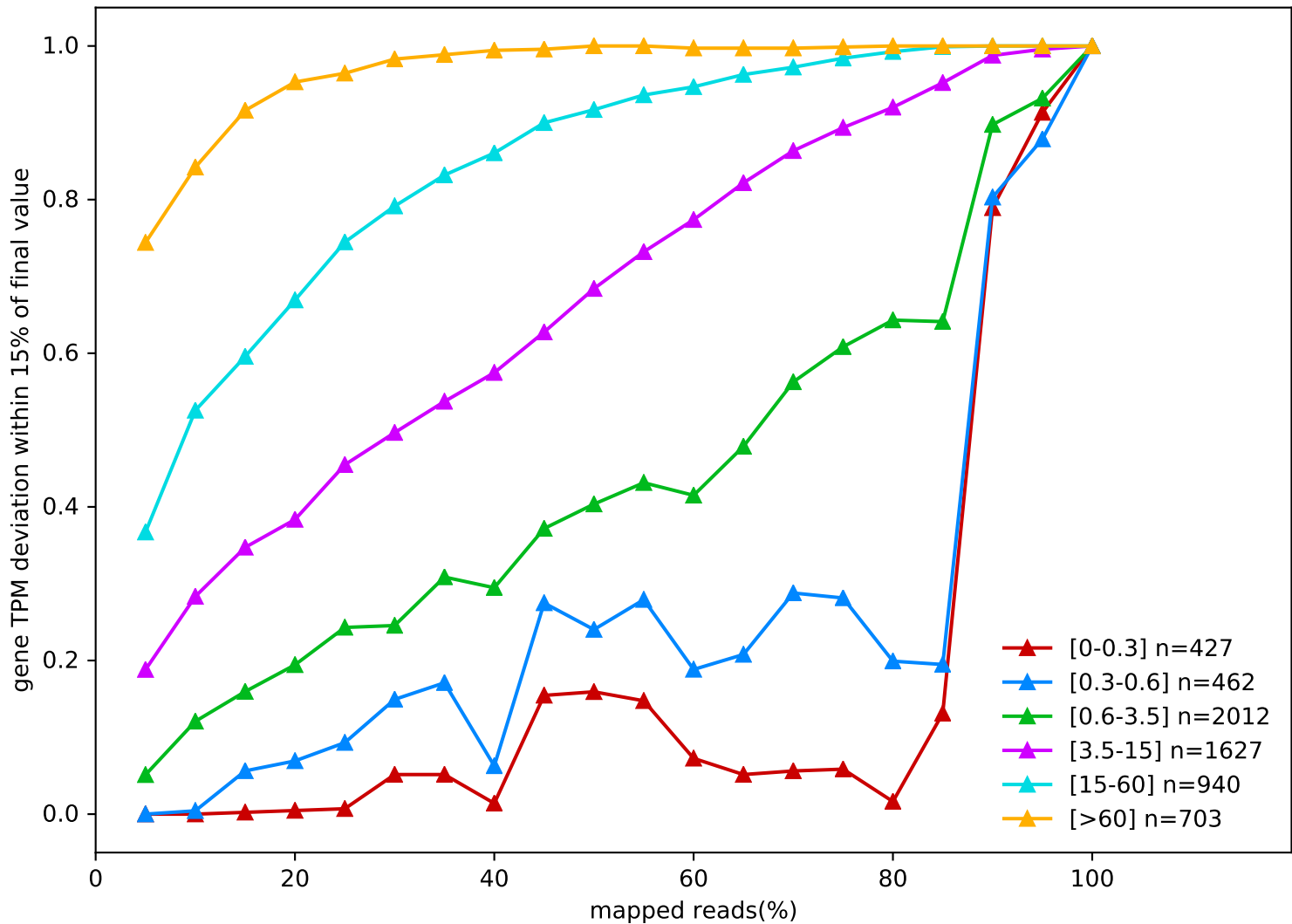

Supplement: Supplementary file 1 [file Supplementary_file_1.zip › RNA_seq_expression/tpm_saturation/W0274-3.tpm_saturation.pdf]

### Saturation curves (WINH-1)

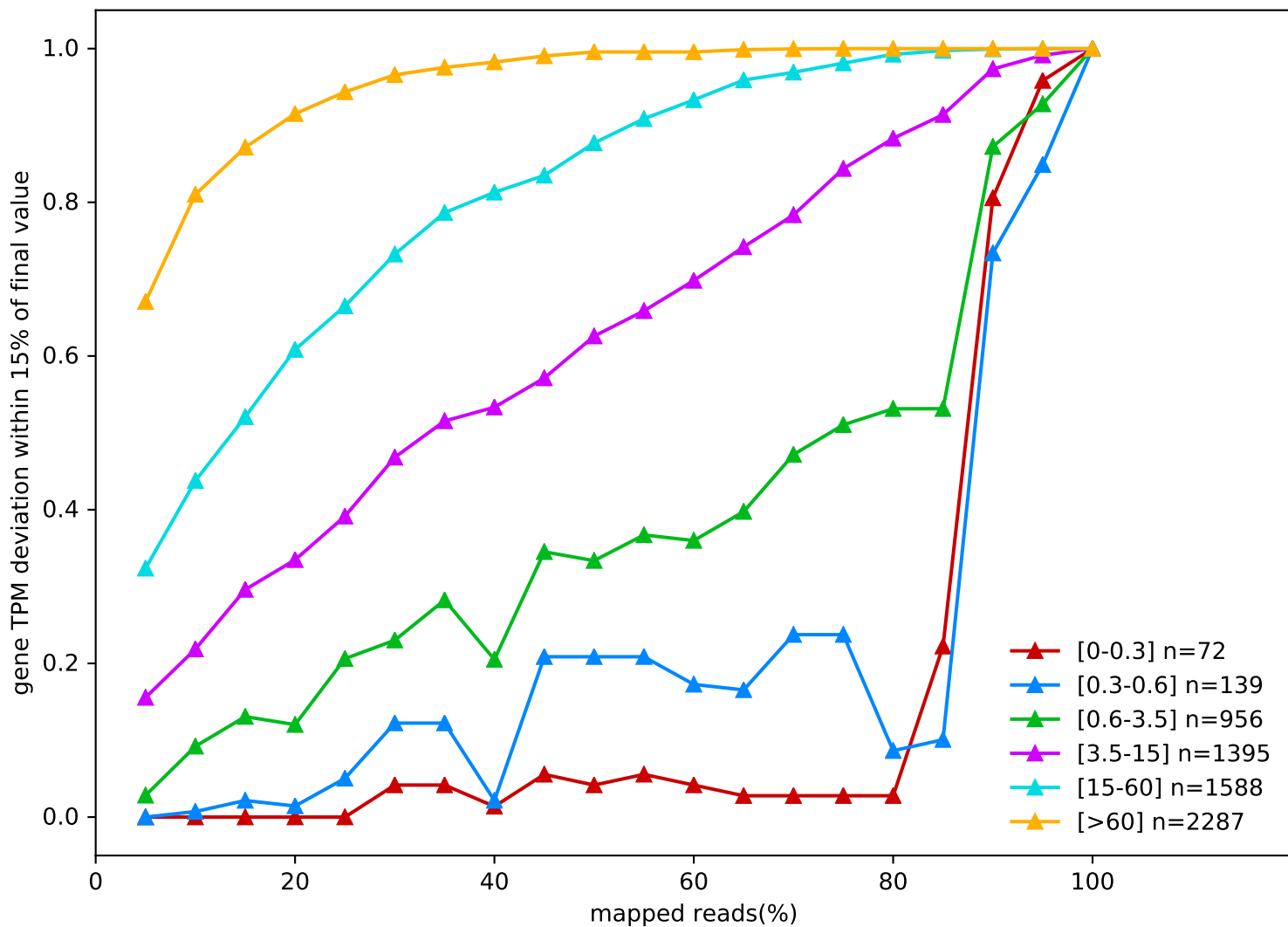

Supplement: Supplementary file 1 [file Supplementary_file_1.zip › RNA_seq_expression/tpm_saturation/WINH-1.tpm_saturation.pdf]

## Saturation curves (WINH-2)

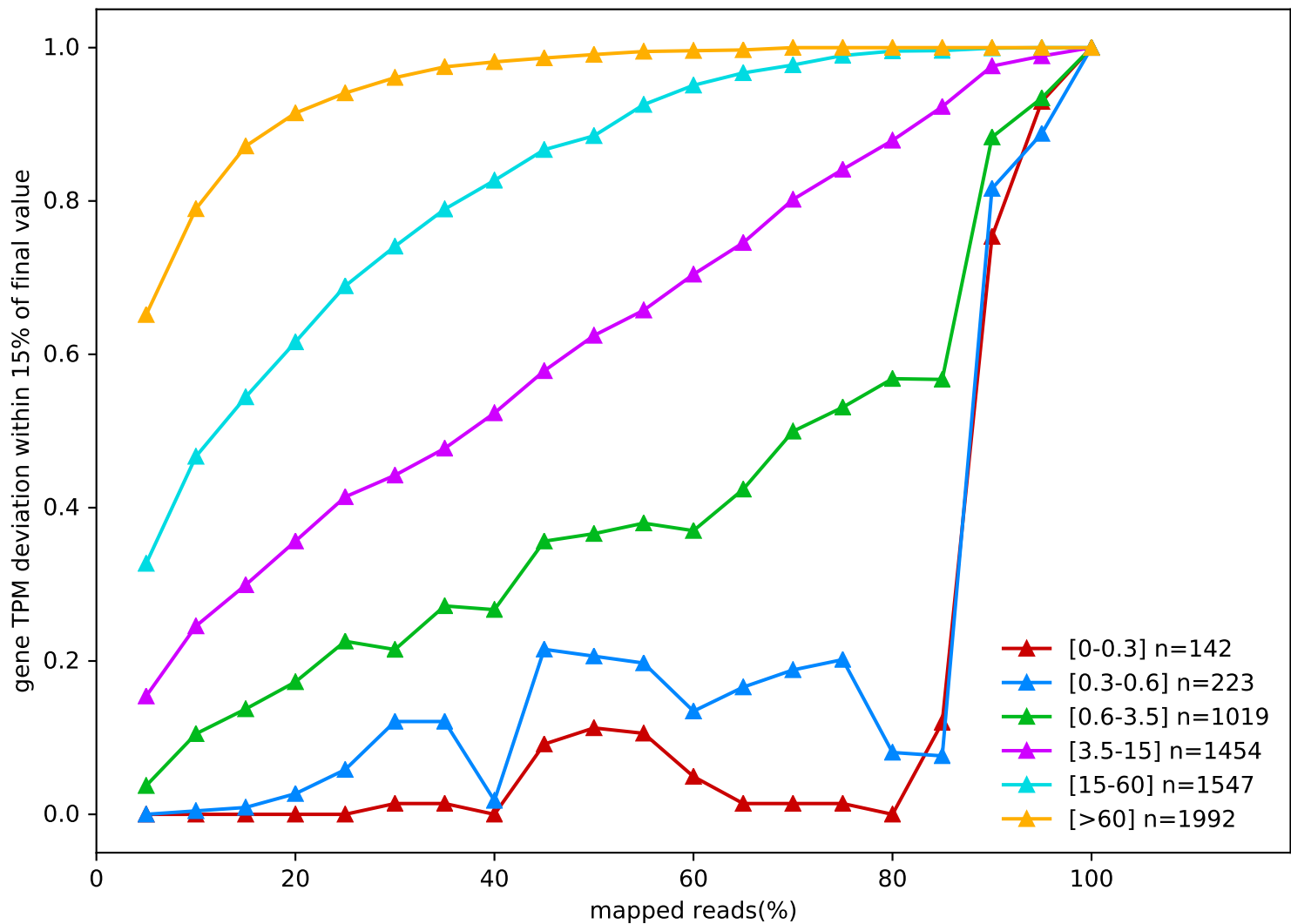

Supplement: Supplementary file 1 [file Supplementary_file_1.zip › RNA_seq_expression/tpm_saturation/WINH-2.tpm_saturation.pdf]

### Saturation curves (WINH-3)

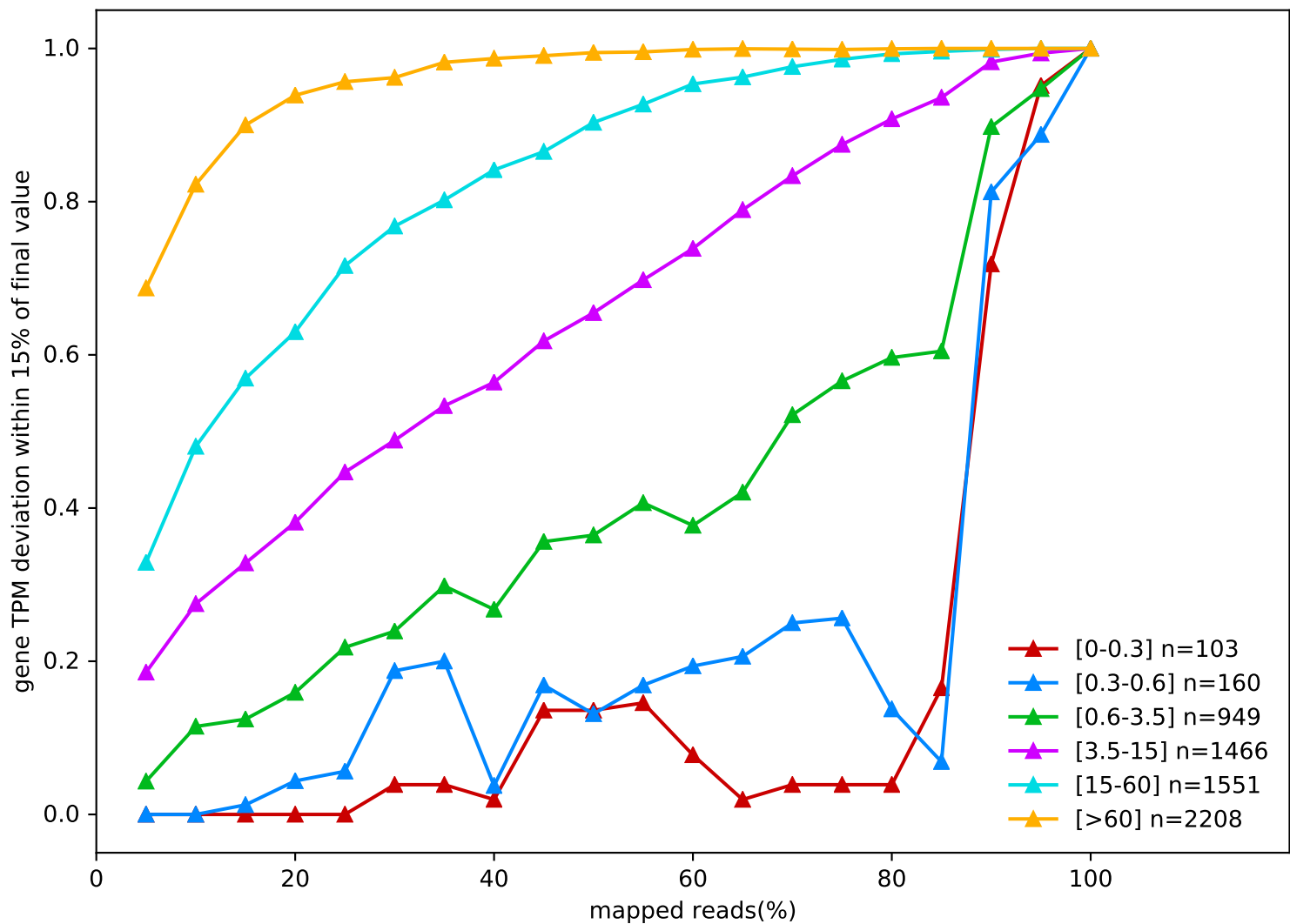

Supplement: Supplementary file 1 [file Supplementary_file_1.zip › RNA_seq_expression/tpm_saturation/WINH-3.tpm_saturation.pdf]

W0274-1

W0274-2

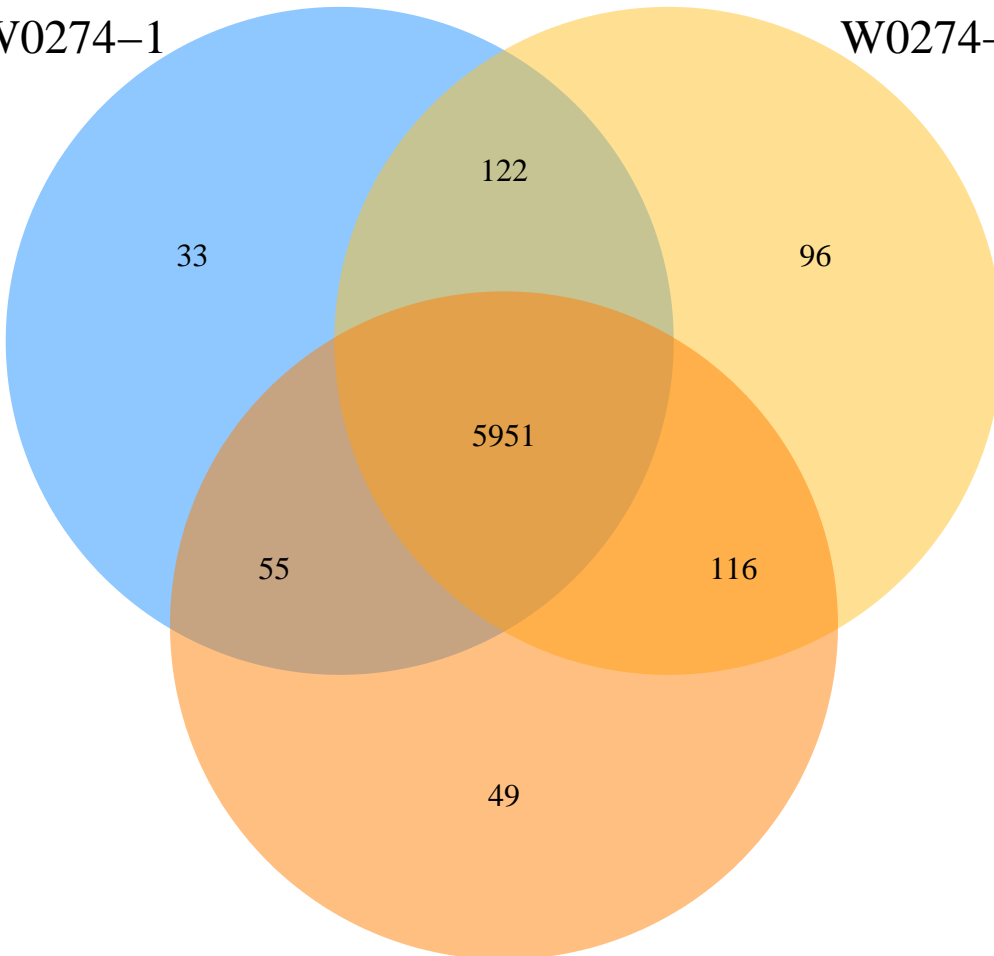

W0274-3

Supplement: Supplementary file 1 [file Supplementary_file_1.zip › RNA_seq_expression/venn/A.gene_tpm_venn.pdf]

D0274-1

D0274-2

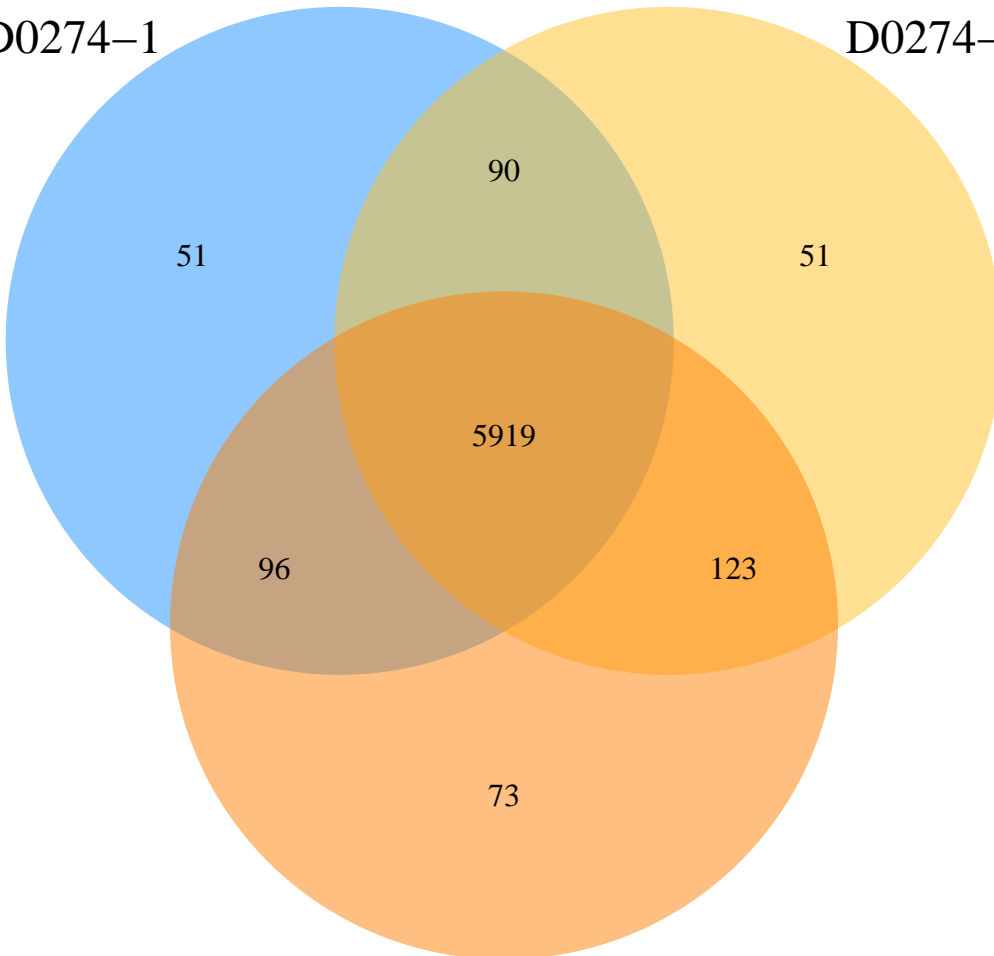

D0274-3

Supplement: Supplementary file 1 [file Supplementary_file_1.zip › RNA_seq_expression/venn/B.gene_tpm_venn.pdf]

WINH-1

WINH-2

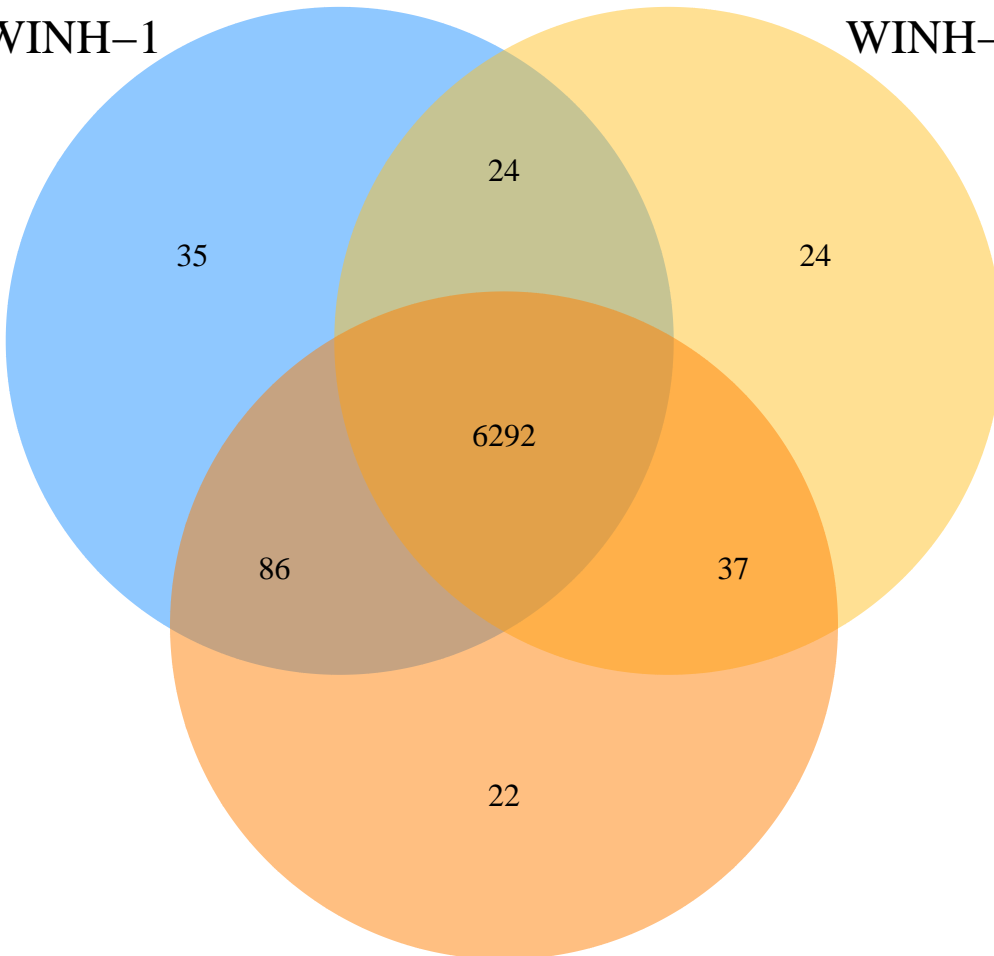

WINH-3

Supplement: Supplementary file 1 [file Supplementary_file_1.zip › RNA_seq_expression/venn/C.gene_tpm_venn.pdf]

DINH-1

DINH-2

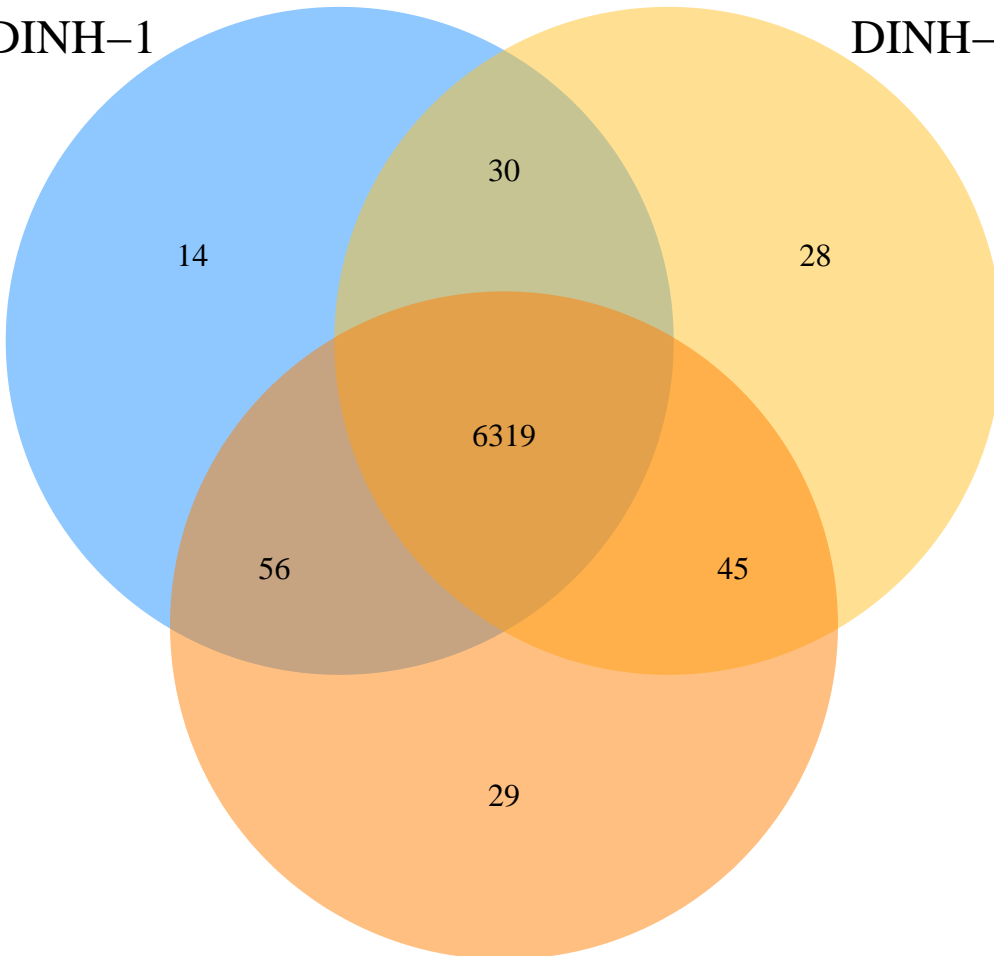

DINH-3

Supplement: Supplementary file 1 [file Supplementary_file_1.zip › RNA_seq_expression/venn/D.gene_tpm_venn.pdf]

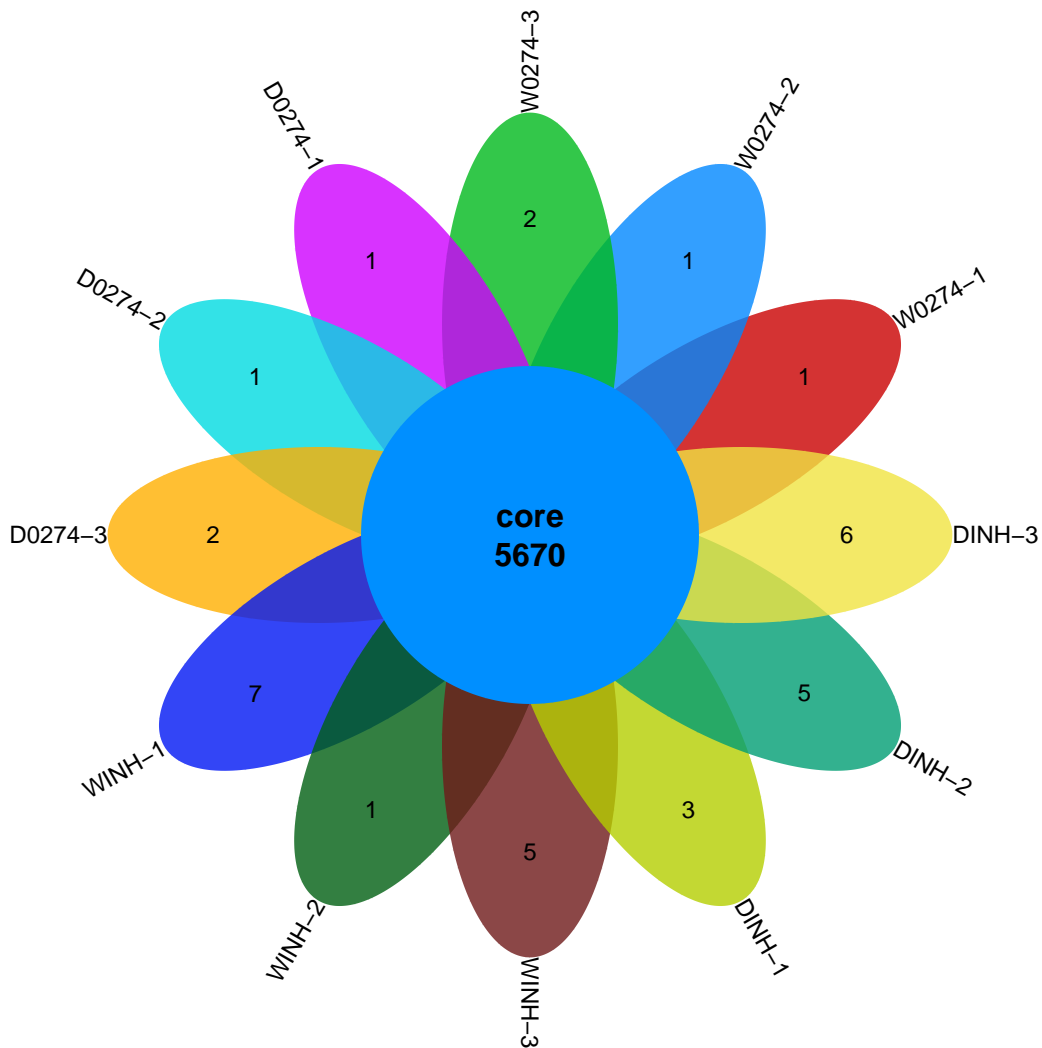

Supplement: Supplementary file 1 [file Supplementary_file_1.zip › RNA_seq_expression/venn/gene_tpm_core_flower.pdf]

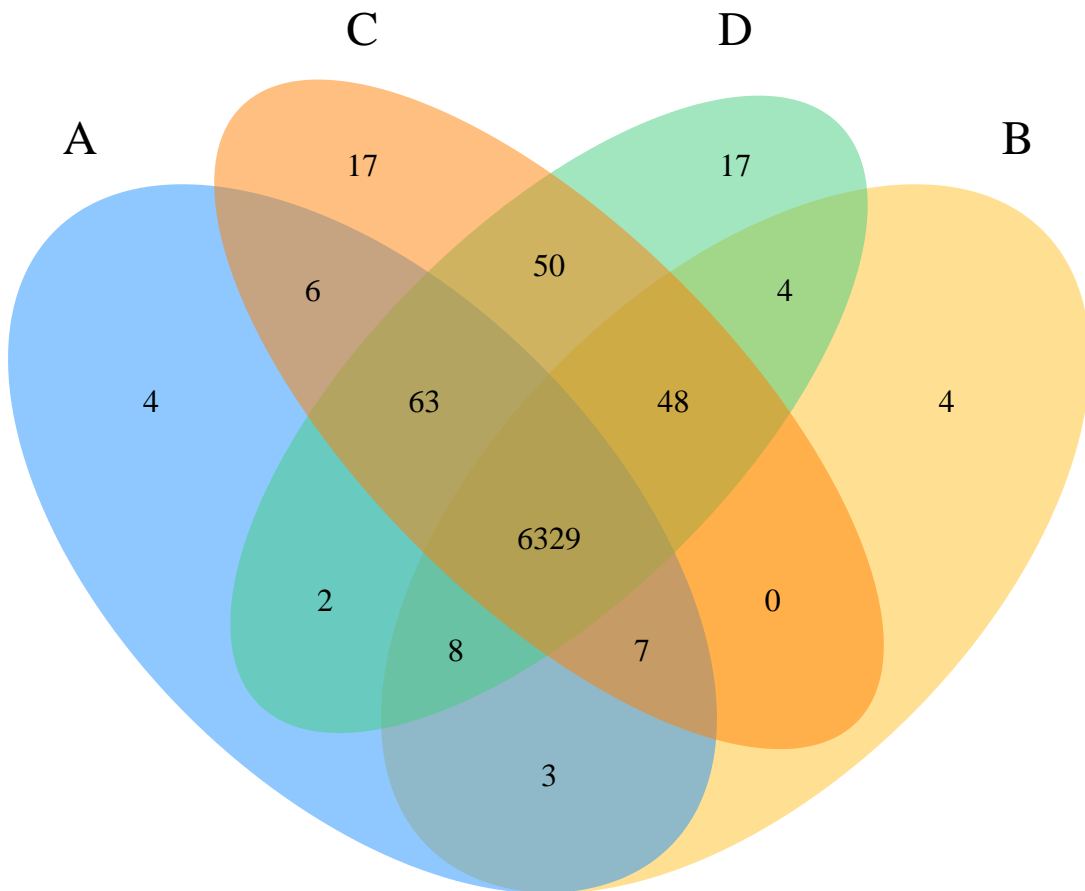

Supplement: Supplementary file 1 [file Supplementary_file_1.zip › RNA_seq_expression/venn/groupmerge.gene_tpm_venn.pdf]

TPM distribution violin

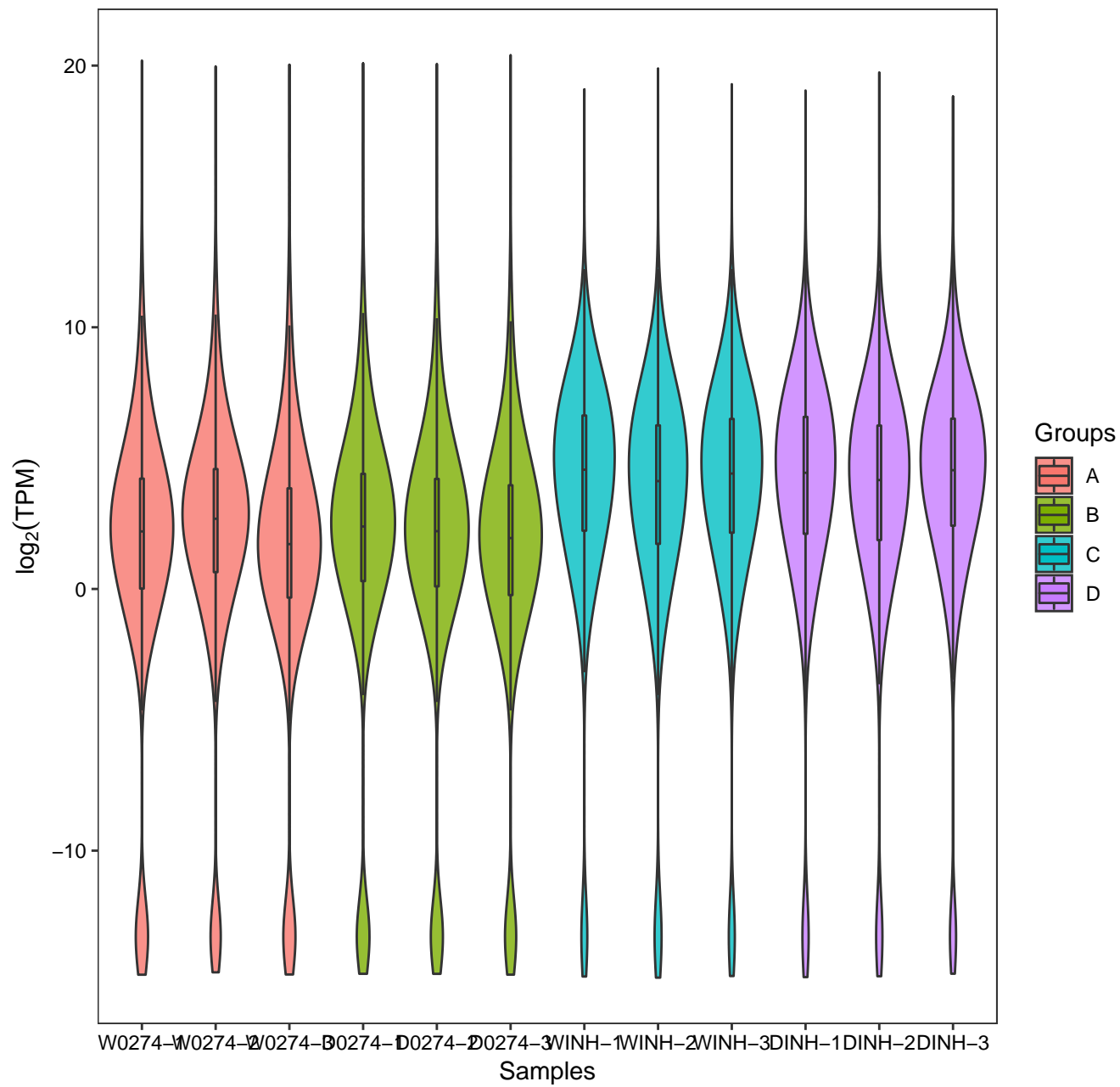

Supplement: Supplementary file 1 [file Supplementary_file_1.zip › RNA_seq_expression/violin/gene_tpm_violin.pdf]
