# Supplementary material for: Decoding drug tolerance: insights into the Rv0274 gene's role in isoniazid tolerance
Source: Front Microbiol. 2025 Nov 27;16:1697416. doi: 10.3389/fmicb.2025.1697416 (PMC12695796; doi:10.3389/fmicb.2025.1697416)
Supplement: Supplementary file 2 [file Data_Sheet_1.pdf]

Operon	IdGene	Type	COGgene	PosLeft	postRight	Strand	Function
1							
	MJLIPHIC_00001	CDS	COG0592	499	1692	+	[L] DNA polymerase sliding clamp subunit (PCNA
	MJLIPHIC_00002	CDS	COG1023	1721	2614	+	[G] Predicted 6-phosphogluconate dehydrogenase
	MJLIPHIC_00003	CDS	COG1195	2624	3778	+	[L] Recombinational DNA repair ATPase (RecF pathway)
	MJLIPHIC_00004	CDS	COG5512	3775	4359	+	[R] Zn-ribbon-containing, possibly RNA-binding protein and truncated
2							
	MJLIPHIC_00005	CDS	COG0187	4591	6618	+	[L] Type IIA topoisomerase (DNA gyrase/topo II,
	MJLIPHIC_00006	CDS	COG0188	6648	9176	+	[L] Type IIA topoisomerase (DNA gyrase/topo II,
	MJLIPHIC_00007	CDS	ROG0150	9229	10011	+	NA
	MJLIPHIC_00008	tRNA	NA	10072	10146	+	NA
	MJLIPHIC_00009	tRNA	NA	10293	10366	+	NA
	MJLIPHIC_00010	CDS	COG2375	10411	11211	+	[P] Siderophore-interacting protein
	MJLIPHIC_00011	CDS	COG0609	11215	12246	+	[P] ABC-type Fe3+-siderophore transport system, permease component
	MJLIPHIC_00012	CDS	COG4779	12243	13301	+	[P] ABC-type enterobactin transport system, permease component
	MJLIPHIC_00013	CDS	COG1120	13310	14140	+	[PH] ABC-type cobalamin/Fe3+-siderophores transport systems, ATPase components
3							
	MJLIPHIC_00014	CDS	COG0223	14130	15143	-	[J] Methionyl-tRNA formyltransferase
4							
	MJLIPHIC_00015	CDS	COG3251	15193	15522	+	[S] Uncharacterized protein conserved in bacteria
	MJLIPHIC_00016	CDS	COG1132	15525	17252	+	[V] ABC-type multidrug transport system, ATPase and
	MJLIPHIC_00017	CDS	COG1132	17249	19018	+	[V] ABC-type multidrug transport system, ATPase and
	MJLIPHIC_00018	CDS	COG1020	19052	41623	+	[Q] Non-ribosomal peptide synthetase modules and related
5							
	MJLIPHIC_00019	CDS	COG0614	41689	42636	-	[P] ABC-type Fe3+-hydroxamate transport system, periplasmic component
6							
	MJLIPHIC_00020	CDS	COG0853	42944	43354	+	[H] Aspartate 1-decarboxylase
	MJLIPHIC_00021	CDS	COG3486	43366	44688	+	[Q] Lysine/ornithine N-monooxygenase
7							
	MJLIPHIC_00022	CDS	ROG0780	44692	45102	-	NA
8							
	MJLIPHIC_00023	CDS	COG0652	45223	45750	+	[O] Peptidyl-prolyl cis-trans isomerase (rotamase) - cyclophilin
9							
	MJLIPHIC_00024	CDS	ROG4686	45854	46195	-	NA
10							
	MJLIPHIC_00025	CDS	ROG0246	46417	46701	-	NA
11							
	MJLIPHIC_00026	CDS	COG3879	46797	47546	+	[S] Uncharacterized protein conserved in bacteria
	MJLIPHIC_00027	CDS	COG0512	47564	48238	+	[EH] Anthranilate/para-aminobenzoate synthases component II
12							
	MJLIPHIC_00028	CDS	COG2815	48216	50093	-	[S] Uncharacterized protein conserved in bacteria
	MJLIPHIC_00029	CDS	COG0515	50090	51349	-	[RTKL] Serine/threonine protein kinase
	MJLIPHIC_00030	CDS	COG0768	51381	52856	-	[M] Cell division protein FtsI/penicillin-binding protein 2
	MJLIPHIC_00031	CDS	COG0772	52853	54268	-	[D] Bacterial cell division membrane protein
	MJLIPHIC_00032	CDS	COG0631	54265	55803	-	[T] Serine/threonine protein phosphatase
	MJLIPHIC_00033	CDS	COG1716	55826	56293	-	[T] FOG: FHA domain
	MJLIPHIC_00034	CDS	COG1716	56429	57874	-	[T] FOG: FHA domain
13							
	MJLIPHIC_00035	tRNA	NA	58144	58227	+	NA
14							
	MJLIPHIC_00036	CDS	COG2303	58478	58648	+	[E] Choline dehydrogenase and related flavoproteins
15							
	MJLIPHIC_00037	CDS	ROG8227	58655	59029	-	NA
	MJLIPHIC_00038	CDS	ROG1461	59041	59346	-	NA
	MJLIPHIC_00039	CDS	NA	59348	59824	-	NA
16							
	MJLIPHIC_00040	CDS	COG1309	59900	60463	+	[K] Transcriptional regulator
17							
	MJLIPHIC_00041	CDS	COG3384	60479	61252	-	[S] Uncharacterized conserved protein
18							
	MJLIPHIC_00042	CDS	ROG2848	61470	63548	+	NA
	MJLIPHIC_00043	CDS	NA	63545	66013	+	NA
	MJLIPHIC_00044	CDS	ROG4845	66010	67167	+	NA
19							
	MJLIPHIC_00045	CDS	ROG0995	67182	68117	-	NA
20							
	MJLIPHIC_00046	CDS	ROG5690	68264	68767	+	NA
21							
	MJLIPHIC_00047	CDS	COG1741	68769	69734	-	[R] Pirin-related protein
22							
	MJLIPHIC_00048	CDS	COG3599	69788	70045	+	[D] Cell division initiation protein
23							
	MJLIPHIC_00049	CDS	ROG0901	70234	70527	-	NA
24							
	MJLIPHIC_00050	CDS	COG2932	70911	71690	+	[K] Predicted transcriptional regulator
25							
	MJLIPHIC_00051	CDS	COG3335	72116	72823	+	[L] Transposase and inactivated derivatives
26							
	MJLIPHIC_00052	CDS	COG0455	72820	74208	-	[D] ATPases involved in chromosome partitioning
27							
	MJLIPHIC_00053	CDS	NA	74600	76177	+	NA
	MJLIPHIC_00054	CDS	ROG5320	76213	76530	+	NA
	MJLIPHIC_00055	CDS	ROG4760	76535	77383	+	NA
	MJLIPHIC_00056	CDS	COG1909	77398	77925	+	[S] Uncharacterized protein conserved in archaea
	MJLIPHIC_00057	CDS	COG0464	77918	79642	+	[O] ATPases of the AAA+ class
	MJLIPHIC_00058	CDS	ROG5420	79650	81089	+	NA
	MJLIPHIC_00059	CDS	COG1674	81089	83317	+	[D] DNA segregation ATPase FtsK/SpoIIIE and related
	MJLIPHIC_00060	CDS	COG1674	83318	85099	+	[D] DNA segregation ATPase FtsK/SpoIIIE and related
28							
	MJLIPHIC_00061	CDS	COG0035	85248	85541	+	[F] Uracil phosphoribosyltransferase
	MJLIPHIC_00062	CDS	COG5651	85589	86911	+	[N] PPE-repeat proteins
	MJLIPHIC_00063	CDS	COG4842	86997	87299	+	[S] Uncharacterized protein conserved in bacteria
	MJLIPHIC_00064	CDS	COG4842	87331	87618	+	[S] Uncharacterized protein conserved in bacteria
29							
	MJLIPHIC_00065	CDS	COG0455	87738	89162	+	[D] ATPases involved in chromosome partitioning
	MJLIPHIC_00066	CDS	ROG3360	89159	90691	+	NA
30							
	MJLIPHIC_00067	CDS	NA	91113	91874	+	NA
	MJLIPHIC_00068	CDS	NA	91906	93072	+	NA

31							
	MJLIPHIC_00069	CDS	NA	94608	95339	+	NA
32							
	MJLIPHIC_00070	CDS	COG5421	95440	96300	-	[L] Transposase
33							
	MJLIPHIC_00071	CDS	COG5421	96424	97935	+	[L] Transposase
34							
	MJLIPHIC_00072	CDS	COG5421	97891	99105	-	[L] Transposase
35							
	MJLIPHIC_00073	CDS	NA	99229	99585	+	NA
36							
	MJLIPHIC_00074	CDS	NA	99798	101360	+	NA
37							
	MJLIPHIC_00075	CDS	NA	101470	101763	+	NA
38							
	MJLIPHIC_00076	CDS	ROG1823	101776	102093	-	NA
	MJLIPHIC_00077	CDS	NA	102127	102861	-	NA
39							
	MJLIPHIC_00078	CDS	NA	102936	103682	-	NA
	MJLIPHIC_00079	CDS	NA	103713	104243	-	NA
	MJLIPHIC_00080	CDS	COG0718	104251	104583	-	[S] Uncharacterized protein conserved in bacteria
	MJLIPHIC_00081	CDS	ROG7780	104594	105988	-	NA
	MJLIPHIC_00082	CDS	COG1404	105985	107334	-	[O] Subtilisin-like serine proteases
40							
	MJLIPHIC_00083	CDS	COG1925	107565	107822	-	[G] Phosphotransferase system, HPr-related proteins
	MJLIPHIC_00084	CDS	COG1299	107834	109849	-	[G] Phosphotransferase system, fructose-specific IIC component
	MJLIPHIC_00085	CDS	COG1105	109846	110829	-	[G] Fructose-1-phosphate kinase and related fructose-6-phosphate kinase
	MJLIPHIC_00086	CDS	COG1349	110826	111593	-	[KG] Transcriptional regulators of sugar metabolism
41							
	MJLIPHIC_00087	CDS	COG1080	111735	113369	+	[G] Phosphoenolpyruvate-protein kinase (PTS system EI component
42							
	MJLIPHIC_00088	CDS	COG1741	113433	114407	+	[R] Pirin-related protein
43							
	MJLIPHIC_00089	CDS	ROG8523	114447	116594	-	NA
	MJLIPHIC_00090	CDS	COG1309	116600	117184	-	[K] Transcriptional regulator
44							
	MJLIPHIC_00091	CDS	COG1309	117396	117941	+	[K] Transcriptional regulator
45							
	MJLIPHIC_00092	CDS	COG3315	118003	118965	+	[Q] O-Methyltransferase involved in polyketide biosynthesis
	MJLIPHIC_00093	CDS	COG3315	118962	119897	+	[Q] O-Methyltransferase involved in polyketide biosynthesis
46							
	MJLIPHIC_00094	CDS	NA	119878	120057	-	NA
47							
	MJLIPHIC_00095	CDS	COG1028	120252	121115	+	[IQR] Dehydrogenases with different specificities (related to
	MJLIPHIC_00096	CDS	COG0604	121119	122084	+	[CR] NADPH:quinone reductase and related Zn-dependent oxidoreductases
	MJLIPHIC_00097	CDS	COG0500	122105	122818	+	[QR] SAM-dependent methyltransferases
	MJLIPHIC_00098	CDS	ROG2627	122829	123314	+	NA
	MJLIPHIC_00099	CDS	COG2353	123311	123865	+	[S] Uncharacterized conserved protein
48							
	MJLIPHIC_00100	CDS	COG2365	123837	124643	-	[T] Protein tyrosine/serine phosphatase
	MJLIPHIC_00101	CDS	COG1960	124640	125854	-	[I] Acyl-CoA dehydrogenases
	MJLIPHIC_00102	CDS	NA	125900	127099	-	NA
	MJLIPHIC_00103	CDS	COG0583	127143	128024	-	[K] Transcriptional regulator
49							
	MJLIPHIC_00104	CDS	COG2197	128096	128734	+	[TK] Response regulator containing a CheY-like receiver
	MJLIPHIC_00105	CDS	COG4585	128750	129916	+	[T] Signal transduction histidine kinase
50							
	MJLIPHIC_00106	CDS	COG1316	129926	131527	-	[K] Transcriptional regulator
	MJLIPHIC_00107	CDS	COG1960	131570	132709	-	[I] Acyl-CoA dehydrogenases
	MJLIPHIC_00108	CDS	COG1282	132711	134153	-	[C] NAD/NADP transhydrogenase beta subunit
	MJLIPHIC_00109	CDS	COG3288	134172	135713	-	[C] NAD/NADP transhydrogenase alpha subunit
51							
	MJLIPHIC_00110	CDS	COG0598	135783	136787	-	[P] Mg2+ and Co2+ transporters
52							
	MJLIPHIC_00111	CDS	COG0600	136950	137810	+	[P] ABC-type nitrate/sulfonate/bicarbonate transport system, permease component
	MJLIPHIC_00112	CDS	COG4521	137817	138845	+	[P] ABC-type taurine transport system, periplasmic component
	MJLIPHIC_00113	CDS	COG1116	138829	139626	+	[P] ABC-type nitrate/sulfonate/bicarbonate transport system, ATPase component
53							
	MJLIPHIC_00114	CDS	COG1011	139607	140287	-	[R] Predicted hydrolase (HAD superfamily)
	MJLIPHIC_00115	CDS	COG0596	140284	141147	-	[R] Predicted hydrolases or acyltransferases (alpha/beta hydrolase
54							
	MJLIPHIC_00116	CDS	COG5516	141232	141732	+	[R] Conserved protein containing a Zn-ribbon-like motif,
55							
	MJLIPHIC_00117	CDS	COG1309	141969	142628	+	[K] Transcriptional regulator
	MJLIPHIC_00118	CDS	COG1028	142625	143398	+	[IQR] Dehydrogenases with different specificities (related to
56							
	MJLIPHIC_00119	CDS	COG3181	143535	144542	+	[S] Uncharacterized protein conserved in bacteria
	MJLIPHIC_00120	CDS	ROG0921	144542	145048	+	NA
	MJLIPHIC_00121	CDS	COG3333	145067	146593	+	[S] Uncharacterized protein conserved in bacteria
57							
	MJLIPHIC_00122	CDS	COG2186	146549	147232	-	[K] Transcriptional regulators
	MJLIPHIC_00123	CDS	COG4948	147229	148347	-	[MR] L-alanine-DL-glutamate epimerase and related enzymes of
	MJLIPHIC_00124	CDS	COG1062	148387	149478	-	[C] Zn-dependent alcohol dehydrogenases, class III
58							
	MJLIPHIC_00125	CDS	COG0824	149514	149963	+	[R] Predicted thioesterase
	MJLIPHIC_00126	CDS	ROG1353	149981	150424	+	NA
59							
	MJLIPHIC_00127	CDS	COG1802	150431	151123	-	[K] Transcriptional regulators
60							
	MJLIPHIC_00128	CDS	COG0318	151273	152907	+	[IQ] Acyl-CoA synthetases (AMP-forming)/AMP-acid ligases II
61							
	MJLIPHIC_00129	CDS	COG0767	153100	153900	+	[Q] ABC-type transport system involved in resistance
	MJLIPHIC_00130	CDS	COG0767	153902	154771	+	[Q] ABC-type transport system involved in resistance
	MJLIPHIC_00131	CDS	COG1463	154776	156005	+	[Q] ABC-type transport system involved in resistance
	MJLIPHIC_00132	CDS	COG1463	156002	157033	+	[Q] ABC-type transport system involved in resistance
	MJLIPHIC_00133	CDS	COG1463	157030	158604	+	[Q] ABC-type transport system involved in resistance
	MJLIPHIC_00134	CDS	COG1463	158623	160266	+	[Q] ABC-type transport system involved in resistance
	MJLIPHIC_00135	CDS	COG1463	160266	161438	+	[Q] ABC-type transport system involved in resistance
	MJLIPHIC_00136	CDS	COG1463	161440	162996	+	[Q] ABC-type transport system involved in resistance
	MJLIPHIC_00137	CDS	ROG5477	162966	163574	+	NA

	MJLIPHIC_00138	CDS	COG1714	163571	164539	+	[S] Predicted membrane protein/domain
	MJLIPHIC_00139	CDS	ROG5477	164536	165084	+	NA
	MJLIPHIC_00140	CDS	ROG8378	165051	165941	+	NA
	MJLIPHIC_00141	CDS	COG3619	165944	166612	+	[S] Predicted membrane protein
62							
	MJLIPHIC_00142	CDS	ROG2733	166609	168387	-	NA
63							
	MJLIPHIC_00143	CDS	COG3217	168543	169361	-	[R] Uncharacterized Fe-S protein
	MJLIPHIC_00144	CDS	COG3000	169419	170306	-	[I] Sterol desaturase
64							
	MJLIPHIC_00145	CDS	COG1309	170367	171014	-	[K] Transcriptional regulator
65							
	MJLIPHIC_00146	CDS	COG0422	171077	172504	-	[H] Thiamine biosynthesis protein ThiC
	MJLIPHIC_00147	CDS	COG1282	172501	173934	-	[C] NAD/NADP transhydrogenase beta subunit
	MJLIPHIC_00148	CDS	COG3288	173931	174254	-	[C] NAD/NADP transhydrogenase alpha subunit
	MJLIPHIC_00149	CDS	COG3288	174258	175340	-	[C] NAD/NADP transhydrogenase alpha subunit
66							
	MJLIPHIC_00150	CDS	COG1893	175555	176568	+	[H] Ketopantoate reductase
	MJLIPHIC_00151	CDS	COG0469	176579	178012	+	[G] Pyruvate kinase
67							
	MJLIPHIC_00152	CDS	COG2202	178054	178524	-	[T] FOG: PAS/PAC domain
68							
	MJLIPHIC_00153	CDS	COG0583	178631	179557	+	[K] Transcriptional regulator
	MJLIPHIC_00154	CDS	COG0028	179717	181450	+	[EH] Thiamine pyrophosphate-requiring enzymes [acetolactate synthase, pyruvate
	MJLIPHIC_00155	CDS	COG1804	181519	182781	+	[C] Predicted acyl-CoA transferases/carnitine dehydratase
69							
	MJLIPHIC_00156	CDS	COG1905	182884	183369	+	[C] NADH:ubiquinone oxidoreductase 24 kD subunit
	MJLIPHIC_00157	CDS	COG1894	183366	184964	+	[C] NADH:ubiquinone oxidoreductase, NADH-binding (51 kD) subunit
	MJLIPHIC_00158	CDS	COG3383	184961	187780	+	[R] Uncharacterized anaerobic dehydrogenase
	MJLIPHIC_00159	CDS	ROG0161	187782	188006	+	NA
70							
	MJLIPHIC_00160	CDS	ROG3550	188070	188381	-	NA
	MJLIPHIC_00161	CDS	NA	188427	189770	-	NA
71							
	MJLIPHIC_00162	CDS	COG1526	189944	190786	+	[C] Uncharacterized protein required for formate dehydrogenase
72							
	MJLIPHIC_00163	CDS	COG1802	190817	191545	-	[K] Transcriptional regulators
73							
	MJLIPHIC_00164	CDS	COG2211	191746	193149	-	[G] Na+/melibiose symporter and related transporters
74							
	MJLIPHIC_00165	CDS	COG1804	193691	194971	+	[C] Predicted acyl-CoA transferases/carnitine dehydratase
75							
	MJLIPHIC_00166	CDS	COG2211	195185	196567	+	[G] Na+/melibiose symporter and related transporters
76							
	MJLIPHIC_00167	CDS	COG0123	196667	197587	+	[BQ] Deacetylases, including yeast histone deacetylase and
77							
	MJLIPHIC_00168	CDS	ROG7907	197588	198208	-	NA
78							
	MJLIPHIC_00169	CDS	NA	198371	198682	-	NA
	MJLIPHIC_00170	CDS	COG2149	198688	199047	-	[S] Predicted membrane protein
79							
	MJLIPHIC_00171	CDS	COG0665	199157	200293	+	[E] Glycine/D-amino acid oxidases (deaminating)
	MJLIPHIC_00172	CDS	COG0834	200280	201158	+	[ET] ABC-type amino acid transport/signal transduction systems,
	MJLIPHIC_00173	CDS	COG0765	201167	202087	+	[E] ABC-type amino acid transport system, permease
	MJLIPHIC_00174	CDS	COG1126	202084	202866	+	[E] ABC-type polar amino acid transport system,
80							
	MJLIPHIC_00175	CDS	COG1802	202876	203547	-	[K] Transcriptional regulators
	MJLIPHIC_00176	CDS	COG1309	203664	204122	-	[K] Transcriptional regulator
81							
	MJLIPHIC_00177	CDS	COG2175	204225	205163	+	[Q] Probable taurine catabolism dioxygenase
	MJLIPHIC_00178	CDS	COG0596	205190	206347	+	[R] Predicted hydrolases or acyltransferases (alpha/beta hydrolase
	MJLIPHIC_00179	CDS	COG0405	206365	208134	+	[E] Gamma-glutamyltransferase
82							
	MJLIPHIC_00180	CDS	NA	208033	208584	-	NA
	MJLIPHIC_00181	CDS	COG2409	208581	211424	-	[R] Predicted drug exporters of the RND
	MJLIPHIC_00182	CDS	NA	211421	211915	-	NA
83							
	MJLIPHIC_00183	CDS	COG1846	212103	212558	+	[K] Transcriptional regulators
84							
	MJLIPHIC_00184	CDS	COG0454	212548	213006	-	[KR] Histone acetyltransferase HPA2 and related acetyltransferases
	MJLIPHIC_00185	CDS	COG2377	212999	214153	-	[O] Predicted molecular chaperone distantly related to
	MJLIPHIC_00186	CDS	COG0591	214183	215742	-	[ER] Na+/proline symporter
	MJLIPHIC_00187	CDS	COG2971	215751	216731	-	[G] Predicted N-acetylglucosamine kinase
85							
	MJLIPHIC_00188	CDS	COG1737	216912	217814	+	[K] Transcriptional regulators
	MJLIPHIC_00189	CDS	COG2103	217828	218724	+	[R] Predicted sugar phosphate isomerase
86							
	MJLIPHIC_00190	CDS	ROG5614	218725	219423	-	NA
87							
	MJLIPHIC_00191	CDS	COG2072	219598	221238	+	[P] Predicted flavoprotein involved in K+ transport
	MJLIPHIC_00192	CDS	COG2141	221243	222139	+	[C] Coenzyme F420-dependent N5,N10-methylene tetrahydromethanopterin reductase and
88							
	MJLIPHIC_00193	CDS	COG1051	222149	222793	-	[F] ADP-ribose pyrophosphatase
	MJLIPHIC_00194	CDS	NA	222803	224260	-	NA
89							
	MJLIPHIC_00195	CDS	COG3001	224302	224775	+	[G] Fructosamine-3-kinase
90							
	MJLIPHIC_00196	CDS	ROG2252	225160	227712	+	NA
91							
	MJLIPHIC_00197	CDS	COG3464	228361	229272	+	[L] Transposase and inactivated derivatives
92							
	MJLIPHIC_00198	CDS	ROG0016	229280	230032	-	NA
93							
	MJLIPHIC_00199	CDS	NA	230357	232255	+	NA
94							
	MJLIPHIC_00200	CDS	ROG1816	232344	232667	-	NA
95							
	MJLIPHIC_00201	CDS	COG1846	232728	233168	+	[K] Transcriptional regulators
96							
	MJLIPHIC_00202	CDS	COG1835	233161	235296	-	[I] Predicted acyltransferases

97
MJLIPHIC_00203 CDS COG4290 235369 235785 + [F] Guanyl-specific ribonuclease Sa
MJLIPHIC_00204 CDS COG2732 235782 236060 + [K] Barstar, RNAse (barnase) inhibitor
98
MJLIPHIC_00205 CDS COG4632 236061 237158 - [G] Exopolysaccharide biosynthesis protein related to N-acetylglucosamine-1-phosphodiester
99
MJLIPHIC_00206 CDS COG0178 237244 239607 - [L] Excinuclease ATPase subunit
MJLIPHIC_00207 CDS COG2764 239607 240026 - [S] Uncharacterized protein conserved in bacteria
MJLIPHIC_00208 CDS COG2207 240031 240471 - [K] AraC-type DNA-binding domain-containing proteins
MJLIPHIC_00209 CDS COG2133 240488 241771 - [G] Glucose/sorbosone dehydrogenases
MJLIPHIC_00210 CDS COG1741 241749 242465 - [R] Pirin-related protein
100
MJLIPHIC_00211 CDS COG1028 242615 243397 + [IQR] Dehydrogenases with different specificities (related to
101
MJLIPHIC_00212 CDS COG1062 243477 244601 - [C] Zn-dependent alcohol dehydrogenases, class III
102
MJLIPHIC_00213 CDS COG3865 244773 245267 + [S] Uncharacterized protein conserved in bacteria
103
MJLIPHIC_00214 CDS COG1595 245271 246293 - [K] DNA-directed RNA polymerase specialized sigma subunit,
104
MJLIPHIC_00215 CDS COG2267 246357 247199 + [I] Lysophospholipase
MJLIPHIC_00216 CDS COG1073 247196 248173 + [R] Hydrolases of the alpha/beta superfamily
MJLIPHIC_00217 CDS ROG1662 248214 248957 + NA
MJLIPHIC_00218 CDS NA 248954 249436 + NA
MJLIPHIC_00219 CDS COG4122 249456 250112 + [R] Predicted O-methyltransferase
105
MJLIPHIC_00220 CDS COG2409 250120 253017 - [R] Predicted drug exporters of the RND
MJLIPHIC_00221 CDS NA 253014 253442 - NA
106
MJLIPHIC_00222 CDS COG1309 253688 254257 + [K] Transcriptional regulator
107
MJLIPHIC_00223 CDS COG3899 254330 257482 + [R] Predicted ATPase
108
MJLIPHIC_00224 CDS COG0129 257573 259288 - [EG] Dihydroxyacid dehydratase/phosphogluconate dehydratase
109
MJLIPHIC_00225 CDS COG1937 259343 259672 + [S] Uncharacterized protein conserved in bacteria
110
MJLIPHIC_00226 CDS COG0011 259691 259990 - [S] Uncharacterized conserved protein
111
MJLIPHIC_00227 CDS COG2814 260053 261282 + [G] Arabinose efflux permease
112
MJLIPHIC_00228 CDS ROG2219 261440 262480 + NA
113
MJLIPHIC_00229 CDS COG3590 262543 264543 - [O] Predicted metalloendopeptidase
114
MJLIPHIC_00230 CDS ROG5477 264607 265227 + NA
MJLIPHIC_00231 CDS ROG3802 265224 265913 + NA
115
MJLIPHIC_00232 CDS ROG0006 266047 267453 + NA
116
MJLIPHIC_00233 CDS COG1832 267632 268102 - [R] Predicted CoA-binding protein
MJLIPHIC_00234 CDS COG2873 268095 269390 - [E] O-acetylhomoserine sulfhydrylase
117
MJLIPHIC_00235 CDS NA 269466 270080 - NA
MJLIPHIC_00236 CDS COG2409 270077 272938 - [R] Predicted drug exporters of the RND
118
MJLIPHIC_00237 CDS NA 273263 273844 + NA
119
MJLIPHIC_00238 CDS NA 274014 274394 + NA
MJLIPHIC_00239 CDS COG0745 274412 275140 + [TK] Response regulators consisting of a CheY-like
MJLIPHIC_00240 CDS COG0642 275161 276513 + [T] Signal transduction histidine kinase
120
MJLIPHIC_00241 CDS ROG0816 276501 276869 - NA
MJLIPHIC_00242 CDS COG0739 276856 278070 - [M] Membrane proteins related to metalloendopeptidases
MJLIPHIC_00243 CDS COG0392 278070 279161 - [S] Predicted integral membrane protein
121
MJLIPHIC_00244 CDS COG0628 279235 280374 + [R] Predicted permease
122
MJLIPHIC_00245 CDS COG2409 280382 283423 - [R] Predicted drug exporters of the RND
123
MJLIPHIC_00246 CDS ROG2010 283518 284222 - NA
MJLIPHIC_00247 CDS COG0220 284219 285070 - [R] Predicted S-adenosylmethionine-dependent methyltransferase
124
MJLIPHIC_00248 CDS COG0486 285179 286186 + [R] Predicted GTPase
MJLIPHIC_00249 CDS ROG5812 286183 287649 + NA
125
MJLIPHIC_00250 CDS COG1274 287807 289633 + [C] Phosphoenolpyruvate carboxykinase (GTP)
126
MJLIPHIC_00251 CDS ROG3094 289749 290651 + NA
MJLIPHIC_00252 CDS COG0318 290685 292199 + [IQ] Acyl-CoA synthetases (AMP-forming)/AMP-acid ligases II
MJLIPHIC_00253 CDS COG1024 292201 293106 + [I] Enoyl-CoA hydratase/carnithine racemase
127
MJLIPHIC_00254 CDS COG4221 293084 293794 - [R] Short-chain alcohol dehydrogenase of unknown specificity
128
MJLIPHIC_00255 CDS COG1396 293906 294721 + [K] Predicted transcriptional regulators
MJLIPHIC_00256 CDS COG2030 294737 295729 + [I] Acyl dehydratase
MJLIPHIC_00257 CDS COG1804 295766 296872 + [C] Predicted acyl-CoA transferases/carnitine dehydratase
MJLIPHIC_00258 CDS COG1574 296866 298137 + [R] Predicted metal-dependent hydrolase with the TIM-barrel
MJLIPHIC_00259 CDS NA 298158 299276 + NA
MJLIPHIC_00260 CDS COG1982 299314 300771 + [E] Arginine/lysine/ornithine decarboxylases
129
MJLIPHIC_00261 CDS COG1573 300761 301390 - [L] Uracil-DNA glycosylase
130
MJLIPHIC_00262 CDS COG0657 301478 302494 + [I] Esterase/lipase
131
MJLIPHIC_00263 CDS COG2188 302495 303274 - [K] Transcriptional regulators
MJLIPHIC_00264 CDS COG1028 303324 304103 - [IQR] Dehydrogenases with different specificities (related to
MJLIPHIC_00265 CDS COG2334 304140 305144 - [R] Putative homoserine kinase type II (protein
MJLIPHIC_00266 CDS ROG0125 305144 305800 - NA

	MJLIPHIC_00267	CDS	COG4577	305803	306084	-	[QC] Carbon dioxide concentrating mechanism/carboxysome shell protein
	MJLIPHIC_00268	CDS	COG4576	306097	306360	-	[QC] Carbon dioxide concentrating mechanism/carboxysome shell protein
	MJLIPHIC_00269	CDS	NA	306377	306898	-	NA
	MJLIPHIC_00270	CDS	ROG0125	306901	307509	-	NA
	MJLIPHIC_00271	CDS	NA	307518	309101	-	NA
	MJLIPHIC_00272	CDS	COG0160	309098	310378	-	[E] 4-aminobutyrate aminotransferase and related aminotransferases
132							
	MJLIPHIC_00273	CDS	COG0531	310563	312098	+	[E] Amino acid transporters
	MJLIPHIC_00274	CDS	NA	312116	312412	+	NA
133							
	MJLIPHIC_00275	CDS	COG0657	312359	313258	-	[I] Esterase/lipase
134							
	MJLIPHIC_00276	CDS	COG2303	313349	314920	+	[E] Choline dehydrogenase and related flavoproteins
	MJLIPHIC_00277	CDS	ROG0920	314913	315233	+	NA
135							
	MJLIPHIC_00278	CDS	COG1028	315285	316055	+	[IQR] Dehydrogenases with different specificities (related to
136							
	MJLIPHIC_00279	CDS	COG2249	316060	316854	-	[R] Putative NADPH-quinone reductase (modulator of drug
	MJLIPHIC_00280	CDS	COG1309	316851	317483	-	[K] Transcriptional regulator
	MJLIPHIC_00281	CDS	COG2188	317634	318320	-	[K] Transcriptional regulators
	MJLIPHIC_00282	CDS	COG4341	318317	318844	-	[R] Predicted HD phosphohydrolase
137							
	MJLIPHIC_00283	CDS	COG0665	318954	320069	+	[E] Glycine/D-amino acid oxidases (deaminating)
	MJLIPHIC_00284	CDS	COG1073	320147	321361	+	[R] Hydrolases of the alpha/beta superfamily
	MJLIPHIC_00285	CDS	ROG1697	321386	322804	+	NA
138							
	MJLIPHIC_00286	CDS	COG2175	322807	323652	-	[Q] Probable taurine catabolism dioxygenase
	MJLIPHIC_00287	CDS	NA	323681	324010	-	NA
	MJLIPHIC_00288	CDS	COG4638	324012	325277	-	[PR] Phenylpropionate dioxygenase and related ring-hydroxylating dioxygenases,
	MJLIPHIC_00289	CDS	COG1028	325317	326060	-	[IQR] Dehydrogenases with different specificities (related to
	MJLIPHIC_00290	CDS	COG1018	326057	327124	-	[C] Flavodoxin reductases (ferredoxin-NADPH reductases) family 1
139							
	MJLIPHIC_00291	CDS	COG1846	327192	327647	-	[K] Transcriptional regulators
	MJLIPHIC_00292	CDS	COG1228	327678	328937	-	[Q] Imidazolonepropionase and related amidohydrolases
	MJLIPHIC_00293	CDS	NA	328934	329275	-	NA
	MJLIPHIC_00294	CDS	COG4638	329279	330544	-	[PR] Phenylpropionate dioxygenase and related ring-hydroxylating dioxygenases,
140							
	MJLIPHIC_00295	CDS	COG2159	330792	331799	+	[R] Predicted metal-dependent hydrolase of the TIM-barrel
141							
	MJLIPHIC_00296	CDS	COG0277	331803	333191	-	[C] FAD/FMN-containing dehydrogenases
142							
	MJLIPHIC_00297	CDS	COG0604	333272	334264	+	[CR] NADPH:quinone reductase and related Zn-dependent oxidoreductases
143							
	MJLIPHIC_00298	CDS	COG2272	334261	335472	-	[I] Carboxylesterase type B
	MJLIPHIC_00299	CDS	COG0318	335465	337096	-	[IQ] Acyl-CoA synthetases (AMP-forming)/AMP-acid ligases II
	MJLIPHIC_00300	CDS	COG0204	337102	337911	-	[I] 1-acyl-sn-glycerol-3-phosphate acyltransferase
144							
	MJLIPHIC_00301	CDS	COG2162	337990	338826	+	[Q] Arylamine N-acetyltransferase
	MJLIPHIC_00302	CDS	COG2207	338885	339805	+	[K] AraC-type DNA-binding domain-containing proteins
	MJLIPHIC_00303	CDS	COG1985	339809	340336	+	[H] Pyrimidine reductase, riboflavin biosynthesis
145							
	MJLIPHIC_00304	CDS	COG1012	340411	341895	-	[C] NAD-dependent aldehyde dehydrogenases
	MJLIPHIC_00305	CDS	COG0500	341951	342403	-	[QR] SAM-dependent methyltransferases
146							
	MJLIPHIC_00306	CDS	COG0438	342778	343923	+	[M] Glycosyltransferase
147							
	MJLIPHIC_00307	CDS	COG0800	343930	344559	-	[G] 2-keto-3-deoxy-6-phosphogluconate aldolase
	MJLIPHIC_00308	CDS	COG0129	344556	346385	-	[EG] Dihydroxyacid dehydratase/phosphogluconate dehydratase
	MJLIPHIC_00309	CDS	COG0364	346382	347869	-	[G] Glucose-6-phosphate 1-dehydrogenase
148							
	MJLIPHIC_00310	CDS	COG1940	348069	349247	+	[KG] Transcriptional regulator/sugar kinase
149							
	MJLIPHIC_00311	CDS	ROG1245	349207	350844	-	NA
	MJLIPHIC_00312	CDS	ROG1501	350874	352070	-	NA
150							
	MJLIPHIC_00313	CDS	COG1835	352170	353429	+	[I] Predicted acyltransferases
151							
	MJLIPHIC_00314	CDS	COG0318	353392	354879	-	[IQ] Acyl-CoA synthetases (AMP-forming)/AMP-acid ligases II
	MJLIPHIC_00315	CDS	COG1735	354881	355858	-	[R] Predicted metal-dependent hydrolase with the TIM-barrel
152							
	MJLIPHIC_00316	CDS	COG2771	355916	358708	-	[K] DNA-binding HTH domain-containing proteins
153							
	MJLIPHIC_00317	CDS	COG1960	359235	360470	-	[I] Acyl-CoA dehydrogenases
154							
	MJLIPHIC_00318	CDS	ROG4360	360723	361178	+	NA
	MJLIPHIC_00319	CDS	ROG5266	361191	361703	+	NA
	MJLIPHIC_00320	CDS	COG0318	361710	363317	+	[IQ] Acyl-CoA synthetases (AMP-forming)/AMP-acid ligases II
	MJLIPHIC_00321	CDS	COG1012	363333	364835	+	[C] NAD-dependent aldehyde dehydrogenases
	MJLIPHIC_00322	CDS	NA	364857	365306	+	NA
	MJLIPHIC_00323	CDS	COG1853	365306	365797	+	[R] Conserved protein/domain typically associated with flavoprotein
155							
	MJLIPHIC_00324	CDS	COG2197	365798	366985	-	[TK] Response regulator containing a CheY-like receiver
	MJLIPHIC_00325	CDS	COG2197	366982	368031	-	[TK] Response regulator containing a CheY-like receiver
156							
	MJLIPHIC_00326	CDS	COG2070	368223	369158	+	[R] Dioxygenases related to 2-nitropropane dioxygenase
	MJLIPHIC_00327	CDS	COG4799	369275	370873	+	[I] Acetyl-CoA carboxylase, carboxyltransferase component (subunits alpha
	MJLIPHIC_00328	CDS	COG4770	370879	372888	+	[I] Acetyl/propionyl-CoA carboxylase, alpha subunit
	MJLIPHIC_00329	CDS	COG1024	372885	373643	+	[I] Enoyl-CoA hydratase/carnithine racemase
	MJLIPHIC_00330	CDS	ROG1303	373643	374437	+	NA
	MJLIPHIC_00331	CDS	ROG0003	374434	376188	+	NA
	MJLIPHIC_00332	CDS	COG1960	376204	377340	+	[I] Acyl-CoA dehydrogenases
	MJLIPHIC_00333	CDS	NA	377365	378393	+	NA
	MJLIPHIC_00334	CDS	COG1024	378402	379154	+	[I] Enoyl-CoA hydratase/carnithine racemase
157							
	MJLIPHIC_00335	CDS	NA	379158	380204	-	NA
	MJLIPHIC_00336	CDS	NA	380208	381053	-	NA
158							
	MJLIPHIC_00337	CDS	COG1309	381177	381809	-	[K] Transcriptional regulator
159							
	MJLIPHIC_00338	CDS	COG0767	381974	382837	+	[Q] ABC-type transport system involved in resistance

	MJLIPHIC_00339	CDS	COG0767	382881	383699	+	[Q] ABC-type transport system involved in resistance
	MJLIPHIC_00340	CDS	COG1463	383703	385127	+	[Q] ABC-type transport system involved in resistance
	MJLIPHIC_00341	CDS	COG1463	385157	386185	+	[Q] ABC-type transport system involved in resistance
	MJLIPHIC_00342	CDS	COG1463	386182	387498	+	[Q] ABC-type transport system involved in resistance
	MJLIPHIC_00343	CDS	COG1463	387495	388796	+	[Q] ABC-type transport system involved in resistance
	MJLIPHIC_00344	CDS	COG1463	388793	389932	+	[Q] ABC-type transport system involved in resistance
	MJLIPHIC_00345	CDS	COG1463	389934	391421	+	[Q] ABC-type transport system involved in resistance
	MJLIPHIC_00346	CDS	ROG5477	391385	391987	+	NA
	MJLIPHIC_00347	CDS	ROG3802	391984	392592	+	NA
	MJLIPHIC_00348	CDS	NA	392616	392957	+	NA
	MJLIPHIC_00349	CDS	COG2340	392954	393448	+	[S] Uncharacterized protein with SCP/PR1 domains
160							
	MJLIPHIC_00350	CDS	COG2388	393601	393915	-	[R] Predicted acetyltransferase
161							
	MJLIPHIC_00351	CDS	NA	394013	395614	+	NA
	MJLIPHIC_00352	CDS	COG0208	395654	396616	+	[F] Ribonucleotide reductase, beta subunit
162							
	MJLIPHIC_00353	CDS	ROG1147	396891	401135	-	NA
	MJLIPHIC_00354	CDS	NA	401170	401343	-	NA
163							
	MJLIPHIC_00355	CDS	COG1472	401448	402611	+	[G] Beta-glucosidase-related glycosidases
164							
	MJLIPHIC_00356	CDS	COG2159	402623	403786	-	[R] Predicted metal-dependent hydrolase of the TIM-barrel
165							
	MJLIPHIC_00357	CDS	COG1309	403918	404502	+	[K] Transcriptional regulator
166							
	MJLIPHIC_00358	CDS	NA	404569	404868	+	NA
	MJLIPHIC_00359	CDS	ROG1748	404871	406751	+	NA
	MJLIPHIC_00360	CDS	NA	406748	407275	+	NA
167							
	MJLIPHIC_00361	CDS	COG0557	407282	408370	-	[K] Exoribonuclease R
168							
	MJLIPHIC_00362	CDS	ROG0431	408516	409982	+	NA
	MJLIPHIC_00363	CDS	ROG1817	409979	410701	+	NA
	MJLIPHIC_00364	CDS	COG4913	410691	414047	+	[S] Uncharacterized protein conserved in bacteria
169							
	MJLIPHIC_00365	CDS	ROG0252	414063	414920	-	NA
	MJLIPHIC_00366	CDS	COG1028	414922	416274	-	[IQR] Dehydrogenases with different specificities (related to
170							
	MJLIPHIC_00367	CDS	COG0183	416355	417653	+	[I] Acetyl-CoA acetyltransferase
171							
	MJLIPHIC_00368	CDS	COG0277	418197	420065	-	[C] FAD/FMN-containing dehydrogenases
172							
	MJLIPHIC_00369	CDS	COG1502	420213	421802	-	[I] Phosphatidylserine/phosphatidylglycerophosphate/cardiolipin synthases and related enzymes
173							
	MJLIPHIC_00370	CDS	COG4977	421906	422928	+	[K] Transcriptional regulator containing an amidase domain
174							
	MJLIPHIC_00371	CDS	ROG0365	422996	423736	+	NA
	MJLIPHIC_00372	CDS	ROG0342	423733	424353	+	NA
	MJLIPHIC_00373	CDS	ROG1122	424350	424631	+	NA
175							
	MJLIPHIC_00374	CDS	NA	425344	425763	+	NA
	MJLIPHIC_00375	CDS	COG2409	425760	428642	+	[R] Predicted drug exporters of the RND
	MJLIPHIC_00376	CDS	COG2409	428652	431636	+	[R] Predicted drug exporters of the RND
176							
	MJLIPHIC_00377	CDS	NA	431707	432045	-	NA
177							
	MJLIPHIC_00378	CDS	COG1209	432229	433095	-	[M] dTDP-glucose pyrophosphorylase
178							
	MJLIPHIC_00379	CDS	COG1819	433692	434870	+	[GC] Glycosyl transferases, related to UDP-glucuronosyltransferase
	MJLIPHIC_00380	CDS	COG0451	434928	436061	+	[MG] Nucleoside-diphosphate-sugar epimerases
179							
	MJLIPHIC_00381	CDS	ROG7051	436232	437044	+	NA
180							
	MJLIPHIC_00382	CDS	ROG0132	437374	438096	+	NA
	MJLIPHIC_00383	CDS	COG1819	438130	439398	+	[GC] Glycosyl transferases, related to UDP-glucuronosyltransferase
181							
	MJLIPHIC_00384	CDS	NA	439518	440687	+	NA
182							
	MJLIPHIC_00385	CDS	ROG0132	440849	441649	+	NA
183							
	MJLIPHIC_00386	CDS	COG1819	441687	442940	-	[GC] Glycosyl transferases, related to UDP-glucuronosyltransferase
184							
	MJLIPHIC_00387	CDS	COG0500	443143	443967	+	[QR] SAM-dependent methyltransferases
185							
	MJLIPHIC_00388	CDS	NA	444075	444329	+	NA
186							
	MJLIPHIC_00389	CDS	COG3464	445715	446626	+	[L] Transposase and inactivated derivatives
187							
	MJLIPHIC_00390	CDS	ROG0016	446634	447386	-	NA
188							
	MJLIPHIC_00391	CDS	COG3251	447638	447868	+	[S] Uncharacterized protein conserved in bacteria
	MJLIPHIC_00392	CDS	COG1020	447897	455186	+	[Q] Non-ribosomal peptide synthetase modules and related
	MJLIPHIC_00393	CDS	COG1020	455162	458191	+	[Q] Non-ribosomal peptide synthetase modules and related
	MJLIPHIC_00394	CDS	COG1020	458188	465867	+	[Q] Non-ribosomal peptide synthetase modules and related
	MJLIPHIC_00395	CDS	ROG2658	465884	466702	+	NA
189							
	MJLIPHIC_00396	CDS	COG3795	466804	467214	+	[S] Uncharacterized protein conserved in bacteria
	MJLIPHIC_00397	CDS	COG4941	467223	468398	+	[K] Predicted RNA polymerase sigma factor containing
190							
	MJLIPHIC_00398	CDS	COG1960	468441	470276	-	[I] Acyl-CoA dehydrogenases
191							
	MJLIPHIC_00399	CDS	COG3804	470547	471602	+	[S] Uncharacterized conserved protein related to dihydrodipicolinate
192							
	MJLIPHIC_00400	CDS	COG3321	471613	482571	-	[Q] Polyketide synthase modules and related proteins
193							
	MJLIPHIC_00401	CDS	NA	482621	484234	+	NA
	MJLIPHIC_00402	CDS	COG2409	484268	487276	+	[R] Predicted drug exporters of the RND
194							

	MJLIPHIC_00403	CDS	COG0318	487386	489095	+	[IQ] Acyl-CoA synthetases (AMP-forming)/AMP-acid ligases II
	MJLIPHIC_00404	CDS	NA	489157	490308	+	NA
195							
	MJLIPHIC_00405	CDS	ROG2658	490313	490990	-	NA
196							
	MJLIPHIC_00406	CDS	COG3491	491026	492051	+	[R] Isopenicillin N synthase and related dioxygenases
	MJLIPHIC_00407	CDS	COG1853	492065	492550	+	[R] Conserved protein/domain typically associated with flavoprotein
197							
	MJLIPHIC_00408	CDS	NA	492595	492837	-	NA
	MJLIPHIC_00409	CDS	COG0479	492834	493583	-	[C] Succinate dehydrogenase/fumarate reductase, Fe-S protein subunit
	MJLIPHIC_00410	CDS	COG1053	493585	495492	-	[C] Succinate dehydrogenase/fumarate reductase, flavoprotein subunit
198							
	MJLIPHIC_00411	CDS	ROG1092	495575	496396	-	NA
	MJLIPHIC_00412	CDS	ROG3550	496449	496745	-	NA
199							
	MJLIPHIC_00413	CDS	COG2513	497010	497777	+	[G] PEP phosphonomutase and related enzymes
200							
	MJLIPHIC_00414	CDS	NA	497860	498360	+	NA
201							
	MJLIPHIC_00415	CDS	COG0071	498438	498875	-	[O] Molecular chaperone (small heat shock protein)
	MJLIPHIC_00416	CDS	COG0697	498953	499888	-	[GER] Permeases of the drug/metabolite transporter (DMT)
202							
	MJLIPHIC_00417	CDS	COG1167	499923	501296	+	[KE] Transcriptional regulators containing a DNA-binding HTH
203							
	MJLIPHIC_00418	CDS	COG1251	501440	504019	+	[C] NAD(P)H-nitrite reductase
	MJLIPHIC_00419	CDS	COG2146	504016	504384	+	[PR] Ferredoxin subunits of nitrite reductase and
204							
	MJLIPHIC_00420	CDS	NA	504399	504599	-	NA
205							
	MJLIPHIC_00421	CDS	COG3464	505070	506392	+	[L] Transposase and inactivated derivatives
206							
	MJLIPHIC_00422	CDS	COG2138	506807	507502	-	[S] Uncharacterized conserved protein
	MJLIPHIC_00423	CDS	COG1587	507499	508656	-	[H] Uroporphyrinogen-III synthase
	MJLIPHIC_00424	CDS	COG2223	508691	510100	-	[P] Nitrate/nitrite transporter
207							
	MJLIPHIC_00425	CDS	COG0454	510209	510841	-	[KR] Histone acetyltransferase HPA2 and related acetyltransferases
	MJLIPHIC_00426	CDS	COG1984	510873	511757	-	[E] Allophanate hydrolase subunit 2
	MJLIPHIC_00427	CDS	COG2049	511773	512435	-	[E] Allophanate hydrolase subunit 1
208							
	MJLIPHIC_00428	CDS	COG1738	512510	513190	-	[S] Uncharacterized conserved protein
	MJLIPHIC_00429	CDS	COG0614	513215	514132	-	[P] ABC-type Fe3+-hydroxamate transport system, periplasmic component
	MJLIPHIC_00430	CDS	ROG1878	514184	515320	-	NA
209							
	MJLIPHIC_00431	CDS	ROG2730	515471	516199	-	NA
	MJLIPHIC_00432	CDS	ROG6516	516209	516811	-	NA
210							
	MJLIPHIC_00433	CDS	COG0388	516901	517893	+	[R] Predicted amidohydrolase
	MJLIPHIC_00434	CDS	COG2957	517901	518923	+	[E] Peptidylarginine deiminase and related enzymes
	MJLIPHIC_00435	CDS	COG1574	518920	520557	+	[R] Predicted metal-dependent hydrolase with the TIM-barrel
	MJLIPHIC_00436	CDS	COG0531	520569	521912	+	[E] Amino acid transporters
211							
	MJLIPHIC_00437	CDS	COG1764	521987	522406	-	[O] Predicted redox protein, regulator of disulfide
212							
	MJLIPHIC_00438	CDS	COG1846	522533	522976	+	[K] Transcriptional regulators
213							
	MJLIPHIC_00439	CDS	COG0589	522988	524988	-	[T] Universal stress protein UspA and related
214							
	MJLIPHIC_00440	CDS	NA	525323	525571	-	NA
	MJLIPHIC_00441	CDS	COG2610	525610	527055	-	[GE] H+/gluconate symporter and related permeases
	MJLIPHIC_00442	CDS	COG3265	527092	527604	-	[G] Gluconate kinase
215							
	MJLIPHIC_00443	tRNA	NA	528038	528127	-	NA
216							
	MJLIPHIC_00444	CDS	COG1012	528536	529963	+	[C] NAD-dependent aldehyde dehydrogenases
217							
	MJLIPHIC_00445	CDS	COG0188	529995	532136	-	[L] Type IIA topoisomerase (DNA gyrase/topo II,
	MJLIPHIC_00446	CDS	COG0187	532147	534180	-	[L] Type IIA topoisomerase (DNA gyrase/topo II,
218							
	MJLIPHIC_00447	CDS	COG4585	534383	535687	+	[T] Signal transduction histidine kinase
	MJLIPHIC_00448	CDS	COG2197	535697	536341	+	[TK] Response regulator containing a CheY-like receiver
219							
	MJLIPHIC_00449	CDS	COG0596	536353	537297	-	[R] Predicted hydrolases or acyltransferases (alpha/beta hydrolase
220							
	MJLIPHIC_00450	CDS	COG1739	537351	537965	+	[S] Uncharacterized conserved protein
	MJLIPHIC_00451	CDS	NA	537928	538545	+	NA
	MJLIPHIC_00452	CDS	COG2409	538542	541427	+	[R] Predicted drug exporters of the RND
	MJLIPHIC_00453	CDS	COG1309	541484	542098	+	[K] Transcriptional regulator
221							
	MJLIPHIC_00454	CDS	COG0351	542092	542898	-	[H] Hydroxymethylpyrimidine/phosphomethylpyrimidine kinase
222							
	MJLIPHIC_00455	CDS	NA	543024	543872	+	NA
223							
	MJLIPHIC_00456	CDS	COG1279	543882	544481	-	[R] Lysine efflux permease
224							
	MJLIPHIC_00457	CDS	COG0454	544595	545467	+	[KR] Histone acetyltransferase HPA2 and related acetyltransferases
225							
	MJLIPHIC_00458	CDS	COG5006	545434	546279	-	[R] Predicted permease, DMT superfamily
226							
	MJLIPHIC_00459	CDS	COG0583	546405	547280	+	[K] Transcriptional regulator
227							
	MJLIPHIC_00460	CDS	COG2272	547268	548872	-	[I] Carboxylesterase type B
228							
	MJLIPHIC_00461	CDS	NA	548983	549786	-	NA
229							
	MJLIPHIC_00462	CDS	COG2197	550356	551168	+	[TK] Response regulator containing a CheY-like receiver
230							
	MJLIPHIC_00463	CDS	COG0449	551334	553121	+	[M] Glucosamine 6-phosphate synthetase, contains amidotransferase and
	MJLIPHIC_00464	CDS	COG0677	553181	554419	+	[M] UDP-N-acetyl-D-mannosaminuronate dehydrogenase
	MJLIPHIC_00465	CDS	COG1215	554422	555717	+	[M] Glycosyltransferases, probably involved in cell wall
	MJLIPHIC_00466	CDS	NA	555747	557249	+	NA

MJLIPHIC_00467 CDS NA 557258 557959 + NA
231
MJLIPHIC_00468 CDS COG1802 557995 558654 - [K] Transcriptional regulators
232
MJLIPHIC_00469 CDS COG0644 558749 560095 + [C] Dehydrogenases (flavoproteins)
MJLIPHIC_00470 CDS COG0129 560092 561786 + [EG] Dihydroxyacid dehydratase/phosphogluconate dehydratase
MJLIPHIC_00471 CDS COG0477 561813 563186 + [GEPR] Permeases of the major facilitator superfamily
233
MJLIPHIC_00472 CDS COG2421 563191 564300 - [C] Predicted acetamidase/formamidase
MJLIPHIC_00473 CDS COG0154 564297 565826 - [J] Asp-tRNAAsn/Glu-tRNAGln amidotransferase A subunit and related
MJLIPHIC_00474 CDS COG0715 565863 566894 - [P] ABC-type nitrate/sulfonate/bicarbonate transport systems, periplasmic
components
MJLIPHIC_00475 CDS COG0600 566907 567740 - [P] ABC-type nitrate/sulfonate/bicarbonate transport system, permease component
MJLIPHIC_00476 CDS COG1116 567784 568701 - [P] ABC-type nitrate/sulfonate/bicarbonate transport system, ATPase component
234
MJLIPHIC_00477 CDS COG1804 568865 570037 + [C] Predicted acyl-CoA transferases/carnitine dehydratase
MJLIPHIC_00478 CDS COG1024 570034 570816 + [I] Enoyl-CoA hydratase/carnithine racemase
235
MJLIPHIC_00479 CDS COG1609 570847 571788 - [K] Transcriptional regulators
236
MJLIPHIC_00480 CDS COG4330 571918 572553 - [S] Predicted membrane protein
237
MJLIPHIC_00481 CDS COG3187 572636 572803 - [O] Heat shock protein
MJLIPHIC_00482 CDS COG3187 573002 573169 - [O] Heat shock protein
238
MJLIPHIC_00483 CDS ROG4508 573276 574046 - NA
MJLIPHIC_00484 CDS ROG4508 574037 574849 - NA
MJLIPHIC_00485 CDS COG4910 574846 575205 - [Q] Propanediol dehydratase, small subunit
MJLIPHIC_00486 CDS COG4909 575202 577466 - [Q] Propanediol dehydratase, large subunit
239
MJLIPHIC_00487 CDS COG0531 577723 579150 + [E] Amino acid transporters
240
MJLIPHIC_00488 CDS COG2508 579284 580351 + [TQ] Regulator of polyketide synthase expression
241
MJLIPHIC_00489 CDS COG0363 580361 581107 - [G] 6-phosphogluconolactonase/Glucosamine-6-phosphate isomerase/deaminase
MJLIPHIC_00490 CDS COG1486 581104 582480 - [G] Alpha-galactosidases/6-phospho-beta-glucosidases, family 4 of glycosyl
hydrolases
MJLIPHIC_00491 CDS COG1349 582477 583370 - [KG] Transcriptional regulators of sugar metabolism
242
MJLIPHIC_00492 CDS COG0524 583427 584413 + [G] Sugar kinases, ribokinase family
MJLIPHIC_00493 CDS COG1653 584410 585780 + [G] ABC-type sugar transport system, periplasmic component
MJLIPHIC_00494 CDS COG1175 585777 586739 + [G] ABC-type sugar transport systems, permease components
MJLIPHIC_00495 CDS COG0395 586736 587560 + [G] ABC-type sugar transport system, permease component
MJLIPHIC_00496 CDS COG3839 587587 588657 + [G] ABC-type sugar transport systems, ATPase components
243
MJLIPHIC_00497 CDS COG1349 588742 589527 - [KG] Transcriptional regulators of sugar metabolism
244
MJLIPHIC_00498 CDS COG4573 589666 590988 + [G] Predicted tagatose 6-phosphate kinase
MJLIPHIC_00499 CDS COG2222 590985 592127 + [M] Predicted phosphosugar isomerases
MJLIPHIC_00500 CDS COG2971 592133 593104 + [G] Predicted N-acetylglucosamine kinase
MJLIPHIC_00501 CDS COG2510 593126 594091 + [S] Predicted membrane protein
MJLIPHIC_00502 CDS COG1486 594088 595401 + [G] Alpha-galactosidases/6-phospho-beta-glucosidases, family 4 of glycosyl
hydrolases
MJLIPHIC_00503 CDS COG1653 595444 596721 + [G] ABC-type sugar transport system, periplasmic component
MJLIPHIC_00504 CDS COG1175 596736 597647 + [G] ABC-type sugar transport systems, permease components
MJLIPHIC_00505 CDS COG0395 597644 598570 + [G] ABC-type sugar transport system, permease component
MJLIPHIC_00506 CDS COG3839 598612 599718 + [G] ABC-type sugar transport systems, ATPase components
245
MJLIPHIC_00507 CDS NA 599812 599922 + NA
246
MJLIPHIC_00508 CDS NA 600086 600733 + NA
247
MJLIPHIC_00509 CDS ROG2998 600745 601362 - NA
MJLIPHIC_00510 CDS ROG2998 601359 601814 - NA
248
MJLIPHIC_00511 CDS NA 601925 602077 + NA
249
MJLIPHIC_00512 CDS ROG2481 602113 603039 - NA
MJLIPHIC_00513 CDS COG4221 603036 603863 - [R] Short-chain alcohol dehydrogenase of unknown specificity
250
MJLIPHIC_00514 CDS COG2303 604076 605674 + [E] Choline dehydrogenase and related flavoproteins
MJLIPHIC_00515 CDS COG1028 605757 606500 + [IQR] Dehydrogenases with different specificities (related to
MJLIPHIC_00516 CDS NA 606524 607450 + NA
251
MJLIPHIC_00517 CDS COG2909 607494 610940 - [K] ATP-dependent transcriptional regulator
252
MJLIPHIC_00518 CDS COG1028 611055 611756 - [IQR] Dehydrogenases with different specificities (related to
MJLIPHIC_00519 CDS COG1960 611979 613190 - [I] Acyl-CoA dehydrogenases
MJLIPHIC_00520 CDS COG1309 613219 613836 - [K] Transcriptional regulator
253
MJLIPHIC_00521 CDS ROG5315 613901 614440 - NA
254
MJLIPHIC_00522 CDS COG0477 614551 615804 - [GEPR] Permeases of the major facilitator superfamily
MJLIPHIC_00523 CDS COG1802 615801 616439 - [K] Transcriptional regulators
MJLIPHIC_00524 CDS COG0693 616498 617064 - [R] Putative intracellular protease/amidase
255
MJLIPHIC_00525 CDS ROG3373 617136 617564 - NA
MJLIPHIC_00526 CDS COG1846 617579 618070 - [K] Transcriptional regulators
MJLIPHIC_00527 CDS COG0664 618144 618818 - [T] cAMP-binding proteins - catabolite gene activator
256
MJLIPHIC_00528 CDS COG2808 618988 619611 + [K] Transcriptional regulator
257
MJLIPHIC_00529 CDS NA 619744 620037 + NA
MJLIPHIC_00530 CDS COG2202 620034 620738 + [T] FOG: PAS/PAC domain
258
MJLIPHIC_00531 CDS COG0583 620827 621762 + [K] Transcriptional regulator
259
MJLIPHIC_00532 CDS ROG7022 621750 622790 - NA
260
MJLIPHIC_00533 CDS NA 622986 623513 - NA

261							
	MJLIPHIC_00534	CDS	COG0500	623786	624613	-	[QR] SAM-dependent methyltransferases
262							
	MJLIPHIC_00535	CDS	COG0583	624722	625600	+	[K] Transcriptional regulator
263							
	MJLIPHIC_00536	CDS	COG3335	625597	626658	-	[L] Transposase and inactivated derivatives
	MJLIPHIC_00537	CDS	COG0600	626702	627430	-	[P] ABC-type nitrate/sulfonate/bicarbonate transport system, permease component
	MJLIPHIC_00538	CDS	COG0715	627472	628440	-	[P] ABC-type nitrate/sulfonate/bicarbonate transport systems, periplasmic components
	MJLIPHIC_00539	CDS	COG1116	628489	629235	-	[P] ABC-type nitrate/sulfonate/bicarbonate transport system, ATPase component
264							
	MJLIPHIC_00540	CDS	ROG2667	629360	629854	+	NA
265							
	MJLIPHIC_00541	CDS	COG1653	629988	631274	+	[G] ABC-type sugar transport system, periplasmic component
	MJLIPHIC_00542	CDS	COG1175	631271	632140	+	[G] ABC-type sugar transport systems, permease components
	MJLIPHIC_00543	CDS	COG0395	632137	632928	+	[G] ABC-type sugar transport system, permease component
	MJLIPHIC_00544	CDS	COG3839	632925	633980	+	[G] ABC-type sugar transport systems, ATPase components
266							
	MJLIPHIC_00545	CDS	NA	634165	634302	+	NA
	MJLIPHIC_00546	CDS	NA	634299	634826	+	NA
267							
	MJLIPHIC_00547	CDS	COG1278	634899	635102	-	[K] Cold shock proteins
268							
	MJLIPHIC_00548	CDS	COG3804	635220	636284	-	[S] Uncharacterized conserved protein related to dihydrodipicolinate
269							
	MJLIPHIC_00549	CDS	ROG6516	636538	637101	+	NA
	MJLIPHIC_00550	CDS	NA	637149	637904	+	NA
270							
	MJLIPHIC_00551	CDS	COG2354	637893	638879	-	[S] Uncharacterized protein conserved in bacteria
	MJLIPHIC_00552	CDS	COG2252	638914	640326	-	[R] Permeases
	MJLIPHIC_00553	CDS	COG0438	640338	641435	-	[M] Glycosyltransferase
	MJLIPHIC_00554	CDS	COG0388	641432	642280	-	[R] Predicted amidohydrolase
	MJLIPHIC_00555	CDS	COG2144	642290	643711	-	[R] Selenophosphate synthetase-related proteins
	MJLIPHIC_00556	CDS	COG2516	643711	644766	-	[R] Biotin synthase-related enzyme
	MJLIPHIC_00557	CDS	COG3486	644774	646045	-	[Q] Lysine/ornithine N-monooxygenase
	MJLIPHIC_00558	CDS	ROG2130	646042	646338	-	NA
	MJLIPHIC_00559	CDS	COG0388	646331	647233	-	[R] Predicted amidohydrolase
	MJLIPHIC_00560	CDS	ROG1893	647243	647764	-	NA
271							
	MJLIPHIC_00561	CDS	COG1595	648122	648733	+	[K] DNA-directed RNA polymerase specialized sigma subunit,
272							
	MJLIPHIC_00562	CDS	COG1595	648700	649296	-	[K] DNA-directed RNA polymerase specialized sigma subunit,
273							
	MJLIPHIC_00563	CDS	ROG3038	649538	649978	+	NA
	MJLIPHIC_00564	CDS	COG2409	649975	652866	+	[R] Predicted drug exporters of the RND
	MJLIPHIC_00565	CDS	NA	652914	654131	+	NA
	MJLIPHIC_00566	CDS	COG1960	654135	655271	+	[I] Acyl-CoA dehydrogenases
	MJLIPHIC_00567	CDS	ROG1797	655294	656088	+	NA
274							
	MJLIPHIC_00568	CDS	COG2508	656078	657565	-	[TQ] Regulator of polyketide synthase expression
275							
	MJLIPHIC_00569	CDS	COG0160	657676	659022	+	[E] 4-aminobutyrate aminotransferase and related aminotransferases
	MJLIPHIC_00570	CDS	COG1012	659025	660479	+	[C] NAD-dependent aldehyde dehydrogenases
276							
	MJLIPHIC_00571	CDS	COG2149	660492	660752	-	[S] Predicted membrane protein
277							
	MJLIPHIC_00572	CDS	COG1804	661023	662237	+	[C] Predicted acyl-CoA transferases/carnitine dehydratase
278							
	MJLIPHIC_00573	CDS	NA	662508	662957	-	NA
279							
	MJLIPHIC_00574	CDS	COG3254	663229	663549	+	[S] Uncharacterized conserved protein
	MJLIPHIC_00575	CDS	COG2814	663590	664921	+	[G] Arabinose efflux permease
	MJLIPHIC_00576	CDS	COG4952	664918	666081	+	[M] Predicted sugar isomerase
	MJLIPHIC_00577	CDS	COG3347	666084	668123	+	[S] Uncharacterized conserved protein
	MJLIPHIC_00578	CDS	COG1070	668184	669641	+	[G] Sugar (pentulose and hexulose) kinases
	MJLIPHIC_00579	CDS	COG1609	669658	670650	+	[K] Transcriptional regulators
280							
	MJLIPHIC_00580	CDS	COG1556	670737	671351	-	[S] Uncharacterized conserved protein
	MJLIPHIC_00581	CDS	COG1139	671348	672817	-	[C] Uncharacterized conserved protein containing a ferredoxin-like
	MJLIPHIC_00582	CDS	COG0247	672814	673551	-	[C] Fe-S oxidoreductase
281							
	MJLIPHIC_00583	CDS	COG2186	673641	674327	+	[K] Transcriptional regulators
	MJLIPHIC_00584	CDS	NA	674388	674783	+	NA
282							
	MJLIPHIC_00585	CDS	COG3285	674780	676021	-	[L] Predicted eukaryotic-type DNA primase
283							
	MJLIPHIC_00586	CDS	COG0318	676050	677750	+	[IQ] Acyl-CoA synthetases (AMP-forming)/AMP-acid ligases II
	MJLIPHIC_00587	CDS	COG1028	677821	678603	+	[IQR] Dehydrogenases with different specificities (related to
284							
	MJLIPHIC_00588	CDS	NA	678620	678922	-	NA
	MJLIPHIC_00589	CDS	NA	678969	679247	-	NA
285							
	MJLIPHIC_00590	CDS	COG1960	679378	681582	-	[I] Acyl-CoA dehydrogenases
	MJLIPHIC_00591	CDS	COG1052	681693	682676	-	[CHR] Lactate dehydrogenase and related dehydrogenases
	MJLIPHIC_00592	CDS	ROG1833	682652	683794	-	NA
	MJLIPHIC_00593	CDS	COG1309	683791	684420	-	[K] Transcriptional regulator
286							
	MJLIPHIC_00594	CDS	COG0346	684535	685134	+	[E] Lactoylglutathione lyase and related lyases
287							
	MJLIPHIC_00595	CDS	COG3315	685104	685958	-	[Q] O-Methyltransferase involved in polyketide biosynthesis
	MJLIPHIC_00596	CDS	ROG2481	685955	686830	-	NA
288							
	MJLIPHIC_00597	CDS	COG1236	686934	687842	+	[J] Predicted exonuclease of the beta-lactamase fold
	MJLIPHIC_00598	CDS	COG2272	687835	689295	+	[I] Carboxylesterase type B
289							
	MJLIPHIC_00599	CDS	COG1309	689301	689912	-	[K] Transcriptional regulator
290							
	MJLIPHIC_00600	CDS	COG3662	689991	690890	+	[S] Uncharacterized protein conserved in bacteria
	MJLIPHIC_00601	CDS	COG3315	690921	691814	+	[Q] O-Methyltransferase involved in polyketide biosynthesis
291							

	MJLIPHIC_00602	CDS	COG0464	691941	693767	+	[O] ATPases of the AAA+ class
	MJLIPHIC_00603	CDS	ROG3025	693764	695320	+	NA
	MJLIPHIC_00604	CDS	COG1674	695317	699294	+	[D] DNA segregation ATPase FtsK/SpoIIIE and related
	MJLIPHIC_00605	CDS	NA	699291	699602	+	NA
	MJLIPHIC_00606	CDS	COG5651	699604	701175	+	[N] PPE-repeat proteins
	MJLIPHIC_00607	CDS	NA	701225	701518	+	NA
	MJLIPHIC_00608	CDS	COG4842	701553	701840	+	[S] Uncharacterized protein conserved in bacteria
	MJLIPHIC_00609	CDS	ROG4760	701853	702734	+	NA
	MJLIPHIC_00610	CDS	ROG3360	702755	704164	+	NA
	MJLIPHIC_00611	CDS	COG1404	704172	705551	+	[O] Subtilisin-like serine proteases
	MJLIPHIC_00612	CDS	ROG7780	705548	706477	+	NA
292							
	MJLIPHIC_00613	CDS	ROG5074	706433	707632	-	NA
	MJLIPHIC_00614	CDS	COG1807	707650	709404	-	[M] 4-amino-4-deoxy-L-arabinose transferase and related glycosyltransferases of
	MJLIPHIC_00615	CDS	COG2159	709441	710328	-	[R] Predicted metal-dependent hydrolase of the TIM-barrel
293							
	MJLIPHIC_00616	CDS	COG4106	710334	711137	+	[R] Trans-aconitate methyltransferase
294							
	MJLIPHIC_00617	CDS	COG4424	711186	711989	-	[S] Uncharacterized protein conserved in bacteria
295							
	MJLIPHIC_00618	CDS	COG3119	712049	713446	-	[P] Arylsulfatase A and related enzymes
296							
	MJLIPHIC_00619	CDS	NA	713531	714025	-	NA
297							
	MJLIPHIC_00620	CDS	COG0671	714093	714764	+	[I] Membrane-associated phospholipid phosphatase
298							
	MJLIPHIC_00621	CDS	COG1597	714718	716181	-	[IR] Sphingosine kinase and enzymes related to
299							
	MJLIPHIC_00622	CDS	COG3786	716330	716914	+	[S] Uncharacterized protein conserved in bacteria
300							
	MJLIPHIC_00623	CDS	ROG3864	716920	717405	-	NA
	MJLIPHIC_00624	CDS	COG0665	717434	718954	-	[E] Glycine/D-amino acid oxidases (deaminating)
301							
	MJLIPHIC_00625	CDS	COG0443	719172	721103	+	[O] Molecular chaperone
302							
	MJLIPHIC_00626	CDS	COG4608	721104	722114	-	[E] ABC-type oligopeptide transport system, ATPase component
	MJLIPHIC_00627	CDS	COG0444	722111	723097	-	[EP] ABC-type dipeptide/oligopeptide/nickel transport system, ATPase component
	MJLIPHIC_00628	CDS	COG0601	723094	724056	-	[EP] ABC-type dipeptide/oligopeptide/nickel transport systems, permease components
	MJLIPHIC_00629	CDS	COG1173	724060	725070	-	[EP] ABC-type dipeptide/oligopeptide/nickel transport systems, permease components
	MJLIPHIC_00630	CDS	COG0747	725067	726722	-	[E] ABC-type dipeptide transport system, periplasmic component
303							
	MJLIPHIC_00631	CDS	COG1917	727054	727503	+	[S] Uncharacterized conserved protein, contains double-stranded beta-helix
304							
	MJLIPHIC_00632	CDS	COG2273	727632	728471	+	[G] Beta-glucanase/Beta-glucan synthetase
305							
	MJLIPHIC_00633	CDS	COG3639	728572	730170	-	[P] ABC-type phosphate/phosphonate transport system, permease component
	MJLIPHIC_00634	CDS	COG3638	730167	730967	-	[P] ABC-type phosphate/phosphonate transport system, ATPase component
	MJLIPHIC_00635	CDS	COG3221	730969	731934	-	[P] ABC-type phosphate/phosphonate transport system, periplasmic component
306							
	MJLIPHIC_00636	CDS	COG2188	732117	732869	+	[K] Transcriptional regulators
307							
	MJLIPHIC_00637	CDS	ROG3702	732984	733664	+	NA
	MJLIPHIC_00638	CDS	ROG1586	733710	734573	+	NA
	MJLIPHIC_00639	CDS	ROG2408	734605	735426	+	NA
308							
	MJLIPHIC_00640	CDS	NA	735585	736505	-	NA
309							
	MJLIPHIC_00641	CDS	COG1028	736636	737415	+	[IQR] Dehydrogenases with different specificities (related to
310							
	MJLIPHIC_00642	CDS	COG3226	737416	738018	-	[S] Uncharacterized protein conserved in bacteria
	MJLIPHIC_00643	CDS	COG4638	738064	739248	-	[PR] Phenylpropionate dioxygenase and related ring-hydroxylating dioxygenases,
	MJLIPHIC_00644	CDS	COG1177	739263	740168	-	[E] ABC-type spermidine/putrescine transport system, permease component
	MJLIPHIC_00645	CDS	COG1176	740165	741106	-	[E] ABC-type spermidine/putrescine transport system, permease component
	MJLIPHIC_00646	CDS	COG0687	741111	742385	-	[E] Spermidine/putrescine-binding periplasmic protein
311							
	MJLIPHIC_00647	CDS	COG0001	742519	743907	+	[H] Glutamate-1-semialdehyde aminotransferase
	MJLIPHIC_00648	CDS	COG3842	743924	745081	+	[E] ABC-type spermidine/putrescine transport systems, ATPase components
312							
	MJLIPHIC_00649	CDS	COG1309	745088	745795	-	[K] Transcriptional regulator
313							
	MJLIPHIC_00650	CDS	COG1233	745913	747574	+	[Q] Phytoene dehydrogenase and related proteins
	MJLIPHIC_00651	CDS	COG1042	747628	749646	+	[C] Acyl-CoA synthetase (NDP forming)
314							
	MJLIPHIC_00652	CDS	COG0665	749627	750838	-	[E] Glycine/D-amino acid oxidases (deaminating)
315							
	MJLIPHIC_00653	CDS	COG1960	750914	752092	+	[I] Acyl-CoA dehydrogenases
	MJLIPHIC_00654	CDS	NA	752138	752347	+	NA
	MJLIPHIC_00655	CDS	COG0665	752392	753333	+	[E] Glycine/D-amino acid oxidases (deaminating)
316							
	MJLIPHIC_00656	CDS	NA	753330	753908	-	NA
	MJLIPHIC_00657	CDS	COG1063	753920	755068	-	[ER] Threonine dehydrogenase and related Zn-dependent dehydrogenases
317							
	MJLIPHIC_00658	CDS	ROG3614	755161	755814	-	NA
318							
	MJLIPHIC_00659	CDS	NA	755982	756524	-	NA
319							
	MJLIPHIC_00660	CDS	NA	756845	757861	-	NA
320							
	MJLIPHIC_00661	CDS	COG2124	758055	759272	-	[Q] Cytochrome P450
321							
	MJLIPHIC_00662	CDS	COG1309	759460	759996	+	[K] Transcriptional regulator
322							
	MJLIPHIC_00663	tRNA	NA	760001	760074	-	NA
323							
	MJLIPHIC_00664	CDS	COG0717	760192	760761	+	[F] Deoxycytidine deaminase
324							
	MJLIPHIC_00665	CDS	NA	761054	762742	+	NA
325							
	MJLIPHIC_00666	CDS	COG1004	762824	764170	+	[M] Predicted UDP-glucose 6-dehydrogenase
326							

	MJLIPHIC_00667	CDS	COG2124	764221	765516	+	[Q] Cytochrome P450
	MJLIPHIC_00668	CDS	ROG2730	765518	766270	+	NA
	MJLIPHIC_00669	CDS	ROG0530	766289	766657	+	NA
327							
	MJLIPHIC_00670	CDS	COG1529	766670	768784	-	[C] Aerobic-type carbon monoxide dehydrogenase, large subunit
	MJLIPHIC_00671	CDS	COG1319	768781	769776	-	[C] Aerobic-type carbon monoxide dehydrogenase, middle subunit
	MJLIPHIC_00672	CDS	COG2080	769773	770294	-	[C] Aerobic-type carbon monoxide dehydrogenase, small subunit
	MJLIPHIC_00673	CDS	COG5438	770334	771569	-	[S] Predicted multitransmembrane protein
	MJLIPHIC_00674	CDS	COG0436	771588	772862	-	[E] Aspartate/tyrosine/aromatic aminotransferase
328							
	MJLIPHIC_00675	CDS	NA	773110	773346	+	NA
329							
	MJLIPHIC_00676	CDS	COG0247	773427	776555	-	[C] Fe-S oxidoreductase
330							
	MJLIPHIC_00677	CDS	COG2771	776854	779364	-	[K] DNA-binding HTH domain-containing proteins
	MJLIPHIC_00678	CDS	COG1924	779379	781166	-	[I] Activator of 2-hydroxyglutaryl-CoA dehydratase (HSP70-class ATPase
331							
	MJLIPHIC_00679	CDS	NA	781385	781909	+	NA
332							
	MJLIPHIC_00680	CDS	NA	782191	783270	+	NA
333							
	MJLIPHIC_00681	CDS	COG0699	783358	785247	+	[R] Predicted GTPases (dynamin-related)
334							
	MJLIPHIC_00682	CDS	ROG2657	785252	785593	-	NA
	MJLIPHIC_00683	CDS	ROG8144	785583	785996	-	NA
335							
	MJLIPHIC_00684	CDS	COG0699	786166	787659	+	[R] Predicted GTPases (dynamin-related)
	MJLIPHIC_00685	CDS	COG1924	787715	789565	+	[I] Activator of 2-hydroxyglutaryl-CoA dehydratase (HSP70-class ATPase
336							
	MJLIPHIC_00686	CDS	ROG0995	789578	790090	-	NA
337							
	MJLIPHIC_00687	CDS	NA	790135	790428	+	NA
338							
	MJLIPHIC_00688	CDS	COG2141	790621	791553	-	[C] Coenzyme F420-dependent N5,N10-methylene tetrahydromethanopterin reductase and
	MJLIPHIC_00689	CDS	ROG0417	791573	792196	-	NA
339							
	MJLIPHIC_00690	CDS	ROG5856	792264	792803	-	NA
	MJLIPHIC_00691	CDS	ROG6218	792856	794181	-	NA
	MJLIPHIC_00692	CDS	COG0684	794200	794811	-	[H] Demethylmenaquinone methyltransferase
	MJLIPHIC_00693	CDS	COG0684	794808	795500	-	[H] Demethylmenaquinone methyltransferase
340							
	MJLIPHIC_00694	CDS	COG0583	795587	796540	+	[K] Transcriptional regulator
341							
	MJLIPHIC_00695	CDS	COG0443	796766	798634	+	[O] Molecular chaperone
	MJLIPHIC_00696	CDS	COG0576	798631	799281	+	[O] Molecular chaperone GrpE (heat shock protein)
	MJLIPHIC_00697	CDS	COG0484	799323	800495	+	[O] DnaJ-class molecular chaperone with C-terminal Zn
	MJLIPHIC_00698	CDS	ROG6095	800492	800851	+	NA
342							
	MJLIPHIC_00699	CDS	COG1309	800848	801438	-	[K] Transcriptional regulator
	MJLIPHIC_00700	CDS	COG1960	801516	802700	-	[I] Acyl-CoA dehydrogenases
	MJLIPHIC_00701	CDS	COG1028	802729	803472	-	[IQR] Dehydrogenases with different specificities (related to
343							
	MJLIPHIC_00702	CDS	COG0477	803623	804960	+	[GEPR] Permeases of the major facilitator superfamily
	MJLIPHIC_00703	CDS	COG0318	804960	806609	+	[IQ] Acyl-CoA synthetases (AMP-forming)/AMP-acid ligases II
344							
	MJLIPHIC_00704	CDS	NA	806596	807498	-	NA
	MJLIPHIC_00705	CDS	COG0543	807543	808712	-	[HC] 2-polyprenylphenol hydroxylase and related flavodoxin oxidoreductases
	MJLIPHIC_00706	CDS	ROG3529	808724	809722	-	NA
345							
	MJLIPHIC_00707	CDS	NA	809984	810451	+	NA
346							
	MJLIPHIC_00708	CDS	NA	810514	811017	-	NA
347							
	MJLIPHIC_00709	CDS	NA	811187	811729	-	NA
348							
	MJLIPHIC_00710	CDS	COG2963	811835	812161	+	[L] Transposase and inactivated derivatives
	MJLIPHIC_00711	CDS	COG2801	812164	813117	+	[L] Transposase and inactivated derivatives
349							
	MJLIPHIC_00712	CDS	NA	813145	813267	-	NA
350							
	MJLIPHIC_00713	CDS	NA	814022	814210	-	NA
351							
	MJLIPHIC_00714	CDS	NA	814372	814698	-	NA
352							
	MJLIPHIC_00715	CDS	ROG2658	814904	815572	-	NA
353							
	MJLIPHIC_00716	CDS	COG1819	815642	816844	-	[GC] Glycosyl transferases, related to UDP-glucuronosyltransferase
	MJLIPHIC_00717	CDS	COG2954	816851	817474	-	[S] Uncharacterized protein conserved in bacteria
354							
	MJLIPHIC_00718	CDS	COG0542	817638	820184	+	[O] ATPases with chaperone activity, ATP-binding subunit
355							
	MJLIPHIC_00719	CDS	COG3804	820263	821342	-	[S] Uncharacterized conserved protein related to dihydrodipicolinate
	MJLIPHIC_00720	CDS	COG4638	821339	822400	-	[PR] Phenylpropionate dioxygenase and related ring-hydroxylating dioxygenases,
356							
	MJLIPHIC_00721	CDS	NA	822502	823164	+	NA
357							
	MJLIPHIC_00722	CDS	ROG2696	823231	824067	+	NA
	MJLIPHIC_00723	CDS	COG0300	824113	824889	+	[R] Short-chain dehydrogenases of various substrate specificities
	MJLIPHIC_00724	CDS	NA	824920	825822	+	NA
	MJLIPHIC_00725	CDS	COG0566	825815	826459	+	[J] rRNA methylases
	MJLIPHIC_00726	CDS	COG4833	826475	827656	+	[G] Predicted glycosyl hydrolase
	MJLIPHIC_00727	CDS	NA	827660	828568	+	NA
358							
	MJLIPHIC_00728	CDS	COG0583	828573	829493	-	[K] Transcriptional regulator
359							
	MJLIPHIC_00729	CDS	COG1975	829538	830698	+	[O] Xanthine and CO dehydrogenases maturation factor,
	MJLIPHIC_00730	CDS	COG1319	830754	831644	+	[C] Aerobic-type carbon monoxide dehydrogenase, middle subunit
	MJLIPHIC_00731	CDS	COG2080	831647	832123	+	[C] Aerobic-type carbon monoxide dehydrogenase, small subunit
	MJLIPHIC_00732	CDS	COG1529	832120	834519	+	[C] Aerobic-type carbon monoxide dehydrogenase, large subunit
	MJLIPHIC_00733	CDS	COG1975	834538	835275	+	[O] Xanthine and CO dehydrogenases maturation factor,

MJLIPHIC_00734 CDS COG5271_4X 835265 836161 + NA
MJLIPHIC_00735 CDS COG3427 836174 836869 + [S] Uncharacterized conserved protein
MJLIPHIC_00736 CDS COG3552 836866 838059 + [R] Protein containing von Willebrand factor type
360
MJLIPHIC_00737 CDS COG0586 838056 838778 - [S] Uncharacterized membrane-associated protein
361
MJLIPHIC_00738 CDS COG0191 838859 839896 + [G] Fructose/tagatose bisphosphate aldolase
362
MJLIPHIC_00739 CDS COG4319 839930 840730 - [S] Ketosteroid isomerase homolog
363
MJLIPHIC_00740 CDS ROG0441 840804 841211 + NA
364
MJLIPHIC_00741 CDS COG1230 841222 842115 - [P] Co/Zn/Cd efflux system component
365
MJLIPHIC_00742 CDS NA 842184 842702 + NA
366
MJLIPHIC_00743 CDS COG1994 842699 843478 - [R] Zn-dependent proteases
MJLIPHIC_00744 CDS ROG1973 843524 844105 - NA
367
MJLIPHIC_00745 CDS COG0104 844214 845509 + [F] Adenylosuccinate synthase
MJLIPHIC_00746 CDS ROG4581 845509 846159 + NA
368
MJLIPHIC_00747 CDS ROG3199 846166 846372 - NA
MJLIPHIC_00748 CDS COG2124 846366 847571 - [Q] Cytochrome P450
MJLIPHIC_00749 CDS COG2409 847627 849633 - [R] Predicted drug exporters of the RND
MJLIPHIC_00750 CDS COG0025 849626 851209 - [P] NhaP-type Na+/H+ and K+/H+ antiporters
369
MJLIPHIC_00751 CDS COG0027 851580 852980 + [F] Formate-dependent phosphoribosylglycinamide formyltransferase (GAR transformylase)
MJLIPHIC_00752 CDS NA 852984 853469 + NA
MJLIPHIC_00753 CDS COG0607 853430 853876 + [P] Rhodanese-related sulfurtransferase
MJLIPHIC_00754 CDS COG0626 853886 855103 + [E] Cystathionine beta-lyases/cystathionine gamma-synthases
MJLIPHIC_00755 CDS NA 855107 855514 + NA
370
MJLIPHIC_00756 CDS COG1028 855584 856522 - [IQR] Dehydrogenases with different specificities (related to
MJLIPHIC_00757 CDS COG4222 856524 857666 - [S] Uncharacterized protein conserved in bacteria
371
MJLIPHIC_00758 CDS COG0454 857779 858309 + [KR] Histone acetyltransferase HPA2 and related acetyltransferases
372
MJLIPHIC_00759 CDS COG2141 858581 859402 + [C] Coenzyme F420-dependent N5,N10-methylene tetrahydromethanopterin reductase and
373
MJLIPHIC_00760 CDS NA 859468 859779 + NA
374
MJLIPHIC_00761 CDS COG1235 859776 860522 - [R] Metal-dependent hydrolases of the beta-lactamase superfamily
375
MJLIPHIC_00762 CDS NA 860554 861567 + NA
376
MJLIPHIC_00763 CDS COG2188 861746 862510 - [K] Transcriptional regulators
MJLIPHIC_00764 CDS COG1028 862519 863295 - [IQR] Dehydrogenases with different specificities (related to
MJLIPHIC_00765 CDS COG2334 863307 864413 - [R] Putative homoserine kinase type II (protein
377
MJLIPHIC_00766 CDS COG0531 864599 866176 + [E] Amino acid transporters
MJLIPHIC_00767 CDS COG0160 866234 867556 + [E] 4-aminobutyrate aminotransferase and related aminotransferases
MJLIPHIC_00768 CDS COG0280 867578 869656 + [C] Phosphotransacetylase
MJLIPHIC_00769 CDS COG0282 869653 870783 + [C] Acetate kinase
378
MJLIPHIC_00770 CDS ROG3094 870902 871771 - NA
379
MJLIPHIC_00771 CDS COG0515 872016 874298 - [RTKL] Serine/threonine protein kinase
MJLIPHIC_00772 CDS COG0834 874298 875266 - [ET] ABC-type amino acid transport/signal transduction systems,
380
MJLIPHIC_00773 CDS ROG1967 875362 876675 - NA
381
MJLIPHIC_00774 CDS COG1051 876865 877341 + [F] ADP-ribose pyrophosphatase
382
MJLIPHIC_00775 CDS COG0352 877325 878089 - [H] Thiamine monophosphate synthase
383
MJLIPHIC_00776 CDS COG0665 878147 879151 + [E] Glycine/D-amino acid oxidases (deaminating)
MJLIPHIC_00777 CDS COG2104 879148 879348 + [H] Sulfur transfer protein involved in thiamine
MJLIPHIC_00778 CDS COG2022 879341 880099 + [H] Uncharacterized enzyme of thiazole biosynthesis
MJLIPHIC_00779 CDS ROG0823 880096 880869 + NA
MJLIPHIC_00780 CDS COG1131 880883 881794 + [V] ABC-type multidrug transport system, ATPase component
MJLIPHIC_00781 CDS ROG1102 881791 882549 + NA
384
MJLIPHIC_00782 CDS ROG1194 882636 883745 + NA
MJLIPHIC_00783 CDS NA 883724 884989 + NA
MJLIPHIC_00784 CDS NA 885011 885736 + NA
385
MJLIPHIC_00785 CDS ROG0016 886770 887522 + NA
386
MJLIPHIC_00786 CDS COG3464 887530 888441 - [L] Transposase and inactivated derivatives
387
MJLIPHIC_00787 CDS NA 888643 889056 + NA
388
MJLIPHIC_00788 CDS NA 889186 889596 - NA
389
MJLIPHIC_00789 CDS NA 889880 890662 + NA
MJLIPHIC_00790 CDS COG2234 890688 892154 + [R] Predicted aminopeptidases
MJLIPHIC_00791 CDS COG2234 892151 893602 + [R] Predicted aminopeptidases
390
MJLIPHIC_00792 CDS COG3424 893674 894762 + [Q] Predicted naringenin-chalcone synthase
MJLIPHIC_00793 CDS COG1755 894762 895265 + [S] Uncharacterized protein conserved in bacteria
MJLIPHIC_00794 CDS ROG6287 895262 896290 + NA
391
MJLIPHIC_00795 CDS COG2233 896271 897566 - [F] Xanthine/uracil permeases
MJLIPHIC_00796 CDS COG0531 897612 898832 - [E] Amino acid transporters
MJLIPHIC_00797 CDS NA 898848 899174 - NA
392
MJLIPHIC_00798 CDS COG1309 899192 900652 + [K] Transcriptional regulator
393

	MJLIPHIC_00799	CDS	COG4270	900619	900891	-	[S] Predicted membrane protein
	MJLIPHIC_00800	CDS	COG2072	900908	902392	-	[P] Predicted flavoprotein involved in K+ transport
394							
	MJLIPHIC_00801	CDS	COG0583	902464	903387	+	[K] Transcriptional regulator
	MJLIPHIC_00802	CDS	COG0477	903499	904851	+	[GEPR] Permeases of the major facilitator superfamily
	MJLIPHIC_00803	CDS	NA	904883	906142	+	NA
	MJLIPHIC_00804	CDS	COG0154	906142	907605	+	[J] Asp-tRNAAsn/Glu-tRNAGln amidotransferase A subunit and related
395							
	MJLIPHIC_00805	CDS	ROG5047	907599	907868	-	NA
	MJLIPHIC_00806	CDS	COG2124	907874	909241	-	[Q] Cytochrome P450
	MJLIPHIC_00807	CDS	ROG2964	909288	909842	-	NA
	MJLIPHIC_00808	CDS	COG3571	909845	910492	-	[R] Predicted hydrolase of the alpha/beta-hydrolase fold
	MJLIPHIC_00809	CDS	COG0351	910482	911318	-	[H] Hydroxymethylpyrimidine/phosphomethylpyrimidine kinase
	MJLIPHIC_00810	CDS	COG0422	911327	912964	-	[H] Thiamine biosynthesis protein ThiC
396							
	MJLIPHIC_00811	CDS	COG2270	913283	915019	+	[R] Permeases of the major facilitator superfamily
397							
	MJLIPHIC_00812	CDS	NA	915135	916205	-	NA
398							
	MJLIPHIC_00813	CDS	COG0708	916410	917192	-	[L] Exonuclease III
399							
	MJLIPHIC_00814	CDS	COG1028	917379	918095	+	[IQR] Dehydrogenases with different specificities (related to
400							
	MJLIPHIC_00815	CDS	COG0454	918079	919005	-	[KR] Histone acetyltransferase HPA2 and related acetyltransferases
	MJLIPHIC_00816	CDS	COG0242	919024	919617	-	[J] N-formylmethionyl-tRNA deformylase
401							
	MJLIPHIC_00817	CDS	ROG0264	919745	920056	+	NA
	MJLIPHIC_00818	CDS	ROG4035	920105	920572	+	NA
	MJLIPHIC_00819	CDS	COG2032	920583	921293	+	[P] Cu/Zn superoxide dismutase
	MJLIPHIC_00820	CDS	COG2170	921274	922404	+	[S] Uncharacterized conserved protein
	MJLIPHIC_00821	CDS	NA	922415	922870	+	NA
402							
	MJLIPHIC_00822	CDS	COG1522	922879	923928	-	[K] Transcriptional regulators
403							
	MJLIPHIC_00823	CDS	COG2802	924025	924651	+	[R] Uncharacterized protein, similar to the N-terminal
404							
	MJLIPHIC_00824	CDS	ROG8360	924856	925416	-	NA
405							
	MJLIPHIC_00825	CDS	NA	925636	926976	-	NA
406							
	MJLIPHIC_00826	CDS	COG1320	927500	927853	-	[P] Multisubunit Na+/H+ antiporter, MnhG subunit
	MJLIPHIC_00827	CDS	COG2212	927850	928140	-	[P] Multisubunit Na+/H+ antiporter, MnhF subunit
	MJLIPHIC_00828	CDS	COG1863	928137	928676	-	[P] Multisubunit Na+/H+ antiporter, MnhE subunit
	MJLIPHIC_00829	CDS	COG0651	928673	930265	-	[CP] Formate hydrogenlyase subunit 3/Multisubunit Na+/H+ antiporter,
	MJLIPHIC_00830	CDS	COG1006	930265	930732	-	[P] Multisubunit Na+/H+ antiporter, MnhC subunit
	MJLIPHIC_00831	CDS	COG1009	930732	933611	-	[CP] NADH:ubiquinone oxidoreductase subunit 5 (chain L)/Multisubunit
407							
	MJLIPHIC_00832	CDS	COG0654	933753	934085	+	[HC] 2-polyprenyl-6-methoxyphenol hydroxylase and related FAD-dependent oxidoreductases
408							
	MJLIPHIC_00833	CDS	ROG3094	934162	935004	+	NA
409							
	MJLIPHIC_00834	CDS	COG1055	935059	936345	-	[P] Na+/H+ antiporter NhaD and related arsenite
	MJLIPHIC_00835	CDS	COG0517	936364	936822	-	[R] FOG: CBS domain
	MJLIPHIC_00836	CDS	NA	936855	937103	-	NA
410							
	MJLIPHIC_00837	CDS	COG4585	937236	938204	+	[T] Signal transduction histidine kinase
	MJLIPHIC_00838	CDS	COG2197	938204	938851	+	[TK] Response regulator containing a CheY-like receiver
411							
	MJLIPHIC_00839	CDS	ROG2484	938818	939270	-	NA
	MJLIPHIC_00840	CDS	NA	939331	939717	-	NA
412							
	MJLIPHIC_00841	CDS	ROG4697	939739	940752	+	NA
413							
	MJLIPHIC_00842	CDS	COG0464	940749	942986	-	[O] ATPases of the AAA+ class
	MJLIPHIC_00843	CDS	COG1183	942988	943848	-	[I] Phosphatidylserine synthase
	MJLIPHIC_00844	CDS	COG0688	943845	944543	-	[I] Phosphatidylserine decarboxylase
	MJLIPHIC_00845	CDS	COG0303	944579	945766	-	[H] Molybdopterin biosynthesis enzyme
	MJLIPHIC_00846	CDS	COG1028	945772	946695	-	[IQR] Dehydrogenases with different specificities (related to
414							
	MJLIPHIC_00847	CDS	COG3402	946733	947272	+	[S] Uncharacterized conserved protein
	MJLIPHIC_00848	CDS	COG3428	947269	948768	+	[S] Predicted membrane protein
	MJLIPHIC_00849	CDS	COG0647	948794	949609	+	[G] Predicted sugar phosphatases of the HAD
415							
	MJLIPHIC_00850	CDS	COG0467	949606	951312	-	[T] RecA-superfamily ATPases implicated in signal transduction
416							
	MJLIPHIC_00851	CDS	NA	951585	955409	-	NA
417							
	MJLIPHIC_00852	CDS	COG1529	956321	958486	-	[C] Aerobic-type carbon monoxide dehydrogenase, large subunit
	MJLIPHIC_00853	CDS	COG1319	958483	959457	-	[C] Aerobic-type carbon monoxide dehydrogenase, middle subunit
	MJLIPHIC_00854	CDS	COG2080	959461	960078	-	[C] Aerobic-type carbon monoxide dehydrogenase, small subunit
418							
	MJLIPHIC_00855	CDS	COG2186	960615	961319	+	[K] Transcriptional regulators
	MJLIPHIC_00856	CDS	COG0477	961391	962794	+	[GEPR] Permeases of the major facilitator superfamily
	MJLIPHIC_00857	CDS	COG0329	962791	963723	+	[EM] Dihydrodipicolinate synthase/N-acetylneuraminate lyase
419							
	MJLIPHIC_00858	CDS	COG1028	963720	964637	-	[IQR] Dehydrogenases with different specificities (related to
420							
	MJLIPHIC_00859	CDS	COG1309	964742	965305	+	[K] Transcriptional regulator
421							
	MJLIPHIC_00860	CDS	COG0459	965428	967158	+	[O] Chaperonin GroEL (HSP60 family)
422							
	MJLIPHIC_00861	CDS	ROG3094	967303	968136	+	NA
423							
	MJLIPHIC_00862	CDS	NA	968200	968583	-	NA
	MJLIPHIC_00863	CDS	COG3653	968609	970351	-	[Q] N-acyl-D-aspartate/D-glutamate deacylase
424							
	MJLIPHIC_00864	CDS	ROG5585	970433	970813	+	NA
425							
	MJLIPHIC_00865	CDS	ROG8378	970817	971293	-	NA

	MJLIPHIC_00866	CDS	COG0515	971320	972810	-	[RTKL] Serine/threonine protein kinase
426	MJLIPHIC_00867	CDS	ROG0796	972865	973422	+	NA
	MJLIPHIC_00868	CDS	COG3374	973419	973982	+	[S] Predicted membrane protein
	MJLIPHIC_00869	CDS	COG1012	973997	975526	+	[C] NAD-dependent aldehyde dehydrogenases
427	MJLIPHIC_00870	CDS	ROG0115	975595	976194	+	NA
	MJLIPHIC_00871	CDS	COG2326	976256	977107	+	[S] Uncharacterized conserved protein
	MJLIPHIC_00872	CDS	NA	977160	978005	+	NA
428	MJLIPHIC_00873	CDS	ROG8329	978024	978347	-	NA
429	MJLIPHIC_00874	CDS	COG4091	978540	979904	+	[E] Predicted homoserine dehydrogenase
430	MJLIPHIC_00875	CDS	COG2186	980004	980747	+	[K] Transcriptional regulators
	MJLIPHIC_00876	CDS	COG0477	980820	982190	+	[GEPR] Permeases of the major facilitator superfamily
	MJLIPHIC_00877	CDS	COG3395	982187	983464	+	[S] Uncharacterized protein conserved in bacteria
	MJLIPHIC_00878	CDS	COG0191	983461	984273	+	[G] Fructose/tagatose bisphosphate aldolase
431	MJLIPHIC_00879	CDS	COG1505	984361	986376	-	[E] Serine proteases of the peptidase family
432	MJLIPHIC_00880	CDS	COG1012	986441	987964	+	[C] NAD-dependent aldehyde dehydrogenases
	MJLIPHIC_00881	CDS	COG3564	987998	988474	+	[S] Uncharacterized protein conserved in bacteria
433	MJLIPHIC_00882	CDS	COG2230	988475	989344	-	[M] Cyclopropane fatty acid synthase and related
434	MJLIPHIC_00883	CDS	COG1249	989521	990915	+	[C] Pyruvate/2-oxoglutarate dehydrogenase complex, dihydrolipoamide dehydrogenase (E3)
	MJLIPHIC_00884	CDS	ROG7128	990876	991181	+	NA
435	MJLIPHIC_00885	CDS	ROG3446	991188	991748	-	NA
	MJLIPHIC_00886	CDS	COG3800	991776	993212	-	[R] Predicted transcriptional regulator
436	MJLIPHIC_00887	CDS	ROG1229	993284	993628	+	NA
	MJLIPHIC_00888	CDS	ROG1082	993604	994002	+	NA
437	MJLIPHIC_00889	CDS	COG3884	994104	994952	+	[I] Acyl-ACP thioesterase
438	MJLIPHIC_00890	CDS	COG2224	995201	996487	+	[C] Isocitrate lyase
439	MJLIPHIC_00891	CDS	COG1250	996663	997478	+	[I] 3-hydroxyacyl-CoA dehydrogenase
440	MJLIPHIC_00892	CDS	COG2230	997643	998515	+	[M] Cyclopropane fatty acid synthase and related
441	MJLIPHIC_00893	CDS	ROG3094	998555	999427	-	NA
442	MJLIPHIC_00894	CDS	COG2326	999537	1000424	-	[S] Uncharacterized conserved protein
	MJLIPHIC_00895	CDS	COG1309	1000429	1001106	-	[K] Transcriptional regulator
443	MJLIPHIC_00896	CDS	COG2733	1001253	1002569	+	[S] Predicted membrane protein
444	MJLIPHIC_00897	tRNA	NA	1002888	1002978	-	NA
445	MJLIPHIC_00898	CDS	ROG7770	1003169	1003867	+	NA
446	MJLIPHIC_00899	CDS	ROG1903	1003944	1004228	+	NA
	MJLIPHIC_00900	CDS	ROG6394	1004233	1004646	+	NA
	MJLIPHIC_00901	CDS	COG0274	1004646	1005329	+	[F] Deoxyribose-phosphate aldolase
447	MJLIPHIC_00902	CDS	ROG2917	1005367	1006302	-	NA
	MJLIPHIC_00903	CDS	COG0388	1006363	1007160	-	[R] Predicted amidohydrolase
	MJLIPHIC_00904	CDS	ROG0540	1007170	1007946	-	NA
	MJLIPHIC_00905	CDS	ROG6628	1007951	1008466	-	NA
	MJLIPHIC_00906	CDS	ROG1199	1008489	1008977	-	NA
448	MJLIPHIC_00907	CDS	COG0812	1009022	1010113	+	[M] UDP-N-acetylmuramate dehydrogenase
449	MJLIPHIC_00908	CDS	NA	1010183	1011478	+	NA
450	MJLIPHIC_00909	CDS	COG4221	1011488	1012204	-	[R] Short-chain alcohol dehydrogenase of unknown specificity
451	MJLIPHIC_00910	CDS	COG1940	1012429	1013760	+	[KG] Transcriptional regulator/sugar kinase
	MJLIPHIC_00911	CDS	COG0438	1013807	1015075	+	[M] Glycosyltransferase
	MJLIPHIC_00912	CDS	ROG1266	1015072	1015569	+	NA
	MJLIPHIC_00913	CDS	COG0588	1015609	1016352	+	[G] Phosphoglycerate mutase 1
452	MJLIPHIC_00914	CDS	COG5002	1016577	1017758	+	[T] Signal transduction histidine kinase
	MJLIPHIC_00915	CDS	COG0745	1017755	1018441	+	[TK] Response regulators consisting of a CheY-like
453	MJLIPHIC_00916	CDS	ROG0915	1018465	1019265	-	NA
454	MJLIPHIC_00917	CDS	COG0248	1019451	1020338	+	[FP] Exopolyphosphatase
	MJLIPHIC_00918	CDS	NA	1020368	1021438	+	NA
	MJLIPHIC_00919	CDS	COG1082	1021461	1022306	+	[G] Sugar phosphate isomerases/epimerases
	MJLIPHIC_00920	CDS	ROG2064	1022339	1023154	+	NA
	MJLIPHIC_00921	CDS	COG0345	1023180	1024040	+	[E] Pyrroline-5-carboxylate reductase
455	MJLIPHIC_00922	CDS	ROG5714	1024269	1024406	+	NA
456	MJLIPHIC_00923	CDS	COG0451	1024722	1025849	+	[MG] Nucleoside-diphosphate-sugar epimerases
	MJLIPHIC_00924	CDS	COG0204	1025886	1026950	+	[I] 1-acyl-sn-glycerol-3-phosphate acyltransferase
457	MJLIPHIC_00925	CDS	ROG0252	1026981	1027514	-	NA
458	MJLIPHIC_00926	CDS	COG0560	1027603	1028532	-	[E] Phosphoserine phosphatase
459	MJLIPHIC_00927	CDS	ROG4471	1028561	1028842	+	NA
460	MJLIPHIC_00928	CDS	COG0373	1029002	1030357	+	[H] Glutamyl-tRNA reductase
	MJLIPHIC_00929	CDS	COG0181	1030361	1031317	+	[H] Porphobilinogen deaminase

MJLIPHIC_00930 CDS COG1587 1031314 1033245 + [H] Uroporphyrinogen-III synthase
MJLIPHIC_00931 CDS COG0113 1033330 1034310 + [H] Delta-aminolevulinic acid dehydratase
MJLIPHIC_00932 CDS ROG7847 1034319 1034738 + NA
MJLIPHIC_00933 CDS NA 1034759 1035154 + NA
MJLIPHIC_00934 CDS ROG5755 1035147 1035683 + NA
MJLIPHIC_00935 CDS ROG0583 1035680 1035910 + NA
461
MJLIPHIC_00936 CDS COG3662 1035998 1036867 + [S] Uncharacterized protein conserved in bacteria
MJLIPHIC_00937 CDS COG1309 1036854 1037447 + [K] Transcriptional regulator
462
MJLIPHIC_00938 CDS NA 1037522 1037764 + NA
MJLIPHIC_00939 CDS COG0259 1037792 1038283 + [H] Pyridoxamine-phosphate oxidase
463
MJLIPHIC_00940 CDS NA 1038908 1039543 + NA
464
MJLIPHIC_00941 CDS ROG8109 1039684 1040067 - NA
MJLIPHIC_00942 CDS ROG8109 1040090 1040470 - NA
465
MJLIPHIC_00943 CDS COG2124 1040556 1042010 + [Q] Cytochrome P450
MJLIPHIC_00944 CDS COG0001 1042046 1043395 + [H] Glutamate-1-semialdehyde aminotransferase
MJLIPHIC_00945 CDS COG0406 1043392 1044054 + [G] Fructose-2,6-bisphosphatase
MJLIPHIC_00946 CDS COG0526 1044054 1044707 + [OC] Thiol-disulfide isomerase and thioredoxins
MJLIPHIC_00947 CDS COG0785 1044704 1045489 + [O] Cytochrome c biogenesis protein
MJLIPHIC_00948 CDS COG1333 1045489 1047138 + [O] ResB protein required for cytochrome c
MJLIPHIC_00949 CDS COG0755 1047138 1048130 + [O] ABC-type transport system involved in cytochrome
MJLIPHIC_00950 CDS COG0455 1048209 1049537 + [D] ATPases involved in chromosome partitioning
466
MJLIPHIC_00951 CDS ROG1154 1049555 1049719 - NA
467
MJLIPHIC_00952 CDS ROG3453 1049796 1050068 + NA
MJLIPHIC_00953 CDS COG0520 1050068 1051105 + [E] Selenocysteine lyase
468
MJLIPHIC_00954 CDS NA 1051203 1051451 + NA
469
MJLIPHIC_00955 CDS COG4585 1051528 1053159 - [T] Signal transduction histidine kinase
470
MJLIPHIC_00956 CDS COG3279 1053329 1053688 + [KT] Response regulator of the LytR/AlgR family
471
MJLIPHIC_00957 CDS COG2358 1053805 1054854 + [R] TRAP-type uncharacterized transport system, periplasmic component
MJLIPHIC_00958 CDS COG4666 1054857 1056935 + [R] TRAP-type uncharacterized transport system, fused permease
472
MJLIPHIC_00959 CDS COG2197 1056913 1057566 - [TK] Response regulator containing a CheY-like receiver
MJLIPHIC_00960 CDS COG0477 1057684 1059033 - [GEPR] Permeases of the major facilitator superfamily
473
MJLIPHIC_00961 CDS ROG6516 1059121 1059849 - NA
474
MJLIPHIC_00962 CDS COG3619 1059955 1060326 + [S] Predicted membrane protein
475
MJLIPHIC_00963 CDS COG1575 1060331 1061257 - [H] 1,4-dihydroxy-2-naphthoate octaprenyltransferase
476
MJLIPHIC_00964 CDS NA 1061291 1061536 + NA
MJLIPHIC_00965 CDS COG0005 1061536 1062312 + [F] Purine nucleoside phosphorylase
MJLIPHIC_00966 CDS COG0451 1062309 1063349 + [MG] Nucleoside-diphosphate-sugar epimerases
MJLIPHIC_00967 CDS COG2070 1063388 1064533 + [R] Dioxygenases related to 2-nitropropane dioxygenase
477
MJLIPHIC_00968 CDS COG2205 1064543 1065556 - [T] Osmosensitive K+ channel histidine kinase
MJLIPHIC_00969 CDS COG0745 1065553 1066230 - [TK] Response regulators consisting of a CheY-like
478
MJLIPHIC_00970 CDS COG1215 1066335 1066994 + [M] Glycosyltransferases, probably involved in cell wall
MJLIPHIC_00971 CDS COG3222 1066994 1067647 + [S] Uncharacterized protein conserved in bacteria
MJLIPHIC_00972 CDS COG0500 1067651 1068280 + [QR] SAM-dependent methyltransferases
MJLIPHIC_00973 CDS COG2041 1068277 1069431 + [R] Sulfite oxidase and related enzymes
MJLIPHIC_00974 CDS COG1276 1069428 1069922 + [P] Putative copper export protein
479
MJLIPHIC_00975 CDS ROG3320 1069848 1071212 - NA
480
MJLIPHIC_00976 CDS ROG4111 1071264 1072121 + NA
481
MJLIPHIC_00977 CDS ROG0016 1072342 1073094 + NA
482
MJLIPHIC_00978 CDS COG3464 1073102 1074013 - [L] Transposase and inactivated derivatives
483
MJLIPHIC_00979 CDS COG1733 1074482 1074643 - [K] Predicted transcriptional regulators
484
MJLIPHIC_00980 CDS COG0500 1074818 1075633 + [QR] SAM-dependent methyltransferases
MJLIPHIC_00981 CDS COG3173 1075656 1076717 + [R] Predicted aminoglycoside phosphotransferase
485
MJLIPHIC_00982 CDS COG0730 1076746 1077507 - [R] Predicted permeases
486
MJLIPHIC_00983 CDS NA 1077699 1078190 + NA
487
MJLIPHIC_00984 CDS COG2124 1078286 1079770 - [Q] Cytochrome P450
488
MJLIPHIC_00985 CDS COG1309 1079902 1080561 + [K] Transcriptional regulator
MJLIPHIC_00986 CDS COG4221 1080585 1081448 + [R] Short-chain alcohol dehydrogenase of unknown specificity
489
MJLIPHIC_00987 CDS COG1785 1081451 1082986 - [P] Alkaline phosphatase
490
MJLIPHIC_00988 CDS COG4637 1083088 1084320 - [R] Predicted ATPase
MJLIPHIC_00989 CDS COG0389 1084283 1085353 - [L] Nucleotidyltransferase/DNA polymerase involved in DNA repair
MJLIPHIC_00990 CDS COG1309 1085358 1085963 - [K] Transcriptional regulator
491
MJLIPHIC_00991 CDS COG0431 1086080 1086643 + [R] Predicted flavoprotein
492
MJLIPHIC_00992 CDS COG0695 1087117 1087356 + [O] Glutaredoxin and related proteins
MJLIPHIC_00993 CDS COG1780 1087501 1087836 + [F] Protein involved in ribonucleotide reduction
MJLIPHIC_00994 CDS COG0209 1087803 1089971 + [F] Ribonucleotide reductase, alpha subunit
493
MJLIPHIC_00995 CDS ROG1121 1090158 1090544 - NA
494

MJLIPHIC_00996 CDS NA 1090573 1091274 + NA
495
MJLIPHIC_00997 CDS COG3607 1091275 1091694 - [R] Predicted lactoylglutathione lyase
496
MJLIPHIC_00998 CDS COG0477 1091966 1093375 + [GEPR] Permeases of the major facilitator superfamily
MJLIPHIC_00999 CDS NA 1093372 1093515 + NA
MJLIPHIC_01000 CDS COG3393 1093566 1094417 + [R] Predicted acetyltransferase
497
MJLIPHIC_01001 CDS COG1309 1094389 1094964 - [K] Transcriptional regulator
498
MJLIPHIC_01002 CDS NA 1095088 1095966 + NA
499
MJLIPHIC_01003 CDS COG0644 1096036 1097223 - [C] Dehydrogenases (flavoproteins)
500
MJLIPHIC_01004 CDS COG1309 1097461 1098111 + [K] Transcriptional regulator
501
MJLIPHIC_01005 CDS COG2072 1098227 1099726 + [P] Predicted flavoprotein involved in K+ transport
502
MJLIPHIC_01006 CDS COG3631 1099812 1100336 + [R] Ketosteroid isomerase-related protein
503
MJLIPHIC_01007 CDS COG0208 1100541 1101527 + [F] Ribonucleotide reductase, beta subunit
504
MJLIPHIC_01008 CDS COG0627 1101597 1102979 + [R] Predicted esterase
MJLIPHIC_01009 CDS ROG5681 1102979 1104559 + NA
505
MJLIPHIC_01010 CDS COG2141 1104748 1106103 - [C] Coenzyme F420-dependent N5,N10-methylene tetrahydromethanopterin reductase and
506
MJLIPHIC_01011 CDS COG1064 1106170 1107219 - [R] Zn-dependent alcohol dehydrogenases
507
MJLIPHIC_01012 CDS COG2350 1107407 1107682 - [S] Uncharacterized protein conserved in bacteria
MJLIPHIC_01013 CDS COG0614 1107692 1108765 - [P] ABC-type Fe3+-hydroxamate transport system, periplasmic component
508
MJLIPHIC_01014 CDS COG0843 1109035 1110723 + [C] Heme/copper-type cytochrome/quinol oxidases, subunit 1
MJLIPHIC_01015 CDS COG0560 1110747 1111988 + [E] Phosphoserine phosphatase
509
MJLIPHIC_01016 CDS NA 1112119 1112409 + NA
510
MJLIPHIC_01017 CDS COG1167 1112411 1113838 - [KE] Transcriptional regulators containing a DNA-binding HTH
511
MJLIPHIC_01018 CDS COG2364 1114069 1114752 + [S] Predicted membrane protein
MJLIPHIC_01019 CDS COG1119 1114707 1115555 + [P] ABC-type molybdenum transport system, ATPase component/photorepair
MJLIPHIC_01020 CDS NA 1115548 1116369 + NA
MJLIPHIC_01021 CDS COG1024 1116366 1117073 + [I] Enoyl-CoA hydratase/carnithine racemase
MJLIPHIC_01022 CDS COG0500 1117108 1118088 + [QR] SAM-dependent methyltransferases
MJLIPHIC_01023 CDS ROG2908 1118097 1119260 + NA
512
MJLIPHIC_01024 CDS ROG0285 1119333 1120034 + NA
513
MJLIPHIC_01025 CDS COG1115 1120066 1121610 - [E] Na+/alanine symporter
514
MJLIPHIC_01026 CDS COG1520 1121728 1123011 - [S] FOG: WD40-like repeat
515
MJLIPHIC_01027 CDS COG1208 1123108 1123851 + [MJ] Nucleoside-diphosphate-sugar pyrophosphorylase involved in lipopolysaccharide biosynthesis/translation
516
MJLIPHIC_01028 CDS NA 1123863 1124141 - NA
517
MJLIPHIC_01029 CDS COG1304 1124631 1125650 + [C] L-lactate dehydrogenase (FMN-dependent) and related alpha-hydroxy
MJLIPHIC_01030 CDS ROG0016 1125608 1126309 + NA
518
MJLIPHIC_01031 CDS COG3464 1126317 1127228 - [L] Transposase and inactivated derivatives
519
MJLIPHIC_01032 CDS ROG0228 1127726 1128130 - NA
520
MJLIPHIC_01033 CDS COG3576 1128363 1128977 - [R] Predicted flavin-nucleotide-binding protein structurally related to
MJLIPHIC_01034 CDS COG0318 1129017 1130057 - [IQ] Acyl-CoA synthetases (AMP-forming)/AMP-acid ligases II
521
MJLIPHIC_01035 CDS ROG1140 1130153 1130476 - NA
522
MJLIPHIC_01036 CDS COG0306 1130758 1132014 + [P] Phosphate/sulphate permeases
MJLIPHIC_01037 CDS NA 1132030 1132308 + NA
MJLIPHIC_01038 CDS ROG1140 1132310 1132621 + NA
523
MJLIPHIC_01039 CDS ROG1880 1132639 1133010 - NA
MJLIPHIC_01040 CDS COG2508 1133059 1134474 - [TQ] Regulator of polyketide synthase expression
524
MJLIPHIC_01041 CDS COG0531 1134639 1136105 + [E] Amino acid transporters
MJLIPHIC_01042 CDS COG1028 1136102 1136797 + [IQR] Dehydrogenases with different specificities (related to
MJLIPHIC_01043 CDS COG1028 1136794 1137555 + [IQR] Dehydrogenases with different specificities (related to
MJLIPHIC_01044 CDS COG0010 1137579 1138550 + [E] Arginase/agmatinase/formimionoglutamate hydrolase, arginase family
MJLIPHIC_01045 CDS COG0726 1138540 1139403 + [G] Predicted xylanase/chitin deacetylase
525
MJLIPHIC_01046 CDS COG0300 1139384 1140298 - [R] Short-chain dehydrogenases of various substrate specificities
526
MJLIPHIC_01047 CDS COG0447 1140401 1141330 - [H] Dihydroxynaphthoic acid synthase
527
MJLIPHIC_01048 CDS NA 1141429 1141704 + NA
528
MJLIPHIC_01049 CDS ROG3956 1141706 1142155 - NA
MJLIPHIC_01050 CDS COG1011 1142177 1142866 - [R] Predicted hydrolase (HAD superfamily)
529
MJLIPHIC_01051 CDS NA 1143028 1143432 + NA
MJLIPHIC_01052 CDS ROG6789 1143429 1145873 + NA
MJLIPHIC_01053 CDS NA 1145899 1146657 + NA
530
MJLIPHIC_01054 CDS COG2197 1146744 1147487 + [TK] Response regulator containing a CheY-like receiver
531
MJLIPHIC_01055 CDS COG0747 1147718 1149322 + [E] ABC-type dipeptide transport system, periplasmic component
MJLIPHIC_01056 CDS COG0601 1149333 1150292 + [EP] ABC-type dipeptide/oligopeptide/nickel transport systems, permease components
MJLIPHIC_01057 CDS COG1173 1150289 1151224 + [EP] ABC-type dipeptide/oligopeptide/nickel transport systems, permease components

	MJLIPHIC_01058	CDS	COG1123	1151224	1152102	+	[R] ATPase components of various ABC-type transport
	MJLIPHIC_01059	CDS	COG1123	1152092	1152904	+	[R] ATPase components of various ABC-type transport
	MJLIPHIC_01060	CDS	COG0154	1152901	1154301	+	[J] Asp-tRNAAsn/Glu-tRNAGln amidotransferase A subunit and related
	MJLIPHIC_01061	CDS	NA	1154316	1154507	+	NA
	MJLIPHIC_01062	CDS	COG0154	1154545	1155951	+	[J] Asp-tRNAAsn/Glu-tRNAGln amidotransferase A subunit and related
	MJLIPHIC_01063	CDS	COG2371	1155953	1156411	+	[O] Urease accessory protein UreE
	MJLIPHIC_01064	CDS	COG0830	1156408	1157070	+	[O] Urease accessory protein UreF
	MJLIPHIC_01065	CDS	COG0832	1157081	1157737	+	[E] Urea amidohydrolase (urease) beta subunit
	MJLIPHIC_01066	CDS	COG0804	1157739	1159445	+	[E] Urea amidohydrolase (urease) alpha subunit
	MJLIPHIC_01067	CDS	COG0378	1159442	1160059	+	[OK] Ni2+-binding GTPase involved in regulation of
	MJLIPHIC_01068	CDS	COG0829	1160056	1160871	+	[O] Urease accessory protein UreH
	MJLIPHIC_01069	CDS	ROG3598	1160901	1161584	+	NA
532							
	MJLIPHIC_01070	CDS	COG0318	1161655	1163370	-	[IQ] Acyl-CoA synthetases (AMP-forming)/AMP-acid ligases II
533							
	MJLIPHIC_01071	CDS	NA	1163418	1163642	+	NA
534							
	MJLIPHIC_01072	CDS	COG3795	1163698	1164150	-	[S] Uncharacterized protein conserved in bacteria
535							
	MJLIPHIC_01073	CDS	COG1520	1164280	1165446	+	[S] FOG: WD40-like repeat
	MJLIPHIC_01074	CDS	COG1574	1165456	1167066	+	[R] Predicted metal-dependent hydrolase with the TIM-barrel
	MJLIPHIC_01075	CDS	COG4948	1167087	1168052	+	[MR] L-alanine-DL-glutamate epimerase and related enzymes of
536							
	MJLIPHIC_01076	CDS	COG3134	1168409	1168600	+	[S] Predicted outer membrane lipoprotein
537							
	MJLIPHIC_01077	CDS	NA	1168665	1169168	+	NA
	MJLIPHIC_01078	CDS	NA	1169158	1169811	+	NA
	MJLIPHIC_01079	CDS	COG4977	1169829	1170854	+	[K] Transcriptional regulator containing an amidase domain
	MJLIPHIC_01080	CDS	COG4977	1170870	1171601	+	[K] Transcriptional regulator containing an amidase domain
	MJLIPHIC_01081	CDS	COG0596	1171639	1172457	+	[R] Predicted hydrolases or acyltransferases (alpha/beta hydrolase
	MJLIPHIC_01082	CDS	COG1165	1172457	1174100	+	[H] 2-succinyl-6-hydroxy-2,4-cyclohexadiene-1-carboxylate synthase
	MJLIPHIC_01083	CDS	ROG1994	1174097	1174594	+	NA
538							
	MJLIPHIC_01084	CDS	ROG6760	1174598	1175275	-	NA
539							
	MJLIPHIC_01085	CDS	COG0438	1175363	1176490	+	[M] Glycosyltransferase
540							
	MJLIPHIC_01086	CDS	COG1048	1176487	1178451	-	[C] Aconitase A
541							
	MJLIPHIC_01087	CDS	COG4221	1178579	1179403	-	[R] Short-chain alcohol dehydrogenase of unknown specificity
542							
	MJLIPHIC_01088	CDS	COG2226	1179428	1180222	+	[H] Methylase involved in ubiquinone/menaquinone biosynthesis
543							
	MJLIPHIC_01089	CDS	COG0174	1180363	1181715	-	[E] Glutamine synthetase
544							
	MJLIPHIC_01090	CDS	COG2186	1181811	1182530	+	[K] Transcriptional regulators
	MJLIPHIC_01091	CDS	COG0531	1182618	1184153	+	[E] Amino acid transporters
	MJLIPHIC_01092	CDS	COG2071	1184153	1184869	+	[R] Predicted glutamine amidotransferases
	MJLIPHIC_01093	CDS	COG0388	1184839	1185837	+	[R] Predicted amidohydrolase
	MJLIPHIC_01094	CDS	NA	1185846	1186217	+	NA
545							
	MJLIPHIC_01095	CDS	COG0523	1186466	1187440	+	[R] Putative GTPases (G3E family)
546							
	MJLIPHIC_01096	CDS	COG1146	1187553	1187897	+	[C] Ferredoxin
	MJLIPHIC_01097	CDS	COG2345	1187897	1188550	+	[K] Predicted transcriptional regulator
	MJLIPHIC_01098	CDS	ROG6277	1188580	1188864	+	NA
	MJLIPHIC_01099	CDS	ROG7907	1188880	1189476	+	NA
547							
	MJLIPHIC_01100	CDS	NA	1189652	1189771	-	NA
548							
	MJLIPHIC_01101	CDS	COG0665	1189972	1191243	+	[E] Glycine/D-amino acid oxidases (deaminating)
549							
	MJLIPHIC_01102	CDS	ROG5631	1191431	1191622	+	NA
550							
	MJLIPHIC_01103	CDS	ROG4835	1191772	1192899	+	NA
551							
	MJLIPHIC_01104	CDS	COG3476	1192990	1193475	+	[T] Tryptophan-rich sensory protein (mitochondrial benzodiazepine receptor
552							
	MJLIPHIC_01105	CDS	COG0644	1193567	1194709	-	[C] Dehydrogenases (flavoproteins)
553							
	MJLIPHIC_01106	CDS	COG0142	1194836	1195825	+	[H] Geranylgeranyl pyrophosphate synthase
	MJLIPHIC_01107	CDS	COG0501	1195912	1196799	+	[O] Zn-dependent protease with chaperone function
554							
	MJLIPHIC_01108	CDS	ROG2604	1197495	1197794	+	NA
555							
	MJLIPHIC_01109	CDS	NA	1197892	1198365	+	NA
556							
	MJLIPHIC_01110	CDS	COG0531	1198367	1199806	-	[E] Amino acid transporters
	MJLIPHIC_01111	CDS	COG1062	1199825	1200988	-	[C] Zn-dependent alcohol dehydrogenases, class III
557							
	MJLIPHIC_01112	CDS	COG1396	1201113	1201682	+	[K] Predicted transcriptional regulators
558							
	MJLIPHIC_01113	CDS	COG0240	1201699	1202724	-	[C] Glycerol-3-phosphate dehydrogenase
559							
	MJLIPHIC_01114	CDS	COG0767	1203291	1204160	+	[Q] ABC-type transport system involved in resistance
	MJLIPHIC_01115	CDS	COG0767	1204162	1205019	+	[Q] ABC-type transport system involved in resistance
	MJLIPHIC_01116	CDS	COG1463	1205021	1206625	+	[Q] ABC-type transport system involved in resistance
	MJLIPHIC_01117	CDS	COG1463	1206622	1207677	+	[Q] ABC-type transport system involved in resistance
	MJLIPHIC_01118	CDS	COG1463	1207670	1208710	+	[Q] ABC-type transport system involved in resistance
	MJLIPHIC_01119	CDS	COG1463	1208707	1209828	+	[Q] ABC-type transport system involved in resistance
	MJLIPHIC_01120	CDS	COG1463	1209828	1211105	+	[Q] ABC-type transport system involved in resistance
	MJLIPHIC_01121	CDS	COG1463	1211102	1212358	+	[Q] ABC-type transport system involved in resistance
	MJLIPHIC_01122	CDS	ROG5477	1212385	1213071	+	NA
	MJLIPHIC_01123	CDS	ROG7723	1213041	1213862	+	NA
560							
	MJLIPHIC_01124	CDS	COG1396	1214090	1214695	+	[K] Predicted transcriptional regulators
561							
	MJLIPHIC_01125	CDS	NA	1214575	1214826	-	NA
562							
	MJLIPHIC_01126	CDS	COG0477	1214906	1216132	+	[GEPR] Permeases of the major facilitator superfamily

MJLIPHIC_01127 CDS COG1148 1216141 1217541 + [C] Heterodisulfide reductase, subunit A and related
MJLIPHIC_01128 CDS COG1804 1217538 1218719 + [C] Predicted acyl-CoA transferases/carnitine dehydratase
MJLIPHIC_01129 CDS COG0447 1218716 1219522 + [H] Dihydroxynaphthoic acid synthase
MJLIPHIC_01130 CDS NA 1219519 1220406 + NA
MJLIPHIC_01131 CDS COG1028 1220407 1221180 + [IQR] Dehydrogenases with different specificities (related to
MJLIPHIC_01132 CDS COG1012 1221223 1222650 + [C] NAD-dependent aldehyde dehydrogenases
563
MJLIPHIC_01133 CDS COG0715 1222785 1223855 + [P] ABC-type nitrate/sulfonate/bicarbonate transport systems, periplasmic components
MJLIPHIC_01134 CDS COG1116 1223852 1224655 + [P] ABC-type nitrate/sulfonate/bicarbonate transport system, ATPase component
MJLIPHIC_01135 CDS COG0600 1224648 1225493 + [P] ABC-type nitrate/sulfonate/bicarbonate transport system, permease component
MJLIPHIC_01136 CDS COG2141 1225516 1226862 + [C] Coenzyme F420-dependent N5,N10-methylene tetrahydromethanopterin reductase and
564
MJLIPHIC_01137 CDS NA 1226978 1227250 + NA
MJLIPHIC_01138 CDS NA 1227282 1227494 + NA
MJLIPHIC_01139 CDS NA 1227491 1227760 + NA
565
MJLIPHIC_01140 CDS COG1666 1227792 1228283 - [S] Uncharacterized protein conserved in bacteria
566
MJLIPHIC_01141 tRNA NA 1228393 1228477 + NA
567
MJLIPHIC_01142 CDS COG2124 1228550 1229989 - [Q] Cytochrome P450
568
MJLIPHIC_01143 CDS COG2207 1230134 1230784 + [K] AraC-type DNA-binding domain-containing proteins
569
MJLIPHIC_01144 CDS ROG2472 1230896 1232140 + NA
570
MJLIPHIC_01145 CDS NA 1232305 1232415 + NA
571
MJLIPHIC_01146 CDS COG0394 1232517 1232927 - [T] Protein-tyrosine-phosphatase
MJLIPHIC_01147 CDS COG0798 1232924 1234000 - [P] Arsenite efflux pump ACR3 and related
MJLIPHIC_01148 CDS COG0640 1233997 1234275 - [K] Predicted transcriptional regulators
572
MJLIPHIC_01149 CDS COG2514 1234447 1234911 - [R] Predicted ring-cleavage extradiol dioxygenase
573
MJLIPHIC_01150 CDS COG0640 1235012 1235371 + [K] Predicted transcriptional regulators
574
MJLIPHIC_01151 CDS NA 1235441 1236928 + NA
575
MJLIPHIC_01152 CDS COG1457 1236938 1238374 - [F] Purine-cytosine permease and related proteins
576
MJLIPHIC_01153 CDS COG1737 1238728 1239618 + [K] Transcriptional regulators
MJLIPHIC_01154 CDS COG2987 1239680 1241338 + [E] Urocanate hydratase
MJLIPHIC_01155 CDS NA 1241335 1242549 + NA
MJLIPHIC_01156 CDS COG0402 1242546 1243877 + [FR] Cytosine deaminase and related metal-dependent hydrolases
MJLIPHIC_01157 CDS COG1228 1243874 1245064 + [Q] Imidazolonepropionase and related amidohydrolases
MJLIPHIC_01158 CDS COG2986 1245061 1246605 + [E] Histidine ammonia-lyase
577
MJLIPHIC_01159 CDS ROG5614 1246752 1247582 + NA
578
MJLIPHIC_01160 CDS COG1522 1247625 1248647 - [K] Transcriptional regulators
579
MJLIPHIC_01161 CDS COG1757 1248760 1250229 + [C] Na+/H+ antiporter
MJLIPHIC_01162 CDS COG3653 1250238 1251833 + [Q] N-acyl-D-aspartate/D-glutamate deacylase
580
MJLIPHIC_01163 CDS NA 1251852 1253759 - NA
581
MJLIPHIC_01164 CDS NA 1253948 1255630 + NA
582
MJLIPHIC_01165 CDS COG0515 1255749 1257644 + [RTKL] Serine/threonine protein kinase
583
MJLIPHIC_01166 CDS NA 1257700 1257921 - NA
MJLIPHIC_01167 CDS COG0739 1257963 1259198 - [M] Membrane proteins related to metalloendopeptidases
584
MJLIPHIC_01168 CDS ROG1358 1259580 1261274 + NA
585
MJLIPHIC_01169 CDS NA 1261318 1261758 - NA
586
MJLIPHIC_01170 CDS COG0643 1261917 1263128 + [NT] Chemotaxis protein histidine kinase and related
587
MJLIPHIC_01171 CDS COG0553 1263197 1265188 - [KL] Superfamily II DNA/RNA helicases, SNF2 family
MJLIPHIC_01172 CDS COG2771 1265252 1265509 - [K] DNA-binding HTH domain-containing proteins
588
MJLIPHIC_01173 CDS NA 1265675 1266436 + NA
589
MJLIPHIC_01174 CDS COG0392 1266507 1268882 - [S] Predicted integral membrane protein
590
MJLIPHIC_01175 CDS COG0515 1269105 1270931 + [RTKL] Serine/threonine protein kinase
591
MJLIPHIC_01176 CDS NA 1270939 1271619 - NA
592
MJLIPHIC_01177 CDS NA 1271822 1271971 + NA
593
MJLIPHIC_01178 CDS COG0304 1272165 1273415 + [IQ] 3-oxoacyl-(acyl-carrier-protein) synthase
594
MJLIPHIC_01179 CDS COG2230 1273412 1274296 - [M] Cyclopropane fatty acid synthase and related
MJLIPHIC_01180 CDS COG2230 1274296 1275219 - [M] Cyclopropane fatty acid synthase and related
595
MJLIPHIC_01181 CDS NA 1275439 1275573 - NA
596
MJLIPHIC_01182 CDS COG0438 1276157 1277335 + [M] Glycosyltransferase
MJLIPHIC_01183 CDS NA 1277332 1278369 + NA
MJLIPHIC_01184 CDS COG2152 1278366 1279841 + [G] Predicted glycosylase
597
MJLIPHIC_01185 CDS COG1432 1279842 1280711 - [S] Uncharacterized conserved protein
598
MJLIPHIC_01186 CDS COG3239 1280838 1281929 + [I] Fatty acid desaturase
599
MJLIPHIC_01187 CDS ROG1869 1282273 1282482 + NA
600

MJLIPHIC_01188 CDS COG2124 1282581 1283942 + [Q] Cytochrome P450
MJLIPHIC_01189 CDS COG2133 1283935 1284996 + [G] Glucose/sorbosone dehydrogenases
MJLIPHIC_01190 CDS NA 1284938 1285705 + NA
601
MJLIPHIC_01191 CDS COG0683 1285707 1286996 - [E] ABC-type branched-chain amino acid transport systems,
MJLIPHIC_01192 CDS COG0410 1287035 1287748 - [E] ABC-type branched-chain amino acid transport systems,
MJLIPHIC_01193 CDS COG0411 1287741 1288457 - [E] ABC-type branched-chain amino acid transport systems,
MJLIPHIC_01194 CDS COG4177 1288454 1289461 - [E] ABC-type branched-chain amino acid transport system,
MJLIPHIC_01195 CDS COG0559 1289458 1290333 - [E] Branched-chain amino acid ABC-type transport system,
602
MJLIPHIC_01196 CDS ROG2639 1290581 1291009 + NA
603
MJLIPHIC_01197 CDS NA 1290996 1291175 - NA
MJLIPHIC_01198 CDS COG3385 1291202 1292839 - [L] FOG: Transposase and inactivated derivatives
604
MJLIPHIC_01199 CDS ROG3511 1293051 1293362 - NA
MJLIPHIC_01200 CDS NA 1293359 1293571 - NA
605
MJLIPHIC_01201 CDS NA 1293942 1294349 - NA
606
MJLIPHIC_01202 CDS ROG1662 1294590 1295882 - NA
607
MJLIPHIC_01203 CDS COG1262 1295995 1296870 + [S] Uncharacterized conserved protein
608
MJLIPHIC_01204 CDS COG2188 1296995 1297819 + [K] Transcriptional regulators
MJLIPHIC_01205 CDS COG3119 1297882 1299234 + [P] Arylsulfatase A and related enzymes
MJLIPHIC_01206 CDS COG0187 1299255 1301174 + [L] Type IIA topoisomerase (DNA gyrase/topo II,
609
MJLIPHIC_01207 CDS COG2149 1301175 1301498 - [S] Predicted membrane protein
MJLIPHIC_01208 CDS COG2149 1301495 1301875 - [S] Predicted membrane protein
610
MJLIPHIC_01209 CDS COG0715 1302007 1303464 + [P] ABC-type nitrate/sulfonate/bicarbonate transport systems, periplasmic
components
MJLIPHIC_01210 CDS COG0600 1303496 1304197 + [P] ABC-type nitrate/sulfonate/bicarbonate transport system, permease component
MJLIPHIC_01211 CDS COG0600 1304194 1304382 + [P] ABC-type nitrate/sulfonate/bicarbonate transport system, permease component
MJLIPHIC_01212 CDS COG1116 1304379 1305128 + [P] ABC-type nitrate/sulfonate/bicarbonate transport system, ATPase component
MJLIPHIC_01213 CDS COG0659 1305154 1306650 + [P] Sulfate permease and related transporters (MFS
611
MJLIPHIC_01214 CDS ROG1691 1306698 1307345 - NA
612
MJLIPHIC_01215 CDS NA 1307514 1307831 + NA
613
MJLIPHIC_01216 CDS COG1061 1308411 1310015 + [KL] DNA or RNA helicases of superfamily
MJLIPHIC_01217 CDS ROG1298 1310015 1310266 + NA
614
MJLIPHIC_01218 CDS COG3464 1310225 1311547 - [L] Transposase and inactivated derivatives
615
MJLIPHIC_01219 CDS NA 1312264 1313316 + NA
MJLIPHIC_01220 CDS NA 1313313 1314251 + NA
616
MJLIPHIC_01221 CDS COG1104 1314552 1315712 + [E] Cysteine sulfinate desulfinase/cysteine desulfurase and related
MJLIPHIC_01222 CDS ROG3642 1315709 1316782 + NA
MJLIPHIC_01223 CDS ROG3691 1316779 1320237 + NA
MJLIPHIC_01224 CDS ROG6454 1320230 1321078 + NA
617
MJLIPHIC_01225 CDS ROG0610 1321294 1322667 + NA
618
MJLIPHIC_01226 CDS NA 1322859 1323980 + NA
MJLIPHIC_01227 CDS ROG5395 1323992 1324906 + NA
619
MJLIPHIC_01228 CDS COG2801 1325180 1326046 - [L] Transposase and inactivated derivatives
MJLIPHIC_01229 CDS COG2963 1326043 1326339 - [L] Transposase and inactivated derivatives
620
MJLIPHIC_01230 CDS NA 1326988 1327353 + NA
621
MJLIPHIC_01231 CDS COG1112 1327543 1333908 + [L] Superfamily I DNA and RNA helicases
622
MJLIPHIC_01232 CDS NA 1334127 1334222 + NA
623
MJLIPHIC_01233 CDS ROG5019 1334462 1337860 + NA
MJLIPHIC_01234 CDS COG1205 1337844 1342847 + [R] Distinct helicase family with a unique
MJLIPHIC_01235 CDS NA 1342903 1343124 + NA
MJLIPHIC_01236 CDS ROG7225 1343125 1345338 + NA
MJLIPHIC_01237 CDS NA 1345348 1345767 + NA
624
MJLIPHIC_01238 CDS ROG0016 1346109 1346861 + NA
625
MJLIPHIC_01239 CDS COG3464 1346869 1347780 - [L] Transposase and inactivated derivatives
626
MJLIPHIC_01240 CDS NA 1348648 1350015 - NA
627
MJLIPHIC_01241 CDS NA 1350379 1351347 + NA
MJLIPHIC_01242 CDS NA 1351351 1351716 + NA
628
MJLIPHIC_01243 CDS NA 1352728 1353054 - NA
629
MJLIPHIC_01244 CDS ROG1490 1353370 1353978 + NA
630
MJLIPHIC_01245 CDS ROG0476 1354095 1355633 - NA
MJLIPHIC_01246 CDS COG0666 1355685 1356374 - [R] FOG: Ankyrin repeat
MJLIPHIC_01247 CDS COG1397 1356367 1357704 - [O] ADP-ribosylglycohydrolase
631
MJLIPHIC_01248 CDS COG1051 1357886 1358554 + [F] ADP-ribose pyrophosphatase
632
MJLIPHIC_01249 CDS NA 1358568 1359566 - NA
MJLIPHIC_01250 CDS ROG3248 1359598 1359942 - NA
633
MJLIPHIC_01251 CDS NA 1360010 1360372 - NA
MJLIPHIC_01252 CDS COG1397 1360369 1361847 - [O] ADP-ribosylglycohydrolase
634

	MJLIPHIC_01253	CDS	NA	1362036	1362377	-	NA
	MJLIPHIC_01254	CDS	COG3386	1362437	1363564	-	[G] Gluconolactonase
635							
	MJLIPHIC_01255	CDS	ROG5686	1363745	1364824	-	NA
636							
	MJLIPHIC_01256	CDS	COG4997	1364892	1365188	+	[S] Uncharacterized conserved protein
637							
	MJLIPHIC_01257	CDS	NA	1365336	1365533	+	NA
	MJLIPHIC_01258	CDS	COG3654	1365530	1365922	+	[R] Prophage maintenance system killer protein
638							
	MJLIPHIC_01259	CDS	ROG0431	1365999	1367429	+	NA
	MJLIPHIC_01260	CDS	ROG1817	1367426	1368148	+	NA
	MJLIPHIC_01261	CDS	COG4913	1368135	1371380	+	[S] Uncharacterized protein conserved in bacteria
	MJLIPHIC_01262	CDS	COG4924	1371386	1372546	+	[S] Uncharacterized protein conserved in bacteria
	MJLIPHIC_01263	CDS	COG4423	1372543	1372821	+	[S] Uncharacterized protein conserved in bacteria
	MJLIPHIC_01264	CDS	COG3742	1372821	1373210	+	[S] Uncharacterized protein conserved in bacteria
639							
	MJLIPHIC_01265	CDS	ROG1898	1373229	1375625	-	NA
	MJLIPHIC_01266	CDS	COG3119	1375656	1377416	-	[P] Arylsulfatase A and related enzymes
	MJLIPHIC_01267	CDS	ROG4322	1377413	1377850	-	NA
640							
	MJLIPHIC_01268	CDS	ROG7225	1378017	1379666	+	NA
641							
	MJLIPHIC_01269	CDS	NA	1379677	1380480	-	NA
642							
	MJLIPHIC_01270	CDS	COG0129	1380557	1382425	+	[EG] Dihydroxyacid dehydratase/phosphogluconate dehydratase
643							
	MJLIPHIC_01271	CDS	COG1529	1382428	1385109	-	[C] Aerobic-type carbon monoxide dehydrogenase, large subunit
	MJLIPHIC_01272	CDS	COG1319	1385106	1385978	-	[C] Aerobic-type carbon monoxide dehydrogenase, middle subunit
644							
	MJLIPHIC_01273	CDS	COG2233	1386036	1387940	+	[F] Xanthine/uracil permeases
	MJLIPHIC_01274	CDS	COG3195	1387942	1388448	+	[S] Uncharacterized protein conserved in bacteria
	MJLIPHIC_01275	CDS	COG2351	1388445	1388762	+	[R] Transthyretin-like protein
	MJLIPHIC_01276	CDS	COG3648	1388764	1389663	+	[Q] Uricase (urate oxidase)
	MJLIPHIC_01277	CDS	COG0402	1389680	1391038	+	[FR] Cytosine deaminase and related metal-dependent hydrolases
	MJLIPHIC_01278	CDS	COG0402	1391035	1392369	+	[FR] Cytosine deaminase and related metal-dependent hydrolases
645							
	MJLIPHIC_01279	CDS	COG3491	1392514	1393572	+	[R] Isopenicillin N synthase and related dioxygenases
646							
	MJLIPHIC_01280	CDS	NA	1393703	1394095	+	NA
647							
	MJLIPHIC_01281	CDS	COG2771	1394209	1394544	+	[K] DNA-binding HTH domain-containing proteins
648							
	MJLIPHIC_01282	CDS	ROG2935	1394787	1394954	+	NA
649							
	MJLIPHIC_01283	CDS	COG1396	1395326	1396159	-	[K] Predicted transcriptional regulators
650							
	MJLIPHIC_01284	CDS	COG0667	1396275	1397318	+	[C] Predicted oxidoreductases (related to aryl-alcohol dehydrogenases)
651							
	MJLIPHIC_01285	CDS	COG1309	1397328	1397936	-	[K] Transcriptional regulator
	MJLIPHIC_01286	CDS	COG1012	1398000	1399523	-	[C] NAD-dependent aldehyde dehydrogenases
	MJLIPHIC_01287	CDS	ROG0920	1399532	1399834	-	NA
	MJLIPHIC_01288	CDS	COG2936	1399868	1401571	-	[R] Predicted acyl esterases
652							
	MJLIPHIC_01289	CDS	ROG8500	1402450	1403853	+	NA
653							
	MJLIPHIC_01290	CDS	COG1297	1404005	1405996	-	[S] Predicted membrane protein
654							
	MJLIPHIC_01291	CDS	ROG1035	1406153	1406749	+	NA
655							
	MJLIPHIC_01292	CDS	ROG0772	1406859	1409201	+	NA
	MJLIPHIC_01293	CDS	COG0668	1409246	1410160	+	[M] Small-conductance mechanosensitive channel
656							
	MJLIPHIC_01294	CDS	ROG1709	1410157	1410906	-	NA
657							
	MJLIPHIC_01295	CDS	COG4108	1411176	1412792	-	[J] Peptide chain release factor RF-3
658							
	MJLIPHIC_01296	CDS	COG1802	1413062	1413751	-	[K] Transcriptional regulators
	MJLIPHIC_01297	CDS	COG0726	1413803	1414714	-	[G] Predicted xylanase/chitin deacetylase
	MJLIPHIC_01298	CDS	COG3473	1414728	1415882	-	[Q] Maleate cis-trans isomerase
	MJLIPHIC_01299	CDS	COG2271	1415879	1417180	-	[G] Sugar phosphate permease
659							
	MJLIPHIC_01300	CDS	COG0179	1417303	1417941	+	[Q] 2-keto-4-pentenoate hydratase/2-oxohepta-3-ene-1,7-dioic acid hydratase (catechol pathway)
660							
	MJLIPHIC_01301	CDS	NA	1418118	1419377	+	NA
661							
	MJLIPHIC_01302	CDS	NA	1419564	1419878	+	NA
662							
	MJLIPHIC_01303	CDS	NA	1420278	1420427	+	NA
663							
	MJLIPHIC_01304	CDS	COG0507	1420424	1422088	-	[L] ATP-dependent exoDNAse (exonuclease V), alpha subunit
	MJLIPHIC_01305	CDS	COG1074	1422085	1425336	-	[L] ATP-dependent exoDNAse (exonuclease V) beta subunit
	MJLIPHIC_01306	CDS	COG1330	1425333	1428590	-	[L] Exonuclease V gamma subunit
	MJLIPHIC_01307	CDS	NA	1428614	1429858	-	NA
	MJLIPHIC_01308	CDS	COG1846	1429855	1430331	-	[K] Transcriptional regulators
	MJLIPHIC_01309	CDS	COG1024	1430426	1431133	-	[I] Enoyl-CoA hydratase/carnithine racemase
	MJLIPHIC_01310	CDS	ROG7305	1431151	1432008	-	NA
	MJLIPHIC_01311	CDS	COG3673	1432067	1433059	-	[S] Uncharacterized conserved protein
664							
	MJLIPHIC_01312	CDS	COG0491	1433302	1434039	-	[R] Zn-dependent hydrolases, including glyoxylases
665							
	MJLIPHIC_01313	CDS	COG1959	1434167	1434628	+	[K] Predicted transcriptional regulator
	MJLIPHIC_01314	CDS	COG1017	1434628	1435863	+	[C] Hemoglobin-like flavoprotein
666							
	MJLIPHIC_01315	tRNA	NA	1435983	1436056	+	NA
	MJLIPHIC_01316	tRNA	NA	1436094	1436168	+	NA
	MJLIPHIC_01317	CDS	COG0267	1436204	1436371	+	[J] Ribosomal protein L33
	MJLIPHIC_01318	CDS	ROG0252	1436409	1436858	+	NA
	MJLIPHIC_01319	CDS	ROG2306	1436845	1437273	+	NA

	MJLIPHIC_01320	CDS	ROG0252	1437283	1437792	+	NA
667							
	MJLIPHIC_01321	tRNA	NA	1437965	1438038	+	NA
668							
	MJLIPHIC_01322	CDS	COG0690	1438122	1438556	+	[U] Preprotein translocase subunit SecE
	MJLIPHIC_01323	CDS	COG0250	1438583	1439425	+	[K] Transcription antiterminator
	MJLIPHIC_01324	CDS	COG0080	1439493	1439921	+	[J] Ribosomal protein L11
	MJLIPHIC_01325	CDS	COG0081	1439987	1440694	+	[J] Ribosomal protein L1
669							
	MJLIPHIC_01326	CDS	COG4941	1440768	1441997	-	[K] Predicted RNA polymerase sigma factor containing
	MJLIPHIC_01327	CDS	COG3795	1442005	1442700	-	[S] Uncharacterized protein conserved in bacteria
670							
	MJLIPHIC_01328	CDS	COG2230	1442825	1443685	-	[M] Cyclopropane fatty acid synthase and related
	MJLIPHIC_01329	CDS	COG2230	1443878	1444789	-	[M] Cyclopropane fatty acid synthase and related
671							
	MJLIPHIC_01330	CDS	COG0596	1444930	1445847	-	[R] Predicted hydrolases or acyltransferases (alpha/beta hydrolase
	MJLIPHIC_01331	CDS	COG0661	1445847	1447268	-	[R] Predicted unusual protein kinase
672							
	MJLIPHIC_01332	CDS	COG3662	1447333	1447941	-	[S] Uncharacterized protein conserved in bacteria
673							
	MJLIPHIC_01333	CDS	COG1309	1448423	1449022	+	[K] Transcriptional regulator
674							
	MJLIPHIC_01334	CDS	ROG0833	1449027	1449638	-	NA
	MJLIPHIC_01335	CDS	ROG5245	1449643	1450170	-	NA
675							
	MJLIPHIC_01336	CDS	COG2378	1450233	1451195	+	[K] Predicted transcriptional regulator
676							
	MJLIPHIC_01337	CDS	ROG0895	1451188	1451748	-	NA
677							
	MJLIPHIC_01338	CDS	COG3568	1451803	1452558	+	[R] Metal-dependent hydrolase
	MJLIPHIC_01339	CDS	COG0383	1452576	1456718	+	[G] Alpha-mannosidase
	MJLIPHIC_01340	CDS	NA	1456715	1457314	+	NA
	MJLIPHIC_01341	CDS	COG1940	1457319	1458224	+	[KG] Transcriptional regulator/sugar kinase
678							
	MJLIPHIC_01342	CDS	COG0244	1458668	1459195	+	[J] Ribosomal protein L10
	MJLIPHIC_01343	CDS	COG0222	1459265	1459657	+	[J] Ribosomal protein L7/L12
679							
	MJLIPHIC_01344	CDS	COG1127	1459848	1460930	+	[Q] ABC-type transport system involved in resistance
680							
	MJLIPHIC_01345	CDS	COG0085	1461648	1465139	+	[K] DNA-directed RNA polymerase, beta subunit/140 kD
	MJLIPHIC_01346	CDS	COG0086	1465203	1469156	+	[K] DNA-directed RNA polymerase, beta' subunit/160 kD
681							
	MJLIPHIC_01347	CDS	COG1609	1469221	1470270	-	[K] Transcriptional regulators
682							
	MJLIPHIC_01348	CDS	COG1063	1470431	1471456	+	[ER] Threonine dehydrogenase and related Zn-dependent dehydrogenases
	MJLIPHIC_01349	CDS	COG1482	1471453	1472454	+	[G] Phosphomannose isomerase
	MJLIPHIC_01350	CDS	COG1129	1472465	1473223	+	[G] ABC-type sugar transport system, ATPase component
	MJLIPHIC_01351	CDS	COG4158	1473220	1474338	+	[R] Predicted ABC-type sugar transport system, permease
	MJLIPHIC_01352	CDS	COG1879	1474328	1475374	+	[G] ABC-type sugar transport system, periplasmic component
683							
	MJLIPHIC_01353	CDS	COG0191	1475387	1476229	-	[G] Fructose/tagatose bisphosphate aldolase
684							
	MJLIPHIC_01354	CDS	COG1070	1476315	1477562	+	[G] Sugar (pentulose and hexulose) kinases
	MJLIPHIC_01355	CDS	COG1349	1477559	1478347	+	[KG] Transcriptional regulators of sugar metabolism
	MJLIPHIC_01356	CDS	COG1105	1478344	1479261	+	[G] Fructose-1-phosphate kinase and related fructose-6-phosphate kinase
	MJLIPHIC_01357	CDS	COG0579	1479288	1480475	+	[R] Predicted dehydrogenase
685							
	MJLIPHIC_01358	CDS	COG1309	1480902	1481606	-	[K] Transcriptional regulator
686							
	MJLIPHIC_01359	CDS	NA	1481776	1482201	+	NA
	MJLIPHIC_01360	CDS	COG2409	1482198	1485101	+	[R] Predicted drug exporters of the RND
	MJLIPHIC_01361	CDS	COG0648	1485148	1485900	+	[L] Endonuclease IV
687							
	MJLIPHIC_01362	CDS	ROG3410	1486024	1487205	+	NA
688							
	MJLIPHIC_01363	CDS	COG3450	1487074	1487409	-	[R] Predicted enzyme of the cupin superfamily
689							
	MJLIPHIC_01364	CDS	NA	1487534	1488700	+	NA
	MJLIPHIC_01365	CDS	COG1960	1488754	1490409	+	[I] Acyl-CoA dehydrogenases
	MJLIPHIC_01366	CDS	COG0447	1490456	1491394	+	[H] Dihydroxynaphthoic acid synthase
	MJLIPHIC_01367	CDS	COG3327	1491407	1492138	+	[K] Phenylacetic acid-responsive transcriptional repressor
	MJLIPHIC_01368	CDS	COG1024	1492135	1492899	+	[I] Enoyl-CoA hydratase/carnithine racemase
690							
	MJLIPHIC_01369	CDS	COG0583	1492993	1493892	-	[K] Transcriptional regulator
691							
	MJLIPHIC_01370	CDS	COG1454	1494011	1495168	+	[C] Alcohol dehydrogenase, class IV
692							
	MJLIPHIC_01371	CDS	ROG4412	1495173	1495583	-	NA
	MJLIPHIC_01372	CDS	NA	1495614	1495991	-	NA
693							
	MJLIPHIC_01373	CDS	ROG6760	1496220	1496753	+	NA
	MJLIPHIC_01374	CDS	ROG7479	1496756	1497277	+	NA
694							
	MJLIPHIC_01375	CDS	ROG6516	1497398	1498018	+	NA
695							
	MJLIPHIC_01376	CDS	COG0048	1498302	1498676	+	[J] Ribosomal protein S12
	MJLIPHIC_01377	CDS	COG0049	1498676	1499146	+	[J] Ribosomal protein S7
	MJLIPHIC_01378	CDS	COG0480	1499500	1501323	+	[J] Translation elongation factors (GTPases)
	MJLIPHIC_01379	CDS	COG0050	1501413	1502603	+	[J] GTPases - translation elongation factors
696							
	MJLIPHIC_01380	CDS	NA	1502841	1503758	+	NA
697							
	MJLIPHIC_01381	CDS	ROG5614	1503836	1504765	+	NA
698							
	MJLIPHIC_01382	CDS	COG3464	1505341	1506252	+	[L] Transposase and inactivated derivatives
699							
	MJLIPHIC_01383	CDS	ROG0016	1506260	1507012	-	NA
700							
	MJLIPHIC_01384	CDS	ROG3910	1507065	1507916	+	NA
701							

	MJLIPHIC_01385	CDS	COG1757	1507983	1508894	+	[C] Na+/H+ antiporter
	MJLIPHIC_01386	CDS	COG1757	1508891	1509466	+	[C] Na+/H+ antiporter
	MJLIPHIC_01387	CDS	COG0531	1509466	1510161	+	[E] Amino acid transporters
	MJLIPHIC_01388	CDS	COG0531	1510331	1510909	+	[E] Amino acid transporters
	MJLIPHIC_01389	CDS	COG2235	1510936	1512213	+	[E] Arginine deiminase
	MJLIPHIC_01390	CDS	COG1028	1512243	1513094	+	[IQR] Dehydrogenases with different specificities (related to
702							
	MJLIPHIC_01391	CDS	COG0589	1513135	1514016	-	[T] Universal stress protein UspA and related
	MJLIPHIC_01392	CDS	COG0531	1514075	1515580	-	[E] Amino acid transporters
	MJLIPHIC_01393	CDS	COG4992	1515608	1516876	-	[E] Ornithine/acetylornithine aminotransferase
	MJLIPHIC_01394	CDS	COG1834	1516873	1517760	-	[E] N-Dimethylarginine dimethylaminohydrolase
703							
	MJLIPHIC_01395	CDS	COG1522	1517874	1518320	+	[K] Transcriptional regulators
	MJLIPHIC_01396	CDS	COG0446	1518420	1519577	+	[R] Uncharacterized NAD(FAD)-dependent dehydrogenases
704							
	MJLIPHIC_01397	CDS	COG2764	1519642	1520925	-	[S] Uncharacterized protein conserved in bacteria
	MJLIPHIC_01398	CDS	COG1595	1520922	1521386	-	[K] DNA-directed RNA polymerase specialized sigma subunit,
705							
	MJLIPHIC_01399	CDS	ROG3669	1521559	1522269	+	NA
706							
	MJLIPHIC_01400	CDS	COG1309	1522270	1522872	-	[K] Transcriptional regulator
707							
	MJLIPHIC_01401	CDS	NA	1522968	1523063	+	NA
	MJLIPHIC_01402	CDS	ROG4802	1523066	1523374	+	NA
	MJLIPHIC_01403	CDS	COG0535	1523371	1524549	+	[R] Predicted Fe-S oxidoreductases
	MJLIPHIC_01404	CDS	COG1304	1524552	1525745	+	[C] L-lactate dehydrogenase (FMN-dependent) and related alpha-hydroxy
708							
	MJLIPHIC_01405	CDS	COG1402	1525942	1526610	+	[R] Uncharacterized protein, putative amidase
	MJLIPHIC_01406	CDS	COG1216	1526607	1528019	+	[R] Predicted glycosyltransferases
	MJLIPHIC_01407	CDS	COG2303	1528038	1529444	+	[E] Choline dehydrogenase and related flavoproteins
709							
	MJLIPHIC_01408	CDS	NA	1529431	1531005	-	NA
710							
	MJLIPHIC_01409	CDS	COG1309	1531094	1531729	+	[K] Transcriptional regulator
711							
	MJLIPHIC_01410	CDS	COG2124	1531695	1532945	-	[Q] Cytochrome P450
	MJLIPHIC_01411	CDS	COG2124	1533049	1534299	-	[Q] Cytochrome P450
712							
	MJLIPHIC_01412	CDS	COG2272	1534440	1536026	+	[I] Carboxylesterase type B
	MJLIPHIC_01413	CDS	ROG1597	1536028	1536393	+	NA
713							
	MJLIPHIC_01414	CDS	ROG7874	1536329	1536862	-	NA
714							
	MJLIPHIC_01415	CDS	COG0051	1537276	1537581	+	[J] Ribosomal protein S10
	MJLIPHIC_01416	CDS	COG0087	1537596	1538249	+	[J] Ribosomal protein L3
	MJLIPHIC_01417	CDS	COG0088	1538249	1538896	+	[J] Ribosomal protein L4
	MJLIPHIC_01418	CDS	COG0089	1538896	1539198	+	[J] Ribosomal protein L23
	MJLIPHIC_01419	CDS	COG0090	1539223	1540059	+	[J] Ribosomal protein L2
	MJLIPHIC_01420	CDS	COG0185	1540081	1540362	+	[J] Ribosomal protein S19
	MJLIPHIC_01421	CDS	COG0091	1540359	1540820	+	[J] Ribosomal protein L22
	MJLIPHIC_01422	CDS	COG0092	1540820	1541647	+	[J] Ribosomal protein S3
	MJLIPHIC_01423	CDS	COG0197	1541650	1542066	+	[J] Ribosomal protein L16/L10E
	MJLIPHIC_01424	CDS	COG0255	1542066	1542299	+	[J] Ribosomal protein L29
	MJLIPHIC_01425	CDS	COG0186	1542299	1542595	+	[J] Ribosomal protein S17
715							
	MJLIPHIC_01426	CDS	COG0494	1542699	1543220	+	[LR] NTP pyrophosphohydrolases including oxidative damage repair
716							
	MJLIPHIC_01427	CDS	COG0789	1543239	1543640	-	[K] Predicted transcriptional regulators
	MJLIPHIC_01428	CDS	COG0659	1543640	1545139	-	[P] Sulfate permease and related transporters (MFS
717							
	MJLIPHIC_01429	CDS	COG2335	1545377	1545529	-	[M] Secreted and surface protein containing fasciclin-like
	MJLIPHIC_01430	CDS	COG2335	1545477	1545797	-	[M] Secreted and surface protein containing fasciclin-like
718							
	MJLIPHIC_01431	CDS	COG3119	1546230	1548581	+	[P] Arylsulfatase A and related enzymes
	MJLIPHIC_01432	CDS	COG1262	1548624	1549514	+	[S] Uncharacterized conserved protein
719							
	MJLIPHIC_01433	CDS	ROG0358	1549538	1551166	-	NA
720							
	MJLIPHIC_01434	CDS	COG0477	1551538	1552914	+	[GEPR] Permeases of the major facilitator superfamily
721							
	MJLIPHIC_01435	CDS	COG2508	1552915	1554600	-	[TQ] Regulator of polyketide synthase expression
722							
	MJLIPHIC_01436	CDS	COG3193	1554800	1555246	+	[R] Uncharacterized protein, possibly involved in utilization
723							
	MJLIPHIC_01437	CDS	COG5424	1555365	1556084	+	[H] Pyrroloquinoline quinone (Coenzyme PQQ) biosynthesis protein
	MJLIPHIC_01438	CDS	COG2146	1556130	1556480	+	[PR] Ferredoxin subunits of nitrite reductase and
	MJLIPHIC_01439	CDS	COG0446	1556477	1557703	+	[R] Uncharacterized NAD(FAD)-dependent dehydrogenases
	MJLIPHIC_01440	CDS	COG1012	1557700	1559142	+	[C] NAD-dependent aldehyde dehydrogenases
	MJLIPHIC_01441	CDS	COG2146	1559157	1559510	+	[PR] Ferredoxin subunits of nitrite reductase and
	MJLIPHIC_01442	CDS	COG0405	1559516	1561111	+	[E] Gamma-glutamyltransferase
	MJLIPHIC_01443	CDS	NA	1561101	1561595	+	NA
724							
	MJLIPHIC_01444	CDS	COG0093	1561939	1562307	+	[J] Ribosomal protein L14
	MJLIPHIC_01445	CDS	COG0198	1562308	1562625	+	[J] Ribosomal protein L24
	MJLIPHIC_01446	CDS	COG0094	1562625	1563188	+	[J] Ribosomal protein L5
	MJLIPHIC_01447	CDS	COG0199	1563192	1563377	+	[J] Ribosomal protein S14
	MJLIPHIC_01448	CDS	COG0096	1563476	1563874	+	[J] Ribosomal protein S8
	MJLIPHIC_01449	CDS	COG0097	1563889	1564428	+	[J] Ribosomal protein L6P/L9E
	MJLIPHIC_01450	CDS	COG0256	1564432	1564839	+	[J] Ribosomal protein L18
	MJLIPHIC_01451	CDS	COG0098	1564865	1565509	+	[J] Ribosomal protein S5
	MJLIPHIC_01452	CDS	COG1841	1565512	1565697	+	[J] Ribosomal protein L30/L7E
	MJLIPHIC_01453	CDS	COG0200	1565694	1566137	+	[J] Ribosomal protein L15
725							
	MJLIPHIC_01454	CDS	COG2141	1566207	1567187	-	[C] Coenzyme F420-dependent N5,N10-methylene tetrahydromethanopterin reductase and
726							
	MJLIPHIC_01455	CDS	COG0616	1567306	1569090	+	[OU] Periplasmic serine proteases (ClpP class)
727							
	MJLIPHIC_01456	CDS	COG0477	1569316	1570743	+	[GEPR] Permeases of the major facilitator superfamily
	MJLIPHIC_01457	CDS	COG1280	1570776	1571426	+	[E] Putative threonine efflux protein
728							

	MJLIPHIC_01458	CDS	COG3315	1571431	1572342	-	[Q] O-Methyltransferase involved in polyketide biosynthesis
	MJLIPHIC_01459	CDS	COG3315	1572351	1573247	-	[Q] O-Methyltransferase involved in polyketide biosynthesis
	MJLIPHIC_01460	CDS	COG3315	1573257	1574171	-	[Q] O-Methyltransferase involved in polyketide biosynthesis
	MJLIPHIC_01461	CDS	COG3315	1574178	1575092	-	[Q] O-Methyltransferase involved in polyketide biosynthesis
729							
	MJLIPHIC_01462	CDS	COG0201	1575442	1576767	+	[U] Preprotein translocase subunit SecY
	MJLIPHIC_01463	CDS	COG0563	1576764	1577309	+	[F] Adenylate kinase and related kinases
	MJLIPHIC_01464	CDS	COG0024	1577329	1578126	+	[J] Methionine aminopeptidase
730							
	MJLIPHIC_01465	CDS	COG1595	1578235	1578777	+	[K] DNA-directed RNA polymerase specialized sigma subunit,
	MJLIPHIC_01466	CDS	COG5662	1578774	1579505	+	[K] Predicted transmembrane transcriptional regulator (anti-sigma factor)
731							
	MJLIPHIC_01467	CDS	COG3960	1579660	1581408	+	[R] Glyoxylate carboligase
	MJLIPHIC_01468	CDS	ROG6273	1581389	1582069	+	NA
	MJLIPHIC_01469	CDS	COG0332	1582078	1583100	+	[I] 3-oxoacyl-[acyl-carrier-protein] synthase III
	MJLIPHIC_01470	CDS	COG0079	1583100	1584137	+	[E] Histidinol-phosphate/aromatic aminotransferase and cobyric acid decarboxylase
	MJLIPHIC_01471	CDS	COG1846	1584173	1584694	+	[K] Transcriptional regulators
732							
	MJLIPHIC_01472	CDS	COG4585	1584746	1585987	-	[T] Signal transduction histidine kinase
	MJLIPHIC_01473	CDS	COG2197	1585984	1586646	-	[TK] Response regulator containing a CheY-like receiver
733							
	MJLIPHIC_01474	CDS	COG0637	1586745	1587569	+	[R] Predicted phosphatase/phosphohexomutase
734							
	MJLIPHIC_01475	CDS	COG2084	1587649	1588521	-	[I] 3-hydroxyisobutyrate dehydrogenase and related beta-hydroxyacid dehydrogenases
	MJLIPHIC_01476	CDS	COG1960	1588538	1589725	-	[I] Acyl-CoA dehydrogenases
	MJLIPHIC_01477	CDS	COG1012	1589732	1591252	-	[C] NAD-dependent aldehyde dehydrogenases
735							
	MJLIPHIC_01478	CDS	COG0654	1591424	1592929	+	[HC] 2-polyprenyl-6-methoxyphenol hydroxylase and related FAD-dependent oxidoreductases
	MJLIPHIC_01479	CDS	COG1309	1592926	1593525	+	[K] Transcriptional regulator
736							
	MJLIPHIC_01480	CDS	COG3315	1593607	1594473	+	[Q] O-Methyltransferase involved in polyketide biosynthesis
737							
	MJLIPHIC_01481	CDS	COG1131	1594527	1595402	+	[V] ABC-type multidrug transport system, ATPase component
	MJLIPHIC_01482	CDS	ROG3613	1595399	1596112	+	NA
	MJLIPHIC_01483	CDS	ROG2436	1596109	1596867	+	NA
738							
	MJLIPHIC_01484	CDS	COG5006	1596886	1597764	-	[R] Predicted permease, DMT superfamily
	MJLIPHIC_01485	CDS	COG1309	1597761	1598429	-	[K] Transcriptional regulator
739							
	MJLIPHIC_01486	CDS	COG3191	1598476	1599585	+	[EQ] L-aminopeptidase/D-esterase
	MJLIPHIC_01487	CDS	COG0531	1599585	1601009	+	[E] Amino acid transporters
740							
	MJLIPHIC_01488	CDS	COG1309	1601016	1601612	-	[K] Transcriptional regulator
741							
	MJLIPHIC_01489	CDS	COG0778	1601665	1602363	+	[C] Nitroreductase
742							
	MJLIPHIC_01490	CDS	COG1898	1602360	1602965	-	[M] dTDP-4-dehydrorhamnose 3,5-epimerase and related enzymes
	MJLIPHIC_01491	CDS	COG1088	1602962	1603957	-	[M] dTDP-D-glucose 4,6-dehydratase
	MJLIPHIC_01492	CDS	NA	1603984	1604835	-	NA
	MJLIPHIC_01493	CDS	COG0778	1604864	1605718	-	[C] Nitroreductase
743							
	MJLIPHIC_01494	CDS	COG3852	1605862	1607334	-	[T] Signal transduction histidine kinase, nitrogen specific
	MJLIPHIC_01495	CDS	COG0492	1607344	1609035	-	[O] Thioredoxin reductase
744							
	MJLIPHIC_01496	CDS	COG2268	1609139	1610719	-	[S] Uncharacterized protein conserved in bacteria
	MJLIPHIC_01497	CDS	ROG2851	1610744	1611241	-	NA
745							
	MJLIPHIC_01498	CDS	COG0361	1611597	1611818	+	[J] Translation initiation factor 1 (IF-1)
	MJLIPHIC_01499	CDS	COG0099	1612201	1612575	+	[J] Ribosomal protein S13
	MJLIPHIC_01500	CDS	COG0100	1612581	1612997	+	[J] Ribosomal protein S11
	MJLIPHIC_01501	CDS	COG0522	1613023	1613628	+	[J] Ribosomal protein S4 and related proteins
	MJLIPHIC_01502	CDS	COG0202	1613721	1614773	+	[K] DNA-directed RNA polymerase, alpha subunit/40 kD
	MJLIPHIC_01503	CDS	COG0203	1614848	1615447	+	[J] Ribosomal protein L17
	MJLIPHIC_01504	CDS	COG0101	1615467	1616342	+	[J] Pseudouridylate synthase
746							
	MJLIPHIC_01505	CDS	ROG5614	1616339	1617031	-	NA
	MJLIPHIC_01506	CDS	ROG5614	1617095	1617703	-	NA
	MJLIPHIC_01507	CDS	ROG5614	1617806	1618477	-	NA
	MJLIPHIC_01508	CDS	COG0697	1618493	1619572	-	[GER] Permeases of the drug/metabolite transporter (DMT)
747							
	MJLIPHIC_01509	CDS	ROG4269	1619643	1620203	+	NA
748							
	MJLIPHIC_01510	CDS	COG1359	1620225	1620545	-	[S] Uncharacterized conserved protein
749							
	MJLIPHIC_01511	CDS	ROG3025	1620683	1622059	+	NA
750							
	MJLIPHIC_01512	CDS	COG1404	1622024	1623349	-	[O] Subtilisin-like serine proteases
	MJLIPHIC_01513	CDS	ROG3360	1623349	1624659	-	NA
751							
	MJLIPHIC_01514	CDS	COG1674	1624781	1628416	+	[D] DNA segregation ATPase FtsK/SpoIIIE and related
	MJLIPHIC_01515	CDS	NA	1628401	1629507	+	NA
752							
	MJLIPHIC_01516	CDS	COG4842	1629580	1629891	+	[S] Uncharacterized protein conserved in bacteria
	MJLIPHIC_01517	CDS	COG4842	1629917	1630213	+	[S] Uncharacterized protein conserved in bacteria
753							
	MJLIPHIC_01518	CDS	COG0513	1630283	1631665	-	[LKJ] Superfamily II DNA and RNA helicases
754							
	MJLIPHIC_01519	CDS	COG3284	1631921	1633222	+	[QK] Transcriptional activator of acetoin/glycerol metabolism
	MJLIPHIC_01520	CDS	COG1012	1633335	1634858	+	[C] NAD-dependent aldehyde dehydrogenases
	MJLIPHIC_01521	CDS	COG3193	1635013	1635411	+	[R] Uncharacterized protein, possibly involved in utilization
	MJLIPHIC_01522	tRNA	NA	1635470	1635560	+	NA
755							
	MJLIPHIC_01523	CDS	COG4910	1636280	1636708	+	[Q] Propanediol dehydratase, small subunit
	MJLIPHIC_01524	CDS	COG4909	1636718	1638430	+	[Q] Propanediol dehydratase, large subunit
	MJLIPHIC_01525	CDS	ROG6636	1638433	1639029	+	NA
	MJLIPHIC_01526	CDS	ROG4508	1639029	1639436	+	NA
	MJLIPHIC_01527	CDS	ROG4508	1639433	1640899	+	NA
	MJLIPHIC_01528	CDS	ROG6636	1640922	1641263	+	NA
756							

	MJLIPHIC_01529	CDS	COG0531	1641497	1642909	+	[E] Amino acid transporters
	MJLIPHIC_01530	CDS	COG4303	1642906	1644321	+	[E] Ethanolamine ammonia-lyase, large subunit
	MJLIPHIC_01531	CDS	COG4302	1644318	1645094	+	[E] Ethanolamine ammonia-lyase, small subunit
757							
	MJLIPHIC_01532	CDS	ROG3094	1645169	1645585	-	NA
758							
	MJLIPHIC_01533	CDS	COG0102	1646296	1646739	+	[J] Ribosomal protein L13
	MJLIPHIC_01534	CDS	COG0103	1646736	1647188	+	[J] Ribosomal protein S9
759							
	MJLIPHIC_01535	CDS	NA	1647536	1648012	+	NA
	MJLIPHIC_01536	CDS	COG1109	1648071	1649525	+	[G] Phosphomannomutase
	MJLIPHIC_01537	CDS	NA	1649573	1649881	+	NA
760							
	MJLIPHIC_01538	CDS	NA	1650129	1651289	+	NA
761							
	MJLIPHIC_01539	CDS	COG0583	1651286	1652176	-	[K] Transcriptional regulator
762							
	MJLIPHIC_01540	CDS	COG1028	1652248	1652964	+	[IQR] Dehydrogenases with different specificities (related to
763							
	MJLIPHIC_01541	CDS	COG3670	1652977	1654452	-	[Q] Lignostilbene-alpha,beta-dioxygenase and related enzymes
764							
	MJLIPHIC_01542	CDS	COG1309	1654752	1655318	+	[K] Transcriptional regulator
765							
	MJLIPHIC_01543	CDS	NA	1655346	1656386	-	NA
	MJLIPHIC_01544	CDS	ROG2202	1656437	1657288	-	NA
766							
	MJLIPHIC_01545	CDS	COG0449	1657467	1659353	+	[M] Glucosamine 6-phosphate synthetase, contains amidotransferase and
	MJLIPHIC_01546	CDS	COG0596	1659357	1660295	+	[R] Predicted hydrolases or acyltransferases (alpha/beta hydrolase
767							
	MJLIPHIC_01547	CDS	COG0697	1660346	1661290	-	[GER] Permeases of the drug/metabolite transporter (DMT)
768							
	MJLIPHIC_01548	CDS	COG1167	1661348	1662760	+	[KE] Transcriptional regulators containing a DNA-binding HTH
769							
	MJLIPHIC_01549	CDS	COG0063	1662840	1664252	+	[G] Predicted sugar kinase
	MJLIPHIC_01550	CDS	COG0076	1664273	1665652	+	[E] Glutamate decarboxylase and related PLP-dependent proteins
	MJLIPHIC_01551	CDS	COG0787	1665708	1666877	+	[M] Alanine racemase
	MJLIPHIC_01552	CDS	COG0596	1666874	1667959	+	[R] Predicted hydrolases or acyltransferases (alpha/beta hydrolase
	MJLIPHIC_01553	CDS	COG0802	1667952	1668419	+	[R] Predicted ATPase or kinase
	MJLIPHIC_01554	CDS	COG1214	1668416	1669060	+	[O] Inactive homolog of metal-dependent proteases, putative
	MJLIPHIC_01555	CDS	COG0456	1669057	1669533	+	[R] Acetyltransferases
	MJLIPHIC_01556	CDS	COG0533	1669530	1670552	+	[O] Metal-dependent proteases with possible chaperone activity
	MJLIPHIC_01557	CDS	ROG2444	1670549	1670998	+	NA
770							
	MJLIPHIC_01558	CDS	COG0234	1671178	1671480	+	[O] Co-chaperonin GroES (HSP10)
	MJLIPHIC_01559	CDS	COG0459	1671564	1673186	+	[O] Chaperonin GroEL (HSP60 family)
771							
	MJLIPHIC_01560	CDS	COG1396	1673706	1674266	+	[K] Predicted transcriptional regulators
772							
	MJLIPHIC_01561	CDS	COG1680	1674284	1675408	-	[V] Beta-lactamase class C and other penicillin
773							
	MJLIPHIC_01562	CDS	NA	1675484	1675864	+	NA
774							
	MJLIPHIC_01563	CDS	COG0596	1675840	1676619	-	[R] Predicted hydrolases or acyltransferases (alpha/beta hydrolase
775							
	MJLIPHIC_01564	CDS	NA	1676817	1678142	-	NA
776							
	MJLIPHIC_01565	CDS	ROG8500	1678351	1679754	+	NA
777							
	MJLIPHIC_01566	CDS	NA	1680305	1681108	+	NA
	MJLIPHIC_01567	CDS	COG1024	1681151	1681948	+	[I] Enoyl-CoA hydratase/carnithine racemase
778							
	MJLIPHIC_01568	CDS	COG0300	1682146	1682877	-	[R] Short-chain dehydrogenases of various substrate specificities
779							
	MJLIPHIC_01569	CDS	COG1309	1682974	1683543	+	[K] Transcriptional regulator
780							
	MJLIPHIC_01570	CDS	ROG1090	1683664	1683954	-	NA
781							
	MJLIPHIC_01571	CDS	ROG5319	1684388	1685194	+	NA
782							
	MJLIPHIC_01572	CDS	COG1595	1685464	1685862	+	[K] DNA-directed RNA polymerase specialized sigma subunit,
	MJLIPHIC_01573	CDS	ROG5191	1685855	1687054	+	NA
783							
	MJLIPHIC_01574	CDS	ROG1396	1687085	1687495	-	NA
784							
	MJLIPHIC_01575	CDS	COG0516	1687637	1689178	+	[F] IMP dehydrogenase/GMP reductase
	MJLIPHIC_01576	CDS	COG0516	1689198	1690334	+	[F] IMP dehydrogenase/GMP reductase
785							
	MJLIPHIC_01577	CDS	COG0665	1690403	1692139	+	[E] Glycine/D-amino acid oxidases (deaminating)
786							
	MJLIPHIC_01578	CDS	COG0704	1692222	1692881	+	[P] Phosphate uptake regulator
787							
	MJLIPHIC_01579	CDS	COG3960	1693036	1694649	+	[R] Glyoxylate carboligase
	MJLIPHIC_01580	CDS	COG1942	1694678	1695097	+	[R] Uncharacterized protein, 4-oxalocrotonate tautomerase homolog
788							
	MJLIPHIC_01581	CDS	COG1554	1695107	1697470	-	[G] Trehalose and maltose hydrolases (possible phosphorylases)
	MJLIPHIC_01582	CDS	COG0637	1697467	1698240	-	[R] Predicted phosphatase/phosphohexomutase
789							
	MJLIPHIC_01583	CDS	COG0519	1698411	1699973	+	[F] GMP synthase, PP-ATPase domain/subunit
	MJLIPHIC_01584	CDS	COG1309	1699984	1700661	+	[K] Transcriptional regulator
790							
	MJLIPHIC_01585	CDS	COG0834	1700804	1701661	+	[ET] ABC-type amino acid transport/signal transduction systems,
	MJLIPHIC_01586	CDS	COG0765	1701672	1702463	+	[E] ABC-type amino acid transport system, permease
	MJLIPHIC_01587	CDS	COG1126	1702467	1703264	+	[E] ABC-type polar amino acid transport system,
	MJLIPHIC_01588	CDS	COG0405	1703278	1704852	+	[E] Gamma-glutamyltransferase
791							
	MJLIPHIC_01589	CDS	NA	1704885	1705619	-	NA
	MJLIPHIC_01590	CDS	ROG2178	1705616	1706848	-	NA
	MJLIPHIC_01591	CDS	COG4934	1706845	1708407	-	[O] Predicted protease
792							
	MJLIPHIC_01592	CDS	ROG4412	1708535	1708825	-	NA

793
MJLIPHIC_01593 CDS ROG1039 1709234 1709941 + NA
MJLIPHIC_01594 CDS COG0389 1709944 1711521 + [L] Nucleotidyltransferase/DNA polymerase involved in DNA repair
794
MJLIPHIC_01595 CDS COG1957 1711512 1712564 - [F] Inosine-uridine nucleoside N-ribohydrolase
MJLIPHIC_01596 CDS COG3320 1712618 1714654 - [Q] Putative dehydrogenase domain of multifunctional non-ribosomal
795
MJLIPHIC_01597 CDS COG0589 1714853 1715740 + [T] Universal stress protein UspA and related
MJLIPHIC_01598 CDS COG0531 1715737 1717305 + [E] Amino acid transporters
796
MJLIPHIC_01599 CDS ROG1633 1717307 1718182 - NA
797
MJLIPHIC_01600 CDS COG1028 1718346 1719197 + [IQR] Dehydrogenases with different specificities (related to
798
MJLIPHIC_01601 CDS NA 1719261 1720430 - NA
799
MJLIPHIC_01602 CDS COG1028 1720546 1721337 - [IQR] Dehydrogenases with different specificities (related to
800
MJLIPHIC_01603 CDS NA 1721356 1722288 + NA
801
MJLIPHIC_01604 CDS COG0587 1722361 1725612 + [L] DNA polymerase III, alpha subunit
802
MJLIPHIC_01605 CDS NA 1725589 1726548 - NA
803
MJLIPHIC_01606 CDS NA 1726761 1727360 + NA
MJLIPHIC_01607 CDS COG0778 1727405 1728046 + [C] Nitroreductase
804
MJLIPHIC_01608 CDS COG0219 1728064 1728528 - [J] Predicted rRNA methylase (SpoU class)
805
MJLIPHIC_01609 CDS COG2972 1728834 1731728 + [T] Predicted signal transduction protein with a
MJLIPHIC_01610 CDS COG2018 1731725 1732132 + [R] Uncharacterized distant relative of homeotic protein
MJLIPHIC_01611 CDS ROG1424 1732149 1732514 + NA
MJLIPHIC_01612 CDS COG2229 1732495 1733076 + [R] Predicted GTPase
MJLIPHIC_01613 CDS COG1357 1733082 1733657 + [S] Uncharacterized low-complexity proteins
806
MJLIPHIC_01614 CDS COG1716 1733760 1736459 - [T] FOG: FHA domain
807
MJLIPHIC_01615 CDS COG1520 1736540 1738318 + [S] FOG: WD40-like repeat
808
MJLIPHIC_01616 CDS NA 1738323 1739228 - NA
MJLIPHIC_01617 CDS COG1902 1739234 1740454 - [C] NADH:flavin oxidoreductases, Old Yellow Enzyme family
809
MJLIPHIC_01618 CDS COG0190 1740526 1741386 + [H] 5,10-methylene-tetrahydrofolate dehydrogenase/Methenyl tetrahydrofolate cyclohydrolase
MJLIPHIC_01619 CDS ROG1820 1741383 1741676 + NA
810
MJLIPHIC_01620 CDS COG1309 1742683 1743300 + [K] Transcriptional regulator
MJLIPHIC_01621 CDS COG0654 1743297 1744841 + [HC] 2-polyprenyl-6-methoxyphenol hydroxylase and related FAD-dependent oxidoreductases
811
MJLIPHIC_01622 CDS COG0500 1744845 1745582 - [QR] SAM-dependent methyltransferases
MJLIPHIC_01623 CDS COG2021 1745579 1746685 - [E] Homoserine acetyltransferase
MJLIPHIC_01624 CDS COG2873 1746742 1748073 - [E] O-acetylhomoserine sulfhydrylase
812
MJLIPHIC_01625 CDS COG2838 1748543 1750774 + [C] Monomeric isocitrate dehydrogenase
813
MJLIPHIC_01626 CDS COG0596 1750851 1751690 - [R] Predicted hydrolases or acyltransferases (alpha/beta hydrolase
814
MJLIPHIC_01627 CDS COG0708 1751775 1752596 + [L] Exonuclease III
MJLIPHIC_01628 CDS COG0180 1752612 1753628 + [J] Tryptophanyl-tRNA synthetase
MJLIPHIC_01629 CDS COG1295 1753643 1754680 + [S] Predicted membrane protein
815
MJLIPHIC_01630 CDS COG2076 1754723 1755040 - [P] Membrane transporters of cations and cationic
816
MJLIPHIC_01631 CDS COG2070 1755180 1756019 + [R] Dioxygenases related to 2-nitropropane dioxygenase
817
MJLIPHIC_01632 CDS COG1686 1756022 1757233 - [M] D-alanyl-D-alanine carboxypeptidase
818
MJLIPHIC_01633 CDS COG0161 1757394 1758779 - [H] Adenosylmethionine-8-amino-7-oxononanoate aminotransferase
MJLIPHIC_01634 CDS COG1522 1758776 1759294 - [K] Transcriptional regulators
819
MJLIPHIC_01635 CDS COG1012 1759428 1760915 + [C] NAD-dependent aldehyde dehydrogenases
MJLIPHIC_01636 CDS COG1595 1761019 1761933 + [K] DNA-directed RNA polymerase specialized sigma subunit,
MJLIPHIC_01637 CDS COG3871 1761930 1762454 + [R] Uncharacterized stress protein (general stress protein
820
MJLIPHIC_01638 CDS COG2128 1762408 1762995 - [S] Uncharacterized conserved protein
821
MJLIPHIC_01639 CDS COG0479 1763124 1763909 - [C] Succinate dehydrogenase/fumarate reductase, Fe-S protein subunit
MJLIPHIC_01640 CDS COG1053 1763912 1765666 - [C] Succinate dehydrogenase/fumarate reductase, flavoprotein subunit
MJLIPHIC_01641 CDS COG2142 1765677 1766147 - [C] Succinate dehydrogenase, hydrophobic anchor subunit
MJLIPHIC_01642 CDS COG2009 1766144 1766560 - [C] Succinate dehydrogenase/fumarate reductase, cytochrome b subunit
822
MJLIPHIC_01643 CDS COG0295 1766778 1767176 + [F] Cytidine deaminase
MJLIPHIC_01644 CDS COG0213 1767305 1768546 + [F] Thymidine phosphorylase
MJLIPHIC_01645 CDS COG1816 1768761 1769849 + [F] Adenosine deaminase
823
MJLIPHIC_01646 CDS COG0583 1769956 1770891 + [K] Transcriptional regulator
824
MJLIPHIC_01647 CDS COG1027 1770884 1772296 - [E] Aspartate ammonia-lyase
MJLIPHIC_01648 CDS ROG5870 1772302 1773459 - NA
MJLIPHIC_01649 CDS COG3342 1773456 1774130 - [S] Uncharacterized conserved protein
MJLIPHIC_01650 CDS COG0251 1774127 1774564 - [J] Putative translation initiation inhibitor, yjgF family
MJLIPHIC_01651 CDS ROG0277 1774567 1775832 - NA
825
MJLIPHIC_01652 CDS COG1457 1775984 1777435 - [F] Purine-cytosine permease and related proteins
826
MJLIPHIC_01653 CDS ROG2394 1777578 1778873 - NA
827
MJLIPHIC_01654 CDS NA 1778947 1779114 - NA
MJLIPHIC_01655 CDS COG0791 1779111 1780178 - [M] Cell wall-associated hydrolases (invasion-associated proteins)

MJLIPHIC_01656 CDS NA 1780185 1780475 - NA
MJLIPHIC_01657 CDS COG1917 1780527 1780937 - [S] Uncharacterized conserved protein, contains double-stranded beta-helix
MJLIPHIC_01658 CDS COG1028 1780950 1781672 - [IQR] Dehydrogenases with different specificities (related to
MJLIPHIC_01659 CDS COG1595 1781688 1782320 - [K] DNA-directed RNA polymerase specialized sigma subunit,
MJLIPHIC_01660 CDS COG2771 1782369 1785050 - [K] DNA-binding HTH domain-containing proteins
828
MJLIPHIC_01661 CDS COG1595 1785177 1786091 + [K] DNA-directed RNA polymerase specialized sigma subunit,
829
MJLIPHIC_01662 CDS COG1053 1786092 1787828 - [C] Succinate dehydrogenase/fumarate reductase, flavoprotein subunit
830
MJLIPHIC_01663 CDS COG0035 1787883 1788506 + [F] Uracil phosphoribosyltransferase
831
MJLIPHIC_01664 CDS COG1109 1788531 1790147 - [G] Phosphomannomutase
MJLIPHIC_01665 CDS COG1846 1790144 1790620 - [K] Transcriptional regulators
832
MJLIPHIC_01666 CDS COG3180 1790726 1791835 + [R] Putative ammonia monooxygenase
833
MJLIPHIC_01667 CDS NA 1791793 1792557 - NA
MJLIPHIC_01668 CDS COG0654 1792582 1793730 - [HC] 2-polyprenyl-6-methoxyphenol hydroxylase and related FAD-dependent oxidoreductases
834
MJLIPHIC_01669 CDS COG1309 1793859 1794374 + [K] Transcriptional regulator
835
MJLIPHIC_01670 CDS COG0005 1794381 1795175 - [F] Purine nucleoside phosphorylase
836
MJLIPHIC_01671 CDS COG0624 1795223 1796407 + [E] Acetylornithine deacetylase/Succinyl-diaminopimelate desuccinylase and related deacylases
MJLIPHIC_01672 CDS COG1473 1796404 1797576 + [R] Metal-dependent amidase/aminoacylase/carboxypeptidase
837
MJLIPHIC_01673 CDS COG4213 1797877 1798974 + [G] ABC-type xylose transport system, periplasmic component
MJLIPHIC_01674 CDS COG1129 1798971 1800500 + [G] ABC-type sugar transport system, ATPase component
MJLIPHIC_01675 CDS COG4214 1800501 1801760 + [G] ABC-type xylose transport system, permease component
838
MJLIPHIC_01676 CDS COG0637 1801804 1802463 - [R] Predicted phosphatase/phosphohexomutase
MJLIPHIC_01677 CDS COG1609 1802474 1803502 - [K] Transcriptional regulators
MJLIPHIC_01678 CDS COG1172 1803499 1804524 - [G] Ribose/xylose/arabinose/galactoside ABC-type transport systems, permease components
MJLIPHIC_01679 CDS COG4158 1804521 1805591 - [R] Predicted ABC-type sugar transport system, permease
MJLIPHIC_01680 CDS COG1129 1805591 1807117 - [G] ABC-type sugar transport system, ATPase component
MJLIPHIC_01681 CDS COG1879 1807122 1808108 - [G] ABC-type sugar transport system, periplasmic component
839
MJLIPHIC_01682 CDS COG1069 1808508 1810199 + [C] Ribulose kinase
MJLIPHIC_01683 CDS COG0235 1810196 1810891 + [G] Ribulose-5-phosphate 4-epimerase and related epimerases and
MJLIPHIC_01684 CDS COG2160 1810894 1812399 + [G] L-arabinose isomerase
840
MJLIPHIC_01685 CDS COG2963 1812612 1812956 + [L] Transposase and inactivated derivatives
MJLIPHIC_01686 CDS COG2801 1813034 1813573 + [L] Transposase and inactivated derivatives
841
MJLIPHIC_01687 CDS COG2826 1813566 1814966 - [L] Transposase and inactivated derivatives, IS30 family
842
MJLIPHIC_01688 CDS COG2801 1815062 1815334 + [L] Transposase and inactivated derivatives
843
MJLIPHIC_01689 CDS COG2963 1815822 1816148 + [L] Transposase and inactivated derivatives
MJLIPHIC_01690 CDS COG2801 1816151 1817104 + [L] Transposase and inactivated derivatives
844
MJLIPHIC_01691 CDS NA 1817135 1817806 - NA
845
MJLIPHIC_01692 CDS NA 1818047 1818370 + NA
846
MJLIPHIC_01693 CDS NA 1818478 1818885 - NA
847
MJLIPHIC_01694 CDS ROG8095 1819248 1819676 + NA
MJLIPHIC_01695 CDS COG2801 1819676 1820689 + [L] Transposase and inactivated derivatives
848
MJLIPHIC_01696 CDS COG3335 1820885 1821124 - [L] Transposase and inactivated derivatives
849
MJLIPHIC_01697 CDS COG2963 1821176 1821511 + [L] Transposase and inactivated derivatives
MJLIPHIC_01698 CDS COG2801 1821508 1822437 + [L] Transposase and inactivated derivatives
850
MJLIPHIC_01699 CDS COG3335 1822434 1823294 - [L] Transposase and inactivated derivatives
851
MJLIPHIC_01700 CDS COG3547 1823425 1824546 + [L] Transposase and inactivated derivatives
852
MJLIPHIC_01701 CDS COG3328 1824488 1825807 - [L] Transposase and inactivated derivatives
853
MJLIPHIC_01702 CDS ROG4618 1826089 1826988 + NA
854
MJLIPHIC_01703 CDS ROG0138 1827076 1827543 - NA
855
MJLIPHIC_01704 CDS COG1249 1827585 1829000 + [C] Pyruvate/2-oxoglutarate dehydrogenase complex, dihydrolipoamide dehydrogenase (E3)
856
MJLIPHIC_01705 CDS COG0578 1829180 1830805 + [C] Glycerol-3-phosphate dehydrogenase
MJLIPHIC_01706 CDS COG0564 1830802 1831677 + [J] Pseudouridylate synthases, 23S RNA-specific
857
MJLIPHIC_01707 CDS ROG7907 1831699 1832286 - NA
858
MJLIPHIC_01708 CDS COG1028 1832468 1833205 + [IQR] Dehydrogenases with different specificities (related to
859
MJLIPHIC_01709 CDS COG1024 1833174 1834004 - [I] Enoyl-CoA hydratase/carnithine racemase
MJLIPHIC_01710 CDS COG1309 1833986 1834702 - [K] Transcriptional regulator
860
MJLIPHIC_01711 CDS COG1018 1834732 1835823 + [C] Flavodoxin reductases (ferredoxin-NADPH reductases) family 1
MJLIPHIC_01712 CDS COG3239 1835832 1836980 + [I] Fatty acid desaturase
861
MJLIPHIC_01713 CDS ROG0001 1837050 1837967 - NA
862
MJLIPHIC_01714 CDS COG1309 1838043 1838741 - [K] Transcriptional regulator
863
MJLIPHIC_01715 CDS COG0277 1838767 1840137 + [C] FAD/FMN-containing dehydrogenases

864							
	MJLIPHIC_01716	CDS	COG1595	1840251	1840865	+	[K] DNA-directed RNA polymerase specialized sigma subunit,
	MJLIPHIC_01717	CDS	COG3542	1840862	1841299	+	[S] Uncharacterized conserved protein
865							
	MJLIPHIC_01718	CDS	NA	1841333	1842214	-	NA
	MJLIPHIC_01719	CDS	COG0667	1842253	1843179	-	[C] Predicted oxidoreductases (related to aryl-alcohol dehydrogenases)
866							
	MJLIPHIC_01720	CDS	COG0775	1843365	1844117	+	[F] Nucleoside phosphorylase
867							
	MJLIPHIC_01721	CDS	COG1917	1844210	1844629	+	[S] Uncharacterized conserved protein, contains double-stranded beta-helix
868							
	MJLIPHIC_01722	CDS	COG3806	1844642	1845010	-	[T] Anti-sigma factor
	MJLIPHIC_01723	CDS	COG0266	1845058	1845816	-	[L] Formamidopyrimidine-DNA glycosylase
	MJLIPHIC_01724	CDS	COG1201	1845819	1850342	-	[R] Lhr-like helicases
	MJLIPHIC_01725	CDS	NA	1850404	1850577	-	NA
869							
	MJLIPHIC_01726	CDS	ROG1828	1850694	1852142	+	NA
870							
	MJLIPHIC_01727	CDS	COG1028	1852110	1852925	-	[IQR] Dehydrogenases with different specificities (related to
	MJLIPHIC_01728	CDS	COG1012	1852964	1854547	-	[C] NAD-dependent aldehyde dehydrogenases
871							
	MJLIPHIC_01729	CDS	COG1522	1854619	1855083	+	[K] Transcriptional regulators
	MJLIPHIC_01730	CDS	COG0160	1855136	1856500	+	[E] 4-aminobutyrate aminotransferase and related aminotransferases
872							
	MJLIPHIC_01731	CDS	COG1787	1856508	1857065	-	[V] Predicted endonuclease distantly related to archaeal
873							
	MJLIPHIC_01732	CDS	ROG1758	1857179	1857421	+	NA
	MJLIPHIC_01733	CDS	NA	1857422	1857826	+	NA
	MJLIPHIC_01734	CDS	NA	1857823	1858440	+	NA
	MJLIPHIC_01735	CDS	ROG6919	1858494	1858793	+	NA
874							
	MJLIPHIC_01736	CDS	COG3237	1858875	1859117	-	[S] Uncharacterized protein conserved in bacteria
875							
	MJLIPHIC_01737	CDS	COG2890	1859306	1859974	+	[J] Methylase of polypeptide chain release factors
	MJLIPHIC_01738	CDS	ROG4921	1859971	1860186	+	NA
876							
	MJLIPHIC_01739	CDS	ROG0931	1860268	1861290	-	NA
877							
	MJLIPHIC_01740	CDS	ROG0868	1861547	1862041	-	NA
878							
	MJLIPHIC_01741	CDS	NA	1862204	1863577	+	NA
879							
	MJLIPHIC_01742	CDS	COG1234	1863597	1864526	-	[R] Metal-dependent hydrolases of the beta-lactamase superfamily
880							
	MJLIPHIC_01743	CDS	ROG6919	1864669	1864974	+	NA
881							
	MJLIPHIC_01744	CDS	NA	1864985	1865326	-	NA
	MJLIPHIC_01745	CDS	COG1873	1865313	1865714	-	[S] Uncharacterized conserved protein
	MJLIPHIC_01746	CDS	COG1914	1865711	1866940	-	[P] Mn2+ and Fe2+ transporters of the
	MJLIPHIC_01747	CDS	NA	1866940	1867401	-	NA
	MJLIPHIC_01748	CDS	COG1028	1867419	1868306	-	[IQR] Dehydrogenases with different specificities (related to
	MJLIPHIC_01749	CDS	NA	1868321	1868563	-	NA
882							
	MJLIPHIC_01750	CDS	COG3569	1868641	1869657	-	[L] Topoisomerase IB
883							
	MJLIPHIC_01751	CDS	COG2172	1869761	1870345	+	[T] Anti-sigma regulatory factor (Ser/Thr protein kinase)
884							
	MJLIPHIC_01752	CDS	COG1366	1870342	1870770	-	[T] Anti-anti-sigma regulatory factor (antagonist of anti-sigma
885							
	MJLIPHIC_01753	CDS	ROG7637	1871279	1871512	+	NA
	MJLIPHIC_01754	CDS	ROG0375	1871520	1871675	+	NA
	MJLIPHIC_01755	CDS	ROG1177	1871713	1871970	+	NA
886							
	MJLIPHIC_01756	CDS	ROG6919	1872145	1872453	+	NA
887							
	MJLIPHIC_01757	CDS	NA	1872508	1872858	-	NA
888							
	MJLIPHIC_01758	CDS	NA	1872933	1873895	+	NA
889							
	MJLIPHIC_01759	CDS	ROG1211	1873863	1874426	-	NA
890							
	MJLIPHIC_01760	CDS	COG1063	1874566	1875648	-	[ER] Threonine dehydrogenase and related Zn-dependent dehydrogenases
	MJLIPHIC_01761	CDS	COG0644	1875645	1877078	-	[C] Dehydrogenases (flavoproteins)
	MJLIPHIC_01762	CDS	COG0596	1877075	1877791	-	[R] Predicted hydrolases or acyltransferases (alpha/beta hydrolase
	MJLIPHIC_01763	CDS	COG0477	1877776	1879104	-	[GEPR] Permeases of the major facilitator superfamily
891							
	MJLIPHIC_01764	CDS	COG0583	1879301	1880209	+	[K] Transcriptional regulator
892							
	MJLIPHIC_01765	CDS	COG4858	1880491	1880622	+	[S] Uncharacterized membrane-bound protein conserved in bacteria
893							
	MJLIPHIC_01766	CDS	ROG1177	1880724	1881143	+	NA
894							
	MJLIPHIC_01767	CDS	COG2172	1881247	1881663	+	[T] Anti-sigma regulatory factor (Ser/Thr protein kinase)
	MJLIPHIC_01768	CDS	COG1191	1881660	1882412	+	[K] DNA-directed RNA polymerase specialized sigma subunit
895							
	MJLIPHIC_01769	CDS	COG2068	1882501	1882986	+	[R] Uncharacterized MobA-related protein
896							
	MJLIPHIC_01770	CDS	ROG5165	1882976	1883332	-	NA
897							
	MJLIPHIC_01771	CDS	COG4770	1883433	1885229	-	[I] Acetyl/propionyl-CoA carboxylase, alpha subunit
898							
	MJLIPHIC_01772	CDS	COG2166	1885624	1886034	-	[R] SufE protein probably involved in Fe-S
	MJLIPHIC_01773	CDS	COG2897	1886037	1886924	-	[P] Rhodanese-related sulfurtransferase
899							
	MJLIPHIC_01774	CDS	NA	1887074	1887301	-	NA
900							
	MJLIPHIC_01775	CDS	COG0424	1887457	1888092	-	[D] Nucleotide-binding protein implicated in inhibition of
901							
	MJLIPHIC_01776	CDS	NA	1888189	1888500	-	NA
	MJLIPHIC_01777	CDS	COG4799	1888497	1890125	-	[I] Acetyl-CoA carboxylase, carboxyltransferase component (subunits alpha

902
MJLIPHIC_01778 CDS NA 1890327 1890539 + NA
903
MJLIPHIC_01779 CDS NA 1890966 1891724 - NA
904
MJLIPHIC_01780 CDS ROG2390 1891756 1892388 + NA
MJLIPHIC_01781 CDS NA 1892436 1892603 + NA
905
MJLIPHIC_01782 CDS ROG0124 1892734 1893252 + NA
906
MJLIPHIC_01783 CDS COG2246 1893207 1893878 - [S] Predicted membrane protein
907
MJLIPHIC_01784 CDS COG0026 1893946 1895181 + [F] Phosphoribosylaminoimidazole carboxylase (NCAIR synthetase)
MJLIPHIC_01785 CDS COG0041 1895178 1895687 + [F] Phosphoribosylcarboxyaminoimidazole (NCAIR) mutase
908
MJLIPHIC_01786 CDS COG1960 1896033 1897202 + [I] Acyl-CoA dehydrogenases
MJLIPHIC_01787 CDS COG0340 1897225 1898037 + [H] Biotin-(acetyl-CoA carboxylase) ligase
909
MJLIPHIC_01788 CDS ROG0408 1898113 1898817 - NA
MJLIPHIC_01789 CDS COG1316 1898840 1900303 - [K] Transcriptional regulator
910
MJLIPHIC_01790 CDS COG1091 1900308 1901291 + [M] dTDP-4-dehydrorhamnose reductase
MJLIPHIC_01791 CDS COG1216 1901294 1902184 + [R] Predicted glycosyltransferases
MJLIPHIC_01792 CDS COG1208 1902273 1903262 + [MJ] Nucleoside-diphosphate-sugar pyrophosphorylase involved in lipopolysaccharide biosynthesis/translation
911
MJLIPHIC_01793 CDS COG1051 1903244 1903792 - [F] ADP-ribose pyrophosphatase
MJLIPHIC_01794 CDS COG1478 1903789 1905153 - [S] Uncharacterized conserved protein
MJLIPHIC_01795 CDS COG0391 1905150 1906145 - [S] Uncharacterized conserved protein
912
MJLIPHIC_01796 CDS ROG0165 1906425 1906814 + NA
913
MJLIPHIC_01797 CDS ROG0181 1906855 1907214 - NA
914
MJLIPHIC_01798 CDS ROG0209 1907455 1907826 + NA
MJLIPHIC_01799 CDS COG1109 1907957 1909375 + [G] Phosphomannomutase
MJLIPHIC_01800 CDS ROG1042 1909372 1910454 + NA
MJLIPHIC_01801 CDS COG1482 1910459 1911685 + [G] Phosphomannose isomerase
915
MJLIPHIC_01802 CDS ROG0288 1911718 1913127 - NA
916
MJLIPHIC_01803 CDS COG0531 1913166 1914650 + [E] Amino acid transporters
917
MJLIPHIC_01804 CDS ROG0102 1914807 1916039 + NA
MJLIPHIC_01805 CDS COG1773 1916036 1916209 + [C] Rubredoxin
MJLIPHIC_01806 CDS COG1773 1916206 1916388 + [C] Rubredoxin
MJLIPHIC_01807 CDS COG1309 1916396 1917121 + [K] Transcriptional regulator
918
MJLIPHIC_01808 CDS COG0499 1917272 1918528 + [H] S-adenosylhomocysteine hydrolase
MJLIPHIC_01809 CDS COG0499 1918525 1918728 + [H] S-adenosylhomocysteine hydrolase
919
MJLIPHIC_01810 CDS COG2323 1918791 1919303 - [S] Predicted membrane protein
920
MJLIPHIC_01811 CDS COG2860 1919373 1919999 - [S] Predicted membrane protein
MJLIPHIC_01812 CDS ROG3256 1920009 1921031 - NA
MJLIPHIC_01813 CDS COG0437 1921055 1921969 - [C] Fe-S-cluster-containing hydrogenase components 1
MJLIPHIC_01814 CDS COG0243 1921966 1922877 - [C] Anaerobic dehydrogenases, typically selenocysteine-containing
MJLIPHIC_01815 CDS COG0243 1922828 1924567 - [C] Anaerobic dehydrogenases, typically selenocysteine-containing
MJLIPHIC_01816 CDS COG0243 1924664 1925233 - [C] Anaerobic dehydrogenases, typically selenocysteine-containing
921
MJLIPHIC_01817 CDS COG3276 1925424 1927136 - [J] Selenocysteine-specific translation elongation factor
MJLIPHIC_01818 CDS COG1921 1927124 1928404 - [E] Selenocysteine synthase [seryl-tRNASer selenium transferase]
MJLIPHIC_01819 tRNA NA 1928428 1928522 - NA
922
MJLIPHIC_01820 CDS COG3004 1929588 1930790 - [P] Na+/H+ antiporter
MJLIPHIC_01821 CDS COG3004 1930787 1931401 - [P] Na+/H+ antiporter
923
MJLIPHIC_01822 CDS COG0464 1931592 1932791 + [O] ATPases of the AAA+ class
MJLIPHIC_01823 CDS COG0457 1932788 1933810 + [R] FOG: TPR repeat
924
MJLIPHIC_01824 CDS NA 1933836 1934534 - NA
925
MJLIPHIC_01825 CDS COG4584 1934812 1936173 + [L] Transposase and inactivated derivatives
MJLIPHIC_01826 CDS COG1484 1936170 1937024 + [L] DNA replication protein
926
MJLIPHIC_01827 CDS NA 1937032 1937322 - NA
MJLIPHIC_01828 CDS NA 1937342 1937611 - NA
MJLIPHIC_01829 CDS NA 1937660 1938472 - NA
927
MJLIPHIC_01830 CDS COG2801 1938555 1939568 - [L] Transposase and inactivated derivatives
MJLIPHIC_01831 CDS ROG8095 1939568 1939996 - NA
928
MJLIPHIC_01832 CDS COG2801 1940145 1940354 - [L] Transposase and inactivated derivatives
929
MJLIPHIC_01833 CDS COG5421 1940452 1941963 + [L] Transposase
930
MJLIPHIC_01834 CDS COG2801 1942190 1943023 - [L] Transposase and inactivated derivatives
MJLIPHIC_01835 CDS ROG6591 1943053 1943334 - NA
931
MJLIPHIC_01836 CDS NA 1943545 1943838 - NA
MJLIPHIC_01837 CDS NA 1943875 1944624 - NA
932
MJLIPHIC_01838 CDS ROG0251 1944699 1948409 - NA
933
MJLIPHIC_01839 CDS ROG7290 1948496 1948804 - NA
MJLIPHIC_01840 CDS COG4842 1948794 1949663 - [S] Uncharacterized protein conserved in bacteria
934
MJLIPHIC_01841 CDS ROG5105 1949753 1950385 + NA
MJLIPHIC_01842 CDS COG0745 1950446 1951123 + [TK] Response regulators consisting of a CheY-like
MJLIPHIC_01843 CDS COG0642 1951191 1952852 + [T] Signal transduction histidine kinase

MJLIPHIC_01844 CDS ROG0274 1952849 1954606 + NA
MJLIPHIC_01845 CDS COG1040 1954646 1955299 + [R] Predicted amidophosphoribosyltransferases
MJLIPHIC_01846 CDS COG1544 1955596 1956288 + [J] Ribosome-associated protein Y (PSrp-1)
935
MJLIPHIC_01847 CDS COG0653 1956525 1959386 + [U] Preprotein translocase subunit SecA (ATPase, RNA
936
MJLIPHIC_01848 CDS ROG7267 1959383 1959853 - NA
937
MJLIPHIC_01849 CDS ROG5583 1960095 1961504 + NA
938
MJLIPHIC_01850 CDS COG1292 1961567 1963423 + [M] Choline-glycine betaine transporter
MJLIPHIC_01851 CDS ROG0673 1963433 1963930 + NA
MJLIPHIC_01852 CDS COG1018 1963982 1965133 + [C] Flavodoxin reductases (ferredoxin-NADPH reductases) family 1
MJLIPHIC_01853 CDS COG3239 1965203 1966459 + [I] Fatty acid desaturase
939
MJLIPHIC_01854 CDS ROG7063 1966654 1967082 + NA
940
MJLIPHIC_01855 CDS COG3315 1967099 1968031 - [Q] O-Methyltransferase involved in polyketide biosynthesis
941
MJLIPHIC_01856 CDS COG1162 1968124 1969131 - [R] Predicted GTPases
MJLIPHIC_01857 CDS COG0128 1969128 1970459 - [E] 5-enolpyruvylshikimate-3-phosphate synthase
942
MJLIPHIC_01858 CDS COG2135 1970515 1971303 + [S] Uncharacterized conserved protein
MJLIPHIC_01859 CDS COG2890 1971300 1972799 + [J] Methylase of polypeptide chain release factors
943
MJLIPHIC_01860 CDS COG0500 1972807 1973613 - [QR] SAM-dependent methyltransferases
MJLIPHIC_01861 CDS COG1028 1973653 1974510 - [IQR] Dehydrogenases with different specificities (related to
MJLIPHIC_01862 CDS COG0583 1974521 1975435 - [K] Transcriptional regulator
944
MJLIPHIC_01863 CDS COG0183 1975509 1976741 + [I] Acetyl-CoA acetyltransferase
MJLIPHIC_01864 CDS COG0596 1976738 1977514 + [R] Predicted hydrolases or acyltransferases (alpha/beta hydrolase
MJLIPHIC_01865 CDS COG1788 1977511 1978308 + [I] Acyl CoA:acetate/3-ketoacid CoA transferase, alpha subunit
MJLIPHIC_01866 CDS COG2057 1978305 1978955 + [I] Acyl CoA:acetate/3-ketoacid CoA transferase, beta subunit
945
MJLIPHIC_01867 CDS COG1876 1978960 1979517 - [M] D-alanyl-D-alanine carboxypeptidase
MJLIPHIC_01868 CDS COG2197 1979558 1982275 - [TK] Response regulator containing a CheY-like receiver
MJLIPHIC_01869 CDS COG0583 1982232 1983185 - [K] Transcriptional regulator
MJLIPHIC_01870 CDS COG1804 1983261 1985660 - [C] Predicted acyl-CoA transferases/carnitine dehydratase
MJLIPHIC_01871 CDS COG1960 1985666 1986859 - [I] Acyl-CoA dehydrogenases
MJLIPHIC_01872 CDS COG1960 1986861 1987976 - [I] Acyl-CoA dehydrogenases
MJLIPHIC_01873 CDS COG0543 1988141 1990903 - [HC] 2-polyprenylphenol hydroxylase and related flavodoxin oxidoreductases
MJLIPHIC_01874 CDS COG5517 1990913 1991428 - [Q] Small subunit of phenylpropionate dioxygenase
MJLIPHIC_01875 CDS COG4638 1991430 1992794 - [PR] Phenylpropionate dioxygenase and related ring-hydroxylating dioxygenases,
946
MJLIPHIC_01876 CDS COG0583 1993004 1993930 + [K] Transcriptional regulator
MJLIPHIC_01877 CDS COG4948 1993927 1995030 + [MR] L-alanine-DL-glutamate epimerase and related enzymes of
MJLIPHIC_01878 CDS COG3485 1995064 1995957 + [Q] Protocatechuate 3,4-dioxygenase beta subunit
MJLIPHIC_01879 CDS COG4829 1995965 1996249 + [Q] Muconolactone delta-isomerase
947
MJLIPHIC_01880 CDS COG2606 1996254 1996811 - [S] Uncharacterized conserved protein
948
MJLIPHIC_01881 CDS COG1595 1997014 1997673 + [K] DNA-directed RNA polymerase specialized sigma subunit,
MJLIPHIC_01882 CDS ROG0463 1997670 1997975 + NA
949
MJLIPHIC_01883 CDS COG4770 1998186 1998401 + [I] Acetyl/propionyl-CoA carboxylase, alpha subunit
950
MJLIPHIC_01884 CDS COG3920 1998476 1999975 + [T] Signal transduction histidine kinase
951
MJLIPHIC_01885 CDS ROG0158 2000143 2000397 - NA
952
MJLIPHIC_01886 CDS COG1597 2000646 2001608 - [IR] Sphingosine kinase and enzymes related to
MJLIPHIC_01887 CDS COG3012 2001654 2002043 - [S] Uncharacterized protein conserved in bacteria
953
MJLIPHIC_01888 CDS COG0757 2002111 2002581 + [E] 3-dehydroquinate dehydratase II
MJLIPHIC_01889 CDS ROG2621 2002581 2002958 + NA
954
MJLIPHIC_01890 CDS COG0454 2002965 2003444 - [KR] Histone acetyltransferase HPA2 and related acetyltransferases
MJLIPHIC_01891 CDS COG1169 2003441 2004532 - [HQ] Isochorismate synthase
MJLIPHIC_01892 CDS COG0406 2004529 2005116 - [G] Fructose-2,6-bisphosphatase
955
MJLIPHIC_01893 CDS COG1192 2005199 2005996 + [D] ATPases involved in chromosome partitioning
MJLIPHIC_01894 CDS COG0631 2006011 2006712 + [T] Serine/threonine protein phosphatase
956
MJLIPHIC_01895 CDS ROG2088 2006724 2007941 - NA
MJLIPHIC_01896 CDS COG0513 2007957 2009465 - [LKJ] Superfamily II DNA and RNA helicases
957
MJLIPHIC_01897 CDS ROG0674 2009771 2010469 + NA
958
MJLIPHIC_01898 CDS COG0839 2010476 2011300 - [C] NADH:ubiquinone oxidoreductase subunit 6 (chain J)
959
MJLIPHIC_01899 CDS ROG0206 2011602 2011841 + NA
960
MJLIPHIC_01900 CDS COG1309 2011850 2012509 - [K] Transcriptional regulator
961
MJLIPHIC_01901 CDS ROG0802 2012548 2013789 + NA
962
MJLIPHIC_01902 CDS COG0476 2013931 2015121 + [H] Dinucleotide-utilizing enzymes involved in molybdopterin and
MJLIPHIC_01903 CDS ROG2239 2015158 2016012 + NA
963
MJLIPHIC_01904 CDS COG3695 2016090 2016386 - [L] Predicted methylated DNA-protein cysteine methyltransferase
MJLIPHIC_01905 CDS COG0596 2016388 2017161 - [R] Predicted hydrolases or acyltransferases (alpha/beta hydrolase
964
MJLIPHIC_01906 CDS COG2887 2017285 2020422 + [L] RecB family exonuclease
MJLIPHIC_01907 CDS COG1074 2020419 2023706 + [L] ATP-dependent exoDNAse (exonuclease V) beta subunit
965
MJLIPHIC_01908 CDS COG1814 2023699 2024412 - [S] Uncharacterized membrane protein
966
MJLIPHIC_01909 CDS COG4270 2024543 2024932 - [S] Predicted membrane protein
967
MJLIPHIC_01910 CDS COG1226 2025002 2026096 + [P] Kef-type K+ transport systems, predicted NAD-binding

MJLIPHIC_01911 CDS COG2816 2026093 2027028 + [L] NTP pyrophosphohydrolases containing a Zn-finger, probably
968
MJLIPHIC_01912 CDS COG0695 2027051 2027272 - [O] Glutaredoxin and related proteins
969
MJLIPHIC_01913 CDS COG3237 2027819 2028406 + [S] Uncharacterized protein conserved in bacteria
MJLIPHIC_01914 CDS NA 2028415 2029071 + NA
970
MJLIPHIC_01915 CDS COG0210 2029146 2031275 + [L] Superfamily I DNA and RNA helicases
971
MJLIPHIC_01916 CDS ROG0901 2031777 2032088 + NA
972
MJLIPHIC_01917 CDS COG0661 2032190 2033509 - [R] Predicted unusual protein kinase
MJLIPHIC_01918 CDS ROG1215 2033567 2034406 - NA
MJLIPHIC_01919 CDS COG5282 2034464 2035891 - [S] Uncharacterized conserved protein
973
MJLIPHIC_01920 CDS COG3480 2035981 2037018 + [T] Predicted secreted protein containing a PDZ
MJLIPHIC_01921 CDS COG1615 2037121 2040126 + [S] Uncharacterized conserved protein
974
MJLIPHIC_01922 CDS COG4842 2040301 2040828 + [S] Uncharacterized protein conserved in bacteria
MJLIPHIC_01923 CDS NA 2040821 2041996 + NA
MJLIPHIC_01924 CDS NA 2041993 2042538 + NA
MJLIPHIC_01925 CDS COG3629 2042577 2045987 + [T] DNA-binding transcriptional activator of the SARP
975
MJLIPHIC_01926 CDS COG0277 2046110 2047510 + [C] FAD/FMN-containing dehydrogenases
976
MJLIPHIC_01927 tRNA NA 2047596 2047670 + NA
977
MJLIPHIC_01928 CDS ROG0232 2048141 2048551 - NA
978
MJLIPHIC_01929 CDS COG1309 2048589 2049275 + [K] Transcriptional regulator
979
MJLIPHIC_01930 CDS COG0300 2049610 2050320 - [R] Short-chain dehydrogenases of various substrate specificities
980
MJLIPHIC_01931 CDS COG0789 2050405 2050770 + [K] Predicted transcriptional regulators
981
MJLIPHIC_01932 CDS COG3284 2050786 2052537 - [QK] Transcriptional activator of acetoin/glycerol metabolism
982
MJLIPHIC_01933 CDS ROG0385 2052753 2054381 + NA
983
MJLIPHIC_01934 CDS COG0543 2054473 2055519 + [HC] 2-polyprenylphenol hydroxylase and related flavodoxin oxidoreductases
MJLIPHIC_01935 CDS ROG0820 2055543 2055929 + NA
MJLIPHIC_01936 CDS ROG0820 2055874 2056650 + NA
MJLIPHIC_01937 CDS ROG2012 2056647 2056991 + NA
MJLIPHIC_01938 CDS COG2159 2057028 2058071 + [R] Predicted metal-dependent hydrolase of the TIM-barrel
MJLIPHIC_01939 CDS COG0489 2058068 2058811 + [D] ATPases involved in chromosome partitioning
MJLIPHIC_01940 CDS COG1063 2058811 2059836 + [ER] Threonine dehydrogenase and related Zn-dependent dehydrogenases
MJLIPHIC_01941 CDS COG0459 2059864 2061513 + [O] Chaperonin GroEL (HSP60 family)
MJLIPHIC_01942 CDS COG1359 2061545 2061880 + [S] Uncharacterized conserved protein
984
MJLIPHIC_01943 CDS COG0654 2061887 2063062 - [HC] 2-polyprenyl-6-methoxyphenol hydroxylase and related FAD-dependent oxidoreductases
MJLIPHIC_01944 CDS ROG6950 2063059 2063547 - NA
985
MJLIPHIC_01945 CDS COG0318 2063768 2065315 + [IQ] Acyl-CoA synthetases (AMP-forming)/AMP-acid ligases II
986
MJLIPHIC_01946 CDS COG0477 2065325 2066677 - [GEPR] Permeases of the major facilitator superfamily
MJLIPHIC_01947 CDS COG0596 2066756 2067646 - [R] Predicted hydrolases or acyltransferases (alpha/beta hydrolase
987
MJLIPHIC_01948 CDS COG0583 2067763 2068695 + [K] Transcriptional regulator
988
MJLIPHIC_01949 CDS COG0473 2068955 2070055 + [CE] Isocitrate/isopropylmalate dehydrogenase
989
MJLIPHIC_01950 CDS COG4766 2070195 2070575 + [E] Ethanolamine utilization protein
MJLIPHIC_01951 CDS ROG1559 2070610 2071146 + NA
MJLIPHIC_01952 CDS COG1018 2071147 2072145 + [C] Flavodoxin reductases (ferredoxin-NADPH reductases) family 1
MJLIPHIC_01953 CDS ROG0247 2072174 2073247 + NA
990
MJLIPHIC_01954 CDS COG1960 2073322 2074476 + [I] Acyl-CoA dehydrogenases
MJLIPHIC_01955 CDS COG1804 2074476 2075642 + [C] Predicted acyl-CoA transferases/carnitine dehydratase
MJLIPHIC_01956 CDS COG3777 2075651 2076505 + [S] Uncharacterized conserved protein
MJLIPHIC_01957 CDS COG2301 2076502 2076633 + [G] Citrate lyase beta subunit
991
MJLIPHIC_01958 CDS COG1802 2076671 2077354 - [K] Transcriptional regulators
992
MJLIPHIC_01959 CDS COG2141 2077513 2078376 + [C] Coenzyme F420-dependent N5,N10-methylene tetrahydromethanopterin reductase and
993
MJLIPHIC_01960 CDS COG3653 2078514 2080274 + [Q] N-acyl-D-aspartate/D-glutamate deacylase
MJLIPHIC_01961 CDS COG0596 2080271 2081164 + [R] Predicted hydrolases or acyltransferases (alpha/beta hydrolase
994
MJLIPHIC_01962 CDS NA 2081235 2081378 - NA
995
MJLIPHIC_01963 CDS COG3631 2081621 2082070 - [R] Ketosteroid isomerase-related protein
996
MJLIPHIC_01964 CDS COG0477 2082169 2082789 - [GEPR] Permeases of the major facilitator superfamily
997
MJLIPHIC_01965 CDS ROG0016 2082936 2083688 + NA
998
MJLIPHIC_01966 CDS COG3464 2083696 2084607 - [L] Transposase and inactivated derivatives
999
MJLIPHIC_01967 CDS NA 2085076 2085885 - NA
MJLIPHIC_01968 CDS COG1082 2085866 2086792 - [G] Sugar phosphate isomerases/epimerases
1000
MJLIPHIC_01969 CDS ROG1185 2086952 2087875 - NA
MJLIPHIC_01970 CDS COG0179 2087878 2088741 - [Q] 2-keto-4-pentenoate hydratase/2-oxohepta-3-ene-1,7-dioic acid hydratase (catechol pathway)
1001
MJLIPHIC_01971 CDS COG1802 2088924 2089604 + [K] Transcriptional regulators
1002
MJLIPHIC_01972 CDS ROG0005 2089639 2089956 - NA
1003

	MJLIPHIC_01973	CDS	COG1609	2090102	2091106	-	[K] Transcriptional regulators
1004							
	MJLIPHIC_01974	CDS	COG0141	2091191	2092510	+	[E] Histidinol dehydrogenase
	MJLIPHIC_01975	CDS	COG1028	2092507	2093262	+	[IQR] Dehydrogenases with different specificities (related to
1005							
	MJLIPHIC_01976	CDS	COG1118	2093263	2094333	-	[P] ABC-type sulfate/molybdate transport systems, ATPase component
	MJLIPHIC_01977	CDS	COG4149	2094345	2095157	-	[P] ABC-type molybdate transport system, permease component
	MJLIPHIC_01978	CDS	COG0725	2095154	2095897	-	[P] ABC-type molybdate transport system, periplasmic component
	MJLIPHIC_01979	CDS	ROG1183	2095894	2096298	-	NA
1006							
	MJLIPHIC_01980	CDS	COG1028	2096408	2097154	+	[IQR] Dehydrogenases with different specificities (related to
1007							
	MJLIPHIC_01981	CDS	COG2124	2097139	2097837	-	[Q] Cytochrome P450
	MJLIPHIC_01982	CDS	COG2124	2097868	2098344	-	[Q] Cytochrome P450
1008							
	MJLIPHIC_01983	CDS	COG1835	2098830	2100914	+	[I] Predicted acyltransferases
1009							
	MJLIPHIC_01984	CDS	COG2610	2101118	2102572	+	[GE] H+/gluconate symporter and related permeases
	MJLIPHIC_01985	CDS	COG1804	2102587	2103825	+	[C] Predicted acyl-CoA transferases/carnitine dehydratase
	MJLIPHIC_01986	CDS	COG0119	2103822	2104766	+	[E] Isopropylmalate/homocitrate/citramalate synthases
	MJLIPHIC_01987	CDS	COG1414	2104783	2105565	+	[K] Transcriptional regulator
	MJLIPHIC_01988	CDS	COG0300	2105584	2106414	+	[R] Short-chain dehydrogenases of various substrate specificities
1010							
	MJLIPHIC_01989	CDS	ROG2514	2106472	2106894	-	NA
	MJLIPHIC_01990	CDS	COG2814	2106948	2108135	-	[G] Arabinose efflux permease
	MJLIPHIC_01991	CDS	COG1028	2108135	2108896	-	[IQR] Dehydrogenases with different specificities (related to
1011							
	MJLIPHIC_01992	CDS	COG1309	2108966	2109559	+	[K] Transcriptional regulator
1012							
	MJLIPHIC_01993	CDS	COG0325	2109768	2110526	+	[R] Predicted enzyme with a TIM-barrel fold
1013							
	MJLIPHIC_01994	CDS	COG0604	2110538	2111509	-	[CR] NADPH:quinone reductase and related Zn-dependent oxidoreductases
1014							
	MJLIPHIC_01995	CDS	COG1062	2111591	2112715	+	[C] Zn-dependent alcohol dehydrogenases, class III
	MJLIPHIC_01996	CDS	COG0596	2112758	2113684	+	[R] Predicted hydrolases or acyltransferases (alpha/beta hydrolase
1015							
	MJLIPHIC_01997	CDS	COG1231	2113659	2115026	-	[E] Monoamine oxidase
1016							
	MJLIPHIC_01998	CDS	COG2513	2115055	2116035	+	[G] PEP phosphonomutase and related enzymes
	MJLIPHIC_01999	CDS	COG2072	2116080	2117639	+	[P] Predicted flavoprotein involved in K+ transport
1017							
	MJLIPHIC_02000	CDS	COG2207	2117665	2118420	-	[K] AraC-type DNA-binding domain-containing proteins
1018							
	MJLIPHIC_02001	CDS	NA	2118476	2118826	+	NA
1019							
	MJLIPHIC_02002	CDS	ROG1114	2118922	2119956	-	NA
	MJLIPHIC_02003	CDS	COG3173	2119958	2121094	-	[R] Predicted aminoglycoside phosphotransferase
1020							
	MJLIPHIC_02004	CDS	COG1309	2121141	2121749	+	[K] Transcriptional regulator
	MJLIPHIC_02005	CDS	COG0477	2121822	2123135	+	[GEPR] Permeases of the major facilitator superfamily
	MJLIPHIC_02006	CDS	COG2141	2123135	2124499	+	[C] Coenzyme F420-dependent N5,N10-methylene tetrahydromethanopterin reductase and
	MJLIPHIC_02007	CDS	COG2120	2124514	2125188	+	[S] Uncharacterized proteins, LmbE homologs
1021							
	MJLIPHIC_02008	CDS	COG1063	2125259	2126242	+	[ER] Threonine dehydrogenase and related Zn-dependent dehydrogenases
1022							
	MJLIPHIC_02009	CDS	COG1024	2126239	2127006	-	[I] Enoyl-CoA hydratase/carnithine racemase
1023							
	MJLIPHIC_02010	CDS	COG3226	2127094	2127675	+	[S] Uncharacterized protein conserved in bacteria
1024							
	MJLIPHIC_02011	CDS	COG1007	2127764	2129329	-	[C] NADH:ubiquinone oxidoreductase subunit 2 (chain N)
	MJLIPHIC_02012	CDS	COG1008	2129331	2130917	-	[C] NADH:ubiquinone oxidoreductase subunit 4 (chain M)
	MJLIPHIC_02013	CDS	COG1009	2130914	2132803	-	[CP] NADH:ubiquinone oxidoreductase subunit 5 (chain L)/Multisubunit
	MJLIPHIC_02014	CDS	COG0713	2132811	2133119	-	[C] NADH:ubiquinone oxidoreductase subunit 11 or 4L
	MJLIPHIC_02015	CDS	COG0839	2133116	2133874	-	[C] NADH:ubiquinone oxidoreductase subunit 6 (chain J)
	MJLIPHIC_02016	CDS	COG1143	2133871	2134413	-	[C] Formate hydrogenlyase subunit 6/NADH:ubiquinone oxidoreductase 23
	MJLIPHIC_02017	CDS	COG1005	2134406	2135632	-	[C] NADH:ubiquinone oxidoreductase subunit 1 (chain H)
	MJLIPHIC_02018	CDS	COG1034	2135629	2138013	-	[C] NADH dehydrogenase/NADH:ubiquinone oxidoreductase 75 kD subunit
	MJLIPHIC_02019	CDS	COG1894	2138102	2139433	-	[C] NADH:ubiquinone oxidoreductase, NADH-binding (51 kD) subunit
	MJLIPHIC_02020	CDS	COG1905	2139430	2140167	-	[C] NADH:ubiquinone oxidoreductase 24 kD subunit
	MJLIPHIC_02021	CDS	COG0649	2140228	2141556	-	[C] NADH:ubiquinone oxidoreductase 49 kD subunit 7
	MJLIPHIC_02022	CDS	COG0852	2141556	2142272	-	[C] NADH:ubiquinone oxidoreductase 27 kD subunit
	MJLIPHIC_02023	CDS	COG0377	2142269	2142823	-	[C] NADH:ubiquinone oxidoreductase 20 kD subunit and
	MJLIPHIC_02024	CDS	COG0838	2142814	2143182	-	[C] NADH:ubiquinone oxidoreductase subunit 3 (chain A)
1025							
	MJLIPHIC_02025	CDS	NA	2143366	2143764	-	NA
1026							
	MJLIPHIC_02026	CDS	COG2353	2143828	2144346	-	[S] Uncharacterized conserved protein
	MJLIPHIC_02027	CDS	ROG6586	2144378	2144779	-	NA
1027							
	MJLIPHIC_02028	CDS	COG0500	2144836	2145471	+	[QR] SAM-dependent methyltransferases
1028							
	MJLIPHIC_02029	CDS	ROG2619	2145479	2145835	-	NA
	MJLIPHIC_02030	CDS	COG3173	2145832	2146821	-	[R] Predicted aminoglycoside phosphotransferase
	MJLIPHIC_02031	CDS	COG1960	2146818	2148062	-	[I] Acyl-CoA dehydrogenases
	MJLIPHIC_02032	CDS	COG1309	2148073	2148684	-	[K] Transcriptional regulator
	MJLIPHIC_02033	CDS	COG0119	2148691	2149593	-	[E] Isopropylmalate/homocitrate/citramalate synthases
	MJLIPHIC_02034	CDS	COG1804	2149593	2150795	-	[C] Predicted acyl-CoA transferases/carnitine dehydratase
	MJLIPHIC_02035	CDS	ROG1833	2150792	2152021	-	NA
	MJLIPHIC_02036	CDS	COG3508	2152022	2153125	-	[Q] Homogentisate 1,2-dioxygenase
1029							
	MJLIPHIC_02037	CDS	COG2320	2153254	2153850	-	[S] Uncharacterized conserved protein
	MJLIPHIC_02038	CDS	COG1960	2153861	2155060	-	[I] Acyl-CoA dehydrogenases
1030							
	MJLIPHIC_02039	CDS	COG0627	2155501	2156478	+	[R] Predicted esterase
1031							
	MJLIPHIC_02040	CDS	COG0604	2156545	2157576	-	[CR] NADPH:quinone reductase and related Zn-dependent oxidoreductases
	MJLIPHIC_02041	CDS	COG1960	2157633	2158853	-	[I] Acyl-CoA dehydrogenases
	MJLIPHIC_02042	CDS	COG1960	2158889	2160286	-	[I] Acyl-CoA dehydrogenases
1032							
	MJLIPHIC_02043	CDS	COG0483	2160404	2161186	-	[G] Archaeal fructose-1,6-bisphosphatase and related enzymes of

1033
MJLIPHIC_02044 CDS NA 2161210 2161590 + NA
1034
MJLIPHIC_02045 CDS NA 2161613 2162020 - NA
MJLIPHIC_02046 CDS COG0493 2162046 2162981 - [ER] NADPH-dependent glutamate synthase beta chain and
1035
MJLIPHIC_02047 CDS COG1186 2163153 2164259 + [J] Protein chain release factor B
MJLIPHIC_02048 CDS COG0668 2164249 2165229 + [M] Small-conductance mechanosensitive channel
MJLIPHIC_02049 CDS NA 2165226 2165747 + NA
MJLIPHIC_02050 CDS COG2884 2165793 2166482 + [D] Predicted ATPase involved in cell division
MJLIPHIC_02051 CDS COG2177 2166483 2167379 + [D] Cell division protein
MJLIPHIC_02052 CDS COG0691 2167390 2167875 + [O] tmRNA-binding protein
1036
MJLIPHIC_02053 CDS COG3191 2168040 2169143 + [EQ] L-aminopeptidase/D-esterase
1037
MJLIPHIC_02055 CDS ROG0746 2169678 2171087 - NA
1038
MJLIPHIC_02056 CDS ROG5614 2171252 2172142 + NA
1039
MJLIPHIC_02057 CDS COG0730 2172275 2173018 - [R] Predicted permeases
MJLIPHIC_02058 CDS ROG2292 2173038 2174138 - NA
MJLIPHIC_02059 CDS COG0179 2174138 2175013 - [Q] 2-keto-4-pentenoate hydratase/2-oxohepta-3-ene-1,7-dioic acid hydratase (catechol pathway)
MJLIPHIC_02060 CDS COG0715 2175058 2176023 - [P] ABC-type nitrate/sulfonate/bicarbonate transport systems, periplasmic components
MJLIPHIC_02061 CDS COG0624 2176102 2177433 - [E] Acetylornithine deacetylase/Succinyl-diaminopimelate desuccinylase and related deacylases
MJLIPHIC_02062 CDS COG0600 2177485 2178255 - [P] ABC-type nitrate/sulfonate/bicarbonate transport system, permease component
MJLIPHIC_02063 CDS COG1116 2178248 2179069 - [P] ABC-type nitrate/sulfonate/bicarbonate transport system, ATPase component
MJLIPHIC_02064 CDS NA 2179121 2180020 - NA
1040
MJLIPHIC_02065 CDS COG1167 2180401 2181750 - [KE] Transcriptional regulators containing a DNA-binding HTH
1041
MJLIPHIC_02066 CDS COG3285 2182038 2183003 - [L] Predicted eukaryotic-type DNA primase
1042
MJLIPHIC_02067 CDS ROG2337 2183185 2184645 + NA
1043
MJLIPHIC_02068 CDS COG1309 2184617 2185249 - [K] Transcriptional regulator
1044
MJLIPHIC_02069 CDS COG3687 2185414 2186352 + [R] Predicted metal-dependent hydrolase
MJLIPHIC_02070 CDS NA 2186345 2187502 + NA
1045
MJLIPHIC_02071 CDS ROG2917 2187753 2188592 + NA
MJLIPHIC_02072 CDS COG1605 2188645 2189190 + [E] Chorismate mutase
1046
MJLIPHIC_02073 CDS ROG2508 2189268 2189852 + NA
MJLIPHIC_02074 CDS ROG3514 2189849 2190382 + NA
1047
MJLIPHIC_02075 CDS COG2140 2190361 2190525 - [GR] Thermophilic glucose-6-phosphate isomerase and related metalloenzymes
MJLIPHIC_02076 CDS COG1263 2190853 2192436 - [G] Phosphotransferase system IIC components, glucose/maltose/N-acetylglucosamine-specific
1048
MJLIPHIC_02077 CDS COG2190 2192581 2193051 + [G] Phosphotransferase system IIA components
MJLIPHIC_02078 CDS COG0363 2193073 2193858 + [G] 6-phosphogluconolactonase/Glucosamine-6-phosphate isomerase/deaminase
MJLIPHIC_02079 CDS COG1820 2193860 2195017 + [G] N-acetylglucosamine-6-phosphate deacetylase
1049
MJLIPHIC_02080 CDS COG2771 2195157 2197775 + [K] DNA-binding HTH domain-containing proteins
1050
MJLIPHIC_02081 CDS COG1080 2197776 2200202 - [G] Phosphoenolpyruvate-protein kinase (PTS system EI component
MJLIPHIC_02082 CDS COG2376 2200199 2200831 - [G] Dihydroxyacetone kinase
MJLIPHIC_02083 CDS COG2376 2200833 2201831 - [G] Dihydroxyacetone kinase
MJLIPHIC_02084 CDS COG0580 2201841 2202608 - [G] Glycerol uptake facilitator and related permeases
1051
MJLIPHIC_02085 CDS COG1414 2202762 2203508 + [K] Transcriptional regulator
1052
MJLIPHIC_02086 CDS NA 2203585 2203779 - NA
1053
MJLIPHIC_02087 CDS COG3189 2203799 2204158 + [S] Uncharacterized conserved protein
1054
MJLIPHIC_02088 CDS NA 2204236 2204883 + NA
1055
MJLIPHIC_02089 CDS COG1960 2205201 2206385 - [I] Acyl-CoA dehydrogenases
MJLIPHIC_02090 CDS COG1022 2206382 2207962 - [I] Long-chain acyl-CoA synthetases (AMP-forming)
MJLIPHIC_02091 CDS COG0236 2207959 2208228 - [IQ] Acyl carrier protein
MJLIPHIC_02092 CDS COG1993 2208288 2209358 - [S] Uncharacterized conserved protein
MJLIPHIC_02093 CDS COG0239 2209355 2209720 - [D] Integral membrane protein possibly involved in
MJLIPHIC_02094 CDS COG0239 2209717 2210115 - [D] Integral membrane protein possibly involved in
1056
MJLIPHIC_02095 CDS COG0033 2210186 2211820 + [G] Phosphoglucomutase
MJLIPHIC_02096 CDS ROG2472 2211817 2213085 + NA
MJLIPHIC_02097 tRNA NA 2213142 2213215 + NA
1057
MJLIPHIC_02098 CDS COG3177 2213518 2214726 - [S] Uncharacterized conserved protein
1058
MJLIPHIC_02099 CDS NA 2214785 2215036 + NA
MJLIPHIC_02100 CDS NA 2215060 2215749 + NA
1059
MJLIPHIC_02101 CDS NA 2215760 2216200 - NA
MJLIPHIC_02102 CDS ROG3866 2216197 2216793 - NA
1060
MJLIPHIC_02103 CDS NA 2217006 2217425 + NA
MJLIPHIC_02104 CDS COG5280 2217440 2221084 + [S] Phage-related minor tail protein
MJLIPHIC_02105 CDS NA 2221081 2221323 + NA
MJLIPHIC_02106 CDS NA 2221320 2222246 + NA
1061
MJLIPHIC_02107 CDS COG1403 2222742 2224376 + [V] Restriction endonuclease
1062
MJLIPHIC_02108 CDS ROG4463 2224483 2224938 + NA
1063
MJLIPHIC_02109 CDS COG1484 2225109 2225963 - [L] DNA replication protein

MJLIPHIC_02110 CDS COG4584 2225960 2227321 - [L] Transposase and inactivated derivatives
1064
MJLIPHIC_02111 CDS COG1309 2228170 2228763 - [K] Transcriptional regulator
1065
MJLIPHIC_02112 CDS ROG1797 2228863 2229687 + NA
1066
MJLIPHIC_02113 CDS COG0604 2229760 2230743 + [CR] NADPH:quinone reductase and related Zn-dependent oxidoreductases
MJLIPHIC_02114 CDS COG0702 2230794 2231729 + [MG] Predicted nucleoside-diphosphate-sugar epimerases
1067
MJLIPHIC_02115 CDS NA 2231882 2232784 + NA
MJLIPHIC_02116 CDS COG3220 2232790 2233644 + [S] Uncharacterized protein conserved in bacteria
MJLIPHIC_02117 CDS NA 2233641 2233796 + NA
1068
MJLIPHIC_02118 CDS COG0318 2234051 2234716 + [IQ] Acyl-CoA synthetases (AMP-forming)/AMP-acid ligases II
MJLIPHIC_02119 CDS COG3320 2234833 2237583 + [Q] Putative dehydrogenase domain of multifunctional non-ribosomal
1069
MJLIPHIC_02120 CDS NA 2237715 2238554 + NA
MJLIPHIC_02121 CDS COG1012 2238592 2240016 + [C] NAD-dependent aldehyde dehydrogenases
MJLIPHIC_02122 CDS COG2186 2240088 2240876 + [K] Transcriptional regulators
1070
MJLIPHIC_02123 CDS COG3959 2241188 2241976 + [G] Transketolase, N-terminal subunit
MJLIPHIC_02124 CDS COG3958 2241973 2243007 + [G] Transketolase, C-terminal subunit
MJLIPHIC_02125 CDS COG0006 2243051 2244442 + [E] Xaa-Pro aminopeptidase
MJLIPHIC_02126 CDS NA 2244483 2244662 + NA
MJLIPHIC_02127 CDS COG2084 2244650 2245630 + [I] 3-hydroxyisobutyrate dehydrogenase and related beta-hydroxyacid dehydrogenases
1071
MJLIPHIC_02128 CDS COG0777 2245536 2247044 - [I] Acetyl-CoA carboxylase beta subunit
MJLIPHIC_02129 CDS COG1804 2247041 2248261 - [C] Predicted acyl-CoA transferases/carnitine dehydratase
1072
MJLIPHIC_02130 CDS COG1055 2248470 2249870 - [P] Na+/H+ antiporter NhaD and related arsenite
1073
MJLIPHIC_02131 CDS COG2186 2250029 2250721 + [K] Transcriptional regulators
1074
MJLIPHIC_02132 CDS COG3973 2250877 2253087 + [R] Superfamily I DNA and RNA helicases
MJLIPHIC_02133 CDS COG1990 2253121 2253447 + [S] Uncharacterized conserved protein
1075
MJLIPHIC_02134 CDS COG2141 2253444 2254820 - [C] Coenzyme F420-dependent N5,N10-methylene tetrahydromethanopterin reductase and
MJLIPHIC_02135 CDS COG2141 2254841 2256019 - [C] Coenzyme F420-dependent N5,N10-methylene tetrahydromethanopterin reductase and
1076
MJLIPHIC_02136 CDS COG1309 2256454 2257758 + [K] Transcriptional regulator
1077
MJLIPHIC_02137 CDS COG2020 2257799 2258323 - [O] Putative protein-S-isoprenylcysteine methyltransferase
1078
MJLIPHIC_02138 CDS COG1309 2258412 2259011 + [K] Transcriptional regulator
1079
MJLIPHIC_02139 CDS COG2184 2258987 2259802 - [D] Protein involved in cell division
MJLIPHIC_02140 CDS COG2844 2259796 2260797 - [O] UTP:GlnB (protein PII) uridylyltransferase
1080
MJLIPHIC_02141 CDS COG0531 2260965 2262506 + [E] Amino acid transporters
MJLIPHIC_02142 CDS COG3665 2262509 2263351 + [S] Uncharacterized conserved protein
MJLIPHIC_02143 CDS COG3665 2263348 2263971 + [S] Uncharacterized conserved protein
MJLIPHIC_02144 CDS COG1984 2264025 2265989 + [E] Allophanate hydrolase subunit 2
MJLIPHIC_02145 CDS COG0154 2265986 2267656 + [J] Asp-tRNAAsn/Glu-tRNAGln amidotransferase A subunit and related
1081
MJLIPHIC_02146 CDS COG0697 2267604 2268596 - [GER] Permeases of the drug/metabolite transporter (DMT)
MJLIPHIC_02147 CDS COG1960 2268648 2269790 - [I] Acyl-CoA dehydrogenases
MJLIPHIC_02148 CDS COG1960 2269802 2271034 - [I] Acyl-CoA dehydrogenases
1082
MJLIPHIC_02149 CDS COG2202 2271207 2271569 + [T] FOG: PAS/PAC domain
MJLIPHIC_02150 CDS COG0789 2271642 2272415 + [K] Predicted transcriptional regulators
1083
MJLIPHIC_02151 CDS COG1309 2272412 2273065 - [K] Transcriptional regulator
MJLIPHIC_02152 CDS COG1309 2273072 2273773 - [K] Transcriptional regulator
1084
MJLIPHIC_02153 CDS NA 2274343 2275170 + NA
1085
MJLIPHIC_02154 CDS COG2200 2275150 2276997 - [T] FOG: EAL domain
MJLIPHIC_02155 CDS ROG0192 2277027 2279198 - NA
MJLIPHIC_02156 CDS ROG6491 2279195 2279947 - NA
1086
MJLIPHIC_02157 CDS COG0788 2280301 2281332 + [F] Formyltetrahydrofolate hydrolase
MJLIPHIC_02158 CDS COG2030 2281354 2281806 + [I] Acyl dehydratase
1087
MJLIPHIC_02159 CDS COG1232 2281948 2283312 + [H] Protoporphyrinogen oxidase
MJLIPHIC_02160 CDS COG1073 2283309 2284205 + [R] Hydrolases of the alpha/beta superfamily
1088
MJLIPHIC_02161 CDS COG1028 2284280 2285101 - [IQR] Dehydrogenases with different specificities (related to
MJLIPHIC_02162 CDS COG1960 2285144 2286337 - [I] Acyl-CoA dehydrogenases
1089
MJLIPHIC_02163 CDS COG1028 2286449 2287189 + [IQR] Dehydrogenases with different specificities (related to
MJLIPHIC_02164 CDS COG0183 2287186 2288409 + [I] Acetyl-CoA acetyltransferase
MJLIPHIC_02165 CDS COG1960 2288417 2289610 + [I] Acyl-CoA dehydrogenases
MJLIPHIC_02166 CDS COG1802 2289614 2290282 + [K] Transcriptional regulators
MJLIPHIC_02167 CDS COG1804 2290327 2290884 + [C] Predicted acyl-CoA transferases/carnitine dehydratase
MJLIPHIC_02168 CDS COG1804 2290862 2291515 + [C] Predicted acyl-CoA transferases/carnitine dehydratase
1090
MJLIPHIC_02169 CDS NA 2291531 2292232 - NA
1091
MJLIPHIC_02170 CDS COG1028 2292544 2293488 + [IQR] Dehydrogenases with different specificities (related to
MJLIPHIC_02171 CDS COG2890 2293485 2294720 + [J] Methylase of polypeptide chain release factors
1092
MJLIPHIC_02172 CDS COG1062 2294858 2294998 - [C] Zn-dependent alcohol dehydrogenases, class III
MJLIPHIC_02173 CDS COG0318 2294995 2296497 - [IQ] Acyl-CoA synthetases (AMP-forming)/AMP-acid ligases II
MJLIPHIC_02174 CDS COG0318 2296490 2297992 - [IQ] Acyl-CoA synthetases (AMP-forming)/AMP-acid ligases II
MJLIPHIC_02175 CDS COG0318 2297967 2299418 - [IQ] Acyl-CoA synthetases (AMP-forming)/AMP-acid ligases II
MJLIPHIC_02176 CDS COG1024 2299418 2300200 - [I] Enoyl-CoA hydratase/carnithine racemase
MJLIPHIC_02177 CDS COG1024 2300197 2301003 - [I] Enoyl-CoA hydratase/carnithine racemase
MJLIPHIC_02178 CDS COG1024 2301024 2301845 - [I] Enoyl-CoA hydratase/carnithine racemase
1093
MJLIPHIC_02179 CDS COG1024 2302116 2303000 + [I] Enoyl-CoA hydratase/carnithine racemase

MJLIPHIC_02180 CDS COG0183 2303054 2304229 + [I] Acetyl-CoA acetyltransferase
1094
MJLIPHIC_02181 CDS ROG6516 2304294 2305031 - NA
1095
MJLIPHIC_02182 CDS COG1024 2305317 2306081 + [I] Enoyl-CoA hydratase/carnithine racemase
MJLIPHIC_02183 CDS COG1028 2306091 2306888 + [IQR] Dehydrogenases with different specificities (related to
MJLIPHIC_02184 CDS COG1024 2307025 2307846 + [I] Enoyl-CoA hydratase/carnithine racemase
MJLIPHIC_02185 CDS COG2159 2307861 2309111 + [R] Predicted metal-dependent hydrolase of the TIM-barrel
MJLIPHIC_02186 CDS COG0318 2309115 2310743 + [IQ] Acyl-CoA synthetases (AMP-forming)/AMP-acid ligases II
MJLIPHIC_02187 CDS COG2159 2310740 2311594 + [R] Predicted metal-dependent hydrolase of the TIM-barrel
MJLIPHIC_02188 CDS COG1960 2311599 2312648 + [I] Acyl-CoA dehydrogenases
MJLIPHIC_02189 CDS COG1960 2312674 2313864 + [I] Acyl-CoA dehydrogenases
1096
MJLIPHIC_02190 CDS COG2159 2313886 2315121 - [R] Predicted metal-dependent hydrolase of the TIM-barrel
1097
MJLIPHIC_02191 CDS COG0332 2315214 2316362 - [I] 3-oxoacyl-[acyl-carrier-protein] synthase III
1098
MJLIPHIC_02192 CDS COG0243 2316518 2318806 + [C] Anaerobic dehydrogenases, typically selenocysteine-containing
MJLIPHIC_02193 CDS COG1012 2318803 2320329 + [C] NAD-dependent aldehyde dehydrogenases
1099
MJLIPHIC_02194 CDS ROG6516 2320363 2321103 - NA
MJLIPHIC_02195 CDS COG2124 2321106 2322314 - [Q] Cytochrome P450
1100
MJLIPHIC_02196 CDS COG1012 2322534 2324021 + [C] NAD-dependent aldehyde dehydrogenases
1101
MJLIPHIC_02197 CDS COG0318 2324005 2325657 - [IQ] Acyl-CoA synthetases (AMP-forming)/AMP-acid ligases II
MJLIPHIC_02198 CDS COG1335 2325677 2326294 - [Q] Amidases related to nicotinamidase
MJLIPHIC_02199 CDS COG4638 2326291 2327307 - [PR] Phenylpropionate dioxygenase and related ring-hydroxylating dioxygenases,
1102
MJLIPHIC_02200 CDS ROG3864 2327539 2327985 + NA
1103
MJLIPHIC_02201 CDS ROG6954 2327988 2328650 - NA
1104
MJLIPHIC_02202 CDS COG0702 2328800 2329819 + [MG] Predicted nucleoside-diphosphate-sugar epimerases
MJLIPHIC_02203 CDS COG0325 2329816 2330946 + [R] Predicted enzyme with a TIM-barrel fold
1105
MJLIPHIC_02204 CDS COG4585 2330943 2333141 - [T] Signal transduction histidine kinase
1106
MJLIPHIC_02205 CDS COG0784 2333298 2333648 + [T] FOG: CheY-like receiver
1107
MJLIPHIC_02206 CDS COG0654 2333777 2335201 + [HC] 2-polyprenyl-6-methoxyphenol hydroxylase and related FAD-dependent
oxidoreductases
1108
MJLIPHIC_02207 CDS COG2140 2335507 2336553 + [GR] Thermophilic glucose-6-phosphate isomerase and related metalloenzymes
1109
MJLIPHIC_02208 CDS COG4799 2336583 2338136 - [I] Acetyl-CoA carboxylase, carboxyltransferase component (subunits alpha
1110
MJLIPHIC_02209 CDS NA 2338174 2339292 + NA
MJLIPHIC_02210 CDS COG2124 2339303 2340538 + [Q] Cytochrome P450
1111
MJLIPHIC_02211 CDS ROG3760 2340549 2340686 - NA
MJLIPHIC_02212 CDS COG1966 2340686 2342992 - [T] Carbon starvation protein, predicted membrane protein
1112
MJLIPHIC_02213 CDS COG2207 2343165 2344226 - [K] AraC-type DNA-binding domain-containing proteins
1113
MJLIPHIC_02214 CDS COG2427 2344455 2345024 + [S] Uncharacterized conserved protein
1114
MJLIPHIC_02215 CDS COG1740 2345095 2346066 + [C] Ni,Fe-hydrogenase I small subunit
MJLIPHIC_02216 CDS COG0374 2346098 2347705 + [C] Ni,Fe-hydrogenase I large subunit
MJLIPHIC_02217 CDS COG0680 2347746 2348543 + [C] Ni,Fe-hydrogenase maturation factor
MJLIPHIC_02218 CDS NA 2348540 2348812 + NA
MJLIPHIC_02219 CDS NA 2348829 2350079 + NA
MJLIPHIC_02220 CDS COG0694 2350076 2350963 + [O] Thioredoxin-like proteins and domains
MJLIPHIC_02221 CDS ROG3815 2350960 2352150 + NA
MJLIPHIC_02222 CDS NA 2352147 2352983 + NA
1115
MJLIPHIC_02223 CDS COG0378 2352990 2353643 - [OK] Ni2+-binding GTPase involved in regulation of
MJLIPHIC_02224 CDS COG0375 2353782 2354117 - [R] Zn finger protein HypA/HybF (possibly regulating
1116
MJLIPHIC_02225 CDS COG0068 2354278 2356566 + [O] Hydrogenase maturation factor
MJLIPHIC_02226 CDS COG0298 2356566 2356826 + [O] Hydrogenase maturation factor
MJLIPHIC_02227 CDS COG0409 2356823 2357926 + [O] Hydrogenase maturation factor
MJLIPHIC_02228 CDS COG0309 2357919 2358992 + [O] Hydrogenase maturation factor
1117
MJLIPHIC_02229 CDS COG1793 2358993 2360558 - [L] ATP-dependent DNA ligase
MJLIPHIC_02230 CDS COG2041 2360646 2362310 - [R] Sulfite oxidase and related enzymes
1118
MJLIPHIC_02231 CDS COG1028 2362387 2363154 + [IQR] Dehydrogenases with different specificities (related to
MJLIPHIC_02232 CDS COG0028 2363217 2364947 + [EH] Thiamine pyrophosphate-requiring enzymes [acetolactate synthase, pyruvate
1119
MJLIPHIC_02233 CDS COG1733 2364966 2365505 - [K] Predicted transcriptional regulators
1120
MJLIPHIC_02234 CDS ROG0016 2365604 2366356 + NA
1121
MJLIPHIC_02235 CDS COG3464 2366364 2367275 - [L] Transposase and inactivated derivatives
1122
MJLIPHIC_02236 CDS COG1733 2367744 2367905 - [K] Predicted transcriptional regulators
1123
MJLIPHIC_02237 CDS COG0500 2368080 2368895 + [QR] SAM-dependent methyltransferases
MJLIPHIC_02238 CDS COG3173 2368918 2369979 + [R] Predicted aminoglycoside phosphotransferase
1124
MJLIPHIC_02239 CDS COG0730 2370008 2370769 - [R] Predicted permeases
1125
MJLIPHIC_02240 CDS NA 2370961 2371452 + NA
1126
MJLIPHIC_02241 CDS COG2124 2371548 2373032 - [Q] Cytochrome P450
1127
MJLIPHIC_02242 CDS COG1309 2373164 2373823 + [K] Transcriptional regulator
MJLIPHIC_02243 CDS COG4221 2373847 2374710 + [R] Short-chain alcohol dehydrogenase of unknown specificity
1128

MJLIPHIC_02244 CDS COG1785 2374713 2376248 - [P] Alkaline phosphatase
1129
MJLIPHIC_02245 CDS COG4637 2376350 2377582 - [R] Predicted ATPase
MJLIPHIC_02246 CDS COG0389 2377545 2378615 - [L] Nucleotidyltransferase/DNA polymerase involved in DNA repair
MJLIPHIC_02247 CDS COG1309 2378620 2379225 - [K] Transcriptional regulator
1130
MJLIPHIC_02248 CDS COG0431 2379342 2379905 + [R] Predicted flavoprotein
1131
MJLIPHIC_02249 CDS COG0695 2380379 2380618 + [O] Glutaredoxin and related proteins
MJLIPHIC_02250 CDS COG1780 2380763 2381098 + [F] Protein involved in ribonucleotide reduction
MJLIPHIC_02251 CDS COG0209 2381065 2383233 + [F] Ribonucleotide reductase, alpha subunit
1132
MJLIPHIC_02252 CDS ROG1121 2383420 2383806 - NA
1133
MJLIPHIC_02253 CDS NA 2383835 2384536 + NA
1134
MJLIPHIC_02254 CDS COG3607 2384537 2384956 - [R] Predicted lactoylglutathione lyase
1135
MJLIPHIC_02255 CDS COG0477 2385228 2386637 + [GEPR] Permeases of the major facilitator superfamily
MJLIPHIC_02256 CDS NA 2386634 2386777 + NA
MJLIPHIC_02257 CDS COG3393 2386828 2387679 + [R] Predicted acetyltransferase
1136
MJLIPHIC_02258 CDS COG1309 2387651 2388226 - [K] Transcriptional regulator
1137
MJLIPHIC_02259 CDS NA 2388350 2389228 + NA
1138
MJLIPHIC_02260 CDS COG0644 2389298 2390485 - [C] Dehydrogenases (flavoproteins)
1139
MJLIPHIC_02261 CDS COG1309 2390723 2391373 + [K] Transcriptional regulator
1140
MJLIPHIC_02262 CDS COG2072 2391489 2392988 + [P] Predicted flavoprotein involved in K+ transport
1141
MJLIPHIC_02263 CDS COG3631 2393074 2393598 + [R] Ketosteroid isomerase-related protein
1142
MJLIPHIC_02264 CDS COG0208 2393803 2394789 + [F] Ribonucleotide reductase, beta subunit
1143
MJLIPHIC_02265 CDS COG0627 2394859 2396241 + [R] Predicted esterase
MJLIPHIC_02266 CDS ROG5681 2396241 2397821 + NA
1144
MJLIPHIC_02267 CDS COG2141 2398010 2399365 - [C] Coenzyme F420-dependent N5,N10-methylene tetrahydromethanopterin reductase and
1145
MJLIPHIC_02268 CDS COG1064 2399432 2400481 - [R] Zn-dependent alcohol dehydrogenases
1146
MJLIPHIC_02269 CDS COG2350 2400669 2400944 - [S] Uncharacterized protein conserved in bacteria
MJLIPHIC_02270 CDS COG0614 2400954 2402027 - [P] ABC-type Fe3+-hydroxamate transport system, periplasmic component
1147
MJLIPHIC_02271 CDS COG0843 2402297 2403985 + [C] Heme/copper-type cytochrome/quinol oxidases, subunit 1
MJLIPHIC_02272 CDS COG0560 2404009 2405250 + [E] Phosphoserine phosphatase
1148
MJLIPHIC_02273 CDS NA 2405381 2405671 + NA
1149
MJLIPHIC_02274 CDS COG1167 2405673 2407100 - [KE] Transcriptional regulators containing a DNA-binding HTH
1150
MJLIPHIC_02275 CDS COG2364 2407331 2408014 + [S] Predicted membrane protein
MJLIPHIC_02276 CDS COG1119 2407969 2408817 + [P] ABC-type molybdenum transport system, ATPase component/photorepair
MJLIPHIC_02277 CDS NA 2408810 2409631 + NA
MJLIPHIC_02278 CDS COG1024 2409628 2410335 + [I] Enoyl-CoA hydratase/carnithine racemase
MJLIPHIC_02279 CDS COG0500 2410370 2411350 + [QR] SAM-dependent methyltransferases
MJLIPHIC_02280 CDS ROG2908 2411359 2412522 + NA
1151
MJLIPHIC_02281 CDS ROG0285 2412595 2413296 + NA
1152
MJLIPHIC_02282 CDS COG1115 2413328 2414872 - [E] Na+/alanine symporter
1153
MJLIPHIC_02283 CDS COG1520 2414990 2416273 - [S] FOG: WD40-like repeat
1154
MJLIPHIC_02284 CDS COG1208 2416370 2417113 + [MJ] Nucleoside-diphosphate-sugar pyrophosphorylase involved in lipopolysaccharide biosynthesis/translation
1155
MJLIPHIC_02285 CDS NA 2417125 2417403 - NA
1156
MJLIPHIC_02286 CDS COG1304 2417893 2418912 + [C] L-lactate dehydrogenase (FMN-dependent) and related alpha-hydroxy
MJLIPHIC_02287 CDS ROG0016 2418870 2419571 + NA
1157
MJLIPHIC_02288 CDS COG3464 2419579 2420490 - [L] Transposase and inactivated derivatives
1158
MJLIPHIC_02289 CDS COG1304 2420692 2421201 + [C] L-lactate dehydrogenase (FMN-dependent) and related alpha-hydroxy
MJLIPHIC_02290 CDS COG1819 2421207 2422448 + [GC] Glycosyl transferases, related to UDP-glucuronosyltransferase
1159
MJLIPHIC_02291 CDS ROG0401 2422408 2422818 - NA
MJLIPHIC_02292 CDS COG2146 2422826 2423842 - [PR] Ferredoxin subunits of nitrite reductase and
MJLIPHIC_02293 CDS COG3349 2423839 2425368 - [S] Uncharacterized conserved protein
MJLIPHIC_02294 CDS ROG3662 2425365 2425508 - NA
MJLIPHIC_02295 CDS ROG3662 2425710 2426033 - NA
MJLIPHIC_02296 CDS COG1562 2426030 2426977 - [I] Phytoene/squalene synthetase
MJLIPHIC_02297 CDS COG1233 2426974 2428527 - [Q] Phytoene dehydrogenase and related proteins
1160
MJLIPHIC_02298 CDS COG0438 2428660 2429865 - [M] Glycosyltransferase
MJLIPHIC_02299 CDS COG1543 2430027 2431571 - [S] Uncharacterized conserved protein
MJLIPHIC_02300 CDS COG0500 2431568 2432353 - [QR] SAM-dependent methyltransferases
1161
MJLIPHIC_02301 CDS COG2086 2432570 2433361 + [C] Electron transfer flavoprotein, beta subunit
MJLIPHIC_02302 CDS COG2025 2433429 2434385 + [C] Electron transfer flavoprotein, alpha subunit
1162
MJLIPHIC_02303 CDS COG0702 2434550 2435293 + [MG] Predicted nucleoside-diphosphate-sugar epimerases
MJLIPHIC_02304 CDS COG0778 2435295 2435942 + [C] Nitroreductase
1163
MJLIPHIC_02305 CDS COG3176 2436073 2436912 + [R] Putative hemolysin
MJLIPHIC_02306 CDS COG0204 2436909 2437739 + [I] 1-acyl-sn-glycerol-3-phosphate acyltransferase
MJLIPHIC_02307 CDS COG1104 2437787 2438995 + [E] Cysteine sulfinate desulfinase/cysteine desulfurase and related

MJLIPHIC_02308 CDS COG0482 2438992 2440062 + [J] Predicted tRNA(5-methylaminomethyl-2-thiouridylate) methyltransferase, contains the PP-loop
MJLIPHIC_02309 CDS ROG0167 2440088 2441098 + NA
1164
MJLIPHIC_02310 CDS COG0318 2441100 2442674 - [IQ] Acyl-CoA synthetases (AMP-forming)/AMP-acid ligases II
MJLIPHIC_02311 CDS ROG6766 2442671 2443087 - NA
1165
MJLIPHIC_02312 CDS COG0272 2443172 2445277 + [L] NAD-dependent DNA ligase (contains BRCT domain
1166
MJLIPHIC_02313 CDS ROG0914 2445348 2446007 - NA
1167
MJLIPHIC_02314 CDS COG0721 2446108 2446407 + [J] Asp-tRNAAsn/Glu-tRNAGln amidotransferase C subunit
MJLIPHIC_02315 CDS COG0154 2446404 2447888 + [J] Asp-tRNAAsn/Glu-tRNAGln amidotransferase A subunit and related
MJLIPHIC_02316 CDS COG0205 2447932 2449029 + [G] 6-phosphofructokinase
MJLIPHIC_02317 CDS COG0064 2449026 2450537 + [J] Asp-tRNAAsn/Glu-tRNAGln amidotransferase B subunit (PET112 homolog)
1168
MJLIPHIC_02318 CDS ROG6516 2450596 2451150 - NA
1169
MJLIPHIC_02319 CDS COG2133 2451352 2452455 - [G] Glucose/sorbosone dehydrogenases
1170
MJLIPHIC_02320 CDS COG2259 2452620 2453462 + [S] Predicted membrane protein
1171
MJLIPHIC_02321 CDS ROG2024 2453493 2453882 - NA
1172
MJLIPHIC_02322 CDS COG0028 2454205 2456061 + [EH] Thiamine pyrophosphate-requiring enzymes [acetolactate synthase, pyruvate
MJLIPHIC_02323 CDS COG0440 2456125 2456637 + [E] Acetolactate synthase, small (regulatory) subunit
MJLIPHIC_02324 CDS COG0059 2456694 2457707 + [EH] Ketol-acid reductoisomerase
1173
MJLIPHIC_02325 CDS COG0655 2457779 2458390 - [R] Multimeric flavodoxin WrbA
MJLIPHIC_02326 CDS ROG2494 2458418 2458879 - NA
MJLIPHIC_02327 CDS COG1233 2458912 2460321 - [Q] Phytoene dehydrogenase and related proteins
1174
MJLIPHIC_02328 CDS COG0111 2460414 2462000 + [HE] Phosphoglycerate dehydrogenase and related dehydrogenases
MJLIPHIC_02329 CDS COG0473 2461997 2463019 + [CE] Isocitrate/isopropylmalate dehydrogenase
1175
MJLIPHIC_02330 CDS COG2271 2463113 2464309 - [G] Sugar phosphate permease
1176
MJLIPHIC_02331 CDS COG1506 2464387 2465580 + [E] Dipeptidyl aminopeptidases/acylaminoacyl-peptidases
MJLIPHIC_02332 CDS COG0179 2465631 2466410 + [Q] 2-keto-4-pentenoate hydratase/2-oxohepta-3-ene-1,7-dioic acid hydratase (catechol pathway)
MJLIPHIC_02333 CDS COG0008 2466407 2467867 + [J] Glutamyl- and glutaminyl-tRNA synthetases
1177
MJLIPHIC_02334 tRNA NA 2467984 2468058 + NA
MJLIPHIC_02335 tRNA NA 2468127 2468200 + NA
1178
MJLIPHIC_02336 CDS COG1414 2468381 2469082 - [K] Transcriptional regulator
1179
MJLIPHIC_02337 CDS COG0065 2469194 2470648 + [E] 3-isopropylmalate dehydratase large subunit
MJLIPHIC_02338 CDS COG0066 2470672 2471265 + [E] 3-isopropylmalate dehydratase small subunit
1180
MJLIPHIC_02339 CDS COG0776 2471469 2472095 + [L] Bacterial nucleoid DNA-binding protein
1181
MJLIPHIC_02340 CDS COG1051 2472156 2473145 - [F] ADP-ribose pyrophosphatase
MJLIPHIC_02341 CDS COG0855 2473142 2475352 - [P] Polyphosphate kinase
MJLIPHIC_02342 CDS COG1920 2475381 2476046 - [S] Uncharacterized conserved protein
1182
MJLIPHIC_02343 CDS COG0240 2476128 2477108 + [C] Glycerol-3-phosphate dehydrogenase
MJLIPHIC_02344 CDS COG0626 2477113 2478222 + [E] Cystathionine beta-lyases/cystathionine gamma-synthases
1183
MJLIPHIC_02345 CDS COG1181 2478455 2479492 + [M] D-alanine-D-alanine ligase and related ATP-grasp enzymes
1184
MJLIPHIC_02346 CDS ROG1774 2479493 2480080 - NA
MJLIPHIC_02347 CDS ROG3776 2480097 2480333 - NA
1185
MJLIPHIC_02348 CDS COG0611 2480440 2481453 + [H] Thiamine monophosphate kinase
MJLIPHIC_02349 CDS COG0692 2481435 2482163 + [L] Uracil DNA glycosylase
1186
MJLIPHIC_02350 CDS ROG3131 2482164 2482478 - NA
1187
MJLIPHIC_02351 CDS COG0227 2482555 2482749 - [J] Ribosomal protein L28
1188
MJLIPHIC_02352 CDS COG1461 2482984 2484648 + [R] Predicted kinase related to dihydroxyacetone kinase
MJLIPHIC_02353 CDS COG1200 2484648 2486906 + [LK] RecG-like helicase
MJLIPHIC_02354 CDS ROG0066 2486903 2487607 + NA
1189
MJLIPHIC_02355 CDS COG0454 2487622 2488128 - [KR] Histone acetyltransferase HPA2 and related acetyltransferases
1190
MJLIPHIC_02356 CDS NA 2488188 2488766 + NA
1191
MJLIPHIC_02357 CDS COG0656 2488797 2489648 - [R] Aldo/keto reductases, related to diketogulonate reductase
MJLIPHIC_02358 CDS COG0656 2489654 2490481 - [R] Aldo/keto reductases, related to diketogulonate reductase
1192
MJLIPHIC_02359 CDS COG0657 2490495 2491631 + [I] Esterase/lipase
MJLIPHIC_02360 CDS COG1651 2491665 2492393 + [O] Protein-disulfide isomerase
MJLIPHIC_02361 CDS COG4243 2492393 2493025 + [S] Predicted membrane protein
MJLIPHIC_02362 CDS COG1038 2493061 2496444 + [C] Pyruvate carboxylase
MJLIPHIC_02363 CDS COG0742 2496486 2496995 + [L] N6-adenine-specific methylase
MJLIPHIC_02364 CDS COG0669 2497021 2497497 + [H] Phosphopantetheine adenylyltransferase
1193
MJLIPHIC_02365 CDS ROG3432 2497580 2498158 + NA
1194
MJLIPHIC_02366 CDS COG3599 2498281 2499018 + [D] Cell division initiation protein
MJLIPHIC_02367 CDS COG1399 2499178 2499699 + [R] Predicted metal-binding, possibly nucleic acid-binding protein
MJLIPHIC_02368 CDS COG0571 2499696 2500388 + [K] dsRNA-specific ribonuclease
MJLIPHIC_02369 CDS COG0266 2500395 2501252 + [L] Formamidopyrimidine-DNA glycosylase
1195
MJLIPHIC_02370 CDS NA 2501271 2502047 - NA
1196
MJLIPHIC_02371 CDS COG1765 2502168 2502587 + [O] Predicted redox protein, regulator of disulfide
MJLIPHIC_02372 CDS COG1254 2502584 2502880 + [C] Acylphosphatases

MJLIPHIC_02373 CDS COG1196 2502907 2506494 + [D] Chromosome segregation ATPases
MJLIPHIC_02374 CDS COG0552 2506567 2508042 + [U] Signal recognition particle GTPase
1197
MJLIPHIC_02375 CDS COG0004 2508282 2509631 + [P] Ammonia permease
MJLIPHIC_02376 CDS COG0347 2509665 2510003 + [E] Nitrogen regulatory protein PII
MJLIPHIC_02377 CDS COG2844 2510091 2512565 + [O] UTP:GlnB (protein PII) uridylyltransferase
1198
MJLIPHIC_02378 CDS NA 2512566 2513411 - NA
1199
MJLIPHIC_02379 CDS COG0667 2513512 2514516 + [C] Predicted oxidoreductases (related to aryl-alcohol dehydrogenases)
MJLIPHIC_02380 CDS COG0541 2514582 2516150 + [U] Signal recognition particle GTPase
MJLIPHIC_02381 CDS COG1228 2516150 2517226 + [Q] Imidazolonepropionase and related amidohydrolases
1200
MJLIPHIC_02382 CDS COG1686 2517308 2518135 - [M] D-alanyl-D-alanine carboxypeptidase
MJLIPHIC_02383 CDS COG1686 2518205 2519095 - [M] D-alanyl-D-alanine carboxypeptidase
1201
MJLIPHIC_02384 CDS ROG2963 2519128 2519562 + NA
1202
MJLIPHIC_02385 CDS COG0228 2519767 2520237 + [J] Ribosomal protein S16
MJLIPHIC_02386 CDS COG1837 2520258 2520488 + [R] Predicted RNA-binding protein (contains KH domain)
MJLIPHIC_02387 CDS COG0806 2520523 2521044 + [J] RimM protein, required for 16S rRNA
MJLIPHIC_02388 CDS COG0336 2521059 2521736 + [J] tRNA-(guanine-N1)-methyltransferase
1203
MJLIPHIC_02389 CDS ROG1505 2521737 2522681 - NA
1204
MJLIPHIC_02390 CDS COG0335 2523015 2523356 + [J] Ribosomal protein L19
MJLIPHIC_02391 CDS COG0681 2523424 2524293 + [U] Signal peptidase I
MJLIPHIC_02392 CDS COG0164 2524304 2525122 + [L] Ribonuclease HII
MJLIPHIC_02393 CDS ROG0213 2525199 2525504 + NA
1205
MJLIPHIC_02394 CDS COG0412 2525519 2526229 - [Q] Dienelactone hydrolase and related enzymes
MJLIPHIC_02395 CDS COG0397 2526276 2527730 - [S] Uncharacterized conserved protein
MJLIPHIC_02396 CDS COG3651 2527780 2528202 - [S] Uncharacterized protein conserved in bacteria
1206
MJLIPHIC_02397 CDS COG2508 2528276 2529892 - [TQ] Regulator of polyketide synthase expression
1207
MJLIPHIC_02398 CDS COG1012 2530003 2531496 + [C] NAD-dependent aldehyde dehydrogenases
MJLIPHIC_02399 CDS COG0160 2531510 2532895 + [E] 4-aminobutyrate aminotransferase and related aminotransferases
1208
MJLIPHIC_02400 CDS COG0647 2533045 2533785 + [G] Predicted sugar phosphatases of the HAD
1209
MJLIPHIC_02401 CDS COG1802 2534312 2535046 + [K] Transcriptional regulators
MJLIPHIC_02402 CDS COG0251 2535043 2535504 + [J] Putative translation initiation inhibitor, yjgF family
1210
MJLIPHIC_02403 CDS COG3427 2535507 2536103 - [S] Uncharacterized conserved protein
1211
MJLIPHIC_02404 CDS COG2141 2536199 2537197 + [C] Coenzyme F420-dependent N5,N10-methylene tetrahydromethanopterin reductase and
1212
MJLIPHIC_02405 CDS COG0715 2537542 2538522 + [P] ABC-type nitrate/sulfonate/bicarbonate transport systems, periplasmic
components
MJLIPHIC_02406 CDS COG1116 2538553 2539353 + [P] ABC-type nitrate/sulfonate/bicarbonate transport system, ATPase component
MJLIPHIC_02407 CDS COG0600 2539350 2540198 + [P] ABC-type nitrate/sulfonate/bicarbonate transport system, permease component
MJLIPHIC_02408 CDS COG0600 2540195 2540983 + [P] ABC-type nitrate/sulfonate/bicarbonate transport system, permease component
1213
MJLIPHIC_02409 CDS COG1529 2541046 2543313 + [C] Aerobic-type carbon monoxide dehydrogenase, large subunit
MJLIPHIC_02410 CDS COG1319 2543310 2544191 + [C] Aerobic-type carbon monoxide dehydrogenase, middle subunit
MJLIPHIC_02411 CDS COG2080 2544178 2544684 + [C] Aerobic-type carbon monoxide dehydrogenase, small subunit
1214
MJLIPHIC_02412 CDS ROG3012 2544747 2545967 + NA
MJLIPHIC_02413 CDS COG1960 2546004 2547203 + [I] Acyl-CoA dehydrogenases
MJLIPHIC_02414 CDS COG0022 2547242 2549401 + [C] Pyruvate/2-oxoglutarate dehydrogenase complex, dehydrogenase (E1) component,
MJLIPHIC_02415 CDS COG1804 2549414 2550649 + [C] Predicted acyl-CoA transferases/carnitine dehydratase
MJLIPHIC_02416 CDS COG1946 2550646 2551455 + [I] Acyl-CoA thioesterase
1215
MJLIPHIC_02417 CDS COG0318 2551339 2553918 - [IQ] Acyl-CoA synthetases (AMP-forming)/AMP-acid ligases II
1216
MJLIPHIC_02418 CDS COG2609 2554008 2556401 - [C] Pyruvate dehydrogenase complex, dehydrogenase (E1) component
1217
MJLIPHIC_02419 CDS COG1522 2556494 2557012 + [K] Transcriptional regulators
1218
MJLIPHIC_02420 CDS COG2814 2557143 2557574 + [G] Arabinose efflux permease
MJLIPHIC_02421 CDS COG0477 2557571 2558533 + [GEPR] Permeases of the major facilitator superfamily
1219
MJLIPHIC_02422 CDS ROG5614 2558654 2559433 + NA
1220
MJLIPHIC_02423 CDS COG0667 2559495 2560349 - [C] Predicted oxidoreductases (related to aryl-alcohol dehydrogenases)
MJLIPHIC_02424 CDS COG1846 2560435 2560866 - [K] Transcriptional regulators
MJLIPHIC_02425 CDS COG1028 2560939 2562006 - [IQR] Dehydrogenases with different specificities (related to
MJLIPHIC_02426 CDS COG0179 2562032 2562889 - [Q] 2-keto-4-pentenoate hydratase/2-oxohepta-3-ene-1,7-dioic acid hydratase
(catechol pathway)
1221
MJLIPHIC_02427 CDS COG1802 2562958 2563698 + [K] Transcriptional regulators
MJLIPHIC_02428 CDS ROG1884 2563709 2564953 + NA
MJLIPHIC_02429 CDS NA 2564950 2565369 + NA
MJLIPHIC_02430 CDS COG1414 2565362 2566084 + [K] Transcriptional regulator
1222
MJLIPHIC_02431 CDS ROG5331 2566112 2566801 - NA
MJLIPHIC_02432 CDS COG0654 2566837 2568414 - [HC] 2-polyprenyl-6-methoxyphenol hydroxylase and related FAD-dependent
oxidoreductases
1223
MJLIPHIC_02433 CDS COG0477 2568753 2570159 + [GEPR] Permeases of the major facilitator superfamily
1224
MJLIPHIC_02434 CDS COG0160 2570219 2571472 - [E] 4-aminobutyrate aminotransferase and related aminotransferases
1225
MJLIPHIC_02435 CDS COG1012 2571561 2573021 + [C] NAD-dependent aldehyde dehydrogenases
1226
MJLIPHIC_02436 CDS COG1802 2573037 2573759 - [K] Transcriptional regulators
MJLIPHIC_02437 CDS COG3473 2573756 2574478 - [Q] Maleate cis-trans isomerase
1227
MJLIPHIC_02438 CDS COG0277 2574544 2577402 + [C] FAD/FMN-containing dehydrogenases

1228
MJLIPHIC_02439 CDS COG0624 2577399 2578571 - [E] Acetylornithine deacetylase/Succinyl-diaminopimelate desuccinylase and related deacylases
MJLIPHIC_02440 CDS COG1167 2578576 2579775 - [KE] Transcriptional regulators containing a DNA-binding HTH
MJLIPHIC_02441 CDS COG0006 2579779 2580945 - [E] Xaa-Pro aminopeptidase
MJLIPHIC_02442 CDS COG3473 2580942 2581664 - [Q] Maleate cis-trans isomerase
1229
MJLIPHIC_02443 CDS COG1052 2581744 2582772 + [CHR] Lactate dehydrogenase and related dehydrogenases
MJLIPHIC_02444 CDS COG0154 2582769 2584172 + [J] Asp-tRNAAsn/Glu-tRNAGln amidotransferase A subunit and related
MJLIPHIC_02445 CDS ROG6690 2584169 2584669 + NA
MJLIPHIC_02446 CDS ROG4240 2584688 2585911 + NA
1230
MJLIPHIC_02447 CDS COG0600 2585908 2586690 - [P] ABC-type nitrate/sulfonate/bicarbonate transport system, permease component
MJLIPHIC_02448 CDS COG0600 2586687 2587553 - [P] ABC-type nitrate/sulfonate/bicarbonate transport system, permease component
MJLIPHIC_02449 CDS COG1116 2587550 2588344 - [P] ABC-type nitrate/sulfonate/bicarbonate transport system, ATPase component
MJLIPHIC_02450 CDS COG0715 2588395 2589414 - [P] ABC-type nitrate/sulfonate/bicarbonate transport systems, periplasmic components
MJLIPHIC_02451 CDS COG0066 2589424 2589951 - [E] 3-isopropylmalate dehydratase small subunit
MJLIPHIC_02452 CDS COG0065 2589951 2591192 - [E] 3-isopropylmalate dehydratase large subunit
MJLIPHIC_02453 CDS COG2513 2591193 2592062 - [G] PEP phosphonomutase and related enzymes
MJLIPHIC_02454 CDS COG1414 2592063 2592866 - [K] Transcriptional regulator
1231
MJLIPHIC_02455 CDS COG0792 2593273 2593641 + [L] Predicted endonuclease distantly related to archaeal
MJLIPHIC_02456 CDS COG0606 2593643 2595151 + [O] Predicted ATPase with chaperone activity
1232
MJLIPHIC_02457 CDS NA 2595271 2595642 - NA
1233
MJLIPHIC_02458 CDS COG0758 2595846 2596268 + [LU] Predicted Rossmann fold nucleotide-binding protein involved
1234
MJLIPHIC_02459 CDS COG2375 2596370 2597242 + [P] Siderophore-interacting protein
MJLIPHIC_02460 CDS COG1304 2597267 2598430 + [C] L-lactate dehydrogenase (FMN-dependent) and related alpha-hydroxy
1235
MJLIPHIC_02461 CDS ROG3096 2598528 2599364 + NA
1236
MJLIPHIC_02462 CDS ROG4954 2599455 2600462 - NA
1237
MJLIPHIC_02463 CDS COG4974 2601428 2602330 + [L] Site-specific recombinase XerD
MJLIPHIC_02464 CDS COG0596 2602327 2603208 + [R] Predicted hydrolases or acyltransferases (alpha/beta hydrolase
1238
MJLIPHIC_02465 CDS NA 2603192 2604121 - NA
MJLIPHIC_02466 CDS COG0739 2604148 2604828 - [M] Membrane proteins related to metalloendopeptidases
1239
MJLIPHIC_02467 CDS COG0052 2604960 2605793 + [J] Ribosomal protein S2
MJLIPHIC_02468 CDS COG0264 2605809 2606636 + [J] Translation elongation factor Ts
1240
MJLIPHIC_02469 CDS COG0154 2606795 2608255 + [J] Asp-tRNAAsn/Glu-tRNAGln amidotransferase A subunit and related
1241
MJLIPHIC_02470 CDS COG2366 2608467 2609555 + [R] Protein related to penicillin acylase
MJLIPHIC_02471 CDS NA 2609552 2610118 + NA
MJLIPHIC_02472 CDS COG1136 2610123 2610845 + [V] ABC-type antimicrobial peptide transport system, ATPase
MJLIPHIC_02473 CDS COG0531 2610869 2612377 + [E] Amino acid transporters
1242
MJLIPHIC_02474 CDS COG3733 2612646 2614619 + [Q] Cu2+-containing amine oxidase
1243
MJLIPHIC_02475 CDS NA 2614912 2615016 + NA
1244
MJLIPHIC_02476 CDS COG1929 2615039 2616163 - [G] Glycerate kinase
MJLIPHIC_02477 CDS COG1052 2616171 2617124 - [CHR] Lactate dehydrogenase and related dehydrogenases
1245
MJLIPHIC_02478 CDS COG0599 2617182 2617610 - [S] Uncharacterized homolog of gamma-carboxymuconolactone decarboxylase subunit
MJLIPHIC_02479 CDS COG1802 2617653 2618345 - [K] Transcriptional regulators
1246
MJLIPHIC_02480 CDS COG0757 2618813 2619349 + [E] 3-dehydroquinate dehydratase II
MJLIPHIC_02481 CDS NA 2619346 2619741 + NA
MJLIPHIC_02482 CDS COG0596 2619738 2620601 + [R] Predicted hydrolases or acyltransferases (alpha/beta hydrolase
MJLIPHIC_02483 CDS COG1028 2620622 2621404 + [IQR] Dehydrogenases with different specificities (related to
MJLIPHIC_02484 CDS COG1028 2621453 2622208 + [IQR] Dehydrogenases with different specificities (related to
MJLIPHIC_02485 CDS COG2814 2622262 2623653 + [G] Arabinose efflux permease
1247
MJLIPHIC_02486 CDS ROG1378 2623666 2624094 - NA
1248
MJLIPHIC_02487 CDS COG0500 2624131 2624811 + [QR] SAM-dependent methyltransferases
1249
MJLIPHIC_02488 CDS COG0528 2624947 2625693 + [F] Uridylate kinase
MJLIPHIC_02489 CDS COG0233 2625741 2626298 + [J] Ribosome recycling factor
MJLIPHIC_02490 CDS COG0575 2626299 2627177 + [I] CDP-diglyceride synthetase
1250
MJLIPHIC_02491 CDS COG0697 2627136 2628050 - [GER] Permeases of the drug/metabolite transporter (DMT)
1251
MJLIPHIC_02492 CDS COG0583 2628197 2629090 + [K] Transcriptional regulator
MJLIPHIC_02493 CDS COG0820 2629087 2630205 + [R] Predicted Fe-S-cluster redox enzyme
1252
MJLIPHIC_02494 CDS COG1802 2630288 2630956 - [K] Transcriptional regulators
1253
MJLIPHIC_02495 CDS COG2390 2631062 2632069 - [K] Transcriptional regulator, contains sigma factor-related N-terminal
1254
MJLIPHIC_02496 CDS COG1028 2632377 2633153 + [IQR] Dehydrogenases with different specificities (related to
MJLIPHIC_02497 CDS COG2271 2633198 2634526 + [G] Sugar phosphate permease
MJLIPHIC_02498 CDS COG1052 2634530 2635594 + [CHR] Lactate dehydrogenase and related dehydrogenases
1255
MJLIPHIC_02499 CDS COG1012 2635751 2637235 - [C] NAD-dependent aldehyde dehydrogenases
MJLIPHIC_02500 CDS COG1309 2637301 2637849 - [K] Transcriptional regulator
MJLIPHIC_02501 CDS ROG0938 2637892 2639031 - NA
1256
MJLIPHIC_02502 CDS COG3776 2639135 2639776 + [S] Predicted membrane protein
1257
MJLIPHIC_02503 CDS COG1472 2639792 2642041 - [G] Beta-glucosidase-related glycosidases
MJLIPHIC_02504 CDS COG1309 2642079 2642627 - [K] Transcriptional regulator
1258
MJLIPHIC_02505 CDS COG2124 2642713 2643969 + [Q] Cytochrome P450

	MJLIPHIC_02506	CDS	COG1141	2643983	2644174	+	[C] Ferredoxin
1259							
	MJLIPHIC_02507	CDS	ROG0200	2644175	2644993	-	NA
	MJLIPHIC_02508	CDS	COG3386	2645003	2645887	-	[G] Gluconolactonase
1260							
	MJLIPHIC_02509	CDS	NA	2645963	2646667	+	NA
	MJLIPHIC_02510	CDS	COG2124	2646664	2647914	+	[Q] Cytochrome P450
	MJLIPHIC_02511	CDS	COG1063	2647916	2648884	+	[ER] Threonine dehydrogenase and related Zn-dependent dehydrogenases
	MJLIPHIC_02512	CDS	COG3836	2648881	2649663	+	[G] 2,4-dihydroxyhept-2-ene-1,7-dioic acid aldolase
	MJLIPHIC_02513	CDS	COG1028	2649678	2650415	+	[IQR] Dehydrogenases with different specificities (related to
	MJLIPHIC_02514	CDS	COG2072	2650418	2651911	+	[P] Predicted flavoprotein involved in K+ transport
	MJLIPHIC_02515	CDS	COG0657	2651913	2652845	+	[I] Esterase/lipase
1261							
	MJLIPHIC_02516	CDS	COG3491	2652955	2653929	-	[R] Isopenicillin N synthase and related dioxygenases
	MJLIPHIC_02517	CDS	COG2233	2653937	2655358	-	[F] Xanthine/uracil permeases
1262							
	MJLIPHIC_02518	CDS	ROG1294	2655727	2656023	+	NA
1263							
	MJLIPHIC_02519	CDS	NA	2656031	2656528	-	NA
	MJLIPHIC_02520	CDS	COG2343	2656525	2657232	-	[S] Uncharacterized protein conserved in bacteria
1264							
	MJLIPHIC_02521	CDS	NA	2657374	2658036	+	NA
1265							
	MJLIPHIC_02522	CDS	ROG1627	2658074	2658328	-	NA
1266							
	MJLIPHIC_02523	CDS	COG0415	2658411	2659769	-	[L] Deoxyribodipyrimidine photolyase
1267							
	MJLIPHIC_02524	CDS	NA	2659776	2659961	+	NA
1268							
	MJLIPHIC_02525	CDS	COG0743	2660068	2661222	+	[I] 1-deoxy-D-xylulose 5-phosphate reductoisomerase
	MJLIPHIC_02526	CDS	COG0750	2661219	2662439	+	[M] Predicted membrane-associated Zn-dependent proteases 1
	MJLIPHIC_02527	CDS	COG0821	2662456	2663622	+	[I] Enzyme involved in the deoxyxylulose pathway
	MJLIPHIC_02528	CDS	COG3393	2663679	2664533	+	[R] Predicted acetyltransferase
1269							
	MJLIPHIC_02529	CDS	ROG5870	2664545	2665084	-	NA
1270							
	MJLIPHIC_02530	CDS	ROG6516	2665175	2665783	+	NA
1271							
	MJLIPHIC_02531	CDS	COG0768	2665920	2667740	+	[M] Cell division protein FtsI/penicillin-binding protein 2
	MJLIPHIC_02532	CDS	ROG6659	2667790	2668377	+	NA
1272							
	MJLIPHIC_02533	CDS	COG2050	2668397	2668783	-	[Q] Uncharacterized protein, possibly involved in aromatic
1273							
	MJLIPHIC_02534	CDS	COG0024	2668859	2669755	+	[J] Methionine aminopeptidase
	MJLIPHIC_02535	CDS	COG1492	2669752	2671242	+	[H] Cobyric acid synthase
1274							
	MJLIPHIC_02536	CDS	COG4954	2671246	2671677	-	[S] Uncharacterized protein conserved in bacteria
1275							
	MJLIPHIC_02537	CDS	COG2207	2671766	2672536	+	[K] AraC-type DNA-binding domain-containing proteins
	MJLIPHIC_02538	CDS	NA	2672585	2673241	+	NA
1276							
	MJLIPHIC_02539	CDS	NA	2673315	2673878	+	NA
1277							
	MJLIPHIC_02540	CDS	COG0454	2673880	2675709	-	[KR] Histone acetyltransferase HPA2 and related acetyltransferases
	MJLIPHIC_02541	CDS	COG0367	2675706	2677511	-	[E] Asparagine synthase (glutamine-hydrolyzing)
1278							
	MJLIPHIC_02542	CDS	COG0174	2677864	2679237	+	[E] Glutamine synthetase
	MJLIPHIC_02543	CDS	COG2071	2679266	2679973	+	[R] Predicted glutamine amidotransferases
	MJLIPHIC_02544	CDS	COG1012	2679970	2681325	+	[C] NAD-dependent aldehyde dehydrogenases
	MJLIPHIC_02545	CDS	COG1028	2681364	2682137	+	[IQR] Dehydrogenases with different specificities (related to
	MJLIPHIC_02546	CDS	COG2186	2682163	2682897	+	[K] Transcriptional regulators
1279							
	MJLIPHIC_02547	CDS	COG1414	2682903	2683643	-	[K] Transcriptional regulator
1280							
	MJLIPHIC_02548	CDS	COG3485	2683715	2684461	+	[Q] Protocatechuate 3,4-dioxygenase beta subunit
	MJLIPHIC_02549	CDS	COG3485	2684461	2685018	+	[Q] Protocatechuate 3,4-dioxygenase beta subunit
	MJLIPHIC_02550	CDS	COG0015	2685015	2686217	+	[F] Adenylosuccinate lyase
	MJLIPHIC_02551	CDS	COG0599	2686214	2686618	+	[S] Uncharacterized homolog of gamma-carboxymuconolactone decarboxylase subunit
1281							
	MJLIPHIC_02552	CDS	COG2186	2686690	2687430	-	[K] Transcriptional regulators
1282							
	MJLIPHIC_02553	CDS	COG0640	2687544	2687846	+	[K] Predicted transcriptional regulators
1283							
	MJLIPHIC_02554	CDS	COG0310	2687906	2688613	+	[P] ABC-type Co2+ transport system, permease component
	MJLIPHIC_02555	CDS	ROG5339	2688610	2688948	+	NA
	MJLIPHIC_02556	CDS	COG0619	2688950	2689729	+	[P] ABC-type cobalt transport system, permease component
	MJLIPHIC_02557	CDS	COG1122	2689726	2690475	+	[P] ABC-type cobalt transport system, ATPase component
1284							
	MJLIPHIC_02558	CDS	COG1249	2690486	2691871	-	[C] Pyruvate/2-oxoglutarate dehydrogenase complex, dihydrolipoamide dehydrogenase (E3)
	MJLIPHIC_02559	CDS	COG2267	2691876	2692907	-	[I] Lysophospholipase
1285							
	MJLIPHIC_02560	CDS	COG0579	2693158	2694690	+	[R] Predicted dehydrogenase
	MJLIPHIC_02561	CDS	COG2153	2694704	2695177	+	[R] Predicted acyltransferase
	MJLIPHIC_02562	CDS	COG1239	2695174	2697057	+	[H] Mg-chelatase subunit ChlI
	MJLIPHIC_02563	CDS	COG2109	2697070	2697684	+	[H] ATP:corrinoid adenosyltransferase
	MJLIPHIC_02564	CDS	COG1797	2697720	2699096	+	[H] Cobyrinic acid a,c-diamide synthase
1286							
	MJLIPHIC_02565	tRNA	NA	2699782	2699875	-	NA
1287							
	MJLIPHIC_02566	CDS	COG0477	2700404	2701951	+	[GEPR] Permeases of the major facilitator superfamily
	MJLIPHIC_02567	CDS	ROG0697	2701968	2702672	+	NA
	MJLIPHIC_02568	CDS	COG0442	2702719	2704464	+	[J] Prolyl-tRNA synthetase
1288							
	MJLIPHIC_02569	CDS	ROG2250	2704485	2704958	-	NA
	MJLIPHIC_02570	CDS	NA	2704955	2705503	-	NA
1289							
	MJLIPHIC_02571	CDS	COG0779	2705700	2706245	+	[S] Uncharacterized protein conserved in bacteria
	MJLIPHIC_02572	CDS	COG0195	2706242	2707273	+	[K] Transcription elongation factor
1290							

MJLIPHIC_02573 CDS COG0532 2707775 2710579 + [J] Translation initiation factor 2 (IF-2; GTPase)
MJLIPHIC_02574 CDS COG0858 2710579 2711049 + [J] Ribosome-binding factor A
MJLIPHIC_02575 CDS COG0618 2711058 2712074 + [R] Exopolyphosphatase-related proteins
MJLIPHIC_02576 CDS COG0534 2712067 2713434 + [V] Na+-driven multidrug efflux pump
1291
MJLIPHIC_02577 CDS COG0500 2713440 2714060 - [QR] SAM-dependent methyltransferases
MJLIPHIC_02578 CDS COG1116 2714104 2714925 - [P] ABC-type nitrate/sulfonate/bicarbonate transport system, ATPase component
MJLIPHIC_02579 CDS COG0715 2714946 2715989 - [P] ABC-type nitrate/sulfonate/bicarbonate transport systems, periplasmic
components
MJLIPHIC_02580 CDS COG0600 2716010 2716903 - [P] ABC-type nitrate/sulfonate/bicarbonate transport system, permease component
1292
MJLIPHIC_02581 CDS COG1609 2717025 2718056 - [K] Transcriptional regulators
1293
MJLIPHIC_02582 CDS COG0673 2718267 2719415 + [R] Predicted dehydrogenases and related proteins
MJLIPHIC_02583 CDS ROG0338 2719412 2720602 + NA
MJLIPHIC_02584 CDS COG1082 2720599 2721432 + [G] Sugar phosphate isomerases/epimerases
1294
MJLIPHIC_02585 CDS COG2510 2721419 2722243 - [S] Predicted membrane protein
1295
MJLIPHIC_02586 CDS COG1024 2722400 2723158 - [I] Enoyl-CoA hydratase/carnithine racemase
1296
MJLIPHIC_02587 CDS COG5552 2723205 2723471 + [S] Uncharacterized conserved protein
MJLIPHIC_02588 CDS COG4293 2723468 2724031 + [S] Uncharacterized protein conserved in bacteria
1297
MJLIPHIC_02589 CDS COG2936 2724054 2725727 - [R] Predicted acyl esterases
MJLIPHIC_02590 CDS NA 2725764 2726348 - NA
1298
MJLIPHIC_02591 CDS COG3547 2726511 2727722 - [L] Transposase and inactivated derivatives
1299
MJLIPHIC_02592 CDS ROG0146 2727996 2728961 + NA
MJLIPHIC_02593 CDS COG2977 2728958 2729623 + [Q] Phosphopantetheinyl transferase component of siderophore synthetase
MJLIPHIC_02594 CDS COG0130 2729620 2730531 + [J] Pseudouridine synthase
1300
MJLIPHIC_02595 CDS COG1960 2730551 2731765 - [I] Acyl-CoA dehydrogenases
MJLIPHIC_02596 CDS COG2141 2731781 2732950 - [C] Coenzyme F420-dependent N5,N10-methylene tetrahydromethanopterin reductase and
MJLIPHIC_02597 CDS COG1321 2733015 2733668 - [K] Mn-dependent transcriptional regulator
1301
MJLIPHIC_02598 CDS COG0196 2733807 2734784 + [H] FAD synthase
MJLIPHIC_02599 CDS COG0184 2734886 2735155 + [J] Ribosomal protein S15P/S13E
1302
MJLIPHIC_02600 CDS NA 2735260 2735667 + NA
1303
MJLIPHIC_02601 CDS COG1185 2735997 2738288 + [J] Polyribonucleotide nucleotidyltransferase (polynucleotide phosphorylase)
MJLIPHIC_02602 CDS COG0612 2738266 2739615 + [R] Predicted Zn-dependent peptidases
MJLIPHIC_02603 CDS COG2367 2739634 2740515 + [V] Beta-lactamase class A
1304
MJLIPHIC_02604 CDS COG0686 2740567 2741682 - [E] Alanine dehydrogenase
1305
MJLIPHIC_02605 CDS COG1522 2741778 2742293 + [K] Transcriptional regulators
1306
MJLIPHIC_02606 CDS COG3187 2742304 2743065 - [O] Heat shock protein
1307
MJLIPHIC_02607 CDS NA 2743205 2743516 + NA
1308
MJLIPHIC_02608 CDS ROG4954 2743713 2745425 - NA
1309
MJLIPHIC_02609 CDS COG0289 2745690 2746427 + [E] Dihydrodipicolinate reductase
MJLIPHIC_02610 CDS ROG1252 2746424 2746891 + NA
MJLIPHIC_02611 CDS COG0655 2746888 2747343 + [R] Multimeric flavodoxin WrbA
MJLIPHIC_02612 CDS NA 2747354 2747815 + NA
1310
MJLIPHIC_02613 CDS COG2301 2747809 2748729 - [G] Citrate lyase beta subunit
MJLIPHIC_02614 CDS COG0412 2748751 2749500 - [Q] Dienelactone hydrolase and related enzymes
1311
MJLIPHIC_02615 CDS COG0207 2749543 2750343 + [F] Thymidylate synthase
MJLIPHIC_02616 CDS COG0262 2750366 2750836 + [H] Dihydrofolate reductase
MJLIPHIC_02617 CDS COG0318 2750910 2751554 + [IQ] Acyl-CoA synthetases (AMP-forming)/AMP-acid ligases II
1312
MJLIPHIC_02618 CDS COG2801 2751582 2751917 - [L] Transposase and inactivated derivatives
1313
MJLIPHIC_02619 CDS COG2826 2751938 2753338 + [L] Transposase and inactivated derivatives, IS30 family
1314
MJLIPHIC_02620 CDS COG2801 2753235 2753993 - [L] Transposase and inactivated derivatives
MJLIPHIC_02621 CDS COG2963 2753996 2754322 - [L] Transposase and inactivated derivatives
MJLIPHIC_02622 CDS COG2409 2754283 2755590 - [R] Predicted drug exporters of the RND
1315
MJLIPHIC_02623 CDS COG3214 2756022 2757218 + [S] Uncharacterized protein conserved in bacteria
1316
MJLIPHIC_02624 CDS COG0531 2757263 2758669 - [E] Amino acid transporters
MJLIPHIC_02625 CDS COG0596 2758699 2759628 - [R] Predicted hydrolases or acyltransferases (alpha/beta hydrolase
1317
MJLIPHIC_02626 CDS COG2186 2759690 2760487 + [K] Transcriptional regulators
MJLIPHIC_02627 CDS COG1351 2760518 2761270 + [F] Predicted alternative thymidylate synthase
MJLIPHIC_02628 CDS COG0329 2761290 2762198 + [EM] Dihydrodipicolinate synthase/N-acetylneuraminate lyase
MJLIPHIC_02629 CDS COG0595 2762206 2763882 + [R] Predicted hydrolase of the metallo-beta-lactamase superfamily
1318
MJLIPHIC_02630 CDS ROG2934 2763894 2764331 - NA
MJLIPHIC_02631 CDS COG1028 2764345 2765172 - [IQR] Dehydrogenases with different specificities (related to
MJLIPHIC_02632 CDS COG1359 2765195 2765509 - [S] Uncharacterized conserved protein
1319
MJLIPHIC_02633 CDS COG1674 2765674 2768304 + [D] DNA segregation ATPase FtsK/SpoIIIE and related
1320
MJLIPHIC_02634 CDS COG1246 2768315 2768860 - [E] N-acetylglutamate synthase and related acetyltransferases
1321
MJLIPHIC_02635 CDS COG0558 2768930 2769508 + [I] Phosphatidylglycerophosphate synthase
MJLIPHIC_02636 CDS COG1546 2769486 2769974 + [R] Uncharacterized protein (competence- and mitomycin-induced)
MJLIPHIC_02637 CDS COG1396 2770097 2770435 + [K] Predicted transcriptional regulators
1322
MJLIPHIC_02638 CDS COG1842 2770675 2771514 + [KT] Phage shock protein A (IM30), suppresses
MJLIPHIC_02639 CDS ROG5122 2771533 2772369 + NA

1323
MJLIPHIC_02640 CDS ROG3373 2772377 2772784 - NA
MJLIPHIC_02641 CDS COG4308 2772781 2773209 - [Q] Limonene-1,2-epoxide hydrolase
1324
MJLIPHIC_02642 CDS COG1819 2773253 2774425 + [GC] Glycosyl transferases, related to UDP-glucuronosyltransferase
MJLIPHIC_02643 CDS ROG0460 2774435 2774629 + NA
MJLIPHIC_02644 CDS COG0531 2774673 2774876 + [E] Amino acid transporters
1325
MJLIPHIC_02645 CDS COG0409 2774889 2776013 - [O] Hydrogenase maturation factor
MJLIPHIC_02646 CDS COG0298 2776054 2776338 - [O] Hydrogenase maturation factor
1326
MJLIPHIC_02647 CDS COG3547 2776595 2777317 + [L] Transposase and inactivated derivatives
MJLIPHIC_02648 CDS COG3547 2777331 2777801 + [L] Transposase and inactivated derivatives
1327
MJLIPHIC_02649 CDS COG0309 2777828 2778901 - [O] Hydrogenase maturation factor
MJLIPHIC_02650 CDS COG0279 2778898 2779581 - [G] Phosphoheptose isomerase
MJLIPHIC_02651 CDS ROG4798 2779578 2780315 - NA
MJLIPHIC_02652 CDS NA 2780312 2780503 - NA
MJLIPHIC_02653 CDS NA 2780500 2781228 - NA
MJLIPHIC_02654 CDS COG0068 2781233 2783524 - [O] Hydrogenase maturation factor
MJLIPHIC_02655 CDS COG0298 2783521 2783781 - [O] Hydrogenase maturation factor
MJLIPHIC_02656 CDS COG0680 2783783 2784265 - [C] Ni,Fe-hydrogenase maturation factor
MJLIPHIC_02657 CDS NA 2784269 2784388 - NA
MJLIPHIC_02658 CDS ROG4883 2784414 2785739 - NA
MJLIPHIC_02659 CDS ROG7608 2785736 2786392 - NA
MJLIPHIC_02660 CDS ROG5017 2786389 2786949 - NA
1328
MJLIPHIC_02661 CDS COG0694 2787037 2787960 - [O] Thioredoxin-like proteins and domains
MJLIPHIC_02662 CDS COG0374 2787964 2789760 - [C] Ni,Fe-hydrogenase I large subunit
MJLIPHIC_02663 CDS COG1740 2789863 2790918 - [C] Ni,Fe-hydrogenase I small subunit
MJLIPHIC_02664 CDS COG0378 2791028 2791807 - [OK] Ni2+-binding GTPase involved in regulation of
MJLIPHIC_02665 CDS COG0375 2791809 2792138 - [R] Zn finger protein HypA/HybF (possibly regulating
1329
MJLIPHIC_02666 CDS COG0468 2792363 2793412 + [L] RecA/RadA recombinase
MJLIPHIC_02667 CDS COG2137 2793381 2793947 + [R] Uncharacterized protein conserved in bacteria
1330
MJLIPHIC_02668 CDS COG0765 2794005 2794904 - [E] ABC-type amino acid transport system, permease
MJLIPHIC_02669 CDS COG0765 2794901 2795581 - [E] ABC-type amino acid transport system, permease
MJLIPHIC_02670 CDS COG0834 2795600 2796421 - [ET] ABC-type amino acid transport/signal transduction systems,
MJLIPHIC_02671 CDS COG1126 2796447 2797232 - [E] ABC-type polar amino acid transport system,
1331
MJLIPHIC_02672 CDS COG0621 2797337 2798875 + [J] 2-methylthioadenine synthetase
MJLIPHIC_02673 CDS ROG2231 2798872 2799285 + NA
MJLIPHIC_02674 CDS ROG2231 2799237 2799497 + NA
1332
MJLIPHIC_02675 CDS ROG0029 2799515 2800843 - NA
1333
MJLIPHIC_02676 CDS ROG0192 2801006 2801893 + NA
MJLIPHIC_02677 CDS ROG1580 2801903 2802589 + NA
MJLIPHIC_02678 CDS COG0324 2802586 2803494 + [J] tRNA delta(2)-isopentenylpyrophosphate transferase
MJLIPHIC_02679 CDS COG0253 2803499 2804374 + [E] Diaminopimelate epimerase
MJLIPHIC_02680 CDS COG2262 2804420 2805832 + [R] GTPases
1334
MJLIPHIC_02681 CDS NA 2805853 2807556 - NA
1335
MJLIPHIC_02682 CDS COG0243 2807642 2809933 - [C] Anaerobic dehydrogenases, typically selenocysteine-containing
1336
MJLIPHIC_02683 CDS COG5479 2810192 2812627 + [M] Uncharacterized protein potentially involved in peptidoglycan
1337
MJLIPHIC_02684 CDS COG1974 2812637 2813368 - [KT] SOS-response transcriptional repressors (RecA-mediated autopeptidases)
1338
MJLIPHIC_02685 CDS COG1388 2813632 2814189 + [M] FOG: LysM repeat
1339
MJLIPHIC_02686 CDS COG1327 2814351 2814803 + [K] Predicted transcriptional regulator, consists of a
1340
MJLIPHIC_02687 CDS COG0384 2814822 2815511 - [R] Predicted epimerase, PhzC/PhzF homolog
MJLIPHIC_02688 CDS COG0596 2815547 2816407 - [R] Predicted hydrolases or acyltransferases (alpha/beta hydrolase
1341
MJLIPHIC_02689 CDS ROG0141 2816586 2817632 - NA
1342
MJLIPHIC_02690 CDS ROG1163 2817834 2818490 + NA
MJLIPHIC_02691 CDS NA 2818487 2818666 + NA
1343
MJLIPHIC_02692 CDS COG1249 2818671 2820086 - [C] Pyruvate/2-oxoglutarate dehydrogenase complex, dihydrolipoamide dehydrogenase
(E3)
1344
MJLIPHIC_02693 CDS ROG1881 2820136 2821314 + NA
1345
MJLIPHIC_02694 CDS COG1321 2821344 2822036 - [K] Mn-dependent transcriptional regulator
1346
MJLIPHIC_02695 CDS COG0568 2822265 2823224 - [K] DNA-directed RNA polymerase, sigma subunit (sigma70/sigma32)
1347
MJLIPHIC_02696 CDS ROG0729 2823372 2823800 - NA
1348
MJLIPHIC_02697 CDS ROG0235 2823872 2824114 + NA
1349
MJLIPHIC_02698 CDS COG1295 2824118 2825110 - [S] Predicted membrane protein
1350
MJLIPHIC_02699 CDS ROG1247 2825183 2825374 - NA
1351
MJLIPHIC_02700 CDS COG3502 2825532 2825897 + [S] Uncharacterized protein conserved in bacteria
1352
MJLIPHIC_02701 CDS COG0568 2826225 2827625 - [K] DNA-directed RNA polymerase, sigma subunit (sigma70/sigma32)
1353
MJLIPHIC_02702 CDS COG1940 2827821 2828639 - [KG] Transcriptional regulator/sugar kinase
1354
MJLIPHIC_02703 CDS COG0483 2828700 2829659 + [G] Archaeal fructose-1,6-bisphosphatase and related enzymes of
1355
MJLIPHIC_02704 CDS ROG4753 2829633 2830283 - NA
1356

	MJLIPHIC_02705	CDS	ROG0095	2830449	2830751	+	NA
1357							
	MJLIPHIC_02706	CDS	ROG0347	2830772	2831284	-	NA
1358							
	MJLIPHIC_02707	CDS	COG0756	2831322	2831741	+	[F] dUTPase
	MJLIPHIC_02708	CDS	ROG0156	2831741	2832532	+	NA
1359							
	MJLIPHIC_02709	CDS	COG3571	2832575	2833273	-	[R] Predicted hydrolase of the alpha/beta-hydrolase fold
1360							
	MJLIPHIC_02710	CDS	ROG0994	2833409	2833780	+	NA
1361							
	MJLIPHIC_02711	CDS	ROG0326	2833867	2834520	+	NA
1362							
	MJLIPHIC_02712	CDS	COG0569	2834517	2835185	-	[P] K+ transport systems, NAD-binding component
	MJLIPHIC_02713	CDS	COG0569	2835223	2835897	-	[P] K+ transport systems, NAD-binding component
1363							
	MJLIPHIC_02714	CDS	COG0531	2836004	2837998	+	[E] Amino acid transporters
	MJLIPHIC_02715	CDS	COG2265	2837995	2839188	+	[J] SAM-dependent methyltransferases related to tRNA (uracil-5-)-methyltransferase
1364							
	MJLIPHIC_02716	CDS	NA	2839197	2840240	-	NA
1365							
	MJLIPHIC_02717	CDS	COG3004	2840636	2841931	+	[P] Na+/H+ antiporter
	MJLIPHIC_02718	CDS	COG1154	2841956	2843872	+	[HI] Deoxyxylulose-5-phosphate synthase
	MJLIPHIC_02719	CDS	COG0596	2843910	2844824	+	[R] Predicted hydrolases or acyltransferases (alpha/beta hydrolase
1366							
	MJLIPHIC_02720	CDS	COG0349	2844852	2846099	-	[J] Ribonuclease D
	MJLIPHIC_02721	CDS	ROG0154	2846102	2846638	-	NA
1367							
	MJLIPHIC_02722	CDS	COG0407	2846805	2847866	+	[H] Uroporphyrinogen-III decarboxylase
	MJLIPHIC_02723	CDS	COG1232	2847869	2849224	+	[H] Protoporphyrinogen oxidase
	MJLIPHIC_02724	CDS	COG3253	2849229	2849924	+	[S] Uncharacterized conserved protein
	MJLIPHIC_02725	CDS	NA	2849956	2850276	+	NA
1368							
	MJLIPHIC_02726	CDS	COG0229	2850324	2850737	-	[O] Conserved domain frequently associated with peptide
	MJLIPHIC_02727	CDS	ROG2061	2850789	2852081	-	NA
	MJLIPHIC_02728	CDS	COG0596	2852103	2853683	-	[R] Predicted hydrolases or acyltransferases (alpha/beta hydrolase
	MJLIPHIC_02729	CDS	COG1985	2853712	2854494	-	[H] Pyrimidine reductase, riboflavin biosynthesis
1369							
	MJLIPHIC_02730	CDS	COG1485	2854529	2855566	+	[R] Predicted ATPase
1370							
	MJLIPHIC_02731	CDS	COG0454	2855568	2856098	-	[KR] Histone acetyltransferase HPA2 and related acetyltransferases
	MJLIPHIC_02732	CDS	NA	2856103	2856636	-	NA
	MJLIPHIC_02733	CDS	ROG1744	2856675	2857100	-	NA
	MJLIPHIC_02734	CDS	COG0542	2857143	2857877	-	[O] ATPases with chaperone activity, ATP-binding subunit
1371							
	MJLIPHIC_02735	CDS	COG2205	2857997	2859376	+	[T] Osmosensitive K+ channel histidine kinase
1372							
	MJLIPHIC_02736	CDS	COG2186	2859380	2860057	-	[K] Transcriptional regulators
1373							
	MJLIPHIC_02737	CDS	COG2807	2860089	2861342	+	[P] Cyanate permease
	MJLIPHIC_02738	CDS	COG1275	2861362	2862573	+	[P] Tellurite resistance protein and related permeases
1374							
	MJLIPHIC_02739	CDS	COG0583	2862579	2863493	-	[K] Transcriptional regulator
1375							
	MJLIPHIC_02740	CDS	COG0722	2863788	2864840	+	[E] 3-deoxy-D-arabino-heptulosonate 7-phosphate (DAHP) synthase
1376							
	MJLIPHIC_02741	CDS	NA	2864837	2865649	-	NA
1377							
	MJLIPHIC_02742	CDS	COG0431	2865884	2866366	-	[R] Predicted flavoprotein
1378							
	MJLIPHIC_02743	CDS	NA	2867058	2867363	-	NA
	MJLIPHIC_02744	CDS	NA	2867398	2867781	-	NA
1379							
	MJLIPHIC_02745	CDS	COG4585	2867987	2869096	+	[T] Signal transduction histidine kinase
1380							
	MJLIPHIC_02746	CDS	NA	2869002	2869184	-	NA
1381							
	MJLIPHIC_02747	CDS	COG3335	2869279	2870340	-	[L] Transposase and inactivated derivatives
1382							
	MJLIPHIC_02748	CDS	COG2197	2870371	2870862	+	[TK] Response regulator containing a CheY-like receiver
1383							
	MJLIPHIC_02749	CDS	COG0300	2871045	2871920	+	[R] Short-chain dehydrogenases of various substrate specificities
	MJLIPHIC_02750	CDS	ROG0281	2871947	2872393	+	NA
	MJLIPHIC_02751	CDS	COG2814	2872390	2873601	+	[G] Arabinose efflux permease
1384							
	MJLIPHIC_02752	CDS	COG2141	2873675	2874541	-	[C] Coenzyme F420-dependent N5,N10-methylene tetrahydromethanopterin reductase and
	MJLIPHIC_02753	CDS	COG3000	2874564	2875424	-	[I] Sterol desaturase
1385							
	MJLIPHIC_02754	CDS	COG4585	2875590	2876747	+	[T] Signal transduction histidine kinase
	MJLIPHIC_02755	CDS	COG2197	2877135	2877413	+	[TK] Response regulator containing a CheY-like receiver
1386							
	MJLIPHIC_02756	CDS	NA	2877880	2878161	+	NA
1387							
	MJLIPHIC_02757	CDS	NA	2878366	2878581	+	NA
1388							
	MJLIPHIC_02758	CDS	NA	2879180	2880211	+	NA
	MJLIPHIC_02759	CDS	ROG2612	2880208	2880504	+	NA
1389							
	MJLIPHIC_02760	CDS	COG2801	2880573	2881502	-	[L] Transposase and inactivated derivatives
	MJLIPHIC_02761	CDS	COG2963	2881499	2881834	-	[L] Transposase and inactivated derivatives
1390							
	MJLIPHIC_02762	CDS	NA	2882092	2882865	+	NA
	MJLIPHIC_02763	CDS	NA	2882924	2884048	+	NA
1391							
	MJLIPHIC_02764	CDS	COG5421	2884840	2885700	-	[L] Transposase
1392							
	MJLIPHIC_02765	CDS	COG5421	2885824	2887335	+	[L] Transposase
1393							
	MJLIPHIC_02766	CDS	COG5421	2887291	2888505	-	[L] Transposase
1394							

	MJLIPHIC_02767	CDS	ROG2723	2888641	2888826	+	NA
1395							
	MJLIPHIC_02768	CDS	ROG5616	2888948	2890171	-	NA
1396							
	MJLIPHIC_02769	CDS	COG3464	2890828	2891739	+	[L] Transposase and inactivated derivatives
1397							
	MJLIPHIC_02770	CDS	ROG0016	2891747	2892499	-	NA
1398							
	MJLIPHIC_02771	CDS	COG3464	2892622	2893944	-	[L] Transposase and inactivated derivatives
1399							
	MJLIPHIC_02772	CDS	NA	2894143	2894352	-	NA
	MJLIPHIC_02773	CDS	NA	2894349	2894651	-	NA
	MJLIPHIC_02774	CDS	NA	2894648	2894974	-	NA
1400							
	MJLIPHIC_02775	tRNA	NA	2895086	2895160	-	NA
	MJLIPHIC_02776	tRNA	NA	2895172	2895245	-	NA
	MJLIPHIC_02777	tRNA	NA	2895273	2895346	-	NA
1401							
	MJLIPHIC_02778	tRNA	NA	2895813	2895887	+	NA
1402							
	MJLIPHIC_02779	CDS	COG0243	2896046	2898433	+	[C] Anaerobic dehydrogenases, typically selenocysteine-containing
	MJLIPHIC_02780	CDS	NA	2898445	2898927	+	NA
1403							
	MJLIPHIC_02781	CDS	COG2183	2898998	2901400	+	[K] Transcriptional accessory protein
1404							
	MJLIPHIC_02782	CDS	COG0398	2901404	2902102	-	[S] Uncharacterized conserved protein
	MJLIPHIC_02783	CDS	COG2141	2902133	2903542	-	[C] Coenzyme F420-dependent N5,N10-methylene tetrahydromethanopterin reductase and
	MJLIPHIC_02784	CDS	COG2141	2903622	2904917	-	[C] Coenzyme F420-dependent N5,N10-methylene tetrahydromethanopterin reductase and
	MJLIPHIC_02785	CDS	COG2141	2904914	2906269	-	[C] Coenzyme F420-dependent N5,N10-methylene tetrahydromethanopterin reductase and
	MJLIPHIC_02786	CDS	COG1123	2906266	2907879	-	[R] ATPase components of various ABC-type transport
	MJLIPHIC_02787	CDS	COG1173	2907894	2908766	-	[EP] ABC-type dipeptide/oligopeptide/nickel transport systems, permease components
	MJLIPHIC_02788	CDS	COG0601	2908763	2909731	-	[EP] ABC-type dipeptide/oligopeptide/nickel transport systems, permease components
	MJLIPHIC_02789	CDS	COG0747	2909734	2911320	-	[E] ABC-type dipeptide transport system, periplasmic component
1405							
	MJLIPHIC_02790	CDS	COG0477	2911537	2912946	-	[GEPR] Permeases of the major facilitator superfamily
1406							
	MJLIPHIC_02791	CDS	COG1733	2913003	2913422	+	[K] Predicted transcriptional regulators
1407							
	MJLIPHIC_02792	CDS	ROG1229	2913508	2913930	+	NA
1408							
	MJLIPHIC_02793	CDS	NA	2913944	2914423	-	NA
	MJLIPHIC_02794	CDS	COG1028	2914474	2915304	-	[IQR] Dehydrogenases with different specificities (related to
1409							
	MJLIPHIC_02795	CDS	COG0767	2915518	2916330	+	[Q] ABC-type transport system involved in resistance
	MJLIPHIC_02796	CDS	COG0767	2916342	2917187	+	[Q] ABC-type transport system involved in resistance
	MJLIPHIC_02797	CDS	COG1463	2917196	2918401	+	[Q] ABC-type transport system involved in resistance
	MJLIPHIC_02798	CDS	COG1463	2918398	2919429	+	[Q] ABC-type transport system involved in resistance
	MJLIPHIC_02799	CDS	COG1463	2919429	2920505	+	[Q] ABC-type transport system involved in resistance
	MJLIPHIC_02800	CDS	COG1463	2920508	2921965	+	[Q] ABC-type transport system involved in resistance
	MJLIPHIC_02801	CDS	COG1463	2921962	2923152	+	[Q] ABC-type transport system involved in resistance
	MJLIPHIC_02802	CDS	COG1463	2923152	2924639	+	[Q] ABC-type transport system involved in resistance
1410							
	MJLIPHIC_02803	CDS	NA	2924650	2925162	-	NA
1411							
	MJLIPHIC_02804	CDS	NA	2925323	2925682	+	NA
	MJLIPHIC_02805	CDS	NA	2925682	2926083	+	NA
1412							
	MJLIPHIC_02806	CDS	ROG5477	2926271	2926948	+	NA
	MJLIPHIC_02807	CDS	ROG8378	2926945	2927517	+	NA
1413							
	MJLIPHIC_02808	CDS	ROG3916	2927518	2928129	-	NA
	MJLIPHIC_02809	CDS	COG3573	2928126	2929694	-	[R] Predicted oxidoreductase
1414							
	MJLIPHIC_02810	CDS	COG1695	2929813	2930508	+	[K] Predicted transcriptional regulators
1415							
	MJLIPHIC_02811	CDS	COG1053	2930521	2932197	-	[C] Succinate dehydrogenase/fumarate reductase, flavoprotein subunit
1416							
	MJLIPHIC_02812	CDS	COG4638	2932334	2933494	+	[PR] Phenylpropionate dioxygenase and related ring-hydroxylating dioxygenases,
	MJLIPHIC_02813	CDS	ROG3284	2933491	2933769	+	NA
	MJLIPHIC_02814	CDS	COG0300	2933753	2934544	+	[R] Short-chain dehydrogenases of various substrate specificities
	MJLIPHIC_02815	CDS	COG1053	2934557	2936146	+	[C] Succinate dehydrogenase/fumarate reductase, flavoprotein subunit
1417							
	MJLIPHIC_02816	CDS	ROG5910	2936210	2937286	-	NA
1418							
	MJLIPHIC_02817	CDS	COG0657	2937393	2938373	-	[I] Esterase/lipase
	MJLIPHIC_02818	CDS	COG1028	2938382	2939260	-	[IQR] Dehydrogenases with different specificities (related to
	MJLIPHIC_02819	CDS	ROG2444	2939257	2939754	-	NA
1419							
	MJLIPHIC_02820	CDS	COG1960	2939825	2940826	-	[I] Acyl-CoA dehydrogenases
	MJLIPHIC_02821	CDS	COG1960	2940823	2941974	-	[I] Acyl-CoA dehydrogenases
	MJLIPHIC_02822	CDS	COG1024	2941974	2942756	-	[I] Enoyl-CoA hydratase/carnithine racemase
	MJLIPHIC_02823	CDS	COG1012	2942785	2944206	-	[C] NAD-dependent aldehyde dehydrogenases
	MJLIPHIC_02824	CDS	COG1063	2944225	2945340	-	[ER] Threonine dehydrogenase and related Zn-dependent dehydrogenases
	MJLIPHIC_02825	CDS	COG0318	2945429	2947012	-	[IQ] Acyl-CoA synthetases (AMP-forming)/AMP-acid ligases II
1420							
	MJLIPHIC_02826	CDS	COG2072	2947132	2949054	+	[P] Predicted flavoprotein involved in K+ transport
	MJLIPHIC_02827	CDS	COG1028	2949051	2949938	+	[IQR] Dehydrogenases with different specificities (related to
1421							
	MJLIPHIC_02828	CDS	ROG6868	2949940	2950560	-	NA
	MJLIPHIC_02829	CDS	ROG8202	2950566	2950952	-	NA
1422							
	MJLIPHIC_02830	CDS	COG1902	2951016	2952254	+	[C] NADH:flavin oxidoreductases, Old Yellow Enzyme family
1423							
	MJLIPHIC_02831	CDS	COG0657	2952197	2953147	-	[I] Esterase/lipase
	MJLIPHIC_02832	CDS	COG1414	2953144	2954028	-	[K] Transcriptional regulator
1424							
	MJLIPHIC_02833	CDS	NA	2954224	2955126	-	NA
	MJLIPHIC_02834	CDS	NA	2955143	2956324	-	NA
	MJLIPHIC_02835	CDS	COG1018	2956384	2957439	-	[C] Flavodoxin reductases (ferredoxin-NADPH reductases) family 1
1425							

MJLIPHIC_02836 CDS COG2072 2957617 2959194 + [P] Predicted flavoprotein involved in K+ transport
MJLIPHIC_02837 CDS COG0300 2959295 2960089 + [R] Short-chain dehydrogenases of various substrate specificities
MJLIPHIC_02838 CDS COG2141 2960091 2960960 + [C] Coenzyme F420-dependent N5,N10-methylene tetrahydromethanopterin reductase and
MJLIPHIC_02839 CDS COG3603 2961004 2961381 + [S] Uncharacterized conserved protein
1426
MJLIPHIC_02840 CDS COG1309 2961476 2962030 + [K] Transcriptional regulator
1427
MJLIPHIC_02841 CDS COG3967 2962139 2962834 + [M] Short-chain dehydrogenase involved in D-alanine esterification
1428
MJLIPHIC_02842 CDS ROG5709 2962927 2963490 + NA
1429
MJLIPHIC_02843 CDS COG0596 2963487 2964347 - [R] Predicted hydrolases or acyltransferases (alpha/beta hydrolase
1430
MJLIPHIC_02844 CDS COG0388 2964432 2965199 + [R] Predicted amidohydrolase
1431
MJLIPHIC_02845 CDS NA 2965162 2965305 - NA
1432
MJLIPHIC_02846 CDS COG3547 2965976 2967049 + [L] Transposase and inactivated derivatives
1433
MJLIPHIC_02847 CDS NA 2967055 2967447 - NA
1434
MJLIPHIC_02848 CDS COG1733 2967713 2968099 + [K] Predicted transcriptional regulators
1435
MJLIPHIC_02849 CDS NA 2968218 2969042 + NA
1436
MJLIPHIC_02850 CDS COG0246 2969066 2970517 - [G] Mannitol-1-phosphate/altronate dehydrogenases
MJLIPHIC_02851 CDS COG0524 2970514 2971422 - [G] Sugar kinases, ribokinase family
MJLIPHIC_02852 CDS COG4948 2971434 2972642 - [MR] L-alanine-DL-glutamate epimerase and related enzymes of
1437
MJLIPHIC_02853 CDS COG1802 2972708 2973415 + [K] Transcriptional regulators
1438
MJLIPHIC_02854 CDS COG0477 2973561 2974910 + [GEPR] Permeases of the major facilitator superfamily
MJLIPHIC_02855 CDS COG0477 2974935 2976311 + [GEPR] Permeases of the major facilitator superfamily
MJLIPHIC_02856 CDS COG1063 2976308 2977282 + [ER] Threonine dehydrogenase and related Zn-dependent dehydrogenases
1439
MJLIPHIC_02857 CDS COG0596 2977263 2978126 - [R] Predicted hydrolases or acyltransferases (alpha/beta hydrolase
MJLIPHIC_02858 CDS COG2205 2978144 2979652 - [T] Osmosensitive K+ channel histidine kinase
MJLIPHIC_02859 CDS COG0745 2979652 2980356 - [TK] Response regulators consisting of a CheY-like
1440
MJLIPHIC_02860 CDS NA 2980507 2981196 - NA
1441
MJLIPHIC_02861 CDS COG1028 2981309 2982106 - [IQR] Dehydrogenases with different specificities (related to
MJLIPHIC_02862 CDS COG1893 2982192 2983193 - [H] Ketopantoate reductase
1442
MJLIPHIC_02863 CDS COG0667 2983601 2984632 + [C] Predicted oxidoreductases (related to aryl-alcohol dehydrogenases)
MJLIPHIC_02864 CDS COG0778 2984629 2985426 + [C] Nitroreductase
1443
MJLIPHIC_02865 CDS ROG1751 2985445 2986239 - NA
1444
MJLIPHIC_02866 CDS COG1028 2986362 2987162 + [IQR] Dehydrogenases with different specificities (related to
1445
MJLIPHIC_02867 CDS COG1732 2987165 2988118 - [M] Periplasmic glycine betaine/choline-binding (lipo)protein of an
MJLIPHIC_02868 CDS COG1174 2988115 2988897 - [E] ABC-type proline/glycine betaine transport systems, permease
MJLIPHIC_02869 CDS COG1125 2988894 2990078 - [E] ABC-type proline/glycine betaine transport systems, ATPase
MJLIPHIC_02870 CDS COG1174 2990071 2990718 - [E] ABC-type proline/glycine betaine transport systems, permease
1446
MJLIPHIC_02871 CDS ROG3761 2990857 2991939 + NA
MJLIPHIC_02872 CDS ROG4581 2991982 2992650 + NA
MJLIPHIC_02873 CDS ROG1029 2992647 2993078 + NA
1447
MJLIPHIC_02874 CDS COG0441 2993163 2995217 + [J] Threonyl-tRNA synthetase
MJLIPHIC_02875 CDS COG0537 2995214 2995843 + [FGR] Diadenosine tetraphosphate (Ap4A) hydrolase and other
MJLIPHIC_02876 CDS ROG5552 2995840 2996508 + NA
MJLIPHIC_02877 CDS COG1560 2996505 2997419 + [M] Lauroyl/myristoyl acyltransferase
MJLIPHIC_02878 CDS COG0438 2997421 2998581 + [M] Glycosyltransferase
MJLIPHIC_02879 CDS ROG1793 2998581 2999636 + NA
1448
MJLIPHIC_02880 CDS COG0214 2999713 3000624 + [H] Pyridoxine biosynthesis enzyme
MJLIPHIC_02881 CDS COG1946 3000634 3001485 + [I] Acyl-CoA thioesterase
MJLIPHIC_02882 CDS COG0311 3001482 3002063 + [H] Predicted glutamine amidotransferase involved in pyridoxine
MJLIPHIC_02883 CDS COG0217 3002244 3002999 + [S] Uncharacterized conserved protein
1449
MJLIPHIC_02884 CDS COG0702 3003003 3003647 - [MG] Predicted nucleoside-diphosphate-sugar epimerases
MJLIPHIC_02885 CDS COG3565 3003678 3004109 - [R] Predicted dioxygenase of extradiol dioxygenase family
1450
MJLIPHIC_02886 CDS COG0817 3004304 3004852 + [L] Holliday junction resolvasome, endonuclease subunit
MJLIPHIC_02887 CDS COG0632 3004849 3005436 + [L] Holliday junction resolvasome, DNA-binding subunit
MJLIPHIC_02888 CDS COG2255 3005439 3006494 + [L] Holliday junction resolvasome, helicase subunit
MJLIPHIC_02889 CDS COG3759 3006499 3006885 + [S] Predicted membrane protein
1451
MJLIPHIC_02890 CDS COG1878 3007026 3007814 + [R] Predicted metal-dependent hydrolase
MJLIPHIC_02891 CDS COG1024 3007814 3008629 + [I] Enoyl-CoA hydratase/carnithine racemase
MJLIPHIC_02892 CDS COG1529 3008674 3011034 + [C] Aerobic-type carbon monoxide dehydrogenase, large subunit
MJLIPHIC_02893 CDS COG1319 3011031 3011849 + [C] Aerobic-type carbon monoxide dehydrogenase, middle subunit
MJLIPHIC_02894 CDS COG2080 3011842 3012327 + [C] Aerobic-type carbon monoxide dehydrogenase, small subunit
1452
MJLIPHIC_02895 CDS COG0477 3012396 3013715 + [GEPR] Permeases of the major facilitator superfamily
1453
MJLIPHIC_02896 CDS ROG1169 3013795 3014115 + NA
MJLIPHIC_02897 CDS COG1609 3014135 3015154 + [K] Transcriptional regulators
1454
MJLIPHIC_02898 CDS COG3631 3015273 3015719 + [R] Ketosteroid isomerase-related protein
1455
MJLIPHIC_02899 CDS COG3320 3015785 3019291 - [Q] Putative dehydrogenase domain of multifunctional non-ribosomal
1456
MJLIPHIC_02900 CDS ROG1436 3019572 3020213 - NA
1457
MJLIPHIC_02901 CDS NA 3020304 3021023 + NA
1458
MJLIPHIC_02902 CDS COG0160 3021057 3022397 - [E] 4-aminobutyrate aminotransferase and related aminotransferases

1459							
	MJLIPHIC_02903	CDS	COG1862	3022735	3023058	+	[U] Preprotein translocase subunit YajC
	MJLIPHIC_02904	CDS	COG0342	3023131	3024993	+	[U] Preprotein translocase subunit SecD
	MJLIPHIC_02905	CDS	COG0341	3024971	3026266	+	[U] Preprotein translocase subunit SecF
	MJLIPHIC_02906	CDS	COG0747	3026272	3027939	+	[E] ABC-type dipeptide transport system, periplasmic component
	MJLIPHIC_02907	CDS	COG0503	3027947	3028489	+	[F] Adenine/guanine phosphoribosyltransferases and related PRPP-binding proteins
	MJLIPHIC_02908	CDS	COG0317	3028533	3030926	+	[TK] Guanosine polyphosphate pyrophosphohydrolases/synthetases
1460							
	MJLIPHIC_02909	CDS	ROG6218	3030981	3032378	-	NA
	MJLIPHIC_02910	CDS	COG1853	3032378	3032989	-	[R] Conserved protein/domain typically associated with flavoprotein
1461							
	MJLIPHIC_02911	CDS	COG3665	3033121	3033750	+	[S] Uncharacterized conserved protein
	MJLIPHIC_02912	CDS	COG1902	3033747	3034880	+	[C] NADH:flavin oxidoreductases, Old Yellow Enzyme family
	MJLIPHIC_02913	CDS	COG0726	3034877	3035743	+	[G] Predicted xylanase/chitin deacetylase
1462							
	MJLIPHIC_02914	CDS	COG2508	3035748	3037301	-	[TQ] Regulator of polyketide synthase expression
1463							
	MJLIPHIC_02915	CDS	COG1457	3037408	3038829	+	[F] Purine-cytosine permease and related proteins
1464							
	MJLIPHIC_02916	CDS	COG0652	3038835	3039719	-	[O] Peptidyl-prolyl cis-trans isomerase (rotamase) - cyclophilin
	MJLIPHIC_02917	CDS	COG0652	3039885	3040748	-	[O] Peptidyl-prolyl cis-trans isomerase (rotamase) - cyclophilin
1465							
	MJLIPHIC_02918	CDS	COG0491	3040917	3041594	+	[R] Zn-dependent hydrolases, including glyoxylases
	MJLIPHIC_02919	CDS	COG0124	3041591	3042871	+	[J] Histidyl-tRNA synthetase
1466							
	MJLIPHIC_02920	CDS	COG0410	3043394	3044086	-	[E] ABC-type branched-chain amino acid transport systems,
	MJLIPHIC_02921	CDS	COG4674	3044086	3044898	-	[R] Uncharacterized ABC-type transport system, ATPase component
	MJLIPHIC_02922	CDS	COG4177	3044895	3046022	-	[E] ABC-type branched-chain amino acid transport system,
	MJLIPHIC_02923	CDS	COG0559	3046019	3046903	-	[E] Branched-chain amino acid ABC-type transport system,
	MJLIPHIC_02924	CDS	COG0683	3046939	3048207	-	[E] ABC-type branched-chain amino acid transport systems,
1467							
	MJLIPHIC_02925	CDS	NA	3048445	3048876	+	NA
1468							
	MJLIPHIC_02926	CDS	COG0596	3049055	3049870	-	[R] Predicted hydrolases or acyltransferases (alpha/beta hydrolase
	MJLIPHIC_02927	CDS	COG1951	3049867	3051504	-	[C] Tartrate dehydratase alpha subunit/Fumarate hydratase class
	MJLIPHIC_02928	CDS	COG0154	3051501	3052844	-	[J] Asp-tRNAAsn/Glu-tRNAGln amidotransferase A subunit and related
	MJLIPHIC_02929	CDS	NA	3052885	3053973	-	NA
	MJLIPHIC_02930	CDS	COG0388	3053983	3054882	-	[R] Predicted amidohydrolase
1469							
	MJLIPHIC_02931	CDS	COG1609	3055061	3056095	+	[K] Transcriptional regulators
1470							
	MJLIPHIC_02932	CDS	COG1533	3056145	3057179	+	[L] DNA repair photolyase
1471							
	MJLIPHIC_02933	CDS	ROG2472	3057281	3058564	+	NA
1472							
	MJLIPHIC_02934	CDS	COG1960	3058595	3059857	-	[I] Acyl-CoA dehydrogenases
	MJLIPHIC_02935	CDS	COG1960	3059854	3060993	-	[I] Acyl-CoA dehydrogenases
	MJLIPHIC_02936	CDS	COG2159	3061057	3062286	-	[R] Predicted metal-dependent hydrolase of the TIM-barrel
1473							
	MJLIPHIC_02937	CDS	COG1309	3062374	3063033	-	[K] Transcriptional regulator
1474							
	MJLIPHIC_02938	tRNA	NA	3063384	3063477	+	NA
1475							
	MJLIPHIC_02939	CDS	COG1946	3063977	3064876	+	[I] Acyl-CoA thioesterase
1476							
	MJLIPHIC_02940	CDS	ROG7597	3064877	3065413	-	NA
	MJLIPHIC_02941	CDS	COG4805	3065438	3067063	-	[S] Uncharacterized protein conserved in bacteria
	MJLIPHIC_02942	CDS	COG2321	3067056	3067949	-	[R] Predicted metalloprotease
1477							
	MJLIPHIC_02943	CDS	COG2272	3068022	3069497	+	[I] Carboxylesterase type B
1478							
	MJLIPHIC_02944	CDS	ROG4322	3069494	3070009	-	NA
1479							
	MJLIPHIC_02945	CDS	COG0173	3070138	3071934	+	[J] Aspartyl-tRNA synthetase
	MJLIPHIC_02946	CDS	ROG1229	3071978	3072433	+	NA
1480							
	MJLIPHIC_02947	CDS	COG1250	3072435	3073376	-	[I] 3-hydroxyacyl-CoA dehydrogenase
	MJLIPHIC_02948	CDS	COG1454	3073373	3074650	-	[C] Alcohol dehydrogenase, class IV
	MJLIPHIC_02949	CDS	COG1012	3074647	3076284	-	[C] NAD-dependent aldehyde dehydrogenases
1481							
	MJLIPHIC_02950	CDS	COG3284	3076326	3078044	+	[QK] Transcriptional activator of acetoin/glycerol metabolism
1482							
	MJLIPHIC_02951	CDS	COG1028	3078062	3078793	-	[IQR] Dehydrogenases with different specificities (related to
1483							
	MJLIPHIC_02952	CDS	COG0657	3078940	3079857	+	[I] Esterase/lipase
	MJLIPHIC_02953	CDS	COG2072	3079874	3081520	+	[P] Predicted flavoprotein involved in K+ transport
1484							
	MJLIPHIC_02954	CDS	COG0183	3081596	3082780	-	[I] Acetyl-CoA acetyltransferase
1485							
	MJLIPHIC_02955	CDS	COG1733	3082884	3083348	+	[K] Predicted transcriptional regulators
1486							
	MJLIPHIC_02956	CDS	COG3801	3083458	3083838	-	[S] Uncharacterized protein conserved in bacteria
	MJLIPHIC_02957	CDS	ROG2658	3083882	3084628	-	NA
1487							
	MJLIPHIC_02958	CDS	COG4196	3084783	3088091	+	[S] Uncharacterized protein conserved in bacteria
	MJLIPHIC_02959	CDS	COG2308	3088091	3090805	+	[S] Uncharacterized conserved protein
	MJLIPHIC_02960	CDS	COG1305	3090802	3091722	+	[E] Transglutaminase-like enzymes, putative cysteine proteases
	MJLIPHIC_02961	CDS	COG4307	3091715	3092743	+	[S] Uncharacterized protein conserved in bacteria
1488							
	MJLIPHIC_02962	CDS	ROG0316	3092925	3093758	+	NA
	MJLIPHIC_02963	CDS	COG2256	3093797	3095140	+	[L] ATPase related to the helicase subunit
1489							
	MJLIPHIC_02964	CDS	ROG2858	3095235	3095540	-	NA
1490							
	MJLIPHIC_02965	CDS	ROG4611	3095627	3096256	-	NA
1491							
	MJLIPHIC_02966	CDS	COG0432	3096381	3096782	+	[S] Uncharacterized conserved protein
	MJLIPHIC_02967	CDS	NA	3096782	3096874	+	NA
1492							
	MJLIPHIC_02968	CDS	COG0013	3096983	3099673	+	[J] Alanyl-tRNA synthetase

MJLIPHIC_02969 CDS COG0816 3099680 3100192 + [L] Predicted endonuclease involved in recombination (possible
MJLIPHIC_02970 CDS COG1559 3100185 3101429 + [R] Predicted periplasmic solute-binding protein
MJLIPHIC_02971 CDS COG0169 3101404 3102249 + [E] Shikimate 5-dehydrogenase
MJLIPHIC_02972 CDS COG1989 3102259 3102702 + [NOU] Type II secretory pathway, prepilin signal
MJLIPHIC_02973 CDS COG0082 3102792 3103991 + [E] Chorismate synthase
MJLIPHIC_02974 CDS COG0703 3103991 3104725 + [E] Shikimate kinase
MJLIPHIC_02975 CDS COG0337 3104718 3105851 + [E] 3-dehydroquinate synthetase
1493
MJLIPHIC_02976 CDS ROG5945 3105842 3106417 - NA
1494
MJLIPHIC_02977 CDS COG0006 3106514 3107587 + [E] Xaa-Pro aminopeptidase
MJLIPHIC_02978 CDS COG0231 3107613 3108176 + [J] Translation elongation factor P (EF-P)/translation initiation
MJLIPHIC_02979 CDS COG0781 3108169 3108651 + [K] Transcription termination factor
1495
MJLIPHIC_02980 CDS COG2771 3109284 3111491 + [K] DNA-binding HTH domain-containing proteins
MJLIPHIC_02981 CDS NA 3111537 3111776 + NA
1496
MJLIPHIC_02982 CDS COG0654 3111781 3112989 - [HC] 2-polyprenyl-6-methoxyphenol hydroxylase and related FAD-dependent oxidoreductases
MJLIPHIC_02983 CDS COG1680 3113057 3114259 - [V] Beta-lactamase class C and other penicillin
MJLIPHIC_02984 CDS COG0500 3114301 3114978 - [QR] SAM-dependent methyltransferases
1497
MJLIPHIC_02985 CDS COG2065 3115075 3115632 + [F] Pyrimidine operon attenuation protein/uracil phosphoribosyltransferase
MJLIPHIC_02986 CDS COG0540 3115629 3116585 + [F] Aspartate carbamoyltransferase, catalytic chain
MJLIPHIC_02987 CDS COG0044 3116582 3117916 + [F] Dihydroorotase and related cyclic amidohydrolases
MJLIPHIC_02988 CDS ROG1472 3117913 3118413 + NA
MJLIPHIC_02989 CDS COG0505 3118410 3119558 + [EF] Carbamoylphosphate synthase small subunit
MJLIPHIC_02990 CDS COG0458 3119664 3123011 + [EF] Carbamoylphosphate synthase large subunit (split gene
MJLIPHIC_02991 CDS NA 3123008 3123838 + NA
1498
MJLIPHIC_02992 CDS ROG0332 3124973 3125290 + NA
1499
MJLIPHIC_02993 CDS COG0194 3125400 3125903 + [F] Guanylate kinase
MJLIPHIC_02994 CDS COG1758 3126019 3126342 + [K] DNA-directed RNA polymerase, subunit K/omega
MJLIPHIC_02995 CDS COG0452 3126361 3127605 + [H] Phosphopantothenoylcysteine synthetase/decarboxylase
MJLIPHIC_02996 CDS COG0192 3127746 3128945 + [H] S-adenosylmethionine synthetase
MJLIPHIC_02997 CDS COG1135 3129096 3130088 + [P] ABC-type metal ion transport system, ATPase
MJLIPHIC_02998 CDS COG2011 3130085 3130825 + [P] ABC-type metal ion transport system, permease
MJLIPHIC_02999 CDS COG1464 3130870 3131709 + [P] ABC-type metal ion transport system, periplasmic
1500
MJLIPHIC_03000 CDS COG0657 3131786 3132673 - [I] Esterase/lipase
1501
MJLIPHIC_03001 CDS COG3714 3132696 3133439 + [S] Predicted membrane protein
MJLIPHIC_03002 CDS COG1198 3133490 3135436 + [L] Primosomal protein N' (replication factor Y)
MJLIPHIC_03003 CDS ROG1462 3135497 3137305 + NA
MJLIPHIC_03004 CDS COG1704 3137302 3137835 + [S] Uncharacterized conserved protein
MJLIPHIC_03005 CDS COG0223 3137866 3138804 + [J] Methionyl-tRNA formyltransferase
MJLIPHIC_03006 CDS COG0144 3138801 3140195 + [J] tRNA and rRNA cytosine-C5-methylases
MJLIPHIC_03007 CDS COG0036 3140241 3140918 + [G] Pentose-5-phosphate-3-epimerase
MJLIPHIC_03008 CDS COG1985 3140939 3141922 + [H] Pyrimidine reductase, riboflavin biosynthesis
1502
MJLIPHIC_03009 CDS NA 3142059 3142244 + NA
1503
MJLIPHIC_03010 CDS COG0477 3142257 3143957 - [GEPR] Permeases of the major facilitator superfamily
MJLIPHIC_03011 CDS ROG4873 3143957 3144667 - NA
1504
MJLIPHIC_03012 CDS COG0307 3144739 3145338 + [H] Riboflavin synthase alpha chain
MJLIPHIC_03013 CDS COG0108 3145459 3146778 + [H] 3,4-dihydroxy-2-butanone 4-phosphate synthase
MJLIPHIC_03014 CDS COG0054 3146775 3147251 + [H] Riboflavin synthase beta-chain
MJLIPHIC_03015 CDS ROG0863 3147254 3147721 + NA
1505
MJLIPHIC_03016 CDS COG0456 3147737 3148219 - [R] Acetyltransferases
MJLIPHIC_03017 CDS COG0405 3148216 3150171 - [E] Gamma-glutamyltransferase
1506
MJLIPHIC_03018 CDS COG0322 3150228 3152348 + [L] Nuclease subunit of the excinuclease complex
MJLIPHIC_03019 CDS COG1660 3152380 3153321 + [R] Predicted P-loop-containing kinase
MJLIPHIC_03020 CDS COG0391 3153357 3154382 + [S] Uncharacterized conserved protein
MJLIPHIC_03021 CDS COG1481 3154373 3155356 + [S] Uncharacterized protein conserved in bacteria
1507
MJLIPHIC_03022 CDS ROG0051 3155445 3155837 + NA
MJLIPHIC_03023 CDS COG1090 3155830 3157173 + [R] Predicted nucleoside-diphosphate sugar epimerase
1508
MJLIPHIC_03024 CDS COG0057 3157277 3158299 + [G] Glyceraldehyde-3-phosphate dehydrogenase/erythrose-4-phosphate dehydrogenase
MJLIPHIC_03025 CDS COG0126 3158305 3159531 + [G] 3-phosphoglycerate kinase
MJLIPHIC_03026 CDS COG0149 3159535 3160320 + [G] Triosephosphate isomerase
MJLIPHIC_03027 CDS COG1314 3160365 3160601 + [U] Preprotein translocase subunit SecG
1509
MJLIPHIC_03028 CDS COG5449 3160719 3161159 + [S] Uncharacterized conserved protein
MJLIPHIC_03029 CDS NA 3161193 3161813 + NA
1510
MJLIPHIC_03030 CDS COG0274 3161830 3162525 - [F] Deoxyribose-phosphate aldolase
MJLIPHIC_03031 CDS COG1172 3162550 3163551 - [G] Ribose/xylose/arabinose/galactoside ABC-type transport systems, permease components
MJLIPHIC_03032 CDS COG1129 3163544 3165091 - [G] ABC-type sugar transport system, ATPase component
MJLIPHIC_03033 CDS COG2390 3165093 3166037 - [K] Transcriptional regulator, contains sigma factor-related N-terminal
MJLIPHIC_03034 CDS COG1069 3166037 3167596 - [C] Ribulose kinase
MJLIPHIC_03035 CDS COG1063 3167600 3168691 - [ER] Threonine dehydrogenase and related Zn-dependent dehydrogenases
MJLIPHIC_03036 CDS COG1879 3168749 3169729 - [G] ABC-type sugar transport system, periplasmic component
1511
MJLIPHIC_03037 CDS COG2352 3169942 3172743 + [C] Phosphoenolpyruvate carboxylase
1512
MJLIPHIC_03038 CDS ROG6046 3172746 3173117 - NA
MJLIPHIC_03039 CDS COG0363 3173171 3173905 - [G] 6-phosphogluconolactonase/Glucosamine-6-phosphate isomerase/deaminase
MJLIPHIC_03040 CDS COG3429 3173902 3174813 - [G] Glucose-6-P dehydrogenase subunit
MJLIPHIC_03041 CDS COG0364 3174830 3176380 - [G] Glucose-6-phosphate 1-dehydrogenase
MJLIPHIC_03042 CDS COG0176 3176377 3177492 - [G] Transaldolase
MJLIPHIC_03043 CDS COG0021 3177527 3179617 - [G] Transketolase
1513
MJLIPHIC_03044 CDS COG0109 3179862 3180788 + [O] Polyprenyltransferase (cytochrome oxidase assembly factor)
1514

	MJLIPHIC_03045	CDS	COG0604	3180841	3181869	-	[CR] NADPH:quinone reductase and related Zn-dependent oxidoreductases
1515							
	MJLIPHIC_03046	CDS	ROG0435	3181900	3182799	+	NA
1516							
	MJLIPHIC_03047	CDS	COG3839	3182812	3183879	-	[G] ABC-type sugar transport systems, ATPase components
	MJLIPHIC_03048	CDS	COG0395	3183900	3184715	-	[G] ABC-type sugar transport system, permease component
	MJLIPHIC_03049	CDS	COG1175	3184712	3185575	-	[G] ABC-type sugar transport systems, permease components
	MJLIPHIC_03050	CDS	COG1653	3185616	3186944	-	[G] ABC-type sugar transport system, periplasmic component
	MJLIPHIC_03051	CDS	COG1028	3187000	3187794	-	[IQR] Dehydrogenases with different specificities (related to
	MJLIPHIC_03052	CDS	COG1070	3187791	3189278	-	[G] Sugar (pentulose and hexulose) kinases
1517							
	MJLIPHIC_03053	CDS	COG0434	3189456	3190271	+	[R] Predicted TIM-barrel enzyme
	MJLIPHIC_03054	CDS	COG0483	3190268	3191017	+	[G] Archaeal fructose-1,6-bisphosphatase and related enzymes of
1518							
	MJLIPHIC_03055	CDS	COG1349	3191000	3191800	-	[KG] Transcriptional regulators of sugar metabolism
1519							
	MJLIPHIC_03056	CDS	COG1612	3191877	3192839	-	[O] Uncharacterized protein required for cytochrome oxidase
	MJLIPHIC_03057	CDS	NA	3192884	3193669	-	NA
	MJLIPHIC_03058	CDS	COG1131	3193666	3194634	-	[V] ABC-type multidrug transport system, ATPase component
	MJLIPHIC_03059	CDS	ROG0866	3194631	3196307	-	NA
1520							
	MJLIPHIC_03060	CDS	COG2345	3196493	3197227	+	[K] Predicted transcriptional regulator
	MJLIPHIC_03061	CDS	COG0719	3197224	3198657	+	[O] ABC-type transport system involved in Fe-S
	MJLIPHIC_03062	CDS	COG0719	3198660	3199871	+	[O] ABC-type transport system involved in Fe-S
	MJLIPHIC_03063	CDS	COG0396	3199871	3200644	+	[O] ABC-type transport system involved in Fe-S
	MJLIPHIC_03064	CDS	COG0520	3200647	3201891	+	[E] Selenocysteine lyase
	MJLIPHIC_03065	CDS	COG0822	3201903	3202382	+	[C] NifU homolog involved in Fe-S cluster
	MJLIPHIC_03066	CDS	COG2151	3202379	3202720	+	[R] Predicted metal-sulfur cluster biosynthetic enzyme
1521							
	MJLIPHIC_03067	CDS	NA	3202721	3203860	-	NA
	MJLIPHIC_03068	CDS	ROG6516	3203857	3204447	-	NA
	MJLIPHIC_03069	CDS	NA	3204495	3204944	-	NA
	MJLIPHIC_03070	CDS	NA	3204893	3205126	-	NA
	MJLIPHIC_03071	CDS	COG1022	3205123	3206808	-	[I] Long-chain acyl-CoA synthetases (AMP-forming)
1522							
	MJLIPHIC_03072	CDS	NA	3207089	3207892	+	NA
1523							
	MJLIPHIC_03073	CDS	COG2124	3208009	3209274	+	[Q] Cytochrome P450
	MJLIPHIC_03074	CDS	NA	3209220	3209747	+	NA
1524							
	MJLIPHIC_03075	CDS	NA	3209887	3210606	+	NA
	MJLIPHIC_03076	CDS	COG1902	3210665	3211774	+	[C] NADH:flavin oxidoreductases, Old Yellow Enzyme family
	MJLIPHIC_03077	CDS	COG0526	3211820	3212194	+	[OC] Thiol-disulfide isomerase and thioredoxins
	MJLIPHIC_03078	CDS	COG1024	3212297	3213070	+	[I] Enoyl-CoA hydratase/carnithine racemase
	MJLIPHIC_03079	CDS	COG0488	3213101	3214729	+	[R] ATPase components of ABC transporters with
1525							
	MJLIPHIC_03080	CDS	ROG1050	3214859	3215056	+	NA
1526							
	MJLIPHIC_03081	CDS	COG1309	3215094	3215708	-	[K] Transcriptional regulator
	MJLIPHIC_03082	CDS	COG1048	3215760	3218591	-	[C] Aconitase A
1527							
	MJLIPHIC_03083	CDS	ROG5700	3218869	3219456	+	NA
1528							
	MJLIPHIC_03084	CDS	COG0791	3219661	3221154	+	[M] Cell wall-associated hydrolases (invasion-associated proteins)
	MJLIPHIC_03085	CDS	COG0791	3221165	3221863	+	[M] Cell wall-associated hydrolases (invasion-associated proteins)
1529							
	MJLIPHIC_03086	CDS	COG0714	3222056	3223219	+	[R] MoxR-like ATPases
	MJLIPHIC_03087	CDS	COG1721	3223216	3224163	+	[R] Uncharacterized conserved protein (some members contain
	MJLIPHIC_03088	CDS	COG2304	3224216	3225223	+	[R] Uncharacterized protein containing a von Willebrand
1530							
	MJLIPHIC_03089	CDS	COG1028	3225346	3226113	+	[IQR] Dehydrogenases with different specificities (related to
	MJLIPHIC_03090	CDS	COG0623	3226161	3226970	+	[I] Enoyl-[acyl-carrier-protein] reductase (NADH)
	MJLIPHIC_03091	CDS	COG0276	3226977	3227999	+	[H] Protoheme ferro-lyase (ferrochelatase)
1531							
	MJLIPHIC_03092	CDS	ROG1457	3228033	3228881	-	NA
1532							
	MJLIPHIC_03093	CDS	COG1585	3228967	3229401	+	[OU] Membrane protein implicated in regulation of
	MJLIPHIC_03094	CDS	COG0330	3229445	3230671	+	[O] Membrane protease subunits, stomatin/prohibitin homologs
	MJLIPHIC_03095	CDS	ROG5036	3230705	3231052	+	NA
1533							
	MJLIPHIC_03096	CDS	COG0398	3231067	3231804	-	[S] Uncharacterized conserved protein
1534							
	MJLIPHIC_03097	CDS	COG1884	3231961	3233832	+	[I] Methylmalonyl-CoA mutase, N-terminal domain/subunit
	MJLIPHIC_03098	CDS	COG1884	3233834	3236089	+	[I] Methylmalonyl-CoA mutase, N-terminal domain/subunit
	MJLIPHIC_03099	CDS	COG1703	3236097	3237074	+	[E] Putative periplasmic protein kinase ArgK and
	MJLIPHIC_03100	CDS	NA	3237127	3237477	+	NA
1535							
	MJLIPHIC_03101	CDS	COG1680	3237553	3238818	+	[V] Beta-lactamase class C and other penicillin
1536							
	MJLIPHIC_03102	CDS	COG3307	3238864	3239595	-	[M] Lipid A core - O-antigen ligase
	MJLIPHIC_03103	CDS	NA	3239637	3239999	-	NA
1537							
	MJLIPHIC_03104	CDS	ROG0016	3240190	3240942	+	NA
1538							
	MJLIPHIC_03105	CDS	COG3464	3240950	3241861	-	[L] Transposase and inactivated derivatives
1539							
	MJLIPHIC_03106	CDS	NA	3242433	3242996	+	NA
1540							
	MJLIPHIC_03107	CDS	COG0060	3243269	3246391	+	[J] Isoleucyl-tRNA synthetase
1541							
	MJLIPHIC_03108	CDS	COG2501	3246446	3246664	-	[S] Uncharacterized conserved protein
1542							
	MJLIPHIC_03109	CDS	COG0389	3246698	3248089	+	[L] Nucleotidyltransferase/DNA polymerase involved in DNA repair
1543							
	MJLIPHIC_03110	CDS	NA	3248071	3248559	-	NA
	MJLIPHIC_03111	CDS	COG0252	3248591	3249553	-	[EJ] L-asparaginase/archaeal Glu-tRNAGln amidotransferase subunit D
1544							
	MJLIPHIC_03112	CDS	COG0597	3249629	3250303	+	[MU] Lipoprotein signal peptidase
	MJLIPHIC_03113	CDS	COG0564	3250318	3251232	+	[J] Pseudouridylate synthases, 23S RNA-specific
	MJLIPHIC_03114	CDS	COG2962	3251229	3252143	+	[R] Predicted permeases

1545							
	MJLIPHIC_03115	CDS	COG1309	3252358	3252924	+	[K] Transcriptional regulator
1546							
	MJLIPHIC_03116	CDS	COG0587	3253073	3256561	+	[L] DNA polymerase III, alpha subunit
1547							
	MJLIPHIC_03117	CDS	ROG0988	3256584	3257036	-	NA
1548							
	MJLIPHIC_03118	CDS	COG0789	3257094	3257483	+	[K] Predicted transcriptional regulators
	MJLIPHIC_03119	CDS	ROG1588	3257483	3257905	+	NA
1549							
	MJLIPHIC_03120	CDS	ROG6011	3257910	3258143	-	NA
1550							
	MJLIPHIC_03121	CDS	COG1171	3258478	3259674	+	[E] Threonine dehydratase
1551							
	MJLIPHIC_03122	CDS	COG1523	3259770	3261521	-	[G] Type II secretory pathway, pullulanase PulA
	MJLIPHIC_03123	CDS	COG3280	3261518	3262867	-	[G] Maltooligosyl trehalose synthase
	MJLIPHIC_03124	CDS	COG3280	3262869	3263795	-	[G] Maltooligosyl trehalose synthase
	MJLIPHIC_03125	CDS	COG1523	3263798	3265921	-	[G] Type II secretory pathway, pullulanase PulA
	MJLIPHIC_03126	CDS	COG1835	3265959	3268055	-	[I] Predicted acyltransferases
1552							
	MJLIPHIC_03127	CDS	COG0161	3268212	3269510	+	[H] Adenosylmethionine-8-amino-7-oxononanoate aminotransferase
	MJLIPHIC_03128	CDS	COG0156	3269639	3270787	+	[H] 7-keto-8-aminopelargonate synthetase and related enzymes
	MJLIPHIC_03129	CDS	COG0132	3270784	3271461	+	[H] Dethiobiotin synthetase
	MJLIPHIC_03130	CDS	ROG3306	3271486	3271995	+	NA
	MJLIPHIC_03131	CDS	ROG2730	3272008	3272769	+	NA
1553							
	MJLIPHIC_03132	CDS	COG1309	3272770	3273387	-	[K] Transcriptional regulator
1554							
	MJLIPHIC_03133	CDS	COG0502	3273453	3274514	+	[H] Biotin synthase and related enzymes
	MJLIPHIC_03134	CDS	ROG3017	3274527	3274763	+	NA
	MJLIPHIC_03135	CDS	ROG6910	3274760	3275455	+	NA
1555							
	MJLIPHIC_03136	CDS	ROG4108	3275485	3276816	-	NA
	MJLIPHIC_03137	CDS	COG1051	3276859	3277551	-	[F] ADP-ribose pyrophosphatase
1556							
	MJLIPHIC_03138	CDS	COG0379	3277598	3278641	+	[H] Quinolinate synthase
	MJLIPHIC_03139	CDS	COG0029	3278638	3280233	+	[H] Aspartate oxidase
	MJLIPHIC_03140	CDS	COG0157	3280233	3281093	+	[H] Nicotinate-nucleotide pyrophosphorylase
1557							
	MJLIPHIC_03141	CDS	COG3794	3281139	3282680	-	[C] Plastocyanin
1558							
	MJLIPHIC_03142	CDS	COG1280	3283047	3283682	-	[E] Putative threonine efflux protein
	MJLIPHIC_03143	CDS	ROG8109	3283704	3284072	-	NA
1559							
	MJLIPHIC_03144	CDS	COG0141	3284206	3285537	+	[E] Histidinol dehydrogenase
	MJLIPHIC_03145	CDS	COG0079	3285537	3286676	+	[E] Histidinol-phosphate/aromatic aminotransferase and cobyric acid decarboxylase
	MJLIPHIC_03146	CDS	COG0131	3286673	3287293	+	[E] Imidazoleglycerol-phosphate dehydratase
	MJLIPHIC_03147	CDS	COG0118	3287290	3287907	+	[E] Glutamine amidotransferase
	MJLIPHIC_03148	CDS	COG0106	3287904	3288698	+	[E] Phosphoribosylformimino-5-aminoimidazole carboxamide ribonucleotide (ProFAR) isomerase
	MJLIPHIC_03149	CDS	COG0483	3288695	3289525	+	[G] Archaeal fructose-1,6-bisphosphatase and related enzymes of
	MJLIPHIC_03150	CDS	COG0107	3289522	3290307	+	[E] Imidazoleglycerol-phosphate synthase
	MJLIPHIC_03151	CDS	COG0139	3290318	3290665	+	[E] Phosphoribosyl-AMP cyclohydrolase
	MJLIPHIC_03152	CDS	COG0827	3290670	3292352	+	[L] Adenine-specific DNA methylase
	MJLIPHIC_03153	CDS	NA	3292342	3293334	+	NA
1560							
	MJLIPHIC_03154	CDS	COG1119	3293342	3294139	-	[P] ABC-type molybdenum transport system, ATPase component/photorepair
1561							
	MJLIPHIC_03155	CDS	COG1225	3294257	3294721	-	[O] Peroxiredoxin
1562							
	MJLIPHIC_03156	CDS	COG0147	3294784	3296358	+	[EH] Anthranilate/para-aminobenzoate synthases component I
	MJLIPHIC_03157	CDS	ROG4119	3296355	3296969	+	NA
1563							
	MJLIPHIC_03158	CDS	COG0134	3297046	3297864	+	[E] Indole-3-glycerol phosphate synthase
	MJLIPHIC_03159	CDS	COG0133	3297900	3299177	+	[E] Tryptophan synthase beta chain
	MJLIPHIC_03160	CDS	COG0159	3299174	3299962	+	[E] Tryptophan synthase alpha chain
	MJLIPHIC_03161	CDS	COG0682	3299959	3301809	+	[M] Prolipoprotein diacylglyceryltransferase
	MJLIPHIC_03162	CDS	COG2314	3301834	3302223	+	[S] Predicted membrane protein
	MJLIPHIC_03163	CDS	ROG0651	3302213	3302626	+	NA
1564							
	MJLIPHIC_03164	CDS	COG0069	3302712	3307268	+	[E] Glutamate synthase domain 2
	MJLIPHIC_03165	CDS	COG0493	3307261	3308763	+	[ER] NADPH-dependent glutamate synthase beta chain and
	MJLIPHIC_03166	CDS	COG0469	3308887	3310221	+	[G] Pyruvate kinase
	MJLIPHIC_03167	CDS	COG1946	3310245	3311108	+	[I] Acyl-CoA thioesterase
1565							
	MJLIPHIC_03168	CDS	ROG2222	3311187	3311930	-	NA
	MJLIPHIC_03169	CDS	COG4987	3311941	3313326	-	[CO] ABC-type transport system involved in cytochrome
	MJLIPHIC_03170	CDS	COG4988	3313470	3314999	-	[CO] ABC-type transport system involved in cytochrome
	MJLIPHIC_03171	CDS	COG1294	3315095	3316147	-	[C] Cytochrome bd-type quinol oxidase, subunit 2
	MJLIPHIC_03172	CDS	COG1271	3316161	3317624	-	[C] Cytochrome bd-type quinol oxidase, subunit 1
1566							
	MJLIPHIC_03173	CDS	COG3247	3317720	3318319	-	[S] Uncharacterized conserved protein
1567							
	MJLIPHIC_03174	CDS	COG0834	3318528	3319415	+	[ET] ABC-type amino acid transport/signal transduction systems,
	MJLIPHIC_03175	CDS	COG0765	3319425	3320357	+	[E] ABC-type amino acid transport system, permease
	MJLIPHIC_03176	CDS	COG1126	3320354	3321139	+	[E] ABC-type polar amino acid transport system,
	MJLIPHIC_03177	CDS	COG2897	3321162	3322022	+	[P] Rhodanese-related sulfurtransferase
	MJLIPHIC_03178	CDS	COG4585	3322077	3323213	+	[T] Signal transduction histidine kinase
	MJLIPHIC_03179	CDS	COG2197	3323213	3323869	+	[TK] Response regulator containing a CheY-like receiver
	MJLIPHIC_03180	CDS	COG5373	3323913	3325754	+	[S] Predicted membrane protein
1568							
	MJLIPHIC_03181	CDS	COG0783	3325818	3326303	+	[P] DNA-binding ferritin-like protein (oxidative damage protectant)
1569							
	MJLIPHIC_03182	CDS	COG2114	3326308	3327105	-	[T] Adenylate cyclase, family 3 (some proteins
1570							
	MJLIPHIC_03183	CDS	NA	3327461	3328519	+	NA
1571							
	MJLIPHIC_03184	tRNA	NA	3328690	3328766	-	NA
1572							
	MJLIPHIC_03185	CDS	COG3707	3328852	3329478	+	[T] Response regulator with putative antiterminator output

1573							
	MJLIPHIC_03186	CDS	COG0683	3329708	3330919	+	[E] ABC-type branched-chain amino acid transport systems,
	MJLIPHIC_03187	CDS	COG0559	3330998	3332035	+	[E] Branched-chain amino acid ABC-type transport system,
	MJLIPHIC_03188	CDS	COG4177	3332032	3333246	+	[E] ABC-type branched-chain amino acid transport system,
	MJLIPHIC_03189	CDS	COG0411	3333243	3334097	+	[E] ABC-type branched-chain amino acid transport systems,
	MJLIPHIC_03190	CDS	COG0410	3334081	3334827	+	[E] ABC-type branched-chain amino acid transport systems,
1574							
	MJLIPHIC_03191	CDS	COG1742	3334948	3335274	+	[S] Uncharacterized conserved protein
1575							
	MJLIPHIC_03192	CDS	ROG3869	3335279	3336217	-	NA
1576							
	MJLIPHIC_03193	CDS	COG1714	3336282	3336767	-	[S] Predicted membrane protein/domain
	MJLIPHIC_03194	CDS	ROG2252	3336777	3337652	-	NA
1577							
	MJLIPHIC_03195	CDS	COG1070	3337958	3339346	+	[G] Sugar (pentulose and hexulose) kinases
1578							
	MJLIPHIC_03196	CDS	NA	3339524	3340744	+	NA
	MJLIPHIC_03197	CDS	COG0583	3340778	3341722	+	[K] Transcriptional regulator
	MJLIPHIC_03198	CDS	ROG4656	3341757	3342605	+	NA
	MJLIPHIC_03199	CDS	COG1070	3342632	3344113	+	[G] Sugar (pentulose and hexulose) kinases
1579							
	MJLIPHIC_03200	CDS	COG1028	3344098	3344886	-	[IQR] Dehydrogenases with different specificities (related to
	MJLIPHIC_03201	CDS	COG2390	3344883	3345905	-	[K] Transcriptional regulator, contains sigma factor-related N-terminal
1580							
	MJLIPHIC_03202	CDS	COG1063	3346024	3347112	+	[ER] Threonine dehydrogenase and related Zn-dependent dehydrogenases
	MJLIPHIC_03203	CDS	COG1653	3347178	3348557	+	[G] ABC-type sugar transport system, periplasmic component
	MJLIPHIC_03204	CDS	COG1175	3348569	3349522	+	[G] ABC-type sugar transport systems, permease components
	MJLIPHIC_03205	CDS	COG0395	3349525	3350397	+	[G] ABC-type sugar transport system, permease component
	MJLIPHIC_03206	CDS	COG3839	3350397	3351506	+	[G] ABC-type sugar transport systems, ATPase components
	MJLIPHIC_03207	CDS	COG3839	3351503	3352636	+	[G] ABC-type sugar transport systems, ATPase components
	MJLIPHIC_03208	CDS	COG2376	3352633	3354375	+	[G] Dihydroxyacetone kinase
	MJLIPHIC_03209	CDS	COG0698	3354375	3354836	+	[G] Ribose 5-phosphate isomerase RpiB
	MJLIPHIC_03210	CDS	COG1363	3354888	3355973	+	[G] Cellulase M and related proteins
	MJLIPHIC_03211	CDS	COG0789	3356052	3356885	+	[K] Predicted transcriptional regulators
1581							
	MJLIPHIC_03212	CDS	NA	3356821	3357093	-	NA
1582							
	MJLIPHIC_03213	CDS	ROG5396	3357375	3358010	+	NA
	MJLIPHIC_03214	CDS	NA	3358007	3358156	+	NA
1583							
	MJLIPHIC_03215	CDS	COG5006	3358295	3359179	+	[R] Predicted permease, DMT superfamily
	MJLIPHIC_03216	CDS	ROG5934	3359207	3359566	+	NA
1584							
	MJLIPHIC_03217	CDS	COG1177	3359563	3360411	-	[E] ABC-type spermidine/putrescine transport system, permease component
	MJLIPHIC_03218	CDS	COG1176	3360408	3361229	-	[E] ABC-type spermidine/putrescine transport system, permease component
	MJLIPHIC_03219	CDS	COG0687	3361287	3362480	-	[E] Spermidine/putrescine-binding periplasmic protein
	MJLIPHIC_03220	CDS	COG3842	3362546	3363667	-	[E] ABC-type spermidine/putrescine transport systems, ATPase components
1585							
	MJLIPHIC_03221	CDS	NA	3364011	3365489	-	NA
1586							
	MJLIPHIC_03222	CDS	ROG1378	3365617	3366084	-	NA
	MJLIPHIC_03223	CDS	COG0473	3366127	3367197	-	[CE] Isocitrate/isopropylmalate dehydrogenase
	MJLIPHIC_03224	CDS	COG0500	3367206	3367937	-	[QR] SAM-dependent methyltransferases
	MJLIPHIC_03225	CDS	COG0596	3367954	3368817	-	[R] Predicted hydrolases or acyltransferases (alpha/beta hydrolase
1587							
	MJLIPHIC_03226	CDS	ROG5049	3368990	3369637	+	NA
1588							
	MJLIPHIC_03227	CDS	COG0596	3369713	3370798	+	[R] Predicted hydrolases or acyltransferases (alpha/beta hydrolase
	MJLIPHIC_03228	CDS	COG2771	3370884	3373658	+	[K] DNA-binding HTH domain-containing proteins
1589							
	MJLIPHIC_03229	CDS	ROG1061	3373987	3374979	+	NA
1590							
	MJLIPHIC_03230	CDS	COG1595	3375460	3376029	+	[K] DNA-directed RNA polymerase specialized sigma subunit,
1591							
	MJLIPHIC_03231	CDS	COG0596	3376204	3377424	+	[R] Predicted hydrolases or acyltransferases (alpha/beta hydrolase
1592							
	MJLIPHIC_03232	CDS	COG3558	3377556	3378146	+	[S] Uncharacterized protein conserved in bacteria
1593							
	MJLIPHIC_03233	CDS	COG0604	3378840	3379142	+	[CR] NADPH:quinone reductase and related Zn-dependent oxidoreductases
	MJLIPHIC_03234	CDS	COG0673	3379139	3380224	+	[R] Predicted dehydrogenases and related proteins
	MJLIPHIC_03235	CDS	COG2141	3380231	3381091	+	[C] Coenzyme F420-dependent N5,N10-methylene tetrahydromethanopterin reductase and
	MJLIPHIC_03236	CDS	COG4221	3381095	3381979	+	[R] Short-chain alcohol dehydrogenase of unknown specificity
	MJLIPHIC_03237	CDS	COG4188	3382040	3383518	+	[R] Predicted dienelactone hydrolase
1594							
	MJLIPHIC_03238	CDS	COG1012	3383623	3384945	+	[C] NAD-dependent aldehyde dehydrogenases
	MJLIPHIC_03239	CDS	COG0697	3384988	3385890	+	[GER] Permeases of the drug/metabolite transporter (DMT)
1595							
	MJLIPHIC_03240	CDS	COG0604	3386129	3387082	+	[CR] NADPH:quinone reductase and related Zn-dependent oxidoreductases
1596							
	MJLIPHIC_03241	CDS	COG0702	3387127	3387678	-	[MG] Predicted nucleoside-diphosphate-sugar epimerases
1597							
	MJLIPHIC_03242	CDS	COG0589	3388088	3388576	+	[T] Universal stress protein UspA and related
	MJLIPHIC_03243	CDS	COG1350	3388626	3389927	+	[R] Predicted alternative tryptophan synthase beta-subunit (paralog
1598							
	MJLIPHIC_03244	CDS	ROG0162	3390024	3390854	+	NA
1599							
	MJLIPHIC_03245	CDS	ROG4257	3390856	3391083	-	NA
1600							
	MJLIPHIC_03246	CDS	ROG3432	3391207	3391764	+	NA
1601							
	MJLIPHIC_03247	CDS	COG1940	3391777	3392985	-	[KG] Transcriptional regulator/sugar kinase
1602							
	MJLIPHIC_03248	CDS	COG2814	3393017	3394192	+	[G] Arabinose efflux permease
1603							
	MJLIPHIC_03249	CDS	NA	3394195	3394533	-	NA
1604							
	MJLIPHIC_03250	CDS	COG3804	3394788	3395795	+	[S] Uncharacterized conserved protein related to dihydrodipicolinate
1605							
	MJLIPHIC_03251	CDS	NA	3395749	3397227	-	NA
	MJLIPHIC_03252	CDS	COG0654	3397224	3398450	-	[HC] 2-polyprenyl-6-methoxyphenol hydroxylase and related FAD-dependent

oxidoreductases

1606							
	MJLIPHIC_03253	CDS	ROG6516	3398604	3399185	+	NA
1607							
	MJLIPHIC_03254	CDS	NA	3399213	3399746	-	NA
	MJLIPHIC_03255	CDS	COG0583	3399695	3400114	-	[K] Transcriptional regulator
1608							
	MJLIPHIC_03256	CDS	ROG0337	3400223	3402250	+	NA
1609							
	MJLIPHIC_03257	CDS	NA	3402317	3403102	+	NA
1610							
	MJLIPHIC_03258	CDS	NA	3403173	3403796	+	NA
	MJLIPHIC_03259	CDS	NA	3403857	3404060	+	NA
1611							
	MJLIPHIC_03260	CDS	NA	3404121	3404666	-	NA
1612							
	MJLIPHIC_03261	CDS	COG2170	3404832	3406028	-	[S] Uncharacterized conserved protein
	MJLIPHIC_03262	CDS	NA	3406015	3406386	-	NA
1613							
	MJLIPHIC_03263	CDS	NA	3406436	3406765	+	NA
1614							
	MJLIPHIC_03264	CDS	ROG1017	3406973	3407374	-	NA
1615							
	MJLIPHIC_03265	CDS	COG0465	3407520	3407699	-	[O] ATP-dependent Zn proteases
	MJLIPHIC_03266	CDS	COG3237	3407751	3407924	-	[S] Uncharacterized protein conserved in bacteria
	MJLIPHIC_03267	CDS	NA	3407950	3408447	-	NA
1616							
	MJLIPHIC_03268	CDS	ROG6516	3408671	3409429	+	NA
1617							
	MJLIPHIC_03269	CDS	COG3239	3409552	3410478	+	[I] Fatty acid desaturase
1618							
	MJLIPHIC_03270	CDS	COG1414	3412864	3413250	+	[K] Transcriptional regulator
1619							
	MJLIPHIC_03271	CDS	COG0596	3413291	3414088	-	[R] Predicted hydrolases or acyltransferases (alpha/beta hydrolase
1620							
	MJLIPHIC_03272	CDS	COG1902	3414688	3415773	+	[C] NADH:flavin oxidoreductases, Old Yellow Enzyme family
1621							
	MJLIPHIC_03273	CDS	NA	3416136	3416618	-	NA
	MJLIPHIC_03274	CDS	COG3712	3416615	3416989	-	[PT] Fe2+-dicitrate sensor, membrane component
	MJLIPHIC_03275	CDS	COG3547	3417007	3418218	-	[L] Transposase and inactivated derivatives
1622							
	MJLIPHIC_03276	CDS	NA	3419267	3420439	+	NA
1623							
	MJLIPHIC_03277	CDS	NA	3420444	3421544	-	NA
1624							
	MJLIPHIC_03278	CDS	COG1802	3421734	3422429	-	[K] Transcriptional regulators
1625							
	MJLIPHIC_03279	CDS	COG3836	3422532	3423314	+	[G] 2,4-dihydroxyhept-2-ene-1,7-dioic acid aldolase
	MJLIPHIC_03280	CDS	COG0388	3423311	3424108	+	[R] Predicted amidohydrolase
	MJLIPHIC_03281	CDS	COG4663	3424110	3425210	+	[Q] TRAP-type mannitol/chloroaromatic compound transport system, periplasmic
	MJLIPHIC_03282	CDS	ROG0528	3425207	3425725	+	NA
	MJLIPHIC_03283	CDS	COG1593	3425722	3427083	+	[G] TRAP-type C4-dicarboxylate transport system, large permease
1626							
	MJLIPHIC_03284	CDS	NA	3427593	3428096	-	NA
1627							
	MJLIPHIC_03285	CDS	ROG0232	3428442	3428870	+	NA
1628							
	MJLIPHIC_03286	CDS	COG1309	3429563	3430054	+	[K] Transcriptional regulator
	MJLIPHIC_03287	CDS	COG0702	3430051	3430938	+	[MG] Predicted nucleoside-diphosphate-sugar epimerases
	MJLIPHIC_03288	CDS	ROG1229	3430925	3431353	+	NA
1629							
	MJLIPHIC_03289	CDS	COG4341	3431438	3432004	+	[R] Predicted HD phosphohydrolase
1630							
	MJLIPHIC_03290	CDS	COG4681	3432244	3432795	+	[S] Uncharacterized protein conserved in bacteria
1631							
	MJLIPHIC_03291	CDS	ROG5845	3433140	3433571	+	NA
1632							
	MJLIPHIC_03292	CDS	COG1309	3433691	3434248	-	[K] Transcriptional regulator
1633							
	MJLIPHIC_03293	CDS	NA	3434447	3435112	-	NA
1634							
	MJLIPHIC_03294	CDS	COG1024	3435357	3436148	-	[I] Enoyl-CoA hydratase/carnithine racemase
1635							
	MJLIPHIC_03295	CDS	COG1309	3436349	3436951	+	[K] Transcriptional regulator
	MJLIPHIC_03296	CDS	COG1280	3436955	3437581	+	[E] Putative threonine efflux protein
	MJLIPHIC_03297	CDS	COG4977	3437645	3438670	+	[K] Transcriptional regulator containing an amidase domain
	MJLIPHIC_03298	CDS	COG4977	3438709	3439404	+	[K] Transcriptional regulator containing an amidase domain
	MJLIPHIC_03299	CDS	COG1028	3439442	3440194	+	[IQR] Dehydrogenases with different specificities (related to
	MJLIPHIC_03300	CDS	COG2211	3440359	3441657	+	[G] Na+/melibiose symporter and related transporters
	MJLIPHIC_03301	CDS	ROG7883	3441702	3441911	+	NA
1636							
	MJLIPHIC_03302	CDS	COG1028	3441941	3442069	-	[IQR] Dehydrogenases with different specificities (related to
	MJLIPHIC_03303	CDS	COG4221	3442145	3442678	-	[R] Short-chain alcohol dehydrogenase of unknown specificity
1637							
	MJLIPHIC_03304	CDS	COG0640	3442809	3443147	-	[K] Predicted transcriptional regulators
1638							
	MJLIPHIC_03305	CDS	COG0477	3443249	3444496	+	[GEPR] Permeases of the major facilitator superfamily
1639							
	MJLIPHIC_03306	CDS	COG3662	3444504	3445607	-	[S] Uncharacterized protein conserved in bacteria
1640							
	MJLIPHIC_03307	CDS	COG2303	3445893	3447587	+	[E] Choline dehydrogenase and related flavoproteins
	MJLIPHIC_03308	CDS	COG0491	3447605	3448519	+	[R] Zn-dependent hydrolases, including glyoxylases
1641							
	MJLIPHIC_03309	CDS	NA	3448679	3449362	-	NA
1642							
	MJLIPHIC_03310	CDS	COG1028	3450104	3450889	+	[IQR] Dehydrogenases with different specificities (related to
1643							
	MJLIPHIC_03311	CDS	COG1680	3450886	3452058	-	[V] Beta-lactamase class C and other penicillin
1644							
	MJLIPHIC_03312	CDS	ROG1744	3452237	3452626	+	NA

1645							
	MJLIPHIC_03313	CDS	COG1182	3452634	3453248	-	[I] Acyl carrier protein phosphodiesterase
1646							
	MJLIPHIC_03314	CDS	COG0640	3453339	3453689	+	[K] Predicted transcriptional regulators
1647							
	MJLIPHIC_03315	CDS	NA	3453838	3453984	-	NA
1648							
	MJLIPHIC_03316	CDS	NA	3454069	3454998	-	NA
	MJLIPHIC_03317	CDS	NA	3454995	3455480	-	NA
	MJLIPHIC_03318	CDS	COG0477	3455477	3456817	-	[GEPR] Permeases of the major facilitator superfamily
	MJLIPHIC_03319	CDS	COG1012	3456825	3458378	-	[C] NAD-dependent aldehyde dehydrogenases
	MJLIPHIC_03320	CDS	COG1062	3458388	3459506	-	[C] Zn-dependent alcohol dehydrogenases, class III
1649							
	MJLIPHIC_03321	CDS	COG3960	3459807	3461426	+	[R] Glyoxylate carboligase
	MJLIPHIC_03322	CDS	COG1024	3461441	3462214	+	[I] Enoyl-CoA hydratase/carnithine racemase
	MJLIPHIC_03323	CDS	COG1804	3462268	3463464	+	[C] Predicted acyl-CoA transferases/carnitine dehydratase
	MJLIPHIC_03324	CDS	COG1960	3463461	3464594	+	[I] Acyl-CoA dehydrogenases
	MJLIPHIC_03325	CDS	COG1960	3464591	3465574	+	[I] Acyl-CoA dehydrogenases
	MJLIPHIC_03326	CDS	ROG2677	3465586	3466005	+	NA
	MJLIPHIC_03327	CDS	COG1028	3466032	3466808	+	[IQR] Dehydrogenases with different specificities (related to
1650							
	MJLIPHIC_03328	CDS	COG1414	3466818	3467576	-	[K] Transcriptional regulator
	MJLIPHIC_03329	CDS	COG0624	3467633	3468964	-	[E] Acetylornithine deacetylase/Succinyl-diaminopimelate desuccinylase and related deacylases
	MJLIPHIC_03330	CDS	COG0477	3469021	3470379	-	[GEPR] Permeases of the major facilitator superfamily
1651							
	MJLIPHIC_03331	CDS	ROG6892	3470418	3471188	+	NA
	MJLIPHIC_03332	CDS	COG2508	3471194	3471556	+	[TQ] Regulator of polyketide synthase expression
1652							
	MJLIPHIC_03333	CDS	COG0154	3471621	3473846	+	[J] Asp-tRNAAsn/Glu-tRNAGln amidotransferase A subunit and related
	MJLIPHIC_03334	CDS	COG1540	3473843	3474640	+	[R] Uncharacterized proteins, homologs of lactam utilization
	MJLIPHIC_03335	CDS	COG1457	3474657	3476018	+	[F] Purine-cytosine permease and related proteins
	MJLIPHIC_03336	CDS	COG0388	3476015	3477016	+	[R] Predicted amidohydrolase
1653							
	MJLIPHIC_03337	CDS	ROG2447	3477140	3478327	+	NA
1654							
	MJLIPHIC_03338	CDS	COG2814	3478441	3479832	-	[G] Arabinose efflux permease
1655							
	MJLIPHIC_03339	CDS	NA	3479977	3480570	+	NA
1656							
	MJLIPHIC_03340	CDS	COG0596	3480577	3481794	-	[R] Predicted hydrolases or acyltransferases (alpha/beta hydrolase
1657							
	MJLIPHIC_03341	CDS	ROG6462	3481863	3482456	+	NA
1658							
	MJLIPHIC_03342	CDS	COG0604	3482577	3483530	-	[CR] NADPH:quinone reductase and related Zn-dependent oxidoreductases
	MJLIPHIC_03343	CDS	COG1309	3483534	3484115	-	[K] Transcriptional regulator
1659							
	MJLIPHIC_03344	CDS	COG2258	3484180	3484680	+	[S] Uncharacterized protein conserved in bacteria
1660							
	MJLIPHIC_03345	CDS	NA	3484684	3485550	-	NA
1661							
	MJLIPHIC_03346	CDS	COG0789	3485576	3485905	+	[K] Predicted transcriptional regulators
1662							
	MJLIPHIC_03347	CDS	COG1289	3486061	3487176	-	[S] Predicted membrane protein
1663							
	MJLIPHIC_03348	CDS	COG1846	3487246	3487755	+	[K] Transcriptional regulators
1664							
	MJLIPHIC_03349	CDS	NA	3487878	3488744	+	NA
1665							
	MJLIPHIC_03350	CDS	ROG4697	3488769	3489257	-	NA
1666							
	MJLIPHIC_03351	CDS	ROG4162	3489356	3489784	-	NA
1667							
	MJLIPHIC_03352	CDS	ROG6556	3489877	3490839	-	NA
1668							
	MJLIPHIC_03353	CDS	COG1028	3491197	3491967	-	[IQR] Dehydrogenases with different specificities (related to
	MJLIPHIC_03354	CDS	COG0477	3491964	3493388	-	[GEPR] Permeases of the major facilitator superfamily
1669							
	MJLIPHIC_03355	CDS	COG1052	3493671	3494702	+	[CHR] Lactate dehydrogenase and related dehydrogenases
	MJLIPHIC_03356	CDS	COG1414	3494759	3495541	+	[K] Transcriptional regulator
	MJLIPHIC_03357	CDS	COG3315	3495569	3496423	+	[Q] O-Methyltransferase involved in polyketide biosynthesis
1670							
	MJLIPHIC_03358	CDS	COG1309	3496425	3497039	-	[K] Transcriptional regulator
	MJLIPHIC_03359	CDS	COG4221	3497064	3497915	-	[R] Short-chain alcohol dehydrogenase of unknown specificity
1671							
	MJLIPHIC_03360	CDS	COG1235	3498088	3499005	+	[R] Metal-dependent hydrolases of the beta-lactamase superfamily
	MJLIPHIC_03361	CDS	ROG0530	3499065	3499430	+	NA
1672							
	MJLIPHIC_03362	CDS	NA	3499991	3501115	+	NA
1673							
	MJLIPHIC_03363	CDS	COG0500	3501227	3501832	+	[QR] SAM-dependent methyltransferases
1674							
	MJLIPHIC_03364	CDS	COG2303	3501834	3502121	-	[E] Choline dehydrogenase and related flavoproteins
1675							
	MJLIPHIC_03365	CDS	COG5421	3502259	3503770	+	[L] Transposase
1676							
	MJLIPHIC_03366	CDS	COG2303	3503726	3505195	-	[E] Choline dehydrogenase and related flavoproteins
1677							
	MJLIPHIC_03367	CDS	COG0545	3505550	3506134	+	[O] FKBP-type peptidyl-prolyl cis-trans isomerases 1
1678							
	MJLIPHIC_03368	CDS	ROG7516	3506198	3506590	+	NA
	MJLIPHIC_03369	CDS	COG4185	3506616	3507194	+	[S] Uncharacterized protein conserved in bacteria
1679							
	MJLIPHIC_03370	CDS	COG0627	3507206	3508558	-	[R] Predicted esterase
1680							
	MJLIPHIC_03371	CDS	NA	3508893	3509048	+	NA
1681							
	MJLIPHIC_03372	CDS	COG1760	3509243	3510631	+	[E] L-serine deaminase
1682							
	MJLIPHIC_03373	CDS	NA	3510665	3511207	-	NA

1683							
	MJLIPHIC_03374	CDS	COG0834	3511367	3512173	+	[ET] ABC-type amino acid transport/signal transduction systems,
1684							
	MJLIPHIC_03375	CDS	NA	3512255	3512413	+	NA
1685							
	MJLIPHIC_03376	CDS	COG2303	3512435	3513934	-	[E] Choline dehydrogenase and related flavoproteins
1686							
	MJLIPHIC_03377	CDS	NA	3514040	3515053	+	NA
1687							
	MJLIPHIC_03378	CDS	COG2197	3515552	3516229	+	[TK] Response regulator containing a CheY-like receiver
1688							
	MJLIPHIC_03379	CDS	COG4585	3516234	3517415	-	[T] Signal transduction histidine kinase
1689							
	MJLIPHIC_03380	CDS	NA	3517620	3518267	-	NA
1690							
	MJLIPHIC_03381	CDS	COG1011	3518377	3519072	+	[R] Predicted hydrolase (HAD superfamily)
1691							
	MJLIPHIC_03382	CDS	COG0300	3519095	3519946	-	[R] Short-chain dehydrogenases of various substrate specificities
1692							
	MJLIPHIC_03383	CDS	COG1309	3520041	3520619	+	[K] Transcriptional regulator
1693							
	MJLIPHIC_03384	CDS	NA	3520788	3521030	+	NA
1694							
	MJLIPHIC_03385	CDS	COG1188	3521174	3521548	+	[J] Ribosome-associated heat shock protein implicated in
1695							
	MJLIPHIC_03386	CDS	NA	3521554	3521997	-	NA
1696							
	MJLIPHIC_03387	CDS	COG1252	3522131	3523510	-	[C] NADH dehydrogenase, FAD-containing subunit
	MJLIPHIC_03388	CDS	ROG4656	3523559	3524395	-	NA
1697							
	MJLIPHIC_03389	CDS	COG2966	3524499	3526022	-	[S] Uncharacterized conserved protein
1698							
	MJLIPHIC_03390	CDS	COG0735	3526370	3526876	+	[P] Fe2+/Zn2+ uptake regulation proteins
	MJLIPHIC_03391	CDS	COG0376	3526916	3529162	+	[P] Catalase (peroxidase I)
1699							
	MJLIPHIC_03392	CDS	COG0778	3529328	3529993	+	[C] Nitroreductase
1700							
	MJLIPHIC_03393	CDS	COG3509	3529999	3530943	-	[Q] Poly(3-hydroxybutyrate) depolymerase
1701							
	MJLIPHIC_03394	CDS	COG1064	3531027	3532019	-	[R] Zn-dependent alcohol dehydrogenases
	MJLIPHIC_03395	CDS	COG0318	3532122	3533981	-	[IQ] Acyl-CoA synthetases (AMP-forming)/AMP-acid ligases II
1702							
	MJLIPHIC_03396	CDS	NA	3534056	3534145	-	NA
1703							
	MJLIPHIC_03397	CDS	COG0318	3534240	3535796	+	[IQ] Acyl-CoA synthetases (AMP-forming)/AMP-acid ligases II
1704							
	MJLIPHIC_03398	CDS	ROG2618	3535783	3536193	-	NA
	MJLIPHIC_03399	CDS	COG1309	3536190	3536777	-	[K] Transcriptional regulator
1705							
	MJLIPHIC_03400	CDS	COG0702	3536879	3537790	+	[MG] Predicted nucleoside-diphosphate-sugar epimerases
1706							
	MJLIPHIC_03401	CDS	NA	3537873	3539153	+	NA
	MJLIPHIC_03402	CDS	ROG1725	3539153	3540352	+	NA
	MJLIPHIC_03403	CDS	COG0035	3540363	3540992	+	[F] Uracil phosphoribosyltransferase
	MJLIPHIC_03404	CDS	COG1266	3540989	3541705	+	[R] Predicted metal-dependent membrane protease
1707							
	MJLIPHIC_03405	CDS	ROG1751	3541709	3542491	-	NA
	MJLIPHIC_03406	CDS	COG0501	3542549	3543670	-	[O] Zn-dependent protease with chaperone function
	MJLIPHIC_03407	CDS	COG0791	3543755	3544417	-	[M] Cell wall-associated hydrolases (invasion-associated proteins)
1708							
	MJLIPHIC_03408	CDS	COG0819	3544701	3545447	+	[K] Putative transcription activator
1709							
	MJLIPHIC_03409	CDS	COG2077	3545574	3546068	-	[O] Peroxiredoxin
1710							
	MJLIPHIC_03410	CDS	COG3570	3546209	3547108	+	[V] Streptomycin 6-kinase
1711							
	MJLIPHIC_03411	CDS	COG4948	3547122	3548141	-	[MR] L-alanine-DL-glutamate epimerase and related enzymes of
1712							
	MJLIPHIC_03412	CDS	NA	3548221	3548475	+	NA
	MJLIPHIC_03413	CDS	COG2258	3548528	3549193	+	[S] Uncharacterized protein conserved in bacteria
1713							
	MJLIPHIC_03414	CDS	COG0662	3549195	3549722	-	[G] Mannose-6-phosphate isomerase
	MJLIPHIC_03415	CDS	COG1595	3549755	3550351	-	[K] DNA-directed RNA polymerase specialized sigma subunit,
	MJLIPHIC_03416	CDS	COG0753	3550348	3551805	-	[P] Catalase
	MJLIPHIC_03417	CDS	ROG0115	3551822	3552478	-	NA
1714							
	MJLIPHIC_03418	CDS	COG4977	3552713	3553684	+	[K] Transcriptional regulator containing an amidase domain
1715							
	MJLIPHIC_03419	CDS	COG2755	3553689	3554375	-	[E] Lysophospholipase L1 and related esterases
1716							
	MJLIPHIC_03420	CDS	COG1835	3554508	3555722	-	[I] Predicted acyltransferases
1717							
	MJLIPHIC_03421	CDS	NA	3556086	3556526	-	NA
1718							
	MJLIPHIC_03422	CDS	COG1524	3556736	3557890	+	[R] Uncharacterized proteins of the AP superfamily
	MJLIPHIC_03423	CDS	NA	3557926	3558267	+	NA
	MJLIPHIC_03424	CDS	NA	3558309	3558770	+	NA
1719							
	MJLIPHIC_03425	CDS	NA	3559123	3559596	+	NA
	MJLIPHIC_03426	CDS	COG2409	3559593	3562502	+	[R] Predicted drug exporters of the RND
1720							
	MJLIPHIC_03427	CDS	COG0640	3562524	3563264	-	[K] Predicted transcriptional regulators
1721							
	MJLIPHIC_03428	CDS	COG0791	3563820	3564401	+	[M] Cell wall-associated hydrolases (invasion-associated proteins)
	MJLIPHIC_03429	CDS	COG3022	3564453	3565199	+	[S] Uncharacterized protein conserved in bacteria
1722							
	MJLIPHIC_03430	CDS	NA	3565323	3565670	-	NA
1723							
	MJLIPHIC_03431	CDS	NA	3566211	3566756	+	NA
1724							

	MJLIPHIC_03432	CDS	COG4907	3566848	3567099	+	[S] Predicted membrane protein
	MJLIPHIC_03433	CDS	COG0154	3567077	3568549	+	[J] Asp-tRNAAsn/Glu-tRNAGln amidotransferase A subunit and related
	MJLIPHIC_03434	CDS	COG0076	3568572	3569969	+	[E] Glutamate decarboxylase and related PLP-dependent proteins
1725							
	MJLIPHIC_03435	CDS	COG3588	3570092	3570985	+	[G] Fructose-1,6-bisphosphate aldolase
	MJLIPHIC_03436	CDS	COG0596	3571022	3571939	+	[R] Predicted hydrolases or acyltransferases (alpha/beta hydrolase
	MJLIPHIC_03437	CDS	COG0491	3572011	3572664	+	[R] Zn-dependent hydrolases, including glyoxylases
1726							
	MJLIPHIC_03438	CDS	ROG0288	3572774	3574159	+	NA
1727							
	MJLIPHIC_03439	CDS	COG3335	3574156	3574863	-	[L] Transposase and inactivated derivatives
1728							
	MJLIPHIC_03440	CDS	COG1546	3575284	3576585	-	[R] Uncharacterized protein (competence- and mitomycin-induced)
	MJLIPHIC_03441	CDS	COG4552	3576626	3577834	-	[R] Predicted acetyltransferase involved in intracellular survival
	MJLIPHIC_03442	CDS	NA	3577888	3578115	-	NA
1729							
	MJLIPHIC_03443	tRNA	NA	3578218	3578313	-	NA
1730							
	MJLIPHIC_03444	CDS	COG3804	3579014	3580246	+	[S] Uncharacterized conserved protein related to dihydrodipicolinate
1731							
	MJLIPHIC_03445	CDS	COG0560	3580099	3580836	-	[E] Phosphoserine phosphatase
1732							
	MJLIPHIC_03446	CDS	ROG1880	3580892	3581272	+	NA
1733							
	MJLIPHIC_03447	CDS	COG2070	3581377	3582507	+	[R] Dioxygenases related to 2-nitropropane dioxygenase
1734							
	MJLIPHIC_03448	CDS	NA	3582635	3583222	+	NA
	MJLIPHIC_03449	CDS	COG0243	3583219	3585468	+	[C] Anaerobic dehydrogenases, typically selenocysteine-containing
1735							
	MJLIPHIC_03450	CDS	NA	3585553	3585981	-	NA
1736							
	MJLIPHIC_03451	CDS	COG2124	3586215	3587507	+	[Q] Cytochrome P450
1737							
	MJLIPHIC_03452	CDS	NA	3587428	3587820	-	NA
1738							
	MJLIPHIC_03453	CDS	COG1396	3587930	3588706	+	[K] Predicted transcriptional regulators
1739							
	MJLIPHIC_03454	CDS	COG2128	3588806	3589417	+	[S] Uncharacterized conserved protein
1740							
	MJLIPHIC_03455	CDS	COG2186	3589470	3590201	+	[K] Transcriptional regulators
	MJLIPHIC_03456	CDS	ROG5361	3590198	3590848	+	NA
1741							
	MJLIPHIC_03457	CDS	COG1376	3590832	3591611	-	[S] Uncharacterized protein conserved in bacteria
1742							
	MJLIPHIC_03458	CDS	ROG0390	3591737	3592342	-	NA
	MJLIPHIC_03459	CDS	NA	3592366	3593796	-	NA
1743							
	MJLIPHIC_03460	CDS	COG3104	3594070	3595527	+	[E] Dipeptide/tripeptide permease
1744							
	MJLIPHIC_03461	CDS	COG1171	3595524	3596474	-	[E] Threonine dehydratase
1745							
	MJLIPHIC_03462	CDS	COG0824	3596576	3597016	+	[R] Predicted thioesterase
	MJLIPHIC_03463	CDS	COG0010	3597069	3598112	+	[E] Arginase/agmatinase/formimionoglutamate hydrolase, arginase family
	MJLIPHIC_03464	CDS	COG0477	3598192	3600066	+	[GEPR] Permeases of the major facilitator superfamily
	MJLIPHIC_03465	CDS	NA	3600074	3600313	+	NA
1746							
	MJLIPHIC_03466	CDS	COG2230	3600326	3601306	-	[M] Cyclopropane fatty acid synthase and related
1747							
	MJLIPHIC_03467	CDS	NA	3601320	3601607	+	NA
	MJLIPHIC_03468	CDS	COG3874	3601604	3601975	+	[S] Uncharacterized conserved protein
1748							
	MJLIPHIC_03469	CDS	COG0785	3601917	3602810	-	[O] Cytochrome c biogenesis protein
	MJLIPHIC_03470	CDS	COG0526	3602815	3603345	-	[OC] Thiol-disulfide isomerase and thioredoxins
	MJLIPHIC_03471	CDS	COG2141	3603465	3604361	-	[C] Coenzyme F420-dependent N5,N10-methylene tetrahydromethanopterin reductase and
	MJLIPHIC_03472	CDS	NA	3604423	3605043	-	NA
1749							
	MJLIPHIC_03473	CDS	ROG5681	3605228	3606676	+	NA
1750							
	MJLIPHIC_03474	CDS	COG0834	3607062	3607949	+	[ET] ABC-type amino acid transport/signal transduction systems,
	MJLIPHIC_03475	CDS	COG0765	3607960	3608673	+	[E] ABC-type amino acid transport system, permease
	MJLIPHIC_03476	CDS	COG0765	3608670	3609350	+	[E] ABC-type amino acid transport system, permease
	MJLIPHIC_03477	CDS	COG1126	3609381	3610136	+	[E] ABC-type polar amino acid transport system,
	MJLIPHIC_03478	CDS	COG2124	3610117	3611469	+	[Q] Cytochrome P450
1751							
	MJLIPHIC_03479	CDS	COG2508	3611488	3612699	-	[TQ] Regulator of polyketide synthase expression
1752							
	MJLIPHIC_03480	CDS	COG0044	3612964	3614376	+	[F] Dihydroorotase and related cyclic amidohydrolases
	MJLIPHIC_03481	CDS	NA	3614417	3615436	+	NA
	MJLIPHIC_03482	CDS	COG0388	3615464	3616312	+	[R] Predicted amidohydrolase
	MJLIPHIC_03483	CDS	COG1953	3616326	3617897	+	[FH] Cytosine/uracil/thiamine/allantoin permeases
1753							
	MJLIPHIC_03484	CDS	COG0531	3617957	3619402	-	[E] Amino acid transporters
1754							
	MJLIPHIC_03485	CDS	COG0412	3619557	3620492	-	[Q] Dienelactone hydrolase and related enzymes
1755							
	MJLIPHIC_03486	CDS	COG1028	3620574	3621500	+	[IQR] Dehydrogenases with different specificities (related to
1756							
	MJLIPHIC_03487	CDS	ROG1185	3621505	3621867	-	NA
	MJLIPHIC_03488	CDS	COG0174	3621908	3623278	-	[E] Glutamine synthetase
	MJLIPHIC_03489	CDS	ROG4605	3623312	3623794	-	NA
1757							
	MJLIPHIC_03490	CDS	COG0477	3623967	3626027	-	[GEPR] Permeases of the major facilitator superfamily
1758							
	MJLIPHIC_03491	CDS	COG2193	3626184	3626669	-	[P] Bacterioferritin (cytochrome b1)
1759							
	MJLIPHIC_03492	CDS	NA	3626921	3627553	+	NA
	MJLIPHIC_03493	CDS	COG2906	3627559	3627768	+	[P] Bacterioferritin-associated ferredoxin
1760							
	MJLIPHIC_03494	CDS	COG1024	3627765	3628532	-	[I] Enoyl-CoA hydratase/carnithine racemase
	MJLIPHIC_03495	CDS	ROG4019	3628592	3629335	-	NA

1761							
	MJLIPHIC_03496	CDS	COG1289	3629372	3631426	+	[S] Predicted membrane protein
1762							
	MJLIPHIC_03497	CDS	ROG3916	3631442	3632071	-	NA
1763							
	MJLIPHIC_03498	CDS	COG1309	3632220	3632795	+	[K] Transcriptional regulator
	MJLIPHIC_03499	CDS	ROG3529	3632831	3633964	+	NA
	MJLIPHIC_03500	CDS	ROG0289	3633903	3635588	+	NA
	MJLIPHIC_03501	CDS	COG0590	3635643	3636122	+	[FJ] Cytosine/adenosine deaminases
	MJLIPHIC_03502	CDS	COG0366	3636125	3637426	+	[G] Glycosidases
1764							
	MJLIPHIC_03503	CDS	COG5579	3637429	3637878	-	[S] Uncharacterized conserved protein
1765							
	MJLIPHIC_03504	CDS	COG2972	3637970	3640156	+	[T] Predicted signal transduction protein with a
	MJLIPHIC_03505	CDS	COG0668	3640153	3641547	+	[M] Small-conductance mechanosensitive channel
1766							
	MJLIPHIC_03506	CDS	COG0627	3641758	3642765	+	[R] Predicted esterase
1767							
	MJLIPHIC_03507	CDS	COG4221	3642828	3643589	-	[R] Short-chain alcohol dehydrogenase of unknown specificity
1768							
	MJLIPHIC_03508	CDS	COG0466	3643729	3646068	+	[O] ATP-dependent Lon protease, bacterial type
1769							
	MJLIPHIC_03509	CDS	COG2141	3646199	3647224	+	[C] Coenzyme F420-dependent N5,N10-methylene tetrahydromethanopterin reductase and
1770							
	MJLIPHIC_03510	CDS	NA	3647152	3647343	-	NA
	MJLIPHIC_03511	CDS	COG2409	3647366	3650227	-	[R] Predicted drug exporters of the RND
	MJLIPHIC_03512	CDS	NA	3650224	3650718	-	NA
1771							
	MJLIPHIC_03513	CDS	COG0477	3650839	3652161	-	[GEPR] Permeases of the major facilitator superfamily
1772							
	MJLIPHIC_03514	CDS	COG1113	3652441	3653880	+	[E] Gamma-aminobutyrate permease and related permeases
1773							
	MJLIPHIC_03515	CDS	NA	3654105	3654329	+	NA
	MJLIPHIC_03516	CDS	COG3662	3654331	3655206	+	[S] Uncharacterized protein conserved in bacteria
	MJLIPHIC_03517	CDS	NA	3655206	3655454	+	NA
	MJLIPHIC_03518	CDS	NA	3655466	3656092	+	NA
	MJLIPHIC_03519	CDS	COG1801	3656099	3656827	+	[S] Uncharacterized conserved protein
	MJLIPHIC_03520	CDS	COG0446	3656839	3658068	+	[R] Uncharacterized NAD(FAD)-dependent dehydrogenases
	MJLIPHIC_03521	CDS	NA	3658095	3658883	+	NA
	MJLIPHIC_03522	CDS	COG0433	3658883	3662089	+	[R] Predicted ATPase
1774							
	MJLIPHIC_03523	CDS	COG1879	3662144	3663193	-	[G] ABC-type sugar transport system, periplasmic component
	MJLIPHIC_03524	CDS	COG1879	3663228	3664277	-	[G] ABC-type sugar transport system, periplasmic component
	MJLIPHIC_03525	CDS	COG1172	3664319	3665353	-	[G] Ribose/xylose/arabinose/galactoside ABC-type transport systems, permease components
	MJLIPHIC_03526	CDS	COG1129	3665350	3666888	-	[G] ABC-type sugar transport system, ATPase component
	MJLIPHIC_03527	CDS	COG1063	3666885	3667931	-	[ER] Threonine dehydrogenase and related Zn-dependent dehydrogenases
	MJLIPHIC_03528	CDS	COG1028	3667925	3668692	-	[IQR] Dehydrogenases with different specificities (related to
	MJLIPHIC_03529	CDS	COG1063	3668719	3669759	-	[ER] Threonine dehydrogenase and related Zn-dependent dehydrogenases
1775							
	MJLIPHIC_03530	CDS	COG2390	3669958	3670938	+	[K] Transcriptional regulator, contains sigma factor-related N-terminal
	MJLIPHIC_03531	CDS	COG1028	3670935	3671717	+	[IQR] Dehydrogenases with different specificities (related to
1776							
	MJLIPHIC_03532	CDS	NA	3671734	3673185	-	NA
1777							
	MJLIPHIC_03533	CDS	COG2141	3673257	3674240	-	[C] Coenzyme F420-dependent N5,N10-methylene tetrahydromethanopterin reductase and
1778							
	MJLIPHIC_03534	CDS	COG3957	3674267	3676639	+	[G] Phosphoketolase
	MJLIPHIC_03535	CDS	COG1266	3676701	3677468	+	[R] Predicted metal-dependent membrane protease
1779							
	MJLIPHIC_03536	CDS	ROG6462	3677470	3678081	-	NA
1780							
	MJLIPHIC_03537	CDS	NA	3678199	3678690	+	NA
1781							
	MJLIPHIC_03538	CDS	COG1064	3678705	3679703	-	[R] Zn-dependent alcohol dehydrogenases
	MJLIPHIC_03539	CDS	COG2261	3679739	3680041	-	[S] Predicted membrane protein
1782							
	MJLIPHIC_03540	CDS	NA	3680523	3680780	+	NA
1783							
	MJLIPHIC_03541	CDS	NA	3680844	3681905	-	NA
1784							
	MJLIPHIC_03542	CDS	COG1028	3682273	3682944	+	[IQR] Dehydrogenases with different specificities (related to
	MJLIPHIC_03543	CDS	COG2141	3682967	3683896	+	[C] Coenzyme F420-dependent N5,N10-methylene tetrahydromethanopterin reductase and
1785							
	MJLIPHIC_03544	CDS	COG1252	3683996	3685369	+	[C] NADH dehydrogenase, FAD-containing subunit
1786							
	MJLIPHIC_03545	CDS	ROG4558	3685389	3686027	-	NA
	MJLIPHIC_03546	CDS	COG0378	3686027	3686713	-	[OK] Ni2+-binding GTPase involved in regulation of
	MJLIPHIC_03547	CDS	COG0830	3686744	3687379	-	[O] Urease accessory protein UreF
	MJLIPHIC_03548	CDS	COG0804	3687380	3689113	-	[E] Urea amidohydrolase (urease) alpha subunit
	MJLIPHIC_03549	CDS	COG0832	3689113	3689442	-	[E] Urea amidohydrolase (urease) beta subunit
	MJLIPHIC_03550	CDS	COG0831	3689452	3689754	-	[E] Urea amidohydrolase (urease) gamma subunit
1787							
	MJLIPHIC_03551	CDS	COG2050	3689837	3690232	-	[Q] Uncharacterized protein, possibly involved in aromatic
1788							
	MJLIPHIC_03552	CDS	ROG4566	3690396	3691169	-	NA
1789							
	MJLIPHIC_03553	CDS	COG3682	3691395	3691811	+	[K] Predicted transcriptional regulator
	MJLIPHIC_03554	CDS	NA	3691826	3692782	+	NA
	MJLIPHIC_03555	CDS	COG0362	3692833	3694299	+	[G] 6-phosphogluconate dehydrogenase
	MJLIPHIC_03556	CDS	COG0516	3694314	3695750	+	[F] IMP dehydrogenase/GMP reductase
1790							
	MJLIPHIC_03557	CDS	COG3842	3695733	3696785	-	[E] ABC-type spermidine/putrescine transport systems, ATPase components
	MJLIPHIC_03558	CDS	COG1178	3696787	3698340	-	[P] ABC-type Fe3+ transport system, permease component
	MJLIPHIC_03559	CDS	COG1840	3698337	3699386	-	[P] ABC-type Fe3+ transport system, periplasmic component
1791							
	MJLIPHIC_03560	CDS	COG1253	3699776	3701146	+	[R] Hemolysins and related proteins containing CBS
	MJLIPHIC_03561	CDS	COG1253	3701139	3702233	+	[R] Hemolysins and related proteins containing CBS
	MJLIPHIC_03562	CDS	ROG0784	3702226	3703254	+	NA
	MJLIPHIC_03563	CDS	COG2225	3703251	3705449	+	[C] Malate synthase

	MJLIPHIC_03564	CDS	ROG3195	3705497	3707785	+	NA
1792	MJLIPHIC_03565	CDS	COG1003	3707851	3710691	-	[E] Glycine cleavage system protein P (pyridoxal-binding),
1793	MJLIPHIC_03566	CDS	ROG3695	3711023	3711637	-	NA
1794	MJLIPHIC_03567	CDS	COG1259	3711891	3712385	-	[S] Uncharacterized conserved protein
	MJLIPHIC_03568	CDS	COG0789	3712479	3713210	-	[K] Predicted transcriptional regulators
	MJLIPHIC_03569	CDS	COG1716	3713279	3713755	-	[T] FOG: FHA domain
1795	MJLIPHIC_03570	CDS	COG0509	3713932	3714327	-	[E] Glycine cleavage system H protein (lipoate-binding)
	MJLIPHIC_03571	CDS	COG3879	3714350	3715183	-	[S] Uncharacterized protein conserved in bacteria
	MJLIPHIC_03572	CDS	COG3856	3715210	3715542	-	[S] Uncharacterized conserved protein (small basic protein)
	MJLIPHIC_03573	CDS	COG3879	3715572	3716495	-	[S] Uncharacterized protein conserved in bacteria
	MJLIPHIC_03574	CDS	COG0558	3716506	3717132	-	[I] Phosphatidylglycerophosphate synthase
	MJLIPHIC_03575	CDS	COG0653	3717156	3719552	-	[U] Preprotein translocase subunit SecA (ATPase, RNA
1796	MJLIPHIC_03576	CDS	COG4178	3719609	3721534	+	[R] ABC-type uncharacterized transport system, permease and
	MJLIPHIC_03577	CDS	COG4178	3721548	3723488	+	[R] ABC-type uncharacterized transport system, permease and
1797	MJLIPHIC_03578	CDS	COG1053	3723502	3725001	-	[C] Succinate dehydrogenase/fumarate reductase, flavoprotein subunit
	MJLIPHIC_03579	CDS	ROG2119	3724998	3725492	-	NA
	MJLIPHIC_03580	CDS	ROG6516	3725537	3726226	-	NA
1798	MJLIPHIC_03581	CDS	ROG3956	3726341	3726784	+	NA
1799	MJLIPHIC_03582	CDS	ROG2168	3726804	3727496	-	NA
1800	MJLIPHIC_03583	CDS	COG1652	3727672	3728298	-	[S] Uncharacterized protein containing LysM domain
1801	MJLIPHIC_03584	CDS	COG0665	3728448	3729887	-	[E] Glycine/D-amino acid oxidases (deaminating)
	MJLIPHIC_03585	CDS	COG0475	3729915	3731060	-	[P] Kef-type K+ transport systems, membrane components
	MJLIPHIC_03586	CDS	COG0490	3731063	3731569	-	[P] Putative regulatory, ligand-binding protein related to
	MJLIPHIC_03587	CDS	COG3000	3731597	3732520	-	[I] Sterol desaturase
	MJLIPHIC_03588	CDS	COG2272	3732542	3734104	-	[I] Carboxylesterase type B
1802	MJLIPHIC_03589	CDS	COG1960	3734280	3735554	+	[I] Acyl-CoA dehydrogenases
	MJLIPHIC_03590	CDS	NA	3735585	3736673	+	NA
	MJLIPHIC_03591	CDS	COG2076	3736700	3737035	+	[P] Membrane transporters of cations and cationic
	MJLIPHIC_03592	CDS	COG2076	3737035	3737358	+	[P] Membrane transporters of cations and cationic
1803	MJLIPHIC_03593	CDS	NA	3737317	3738537	-	NA
1804	MJLIPHIC_03594	CDS	COG1640	3738781	3740985	+	[G] 4-alpha-glucanotransferase
1805	MJLIPHIC_03595	CDS	NA	3741065	3741475	+	NA
1806	MJLIPHIC_03596	CDS	COG3687	3741641	3742540	+	[R] Predicted metal-dependent hydrolase
	MJLIPHIC_03597	CDS	COG1018	3742546	3743655	+	[C] Flavodoxin reductases (ferredoxin-NADPH reductases) family 1
	MJLIPHIC_03598	CDS	COG0515	3743679	3745160	+	[RTKL] Serine/threonine protein kinase
1807	MJLIPHIC_03599	CDS	NA	3745516	3745836	+	NA
1808	MJLIPHIC_03600	CDS	COG4119	3746175	3746669	-	[LR] Predicted NTP pyrophosphohydrolase
1809	MJLIPHIC_03601	CDS	NA	3746956	3747261	+	NA
1810	MJLIPHIC_03602	CDS	ROG0823	3747389	3748171	+	NA
1811	MJLIPHIC_03603	CDS	NA	3748168	3748581	-	NA
1812	MJLIPHIC_03604	CDS	NA	3748660	3749223	-	NA
1813	MJLIPHIC_03605	CDS	ROG8430	3749291	3750160	-	NA
1814	MJLIPHIC_03606	CDS	ROG6017	3750230	3750661	+	NA
1815	MJLIPHIC_03607	CDS	NA	3750763	3751188	+	NA
1816	MJLIPHIC_03608	CDS	COG0318	3751261	3752916	+	[IQ] Acyl-CoA synthetases (AMP-forming)/AMP-acid ligases II
1817	MJLIPHIC_03609	CDS	ROG2318	3752918	3753229	-	NA
	MJLIPHIC_03610	CDS	COG0591	3753243	3754967	-	[ER] Na+/proline symporter
1818	MJLIPHIC_03611	CDS	COG1349	3755127	3755717	+	[KG] Transcriptional regulators of sugar metabolism
	MJLIPHIC_03612	CDS	COG1349	3755662	3755931	+	[KG] Transcriptional regulators of sugar metabolism
	MJLIPHIC_03613	CDS	COG1085	3755928	3757010	+	[C] Galactose-1-phosphate uridylyltransferase
	MJLIPHIC_03614	CDS	COG0153	3757013	3758101	+	[G] Galactokinase
1819	MJLIPHIC_03615	CDS	COG2124	3758177	3759400	-	[Q] Cytochrome P450
1820	MJLIPHIC_03616	CDS	COG0318	3759496	3760995	+	[IQ] Acyl-CoA synthetases (AMP-forming)/AMP-acid ligases II
1821	MJLIPHIC_03617	CDS	ROG8452	3761143	3761553	+	NA
	MJLIPHIC_03618	CDS	ROG0016	3761602	3762354	+	NA
1822	MJLIPHIC_03619	CDS	COG3464	3762362	3763273	-	[L] Transposase and inactivated derivatives
1823	MJLIPHIC_03620	CDS	ROG8452	3763858	3763968	+	NA
	MJLIPHIC_03621	CDS	COG2837	3763965	3765302	+	[P] Predicted iron-dependent peroxidase
	MJLIPHIC_03622	CDS	ROG5429	3765299	3766309	+	NA
1824	MJLIPHIC_03623	CDS	NA	3766313	3767392	-	NA
1825	MJLIPHIC_03624	CDS	COG1028	3767815	3768630	+	[IQR] Dehydrogenases with different specificities (related to
	MJLIPHIC_03625	CDS	COG0477	3768650	3769939	+	[GEPR] Permeases of the major facilitator superfamily
1826	MJLIPHIC_03626	CDS	COG2224	3770113	3772422	+	[C] Isocitrate lyase
	MJLIPHIC_03627	CDS	ROG0744	3772431	3773042	+	NA
	MJLIPHIC_03628	CDS	COG0753	3773103	3774191	+	[P] Catalase

	MJLIPHIC_03629	CDS	COG3038	3774188	3774766	+	[C] Cytochrome B561
1827							
	MJLIPHIC_03630	CDS	ROG2412	3774749	3776077	-	NA
1828							
	MJLIPHIC_03631	CDS	NA	3776153	3776272	-	NA
1829							
	MJLIPHIC_03632	CDS	COG4324	3776635	3777081	+	[R] Predicted aminopeptidase
1830							
	MJLIPHIC_03633	CDS	ROG3702	3777183	3777833	+	NA
1831							
	MJLIPHIC_03634	CDS	COG1020	3778220	3785944	+	[Q] Non-ribosomal peptide synthetase modules and related
1832							
	MJLIPHIC_03635	CDS	COG1028	3786493	3787038	+	[IQR] Dehydrogenases with different specificities (related to
1833							
	MJLIPHIC_03636	CDS	COG3339	3787202	3787639	+	[S] Uncharacterized conserved protein
1834							
	MJLIPHIC_03637	CDS	COG0530	3787674	3788843	-	[P] Ca2+/Na+ antiporter
1835							
	MJLIPHIC_03638	CDS	COG0535	3789058	3790188	-	[R] Predicted Fe-S oxidoreductases
	MJLIPHIC_03639	CDS	ROG0297	3790181	3790477	-	NA
	MJLIPHIC_03640	CDS	COG5424	3790446	3791162	-	[H] Pyrroloquinoline quinone (Coenzyme PQQ) biosynthesis protein
	MJLIPHIC_03641	CDS	COG1235	3791172	3791972	-	[R] Metal-dependent hydrolases of the beta-lactamase superfamily
1836							
	MJLIPHIC_03642	CDS	NA	3792156	3792269	-	NA
	MJLIPHIC_03643	CDS	COG4993	3792319	3794004	-	[G] Glucose dehydrogenase
	MJLIPHIC_03644	CDS	ROG0430	3794018	3794488	-	NA
1837							
	MJLIPHIC_03645	CDS	COG0376	3794904	3797138	-	[P] Catalase (peroxidase I)
	MJLIPHIC_03646	CDS	COG3279	3797154	3798542	-	[KT] Response regulator of the LytR/AlgR family
1838							
	MJLIPHIC_03647	CDS	COG2355	3798739	3799824	-	[E] Zn-dependent dipeptidase, microsomal dipeptidase homolog
1839							
	MJLIPHIC_03648	CDS	ROG0065	3800054	3800311	-	NA
1840							
	MJLIPHIC_03649	tRNA	NA	3800548	3800622	-	NA
1841							
	MJLIPHIC_03650	CDS	COG0730	3800703	3801659	-	[R] Predicted permeases
	MJLIPHIC_03651	CDS	NA	3801719	3801829	-	NA
1842							
	MJLIPHIC_03652	CDS	COG0730	3801921	3802688	-	[R] Predicted permeases
1843							
	MJLIPHIC_03653	CDS	COG1160	3802774	3804189	-	[R] Predicted GTPases
	MJLIPHIC_03654	CDS	COG0283	3804186	3804872	-	[F] Cytidylate kinase
	MJLIPHIC_03655	CDS	COG1187	3804869	3805612	-	[J] 16S rRNA uridine-516 pseudouridylate synthase and
	MJLIPHIC_03656	CDS	COG1386	3805614	3806444	-	[K] Predicted transcriptional regulator containing the HTH
	MJLIPHIC_03657	CDS	COG1354	3806453	3807355	-	[S] Uncharacterized conserved protein
	MJLIPHIC_03658	CDS	COG1192	3807352	3808221	-	[D] ATPases involved in chromosome partitioning
	MJLIPHIC_03659	CDS	COG4974	3808346	3809302	-	[L] Site-specific recombinase XerD
	MJLIPHIC_03660	CDS	COG0494	3809299	3809919	-	[LR] NTP pyrophosphohydrolases including oxidative damage repair
	MJLIPHIC_03661	CDS	COG0504	3809912	3811684	-	[F] CTP synthase (UTP-ammonia lyase)
1844							
	MJLIPHIC_03662	CDS	ROG0882	3811835	3812761	-	NA
	MJLIPHIC_03663	CDS	COG4825	3812765	3813961	-	[S] Uncharacterized membrane-anchored protein conserved in bacteria
	MJLIPHIC_03664	CDS	COG0497	3814080	3815234	-	[L] ATPase involved in DNA repair
	MJLIPHIC_03665	CDS	COG0497	3815261	3815851	-	[L] ATPase involved in DNA repair
	MJLIPHIC_03666	CDS	COG0061	3815858	3816781	-	[G] Predicted sugar kinase
	MJLIPHIC_03667	CDS	COG1189	3816778	3817512	-	[J] Predicted rRNA methylase
1845							
	MJLIPHIC_03668	CDS	NA	3817594	3817800	-	NA
	MJLIPHIC_03669	CDS	COG0647	3817814	3818842	-	[G] Predicted sugar phosphatases of the HAD
	MJLIPHIC_03670	CDS	ROG0426	3818844	3819437	-	NA
1846							
	MJLIPHIC_03671	CDS	COG0162	3825494	3826816	-	[J] Tyrosyl-tRNA synthetase
	MJLIPHIC_03672	CDS	COG2094	3826823	3827446	-	[L] 3-methyladenine DNA glycosylase
1847							
	MJLIPHIC_03673	CDS	ROG4349	3827521	3827730	+	NA
	MJLIPHIC_03674	CDS	COG0542	3827733	3828299	+	[O] ATPases with chaperone activity, ATP-binding subunit
1848							
	MJLIPHIC_03675	CDS	COG4555	3828376	3829140	+	[CP] ABC-type Na+ transport system, ATPase component
	MJLIPHIC_03676	CDS	COG0842	3829137	3829907	+	[V] ABC-type multidrug transport system, permease component
	MJLIPHIC_03677	CDS	COG1309	3829921	3830556	+	[K] Transcriptional regulator
1849							
	MJLIPHIC_03678	CDS	COG2835	3830530	3830739	-	[S] Uncharacterized conserved protein
	MJLIPHIC_03679	CDS	NA	3830749	3831285	-	NA
1850							
	MJLIPHIC_03680	CDS	COG3243	3831361	3834354	-	[I] Poly(3-hydroxyalkanoate) synthetase
1851							
	MJLIPHIC_03681	CDS	COG0488	3834599	3836371	+	[R] ATPase components of ABC transporters with
1852							
	MJLIPHIC_03682	CDS	COG0165	3836484	3837893	-	[E] Argininosuccinate lyase
	MJLIPHIC_03683	CDS	COG0137	3837986	3839185	-	[E] Argininosuccinate synthase
	MJLIPHIC_03684	CDS	COG1438	3839211	3839687	-	[K] Arginine repressor
	MJLIPHIC_03685	CDS	COG0078	3839692	3840615	-	[E] Ornithine carbamoyltransferase
	MJLIPHIC_03686	CDS	COG4992	3840612	3841784	-	[E] Ornithine/acetylornithine aminotransferase
	MJLIPHIC_03687	CDS	COG0548	3841781	3842662	-	[E] Acetylglutamate kinase
	MJLIPHIC_03688	CDS	COG1364	3842659	3843867	-	[E] N-acetylglutamate synthase (N-acetylornithine aminotransferase)
	MJLIPHIC_03689	CDS	COG0002	3843864	3844895	-	[E] Acetylglutamate semialdehyde dehydrogenase
1853							
	MJLIPHIC_03690	CDS	COG0072	3845187	3847685	-	[J] Phenylalanyl-tRNA synthetase beta subunit
	MJLIPHIC_03691	CDS	COG0016	3847685	3848755	-	[J] Phenylalanyl-tRNA synthetase alpha subunit
	MJLIPHIC_03692	CDS	ROG5803	3848763	3849110	-	NA
1854							
	MJLIPHIC_03693	CDS	COG2114	3849549	3850367	-	[T] Adenylate cyclase, family 3 (some proteins
1855							
	MJLIPHIC_03694	CDS	COG3662	3850705	3851742	+	[S] Uncharacterized protein conserved in bacteria
	MJLIPHIC_03695	CDS	COG1960	3851794	3853026	+	[I] Acyl-CoA dehydrogenases
1856							
	MJLIPHIC_03696	CDS	COG0251	3853023	3853430	-	[J] Putative translation initiation inhibitor, yjgF family
	MJLIPHIC_03697	CDS	COG1414	3853450	3854205	-	[K] Transcriptional regulator
	MJLIPHIC_03698	CDS	COG0524	3854210	3855205	-	[G] Sugar kinases, ribokinase family

1857							
	MJLIPHIC_03699	CDS	COG3616	3855341	3856615	+	[E] Predicted amino acid aldolase or racemase
	MJLIPHIC_03700	CDS	COG3653	3856615	3858216	+	[Q] N-acyl-D-aspartate/D-glutamate deacylase
	MJLIPHIC_03701	CDS	COG0800	3858213	3858842	+	[G] 2-keto-3-deoxy-6-phosphogluconate aldolase
	MJLIPHIC_03702	CDS	COG2610	3858872	3860263	+	[GE] H+/gluconate symporter and related permeases
1858							
	MJLIPHIC_03703	CDS	COG0566	3860269	3861027	-	[J] rRNA methylases
	MJLIPHIC_03704	CDS	COG0292	3861051	3861440	-	[J] Ribosomal protein L20
	MJLIPHIC_03705	CDS	COG0291	3861482	3861676	-	[J] Ribosomal protein L35
	MJLIPHIC_03706	CDS	COG0290	3861730	3862230	-	[J] Translation initiation factor 3 (IF-3)
1859							
	MJLIPHIC_03707	CDS	ROG2029	3862580	3862939	+	NA
1860							
	MJLIPHIC_03708	CDS	COG1190	3863007	3866327	+	[J] Lysyl-tRNA synthetase (class II)
	MJLIPHIC_03709	CDS	COG0627	3866375	3867739	+	[R] Predicted esterase
1861							
	MJLIPHIC_03710	CDS	NA	3867751	3868779	-	NA
	MJLIPHIC_03711	CDS	ROG4420	3868776	3870062	-	NA
1862							
	MJLIPHIC_03712	CDS	NA	3870193	3870660	+	NA
1863							
	MJLIPHIC_03713	CDS	NA	3870666	3871868	-	NA
	MJLIPHIC_03714	CDS	NA	3871850	3873571	-	NA
1864							
	MJLIPHIC_03715	CDS	COG3126	3873637	3874113	-	[S] Uncharacterized protein conserved in bacteria
1865							
	MJLIPHIC_03716	CDS	COG1842	3874246	3874500	+	[KT] Phage shock protein A (IM30), suppresses
	MJLIPHIC_03717	CDS	ROG0492	3874504	3875688	+	NA
1866							
	MJLIPHIC_03718	CDS	COG0300	3875678	3876454	-	[R] Short-chain dehydrogenases of various substrate specificities
1867							
	MJLIPHIC_03719	CDS	COG1309	3876488	3877093	+	[K] Transcriptional regulator
	MJLIPHIC_03720	CDS	NA	3877122	3877925	+	NA
1868							
	MJLIPHIC_03721	CDS	COG0178	3877922	3880816	-	[L] Excinuclease ATPase subunit
1869							
	MJLIPHIC_03722	CDS	COG0491	3880937	3881641	+	[R] Zn-dependent hydrolases, including glyoxylases
1870							
	MJLIPHIC_03723	CDS	COG0589	3881645	3882088	-	[T] Universal stress protein UspA and related
1871							
	MJLIPHIC_03724	CDS	COG1946	3882386	3883237	-	[I] Acyl-CoA thioesterase
	MJLIPHIC_03725	CDS	NA	3883219	3883497	-	NA
1872							
	MJLIPHIC_03726	tRNA	NA	3883647	3883718	-	NA
1873							
	MJLIPHIC_03727	CDS	NA	3883930	3885315	-	NA
	MJLIPHIC_03728	CDS	COG0556	3885360	3887519	-	[L] Helicase subunit of the DNA excision
1874							
	MJLIPHIC_03729	CDS	COG2306	3887653	3888093	+	[S] Uncharacterized conserved protein
	MJLIPHIC_03730	CDS	NA	3888105	3888686	+	NA
1875							
	MJLIPHIC_03731	CDS	COG2066	3888683	3889606	-	[E] Glutaminase
1876							
	MJLIPHIC_03732	CDS	COG0531	3889806	3891341	+	[E] Amino acid transporters
1877							
	MJLIPHIC_03733	CDS	COG2186	3891418	3892152	-	[K] Transcriptional regulators
1878							
	MJLIPHIC_03734	CDS	COG1028	3892337	3893056	+	[IQR] Dehydrogenases with different specificities (related to
	MJLIPHIC_03735	CDS	ROG1330	3893053	3894336	+	NA
	MJLIPHIC_03736	CDS	COG1028	3894333	3895127	+	[IQR] Dehydrogenases with different specificities (related to
	MJLIPHIC_03737	CDS	NA	3895124	3896194	+	NA
	MJLIPHIC_03738	CDS	COG0174	3896187	3897479	+	[E] Glutamine synthetase
	MJLIPHIC_03739	CDS	COG0174	3897476	3898855	+	[E] Glutamine synthetase
	MJLIPHIC_03740	CDS	COG0657	3898899	3899843	+	[I] Esterase/lipase
	MJLIPHIC_03741	CDS	COG1028	3899840	3900568	+	[IQR] Dehydrogenases with different specificities (related to
	MJLIPHIC_03742	CDS	COG2072	3900662	3902119	+	[P] Predicted flavoprotein involved in K+ transport
1879							
	MJLIPHIC_03743	CDS	COG0237	3902068	3903270	-	[H] Dephospho-CoA kinase
	MJLIPHIC_03744	CDS	COG0539	3903380	3904819	-	[J] Ribosomal protein S1
1880							
	MJLIPHIC_03745	CDS	COG1309	3905025	3905606	-	[K] Transcriptional regulator
	MJLIPHIC_03746	CDS	COG0702	3905603	3905764	-	[MG] Predicted nucleoside-diphosphate-sugar epimerases
	MJLIPHIC_03747	CDS	COG3631	3905761	3906219	-	[R] Ketosteroid isomerase-related protein
1881							
	MJLIPHIC_03748	CDS	COG2339	3906374	3907534	+	[S] Predicted membrane protein
1882							
	MJLIPHIC_03749	CDS	COG2966	3907516	3908757	-	[S] Uncharacterized conserved protein
	MJLIPHIC_03750	CDS	COG0749	3908769	3911495	-	[L] DNA polymerase I - 3'-5' exonuclease
	MJLIPHIC_03751	CDS	COG0583	3911506	3912411	-	[K] Transcriptional regulator
1883							
	MJLIPHIC_03752	CDS	COG0665	3912482	3913630	+	[E] Glycine/D-amino acid oxidases (deaminating)
	MJLIPHIC_03753	CDS	COG0596	3913627	3914298	+	[R] Predicted hydrolases or acyltransferases (alpha/beta hydrolase
1884							
	MJLIPHIC_03754	CDS	COG1545	3914357	3914806	+	[R] Predicted nucleic-acid-binding protein containing a Zn-ribbon
	MJLIPHIC_03755	CDS	COG0304	3914803	3916005	+	[IQ] 3-oxoacyl-(acyl-carrier-protein) synthase
1885							
	MJLIPHIC_03756	CDS	COG1872	3916139	3916366	+	[S] Uncharacterized conserved protein
1886							
	MJLIPHIC_03757	CDS	ROG0938	3916414	3918393	-	NA
1887							
	MJLIPHIC_03758	CDS	COG3733	3918460	3920481	-	[Q] Cu2+-containing amine oxidase
	MJLIPHIC_03759	CDS	COG2272	3920451	3921959	-	[I] Carboxylesterase type B
1888							
	MJLIPHIC_03760	CDS	NA	3922232	3922738	+	NA
1889							
	MJLIPHIC_03761	CDS	COG2141	3922735	3923841	-	[C] Coenzyme F420-dependent N5,N10-methylene tetrahydromethanopterin reductase and
	MJLIPHIC_03762	CDS	COG0715	3923853	3924875	-	[P] ABC-type nitrate/sulfonate/bicarbonate transport systems, periplasmic components
	MJLIPHIC_03763	CDS	COG1116	3924920	3925657	-	[P] ABC-type nitrate/sulfonate/bicarbonate transport system, ATPase component
	MJLIPHIC_03764	CDS	COG0600	3925654	3926445	-	[P] ABC-type nitrate/sulfonate/bicarbonate transport system, permease component

1890							
	MJLIPHIC_03765	CDS	ROG4412	3926606	3927340	-	NA
1891							
	MJLIPHIC_03766	CDS	COG0596	3927427	3928698	-	[R] Predicted hydrolases or acyltransferases (alpha/beta hydrolase
1892							
	MJLIPHIC_03767	CDS	ROG2015	3928773	3929054	-	NA
1893							
	MJLIPHIC_03768	CDS	ROG0198	3929250	3929594	+	NA
1894							
	MJLIPHIC_03769	CDS	COG0463	3929678	3930475	-	[M] Glycosyltransferases involved in cell wall biogenesis
	MJLIPHIC_03770	CDS	COG0815	3930485	3932269	-	[M] Apolipoprotein N-acyltransferase
	MJLIPHIC_03771	CDS	COG1574	3932325	3933917	-	[R] Predicted metal-dependent hydrolase with the TIM-barrel
	MJLIPHIC_03772	CDS	COG3030	3933944	3934450	-	[R] Protein affecting phage T7 exclusion by
	MJLIPHIC_03773	CDS	ROG0988	3934505	3934897	-	NA
	MJLIPHIC_03774	CDS	COG1429	3934939	3938574	-	[H] Cobalamin biosynthesis protein CobN and related
	MJLIPHIC_03775	CDS	NA	3938632	3938739	-	NA
1895							
	MJLIPHIC_03776	CDS	NA	3938878	3939351	-	NA
	MJLIPHIC_03777	CDS	COG0714	3939351	3941570	-	[R] MoxR-like ATPases
	MJLIPHIC_03778	CDS	ROG7547	3941567	3942052	-	NA
	MJLIPHIC_03779	CDS	ROG6247	3942049	3942978	-	NA
1896							
	MJLIPHIC_03780	CDS	COG2175	3943106	3943993	+	[Q] Probable taurine catabolism dioxygenase
	MJLIPHIC_03781	CDS	COG0155	3944003	3945133	+	[P] Sulfite reductase, beta subunit (hemoprotein)
	MJLIPHIC_03782	CDS	COG2082	3945143	3945769	+	[H] Precorrin isomerase
	MJLIPHIC_03783	CDS	COG1010	3945766	3947277	+	[H] Precorrin-3B methylase
1897							
	MJLIPHIC_03784	CDS	COG1309	3947354	3947998	+	[K] Transcriptional regulator
	MJLIPHIC_03785	CDS	ROG0938	3947995	3949062	+	NA
1898							
	MJLIPHIC_03786	CDS	COG2099	3949043	3949798	-	[H] Precorrin-6x reductase
	MJLIPHIC_03787	CDS	COG2875	3949795	3950595	-	[H] Precorrin-4 methylase
	MJLIPHIC_03788	CDS	COG2242	3950547	3951731	-	[H] Precorrin-6B methylase 2
	MJLIPHIC_03789	CDS	COG0300	3951760	3952485	-	[R] Short-chain dehydrogenases of various substrate specificities
1899							
	MJLIPHIC_03790	CDS	ROG2268	3952554	3952973	+	NA
1900							
	MJLIPHIC_03791	CDS	COG0006	3952974	3954101	-	[E] Xaa-Pro aminopeptidase
	MJLIPHIC_03792	CDS	COG0346	3954112	3954516	-	[E] Lactoylglutathione lyase and related lyases
1901							
	MJLIPHIC_03793	CDS	COG0258	3954552	3955568	+	[L] 5'-3' exonuclease (including N-terminal domain of
1902							
	MJLIPHIC_03794	CDS	COG0839	3955575	3956396	-	[C] NADH:ubiquinone oxidoreductase subunit 6 (chain J)
	MJLIPHIC_03795	CDS	COG4581	3956438	3959182	-	[L] Superfamily II RNA helicase
	MJLIPHIC_03796	CDS	COG0805	3959166	3960119	-	[U] Sec-independent protein secretion pathway component TatC
	MJLIPHIC_03797	CDS	COG1826	3960196	3960441	-	[U] Sec-independent protein secretion pathway components
1903							
	MJLIPHIC_03798	CDS	COG2378	3960513	3961469	-	[K] Predicted transcriptional regulator
	MJLIPHIC_03799	CDS	NA	3961466	3961744	-	NA
	MJLIPHIC_03800	CDS	COG2378	3961710	3962435	-	[K] Predicted transcriptional regulator
	MJLIPHIC_03801	CDS	ROG0364	3962479	3963822	-	NA
1904							
	MJLIPHIC_03802	CDS	COG0596	3963990	3964871	+	[R] Predicted hydrolases or acyltransferases (alpha/beta hydrolase
1905							
	MJLIPHIC_03803	CDS	NA	3964940	3965479	+	NA
	MJLIPHIC_03804	CDS	COG2339	3965476	3966558	+	[S] Predicted membrane protein
1906							
	MJLIPHIC_03805	CDS	COG0638	3966564	3967304	-	[O] 20S proteasome, alpha and beta subunits
	MJLIPHIC_03806	CDS	COG0638	3967301	3968212	-	[O] 20S proteasome, alpha and beta subunits
	MJLIPHIC_03807	CDS	ROG4180	3968209	3968403	-	NA
1907							
	MJLIPHIC_03808	CDS	ROG0268	3968511	3970007	-	NA
	MJLIPHIC_03809	CDS	ROG1999	3970017	3970934	-	NA
	MJLIPHIC_03810	CDS	ROG0034	3970964	3971356	-	NA
	MJLIPHIC_03811	CDS	COG0160	3971368	3972654	-	[E] 4-aminobutyrate aminotransferase and related aminotransferases
	MJLIPHIC_03812	CDS	COG0454	3972734	3973267	-	[KR] Histone acetyltransferase HPA2 and related acetyltransferases
1908							
	MJLIPHIC_03813	CDS	COG0464	3974235	3976076	-	[O] ATPases of the AAA+ class
1909							
	MJLIPHIC_03814	CDS	NA	3976222	3976725	+	NA
	MJLIPHIC_03815	CDS	ROG4412	3976734	3977297	+	NA
	MJLIPHIC_03816	CDS	COG1550	3977303	3977596	+	[S] Uncharacterized protein conserved in bacteria
1910							
	MJLIPHIC_03817	CDS	COG2519	3977602	3978369	-	[J] tRNA(1-methyladenosine) methyltransferase and related methyltransferases
1911							
	MJLIPHIC_03818	CDS	COG2887	3978534	3979409	+	[L] RecB family exonuclease
	MJLIPHIC_03819	CDS	ROG0200	3979406	3980203	+	NA
	MJLIPHIC_03820	CDS	ROG3895	3980200	3980706	+	NA
1912							
	MJLIPHIC_03821	CDS	ROG1229	3980700	3981236	-	NA
1913							
	MJLIPHIC_03822	CDS	COG1028	3981603	3982166	-	[IQR] Dehydrogenases with different specificities (related to
1914							
	MJLIPHIC_03823	CDS	COG1680	3982213	3983361	+	[V] Beta-lactamase class C and other penicillin
1915							
	MJLIPHIC_03824	CDS	NA	3983362	3983514	-	NA
1916							
	MJLIPHIC_03825	CDS	COG1064	3983780	3984850	-	[R] Zn-dependent alcohol dehydrogenases
1917							
	MJLIPHIC_03826	CDS	ROG1139	3985843	3986388	-	NA
1918							
	MJLIPHIC_03827	CDS	ROG5054	3986453	3986968	-	NA
1919							
	MJLIPHIC_03828	CDS	NA	3987416	3988021	+	NA
	MJLIPHIC_03829	CDS	ROG1245	3988011	3988562	+	NA
	MJLIPHIC_03830	CDS	NA	3988559	3989284	+	NA
1920							
	MJLIPHIC_03831	CDS	NA	3989281	3990084	-	NA
1921							
	MJLIPHIC_03832	CDS	ROG5591	3990221	3990724	+	NA

1922							
	MJLIPHIC_03833	CDS	ROG0037	3990727	3991974	-	NA
1923							
	MJLIPHIC_03834	CDS	COG1237	3992368	3993123	+	[R] Metal-dependent hydrolases of the beta-lactamase superfamily
1924							
	MJLIPHIC_03835	CDS	COG0474	3993256	3995970	-	[P] Cation transport ATPase
1925							
	MJLIPHIC_03836	CDS	COG0680	3996059	3996526	-	[C] Ni,Fe-hydrogenase maturation factor
	MJLIPHIC_03837	CDS	COG3259	3996523	3997815	-	[C] Coenzyme F420-reducing hydrogenase, alpha subunit
	MJLIPHIC_03838	CDS	COG1941	3997812	3998570	-	[C] Coenzyme F420-reducing hydrogenase, gamma subunit
	MJLIPHIC_03839	CDS	COG0543	3998571	3999410	-	[HC] 2-polyprenylphenol hydroxylase and related flavodoxin oxidoreductases
	MJLIPHIC_03840	CDS	COG1145	3999400	4000503	-	[C] Ferredoxin
	MJLIPHIC_03841	CDS	COG0071	4000508	4000945	-	[O] Molecular chaperone (small heat shock protein)
1926							
	MJLIPHIC_03842	CDS	COG0574	4001326	4003635	+	[G] Phosphoenolpyruvate synthase/pyruvate phosphate dikinase
1927							
	MJLIPHIC_03843	CDS	ROG0037	4003514	4004869	-	NA
1928							
	MJLIPHIC_03844	CDS	COG1544	4004936	4005712	-	[J] Ribosome-associated protein Y (PSrp-1)
	MJLIPHIC_03845	CDS	COG0589	4005847	4006746	-	[T] Universal stress protein UspA and related
	MJLIPHIC_03846	CDS	ROG6062	4006743	4007213	-	NA
1929							
	MJLIPHIC_03847	CDS	COG0589	4007445	4008236	+	[T] Universal stress protein UspA and related
	MJLIPHIC_03848	CDS	COG0589	4008412	4009293	+	[T] Universal stress protein UspA and related
1930							
	MJLIPHIC_03849	CDS	COG4585	4009310	4010905	-	[T] Signal transduction histidine kinase
1931							
	MJLIPHIC_03850	CDS	COG2187	4011176	4012672	-	[S] Uncharacterized protein conserved in bacteria
1932							
	MJLIPHIC_03851	CDS	ROG4031	4012805	4013794	+	NA
	MJLIPHIC_03852	CDS	COG2197	4013788	4014441	+	[TK] Response regulator containing a CheY-like receiver
1933							
	MJLIPHIC_03853	CDS	COG0589	4014443	4015345	-	[T] Universal stress protein UspA and related
	MJLIPHIC_03854	CDS	ROG7907	4015330	4015920	-	NA
1934							
	MJLIPHIC_03855	CDS	ROG0037	4016005	4017468	+	NA
1935							
	MJLIPHIC_03856	CDS	COG1105	4017452	4018450	-	[G] Fructose-1-phosphate kinase and related fructose-6-phosphate kinase
1936							
	MJLIPHIC_03857	CDS	NA	4018621	4018917	+	NA
1937							
	MJLIPHIC_03858	CDS	COG0589	4019051	4019935	+	[T] Universal stress protein UspA and related
	MJLIPHIC_03859	CDS	ROG4031	4019978	4020961	+	NA
1938							
	MJLIPHIC_03860	CDS	COG0332	4020929	4021870	-	[I] 3-oxoacyl-[acyl-carrier-protein] synthase III
	MJLIPHIC_03861	CDS	COG0332	4021891	4022955	-	[I] 3-oxoacyl-[acyl-carrier-protein] synthase III
1939							
	MJLIPHIC_03862	CDS	COG1554	4023106	4025790	+	[G] Trehalose and maltose hydrolases (possible phosphorylases)
1940							
	MJLIPHIC_03863	CDS	ROG4031	4025793	4026797	-	NA
	MJLIPHIC_03864	CDS	COG2128	4026826	4027074	-	[S] Uncharacterized conserved protein
	MJLIPHIC_03865	CDS	ROG2733	4027120	4028703	-	NA
1941							
	MJLIPHIC_03866	CDS	ROG1880	4028808	4029332	-	NA
1942							
	MJLIPHIC_03867	CDS	COG1802	4029539	4030411	+	[K] Transcriptional regulators
	MJLIPHIC_03868	CDS	ROG5942	4030440	4030958	+	NA
	MJLIPHIC_03869	CDS	COG1309	4030991	4031584	+	[K] Transcriptional regulator
1943							
	MJLIPHIC_03870	CDS	COG1304	4031670	4032854	-	[C] L-lactate dehydrogenase (FMN-dependent) and related alpha-hydroxy
1944							
	MJLIPHIC_03871	CDS	COG0028	4033298	4034893	+	[EH] Thiamine pyrophosphate-requiring enzymes [acetolactate synthase, pyruvate
1945							
	MJLIPHIC_03872	CDS	ROG3131	4035225	4035536	+	NA
1946							
	MJLIPHIC_03873	CDS	COG2378	4035550	4036224	-	[K] Predicted transcriptional regulator
	MJLIPHIC_03874	CDS	COG4922	4036264	4036668	-	[S] Uncharacterized protein conserved in bacteria
	MJLIPHIC_03875	CDS	COG0154	4036718	4038121	-	[J] Asp-tRNAAsn/Glu-tRNAGln amidotransferase A subunit and related
1947							
	MJLIPHIC_03876	CDS	COG0583	4038229	4039044	+	[K] Transcriptional regulator
1948							
	MJLIPHIC_03877	CDS	COG0146	4039051	4041021	-	[EQ] N-methylhydantoinase B/acetone carboxylase, alpha subunit
	MJLIPHIC_03878	CDS	COG0145	4041018	4042334	-	[EQ] N-methylhydantoinase A/acetone carboxylase, beta subunit
1949							
	MJLIPHIC_03879	CDS	COG2508	4043213	4044805	+	[TQ] Regulator of polyketide synthase expression
1950							
	MJLIPHIC_03880	CDS	ROG5811	4044704	4044979	-	NA
1951							
	MJLIPHIC_03881	CDS	COG1309	4045146	4045796	-	[K] Transcriptional regulator
1952							
	MJLIPHIC_03882	CDS	NA	4045998	4046852	+	NA
1953							
	MJLIPHIC_03883	CDS	COG1680	4047077	4048078	+	[V] Beta-lactamase class C and other penicillin
1954							
	MJLIPHIC_03884	CDS	COG0596	4048092	4048514	-	[R] Predicted hydrolases or acyltransferases (alpha/beta hydrolase
	MJLIPHIC_03885	CDS	COG0596	4048511	4048795	-	[R] Predicted hydrolases or acyltransferases (alpha/beta hydrolase
1955							
	MJLIPHIC_03886	CDS	COG1802	4049025	4049669	-	[K] Transcriptional regulators
	MJLIPHIC_03887	CDS	COG1804	4049735	4050916	-	[C] Predicted acyl-CoA transferases/carnitine dehydratase
	MJLIPHIC_03888	CDS	COG1960	4050913	4052046	-	[I] Acyl-CoA dehydrogenases
	MJLIPHIC_03889	CDS	COG1804	4052360	4053208	-	[C] Predicted acyl-CoA transferases/carnitine dehydratase
1956							
	MJLIPHIC_03890	CDS	COG3547	4053599	4054810	+	[L] Transposase and inactivated derivatives
1957							
	MJLIPHIC_03891	CDS	COG0477	4055023	4056354	+	[GEPR] Permeases of the major facilitator superfamily
	MJLIPHIC_03892	CDS	COG0318	4056401	4058032	+	[IQ] Acyl-CoA synthetases (AMP-forming)/AMP-acid ligases II
	MJLIPHIC_03893	CDS	COG0644	4058087	4059460	+	[C] Dehydrogenases (flavoproteins)
1958							
	MJLIPHIC_03894	CDS	COG2345	4059480	4060481	-	[K] Predicted transcriptional regulator
1959							

MJLIPHIC_03895 CDS COG1473 4060590 4061819 + [R] Metal-dependent amidase/aminoacylase/carboxypeptidase
MJLIPHIC_03896 CDS COG0477 4061819 4063096 + [GEPR] Permeases of the major facilitator superfamily
MJLIPHIC_03897 CDS ROG0216 4063093 4063911 + NA
1960
MJLIPHIC_03898 CDS COG0477 4063918 4065294 - [GEPR] Permeases of the major facilitator superfamily
1961
MJLIPHIC_03899 CDS COG3473 4065447 4066202 - [Q] Maleate cis-trans isomerase
MJLIPHIC_03900 CDS COG1028 4066219 4067007 - [IQR] Dehydrogenases with different specificities (related to
1962
MJLIPHIC_03901 CDS COG2195 4067064 4068380 - [E] Di- and tripeptidases
MJLIPHIC_03902 CDS COG0044 4068425 4069843 - [F] Dihydroorotase and related cyclic amidohydrolases
1963
MJLIPHIC_03903 CDS COG2508 4070052 4071197 + [TQ] Regulator of polyketide synthase expression
1964
MJLIPHIC_03904 CDS COG2207 4071433 4072329 - [K] AraC-type DNA-binding domain-containing proteins
1965
MJLIPHIC_03905 CDS COG1879 4072558 4073547 + [G] ABC-type sugar transport system, periplasmic component
MJLIPHIC_03906 CDS COG1129 4073608 4075119 + [G] ABC-type sugar transport system, ATPase component
MJLIPHIC_03907 CDS COG1172 4075142 4076146 + [G] Ribose/xylose/arabinose/galactoside ABC-type transport systems, permease
components
MJLIPHIC_03908 CDS COG1063 4076191 4077273 + [ER] Threonine dehydrogenase and related Zn-dependent dehydrogenases
MJLIPHIC_03909 CDS ROG3340 4077270 4078223 + NA
MJLIPHIC_03910 CDS COG1028 4078223 4078969 + [IQR] Dehydrogenases with different specificities (related to
MJLIPHIC_03911 CDS COG3386 4078966 4079841 + [G] Gluconolactonase
1966
MJLIPHIC_03912 CDS COG2508 4079904 4081523 - [TQ] Regulator of polyketide synthase expression
1967
MJLIPHIC_03913 CDS COG3491 4081539 4082390 + [R] Isopenicillin N synthase and related dioxygenases
MJLIPHIC_03914 CDS COG3491 4082574 4083587 + [R] Isopenicillin N synthase and related dioxygenases
MJLIPHIC_03915 CDS COG1018 4083580 4083852 + [C] Flavodoxin reductases (ferredoxin-NADPH reductases) family 1
MJLIPHIC_03916 CDS COG3324 4083868 4084278 + [R] Predicted enzyme related to lactoylglutathione lyase
MJLIPHIC_03917 CDS COG2233 4084288 4085598 + [F] Xanthine/uracil permeases
MJLIPHIC_03918 CDS COG0044 4085598 4087019 + [F] Dihydroorotase and related cyclic amidohydrolases
MJLIPHIC_03919 CDS COG0325 4087035 4088054 + [R] Predicted enzyme with a TIM-barrel fold
MJLIPHIC_03920 CDS NA 4088051 4089346 + NA
1968
MJLIPHIC_03921 CDS ROG1098 4089826 4090581 - NA
1969
MJLIPHIC_03922 CDS COG0318 4090775 4092508 - [IQ] Acyl-CoA synthetases (AMP-forming)/AMP-acid ligases II
MJLIPHIC_03923 CDS COG1024 4092516 4093307 - [I] Enoyl-CoA hydratase/carnithine racemase
1970
MJLIPHIC_03924 CDS COG1309 4093801 4094346 + [K] Transcriptional regulator
1971
MJLIPHIC_03925 CDS COG1249 4094612 4096099 - [C] Pyruvate/2-oxoglutarate dehydrogenase complex, dihydrolipoamide dehydrogenase
(E3)
1972
MJLIPHIC_03926 CDS COG1309 4096520 4097857 - [K] Transcriptional regulator
MJLIPHIC_03927 CDS COG0583 4097995 4098903 - [K] Transcriptional regulator
1973
MJLIPHIC_03928 CDS NA 4099550 4099693 + NA
MJLIPHIC_03929 CDS COG0604 4099690 4100754 + [CR] NADPH:quinone reductase and related Zn-dependent oxidoreductases
1974
MJLIPHIC_03930 CDS NA 4100797 4101018 - NA
1975
MJLIPHIC_03931 CDS ROG6462 4101244 4101855 - NA
1976
MJLIPHIC_03932 CDS COG3059 4102263 4102760 + [S] Predicted membrane protein
1977
MJLIPHIC_03933 CDS NA 4102959 4103162 + NA
1978
MJLIPHIC_03934 CDS COG0604 4103378 4104352 - [CR] NADPH:quinone reductase and related Zn-dependent oxidoreductases
1979
MJLIPHIC_03935 CDS COG1309 4104482 4105129 + [K] Transcriptional regulator
MJLIPHIC_03936 CDS COG0702 4105207 4106040 + [MG] Predicted nucleoside-diphosphate-sugar epimerases
1980
MJLIPHIC_03937 CDS COG0372 4106113 4106928 - [C] Citrate synthase
MJLIPHIC_03938 CDS COG0318 4106918 4108525 - [IQ] Acyl-CoA synthetases (AMP-forming)/AMP-acid ligases II
MJLIPHIC_03939 CDS ROG5266 4108522 4109028 - NA
1981
MJLIPHIC_03940 CDS COG1012 4109480 4110982 + [C] NAD-dependent aldehyde dehydrogenases
MJLIPHIC_03941 CDS COG1062 4110996 4112135 + [C] Zn-dependent alcohol dehydrogenases, class III
1982
MJLIPHIC_03942 CDS COG0778 4112206 4112853 + [C] Nitroreductase
1983
MJLIPHIC_03943 CDS COG3485 4113063 4113947 + [Q] Protocatechuate 3,4-dioxygenase beta subunit
1984
MJLIPHIC_03944 CDS COG1802 4114106 4114864 + [K] Transcriptional regulators
1985
MJLIPHIC_03945 CDS COG2159 4114950 4115840 + [R] Predicted metal-dependent hydrolase of the TIM-barrel
1986
MJLIPHIC_03946 CDS COG2508 4115906 4117693 - [TQ] Regulator of polyketide synthase expression
MJLIPHIC_03947 CDS COG4829 4117738 4118019 - [Q] Muconolactone delta-isomerase
MJLIPHIC_03948 CDS COG1804 4118016 4119176 - [C] Predicted acyl-CoA transferases/carnitine dehydratase
MJLIPHIC_03949 CDS COG1237 4119176 4120141 - [R] Metal-dependent hydrolases of the beta-lactamase superfamily
1987
MJLIPHIC_03950 CDS ROG0216 4120239 4121222 - NA
MJLIPHIC_03951 CDS COG0477 4121265 4122713 - [GEPR] Permeases of the major facilitator superfamily
MJLIPHIC_03952 CDS NA 4122773 4123459 - NA
1988
MJLIPHIC_03953 CDS COG2771 4124109 4126841 - [K] DNA-binding HTH domain-containing proteins
1989
MJLIPHIC_03954 CDS COG1028 4127253 4128044 + [IQR] Dehydrogenases with different specificities (related to
MJLIPHIC_03955 CDS COG0702 4128118 4128876 + [MG] Predicted nucleoside-diphosphate-sugar epimerases
1990
MJLIPHIC_03956 CDS COG1802 4129257 4129928 + [K] Transcriptional regulators
MJLIPHIC_03957 CDS COG0477 4129996 4131315 + [GEPR] Permeases of the major facilitator superfamily
MJLIPHIC_03958 CDS COG1011 4131375 4132064 + [R] Predicted hydrolase (HAD superfamily)
MJLIPHIC_03959 CDS COG0436 4132099 4133034 + [E] Aspartate/tyrosine/aromatic aminotransferase
1991
MJLIPHIC_03960 CDS COG0251 4133329 4133757 + [J] Putative translation initiation inhibitor, yjgF family

	MJLIPHIC_03961	CDS	COG0154	4133757	4135040	+	[J] Asp-tRNAAsn/Glu-tRNAGln amidotransferase A subunit and related
1992							
	MJLIPHIC_03962	CDS	COG2159	4135328	4135795	-	[R] Predicted metal-dependent hydrolase of the TIM-barrel
1993							
	MJLIPHIC_03963	CDS	COG0604	4136734	4137708	-	[CR] NADPH:quinone reductase and related Zn-dependent oxidoreductases
1994							
	MJLIPHIC_03964	CDS	COG0318	4138269	4139825	-	[IQ] Acyl-CoA synthetases (AMP-forming)/AMP-acid ligases II
	MJLIPHIC_03965	CDS	COG1073	4139932	4141287	-	[R] Hydrolases of the alpha/beta superfamily
1995							
	MJLIPHIC_03966	CDS	COG0604	4141631	4142620	-	[CR] NADPH:quinone reductase and related Zn-dependent oxidoreductases
1996							
	MJLIPHIC_03967	CDS	COG1309	4142735	4143292	+	[K] Transcriptional regulator
1997							
	MJLIPHIC_03968	CDS	COG3335	4144204	4145160	+	[L] Transposase and inactivated derivatives
1998							
	MJLIPHIC_03969	CDS	COG1309	4145792	4146259	-	[K] Transcriptional regulator
1999							
	MJLIPHIC_03970	CDS	COG1028	4146668	4147465	+	[IQR] Dehydrogenases with different specificities (related to
	MJLIPHIC_03971	CDS	COG1042	4147462	4149525	+	[C] Acyl-CoA synthetase (NDP forming)
	MJLIPHIC_03972	CDS	COG1960	4149545	4150744	+	[I] Acyl-CoA dehydrogenases
	MJLIPHIC_03973	CDS	COG1024	4150741	4151499	+	[I] Enoyl-CoA hydratase/carnithine racemase
	MJLIPHIC_03974	CDS	COG2211	4151496	4152893	+	[G] Na+/melibiose symporter and related transporters
2000							
	MJLIPHIC_03975	CDS	COG0596	4152916	4153683	-	[R] Predicted hydrolases or acyltransferases (alpha/beta hydrolase
2001							
	MJLIPHIC_03976	CDS	COG2141	4153904	4154569	-	[C] Coenzyme F420-dependent N5,N10-methylene tetrahydromethanopterin reductase and
2002							
	MJLIPHIC_03977	CDS	COG3547	4155111	4156322	+	[L] Transposase and inactivated derivatives
2003							
	MJLIPHIC_03978	CDS	COG2141	4157064	4158410	-	[C] Coenzyme F420-dependent N5,N10-methylene tetrahydromethanopterin reductase and
	MJLIPHIC_03979	CDS	COG2141	4158407	4159822	-	[C] Coenzyme F420-dependent N5,N10-methylene tetrahydromethanopterin reductase and
	MJLIPHIC_03980	CDS	NA	4159822	4161051	-	NA
	MJLIPHIC_03981	CDS	COG2141	4161044	4162327	-	[C] Coenzyme F420-dependent N5,N10-methylene tetrahydromethanopterin reductase and
	MJLIPHIC_03982	CDS	COG2141	4162324	4162986	-	[C] Coenzyme F420-dependent N5,N10-methylene tetrahydromethanopterin reductase and
	MJLIPHIC_03983	CDS	COG0477	4162983	4164401	-	[GEPR] Permeases of the major facilitator superfamily
2004							
	MJLIPHIC_03984	CDS	COG2963	4164825	4165133	+	[L] Transposase and inactivated derivatives
	MJLIPHIC_03985	CDS	COG2801	4165130	4165990	+	[L] Transposase and inactivated derivatives
2005							
	MJLIPHIC_03986	CDS	COG1414	4166046	4166819	+	[K] Transcriptional regulator
2006							
	MJLIPHIC_03987	CDS	COG2141	4167383	4168717	-	[C] Coenzyme F420-dependent N5,N10-methylene tetrahydromethanopterin reductase and
	MJLIPHIC_03988	CDS	COG2141	4168736	4169806	-	[C] Coenzyme F420-dependent N5,N10-methylene tetrahydromethanopterin reductase and
	MJLIPHIC_03989	CDS	COG0667	4169808	4170839	-	[C] Predicted oxidoreductases (related to aryl-alcohol dehydrogenases)
2007							
	MJLIPHIC_03990	CDS	ROG6169	4170932	4172143	-	NA
	MJLIPHIC_03991	CDS	COG2141	4172140	4173057	-	[C] Coenzyme F420-dependent N5,N10-methylene tetrahydromethanopterin reductase and
	MJLIPHIC_03992	CDS	COG2141	4173054	4174289	-	[C] Coenzyme F420-dependent N5,N10-methylene tetrahydromethanopterin reductase and
	MJLIPHIC_03993	CDS	COG1960	4174286	4175515	-	[I] Acyl-CoA dehydrogenases
	MJLIPHIC_03994	CDS	COG1123	4175512	4177173	-	[R] ATPase components of various ABC-type transport
	MJLIPHIC_03995	CDS	COG1173	4177170	4178078	-	[EP] ABC-type dipeptide/oligopeptide/nickel transport systems, permease components
	MJLIPHIC_03996	CDS	COG0601	4178075	4179127	-	[EP] ABC-type dipeptide/oligopeptide/nickel transport systems, permease components
	MJLIPHIC_03997	CDS	COG0747	4179138	4180772	-	[E] ABC-type dipeptide transport system, periplasmic component
2008							
	MJLIPHIC_03998	CDS	COG1960	4181131	4182240	+	[I] Acyl-CoA dehydrogenases
	MJLIPHIC_03999	CDS	ROG6447	4182282	4183370	+	NA
	MJLIPHIC_04000	CDS	COG2223	4183367	4184623	+	[P] Nitrate/nitrite transporter
2009							
	MJLIPHIC_04001	CDS	COG2801	4184704	4185564	-	[L] Transposase and inactivated derivatives
	MJLIPHIC_04002	CDS	COG2963	4185561	4185869	-	[L] Transposase and inactivated derivatives
2010							
	MJLIPHIC_04003	CDS	COG0406	4185948	4186634	-	[G] Fructose-2,6-bisphosphatase
	MJLIPHIC_04004	CDS	COG1282	4186644	4188083	-	[C] NAD/NADP transhydrogenase beta subunit
	MJLIPHIC_04005	CDS	COG3288	4188102	4189643	-	[C] NAD/NADP transhydrogenase alpha subunit
2011							
	MJLIPHIC_04006	CDS	COG1250	4189781	4190623	-	[I] 3-hydroxyacyl-CoA dehydrogenase
	MJLIPHIC_04007	CDS	COG1804	4190676	4191806	-	[C] Predicted acyl-CoA transferases/carnitine dehydratase
	MJLIPHIC_04008	CDS	COG0318	4191803	4193470	-	[IQ] Acyl-CoA synthetases (AMP-forming)/AMP-acid ligases II
	MJLIPHIC_04009	CDS	COG1960	4193473	4194528	-	[I] Acyl-CoA dehydrogenases
	MJLIPHIC_04010	CDS	COG0447	4194525	4195301	-	[H] Dihydroxynaphthoic acid synthase
	MJLIPHIC_04011	CDS	COG1250	4195303	4196184	-	[I] 3-hydroxyacyl-CoA dehydrogenase
	MJLIPHIC_04012	CDS	COG1250	4196194	4197084	-	[I] 3-hydroxyacyl-CoA dehydrogenase
	MJLIPHIC_04013	CDS	COG1028	4197104	4197868	-	[IQR] Dehydrogenases with different specificities (related to
	MJLIPHIC_04014	CDS	COG1960	4197865	4199088	-	[I] Acyl-CoA dehydrogenases
	MJLIPHIC_04015	CDS	COG1024	4199085	4199873	-	[I] Enoyl-CoA hydratase/carnithine racemase
	MJLIPHIC_04016	CDS	COG1804	4199870	4201099	-	[C] Predicted acyl-CoA transferases/carnitine dehydratase
2012							
	MJLIPHIC_04017	CDS	COG2186	4201359	4202099	-	[K] Transcriptional regulators
2013							
	MJLIPHIC_04018	CDS	COG0477	4202248	4203609	-	[GEPR] Permeases of the major facilitator superfamily
2014							
	MJLIPHIC_04019	CDS	COG2084	4203971	4204870	+	[I] 3-hydroxyisobutyrate dehydrogenase and related beta-hydroxyacid dehydrogenases
	MJLIPHIC_04020	CDS	COG2079	4204867	4206159	+	[R] Uncharacterized protein involved in propionate catabolism
2015							
	MJLIPHIC_04021	CDS	COG1028	4206211	4206993	-	[IQR] Dehydrogenases with different specificities (related to
2016							
	MJLIPHIC_04022	CDS	COG1335	4207206	4207916	+	[Q] Amidases related to nicotinamidase
2017							
	MJLIPHIC_04023	CDS	ROG8500	4208139	4209542	-	NA
	MJLIPHIC_04024	CDS	COG0500	4209578	4210414	-	[QR] SAM-dependent methyltransferases
2018							
	MJLIPHIC_04025	CDS	NA	4210903	4211103	+	NA
2019							
	MJLIPHIC_04026	CDS	NA	4211259	4212110	-	NA
2020							
	MJLIPHIC_04027	CDS	ROG8095	4212365	4212793	+	NA
	MJLIPHIC_04028	CDS	COG2801	4212793	4213806	+	[L] Transposase and inactivated derivatives
2021							
	MJLIPHIC_04029	CDS	ROG8500	4213904	4214770	+	NA
	MJLIPHIC_04030	CDS	ROG8500	4214773	4215222	+	NA

```
2022
        MJLIPHIC_04031  CDS     COG1611 4215569 4216120 -       [R] Predicted Rossmann fold nucleotide-binding protein
        MJLIPHIC_04032  CDS     COG0702 4216158 4216742 -       [MG] Predicted nucleoside-diphosphate-sugar epimerases
        MJLIPHIC_04033  CDS     NA      4216742 4216960 -       NA
2023
        MJLIPHIC_04034  CDS     COG0388 4217342 4218319 +       [R] Predicted amidohydrolase
2024
        MJLIPHIC_04035  CDS     NA      4218757 4219683 +       NA
        MJLIPHIC_04036  CDS     COG1167 4219709 4221142 +       [KE] Transcriptional regulators containing a DNA-binding HTH
2025
        MJLIPHIC_04037  CDS     NA      4221338 4222225 +       NA
2026
        MJLIPHIC_04038  CDS     COG1309 4222396 4223097 -       [K] Transcriptional regulator
2027
        MJLIPHIC_04039  CDS     COG5470 4223158 4223451 +       [S] Uncharacterized conserved protein
        MJLIPHIC_04040  CDS     COG0583 4223495 4224433 +       [K] Transcriptional regulator
2028
        MJLIPHIC_04041  CDS     ROG1651 4224738 4225298 -       NA
2029
        MJLIPHIC_04042  CDS     COG0604 4225664 4226641 +       [CR] NADPH:quinone reductase and related Zn-dependent oxidoreductases
        MJLIPHIC_04043  CDS     ROG6476 4226696 4227970 +       NA
2030
        MJLIPHIC_04044  CDS     COG0657 4228090 4229016 -       [I] Esterase/lipase
2031
        MJLIPHIC_04045  CDS     COG4569 4229192 4230127 +       [Q] Acetaldehyde dehydrogenase (acetylating)
        MJLIPHIC_04046  CDS     NA      4230130 4231164 +       NA
2032
        MJLIPHIC_04047  CDS     ROG5258 4231173 4231424 -       NA
        MJLIPHIC_04048  CDS     COG1018 4231421 4232374 -       [C] Flavodoxin reductases (ferredoxin-NADPH reductases) family 1
        MJLIPHIC_04049  CDS     COG2124 4232377 4232835 -       [Q] Cytochrome P450
2033
        MJLIPHIC_04050  CDS     COG3328 4232893 4234068 +       [L] Transposase and inactivated derivatives
        MJLIPHIC_04051  CDS     COG4974 4234028 4235137 +       [L] Site-specific recombinase XerD
        MJLIPHIC_04052  CDS     ROG8437 4235134 4237359 +       NA
        MJLIPHIC_04053  CDS     ROG0535 4237349 4237777 +       NA
2034
        MJLIPHIC_04054  CDS     NA      4237966 4238103 +       NA
2035
        MJLIPHIC_04055  CDS     COG3328 4238141 4239460 -       [L] Transposase and inactivated derivatives
2036
        MJLIPHIC_04056  CDS     ROG0338 4240426 4241589 -       NA
        MJLIPHIC_04057  CDS     COG1028 4241589 4242311 -       [IQR] Dehydrogenases with different specificities (related to
        MJLIPHIC_04058  CDS     COG1024 4242311 4243027 -       [I] Enoyl-CoA hydratase/carnithine racemase
        MJLIPHIC_04059  CDS     COG1012 4243036 4244478 -       [C] NAD-dependent aldehyde dehydrogenases
        MJLIPHIC_04060  CDS     COG1960 4244495 4245649 -       [I] Acyl-CoA dehydrogenases
        MJLIPHIC_04061  CDS     COG1062 4245646 4246779 -       [C] Zn-dependent alcohol dehydrogenases, class III
        MJLIPHIC_04062  CDS     COG4670 4246781 4248370 -       [I] Acyl CoA:acetate/3-ketoacid CoA transferase
        MJLIPHIC_04063  CDS     COG1028 4248367 4249077 -       [IQR] Dehydrogenases with different specificities (related to
        MJLIPHIC_04064  CDS     COG1129 4249070 4250605 -       [G] ABC-type sugar transport system, ATPase component
        MJLIPHIC_04065  CDS     COG1172 4250602 4251639 -       [G] Ribose/xylose/arabinose/galactoside ABC-type transport systems, permease
components
        MJLIPHIC_04066  CDS     COG1879 4251653 4252771 -       [G] ABC-type sugar transport system, periplasmic component
        MJLIPHIC_04067  CDS     COG2030 4252768 4253202 -       [I] Acyl dehydratase
        MJLIPHIC_04068  CDS     COG1414 4253199 4253996 -       [K] Transcriptional regulator
2037
        MJLIPHIC_04069  CDS     COG0640 4254189 4254506 +       [K] Predicted transcriptional regulators
        MJLIPHIC_04070  CDS     COG3832 4254503 4255015 +       [S] Uncharacterized conserved protein
        MJLIPHIC_04071  CDS     COG0655 4255057 4255647 +       [R] Multimeric flavodoxin WrbA
2038
        MJLIPHIC_04072  CDS     COG1249 4255627 4256982 -       [C] Pyruvate/2-oxoglutarate dehydrogenase complex, dihydrolipoamide dehydrogenase
(E3)
        MJLIPHIC_04073  CDS     COG3918 4256984 4257466 -       [S] Predicted membrane protein
2039
        MJLIPHIC_04074  CDS     COG0040 4257530 4258396 -       [E] ATP phosphoribosyltransferase
        MJLIPHIC_04075  CDS     COG0140 4258399 4258680 -       [E] Phosphoribosyl-ATP pyrophosphohydrolase
        MJLIPHIC_04076  CDS     COG0477 4258730 4260184 -       [GEPR] Permeases of the major facilitator superfamily
        MJLIPHIC_04077  CDS     COG0637 4260273 4261061 -       [R] Predicted phosphatase/phosphohexomutase
2040
        MJLIPHIC_04078  CDS     COG1410 4261269 4265009 -       [E] Methionine synthase I, cobalamin-binding domain
2041
        MJLIPHIC_04079  CDS     ROG0580 4265484 4266362 +       NA
2042
        MJLIPHIC_04080  CDS     COG5361 4266384 4267841 -       [S] Uncharacterized conserved protein
        MJLIPHIC_04081  CDS     COG4221 4267841 4268728 -       [R] Short-chain alcohol dehydrogenase of unknown specificity
        MJLIPHIC_04082  CDS     COG0215 4268754 4270046 -       [J] Cysteinyl-tRNA synthetase
2043
        MJLIPHIC_04083  CDS     COG1218 4270116 4270877 -       [P] 3'-Phosphoadenosine 5'-phosphosulfate (PAPS) 3'-phosphatase
2044
        MJLIPHIC_04084  CDS     ROG0334 4271019 4271867 -       NA
        MJLIPHIC_04085  CDS     ROG0402 4271851 4272438 -       NA
        MJLIPHIC_04086  CDS     COG0406 4272474 4273145 -       [G] Fructose-2,6-bisphosphatase
        MJLIPHIC_04087  CDS     COG1968 4273156 4273986 -       [V] Uncharacterized bacitracin resistance protein
2045
        MJLIPHIC_04088  CDS     ROG6163 4274087 4274392 -       NA
2046
        MJLIPHIC_04089  CDS     ROG2096 4274483 4275574 +       NA
        MJLIPHIC_04090  CDS     ROG1538 4275571 4275840 +       NA
        MJLIPHIC_04091  CDS     COG0167 4275840 4276901 +       [F] Dihydroorotate dehydrogenase
2047
        MJLIPHIC_04092  CDS     COG1881 4276933 4277454 -       [R] Phospholipid-binding protein
        MJLIPHIC_04093  CDS     COG0624 4277491 4278843 -       [E] Acetylornithine deacetylase/Succinyl-diaminopimelate desuccinylase and related
deacylases
2048
        MJLIPHIC_04094  tRNA    NA      4278994 4279080 +       NA
2049
        MJLIPHIC_04095  CDS     ROG5091 4279122 4280453 -       NA
        MJLIPHIC_04096  CDS     COG1404 4280469 4282265 -       [O] Subtilisin-like serine proteases
2050
        MJLIPHIC_04097  CDS     ROG1722 4282640 4283755 +       NA
        MJLIPHIC_04098  CDS     COG2197 4283755 4286454 +       [TK] Response regulator containing a CheY-like receiver
```

2051
MJLIPHIC_04099 CDS COG0369 4286460 4288643 - [P] Sulfite reductase, alpha subunit (flavoprotein)
MJLIPHIC_04100 CDS COG0243 4288640 4290514 - [C] Anaerobic dehydrogenases, typically selenocysteine-containing
2052
MJLIPHIC_04101 CDS COG0589 4290634 4291026 - [T] Universal stress protein UspA and related
MJLIPHIC_04102 CDS COG3333 4291023 4292564 - [S] Uncharacterized protein conserved in bacteria
MJLIPHIC_04103 CDS ROG0803 4292564 4293100 - NA
MJLIPHIC_04104 CDS COG3181 4293097 4294080 - [S] Uncharacterized protein conserved in bacteria
2053
MJLIPHIC_04105 CDS COG3290 4294185 4295864 + [T] Signal transduction histidine kinase regulating citrate/malate
MJLIPHIC_04106 CDS COG4565 4295857 4296519 + [KT] Response regulator of citrate/malate metabolism
2054
MJLIPHIC_04107 CDS COG2124 4296613 4297770 + [Q] Cytochrome P450
MJLIPHIC_04108 CDS COG0518 4297870 4298610 + [F] GMP synthase - Glutamine amidotransferase domain
MJLIPHIC_04109 CDS COG1926 4298656 4299333 + [R] Predicted phosphoribosyltransferases
2055
MJLIPHIC_04110 CDS ROG4896 4299335 4299844 - NA
2056
MJLIPHIC_04111 CDS COG3599 4299939 4300757 - [D] Cell division initiation protein
MJLIPHIC_04112 CDS COG0762 4300998 4301288 - [S] Predicted integral membrane protein
MJLIPHIC_04113 CDS COG1799 4301451 4302095 - [S] Uncharacterized protein conserved in bacteria
MJLIPHIC_04114 CDS COG0325 4302079 4302933 - [R] Predicted enzyme with a TIM-barrel fold
MJLIPHIC_04115 CDS COG1496 4302930 4303643 - [S] Uncharacterized conserved protein
MJLIPHIC_04116 CDS COG0206 4303689 4304846 - [D] Cell division GTPase
MJLIPHIC_04117 CDS COG1589 4305093 4306094 - [M] Cell division septal protein
MJLIPHIC_04118 CDS COG0773 4306091 4307539 - [M] UDP-N-acetylmuramate-alanine ligase
MJLIPHIC_04119 CDS COG0707 4307536 4308693 - [M] UDP-N-acetylglucosamine:LPS N-acetylglucosamine transferase
MJLIPHIC_04120 CDS COG0772 4308690 4310396 - [D] Bacterial cell division membrane protein
MJLIPHIC_04121 CDS COG0771 4310401 4311873 - [M] UDP-N-acetylmuramoylalanine-D-glutamate ligase
MJLIPHIC_04122 CDS COG0472 4311875 4312954 - [M] UDP-N-acetylmuramyl pentapeptide phosphotransferase/UDP-N-acetylglucosamine-1-phosphate transferase
MJLIPHIC_04123 CDS COG0770 4312951 4314480 - [M] UDP-N-acetylmuramyl pentapeptide synthase
MJLIPHIC_04124 CDS COG0769 4314477 4316039 - [M] UDP-N-acetylmuramyl tripeptide synthase
MJLIPHIC_04125 CDS COG0768 4316086 4317975 - [M] Cell division protein FtsI/penicillin-binding protein 2
MJLIPHIC_04126 CDS ROG6184 4318029 4319171 - NA
MJLIPHIC_04127 CDS COG0275 4319171 4320151 - [M] Predicted S-adenosylmethionine-dependent methyltransferase involved in cell
MJLIPHIC_04128 CDS COG2001 4320309 4320740 - [S] Uncharacterized protein conserved in bacteria
2057
MJLIPHIC_04129 CDS ROG0375 4321263 4321664 - NA
2058
MJLIPHIC_04130 CDS COG0454 4321930 4322538 + [KR] Histone acetyltransferase HPA2 and related acetyltransferases
MJLIPHIC_04131 CDS ROG5393 4322584 4323288 + NA
2059
MJLIPHIC_04132 CDS COG0142 4323431 4324519 + [H] Geranylgeranyl pyrophosphate synthase
MJLIPHIC_04133 CDS ROG1842 4324544 4326061 + NA
2060
MJLIPHIC_04134 CDS ROG0418 4326128 4326532 - NA
2061
MJLIPHIC_04135 CDS COG0515 4326569 4327846 + [RTKL] Serine/threonine protein kinase
2062
MJLIPHIC_04136 CDS COG3200 4327868 4329262 - [E] 3-deoxy-D-arabino-heptulosonate 7-phosphate (DAHP) synthase
MJLIPHIC_04137 CDS ROG1312 4329267 4329776 - NA
MJLIPHIC_04138 CDS ROG2468 4329722 4330684 - NA
2063
MJLIPHIC_04139 CDS ROG0801 4330768 4332081 + NA
2064
MJLIPHIC_04140 CDS COG0204 4332098 4332823 - [I] 1-acyl-sn-glycerol-3-phosphate acyltransferase
2065
MJLIPHIC_04141 CDS ROG4640 4332930 4333337 - NA
MJLIPHIC_04142 CDS COG0003 4333334 4334611 - [P] Oxyanion-translocating ATPase
MJLIPHIC_04143 CDS ROG1353 4334612 4335049 - NA
2066
MJLIPHIC_04144 CDS ROG5112 4335143 4335532 - NA
2067
MJLIPHIC_04145 CDS COG1022 4335624 4337423 + [I] Long-chain acyl-CoA synthetases (AMP-forming)
2068
MJLIPHIC_04146 CDS COG0438 4337414 4338571 - [M] Glycosyltransferase
MJLIPHIC_04147 CDS ROG2443 4338603 4339400 - NA
2069
MJLIPHIC_04148 CDS COG0791 4339480 4340523 - [M] Cell wall-associated hydrolases (invasion-associated proteins)
2070
MJLIPHIC_04149 CDS ROG4577 4340779 4341150 - NA
2071
MJLIPHIC_04150 CDS COG0847 4341273 4343180 + [L] DNA polymerase III, epsilon subunit and
2072
MJLIPHIC_04151 CDS COG0547 4343055 4344149 - [E] Anthranilate phosphoribosyltransferase
2073
MJLIPHIC_04152 CDS COG1845 4344330 4344872 + [C] Heme/copper-type cytochrome/quinol oxidase, subunit 3
MJLIPHIC_04153 CDS COG2010 4344911 4345717 + [C] Cytochrome c, mono- and diheme variants
MJLIPHIC_04154 CDS COG0723 4345714 4346922 + [C] Rieske Fe-S protein
MJLIPHIC_04155 CDS COG1290 4346919 4348559 + [C] Cytochrome b subunit of the bc
2074
MJLIPHIC_04156 CDS ROG1037 4349138 4349701 - NA
MJLIPHIC_04157 CDS ROG3872 4349701 4350594 - NA
2075
MJLIPHIC_04158 CDS ROG0175 4350770 4351189 - NA
MJLIPHIC_04159 CDS COG1622 4351209 4352234 - [C] Heme/copper-type cytochrome/quinol oxidases, subunit 2
2076
MJLIPHIC_04160 CDS COG0367 4352415 4354370 + [E] Asparagine synthase (glutamine-hydrolyzing)
2077
MJLIPHIC_04161 CDS COG0524 4354392 4355369 - [G] Sugar kinases, ribokinase family
2078
MJLIPHIC_04162 CDS ROG1389 4355600 4356217 + NA
2079
MJLIPHIC_04163 CDS COG0316 4356255 4356611 - [S] Uncharacterized conserved protein
2080
MJLIPHIC_04164 CDS ROG0152 4356754 4357524 + NA
MJLIPHIC_04165 CDS COG2087 4357552 4358091 + [H] Adenosyl cobinamide kinase/adenosyl cobinamide phosphate guanylyltransferase
MJLIPHIC_04166 CDS COG2038 4358088 4359143 + [H] NaMN:DMB phosphoribosyltransferase
MJLIPHIC_04167 CDS COG0368 4359163 4359888 + [H] Cobalamin-5-phosphate synthase

2081							
	MJLIPHIC_04168	CDS	COG0115	4359885	4360991	-	[EH] Branched-chain amino acid aminotransferase/4-amino-4-deoxychorismate lyase
	MJLIPHIC_04169	CDS	COG0404	4361041	4362138	-	[E] Glycine cleavage system T protein (aminomethyltransferase)
2082							
	MJLIPHIC_04170	CDS	COG2114	4362195	4363286	+	[T] Adenylate cyclase, family 3 (some proteins
	MJLIPHIC_04171	CDS	COG0260	4363297	4364850	+	[E] Leucyl aminopeptidase
2083							
	MJLIPHIC_04172	CDS	COG1028	4364847	4366583	-	[IQR] Dehydrogenases with different specificities (related to
2084							
	MJLIPHIC_04173	CDS	ROG1405	4366658	4366969	-	NA
2085							
	MJLIPHIC_04174	CDS	COG0508	4367210	4368967	+	[C] Pyruvate/2-oxoglutarate dehydrogenase complex, dihydrolipoamide acyltransferase (E2)
	MJLIPHIC_04175	CDS	COG1090	4369020	4369928	+	[R] Predicted nucleoside-diphosphate sugar epimerase
	MJLIPHIC_04176	CDS	COG0321	4369946	4370632	+	[H] Lipoate-protein ligase B
	MJLIPHIC_04177	CDS	COG0320	4370637	4371581	+	[H] Lipoate synthase
	MJLIPHIC_04178	CDS	ROG0149	4371586	4372338	+	NA
	MJLIPHIC_04179	CDS	COG1574	4372372	4374180	+	[R] Predicted metal-dependent hydrolase with the TIM-barrel
2086							
	MJLIPHIC_04180	CDS	ROG0296	4374158	4374655	-	NA
2087							
	MJLIPHIC_04181	CDS	COG0174	4374853	4376289	+	[E] Glutamine synthetase
2088							
	MJLIPHIC_04182	CDS	COG1490	4376518	4376949	+	[J] D-Tyr-tRNAtyr deacylase
	MJLIPHIC_04183	CDS	COG2259	4376977	4377387	+	[S] Predicted membrane protein
2089							
	MJLIPHIC_04184	CDS	COG1391	4377492	4380491	-	[OT] Glutamine synthetase adenylyltransferase
	MJLIPHIC_04185	CDS	COG0174	4380541	4381881	-	[E] Glutamine synthetase
	MJLIPHIC_04186	CDS	COG0596	4381934	4383451	-	[R] Predicted hydrolases or acyltransferases (alpha/beta hydrolase
	MJLIPHIC_04187	CDS	COG0596	4383565	4385097	-	[R] Predicted hydrolases or acyltransferases (alpha/beta hydrolase
	MJLIPHIC_04188	CDS	ROG4443	4385129	4386526	-	NA
2090							
	MJLIPHIC_04189	CDS	COG0413	4386689	4387552	+	[H] Ketopantoate hydroxymethyltransferase
2091							
	MJLIPHIC_04190	CDS	COG1024	4387638	4388423	-	[I] Enoyl-CoA hydratase/carnithine racemase
	MJLIPHIC_04191	CDS	COG1309	4388435	4389112	-	[K] Transcriptional regulator
	MJLIPHIC_04192	CDS	COG0318	4389120	4390946	-	[IQ] Acyl-CoA synthetases (AMP-forming)/AMP-acid ligases II
2092							
	MJLIPHIC_04193	CDS	COG5607	4391109	4392635	+	[S] Uncharacterized conserved protein
2093							
	MJLIPHIC_04194	CDS	COG0500	4393133	4394689	-	[QR] SAM-dependent methyltransferases
2094							
	MJLIPHIC_04195	CDS	COG1396	4394719	4395333	+	[K] Predicted transcriptional regulators
2095							
	MJLIPHIC_04196	CDS	COG0406	4395335	4396432	-	[G] Fructose-2,6-bisphosphatase
	MJLIPHIC_04197	CDS	COG1579	4396429	4397157	-	[R] Zn-ribbon protein, possibly nucleic acid-binding
	MJLIPHIC_04198	CDS	COG0327	4397147	4398298	-	[S] Uncharacterized conserved protein
2096							
	MJLIPHIC_04199	CDS	COG0546	4398573	4399265	+	[R] Predicted phosphatases
	MJLIPHIC_04200	CDS	COG0394	4399216	4399749	+	[T] Protein-tyrosine-phosphatase
	MJLIPHIC_04201	CDS	COG3346	4399749	4400582	+	[S] Uncharacterized conserved protein
2097							
	MJLIPHIC_04202	CDS	COG1270	4400536	4401492	-	[H] Cobalamin biosynthesis protein CobD/CbiB
2098							
	MJLIPHIC_04203	CDS	COG0346	4401829	4402245	+	[E] Lactoylglutathione lyase and related lyases
2099							
	MJLIPHIC_04204	CDS	NA	4402310	4402825	+	NA
2100							
	MJLIPHIC_04205	CDS	COG1595	4402920	4403411	+	[K] DNA-directed RNA polymerase specialized sigma subunit,
	MJLIPHIC_04206	CDS	COG0350	4403408	4403971	+	[L] Methylated DNA-protein cysteine methyltransferase
	MJLIPHIC_04207	CDS	COG3826	4403968	4404681	+	[S] Uncharacterized protein conserved in bacteria
2101							
	MJLIPHIC_04208	CDS	COG2116	4404837	4405655	+	[P] Formate/nitrite family of transporters
2102							
	MJLIPHIC_04209	tRNA	NA	4405774	4405846	-	NA
	MJLIPHIC_04210	CDS	COG0450	4405907	4406377	-	[O] Peroxiredoxin
	MJLIPHIC_04211	CDS	ROG0272	4406378	4406815	-	NA
2103							
	MJLIPHIC_04212	CDS	ROG5373	4406957	4407526	-	NA
2104							
	MJLIPHIC_04213	CDS	COG2609	4407679	4410468	+	[C] Pyruvate dehydrogenase complex, dehydrogenase (E1) component
	MJLIPHIC_04214	CDS	COG2508	4410564	4411850	+	[TQ] Regulator of polyketide synthase expression
	MJLIPHIC_04215	CDS	COG0331	4412023	4412940	+	[I] (acyl-carrier-protein) S-malonyltransferase
	MJLIPHIC_04216	CDS	COG0236	4413021	4413320	+	[IQ] Acyl carrier protein
	MJLIPHIC_04217	CDS	COG0304	4413317	4414567	+	[IQ] 3-oxoacyl-(acyl-carrier-protein) synthase
	MJLIPHIC_04218	CDS	COG0304	4414597	4415850	+	[IQ] 3-oxoacyl-(acyl-carrier-protein) synthase
	MJLIPHIC_04219	CDS	COG4799	4415919	4417343	+	[I] Acetyl-CoA carboxylase, carboxyltransferase component (subunits alpha
2105							
	MJLIPHIC_04220	CDS	COG1028	4417418	4418167	-	[IQR] Dehydrogenases with different specificities (related to
2106							
	MJLIPHIC_04221	CDS	COG0583	4418265	4419254	+	[K] Transcriptional regulator
2107							
	MJLIPHIC_04222	CDS	COG4392	4419138	4419461	-	[S] Predicted membrane protein
	MJLIPHIC_04223	CDS	COG0578	4419488	4421044	-	[C] Glycerol-3-phosphate dehydrogenase
	MJLIPHIC_04224	CDS	COG1309	4421041	4421625	-	[K] Transcriptional regulator
2108							
	MJLIPHIC_04225	CDS	COG0277	4421643	4423253	+	[C] FAD/FMN-containing dehydrogenases
	MJLIPHIC_04226	CDS	COG1597	4423250	4424152	+	[IR] Sphingosine kinase and enzymes related to
2109							
	MJLIPHIC_04227	CDS	ROG0331	4424220	4424732	-	NA
2110							
	MJLIPHIC_04228	CDS	COG1680	4424856	4425689	-	[V] Beta-lactamase class C and other penicillin
	MJLIPHIC_04229	CDS	COG0500	4425730	4426842	-	[QR] SAM-dependent methyltransferases
	MJLIPHIC_04230	CDS	COG2197	4426949	4428589	-	[TK] Response regulator containing a CheY-like receiver
2111							
	MJLIPHIC_04231	CDS	COG1062	4428679	4429770	+	[C] Zn-dependent alcohol dehydrogenases, class III
	MJLIPHIC_04232	CDS	COG0491	4429796	4430428	+	[R] Zn-dependent hydrolases, including glyoxylases
2112							
	MJLIPHIC_04233	CDS	ROG1170	4430800	4432173	+	NA
2113							

	MJLIPHIC_04234	CDS	ROG3728	4432170	4432934	-	NA
2114							
	MJLIPHIC_04235	CDS	ROG5406	4433006	4433236	+	NA
	MJLIPHIC_04236	CDS	ROG6228	4433239	4434321	+	NA
2115							
	MJLIPHIC_04237	CDS	COG0028	4434329	4435885	-	[EH] Thiamine pyrophosphate-requiring enzymes [acetolactate synthase, pyruvate
2116							
	MJLIPHIC_04238	CDS	COG1924	4435960	4437738	-	[I] Activator of 2-hydroxyglutaryl-CoA dehydratase (HSP70-class ATPase
2117							
	MJLIPHIC_04239	CDS	COG3804	4437803	4438945	-	[S] Uncharacterized conserved protein related to dihydrodipicolinate
2118							
	MJLIPHIC_04240	CDS	COG1028	4438978	4439736	+	[IQR] Dehydrogenases with different specificities (related to
2119							
	MJLIPHIC_04241	CDS	ROG5359	4439737	4440798	-	NA
	MJLIPHIC_04242	CDS	NA	4440809	4441339	-	NA
2120							
	MJLIPHIC_04243	CDS	COG0747	4441453	4442991	+	[E] ABC-type dipeptide transport system, periplasmic component
	MJLIPHIC_04244	CDS	COG0601	4442988	4443965	+	[EP] ABC-type dipeptide/oligopeptide/nickel transport systems, permease components
	MJLIPHIC_04245	CDS	COG1173	4443962	4444840	+	[EP] ABC-type dipeptide/oligopeptide/nickel transport systems, permease components
	MJLIPHIC_04246	CDS	COG1123	4444837	4446417	+	[R] ATPase components of various ABC-type transport
	MJLIPHIC_04247	CDS	COG1028	4446420	4447160	+	[IQR] Dehydrogenases with different specificities (related to
2121							
	MJLIPHIC_04248	CDS	ROG0954	4447270	4447512	+	NA
2122							
	MJLIPHIC_04249	CDS	ROG2337	4447509	4448855	-	NA
2123							
	MJLIPHIC_04250	CDS	COG0589	4449005	4449823	-	[T] Universal stress protein UspA and related
2124							
	MJLIPHIC_04251	CDS	COG3631	4450008	4450583	-	[R] Ketosteroid isomerase-related protein
2125							
	MJLIPHIC_04252	CDS	ROG2337	4450843	4453014	+	NA
2126							
	MJLIPHIC_04253	CDS	COG0515	4453016	4454593	-	[RTKL] Serine/threonine protein kinase
2127							
	MJLIPHIC_04254	CDS	COG2421	4454720	4455976	-	[C] Predicted acetamidase/formamidase
	MJLIPHIC_04255	CDS	ROG0648	4455998	4456285	-	NA
2128							
	MJLIPHIC_04256	CDS	COG2508	4456431	4457708	-	[TQ] Regulator of polyketide synthase expression
2129							
	MJLIPHIC_04257	CDS	COG1018	4457769	4458716	-	[C] Flavodoxin reductases (ferredoxin-NADPH reductases) family 1
	MJLIPHIC_04258	CDS	COG4638	4458713	4459807	-	[PR] Phenylpropionate dioxygenase and related ring-hydroxylating dioxygenases,
	MJLIPHIC_04259	CDS	COG0726	4459820	4460713	-	[G] Predicted xylanase/chitin deacetylase
	MJLIPHIC_04260	CDS	COG0010	4460713	4461672	-	[E] Arginase/agmatinase/formimionoglutamate hydrolase, arginase family
	MJLIPHIC_04261	CDS	COG1028	4461684	4462337	-	[IQR] Dehydrogenases with different specificities (related to
	MJLIPHIC_04262	CDS	COG1028	4462339	4463046	-	[IQR] Dehydrogenases with different specificities (related to
	MJLIPHIC_04263	CDS	COG0531	4463043	4464521	-	[E] Amino acid transporters
2130							
	MJLIPHIC_04264	CDS	COG2197	4464835	4465473	+	[TK] Response regulator containing a CheY-like receiver
2131							
	MJLIPHIC_04265	CDS	COG1335	4465578	4466222	+	[Q] Amidases related to nicotinamidase
2132							
	MJLIPHIC_04266	CDS	COG4178	4466375	4468300	+	[R] ABC-type uncharacterized transport system, permease and
2133							
	MJLIPHIC_04267	CDS	COG2421	4468358	4469371	+	[C] Predicted acetamidase/formamidase
	MJLIPHIC_04268	CDS	COG1028	4469384	4470145	+	[IQR] Dehydrogenases with different specificities (related to
2134							
	MJLIPHIC_04269	CDS	COG2409	4470276	4473152	+	[R] Predicted drug exporters of the RND
2135							
	MJLIPHIC_04270	CDS	COG0744	4473252	4475720	+	[M] Membrane carboxypeptidase (penicillin-binding protein)
2136							
	MJLIPHIC_04271	CDS	COG0747	4476002	4477612	+	[E] ABC-type dipeptide transport system, periplasmic component
	MJLIPHIC_04272	CDS	COG0601	4477609	4478577	+	[EP] ABC-type dipeptide/oligopeptide/nickel transport systems, permease components
	MJLIPHIC_04273	CDS	COG1173	4478574	4479428	+	[EP] ABC-type dipeptide/oligopeptide/nickel transport systems, permease components
	MJLIPHIC_04274	CDS	COG1123	4479425	4481053	+	[R] ATPase components of various ABC-type transport
	MJLIPHIC_04275	CDS	COG2141	4481050	4482420	+	[C] Coenzyme F420-dependent N5,N10-methylene tetrahydromethanopterin reductase and
	MJLIPHIC_04276	CDS	COG1960	4482417	4483634	+	[I] Acyl-CoA dehydrogenases
2137							
	MJLIPHIC_04277	CDS	COG1228	4483839	4485146	+	[Q] Imidazolonepropionase and related amidohydrolases
	MJLIPHIC_04278	CDS	COG2513	4485158	4486039	+	[G] PEP phosphonomutase and related enzymes
2138							
	MJLIPHIC_04279	CDS	COG0583	4486041	4486934	-	[K] Transcriptional regulator
2139							
	MJLIPHIC_04280	CDS	NA	4487077	4487268	+	NA
2140							
	MJLIPHIC_04281	CDS	COG1335	4487275	4488030	-	[Q] Amidases related to nicotinamidase
	MJLIPHIC_04282	CDS	COG0789	4488131	4488526	-	[K] Predicted transcriptional regulators
2141							
	MJLIPHIC_04283	CDS	NA	4488622	4489575	+	NA
	MJLIPHIC_04284	CDS	ROG8172	4489599	4489976	+	NA
2142							
	MJLIPHIC_04285	CDS	COG1063	4489984	4491066	-	[ER] Threonine dehydrogenase and related Zn-dependent dehydrogenases
	MJLIPHIC_04286	CDS	COG0546	4491063	4491764	-	[R] Predicted phosphatases
2143							
	MJLIPHIC_04287	CDS	ROG0016	4492416	4493168	+	NA
2144							
	MJLIPHIC_04288	CDS	COG3464	4493176	4494087	-	[L] Transposase and inactivated derivatives
2145							
	MJLIPHIC_04289	CDS	NA	4494638	4494880	+	NA
2146							
	MJLIPHIC_04290	CDS	COG1595	4495047	4495625	-	[K] DNA-directed RNA polymerase specialized sigma subunit,
2147							
	MJLIPHIC_04291	CDS	ROG6919	4495727	4496029	-	NA
	MJLIPHIC_04292	CDS	NA	4496063	4496482	-	NA
	MJLIPHIC_04293	CDS	COG3059	4496500	4497018	-	[S] Predicted membrane protein
2148							
	MJLIPHIC_04294	CDS	NA	4497433	4497702	-	NA
	MJLIPHIC_04295	CDS	COG1018	4497720	4498460	-	[C] Flavodoxin reductases (ferredoxin-NADPH reductases) family 1
	MJLIPHIC_04296	CDS	COG2041	4498464	4499063	-	[R] Sulfite oxidase and related enzymes
	MJLIPHIC_04297	CDS	ROG4412	4499073	4499372	-	NA
	MJLIPHIC_04298	CDS	COG2323	4499356	4499895	-	[S] Predicted membrane protein

	MJLIPHIC_04299	CDS	COG0176	4499892	4501004	-	[G] Transaldolase
	MJLIPHIC_04300	CDS	COG0225	4501152	4501721	-	[O] Peptide methionine sulfoxide reductase
	MJLIPHIC_04301	CDS	COG0229	4501718	4502212	-	[O] Conserved domain frequently associated with peptide
	MJLIPHIC_04302	CDS	COG1028	4502235	4503011	-	[IQR] Dehydrogenases with different specificities (related to
2149							
	MJLIPHIC_04303	CDS	COG1917	4503248	4503682	+	[S] Uncharacterized conserved protein, contains double-stranded beta-helix
	MJLIPHIC_04304	CDS	ROG0107	4503695	4504246	+	NA
	MJLIPHIC_04305	CDS	COG1249	4504243	4505727	+	[C] Pyruvate/2-oxoglutarate dehydrogenase complex, dihydrolipoamide dehydrogenase (E3)
	MJLIPHIC_04306	CDS	COG0604	4505724	4506692	+	[CR] NADPH:quinone reductase and related Zn-dependent oxidoreductases
	MJLIPHIC_04307	CDS	COG0251	4506742	4507137	+	[J] Putative translation initiation inhibitor, yjgF family
	MJLIPHIC_04308	CDS	COG0778	4507134	4507535	+	[C] Nitroreductase
	MJLIPHIC_04309	CDS	NA	4507529	4508977	+	NA
2150							
	MJLIPHIC_04310	CDS	COG0702	4508963	4509754	-	[MG] Predicted nucleoside-diphosphate-sugar epimerases
	MJLIPHIC_04311	CDS	ROG6534	4509784	4510221	-	NA
2151							
	MJLIPHIC_04312	CDS	NA	4510350	4510757	-	NA
2152							
	MJLIPHIC_04313	CDS	COG2909	4510896	4513175	-	[K] ATP-dependent transcriptional regulator
2153							
	MJLIPHIC_04314	CDS	COG3899	4513754	4516432	-	[R] Predicted ATPase
2154							
	MJLIPHIC_04315	CDS	COG1252	4516806	4518224	+	[C] NADH dehydrogenase, FAD-containing subunit
	MJLIPHIC_04316	CDS	COG0561	4518221	4519033	+	[R] Predicted hydrolases of the HAD superfamily
2155							
	MJLIPHIC_04317	CDS	COG0684	4519067	4519702	-	[H] Demethylmenaquinone methyltransferase
	MJLIPHIC_04318	CDS	COG3959	4519702	4521636	-	[G] Transketolase, N-terminal subunit
	MJLIPHIC_04319	CDS	COG0843	4521629	4523380	-	[C] Heme/copper-type cytochrome/quinol oxidases, subunit 1
2156							
	MJLIPHIC_04320	CDS	COG1670	4523543	4524148	-	[J] Acetyltransferases, including N-acetylases of ribosomal proteins
	MJLIPHIC_04321	CDS	COG1028	4524154	4524924	-	[IQR] Dehydrogenases with different specificities (related to
	MJLIPHIC_04322	CDS	COG1917	4524954	4525862	-	[S] Uncharacterized conserved protein, contains double-stranded beta-helix
	MJLIPHIC_04323	CDS	COG0604	4525892	4526812	-	[CR] NADPH:quinone reductase and related Zn-dependent oxidoreductases
	MJLIPHIC_04324	CDS	NA	4526809	4527093	-	NA
2157							
	MJLIPHIC_04325	CDS	COG1249	4527355	4528722	+	[C] Pyruvate/2-oxoglutarate dehydrogenase complex, dihydrolipoamide dehydrogenase (E3)
2158							
	MJLIPHIC_04326	CDS	ROG1120	4528949	4529092	+	NA
	MJLIPHIC_04327	CDS	COG2337	4529095	4529424	+	[T] Growth inhibitor
2159							
	MJLIPHIC_04328	CDS	COG1309	4529467	4530030	-	[K] Transcriptional regulator
2160							
	MJLIPHIC_04329	CDS	COG1073	4530136	4531071	+	[R] Hydrolases of the alpha/beta superfamily
	MJLIPHIC_04330	CDS	COG2072	4531068	4532780	+	[P] Predicted flavoprotein involved in K+ transport
2161							
	MJLIPHIC_04331	tRNA	NA	4532820	4532894	-	NA
2162							
	MJLIPHIC_04332	CDS	ROG0270	4532997	4533377	+	NA
	MJLIPHIC_04333	CDS	COG2008	4533395	4534420	+	[E] Threonine aldolase
2163							
	MJLIPHIC_04334	CDS	COG1680	4534594	4535847	+	[V] Beta-lactamase class C and other penicillin
2164							
	MJLIPHIC_04335	CDS	ROG4605	4535851	4536666	-	NA
	MJLIPHIC_04336	CDS	COG1522	4536707	4537606	-	[K] Transcriptional regulators
2165							
	MJLIPHIC_04337	CDS	COG1457	4537835	4539388	+	[F] Purine-cytosine permease and related proteins
	MJLIPHIC_04338	CDS	COG0010	4539385	4540344	+	[E] Arginase/agmatinase/formimionoglutamate hydrolase, arginase family
	MJLIPHIC_04339	CDS	COG0028	4540344	4541966	+	[EH] Thiamine pyrophosphate-requiring enzymes [acetolactate synthase, pyruvate
	MJLIPHIC_04340	CDS	COG0591	4541971	4543458	+	[ER] Na+/proline symporter
2166							
	MJLIPHIC_04341	CDS	COG5522	4543424	4544110	-	[S] Predicted integral membrane protein
	MJLIPHIC_04342	CDS	NA	4544142	4545503	-	NA
	MJLIPHIC_04343	CDS	COG0312	4545500	4546999	-	[R] Predicted Zn-dependent proteases and their inactivated
2167							
	MJLIPHIC_04344	CDS	ROG5614	4547090	4547860	-	NA
2168							
	MJLIPHIC_04345	CDS	COG1175	4548039	4548956	+	[G] ABC-type sugar transport systems, permease components
	MJLIPHIC_04346	CDS	COG0395	4548943	4549770	+	[G] ABC-type sugar transport system, permease component
	MJLIPHIC_04347	CDS	COG1653	4549859	4551151	+	[G] ABC-type sugar transport system, periplasmic component
2169							
	MJLIPHIC_04348	CDS	COG0619	4551156	4552001	-	[P] ABC-type cobalt transport system, permease component
	MJLIPHIC_04349	CDS	COG1122	4551998	4553965	-	[P] ABC-type cobalt transport system, ATPase component
2170							
	MJLIPHIC_04350	CDS	COG1846	4554138	4554617	+	[K] Transcriptional regulators
	MJLIPHIC_04351	CDS	COG2814	4554636	4555844	+	[G] Arabinose efflux permease
2171							
	MJLIPHIC_04352	CDS	COG1814	4555859	4556590	-	[S] Uncharacterized membrane protein
2172							
	MJLIPHIC_04353	CDS	COG1960	4556645	4558594	+	[I] Acyl-CoA dehydrogenases
2173							
	MJLIPHIC_04354	CDS	NA	4558601	4559095	-	NA
2174							
	MJLIPHIC_04355	CDS	COG2822	4559240	4559560	+	[P] Predicted periplasmic lipoprotein involved in iron
2175							
	MJLIPHIC_04356	CDS	COG0596	4559577	4561130	-	[R] Predicted hydrolases or acyltransferases (alpha/beta hydrolase
2176							
	MJLIPHIC_04357	tRNA	NA	4561209	4561282	-	NA
2177							
	MJLIPHIC_04358	CDS	ROG4050	4561426	4561695	+	NA
2178							
	MJLIPHIC_04359	CDS	NA	4561717	4561908	-	NA
2179							
	MJLIPHIC_04360	CDS	COG0358	4562121	4564031	-	[L] DNA primase (bacterial type)
	MJLIPHIC_04361	CDS	COG0232	4564085	4565371	-	[F] dGTP triphosphohydrolase
2180							
	MJLIPHIC_04362	CDS	ROG1389	4565431	4567473	+	NA
2181							
	MJLIPHIC_04363	CDS	COG0423	4567679	4569064	-	[J] Glycyl-tRNA synthetase (class II)

2182							
	MJLIPHIC_04364	CDS	COG0640	4569182	4569544	+	[K] Predicted transcriptional regulators
	MJLIPHIC_04365	CDS	COG0735	4569541	4569951	+	[P] Fe2+/Zn2+ uptake regulation proteins
2183							
	MJLIPHIC_04366	CDS	COG0494	4569965	4570369	-	[LR] NTP pyrophosphohydrolases including oxidative damage repair
	MJLIPHIC_04367	CDS	ROG4483	4570366	4570821	-	NA
	MJLIPHIC_04368	CDS	COG0020	4570835	4571716	-	[I] Undecaprenyl pyrophosphate synthase
	MJLIPHIC_04369	CDS	COG1381	4571664	4572506	-	[L] Recombinational DNA repair protein (RecF pathway)
2184							
	MJLIPHIC_04370	CDS	COG0154	4572570	4574156	+	[J] Asp-tRNAAsn/Glu-tRNAGln amidotransferase A subunit and related
2185							
	MJLIPHIC_04371	CDS	COG1159	4574188	4575087	-	[R] GTPase
	MJLIPHIC_04372	CDS	ROG1573	4575100	4575417	-	NA
	MJLIPHIC_04373	CDS	COG1253	4575410	4576780	-	[R] Hemolysins and related proteins containing CBS
	MJLIPHIC_04374	CDS	COG0319	4576777	4577313	-	[R] Predicted metal-dependent hydrolase
	MJLIPHIC_04375	CDS	COG1702	4577310	4578356	-	[T] Phosphate starvation-inducible protein PhoH, predicted ATPase
2186							
	MJLIPHIC_04376	CDS	ROG6378	4578517	4578684	-	NA
2187							
	MJLIPHIC_04377	CDS	COG1301	4578798	4580201	-	[C] Na+/H+-dicarboxylate symporters
2188							
	MJLIPHIC_04378	CDS	COG1385	4580289	4581029	-	[S] Uncharacterized protein conserved in bacteria
	MJLIPHIC_04379	CDS	COG0484	4581049	4582194	-	[O] DnaJ-class molecular chaperone with C-terminal Zn
	MJLIPHIC_04380	CDS	COG1420	4582259	4583290	-	[K] Transcriptional regulator of heat shock gene
2189							
	MJLIPHIC_04381	CDS	COG5450	4583484	4583798	+	[K] Transcription regulator of the Arc/MetJ class
2190							
	MJLIPHIC_04382	CDS	COG3251	4583805	4584014	-	[S] Uncharacterized protein conserved in bacteria
	MJLIPHIC_04383	CDS	ROG6973	4583992	4585323	-	NA
	MJLIPHIC_04384	CDS	COG1020	4585320	4589759	-	[Q] Non-ribosomal peptide synthetase modules and related
	MJLIPHIC_04385	CDS	COG1020	4589756	4595215	-	[Q] Non-ribosomal peptide synthetase modules and related
	MJLIPHIC_04386	CDS	COG3321	4595208	4598198	-	[Q] Polyketide synthase modules and related proteins
	MJLIPHIC_04387	CDS	COG3321	4598198	4599499	-	[Q] Polyketide synthase modules and related proteins
	MJLIPHIC_04388	CDS	COG3208	4599486	4600232	-	[Q] Predicted thioesterase involved in non-ribosomal peptide
	MJLIPHIC_04389	CDS	COG1020	4600229	4603738	-	[Q] Non-ribosomal peptide synthetase modules and related
2191							
	MJLIPHIC_04390	CDS	COG1021	4603836	4605512	+	[Q] Peptide arylation enzymes
2192							
	MJLIPHIC_04391	CDS	COG1309	4605520	4606146	-	[K] Transcriptional regulator
2193							
	MJLIPHIC_04392	CDS	ROG2476	4606212	4606742	+	NA
	MJLIPHIC_04393	CDS	NA	4606739	4607761	+	NA
	MJLIPHIC_04394	CDS	COG1116	4607737	4608453	+	[P] ABC-type nitrate/sulfonate/bicarbonate transport system, ATPase component
	MJLIPHIC_04395	CDS	COG0715	4608450	4609616	+	[P] ABC-type nitrate/sulfonate/bicarbonate transport systems, periplasmic components
	MJLIPHIC_04396	CDS	COG0600	4609613	4610410	+	[P] ABC-type nitrate/sulfonate/bicarbonate transport system, permease component
2194							
	MJLIPHIC_04397	CDS	COG3335	4610407	4611114	-	[L] Transposase and inactivated derivatives
2195							
	MJLIPHIC_04398	CDS	COG0147	4611535	4612887	-	[EH] Anthranilate/para-aminobenzoate synthases component I
	MJLIPHIC_04399	CDS	COG0635	4612935	4614107	-	[H] Coproporphyrinogen III oxidase and related Fe-S
2196							
	MJLIPHIC_04400	CDS	COG0155	4614429	4616138	+	[P] Sulfite reductase, beta subunit (hemoprotein)
	MJLIPHIC_04401	CDS	COG0175	4616144	4616854	+	[EH] 3'-phosphoadenosine 5'-phosphosulfate sulfotransferase (PAPS reductase)/FAD synthetase
	MJLIPHIC_04402	CDS	COG2138	4616851	4617564	+	[S] Uncharacterized conserved protein
2197							
	MJLIPHIC_04403	CDS	COG1118	4617583	4618668	-	[P] ABC-type sulfate/molybdate transport systems, ATPase component
	MJLIPHIC_04404	CDS	COG4208	4618684	4619508	-	[P] ABC-type sulfate transport system, permease component
	MJLIPHIC_04405	CDS	COG0555	4619505	4620362	-	[O] ABC-type sulfate transport system, permease component
	MJLIPHIC_04406	CDS	COG1613	4620359	4621408	-	[P] ABC-type sulfate transport system, periplasmic component
2198							
	MJLIPHIC_04407	CDS	ROG6457	4621748	4621951	-	NA
2199							
	MJLIPHIC_04408	CDS	COG3387	4622057	4624063	+	[G] Glucoamylase and related glycosyl hydrolases
2200							
	MJLIPHIC_04409	CDS	NA	4624067	4625263	-	NA
2201							
	MJLIPHIC_04410	CDS	ROG0961	4625673	4626596	+	NA
	MJLIPHIC_04411	CDS	COG0520	4626583	4628262	+	[E] Selenocysteine lyase
	MJLIPHIC_04412	CDS	COG2141	4628321	4629451	+	[C] Coenzyme F420-dependent N5,N10-methylene tetrahydromethanopterin reductase and
	MJLIPHIC_04413	CDS	COG4552	4629448	4630668	+	[R] Predicted acetyltransferase involved in intracellular survival
	MJLIPHIC_04414	CDS	COG1123	4630773	4632392	+	[R] ATPase components of various ABC-type transport
	MJLIPHIC_04415	CDS	COG1173	4632389	4633327	+	[EP] ABC-type dipeptide/oligopeptide/nickel transport systems, permease components
	MJLIPHIC_04416	CDS	COG0601	4633332	4634315	+	[EP] ABC-type dipeptide/oligopeptide/nickel transport systems, permease components
	MJLIPHIC_04417	CDS	COG0747	4634344	4636113	+	[E] ABC-type dipeptide transport system, periplasmic component
2202							
	MJLIPHIC_04418	CDS	COG0500	4636110	4636661	-	[QR] SAM-dependent methyltransferases
2203							
	MJLIPHIC_04419	CDS	COG0604	4637301	4637630	+	[CR] NADPH:quinone reductase and related Zn-dependent oxidoreductases
2204							
	MJLIPHIC_04420	CDS	COG1960	4637637	4638872	-	[I] Acyl-CoA dehydrogenases
	MJLIPHIC_04421	CDS	COG0454	4638869	4639852	-	[KR] Histone acetyltransferase HPA2 and related acetyltransferases
	MJLIPHIC_04422	CDS	COG0531	4639849	4641354	-	[E] Amino acid transporters
2205							
	MJLIPHIC_04423	CDS	COG3547	4641611	4642816	+	[L] Transposase and inactivated derivatives
2206							
	MJLIPHIC_04424	CDS	COG2141	4642947	4644329	-	[C] Coenzyme F420-dependent N5,N10-methylene tetrahydromethanopterin reductase and
	MJLIPHIC_04425	CDS	COG2141	4644326	4645660	-	[C] Coenzyme F420-dependent N5,N10-methylene tetrahydromethanopterin reductase and
2207							
	MJLIPHIC_04426	CDS	NA	4645732	4646238	+	NA
2208							
	MJLIPHIC_04427	CDS	NA	4646256	4646960	-	NA
	MJLIPHIC_04428	CDS	NA	4646989	4647786	-	NA
2209							
	MJLIPHIC_04429	CDS	COG0481	4647861	4649756	-	[M] Membrane GTPase LepA
2210							
	MJLIPHIC_04430	CDS	ROG0953	4649836	4650471	+	NA
2211							
	MJLIPHIC_04431	CDS	COG1120	4650468	4651241	-	[PH] ABC-type cobalamin/Fe3+-siderophores transport systems, ATPase components

MJLIPHIC_04432 CDS COG0609 4651238 4652218 - [P] ABC-type Fe3+-siderophore transport system, permease component
MJLIPHIC_04433 CDS COG0614 4652260 4653279 - [P] ABC-type Fe3+-hydroxamate transport system, periplasmic component
MJLIPHIC_04434 CDS COG0614 4653276 4654298 - [P] ABC-type Fe3+-hydroxamate transport system, periplasmic component
2212
MJLIPHIC_04435 CDS COG2153 4654776 4655399 - [R] Predicted acyltransferase
2213
MJLIPHIC_04436 CDS COG0517 4655467 4655898 - [R] FOG: CBS domain
2214
MJLIPHIC_04437 CDS COG0667 4656041 4657063 + [C] Predicted oxidoreductases (related to aryl-alcohol dehydrogenases)
2215
MJLIPHIC_04438 CDS ROG2733 4657101 4657679 - NA
2216
MJLIPHIC_04439 CDS NA 4657804 4658643 - NA
2217
MJLIPHIC_04440 CDS COG1234 4658741 4659601 + [R] Metal-dependent hydrolases of the beta-lactamase superfamily
2218
MJLIPHIC_04441 CDS COG1305 4659585 4660424 - [E] Transglutaminase-like enzymes, putative cysteine proteases
MJLIPHIC_04442 CDS COG2307 4660425 4661402 - [S] Uncharacterized protein conserved in bacteria
MJLIPHIC_04443 CDS COG2308 4661412 4663040 - [S] Uncharacterized conserved protein
2219
MJLIPHIC_04444 CDS COG0268 4663326 4663586 + [J] Ribosomal protein S20
2220
MJLIPHIC_04445 CDS COG1466 4663655 4664614 - [L] DNA polymerase III, delta subunit
MJLIPHIC_04446 CDS COG0658 4664622 4666136 - [R] Predicted membrane metal-binding protein
MJLIPHIC_04447 CDS COG1555 4666133 4666975 - [L] DNA uptake protein and related DNA-binding
2221
MJLIPHIC_04448 CDS NA 4667078 4668343 - NA
2222
MJLIPHIC_04449 CDS COG0330 4668449 4669255 - [O] Membrane protease subunits, stomatin/prohibitin homologs
2223
MJLIPHIC_04450 CDS COG1307 4669359 4670201 - [S] Uncharacterized protein conserved in bacteria
MJLIPHIC_04451 CDS ROG6620 4670203 4670961 - NA
MJLIPHIC_04452 CDS COG0406 4670951 4671631 - [G] Fructose-2,6-bisphosphatase
MJLIPHIC_04453 CDS COG0799 4671642 4672043 - [S] Uncharacterized homolog of plant Iojap protein
MJLIPHIC_04454 CDS COG1057 4672040 4672681 - [H] Nicotinic acid mononucleotide adenylyltransferase
2224
MJLIPHIC_04455 CDS COG3552 4672767 4674224 - [R] Protein containing von Willebrand factor type
MJLIPHIC_04456 CDS COG0464 4674237 4675133 - [O] ATPases of the AAA+ class
MJLIPHIC_04457 CDS COG0014 4675173 4676447 - [E] Gamma-glutamyl phosphate reductase
2225
MJLIPHIC_04458 CDS COG0524 4676563 4677423 + [G] Sugar kinases, ribokinase family
2226
MJLIPHIC_04459 CDS COG0715 4677428 4678480 - [P] ABC-type nitrate/sulfonate/bicarbonate transport systems, periplasmic components
MJLIPHIC_04460 CDS COG1116 4678486 4679235 - [P] ABC-type nitrate/sulfonate/bicarbonate transport system, ATPase component
MJLIPHIC_04461 CDS COG0600 4679222 4680112 - [P] ABC-type nitrate/sulfonate/bicarbonate transport system, permease component
MJLIPHIC_04462 CDS COG0607 4680109 4681665 - [P] Rhodanese-related sulfurtransferase
MJLIPHIC_04463 CDS COG2141 4681779 4683140 - [C] Coenzyme F420-dependent N5,N10-methylene tetrahydromethanopterin reductase and
2227
MJLIPHIC_04464 CDS COG1960 4683272 4684516 + [I] Acyl-CoA dehydrogenases
MJLIPHIC_04465 CDS NA 4684539 4685030 + NA
2228
MJLIPHIC_04466 CDS NA 4685647 4685997 + NA
2229
MJLIPHIC_04467 CDS NA 4686360 4687001 + NA
2230
MJLIPHIC_04468 CDS COG1835 4687021 4687713 - [I] Predicted acyltransferases
MJLIPHIC_04469 CDS NA 4687710 4688258 - NA
2231
MJLIPHIC_04470 CDS NA 4688858 4690024 - NA
2232
MJLIPHIC_04471 CDS COG2207 4690381 4691154 - [K] AraC-type DNA-binding domain-containing proteins
2233
MJLIPHIC_04472 CDS COG0730 4691204 4692046 + [R] Predicted permeases
2234
MJLIPHIC_04473 CDS NA 4692127 4692264 - NA
2235
MJLIPHIC_04474 CDS COG0666 4692393 4692644 - [R] FOG: Ankyrin repeat
MJLIPHIC_04475 CDS COG0666 4692657 4692797 - [R] FOG: Ankyrin repeat
2236
MJLIPHIC_04476 CDS ROG0200 4693630 4694682 + NA
2237
MJLIPHIC_04477 CDS COG3473 4695322 4696107 + [Q] Maleate cis-trans isomerase
MJLIPHIC_04478 CDS NA 4696122 4697426 + NA
2238
MJLIPHIC_04479 CDS ROG5591 4697519 4697881 + NA
MJLIPHIC_04480 CDS ROG0833 4697866 4697982 + NA
2239
MJLIPHIC_04481 CDS COG4585 4698213 4698959 - [T] Signal transduction histidine kinase
2240
MJLIPHIC_04482 CDS COG0305 4699433 4700155 - [L] Replicative DNA helicase
MJLIPHIC_04483 CDS COG0305 4700152 4700397 - [L] Replicative DNA helicase
2241
MJLIPHIC_04484 CDS NA 4700604 4700966 + NA
2242
MJLIPHIC_04485 CDS COG0171 4700974 4703016 - [H] NAD synthase
2243
MJLIPHIC_04486 CDS COG1335 4703074 4703613 + [Q] Amidases related to nicotinamidase
MJLIPHIC_04487 CDS COG0846 4703663 4704511 + [K] NAD-dependent protein deacetylases, SIR2 family
2244
MJLIPHIC_04488 CDS COG2124 4704480 4705739 - [Q] Cytochrome P450
2245
MJLIPHIC_04489 CDS COG1309 4705806 4706447 + [K] Transcriptional regulator
2246
MJLIPHIC_04490 CDS COG0263 4706444 4707556 - [E] Glutamate 5-kinase
MJLIPHIC_04491 CDS COG0536 4707553 4709010 - [R] Predicted GTPase
MJLIPHIC_04492 CDS COG0211 4709102 4709368 - [J] Ribosomal protein L27
MJLIPHIC_04493 CDS COG0261 4709385 4709696 - [J] Ribosomal protein L21
2247
MJLIPHIC_04494 CDS COG1530 4709887 4713000 - [J] Ribonucleases G and E

```
2248
        MJLIPHIC_04495  CDS     COG0105 4713398 4713817 -       [F] Nucleoside diphosphate kinase
2249
        MJLIPHIC_04496  CDS     ROG1966 4713903 4714307 -       NA
        MJLIPHIC_04497  CDS     COG0285 4714304 4715521 -       [H] Folylpolyglutamate synthase
        MJLIPHIC_04498  CDS     COG0525 4715751 4718369 -       [J] Valyl-tRNA synthetase
2250
        MJLIPHIC_04499  CDS     ROG7888 4718475 4719089 -       NA
2251
        MJLIPHIC_04500  CDS     COG3268 4719210 4720460 -       [S] Uncharacterized conserved protein
        MJLIPHIC_04501  CDS     COG1506 4720471 4722462 -       [E] Dipeptidyl aminopeptidases/acylaminoacyl-peptidases
2252
        MJLIPHIC_04502  CDS     COG0004 4722620 4723969 +       [P] Ammonia permease
        MJLIPHIC_04503  CDS     ROG7154 4723973 4724191 +       NA
2253
        MJLIPHIC_04504  CDS     ROG0247 4724276 4725268 -       NA
        MJLIPHIC_04505  CDS     COG1018 4725265 4726206 -       [C] Flavodoxin reductases (ferredoxin-NADPH reductases) family 1
        MJLIPHIC_04506  CDS     ROG1559 4726203 4726733 -       NA
2254
        MJLIPHIC_04507  CDS     COG0665 4726899 4728047 +       [E] Glycine/D-amino acid oxidases (deaminating)
2255
        MJLIPHIC_04508  CDS     ROG8173 4728044 4728529 -       NA
        MJLIPHIC_04509  CDS     ROG8173 4728762 4729085 -       NA
2256
        MJLIPHIC_04510  CDS     COG0746 4730153 4730662 -       [H] Molybdopterin-guanine dinucleotide biosynthesis protein A
        MJLIPHIC_04511  CDS     COG1013 4730736 4731818 -       [C] Pyruvate:ferredoxin oxidoreductase and related 2-oxoacid:ferredoxin
oxidoreductases,
        MJLIPHIC_04512  CDS     COG0674 4731815 4733770 -       [C] Pyruvate:ferredoxin oxidoreductase and related 2-oxoacid:ferredoxin
oxidoreductases,
2257
        MJLIPHIC_04513  CDS     COG0524 4734131 4734979 -       [G] Sugar kinases, ribokinase family
        MJLIPHIC_04514  CDS     COG1609 4735102 4736106 -       [K] Transcriptional regulators
2258
        MJLIPHIC_04515  CDS     COG5285 4736191 4737387 +       [Q] Protein involved in biosynthesis of mitomycin
        MJLIPHIC_04516  CDS     COG0673 4737384 4738391 +       [R] Predicted dehydrogenases and related proteins
        MJLIPHIC_04517  CDS     COG1082 4738388 4739257 +       [G] Sugar phosphate isomerases/epimerases
        MJLIPHIC_04518  CDS     COG0673 4739254 4740246 +       [R] Predicted dehydrogenases and related proteins
        MJLIPHIC_04519  CDS     COG3622 4740243 4741019 +       [G] Hydroxypyruvate isomerase
2259
        MJLIPHIC_04520  CDS     COG0667 4741023 4742108 -       [C] Predicted oxidoreductases (related to aryl-alcohol dehydrogenases)
2260
        MJLIPHIC_04521  CDS     COG2207 4742250 4743161 +       [K] AraC-type DNA-binding domain-containing proteins
2261
        MJLIPHIC_04522  CDS     COG1129 4743236 4744054 -       [G] ABC-type sugar transport system, ATPase component
        MJLIPHIC_04523  CDS     COG1172 4744059 4745117 -       [G] Ribose/xylose/arabinose/galactoside ABC-type transport systems, permease
components
        MJLIPHIC_04524  CDS     COG1879 4745120 4746109 -       [G] ABC-type sugar transport system, periplasmic component
2262
        MJLIPHIC_04525  CDS     COG2188 4746349 4747086 -       [K] Transcriptional regulators
2263
        MJLIPHIC_04526  CDS     COG0524 4747223 4748260 +       [G] Sugar kinases, ribokinase family
        MJLIPHIC_04527  CDS     ROG1454 4748253 4749137 +       NA
        MJLIPHIC_04528  CDS     COG3718 4749139 4750014 +       [G] Uncharacterized enzyme involved in inositol metabolism
        MJLIPHIC_04529  CDS     COG3962 4750019 4751968 +       [E] Acetolactate synthase
        MJLIPHIC_04530  CDS     COG1082 4751978 4752898 +       [G] Sugar phosphate isomerases/epimerases
        MJLIPHIC_04531  CDS     COG0673 4752923 4753960 +       [R] Predicted dehydrogenases and related proteins
2264
        MJLIPHIC_04532  CDS     COG0583 4753970 4754908 -       [K] Transcriptional regulator
2265
        MJLIPHIC_04533  CDS     COG0243 4755030 4757345 +       [C] Anaerobic dehydrogenases, typically selenocysteine-containing
        MJLIPHIC_04534  CDS     COG1526 4757345 4758169 +       [C] Uncharacterized protein required for formate dehydrogenase
2266
        MJLIPHIC_04535  CDS     COG1819 4758307 4759647 +       [GC] Glycosyl transferases, related to UDP-glucuronosyltransferase
2267
        MJLIPHIC_04536  CDS     COG1219 4759715 4760995 -       [O] ATP-dependent protease Clp, ATPase subunit
        MJLIPHIC_04537  CDS     COG0740 4761452 4762108 -       [OU] Protease subunit of ATP-dependent Clp proteases
        MJLIPHIC_04538  CDS     COG0740 4762105 4762716 -       [OU] Protease subunit of ATP-dependent Clp proteases
        MJLIPHIC_04539  CDS     COG0544 4762826 4764235 -       [O] FKBP-type peptidyl-prolyl cis-trans isomerase (trigger factor)
        MJLIPHIC_04540  tRNA    NA      4764297 4764371 -       NA
2268
        MJLIPHIC_04541  tRNA    NA      4764493 4764564 +       NA
2269
        MJLIPHIC_04542  CDS     ROG3195 4764745 4765266 +       NA
        MJLIPHIC_04543  CDS     ROG6444 4765263 4765766 +       NA
2270
        MJLIPHIC_04544  CDS     COG1680 4765898 4767085 +       [V] Beta-lactamase class C and other penicillin
        MJLIPHIC_04545  CDS     ROG5000 4767137 4767955 +       NA
2271
        MJLIPHIC_04546  CDS     COG0025 4767975 4769675 -       [P] NhaP-type Na+/H+ and K+/H+ antiporters
        MJLIPHIC_04547  CDS     COG0266 4769713 4770519 -       [L] Formamidopyrimidine-DNA glycosylase
        MJLIPHIC_04548  CDS     COG0698 4770535 4771014 -       [G] Ribose 5-phosphate isomerase RpiB
2272
        MJLIPHIC_04549  CDS     COG0667 4771078 4772004 +       [C] Predicted oxidoreductases (related to aryl-alcohol dehydrogenases)
2273
        MJLIPHIC_04550  CDS     COG3491 4771960 4772919 -       [R] Isopenicillin N synthase and related dioxygenases
        MJLIPHIC_04551  CDS     COG0590 4772916 4773341 -       [FJ] Cytosine/adenosine deaminases
2274
        MJLIPHIC_04552  CDS     ROG0317 4773420 4774028 -       NA
2275
        MJLIPHIC_04553  CDS     NA      4774279 4774683 +       NA
        MJLIPHIC_04554  CDS     COG0308 4774721 4777303 +       [E] Aminopeptidase N
2276
        MJLIPHIC_04555  CDS     COG1403 4777394 4777744 +       [V] Restriction endonuclease
2277
        MJLIPHIC_04556  CDS     ROG1790 4777808 4778281 -       NA
        MJLIPHIC_04557  CDS     NA      4778268 4778462 -       NA
2278
        MJLIPHIC_04558  CDS     COG1403 4778559 4779203 -       [V] Restriction endonuclease
2279
        MJLIPHIC_04559  CDS     COG2346 4779339 4779743 +       [R] Truncated hemoglobins
```

MJLIPHIC_04560 CDS COG0366 4779743 4781281 + [G] Glycosidases
2280
MJLIPHIC_04561 CDS ROG1468 4781282 4781938 - NA
MJLIPHIC_04562 CDS COG0824 4781935 4782342 - [R] Predicted thioesterase
MJLIPHIC_04563 CDS COG2902 4782372 4787213 - [E] NAD-specific glutamate dehydrogenase
2281
MJLIPHIC_04564 CDS COG0488 4787325 4788998 - [R] ATPase components of ABC transporters with
MJLIPHIC_04565 CDS COG0629 4789106 4789612 - [L] Single-stranded DNA-binding protein
2282
MJLIPHIC_04566 CDS COG3336 4789876 4791894 - [S] Predicted membrane protein
2283
MJLIPHIC_04567 CDS COG2937 4791964 4794324 - [I] Glycerol-3-phosphate O-acyltransferase
MJLIPHIC_04568 CDS COG0204 4794321 4795847 - [I] 1-acyl-sn-glycerol-3-phosphate acyltransferase
MJLIPHIC_04569 CDS ROG0037 4795874 4797340 - NA
2284
MJLIPHIC_04570 tRNA NA 4797480 4797553 + NA
2285
MJLIPHIC_04571 CDS COG0596 4797653 4798507 - [R] Predicted hydrolases or acyltransferases (alpha/beta hydrolase
2286
MJLIPHIC_04572 CDS COG0500 4798800 4799402 + [QR] SAM-dependent methyltransferases
MJLIPHIC_04573 CDS COG1024 4799413 4800162 + [I] Enoyl-CoA hydratase/carnithine racemase
2287
MJLIPHIC_04574 CDS COG0508 4800165 4801385 - [C] Pyruvate/2-oxoglutarate dehydrogenase complex, dihydrolipoamide acyltransferase
(E2)
MJLIPHIC_04575 CDS COG0022 4801382 4802458 - [C] Pyruvate/2-oxoglutarate dehydrogenase complex, dehydrogenase (E1) component,
MJLIPHIC_04576 CDS COG1071 4802460 4803572 - [C] Pyruvate/2-oxoglutarate dehydrogenase complex, dehydrogenase (E1) component,
2288
MJLIPHIC_04577 CDS COG2301 4803784 4804593 - [G] Citrate lyase beta subunit
MJLIPHIC_04578 CDS COG2030 4804593 4805093 - [I] Acyl dehydratase
MJLIPHIC_04579 CDS COG1960 4805090 4806253 - [I] Acyl-CoA dehydrogenases
MJLIPHIC_04580 CDS COG4770 4806250 4808253 - [I] Acetyl/propionyl-CoA carboxylase, alpha subunit
MJLIPHIC_04581 CDS COG4799 4808256 4809797 - [I] Acetyl-CoA carboxylase, carboxyltransferase component (subunits alpha
2289
MJLIPHIC_04582 CDS COG1309 4809888 4810493 + [K] Transcriptional regulator
2290
MJLIPHIC_04583 CDS COG1506 4810498 4812384 - [E] Dipeptidyl aminopeptidases/acylaminoacyl-peptidases
2291
MJLIPHIC_04584 CDS NA 4812503 4813300 + NA
2292
MJLIPHIC_04585 CDS COG2270 4813302 4814639 - [R] Permeases of the major facilitator superfamily
2293
MJLIPHIC_04586 CDS COG0300 4814674 4815444 + [R] Short-chain dehydrogenases of various substrate specificities
2294
MJLIPHIC_04587 CDS COG0433 4815452 4817023 - [R] Predicted ATPase
2295
MJLIPHIC_04588 CDS COG1949 4817102 4817773 + [A] Oligoribonuclease (3'->5' exoribonuclease)
MJLIPHIC_04589 tRNA NA 4817832 4817906 + NA
2296
MJLIPHIC_04590 CDS NA 4818029 4818226 + NA
2297
MJLIPHIC_04591 CDS COG3321 4819243 4825539 + [Q] Polyketide synthase modules and related proteins
MJLIPHIC_04592 CDS NA 4825597 4827039 + NA
MJLIPHIC_04593 CDS ROG6266 4827057 4828424 + NA
MJLIPHIC_04594 CDS ROG6266 4828501 4829868 + NA
MJLIPHIC_04595 CDS COG0318 4829885 4831618 + [IQ] Acyl-CoA synthetases (AMP-forming)/AMP-acid ligases II
MJLIPHIC_04596 CDS COG1216 4831623 4832453 + [R] Predicted glycosyltransferases
2298
MJLIPHIC_04597 CDS ROG2658 4832458 4833228 - NA
MJLIPHIC_04598 CDS NA 4833240 4835105 - NA
MJLIPHIC_04599 CDS NA 4835122 4836006 - NA
2299
MJLIPHIC_04600 CDS COG2327 4836134 4837201 - [S] Uncharacterized conserved protein
MJLIPHIC_04601 CDS COG2327 4837198 4838274 - [S] Uncharacterized conserved protein
2300
MJLIPHIC_04602 CDS NA 4838569 4839141 + NA
2301
MJLIPHIC_04603 CDS ROG4526 4839149 4839829 - NA
2302
MJLIPHIC_04604 CDS COG1819 4839918 4841168 - [GC] Glycosyl transferases, related to UDP-glucuronosyltransferase
2303
MJLIPHIC_04605 CDS COG2409 4841298 4844360 + [R] Predicted drug exporters of the RND
2304
MJLIPHIC_04606 CDS COG4221 4844394 4845170 - [R] Short-chain alcohol dehydrogenase of unknown specificity
2305
MJLIPHIC_04607 CDS COG4312 4845342 4846067 + [S] Uncharacterized protein conserved in bacteria
MJLIPHIC_04608 CDS ROG4697 4846064 4846534 + NA
2306
MJLIPHIC_04609 CDS NA 4846538 4847758 - NA
2307
MJLIPHIC_04610 tRNA NA 4847908 4847983 + NA
2308
MJLIPHIC_04611 CDS NA 4848080 4848250 - NA
2309
MJLIPHIC_04612 CDS NA 4849104 4849265 + NA
2310
MJLIPHIC_04613 CDS ROG2657 4849354 4849581 - NA
2311
MJLIPHIC_04614 CDS COG1225 4849686 4850159 + [O] Peroxiredoxin
2312
MJLIPHIC_04615 CDS COG0530 4850171 4851178 - [P] Ca2+/Na+ antiporter
MJLIPHIC_04616 CDS COG0624 4851185 4852513 - [E] Acetylornithine deacetylase/Succinyl-diaminopimelate desuccinylase and related
deacylases
MJLIPHIC_04617 CDS COG0736 4852549 4852941 - [I] Phosphopantetheinyl transferase (holo-ACP synthase)
MJLIPHIC_04618 CDS COG4982 4852999 4862268 - [I] 3-oxoacyl-[acyl-carrier protein] reductase
2313
MJLIPHIC_04619 CDS ROG3756 4862862 4863530 - NA
2314
MJLIPHIC_04620 CDS COG3842 4863586 4864692 + [E] ABC-type spermidine/putrescine transport systems, ATPase components
MJLIPHIC_04621 CDS COG0687 4864692 4865918 + [E] Spermidine/putrescine-binding periplasmic protein
MJLIPHIC_04622 CDS COG1176 4865897 4866736 + [E] ABC-type spermidine/putrescine transport system, permease component

	MJLIPHIC_04623	CDS	COG1177	4866736	4867536	+	[E] ABC-type spermidine/putrescine transport system, permease component
2315							
	MJLIPHIC_04624	CDS	ROG6460	4867557	4868219	-	NA
2316							
	MJLIPHIC_04625	CDS	COG3104	4868498	4869766	+	[E] Dipeptide/tripeptide permease
2317							
	MJLIPHIC_04626	CDS	COG4292	4869751	4871082	-	[S] Predicted membrane protein
2318							
	MJLIPHIC_04627	CDS	COG3629	4871212	4871703	+	[T] DNA-binding transcriptional activator of the SARP
	MJLIPHIC_04628	CDS	NA	4871700	4871825	+	NA
2319							
	MJLIPHIC_04629	CDS	ROG8378	4871838	4872440	-	NA
	MJLIPHIC_04630	CDS	ROG5477	4872437	4873348	-	NA
2320							
	MJLIPHIC_04631	CDS	COG0318	4873491	4875002	-	[IQ] Acyl-CoA synthetases (AMP-forming)/AMP-acid ligases II
	MJLIPHIC_04632	CDS	COG2159	4874999	4876189	-	[R] Predicted metal-dependent hydrolase of the TIM-barrel
	MJLIPHIC_04633	CDS	COG0318	4876225	4877802	-	[IQ] Acyl-CoA synthetases (AMP-forming)/AMP-acid ligases II
2321							
	MJLIPHIC_04634	CDS	COG1024	4877859	4878575	+	[I] Enoyl-CoA hydratase/carnithine racemase
2322							
	MJLIPHIC_04635	CDS	ROG2119	4878596	4879120	-	NA
2323							
	MJLIPHIC_04636	CDS	COG1545	4879224	4879655	+	[R] Predicted nucleic-acid-binding protein containing a Zn-ribbon
	MJLIPHIC_04637	CDS	ROG0072	4879668	4880813	+	NA
2324							
	MJLIPHIC_04638	CDS	COG1309	4880896	4881501	-	[K] Transcriptional regulator
2325							
	MJLIPHIC_04639	CDS	COG2124	4881695	4883008	+	[Q] Cytochrome P450
2326							
	MJLIPHIC_04640	CDS	NA	4883097	4883573	+	NA
	MJLIPHIC_04641	CDS	NA	4883577	4883996	+	NA
2327							
	MJLIPHIC_04642	CDS	NA	4884016	4884420	-	NA
2328							
	MJLIPHIC_04643	CDS	NA	4884580	4885089	+	NA
2329							
	MJLIPHIC_04644	CDS	COG1463	4885100	4886830	-	[Q] ABC-type transport system involved in resistance
	MJLIPHIC_04645	CDS	COG1463	4886830	4888230	-	[Q] ABC-type transport system involved in resistance
	MJLIPHIC_04646	CDS	COG1463	4888227	4889687	-	[Q] ABC-type transport system involved in resistance
	MJLIPHIC_04647	CDS	COG1463	4889687	4890154	-	[Q] ABC-type transport system involved in resistance
2330							
	MJLIPHIC_04648	CDS	COG3464	4890856	4891767	+	[L] Transposase and inactivated derivatives
2331							
	MJLIPHIC_04649	CDS	ROG0016	4891775	4892527	-	NA
	MJLIPHIC_04650	CDS	COG1463	4892576	4893037	-	[Q] ABC-type transport system involved in resistance
	MJLIPHIC_04651	CDS	COG1463	4893037	4894062	-	[Q] ABC-type transport system involved in resistance
	MJLIPHIC_04652	CDS	COG1463	4894059	4895333	-	[Q] ABC-type transport system involved in resistance
	MJLIPHIC_04653	CDS	COG0767	4895343	4896200	-	[Q] ABC-type transport system involved in resistance
	MJLIPHIC_04654	CDS	COG0767	4896209	4897006	-	[Q] ABC-type transport system involved in resistance
2332							
	MJLIPHIC_04655	CDS	COG1804	4897386	4898594	-	[C] Predicted acyl-CoA transferases/carnitine dehydratase
	MJLIPHIC_04656	CDS	COG1012	4898623	4900095	-	[C] NAD-dependent aldehyde dehydrogenases
2333							
	MJLIPHIC_04657	CDS	COG1028	4900364	4901065	+	[IQR] Dehydrogenases with different specificities (related to
	MJLIPHIC_04658	CDS	COG1028	4901113	4901985	+	[IQR] Dehydrogenases with different specificities (related to
	MJLIPHIC_04659	CDS	COG1141	4902029	4902220	+	[C] Ferredoxin
	MJLIPHIC_04660	CDS	COG2124	4902298	4903659	+	[Q] Cytochrome P450
2334							
	MJLIPHIC_04661	CDS	COG3807	4903779	4904126	+	[S] Uncharacterized protein conserved in bacteria
2335							
	MJLIPHIC_04662	CDS	COG1960	4904142	4905317	-	[I] Acyl-CoA dehydrogenases
	MJLIPHIC_04663	CDS	COG1960	4905320	4906324	-	[I] Acyl-CoA dehydrogenases
2336							
	MJLIPHIC_04664	CDS	COG2159	4906595	4907713	+	[R] Predicted metal-dependent hydrolase of the TIM-barrel
	MJLIPHIC_04665	CDS	COG2159	4907734	4908876	+	[R] Predicted metal-dependent hydrolase of the TIM-barrel
	MJLIPHIC_04666	CDS	COG2146	4908876	4909268	+	[PR] Ferredoxin subunits of nitrite reductase and
	MJLIPHIC_04667	CDS	COG0596	4909265	4910176	+	[R] Predicted hydrolases or acyltransferases (alpha/beta hydrolase
2337							
	MJLIPHIC_04668	CDS	NA	4910179	4910655	-	NA
2338							
	MJLIPHIC_04669	CDS	COG1894	4910683	4911978	+	[C] NADH:ubiquinone oxidoreductase, NADH-binding (51 kD) subunit
	MJLIPHIC_04670	CDS	COG1141	4911975	4912205	+	[C] Ferredoxin
	MJLIPHIC_04671	CDS	COG1545	4912253	4913908	+	[R] Predicted nucleic-acid-binding protein containing a Zn-ribbon
	MJLIPHIC_04672	CDS	COG0596	4913911	4914807	+	[R] Predicted hydrolases or acyltransferases (alpha/beta hydrolase
	MJLIPHIC_04673	CDS	COG2084	4914807	4915625	+	[I] 3-hydroxyisobutyrate dehydrogenase and related beta-hydroxyacid dehydrogenases
	MJLIPHIC_04674	CDS	COG2084	4915625	4916416	+	[I] 3-hydroxyisobutyrate dehydrogenase and related beta-hydroxyacid dehydrogenases
2339							
	MJLIPHIC_04675	CDS	COG2124	4916557	4917759	+	[Q] Cytochrome P450
	MJLIPHIC_04676	CDS	COG1141	4917759	4917959	+	[C] Ferredoxin
2340							
	MJLIPHIC_04677	CDS	COG2141	4917967	4918878	-	[C] Coenzyme F420-dependent N5,N10-methylene tetrahydromethanopterin reductase and
	MJLIPHIC_04678	CDS	COG5470	4918894	4919184	-	[S] Uncharacterized conserved protein
	MJLIPHIC_04679	CDS	COG1141	4919227	4919415	-	[C] Ferredoxin
	MJLIPHIC_04680	CDS	COG2124	4919412	4920689	-	[Q] Cytochrome P450
2341							
	MJLIPHIC_04681	CDS	NA	4920923	4921702	+	NA
2342							
	MJLIPHIC_04682	CDS	COG1309	4921789	4922448	+	[K] Transcriptional regulator
	MJLIPHIC_04683	CDS	COG1960	4922448	4923749	+	[I] Acyl-CoA dehydrogenases
	MJLIPHIC_04684	CDS	COG3173	4923746	4924777	+	[R] Predicted aminoglycoside phosphotransferase
	MJLIPHIC_04685	CDS	COG0604	4924842	4925753	+	[CR] NADPH:quinone reductase and related Zn-dependent oxidoreductases
	MJLIPHIC_04686	CDS	COG2124	4925880	4927070	+	[Q] Cytochrome P450
2343							
	MJLIPHIC_04687	CDS	NA	4927084	4927587	-	NA
	MJLIPHIC_04688	CDS	COG1309	4927584	4928201	-	[K] Transcriptional regulator
	MJLIPHIC_04689	CDS	COG1960	4928318	4929466	-	[I] Acyl-CoA dehydrogenases
	MJLIPHIC_04690	CDS	COG0183	4929479	4930627	-	[I] Acetyl-CoA acetyltransferase
	MJLIPHIC_04691	CDS	COG1804	4930620	4931879	-	[C] Predicted acyl-CoA transferases/carnitine dehydratase
	MJLIPHIC_04692	CDS	COG1028	4931885	4932643	-	[IQR] Dehydrogenases with different specificities (related to
	MJLIPHIC_04693	CDS	COG1960	4932660	4933862	-	[I] Acyl-CoA dehydrogenases

MJLIPHIC_04694 CDS COG2159 4933892 4935187 - [R] Predicted metal-dependent hydrolase of the TIM-barrel
2344
MJLIPHIC_04695 CDS NA 4935302 4935427 + NA
MJLIPHIC_04696 CDS ROG3446 4935469 4935999 + NA
MJLIPHIC_04697 CDS COG4638 4936035 4937378 + [PR] Phenylpropionate dioxygenase and related ring-hydroxylating dioxygenases,
MJLIPHIC_04698 CDS NA 4937371 4937718 + NA
MJLIPHIC_04699 CDS COG1228 4937721 4938941 + [Q] Imidazolonepropionase and related amidohydrolases
MJLIPHIC_04700 CDS ROG2444 4938925 4939368 + NA
MJLIPHIC_04701 CDS COG1960 4939369 4940490 + [I] Acyl-CoA dehydrogenases
MJLIPHIC_04702 CDS COG1960 4940496 4941302 + [I] Acyl-CoA dehydrogenases
2345
MJLIPHIC_04703 CDS COG0447 4941334 4942155 - [H] Dihydroxynaphthoic acid synthase
2346
MJLIPHIC_04704 CDS COG1309 4942335 4942997 + [K] Transcriptional regulator
2347
MJLIPHIC_04705 CDS COG1028 4943348 4944193 + [IQR] Dehydrogenases with different specificities (related to
2348
MJLIPHIC_04706 CDS COG1028 4944203 4945003 - [IQR] Dehydrogenases with different specificities (related to
MJLIPHIC_04707 CDS COG1804 4945030 4947447 - [C] Predicted acyl-CoA transferases/carnitine dehydratase
MJLIPHIC_04708 CDS COG0447 4947453 4948409 - [H] Dihydroxynaphthoic acid synthase
MJLIPHIC_04709 CDS COG0006 4948402 4949535 - [E] Xaa-Pro aminopeptidase
MJLIPHIC_04710 CDS COG0006 4949525 4950802 - [E] Xaa-Pro aminopeptidase
MJLIPHIC_04711 CDS COG2159 4950799 4951971 - [R] Predicted metal-dependent hydrolase of the TIM-barrel
MJLIPHIC_04712 CDS COG2124 4952011 4953243 - [Q] Cytochrome P450
MJLIPHIC_04713 CDS ROG3199 4953276 4953470 - NA
MJLIPHIC_04714 CDS COG3226 4953474 4954241 - [S] Uncharacterized protein conserved in bacteria
MJLIPHIC_04715 CDS COG1028 4954289 4955095 - [IQR] Dehydrogenases with different specificities (related to
MJLIPHIC_04716 CDS COG0596 4955092 4955928 - [R] Predicted hydrolases or acyltransferases (alpha/beta hydrolase
MJLIPHIC_04717 CDS ROG0216 4955940 4956938 - NA
MJLIPHIC_04718 CDS COG1028 4956939 4957775 - [IQR] Dehydrogenases with different specificities (related to
MJLIPHIC_04719 CDS COG2141 4957779 4959089 - [C] Coenzyme F420-dependent N5,N10-methylene tetrahydromethanopterin reductase and
MJLIPHIC_04720 CDS COG1053 4959104 4960867 - [C] Succinate dehydrogenase/fumarate reductase, flavoprotein subunit
MJLIPHIC_04721 CDS ROG7549 4960864 4961784 - NA
MJLIPHIC_04722 CDS COG0654 4961732 4963420 - [HC] 2-polyprenyl-6-methoxyphenol hydroxylase and related FAD-dependent
oxidoreductases
MJLIPHIC_04723 CDS COG0596 4963428 4964336 - [R] Predicted hydrolases or acyltransferases (alpha/beta hydrolase
2349
MJLIPHIC_04724 CDS COG1414 4964467 4965192 + [K] Transcriptional regulator
MJLIPHIC_04725 CDS COG1053 4965207 4966847 + [C] Succinate dehydrogenase/fumarate reductase, flavoprotein subunit
2350
MJLIPHIC_04726 CDS COG1703 4966836 4967720 - [E] Putative periplasmic protein kinase ArgK and
2351
MJLIPHIC_04727 CDS COG1028 4967796 4968560 + [IQR] Dehydrogenases with different specificities (related to
MJLIPHIC_04728 CDS COG1028 4968645 4969424 + [IQR] Dehydrogenases with different specificities (related to
2352
MJLIPHIC_04729 CDS COG1024 4969432 4970199 - [I] Enoyl-CoA hydratase/carnithine racemase
MJLIPHIC_04730 CDS COG1960 4970204 4971292 - [I] Acyl-CoA dehydrogenases
MJLIPHIC_04731 CDS COG1960 4971279 4972109 - [I] Acyl-CoA dehydrogenases
MJLIPHIC_04732 CDS COG0318 4972087 4973457 - [IQ] Acyl-CoA synthetases (AMP-forming)/AMP-acid ligases II
2353
MJLIPHIC_04733 CDS COG1018 4973597 4973908 + [C] Flavodoxin reductases (ferredoxin-NADPH reductases) family 1
2354
MJLIPHIC_04734 CDS COG1804 4973893 4974972 - [C] Predicted acyl-CoA transferases/carnitine dehydratase
MJLIPHIC_04735 CDS NA 4974969 4975925 - NA
MJLIPHIC_04736 CDS COG2185 4975922 4976320 - [I] Methylmalonyl-CoA mutase, C-terminal domain/subunit (cobalamin-binding)
MJLIPHIC_04737 CDS COG1884 4976416 4978077 - [I] Methylmalonyl-CoA mutase, N-terminal domain/subunit
2355
MJLIPHIC_04738 CDS COG1028 4978264 4979124 + [IQR] Dehydrogenases with different specificities (related to
MJLIPHIC_04739 CDS COG0318 4979121 4980611 + [IQ] Acyl-CoA synthetases (AMP-forming)/AMP-acid ligases II
MJLIPHIC_04740 CDS NA 4980630 4980932 + NA
MJLIPHIC_04741 CDS COG0477 4980943 4982244 + [GEPR] Permeases of the major facilitator superfamily
2356
MJLIPHIC_04742 CDS ROG0938 4982241 4983317 - NA
MJLIPHIC_04743 CDS COG2124 4983314 4984516 - [Q] Cytochrome P450
2357
MJLIPHIC_04744 CDS COG1609 4984613 4985614 - [K] Transcriptional regulators
MJLIPHIC_04745 CDS COG0477 4985611 4986933 - [GEPR] Permeases of the major facilitator superfamily
2358
MJLIPHIC_04746 CDS COG2128 4987073 4987606 - [S] Uncharacterized conserved protein
MJLIPHIC_04747 CDS COG0450 4987610 4988197 - [O] Peroxiredoxin
2359
MJLIPHIC_04748 CDS COG2321 4988787 4990256 + [R] Predicted metalloprotease
2360
MJLIPHIC_04749 CDS NA 4990579 4990884 + NA
2361
MJLIPHIC_04750 CDS COG0318 4990987 4992840 + [IQ] Acyl-CoA synthetases (AMP-forming)/AMP-acid ligases II
2362
MJLIPHIC_04751 tRNA NA 4992841 4992927 - NA
2363
MJLIPHIC_04752 CDS COG4578 4993024 4993398 + [K] Glucitol operon activator
MJLIPHIC_04753 CDS ROG0032 4993399 4993743 + NA
2364
MJLIPHIC_04754 CDS COG0127 4993740 4994342 - [F] Xanthosine triphosphate pyrophosphatase
MJLIPHIC_04755 CDS COG0689 4994349 4995083 - [J] RNase PH
MJLIPHIC_04756 CDS COG1234 4995163 4995921 - [R] Metal-dependent hydrolases of the beta-lactamase superfamily
MJLIPHIC_04757 CDS COG0796 4995984 4996817 - [M] Glutamate racemase
MJLIPHIC_04758 CDS ROG0040 4996814 4997473 - NA
MJLIPHIC_04759 CDS COG0031 4997511 4998482 - [E] Cysteine synthase
MJLIPHIC_04760 CDS COG1977 4998490 4998771 - [H] Molybdopterin converting factor, small subunit
MJLIPHIC_04761 CDS COG1310 4998781 4999191 - [R] Predicted metal-dependent protease of the PAD1/JAB1
MJLIPHIC_04762 CDS COG3191 4999233 5000252 - [EQ] L-aminopeptidase/D-esterase
MJLIPHIC_04763 CDS ROG0300 5000259 5000837 - NA
MJLIPHIC_04764 CDS COG2127 5000844 5001146 - [S] Uncharacterized conserved protein
2365
MJLIPHIC_04765 CDS COG1488 5001213 5002517 + [H] Nicotinic acid phosphoribosyltransferase
MJLIPHIC_04766 CDS COG1199 5002521 5004572 + [KL] Rad3-related DNA helicases
2366
MJLIPHIC_04767 CDS COG2321 5004664 5006106 + [R] Predicted metalloprotease
2367
MJLIPHIC_04768 CDS COG4257 5006124 5006972 - [V] Streptogramin lyase

MJLIPHIC_04769 CDS COG0058 5006969 5009569 - [G] Glucan phosphorylase
2368
MJLIPHIC_04770 CDS COG3280 5009741 5011819 + [G] Maltooligosyl trehalose synthase
MJLIPHIC_04771 CDS COG0296 5011827 5014037 + [G] 1,4-alpha-glucan branching enzyme
2369
MJLIPHIC_04772 CDS COG3118 5014034 5014924 - [O] Thioredoxin domain-containing protein
MJLIPHIC_04773 CDS ROG1220 5014938 5015321 - NA
MJLIPHIC_04774 CDS COG0183 5015379 5016557 - [I] Acetyl-CoA acetyltransferase
2370
MJLIPHIC_04775 CDS ROG2119 5016652 5017149 + NA
2371
MJLIPHIC_04776 CDS ROG0427 5017167 5017466 - NA
MJLIPHIC_04777 CDS COG1637 5017466 5018140 - [L] Predicted nuclease of the RecB family
2372
MJLIPHIC_04778 CDS COG2114 5018187 5019794 + [T] Adenylate cyclase, family 3 (some proteins
MJLIPHIC_04779 CDS COG0122 5019822 5021360 + [L] 3-methyladenine DNA glycosylase/8-oxoguanine DNA glycosylase
2373
MJLIPHIC_04780 CDS COG3464 5021908 5022819 + [L] Transposase and inactivated derivatives
2374
MJLIPHIC_04781 CDS ROG0016 5022827 5023579 - NA
2375
MJLIPHIC_04782 CDS COG0350 5023632 5024129 + [L] Methylated DNA-protein cysteine methyltransferase
2376
MJLIPHIC_04783 CDS COG0766 5029849 5031153 - [M] UDP-N-acetylglucosamine enolpyruvyl transferase
2377
MJLIPHIC_04784 CDS COG2096 5031204 5031776 + [S] Uncharacterized conserved protein
2378
MJLIPHIC_04785 CDS ROG0392 5031770 5032204 - NA
MJLIPHIC_04786 CDS COG0355 5032225 5032590 - [C] FOF1-type ATP synthase, epsilon subunit (mitochondrial
MJLIPHIC_04787 CDS COG0055 5032627 5034054 - [C] FOF1-type ATP synthase, beta subunit
MJLIPHIC_04788 CDS COG0224 5034092 5035015 - [C] FOF1-type ATP synthase, gamma subunit
MJLIPHIC_04789 CDS COG0056 5035023 5036669 - [C] FOF1-type ATP synthase, alpha subunit
2379
MJLIPHIC_04790 tRNA NA 5037205 5037298 + NA
2380
MJLIPHIC_04791 CDS COG0711 5038054 5038566 - [C] FOF1-type ATP synthase, subunit b
MJLIPHIC_04792 CDS COG0636 5038576 5038836 - [C] FOF1-type ATP synthase, subunit c/Archaeal/vacuolar-type H+-ATPase,
MJLIPHIC_04793 CDS COG0356 5038938 5039714 - [C] FOF1-type ATP synthase, subunit a
MJLIPHIC_04794 CDS ROG3812 5039725 5040171 - NA
2381
MJLIPHIC_04795 CDS COG3464 5040870 5041781 + [L] Transposase and inactivated derivatives
2382
MJLIPHIC_04796 CDS ROG0016 5041789 5042541 - NA
2383
MJLIPHIC_04797 CDS COG0472 5042722 5043942 - [M] UDP-N-acetylmuramyl pentapeptide phosphotransferase/UDP-N-acetylglucosamine-1-phosphate transferase
MJLIPHIC_04798 CDS COG0009 5043950 5044615 - [J] Putative translation factor (SUA5)
MJLIPHIC_04799 CDS COG2890 5044653 5045498 - [J] Methylase of polypeptide chain release factors
MJLIPHIC_04800 CDS COG0216 5045509 5046588 - [J] Protein chain release factor A
MJLIPHIC_04801 CDS COG0254 5046684 5046911 - [J] Ribosomal protein L31
2384
MJLIPHIC_04802 CDS COG0318 5047025 5048623 - [IQ] Acyl-CoA synthetases (AMP-forming)/AMP-acid ligases II
2385
MJLIPHIC_04803 CDS COG1309 5048768 5049373 + [K] Transcriptional regulator
2386
MJLIPHIC_04804 CDS COG1158 5049428 5051422 - [K] Transcription termination factor
2387
MJLIPHIC_04805 CDS COG0083 5051708 5052661 - [E] Homoserine kinase
MJLIPHIC_04806 CDS COG0498 5052675 5053757 - [E] Threonine synthase
MJLIPHIC_04807 CDS COG0460 5053754 5055073 - [E] Homoserine dehydrogenase
MJLIPHIC_04808 CDS COG0019 5055070 5056494 - [E] Diaminopimelate decarboxylase
MJLIPHIC_04809 CDS COG0018 5056491 5058143 - [J] Arginyl-tRNA synthetase
2388
MJLIPHIC_04810 tRNA NA 5058252 5058326 + NA
2389
MJLIPHIC_04811 CDS NA 5059059 5059256 - NA
2390
MJLIPHIC_04812 CDS COG1020 5059596 5060855 + [Q] Non-ribosomal peptide synthetase modules and related
2391
MJLIPHIC_04813 CDS COG0654 5060847 5062073 - [HC] 2-polyprenyl-6-methoxyphenol hydroxylase and related FAD-dependent oxidoreductases
MJLIPHIC_04814 CDS COG1309 5062076 5062870 - [K] Transcriptional regulator
2392
MJLIPHIC_04815 CDS NA 5062989 5063390 - NA
2393
MJLIPHIC_04816 CDS COG4312 5063477 5064157 - [S] Uncharacterized protein conserved in bacteria
MJLIPHIC_04817 CDS NA 5064154 5065035 - NA
2394
MJLIPHIC_04818 CDS COG4585 5065204 5066415 + [T] Signal transduction histidine kinase
MJLIPHIC_04819 CDS COG2197 5066403 5067047 + [TK] Response regulator containing a CheY-like receiver
MJLIPHIC_04820 CDS ROG2123 5067065 5067952 + NA
2395
MJLIPHIC_04821 CDS COG2070 5067953 5068972 - [R] Dioxygenases related to 2-nitropropane dioxygenase
MJLIPHIC_04822 CDS COG1670 5068972 5069496 - [J] Acetyltransferases, including N-acetylases of ribosomal proteins
2396
MJLIPHIC_04823 CDS NA 5069546 5069935 + NA
2397
MJLIPHIC_04824 CDS COG1959 5069936 5070400 - [K] Predicted transcriptional regulator
MJLIPHIC_04825 CDS COG3467 5070449 5071123 - [R] Predicted flavin-nucleotide-binding protein
MJLIPHIC_04826 CDS COG1335 5071120 5071716 - [Q] Amidases related to nicotinamidase
MJLIPHIC_04827 CDS COG1218 5071765 5072496 - [P] 3'-Phosphoadenosine 5'-phosphosulfate (PAPS) 3'-phosphatase
MJLIPHIC_04828 CDS COG2895 5072493 5074346 - [P] GTPases - Sulfate adenylate transferase subunit
MJLIPHIC_04829 CDS COG0175 5074346 5075281 - [EH] 3'-phosphoadenosine 5'-phosphosulfate sulfotransferase (PAPS reductase)/FAD synthetase
MJLIPHIC_04830 CDS COG2223 5075384 5076751 - [P] Nitrate/nitrite transporter
MJLIPHIC_04831 CDS COG3173 5076771 5077787 - [R] Predicted aminoglycoside phosphotransferase
MJLIPHIC_04832 CDS ROG1114 5077781 5078908 - NA
2398
MJLIPHIC_04833 CDS COG1309 5078986 5079549 + [K] Transcriptional regulator
2399

	MJLIPHIC_04834	CDS	COG2050	5079613	5080017	+	[Q] Uncharacterized protein, possibly involved in aromatic
2400							
	MJLIPHIC_04835	CDS	ROG1631	5080106	5080597	-	NA
	MJLIPHIC_04836	CDS	COG1807	5080643	5082547	-	[M] 4-amino-4-deoxy-L-arabinose transferase and related glycosyltransferases of
	MJLIPHIC_04837	CDS	ROG2901	5082551	5083849	-	NA
	MJLIPHIC_04838	CDS	COG1807	5083905	5085776	-	[M] 4-amino-4-deoxy-L-arabinose transferase and related glycosyltransferases of
	MJLIPHIC_04839	CDS	COG0642	5085828	5087411	-	[T] Signal transduction histidine kinase
	MJLIPHIC_04840	CDS	COG0745	5087389	5088138	-	[TK] Response regulators consisting of a CheY-like
2401							
	MJLIPHIC_04841	CDS	NA	5088262	5088714	+	NA
2402							
	MJLIPHIC_04842	CDS	NA	5088696	5088929	-	NA
2403							
	MJLIPHIC_04843	CDS	ROG4472	5089057	5089320	-	NA
2404							
	MJLIPHIC_04844	CDS	NA	5089411	5089644	-	NA
2405							
	MJLIPHIC_04845	CDS	COG0601	5089843	5090820	+	[EP] ABC-type dipeptide/oligopeptide/nickel transport systems, permease components
	MJLIPHIC_04846	CDS	COG1173	5090817	5091749	+	[EP] ABC-type dipeptide/oligopeptide/nickel transport systems, permease components
	MJLIPHIC_04847	CDS	COG1123	5091746	5093590	+	[R] ATPase components of various ABC-type transport
	MJLIPHIC_04848	CDS	COG0747	5093630	5095336	+	[E] ABC-type dipeptide transport system, periplasmic component
2406							
	MJLIPHIC_04849	CDS	COG0596	5095326	5096555	-	[R] Predicted hydrolases or acyltransferases (alpha/beta hydrolase
2407							
	MJLIPHIC_04850	CDS	NA	5096614	5097858	+	NA
2408							
	MJLIPHIC_04851	CDS	NA	5097849	5098754	-	NA
2409							
	MJLIPHIC_04852	CDS	COG4122	5098832	5099509	+	[R] Predicted O-methyltransferase
2410							
	MJLIPHIC_04853	CDS	ROG0421	5099506	5102139	-	NA
	MJLIPHIC_04854	CDS	COG0420	5102136	5103290	-	[L] DNA repair exonuclease
2411							
	MJLIPHIC_04855	CDS	COG2062	5103405	5103905	+	[T] Phosphohistidine phosphatase SixA
2412							
	MJLIPHIC_04856	CDS	ROG6760	5103902	5104462	-	NA
	MJLIPHIC_04857	CDS	ROG7479	5104459	5104971	-	NA
2413							
	MJLIPHIC_04858	CDS	COG1132	5105176	5106912	+	[V] ABC-type multidrug transport system, ATPase and
	MJLIPHIC_04859	CDS	COG1132	5106924	5108801	+	[V] ABC-type multidrug transport system, ATPase and
2414							
	MJLIPHIC_04860	CDS	ROG3731	5108808	5109329	-	NA
2415							
	MJLIPHIC_04861	CDS	NA	5109397	5109594	-	NA
2416							
	MJLIPHIC_04862	CDS	COG2013	5109663	5110502	+	[S] Uncharacterized conserved protein
2417							
	MJLIPHIC_04863	CDS	COG2217	5110506	5112737	-	[P] Cation transport ATPase
	MJLIPHIC_04864	CDS	ROG0883	5112759	5113535	-	NA
	MJLIPHIC_04865	CDS	COG2608	5113573	5113779	-	[P] Copper chaperone
2418							
	MJLIPHIC_04866	CDS	COG3544	5113830	5114462	-	[S] Uncharacterized protein conserved in bacteria
	MJLIPHIC_04867	CDS	COG2114	5114459	5115583	-	[T] Adenylate cyclase, family 3 (some proteins
	MJLIPHIC_04868	CDS	COG3284	5115599	5116882	-	[QK] Transcriptional activator of acetoin/glycerol metabolism
	MJLIPHIC_04869	CDS	COG1028	5116927	5117706	-	[IQR] Dehydrogenases with different specificities (related to
	MJLIPHIC_04870	CDS	COG1063	5117738	5118784	-	[ER] Threonine dehydrogenase and related Zn-dependent dehydrogenases
2419							
	MJLIPHIC_04871	CDS	COG2072	5118895	5120736	+	[P] Predicted flavoprotein involved in K+ transport
	MJLIPHIC_04872	CDS	COG0726	5120775	5121482	+	[G] Predicted xylanase/chitin deacetylase
	MJLIPHIC_04873	CDS	COG3663	5121479	5121967	+	[L] G:T/U mismatch-specific DNA glycosylase
2420							
	MJLIPHIC_04874	CDS	ROG2733	5122047	5123474	+	NA
2421							
	MJLIPHIC_04875	CDS	ROG4388	5123453	5124289	-	NA
2422							
	MJLIPHIC_04876	CDS	COG2764	5124317	5124787	+	[S] Uncharacterized protein conserved in bacteria
	MJLIPHIC_04877	CDS	COG0537	5124797	5125234	+	[FGR] Diadenosine tetraphosphate (Ap4A) hydrolase and other
	MJLIPHIC_04878	CDS	ROG6930	5125238	5125693	+	NA
2423							
	MJLIPHIC_04879	CDS	COG2141	5125690	5126766	-	[C] Coenzyme F420-dependent N5,N10-methylene tetrahydromethanopterin reductase and
	MJLIPHIC_04880	CDS	COG1573	5126753	5127799	-	[L] Uracil-DNA glycosylase
2424							
	MJLIPHIC_04881	CDS	NA	5127857	5128681	+	NA
2425							
	MJLIPHIC_04882	CDS	COG1733	5128678	5129328	-	[K] Predicted transcriptional regulators
2426							
	MJLIPHIC_04883	CDS	ROG1436	5129405	5130091	+	NA
	MJLIPHIC_04884	CDS	ROG0922	5130102	5130584	+	NA
	MJLIPHIC_04885	CDS	COG0277	5130603	5131970	+	[C] FAD/FMN-containing dehydrogenases
2427							
	MJLIPHIC_04886	CDS	COG0705	5131967	5132719	-	[R] Uncharacterized membrane protein (homolog of Drosophila
2428							
	MJLIPHIC_04887	CDS	COG2124	5132901	5134121	+	[Q] Cytochrome P450
	MJLIPHIC_04888	CDS	COG1309	5134121	5134708	+	[K] Transcriptional regulator
2429							
	MJLIPHIC_04889	CDS	COG2375	5134693	5135370	-	[P] Siderophore-interacting protein
	MJLIPHIC_04890	CDS	COG1835	5135426	5136574	-	[I] Predicted acyltransferases
	MJLIPHIC_04891	CDS	COG0513	5136571	5138316	-	[LKJ] Superfamily II DNA and RNA helicases
2430							
	MJLIPHIC_04892	CDS	ROG3132	5138407	5138946	+	NA
	MJLIPHIC_04893	CDS	COG2251	5138997	5142446	+	[R] Predicted nuclease (RecB family)
2431							
	MJLIPHIC_04894	tRNA	NA	5143768	5143858	-	NA
2432							
	MJLIPHIC_04895	CDS	COG0477	5145619	5146854	-	[GEPR] Permeases of the major facilitator superfamily
2433							
	MJLIPHIC_04896	CDS	ROG1326	5147070	5147831	+	NA
2434							
	MJLIPHIC_04897	CDS	COG0567	5147981	5151664	+	[C] 2-oxoglutarate dehydrogenase complex, dehydrogenase (E1) component,
	MJLIPHIC_04898	CDS	COG0477	5151761	5153071	+	[GEPR] Permeases of the major facilitator superfamily

```
2435
        MJLIPHIC_04899  CDS     COG0024 5153068 5153832 -       [J] Methionine aminopeptidase
2436
        MJLIPHIC_04900  CDS     ROG1633 5153902 5154162 +       NA
2437
        MJLIPHIC_04901  CDS     COG4221 5154241 5155065 +       [R] Short-chain alcohol dehydrogenase of unknown specificity
2438
        MJLIPHIC_04902  CDS     COG1732 5155073 5155915 -       [M] Periplasmic glycine betaine/choline-binding (lipo)protein of an
        MJLIPHIC_04903  CDS     COG0281 5155924 5157111 -       [C] Malic enzyme
2439
        MJLIPHIC_04904  CDS     COG0598 5157213 5158280 +       [P] Mg2+ and Co2+ transporters
2440
        MJLIPHIC_04905  CDS     ROG4943 5158285 5158890 -       NA
        MJLIPHIC_04906  CDS     COG3839 5158887 5160107 -       [G] ABC-type sugar transport systems, ATPase components
        MJLIPHIC_04907  CDS     COG0395 5160112 5160948 -       [G] ABC-type sugar transport system, permease component
        MJLIPHIC_04908  CDS     COG1175 5160948 5161865 -       [G] ABC-type sugar transport systems, permease components
        MJLIPHIC_04909  CDS     COG1653 5161862 5163259 -       [G] ABC-type sugar transport system, periplasmic component
2441
        MJLIPHIC_04910  CDS     ROG0211 5163424 5163936 -       NA
2442
        MJLIPHIC_04911  CDS     ROG2222 5164005 5164454 +       NA
2443
        MJLIPHIC_04912  CDS     COG2301 5164473 5165369 -       [G] Citrate lyase beta subunit
2444
        MJLIPHIC_04913  CDS     COG2239 5165455 5166741 +       [P] Mg/Co/Ni transporter MgtE (contains CBS domain)
        MJLIPHIC_04914  CDS     COG4420 5166738 5167286 +       [S] Predicted membrane protein
2445
        MJLIPHIC_04915  CDS     ROG1401 5167401 5168702 +       NA
        MJLIPHIC_04916  CDS     COG0489 5168714 5169850 +       [D] ATPases involved in chromosome partitioning
2446
        MJLIPHIC_04917  CDS     COG1826 5169857 5170255 -       [U] Sec-independent protein secretion pathway components
        MJLIPHIC_04918  CDS     COG0265 5170258 5171754 -       [O] Trypsin-like serine proteases, typically periplasmic, contain
        MJLIPHIC_04919  CDS     ROG3063 5171803 5172201 -       NA
2447
        MJLIPHIC_04920  CDS     COG1595 5172290 5173063 -       [K] DNA-directed RNA polymerase specialized sigma subunit,
2448
        MJLIPHIC_04921  CDS     COG4122 5173240 5173872 +       [R] Predicted O-methyltransferase
2449
        MJLIPHIC_04922  CDS     COG1309 5173982 5174614 +       [K] Transcriptional regulator
        MJLIPHIC_04923  CDS     COG4555 5174611 5175513 +       [CP] ABC-type Na+ transport system, ATPase component
        MJLIPHIC_04924  CDS     COG3559 5175510 5177072 +       [M] Putative exporter of polyketide antibiotics
        MJLIPHIC_04925  CDS     COG2020 5177093 5177767 +       [O] Putative protein-S-isoprenylcysteine methyltransferase
        MJLIPHIC_04926  CDS     ROG0262 5177781 5178398 +       NA
2450
        MJLIPHIC_04927  CDS     COG0448 5178352 5179566 -       [G] ADP-glucose pyrophosphorylase
2451
        MJLIPHIC_04928  CDS     COG0438 5179776 5180924 +       [M] Glycosyltransferase
2452
        MJLIPHIC_04929  CDS     ROG0443 5180979 5181230 -       NA
        MJLIPHIC_04930  CDS     COG2818 5181263 5181892 -       [L] 3-methyladenine DNA glycosylase
        MJLIPHIC_04931  CDS     ROG1928 5181889 5182272 -       NA
        MJLIPHIC_04932  CDS     COG0463 5182312 5183271 -       [M] Glycosyltransferases involved in cell wall biogenesis
        MJLIPHIC_04933  CDS     COG0294 5183268 5184143 -       [H] Dihydropteroate synthase and related enzymes
        MJLIPHIC_04934  CDS     COG0318 5184162 5185940 -       [IQ] Acyl-CoA synthetases (AMP-forming)/AMP-acid ligases II
2453
        MJLIPHIC_04935  CDS     COG1611 5186042 5186626 -       [R] Predicted Rossmann fold nucleotide-binding protein
2454
        MJLIPHIC_04936  CDS     NA      5186680 5188770 +       NA
2455
        MJLIPHIC_04937  CDS     NA      5188843 5191101 +       NA
        MJLIPHIC_04938  CDS     NA      5191098 5191916 +       NA
        MJLIPHIC_04939  CDS     NA      5191913 5192047 +       NA
        MJLIPHIC_04940  CDS     NA      5192054 5193022 +       NA
        MJLIPHIC_04941  CDS     NA      5193071 5193205 +       NA
        MJLIPHIC_04942  CDS     NA      5193246 5193383 +       NA
        MJLIPHIC_04943  CDS     ROG0016 5193424 5194176 +       NA
2456
        MJLIPHIC_04944  CDS     COG3464 5194184 5195095 -       [L] Transposase and inactivated derivatives
2457
        MJLIPHIC_04945  CDS     COG2801 5195601 5196512 -       [L] Transposase and inactivated derivatives
        MJLIPHIC_04946  CDS     COG2963 5196509 5196844 -       [L] Transposase and inactivated derivatives
2458
        MJLIPHIC_04947  CDS     NA      5197576 5198595 +       NA
2459
        MJLIPHIC_04948  CDS     NA      5198683 5199513 +       NA
2460
        MJLIPHIC_04949  CDS     COG4231 5199494 5202979 -       [C] Indolepyruvate ferredoxin oxidoreductase, alpha and beta
2461
        MJLIPHIC_04950  CDS     COG0488 5203097 5204719 -       [R] ATPase components of ABC transporters with
2462
        MJLIPHIC_04951  CDS     COG0624 5204819 5205892 -       [E] Acetylornithine deacetylase/Succinyl-diaminopimelate desuccinylase and related
deacylases
2463
        MJLIPHIC_04952  CDS     COG2171 5205921 5206865 +       [E] Tetrahydrodipicolinate N-succinyltransferase
2464
        MJLIPHIC_04953  CDS     ROG2444 5206878 5207339 -       NA
        MJLIPHIC_04954  CDS     ROG2733 5207358 5209205 -       NA
2465
        MJLIPHIC_04955  CDS     NA      5209320 5209718 +       NA
2466
        MJLIPHIC_04956  CDS     COG0596 5209709 5210374 -       [R] Predicted hydrolases or acyltransferases (alpha/beta hydrolase
        MJLIPHIC_04957  CDS     COG2220 5210371 5211210 -       [R] Predicted Zn-dependent hydrolases of the beta-lactamase
        MJLIPHIC_04958  CDS     COG3119 5211158 5212009 -       [P] Arylsulfatase A and related enzymes
2467
        MJLIPHIC_04959  CDS     NA      5212083 5212454 +       NA
        MJLIPHIC_04960  CDS     NA      5212470 5212793 +       NA
2468
        MJLIPHIC_04961  CDS     ROG6305 5212808 5213044 -       NA
2469
        MJLIPHIC_04962  CDS     COG0318 5213151 5214557 -       [IQ] Acyl-CoA synthetases (AMP-forming)/AMP-acid ligases II
```

2470							
	MJLIPHIC_04963	CDS	COG1547	5214644	5215138	+	[S] Uncharacterized conserved protein
	MJLIPHIC_04964	CDS	COG0494	5215146	5215685	+	[LR] NTP pyrophosphohydrolases including oxidative damage repair
2471							
	MJLIPHIC_04965	CDS	COG0506	5215682	5216635	-	[E] Proline dehydrogenase
	MJLIPHIC_04966	CDS	COG1012	5216637	5218265	-	[C] NAD-dependent aldehyde dehydrogenases
2472							
	MJLIPHIC_04967	CDS	COG2508	5218349	5219926	+	[TQ] Regulator of polyketide synthase expression
2473							
	MJLIPHIC_04968	CDS	COG0436	5219928	5221031	-	[E] Aspartate/tyrosine/aromatic aminotransferase
	MJLIPHIC_04969	CDS	COG1146	5221049	5221372	-	[C] Ferredoxin
2474							
	MJLIPHIC_04970	CDS	NA	5221594	5222040	-	NA
2475							
	MJLIPHIC_04971	CDS	COG1902	5222843	5224858	+	[C] NADH:flavin oxidoreductases, Old Yellow Enzyme family
2476							
	MJLIPHIC_04972	CDS	COG2353	5224977	5225525	-	[S] Uncharacterized conserved protein
2477							
	MJLIPHIC_04973	CDS	COG1060	5225815	5228394	-	[HR] Thiamine biosynthesis enzyme ThiH and related
2478							
	MJLIPHIC_04974	CDS	COG0532	5228643	5229797	+	[J] Translation initiation factor 2 (IF-2; GTPase)
2479							
	MJLIPHIC_04975	CDS	ROG5132	5229798	5230202	-	NA
	MJLIPHIC_04976	CDS	NA	5230154	5230261	-	NA
	MJLIPHIC_04977	CDS	COG2120	5230310	5231182	-	[S] Uncharacterized proteins, LmbE homologs
	MJLIPHIC_04978	CDS	COG0747	5231207	5233090	-	[E] ABC-type dipeptide transport system, periplasmic component
2480							
	MJLIPHIC_04979	CDS	NA	5233586	5234785	+	NA
2481							
	MJLIPHIC_04980	CDS	COG1217	5234873	5236774	-	[T] Predicted membrane GTPase involved in stress
2482							
	MJLIPHIC_04981	CDS	NA	5237084	5237347	+	NA
2483							
	MJLIPHIC_04982	CDS	ROG1141	5237353	5237805	-	NA
2484							
	MJLIPHIC_04983	CDS	COG2181	5237887	5238618	-	[C] Nitrate reductase gamma subunit
	MJLIPHIC_04984	CDS	COG2180	5238618	5239256	-	[C] Nitrate reductase delta subunit
	MJLIPHIC_04985	CDS	COG1140	5239253	5240899	-	[C] Nitrate reductase beta subunit
	MJLIPHIC_04986	CDS	COG5013	5240896	5244570	-	[C] Nitrate reductase alpha subunit
	MJLIPHIC_04987	CDS	COG2223	5244596	5245825	-	[P] Nitrate/nitrite transporter
	MJLIPHIC_04988	CDS	COG2723	5245838	5247265	-	[G] Beta-glucosidase/6-phospho-beta-glucosidase/beta-galactosidase
	MJLIPHIC_04989	CDS	COG1609	5247286	5248347	-	[K] Transcriptional regulators
	MJLIPHIC_04990	CDS	COG1472	5248344	5250791	-	[G] Beta-glucosidase-related glycosidases
2485							
	MJLIPHIC_04991	CDS	COG1653	5251100	5252485	+	[G] ABC-type sugar transport system, periplasmic component
	MJLIPHIC_04992	CDS	COG1175	5252482	5253402	+	[G] ABC-type sugar transport systems, permease components
	MJLIPHIC_04993	CDS	COG0395	5253404	5254303	+	[G] ABC-type sugar transport system, permease component
2486							
	MJLIPHIC_04994	CDS	COG1194	5254309	5254701	-	[L] A/G-specific DNA glycosylase
2487							
	MJLIPHIC_04995	CDS	COG2154	5254729	5255013	+	[H] Pterin-4a-carbinolamine dehydratase
2488							
	MJLIPHIC_04996	CDS	ROG8016	5254991	5256124	-	NA
2489							
	MJLIPHIC_04997	CDS	NA	5256323	5256931	+	NA
	MJLIPHIC_04998	CDS	ROG2559	5256973	5257956	+	NA
2490							
	MJLIPHIC_04999	CDS	NA	5258052	5258201	-	NA
2491							
	MJLIPHIC_05000	CDS	COG0778	5258247	5258900	+	[C] Nitroreductase
2492							
	MJLIPHIC_05001	CDS	ROG1126	5258919	5259500	-	NA
2493							
	MJLIPHIC_05002	CDS	COG4706	5259594	5259890	+	[I] Predicted 3-hydroxylacyl-(acyl carrier protein) dehydratase
	MJLIPHIC_05003	CDS	COG2972	5259912	5261111	+	[T] Predicted signal transduction protein with a
	MJLIPHIC_05004	CDS	COG3279	5261108	5261893	+	[KT] Response regulator of the LytR/AlgR family
	MJLIPHIC_05005	CDS	ROG2409	5261949	5262260	+	NA
	MJLIPHIC_05006	CDS	COG4147	5262257	5263999	+	[R] Predicted symporter
2494							
	MJLIPHIC_05007	CDS	COG1733	5264003	5264395	-	[K] Predicted transcriptional regulators
2495							
	MJLIPHIC_05008	CDS	COG2113	5264488	5265240	+	[E] ABC-type proline/glycine betaine transport systems, periplasmic
2496							
	MJLIPHIC_05009	CDS	COG0604	5265330	5266328	+	[CR] NADPH:quinone reductase and related Zn-dependent oxidoreductases
2497							
	MJLIPHIC_05010	CDS	COG3162	5266487	5266834	+	[S] Predicted membrane protein
	MJLIPHIC_05011	CDS	COG4147	5266831	5268462	+	[R] Predicted symporter
2498							
	MJLIPHIC_05012	CDS	ROG2472	5268527	5269732	+	NA
	MJLIPHIC_05013	CDS	ROG2412	5269769	5271097	+	NA
2499							
	MJLIPHIC_05014	CDS	NA	5271099	5271269	-	NA
	MJLIPHIC_05015	CDS	ROG1053	5271288	5271731	-	NA
2500							
	MJLIPHIC_05016	CDS	COG1506	5271798	5272811	+	[E] Dipeptidyl aminopeptidases/acylaminoacyl-peptidases
	MJLIPHIC_05017	CDS	COG3797	5272831	5273346	+	[S] Uncharacterized protein conserved in bacteria
	MJLIPHIC_05018	CDS	COG3315	5273370	5274206	+	[Q] O-Methyltransferase involved in polyketide biosynthesis
2501							
	MJLIPHIC_05019	CDS	COG1725	5274219	5274584	-	[K] Predicted transcriptional regulators
2502							
	MJLIPHIC_05020	CDS	COG0846	5274659	5275381	+	[K] NAD-dependent protein deacetylases, SIR2 family
2503							
	MJLIPHIC_05021	CDS	COG0500	5275385	5276035	-	[QR] SAM-dependent methyltransferases
	MJLIPHIC_05022	CDS	COG1309	5276022	5276741	-	[K] Transcriptional regulator
	MJLIPHIC_05023	CDS	COG2409	5276743	5279016	-	[R] Predicted drug exporters of the RND
2504							
	MJLIPHIC_05024	CDS	COG2259	5279234	5279794	+	[S] Predicted membrane protein
2505							
	MJLIPHIC_05025	CDS	NA	5279899	5280030	+	NA
2506							

MJLIPHIC_05026 CDS COG5421 5280066 5281577 - [L] Transposase
2507
MJLIPHIC_05027 CDS COG2070 5281804 5282817 - [R] Dioxygenases related to 2-nitropropane dioxygenase
MJLIPHIC_05028 CDS COG1028 5282834 5283595 - [IQR] Dehydrogenases with different specificities (related to
MJLIPHIC_05029 CDS COG1804 5283647 5284738 - [C] Predicted acyl-CoA transferases/carnitine dehydratase
2508
MJLIPHIC_05030 CDS COG1024 5284871 5285698 + [I] Enoyl-CoA hydratase/carnithine racemase
2509
MJLIPHIC_05031 CDS ROG3505 5285867 5286271 - NA
2510
MJLIPHIC_05032 CDS NA 5286339 5287598 + NA
2511
MJLIPHIC_05033 CDS COG1266 5287602 5288285 - [R] Predicted metal-dependent membrane protease
MJLIPHIC_05034 CDS COG0702 5288282 5289418 - [MG] Predicted nucleoside-diphosphate-sugar epimerases
MJLIPHIC_05035 CDS ROG6516 5289428 5289988 - NA
2512
MJLIPHIC_05036 CDS COG0667 5290103 5291140 + [C] Predicted oxidoreductases (related to aryl-alcohol dehydrogenases)
2513
MJLIPHIC_05037 CDS COG0663 5291112 5291633 - [R] Carbonic anhydrases/acetyltransferases, isoleucine patch superfamily
MJLIPHIC_05038 CDS COG4977 5291664 5292626 - [K] Transcriptional regulator containing an amidase domain
2514
MJLIPHIC_05039 CDS COG2271 5292709 5294022 + [G] Sugar phosphate permease
2515
MJLIPHIC_05040 CDS COG2335 5294168 5294923 + [M] Secreted and surface protein containing fasciclin-like
2516
MJLIPHIC_05041 CDS COG1960 5294997 5296139 - [I] Acyl-CoA dehydrogenases
MJLIPHIC_05042 CDS COG1024 5296145 5296939 - [I] Enoyl-CoA hydratase/carnithine racemase
MJLIPHIC_05043 CDS COG0183 5296936 5298093 - [I] Acetyl-CoA acetyltransferase
2517
MJLIPHIC_05044 CDS COG2186 5298396 5299076 + [K] Transcriptional regulators
2518
MJLIPHIC_05045 CDS NA 5299073 5299429 - NA
MJLIPHIC_05046 CDS ROG6066 5299440 5301194 - NA
MJLIPHIC_05047 CDS COG2259 5301228 5301752 - [S] Predicted membrane protein
MJLIPHIC_05048 CDS COG1028 5301775 5302539 - [IQR] Dehydrogenases with different specificities (related to
2519
MJLIPHIC_05049 CDS COG1309 5302564 5303166 + [K] Transcriptional regulator
2520
MJLIPHIC_05050 CDS ROG0037 5303163 5304428 - NA
MJLIPHIC_05051 CDS ROG3802 5304455 5305150 - NA
MJLIPHIC_05052 CDS ROG5477 5305147 5305725 - NA
2521
MJLIPHIC_05053 CDS COG0596 5305945 5306808 + [R] Predicted hydrolases or acyltransferases (alpha/beta hydrolase
MJLIPHIC_05054 CDS COG0160 5306805 5309738 + [E] 4-aminobutyrate aminotransferase and related aminotransferases
2522
MJLIPHIC_05055 CDS COG0596 5309722 5310480 - [R] Predicted hydrolases or acyltransferases (alpha/beta hydrolase
2523
MJLIPHIC_05056 CDS NA 5310644 5310811 - NA
2524
MJLIPHIC_05057 CDS ROG2740 5310838 5311533 + NA
2525
MJLIPHIC_05058 CDS COG1595 5311646 5312170 + [K] DNA-directed RNA polymerase specialized sigma subunit,
MJLIPHIC_05059 CDS ROG1229 5312178 5312690 + NA
2526
MJLIPHIC_05060 CDS ROG1880 5312778 5313185 - NA
2527
MJLIPHIC_05061 CDS COG1418 5313276 5313614 - [R] Predicted HD superfamily hydrolase
MJLIPHIC_05062 CDS COG5485 5313647 5314048 - [R] Predicted ester cyclase
2528
MJLIPHIC_05063 CDS COG1985 5314122 5314679 - [H] Pyrimidine reductase, riboflavin biosynthesis
2529
MJLIPHIC_05064 CDS COG0657 5314772 5315719 - [I] Esterase/lipase
MJLIPHIC_05065 CDS COG1670 5315767 5316321 - [J] Acetyltransferases, including N-acetylases of ribosomal proteins
MJLIPHIC_05066 CDS COG0012 5316318 5317433 - [J] Predicted GTPase, probable translation factor
2530
MJLIPHIC_05067 CDS ROG5332 5317596 5318684 + NA
2531
MJLIPHIC_05068 CDS COG0761 5318699 5319694 - [IM] Penicillin tolerance protein
2532
MJLIPHIC_05069 CDS ROG4113 5319811 5320383 + NA
MJLIPHIC_05070 CDS COG1570 5320380 5321633 + [L] Exonuclease VII, large subunit
MJLIPHIC_05071 CDS COG1722 5321630 5321842 + [L] Exonuclease VII small subunit
2533
MJLIPHIC_05072 CDS COG0702 5321905 5322975 + [MG] Predicted nucleoside-diphosphate-sugar epimerases
2534
MJLIPHIC_05073 CDS ROG3306 5322977 5323501 - NA
MJLIPHIC_05074 CDS ROG0954 5323505 5323783 - NA
2535
MJLIPHIC_05075 CDS NA 5323876 5324151 - NA
2536
MJLIPHIC_05076 CDS COG0702 5324552 5325631 + [MG] Predicted nucleoside-diphosphate-sugar epimerases
MJLIPHIC_05077 CDS COG1028 5325628 5326398 + [IQR] Dehydrogenases with different specificities (related to
MJLIPHIC_05078 CDS COG1028 5326395 5327228 + [IQR] Dehydrogenases with different specificities (related to
MJLIPHIC_05079 CDS COG0628 5327291 5328478 + [R] Predicted permease
2537
MJLIPHIC_05080 CDS COG0412 5328456 5329280 - [Q] Dienelactone hydrolase and related enzymes
MJLIPHIC_05081 CDS ROG2120 5329295 5329975 - NA
2538
MJLIPHIC_05082 CDS COG1494 5330049 5331002 + [G] Fructose-1,6-bisphosphatase/sedoheptulose 1,7-bisphosphatase and related proteins
MJLIPHIC_05083 CDS COG0114 5331034 5332440 + [C] Fumarase
2539
MJLIPHIC_05084 CDS COG4585 5332463 5334178 - [T] Signal transduction histidine kinase
2540
MJLIPHIC_05085 CDS ROG0037 5334246 5335652 - NA
MJLIPHIC_05086 CDS ROG1141 5335649 5336092 - NA
2541
MJLIPHIC_05087 CDS COG2197 5336164 5336799 - [TK] Response regulator containing a CheY-like receiver
MJLIPHIC_05088 CDS COG0589 5336810 5337652 - [T] Universal stress protein UspA and related
MJLIPHIC_05089 CDS ROG4031 5337654 5338643 - NA

2542							
	MJLIPHIC_05090	CDS	COG1875	5338763	5340076	-	[T] Predicted ATPase related to phosphate starvation-inducible
2543							
	MJLIPHIC_05091	CDS	ROG1001	5340381	5341208	-	NA
2544							
	MJLIPHIC_05092	CDS	COG0112	5341287	5342759	-	[E] Glycine/serine hydroxymethyltransferase
2545							
	MJLIPHIC_05093	CDS	ROG1390	5342839	5344104	-	NA
2546							
	MJLIPHIC_05094	CDS	COG1072	5344147	5345085	+	[H] Panthothenate kinase
2547							
	MJLIPHIC_05095	CDS	ROG5750	5345057	5346574	-	NA
2548							
	MJLIPHIC_05096	CDS	ROG1699	5346674	5347081	-	NA
	MJLIPHIC_05097	CDS	ROG1699	5347134	5347541	-	NA
2549							
	MJLIPHIC_05098	CDS	NA	5347702	5349066	-	NA
	MJLIPHIC_05099	CDS	COG0020	5349086	5349877	-	[I] Undecaprenyl pyrophosphate synthase
2550							
	MJLIPHIC_05100	CDS	COG1272	5349936	5350706	+	[R] Predicted membrane protein, hemolysin III homolog
2551							
	MJLIPHIC_05101	CDS	ROG1929	5350825	5351199	-	NA
	MJLIPHIC_05102	CDS	COG1331	5351211	5353184	-	[O] Highly conserved protein containing a thioredoxin
	MJLIPHIC_05103	CDS	ROG6103	5353204	5353482	-	NA
	MJLIPHIC_05104	CDS	COG2120	5353479	5354345	-	[S] Uncharacterized proteins, LmbE homologs
2552							
	MJLIPHIC_05105	CDS	ROG1398	5354473	5354901	+	NA
2553							
	MJLIPHIC_05106	CDS	COG0782	5355102	5355596	+	[K] Transcription elongation factor
2554							
	MJLIPHIC_05107	CDS	ROG1728	5355721	5356314	+	NA
2555							
	MJLIPHIC_05108	CDS	COG0626	5356323	5357477	-	[E] Cystathionine beta-lyases/cystathionine gamma-synthases
2556							
	MJLIPHIC_05109	CDS	COG1960	5357517	5358707	+	[I] Acyl-CoA dehydrogenases
2557							
	MJLIPHIC_05110	CDS	COG1714	5358691	5359428	-	[S] Predicted membrane protein/domain
2558							
	MJLIPHIC_05111	CDS	ROG0319	5359516	5360649	-	NA
2559							
	MJLIPHIC_05112	CDS	COG0031	5360957	5362348	-	[E] Cysteine synthase
	MJLIPHIC_05113	CDS	COG0657	5362378	5363415	-	[I] Esterase/lipase
2560							
	MJLIPHIC_05114	CDS	COG2755	5363628	5364581	+	[E] Lysophospholipase L1 and related esterases
	MJLIPHIC_05115	CDS	COG0183	5364613	5365830	+	[I] Acetyl-CoA acetyltransferase
2561							
	MJLIPHIC_05116	CDS	COG0027	5365924	5367126	+	[F] Formate-dependent phosphoribosylglycinamide formyltransferase (GAR transformylase)
2562							
	MJLIPHIC_05117	CDS	COG4760	5367202	5368038	-	[S] Predicted membrane protein
2563							
	MJLIPHIC_05118	CDS	COG1024	5368214	5369260	+	[I] Enoyl-CoA hydratase/carnithine racemase
	MJLIPHIC_05119	CDS	COG1024	5369260	5370036	+	[I] Enoyl-CoA hydratase/carnithine racemase
	MJLIPHIC_05120	CDS	COG4425	5370047	5371777	+	[S] Predicted membrane protein
2564							
	MJLIPHIC_05121	CDS	COG0607	5371788	5372156	-	[P] Rhodanese-related sulfurtransferase
	MJLIPHIC_05122	CDS	ROG0977	5372158	5372706	-	NA
2565							
	MJLIPHIC_05123	CDS	ROG4412	5372831	5373301	+	NA
2566							
	MJLIPHIC_05124	CDS	COG1309	5373318	5373962	-	[K] Transcriptional regulator
2567							
	MJLIPHIC_05125	CDS	COG2936	5374047	5375750	+	[R] Predicted acyl esterases
	MJLIPHIC_05126	CDS	COG1752	5375747	5376769	+	[R] Predicted esterase of the alpha-beta hydrolase
2568							
	MJLIPHIC_05127	CDS	COG1752	5376770	5377804	-	[R] Predicted esterase of the alpha-beta hydrolase
2569							
	MJLIPHIC_05128	CDS	COG1063	5377817	5378761	+	[ER] Threonine dehydrogenase and related Zn-dependent dehydrogenases
2570							
	MJLIPHIC_05129	CDS	COG3804	5378758	5379807	-	[S] Uncharacterized conserved protein related to dihydrodipicolinate
2571							
	MJLIPHIC_05130	CDS	COG2909	5379874	5382108	-	[K] ATP-dependent transcriptional regulator
2572							
	MJLIPHIC_05131	CDS	NA	5382453	5382716	+	NA
2573							
	MJLIPHIC_05132	CDS	COG0318	5382807	5384423	-	[IQ] Acyl-CoA synthetases (AMP-forming)/AMP-acid ligases II
2574							
	MJLIPHIC_05133	CDS	NA	5384566	5384877	+	NA
2575							
	MJLIPHIC_05134	CDS	ROG5416	5384898	5385158	-	NA
2576							
	MJLIPHIC_05135	CDS	COG0318	5385285	5386811	-	[IQ] Acyl-CoA synthetases (AMP-forming)/AMP-acid ligases II
2577							
	MJLIPHIC_05136	CDS	COG0028	5386954	5388684	+	[EH] Thiamine pyrophosphate-requiring enzymes [acetolactate synthase, pyruvate
	MJLIPHIC_05137	CDS	NA	5388719	5389042	+	NA
2578							
	MJLIPHIC_05138	CDS	NA	5389047	5389361	-	NA
2579							
	MJLIPHIC_05139	CDS	COG1028	5389451	5390179	-	[IQR] Dehydrogenases with different specificities (related to
2580							
	MJLIPHIC_05140	CDS	COG1309	5390260	5390850	+	[K] Transcriptional regulator
2581							
	MJLIPHIC_05141	CDS	COG1301	5390951	5392324	-	[C] Na+/H+-dicarboxylate symporters
2582							
	MJLIPHIC_05142	CDS	COG0591	5392572	5394098	+	[ER] Na+/proline symporter
2583							
	MJLIPHIC_05143	CDS	COG3290	5394189	5395772	+	[T] Signal transduction histidine kinase regulating citrate/malate
	MJLIPHIC_05144	CDS	COG4565	5395769	5396461	+	[KT] Response regulator of citrate/malate metabolism
2584							
	MJLIPHIC_05145	CDS	COG2303	5396446	5397849	-	[E] Choline dehydrogenase and related flavoproteins

2585							
	MJLIPHIC_05146	CDS	COG1309	5397936	5398649	+	[K] Transcriptional regulator
2586							
	MJLIPHIC_05147	CDS	COG3391	5398656	5399762	-	[S] Uncharacterized conserved protein
2587							
	MJLIPHIC_05148	CDS	COG2345	5400142	5400870	-	[K] Predicted transcriptional regulator
	MJLIPHIC_05149	CDS	COG0500	5400867	5401676	-	[QR] SAM-dependent methyltransferases
	MJLIPHIC_05150	CDS	ROG1102	5401673	5402452	-	NA
	MJLIPHIC_05151	CDS	COG4555	5402449	5403156	-	[CP] ABC-type Na+ transport system, ATPase component
	MJLIPHIC_05152	CDS	ROG4412	5403153	5403605	-	NA
2588							
	MJLIPHIC_05153	CDS	ROG3094	5403732	5404646	-	NA
2589							
	MJLIPHIC_05154	CDS	COG3409	5404783	5405610	-	[M] Putative peptidoglycan-binding domain-containing protein
2590							
	MJLIPHIC_05155	CDS	NA	5405691	5406437	+	NA
2591							
	MJLIPHIC_05156	CDS	COG1126	5406434	5407207	-	[E] ABC-type polar amino acid transport system,
	MJLIPHIC_05157	CDS	COG0765	5407204	5408679	-	[E] ABC-type amino acid transport system, permease
2592							
	MJLIPHIC_05158	CDS	COG2128	5408767	5409213	-	[S] Uncharacterized conserved protein
2593							
	MJLIPHIC_05159	CDS	NA	5409376	5409696	+	NA
2594							
	MJLIPHIC_05160	CDS	NA	5409749	5411089	-	NA
2595							
	MJLIPHIC_05161	CDS	COG3176	5411574	5412371	-	[R] Putative hemolysin
	MJLIPHIC_05162	CDS	COG3176	5412499	5413200	-	[R] Putative hemolysin
2596							
	MJLIPHIC_05163	CDS	NA	5413309	5413632	-	NA
2597							
	MJLIPHIC_05164	CDS	NA	5413873	5414196	+	NA
2598							
	MJLIPHIC_05165	CDS	ROG0095	5414534	5414827	+	NA
2599							
	MJLIPHIC_05166	CDS	COG2119	5414886	5415626	-	[S] Predicted membrane protein
2600							
	MJLIPHIC_05167	CDS	COG2222	5415960	5416961	-	[M] Predicted phosphosugar isomerases
	MJLIPHIC_05168	CDS	COG3281	5416958	5418157	-	[G] Uncharacterized protein, probably involved in trehalose
	MJLIPHIC_05169	CDS	COG1819	5418154	5419464	-	[GC] Glycosyl transferases, related to UDP-glucuronosyltransferase
	MJLIPHIC_05170	CDS	COG1309	5419476	5420024	-	[K] Transcriptional regulator
2601							
	MJLIPHIC_05171	CDS	NA	5420232	5420573	+	NA
2602							
	MJLIPHIC_05172	CDS	COG4117	5420674	5421282	-	[C] Thiosulfate reductase cytochrome B subunit (membrane
2603							
	MJLIPHIC_05173	CDS	COG2421	5421419	5422666	-	[C] Predicted acetamidase/formamidase
	MJLIPHIC_05174	CDS	ROG1393	5422683	5423324	-	NA
2604							
	MJLIPHIC_05175	CDS	COG1846	5423695	5424123	-	[K] Transcriptional regulators
2605							
	MJLIPHIC_05176	CDS	COG0683	5424226	5425299	+	[E] ABC-type branched-chain amino acid transport systems,
2606							
	MJLIPHIC_05177	CDS	COG1309	5425307	5425912	-	[K] Transcriptional regulator
2607							
	MJLIPHIC_05178	CDS	COG1073	5426007	5427203	+	[R] Hydrolases of the alpha/beta superfamily
2608							
	MJLIPHIC_05179	CDS	ROG6556	5427211	5427540	-	NA
	MJLIPHIC_05180	CDS	ROG6534	5427543	5427890	-	NA
2609							
	MJLIPHIC_05181	CDS	COG1495	5427953	5428183	-	[O] Disulfide bond formation protein DsbB
2610							
	MJLIPHIC_05182	CDS	COG2273	5428296	5429072	+	[G] Beta-glucanase/Beta-glucan synthetase
2611							
	MJLIPHIC_05183	CDS	COG1309	5429236	5429892	-	[K] Transcriptional regulator
2612							
	MJLIPHIC_05184	CDS	NA	5430067	5431389	+	NA
	MJLIPHIC_05185	CDS	COG0318	5431409	5433004	+	[IQ] Acyl-CoA synthetases (AMP-forming)/AMP-acid ligases II
	MJLIPHIC_05186	CDS	ROG2017	5433042	5433650	+	NA
2613							
	MJLIPHIC_05187	CDS	ROG2617	5433830	5435275	-	NA
2614							
	MJLIPHIC_05188	CDS	COG4729	5435376	5435558	-	[S] Uncharacterized conserved protein
2615							
	MJLIPHIC_05189	CDS	COG2388	5435666	5435977	+	[R] Predicted acetyltransferase
2616							
	MJLIPHIC_05190	CDS	COG0500	5435982	5436746	-	[QR] SAM-dependent methyltransferases
2617							
	MJLIPHIC_05191	CDS	NA	5436911	5437936	+	NA
2618							
	MJLIPHIC_05192	CDS	NA	5437985	5438560	-	NA
2619							
	MJLIPHIC_05193	CDS	NA	5438797	5439021	-	NA
2620							
	MJLIPHIC_05194	CDS	NA	5439081	5439218	+	NA
2621							
	MJLIPHIC_05195	CDS	COG2421	5439246	5440604	-	[C] Predicted acetamidase/formamidase
2622							
	MJLIPHIC_05196	CDS	COG1513	5440718	5441167	-	[P] Cyanate lyase
	MJLIPHIC_05197	CDS	COG2116	5441164	5442024	-	[P] Formate/nitrite family of transporters
2623							
	MJLIPHIC_05198	CDS	NA	5442249	5442995	+	NA
	MJLIPHIC_05199	CDS	ROG2203	5442992	5443366	+	NA
2624							
	MJLIPHIC_05200	CDS	COG2159	5443516	5444469	-	[R] Predicted metal-dependent hydrolase of the TIM-barrel
2625							
	MJLIPHIC_05201	CDS	NA	5444594	5444866	-	NA
2626							
	MJLIPHIC_05202	CDS	COG0702	5444953	5445444	+	[MG] Predicted nucleoside-diphosphate-sugar epimerases
2627							

MJLIPHIC_05203 CDS ROG3956 5445596 5445898 + NA
2628
MJLIPHIC_05204 CDS COG0834 5446176 5447081 + [ET] ABC-type amino acid transport/signal transduction systems,
MJLIPHIC_05205 CDS COG0765 5447086 5447784 + [E] ABC-type amino acid transport system, permease
MJLIPHIC_05206 CDS COG0765 5447781 5448449 + [E] ABC-type amino acid transport system, permease
MJLIPHIC_05207 CDS COG1126 5448466 5449230 + [E] ABC-type polar amino acid transport system,
2629
MJLIPHIC_05208 CDS COG2205 5449234 5451729 - [T] Osmosensitive K+ channel histidine kinase
2630
MJLIPHIC_05209 CDS COG0388 5451846 5452871 - [R] Predicted amidohydrolase
MJLIPHIC_05210 CDS COG0174 5452917 5454350 - [E] Glutamine synthetase
2631
MJLIPHIC_05211 CDS COG2186 5454493 5455185 - [K] Transcriptional regulators
2632
MJLIPHIC_05212 CDS ROG8109 5455289 5455684 - NA
2633
MJLIPHIC_05213 CDS COG3464 5456841 5457752 + [L] Transposase and inactivated derivatives
2634
MJLIPHIC_05214 CDS ROG0016 5457760 5458512 - NA
2635
MJLIPHIC_05215 tRNA NA 5458910 5458984 - NA
2636
MJLIPHIC_05216 CDS NA 5459359 5460096 - NA
2637
MJLIPHIC_05217 CDS NA 5460530 5461252 - NA
2638
MJLIPHIC_05218 CDS NA 5461788 5462753 + NA
2639
MJLIPHIC_05219 CDS COG1028 5462776 5463546 - [IQR] Dehydrogenases with different specificities (related to
MJLIPHIC_05220 CDS COG0607 5463644 5465023 - [P] Rhodanese-related sulfurtransferase
2640
MJLIPHIC_05221 CDS COG0607 5465174 5465761 + [P] Rhodanese-related sulfurtransferase
MJLIPHIC_05222 CDS COG0730 5465768 5466640 + [R] Predicted permeases
MJLIPHIC_05223 CDS COG1937 5466704 5466973 + [S] Uncharacterized protein conserved in bacteria
MJLIPHIC_05224 CDS COG3439 5466991 5467392 + [S] Uncharacterized conserved protein
2641
MJLIPHIC_05225 CDS NA 5467389 5468129 - NA
2642
MJLIPHIC_05226 CDS COG2060 5468513 5470183 + [P] K+-transporting ATPase, A chain
MJLIPHIC_05227 CDS COG2216 5470183 5472330 + [P] High-affinity K+ transport system, ATPase chain
MJLIPHIC_05228 CDS COG2156 5472332 5473198 + [P] K+-transporting ATPase, c chain
MJLIPHIC_05229 CDS COG2205 5473191 5475725 + [T] Osmosensitive K+ channel histidine kinase
MJLIPHIC_05230 CDS COG0745 5475722 5476414 + [TK] Response regulators consisting of a CheY-like
2643
MJLIPHIC_05231 CDS COG0514 5476470 5477615 - [L] Superfamily II DNA helicase
MJLIPHIC_05232 CDS COG3464 5477639 5478961 - [L] Transposase and inactivated derivatives
2644
MJLIPHIC_05233 CDS COG0514 5479025 5479549 - [L] Superfamily II DNA helicase
MJLIPHIC_05234 CDS COG1233 5479546 5481138 - [Q] Phytoene dehydrogenase and related proteins
MJLIPHIC_05235 CDS ROG3296 5481135 5481614 - NA
MJLIPHIC_05236 CDS COG0300 5481632 5482396 - [R] Short-chain dehydrogenases of various substrate specificities
2645
MJLIPHIC_05237 CDS COG0365 5482552 5484423 + [I] Acyl-coenzyme A synthetases/AMP-(fatty) acid ligases
2646
MJLIPHIC_05238 CDS COG2217 5484398 5486341 - [P] Cation transport ATPase
2647
MJLIPHIC_05239 CDS COG0640 5486438 5486791 + [K] Predicted transcriptional regulators
2648
MJLIPHIC_05240 CDS ROG8208 5487241 5487942 + NA
MJLIPHIC_05241 CDS COG0682 5487967 5488752 + [M] Prolipoprotein diacylglyceryltransferase
2649
MJLIPHIC_05242 CDS COG0575 5489079 5489366 + [I] CDP-diglyceride synthetase
MJLIPHIC_05243 CDS COG1964 5489368 5490936 + [R] Predicted Fe-S oxidoreductases
MJLIPHIC_05244 CDS NA 5490933 5491193 + NA
2650
MJLIPHIC_05245 CDS NA 5491345 5492376 + NA
2651
MJLIPHIC_05246 CDS COG0248 5492439 5493389 - [FP] Exopolyphosphatase
MJLIPHIC_05247 CDS COG1507 5493380 5494549 - [S] Uncharacterized conserved protein
MJLIPHIC_05248 CDS COG0148 5494583 5495866 - [G] Enolase
MJLIPHIC_05249 CDS COG2951 5495931 5496605 - [M] Membrane-bound lytic murein transglycosylase B
2652
MJLIPHIC_05250 CDS COG0672 5496846 5497778 + [P] High-affinity Fe2+/Pb2+ permease
MJLIPHIC_05251 CDS COG2822 5497781 5498983 + [P] Predicted periplasmic lipoprotein involved in iron
MJLIPHIC_05252 CDS COG2837 5498929 5500266 + [P] Predicted iron-dependent peroxidase
2653
MJLIPHIC_05253 CDS NA 5500983 5503937 + NA
2654
MJLIPHIC_05254 CDS COG1694 5504059 5505033 - [R] Predicted pyrophosphatase
MJLIPHIC_05255 CDS COG1197 5505030 5508677 - [LK] Transcription-repair coupling factor (superfamily II helicase)
2655
MJLIPHIC_05256 CDS COG1309 5508726 5509361 - [K] Transcriptional regulator
2656
MJLIPHIC_05257 tRNA NA 5509404 5509476 + NA
2657
MJLIPHIC_05258 CDS COG1207 5509565 5511013 + [M] N-acetylglucosamine-1-phosphate uridyltransferase (contains nucleotidyltransferase and I-patch
MJLIPHIC_05259 CDS COG0462 5511115 5512095 + [FE] Phosphoribosylpyrophosphate synthetase
MJLIPHIC_05260 CDS COG1393 5512096 5512443 + [P] Arsenate reductase and related proteins, glutaredoxin
MJLIPHIC_05261 CDS NA 5512458 5513096 + NA
MJLIPHIC_05262 CDS COG3967 5513126 5513998 + [M] Short-chain dehydrogenase involved in D-alanine esterification
2658
MJLIPHIC_05263 CDS COG1825 5514158 5514805 + [J] Ribosomal protein L25 (general stress protein
MJLIPHIC_05264 CDS COG0193 5514818 5515393 + [J] Peptidyl-tRNA hydrolase
2659
MJLIPHIC_05265 CDS COG1600 5515581 5516618 - [C] Uncharacterized Fe-S protein
2660
MJLIPHIC_05266 CDS ROG0221 5516700 5517245 + NA
2661
MJLIPHIC_05267 CDS COG0318 5517344 5518981 - [IQ] Acyl-CoA synthetases (AMP-forming)/AMP-acid ligases II

2662							
	MJLIPHIC_05268	CDS	COG1947	5519402	5520364	-	[I] 4-diphosphocytidyl-2C-methyl-D-erythritol 2-phosphate synthase
2663							
	MJLIPHIC_05269	CDS	COG0515	5520425	5521288	+	[RTKL] Serine/threonine protein kinase
2664							
	MJLIPHIC_05270	CDS	COG0030	5521390	5522283	-	[J] Dimethyladenosine transferase (rRNA methylation)
	MJLIPHIC_05271	CDS	COG3583	5522319	5523392	-	[S] Uncharacterized protein conserved in bacteria
2665							
	MJLIPHIC_05272	CDS	COG0084	5523539	5524393	-	[L] Mg-dependent DNase
2666							
	MJLIPHIC_05273	CDS	COG0143	5524418	5525965	+	[J] Methionyl-tRNA synthetase
	MJLIPHIC_05274	CDS	COG0334	5525967	5527316	+	[E] Glutamate dehydrogenase/leucine dehydrogenase
	MJLIPHIC_05275	CDS	COG1252	5527410	5528603	+	[C] NADH dehydrogenase, FAD-containing subunit
	MJLIPHIC_05276	CDS	COG1595	5528600	5529505	+	[K] DNA-directed RNA polymerase specialized sigma subunit,
	MJLIPHIC_05277	CDS	COG0147	5529526	5530779	+	[EH] Anthranilate/para-aminobenzoate synthases component I
2667							
	MJLIPHIC_05278	CDS	COG0313	5530749	5531585	-	[R] Predicted methyltransferases
2668							
	MJLIPHIC_05279	CDS	COG1928	5531676	5533160	+	[O] Dolichyl-phosphate-mannose--protein O-mannosyl transferase
2669							
	MJLIPHIC_05280	CDS	ROG3094	5533545	5534486	+	NA
2670							
	MJLIPHIC_05281	CDS	COG2235	5534381	5535589	-	[E] Arginine deiminase
2671							
	MJLIPHIC_05282	CDS	COG0789	5535636	5536067	+	[K] Predicted transcriptional regulators
	MJLIPHIC_05283	CDS	COG3145	5536113	5536697	+	[L] Alkylated DNA repair protein
2672							
	MJLIPHIC_05284	CDS	NA	5536711	5537388	-	NA
	MJLIPHIC_05285	CDS	NA	5537418	5538098	-	NA
2673							
	MJLIPHIC_05286	CDS	COG3049	5538195	5539166	+	[M] Penicillin V acylase and related amidases
2674							
	MJLIPHIC_05287	CDS	NA	5539167	5540630	-	NA
2675							
	MJLIPHIC_05288	CDS	NA	5540925	5541641	+	NA
2676							
	MJLIPHIC_05289	CDS	COG0169	5541650	5542498	-	[E] Shikimate 5-dehydrogenase
	MJLIPHIC_05290	CDS	COG0664	5542495	5543496	-	[T] cAMP-binding proteins - catabolite gene activator
2677							
	MJLIPHIC_05291	CDS	NA	5543717	5544271	-	NA
	MJLIPHIC_05292	CDS	NA	5544268	5544540	-	NA
2678							
	MJLIPHIC_05293	CDS	COG2962	5545249	5546187	+	[R] Predicted permeases
	MJLIPHIC_05294	CDS	COG2084	5546238	5547116	+	[I] 3-hydroxyisobutyrate dehydrogenase and related beta-hydroxyacid dehydrogenases
	MJLIPHIC_05295	CDS	ROG0552	5547113	5548270	+	NA
	MJLIPHIC_05296	CDS	COG1028	5548286	5549011	+	[IQR] Dehydrogenases with different specificities (related to
2679							
	MJLIPHIC_05297	CDS	COG2207	5548969	5549808	-	[K] AraC-type DNA-binding domain-containing proteins
2680							
	MJLIPHIC_05298	tRNA	NA	5550051	5550124	-	NA
2681							
	MJLIPHIC_05299	CDS	ROG2329	5550191	5551345	-	NA
2682							
	MJLIPHIC_05300	CDS	COG1670	5551581	5552234	-	[J] Acetyltransferases, including N-acetylases of ribosomal proteins
	MJLIPHIC_05301	CDS	COG0303	5552239	5553456	-	[H] Molybdopterin biosynthesis enzyme
	MJLIPHIC_05302	CDS	COG1210	5553543	5554466	-	[M] UDP-glucose pyrophosphorylase
2683							
	MJLIPHIC_05303	CDS	COG0212	5554494	5555090	+	[H] 5-formyltetrahydrofolate cyclo-ligase
2684							
	MJLIPHIC_05304	CDS	COG1113	5555211	5557037	+	[E] Gamma-aminobutyrate permease and related permeases
	MJLIPHIC_05305	CDS	COG0589	5557034	5557930	+	[T] Universal stress protein UspA and related
2685							
	MJLIPHIC_05306	CDS	COG1414	5557934	5558698	-	[K] Transcriptional regulator
2686							
	MJLIPHIC_05307	CDS	COG3960	5558806	5560602	-	[R] Glyoxylate carboligase
	MJLIPHIC_05308	CDS	COG2084	5560599	5561513	-	[I] 3-hydroxyisobutyrate dehydrogenase and related beta-hydroxyacid dehydrogenases
	MJLIPHIC_05309	CDS	COG3622	5561544	5562377	-	[G] Hydroxypyruvate isomerase
2687							
	MJLIPHIC_05310	CDS	COG2331	5562618	5562938	+	[S] Uncharacterized protein conserved in bacteria
2688							
	MJLIPHIC_05311	CDS	ROG1253	5563017	5563667	+	NA
2689							
	MJLIPHIC_05312	CDS	ROG7970	5563661	5563867	-	NA
2690							
	MJLIPHIC_05313	CDS	COG1970	5564013	5564483	+	[M] Large-conductance mechanosensitive channel
2691							
	MJLIPHIC_05314	CDS	NA	5564562	5565209	-	NA
2692							
	MJLIPHIC_05315	CDS	NA	5565602	5565760	-	NA
2693							
	MJLIPHIC_05316	CDS	COG0521	5565920	5566510	-	[H] Molybdopterin biosynthesis enzymes
	MJLIPHIC_05317	CDS	COG0265	5566507	5567841	-	[O] Trypsin-like serine proteases, typically periplasmic, contain
2694							
	MJLIPHIC_05318	CDS	COG2205	5568015	5569493	-	[T] Osmosensitive K+ channel histidine kinase
	MJLIPHIC_05319	CDS	COG0745	5569498	5570190	-	[TK] Response regulators consisting of a CheY-like
2695							
	MJLIPHIC_05320	CDS	ROG1431	5570348	5570521	-	NA
2696							
	MJLIPHIC_05321	CDS	ROG0003	5570784	5572490	+	NA
	MJLIPHIC_05322	CDS	COG1960	5572487	5573635	+	[I] Acyl-CoA dehydrogenases
	MJLIPHIC_05323	CDS	COG4799	5573632	5575227	+	[I] Acetyl-CoA carboxylase, carboxyltransferase component (subunits alpha
	MJLIPHIC_05324	CDS	COG4770	5575327	5577273	+	[I] Acetyl/propionyl-CoA carboxylase, alpha subunit
	MJLIPHIC_05325	CDS	COG1960	5577270	5578430	+	[I] Acyl-CoA dehydrogenases
	MJLIPHIC_05326	CDS	COG1024	5578430	5579197	+	[I] Enoyl-CoA hydratase/carnithine racemase
	MJLIPHIC_05327	CDS	COG0668	5579252	5580490	+	[M] Small-conductance mechanosensitive channel
	MJLIPHIC_05328	CDS	ROG7623	5580542	5580670	+	NA
2697							
	MJLIPHIC_05329	CDS	NA	5580675	5581037	-	NA
	MJLIPHIC_05330	CDS	NA	5581091	5581405	-	NA
2698							

```
        MJLIPHIC_05331  CDS     NA      5581701 5584286 +       NA
2699
        MJLIPHIC_05332  CDS     NA      5584716 5586893 +       NA
2700
        MJLIPHIC_05333  CDS     NA      5587558 5588505 +       NA
        MJLIPHIC_05334  CDS     COG0702 5588558 5589319 +       [MG] Predicted nucleoside-diphosphate-sugar epimerases
2701
        MJLIPHIC_05335  CDS     ROG0549 5589415 5589999 +       NA
2702
        MJLIPHIC_05336  CDS     NA      5590070 5590732 -       NA
2703
        MJLIPHIC_05337  CDS     NA      5590921 5592249 -       NA
2704
        MJLIPHIC_05338  CDS     COG3540 5593079 5594662 -       [P] Phosphodiesterase/alkaline phosphatase D
2705
        MJLIPHIC_05339  CDS     COG2220 5594766 5595632 +       [R] Predicted Zn-dependent hydrolases of the beta-lactamase
2706
        MJLIPHIC_05340  CDS     ROG3094 5595683 5596123 -       NA
        MJLIPHIC_05341  CDS     ROG3094 5596261 5596530 -       NA
2707
        MJLIPHIC_05342  CDS     COG4867 5596653 5598653 -       [R] Uncharacterized protein with a von Willebrand
        MJLIPHIC_05343  CDS     COG1239 5598646 5600031 -       [H] Mg-chelatase subunit ChlI
2708
        MJLIPHIC_05344  CDS     COG0515 5600142 5601998 +       [RTKL] Serine/threonine protein kinase
2709
        MJLIPHIC_05345  CDS     NA      5602005 5602658 -       NA
        MJLIPHIC_05346  CDS     COG0138 5602692 5604275 -       [F] AICAR transformylase/IMP cyclohydrolase PurH (only IMP
        MJLIPHIC_05347  CDS     COG0299 5604286 5604870 -       [F] Folate-dependent phosphoribosylglycinamide formyltransferase PurN
        MJLIPHIC_05348  CDS     ROG1212 5604926 5606389 -       NA
        MJLIPHIC_05349  CDS     NA      5606450 5607349 -       NA
2710
        MJLIPHIC_05350  CDS     COG2141 5607496 5608626 -       [C] Coenzyme F420-dependent N5,N10-methylene tetrahydromethanopterin reductase and
2711
        MJLIPHIC_05351  CDS     COG2141 5608770 5609618 +       [C] Coenzyme F420-dependent N5,N10-methylene tetrahydromethanopterin reductase and
2712
        MJLIPHIC_05352  CDS     NA      5609621 5611144 -       NA
2713
        MJLIPHIC_05353  CDS     COG1309 5611326 5611943 +       [K] Transcriptional regulator
2714
        MJLIPHIC_05354  CDS     COG1506 5612016 5613869 +       [E] Dipeptidyl aminopeptidases/acylaminoacyl-peptidases
2715
        MJLIPHIC_05355  CDS     COG0074 5613938 5614840 -       [C] Succinyl-CoA synthetase, alpha subunit
        MJLIPHIC_05356  CDS     COG0045 5614853 5616016 -       [C] Succinyl-CoA synthetase, beta subunit
2716
        MJLIPHIC_05357  CDS     COG0739 5616276 5617415 +       [M] Membrane proteins related to metalloendopeptidases
        MJLIPHIC_05358  CDS     NA      5617416 5618099 +       NA
2717
        MJLIPHIC_05359  CDS     COG0715 5618274 5619242 +       [P] ABC-type nitrate/sulfonate/bicarbonate transport systems, periplasmic
components
        MJLIPHIC_05360  CDS     COG0600 5619239 5620093 +       [P] ABC-type nitrate/sulfonate/bicarbonate transport system, permease component
        MJLIPHIC_05361  CDS     COG1116 5620090 5620818 +       [P] ABC-type nitrate/sulfonate/bicarbonate transport system, ATPase component
        MJLIPHIC_05362  CDS     COG1053 5620808 5622400 +       [C] Succinate dehydrogenase/fumarate reductase, flavoprotein subunit
        MJLIPHIC_05363  CDS     COG1146 5622397 5622798 +       [C] Ferredoxin
2718
        MJLIPHIC_05364  CDS     COG0210 5622802 5625153 -       [L] Superfamily I DNA and RNA helicases
2719
        MJLIPHIC_05365  CDS     COG1605 5625390 5625656 +       [E] Chorismate mutase
2720
        MJLIPHIC_05366  CDS     COG1835 5625750 5627759 -       [I] Predicted acyltransferases
2721
        MJLIPHIC_05367  CDS     COG1289 5627890 5628903 +       [S] Predicted membrane protein
2722
        MJLIPHIC_05368  CDS     COG1012 5628893 5630341 -       [C] NAD-dependent aldehyde dehydrogenases
2723
        MJLIPHIC_05369  CDS     COG0166 5630392 5632041 +       [G] Glucose-6-phosphate isomerase
2724
        MJLIPHIC_05370  CDS     ROG0102 5632038 5632613 -       NA
        MJLIPHIC_05371  CDS     COG1476 5632606 5632896 -       [K] Predicted transcriptional regulators
2725
        MJLIPHIC_05372  CDS     NA      5632985 5633395 -       NA
        MJLIPHIC_05373  CDS     COG0300 5633446 5634195 -       [R] Short-chain dehydrogenases of various substrate specificities
        MJLIPHIC_05374  CDS     COG0266 5634240 5635106 -       [L] Formamidopyrimidine-DNA glycosylase
2726
        MJLIPHIC_05375  CDS     COG1289 5635137 5636897 +       [S] Predicted membrane protein
2727
        MJLIPHIC_05376  CDS     COG1950 5636862 5637251 -       [S] Predicted membrane protein
        MJLIPHIC_05377  CDS     COG2243 5637258 5637998 -       [H] Precorrin-2 methylase
2728
        MJLIPHIC_05378  CDS     ROG0932 5638116 5639345 +       NA
2729
        MJLIPHIC_05379  CDS     COG2201 5639461 5639820 +       [NT] Chemotaxis response regulator containing a CheY-like
        MJLIPHIC_05380  CDS     COG2201 5639830 5640474 +       [NT] Chemotaxis response regulator containing a CheY-like
        MJLIPHIC_05381  CDS     ROG6775 5640458 5641219 +       NA
        MJLIPHIC_05382  CDS     COG1352 5641267 5643117 +       [NT] Methylase of chemotaxis methyl-accepting proteins
2730
        MJLIPHIC_05383  CDS     NA      5643114 5643251 -       NA
2731
        MJLIPHIC_05384  CDS     ROG3614 5643478 5644194 -       NA
2732
        MJLIPHIC_05385  CDS     COG2141 5644293 5644796 +       [C] Coenzyme F420-dependent N5,N10-methylene tetrahydromethanopterin reductase and
        MJLIPHIC_05386  CDS     NA      5644793 5645176 +       NA
2733
        MJLIPHIC_05387  CDS     COG2814 5645336 5646550 +       [G] Arabinose efflux permease
2734
        MJLIPHIC_05388  CDS     NA      5646557 5647120 -       NA
2735
        MJLIPHIC_05389  CDS     NA      5647334 5647645 +       NA
2736
        MJLIPHIC_05390  CDS     COG2814 5647818 5649233 +       [G] Arabinose efflux permease
        MJLIPHIC_05391  CDS     ROG6189 5649256 5649882 +       NA
```

	MJLIPHIC_05392	CDS	COG0328	5649928	5650446	+	[L] Ribonuclease HI
2737							
	MJLIPHIC_05393	CDS	COG3448	5650412	5650972	-	[T] CBS-domain-containing membrane protein
2738							
	MJLIPHIC_05394	CDS	COG4977	5651232	5652230	-	[K] Transcriptional regulator containing an amidase domain
2739							
	MJLIPHIC_05395	CDS	COG2249	5652298	5653074	+	[R] Putative NADPH-quinone reductase (modulator of drug
2740							
	MJLIPHIC_05396	CDS	COG5514	5653075	5653701	-	[S] Uncharacterized conserved protein
2741							
	MJLIPHIC_05397	CDS	COG1846	5653799	5654260	-	[K] Transcriptional regulators
	MJLIPHIC_05398	CDS	COG1309	5654425	5655027	-	[K] Transcriptional regulator
2742							
	MJLIPHIC_05399	CDS	COG4221	5655119	5655880	+	[R] Short-chain alcohol dehydrogenase of unknown specificity
	MJLIPHIC_05400	CDS	COG4221	5655917	5656684	+	[R] Short-chain alcohol dehydrogenase of unknown specificity
2743							
	MJLIPHIC_05401	CDS	COG1793	5656824	5659091	-	[L] ATP-dependent DNA ligase
	MJLIPHIC_05402	CDS	COG3839	5659133	5660218	-	[G] ABC-type sugar transport systems, ATPase components
	MJLIPHIC_05403	CDS	COG0395	5660234	5661109	-	[G] ABC-type sugar transport system, permease component
	MJLIPHIC_05404	CDS	COG1175	5661109	5662071	-	[G] ABC-type sugar transport systems, permease components
	MJLIPHIC_05405	CDS	COG1653	5662068	5663411	-	[G] ABC-type sugar transport system, periplasmic component
	MJLIPHIC_05406	CDS	COG2390	5663425	5664360	-	[K] Transcriptional regulator, contains sigma factor-related N-terminal
2744							
	MJLIPHIC_05407	CDS	COG0246	5664415	5665851	+	[G] Mannitol-1-phosphate/altronate dehydrogenases
	MJLIPHIC_05408	CDS	COG0524	5665848	5666768	+	[G] Sugar kinases, ribokinase family
2745							
	MJLIPHIC_05409	CDS	NA	5666805	5668100	-	NA
2746							
	MJLIPHIC_05410	CDS	COG1846	5668343	5668813	-	[K] Transcriptional regulators
2747							
	MJLIPHIC_05411	CDS	COG1273	5669000	5669956	+	[S] Uncharacterized conserved protein
2748							
	MJLIPHIC_05412	CDS	COG5522	5669968	5670690	-	[S] Predicted integral membrane protein
2749							
	MJLIPHIC_05413	CDS	NA	5670763	5671227	-	NA
	MJLIPHIC_05414	CDS	ROG2733	5671280	5673136	-	NA
2750							
	MJLIPHIC_05415	CDS	COG1028	5673257	5674048	+	[IQR] Dehydrogenases with different specificities (related to
	MJLIPHIC_05416	CDS	COG3804	5674061	5675137	+	[S] Uncharacterized conserved protein related to dihydrodipicolinate
	MJLIPHIC_05417	CDS	NA	5675160	5675426	+	NA
2751							
	MJLIPHIC_05418	CDS	NA	5675497	5675724	+	NA
2752							
	MJLIPHIC_05419	CDS	COG1914	5675819	5677042	+	[P] Mn2+ and Fe2+ transporters of the
	MJLIPHIC_05420	CDS	COG4195	5677043	5678101	+	[R] Phage-related replication protein
2753							
	MJLIPHIC_05421	CDS	COG2170	5678088	5679185	-	[S] Uncharacterized conserved protein
2754							
	MJLIPHIC_05422	CDS	COG2141	5679247	5680065	+	[C] Coenzyme F420-dependent N5,N10-methylene tetrahydromethanopterin reductase and
2755							
	MJLIPHIC_05423	CDS	COG0028	5680074	5682029	-	[EH] Thiamine pyrophosphate-requiring enzymes [acetolactate synthase, pyruvate
	MJLIPHIC_05424	CDS	COG0069	5682026	5683654	-	[E] Glutamate synthase domain 2
	MJLIPHIC_05425	CDS	COG1846	5683665	5684138	-	[K] Transcriptional regulators
2756							
	MJLIPHIC_05426	CDS	COG1028	5684407	5685162	+	[IQR] Dehydrogenases with different specificities (related to
	MJLIPHIC_05427	CDS	COG1309	5685250	5685846	+	[K] Transcriptional regulator
2757							
	MJLIPHIC_05428	tRNA	NA	5685927	5686000	+	NA
2758							
	MJLIPHIC_05429	CDS	NA	5686166	5686792	+	NA
	MJLIPHIC_05430	CDS	NA	5686847	5687998	+	NA
	MJLIPHIC_05431	CDS	NA	5687995	5688570	+	NA
2759							
	MJLIPHIC_05432	CDS	COG3339	5688973	5689389	+	[S] Uncharacterized conserved protein
	MJLIPHIC_05433	CDS	COG0640	5689444	5689782	+	[K] Predicted transcriptional regulators
	MJLIPHIC_05434	CDS	ROG2839	5689779	5690408	+	NA
	MJLIPHIC_05435	CDS	COG1271	5690437	5691825	+	[C] Cytochrome bd-type quinol oxidase, subunit 1
	MJLIPHIC_05436	CDS	COG1294	5691822	5692820	+	[C] Cytochrome bd-type quinol oxidase, subunit 2
2760							
	MJLIPHIC_05437	CDS	NA	5693137	5693385	+	NA
2761							
	MJLIPHIC_05438	CDS	COG0183	5693502	5694701	+	[I] Acetyl-CoA acetyltransferase
	MJLIPHIC_05439	CDS	COG3670	5694705	5696228	+	[Q] Lignostilbene-alpha,beta-dioxygenase and related enzymes
	MJLIPHIC_05440	CDS	COG1733	5696228	5697178	+	[K] Predicted transcriptional regulators
2762							
	MJLIPHIC_05441	CDS	COG0071	5697272	5697730	+	[O] Molecular chaperone (small heat shock protein)
2763							
	MJLIPHIC_05442	CDS	COG1670	5697737	5698342	-	[J] Acetyltransferases, including N-acetylases of ribosomal proteins
	MJLIPHIC_05443	CDS	NA	5698339	5698731	-	NA
2764							
	MJLIPHIC_05444	CDS	COG3542	5698778	5699224	+	[S] Uncharacterized conserved protein
2765							
	MJLIPHIC_05445	CDS	COG3246	5699214	5699942	-	[S] Uncharacterized conserved protein
	MJLIPHIC_05446	CDS	COG3324	5699957	5700727	-	[R] Predicted enzyme related to lactoylglutathione lyase
2766							
	MJLIPHIC_05447	CDS	ROG1691	5700826	5701302	-	NA
2767							
	MJLIPHIC_05448	CDS	COG1960	5701411	5702436	-	[I] Acyl-CoA dehydrogenases
	MJLIPHIC_05449	CDS	COG1960	5702436	5703611	-	[I] Acyl-CoA dehydrogenases
	MJLIPHIC_05450	CDS	COG1024	5703608	5704387	-	[I] Enoyl-CoA hydratase/carnithine racemase
	MJLIPHIC_05451	CDS	COG1960	5704384	5705304	-	[I] Acyl-CoA dehydrogenases
	MJLIPHIC_05452	CDS	COG1960	5705301	5706458	-	[I] Acyl-CoA dehydrogenases
	MJLIPHIC_05453	CDS	COG1804	5706470	5707621	-	[C] Predicted acyl-CoA transferases/carnitine dehydratase
	MJLIPHIC_05454	CDS	COG1028	5707618	5708538	-	[IQR] Dehydrogenases with different specificities (related to
	MJLIPHIC_05455	CDS	COG2072	5708555	5710393	-	[P] Predicted flavoprotein involved in K+ transport
	MJLIPHIC_05456	CDS	COG2141	5710390	5711562	-	[C] Coenzyme F420-dependent N5,N10-methylene tetrahydromethanopterin reductase and
	MJLIPHIC_05457	CDS	COG4638	5711559	5712947	-	[PR] Phenylpropionate dioxygenase and related ring-hydroxylating dioxygenases,
	MJLIPHIC_05458	CDS	COG1028	5712981	5713826	-	[IQR] Dehydrogenases with different specificities (related to
2768							
	MJLIPHIC_05459	CDS	ROG1158	5713850	5715571	+	NA

	MJLIPHIC_05460	CDS	COG2188	5715578	5716315	+	[K] Transcriptional regulators
	MJLIPHIC_05461	CDS	COG0318	5716317	5717849	+	[IQ] Acyl-CoA synthetases (AMP-forming)/AMP-acid ligases II
	MJLIPHIC_05462	CDS	COG1028	5717850	5718623	+	[IQR] Dehydrogenases with different specificities (related to
	MJLIPHIC_05463	CDS	COG0318	5718620	5720092	+	[IQ] Acyl-CoA synthetases (AMP-forming)/AMP-acid ligases II
2769							
	MJLIPHIC_05464	CDS	ROG4162	5720095	5720538	-	NA
	MJLIPHIC_05465	CDS	ROG5312	5720548	5720736	-	NA
	MJLIPHIC_05466	CDS	COG2217	5720762	5723128	-	[P] Cation transport ATPase
	MJLIPHIC_05467	CDS	COG1680	5723151	5724758	-	[V] Beta-lactamase class C and other penicillin
	MJLIPHIC_05468	CDS	COG2220	5724814	5725929	-	[R] Predicted Zn-dependent hydrolases of the beta-lactamase
	MJLIPHIC_05469	CDS	COG1024	5725931	5726662	-	[I] Enoyl-CoA hydratase/carnithine racemase
	MJLIPHIC_05470	CDS	COG3408	5726689	5728815	-	[G] Glycogen debranching enzyme
	MJLIPHIC_05471	CDS	COG0438	5728812	5729963	-	[M] Glycosyltransferase
2770							
	MJLIPHIC_05472	CDS	COG0777	5730128	5731561	+	[I] Acetyl-CoA carboxylase beta subunit
2771							
	MJLIPHIC_05473	CDS	ROG3094	5731669	5732505	+	NA
2772							
	MJLIPHIC_05474	CDS	COG1511	5732558	5734552	-	[S] Predicted membrane protein
	MJLIPHIC_05475	CDS	COG4988	5734567	5735295	-	[CO] ABC-type transport system involved in cytochrome
2773							
	MJLIPHIC_05476	CDS	NA	5735881	5736054	+	NA
2774							
	MJLIPHIC_05477	CDS	NA	5736231	5736935	+	NA
2775							
	MJLIPHIC_05478	CDS	ROG6247	5736936	5737325	-	NA
2776							
	MJLIPHIC_05479	CDS	COG0318	5737445	5739058	-	[IQ] Acyl-CoA synthetases (AMP-forming)/AMP-acid ligases II
	MJLIPHIC_05480	CDS	COG0318	5739055	5740743	-	[IQ] Acyl-CoA synthetases (AMP-forming)/AMP-acid ligases II
2777							
	MJLIPHIC_05481	CDS	COG2771	5740801	5741643	+	[K] DNA-binding HTH domain-containing proteins
2778							
	MJLIPHIC_05482	CDS	COG0596	5741657	5742694	-	[R] Predicted hydrolases or acyltransferases (alpha/beta hydrolase
2779							
	MJLIPHIC_05483	CDS	COG0583	5742793	5743662	+	[K] Transcriptional regulator
2780							
	MJLIPHIC_05484	CDS	COG1457	5743637	5745124	-	[F] Purine-cytosine permease and related proteins
	MJLIPHIC_05485	CDS	COG0388	5745137	5746042	-	[R] Predicted amidohydrolase
	MJLIPHIC_05486	CDS	COG0141	5745996	5747351	-	[E] Histidinol dehydrogenase
2781							
	MJLIPHIC_05487	CDS	COG0583	5747436	5748392	-	[K] Transcriptional regulator
2782							
	MJLIPHIC_05488	CDS	COG0596	5748411	5749523	+	[R] Predicted hydrolases or acyltransferases (alpha/beta hydrolase
2783							
	MJLIPHIC_05489	CDS	COG1132	5749605	5751437	-	[V] ABC-type multidrug transport system, ATPase and
	MJLIPHIC_05490	CDS	COG1132	5751434	5753380	-	[V] ABC-type multidrug transport system, ATPase and
	MJLIPHIC_05491	CDS	COG1132	5753419	5755335	-	[V] ABC-type multidrug transport system, ATPase and
2784							
	MJLIPHIC_05492	CDS	COG0745	5755493	5756161	+	[TK] Response regulators consisting of a CheY-like
	MJLIPHIC_05493	CDS	COG2205	5756171	5757511	+	[T] Osmosensitive K+ channel histidine kinase
2785							
	MJLIPHIC_05494	CDS	COG0545	5757592	5757954	-	[O] FKBP-type peptidyl-prolyl cis-trans isomerases 1
2786							
	MJLIPHIC_05495	CDS	ROG1734	5757986	5758234	+	NA
	MJLIPHIC_05496	CDS	COG1233	5758241	5759800	+	[Q] Phytoene dehydrogenase and related proteins
2787							
	MJLIPHIC_05497	CDS	COG3335	5760221	5760928	+	[L] Transposase and inactivated derivatives
2788							
	MJLIPHIC_05498	CDS	COG1846	5760925	5761374	-	[K] Transcriptional regulators
	MJLIPHIC_05499	CDS	ROG2472	5761364	5762821	-	NA
2789							
	MJLIPHIC_05500	CDS	COG2606	5762879	5763358	+	[S] Uncharacterized conserved protein
2790							
	MJLIPHIC_05501	CDS	COG0372	5763378	5764682	-	[C] Citrate synthase
2791							
	MJLIPHIC_05502	CDS	COG1309	5764801	5765442	-	[K] Transcriptional regulator
2792							
	MJLIPHIC_05503	CDS	NA	5765508	5766815	-	NA
	MJLIPHIC_05504	CDS	COG0259	5766819	5767454	-	[H] Pyridoxamine-phosphate oxidase
2793							
	MJLIPHIC_05505	CDS	COG0372	5767553	5768686	+	[C] Citrate synthase
	MJLIPHIC_05506	CDS	ROG6954	5768683	5769285	+	NA
2794							
	MJLIPHIC_05507	CDS	ROG5585	5769287	5769673	-	NA
	MJLIPHIC_05508	CDS	COG0640	5769670	5770056	-	[K] Predicted transcriptional regulators
2795							
	MJLIPHIC_05509	CDS	COG2764	5770134	5770589	+	[S] Uncharacterized protein conserved in bacteria
2796							
	MJLIPHIC_05510	CDS	COG1146	5770593	5772266	-	[C] Ferredoxin
	MJLIPHIC_05511	CDS	ROG0932	5772279	5773295	-	NA
2797							
	MJLIPHIC_05512	CDS	COG0024	5773416	5774180	+	[J] Methionine aminopeptidase
2798							
	MJLIPHIC_05513	CDS	COG1932	5774274	5775386	+	[HE] Phosphoserine aminotransferase
2799							
	MJLIPHIC_05514	CDS	ROG0214	5775478	5776308	+	NA
2800							
	MJLIPHIC_05515	CDS	ROG5373	5776322	5776597	-	NA
	MJLIPHIC_05516	CDS	COG0566	5776618	5777451	-	[J] rRNA methylases
	MJLIPHIC_05517	CDS	COG1846	5777448	5777879	-	[K] Transcriptional regulators
2801							
	MJLIPHIC_05518	CDS	NA	5778000	5778230	+	NA
	MJLIPHIC_05519	CDS	ROG3519	5778239	5778691	+	NA
2802							
	MJLIPHIC_05520	CDS	ROG0263	5778712	5779506	-	NA
2803							
	MJLIPHIC_05521	CDS	NA	5779647	5781245	+	NA
	MJLIPHIC_05522	CDS	ROG5751	5781230	5781730	+	NA
2804							
	MJLIPHIC_05523	CDS	COG0435	5781741	5782766	-	[O] Predicted glutathione S-transferase

	MJLIPHIC_05524	CDS	COG1278	5782812	5783261	-	[K] Cold shock proteins
2805							
	MJLIPHIC_05525	CDS	COG3304	5783385	5783795	+	[S] Predicted membrane protein
	MJLIPHIC_05526	CDS	COG2896	5783792	5784877	+	[H] Molybdenum cofactor biosynthesis enzyme
	MJLIPHIC_05527	CDS	ROG5635	5784878	5785144	+	NA
2806							
	MJLIPHIC_05528	CDS	ROG0976	5785578	5787038	+	NA
2807							
	MJLIPHIC_05529	CDS	COG0314	5787056	5787484	-	[H] Molybdopterin converting factor, large subunit
	MJLIPHIC_05530	CDS	COG0521	5787521	5788018	-	[H] Molybdopterin biosynthesis enzymes
	MJLIPHIC_05531	CDS	COG0315	5787997	5788470	-	[H] Molybdenum cofactor biosynthesis enzyme
	MJLIPHIC_05532	CDS	ROG2982	5788473	5788670	-	NA
2808							
	MJLIPHIC_05533	CDS	ROG0336	5788729	5790984	+	NA
2809							
	MJLIPHIC_05534	CDS	COG1061	5791133	5792782	+	[KL] DNA or RNA helicases of superfamily
2810							
	MJLIPHIC_05535	CDS	ROG2287	5792803	5793156	-	NA
2811							
	MJLIPHIC_05536	CDS	NA	5793234	5793884	-	NA
	MJLIPHIC_05537	CDS	NA	5793898	5794431	-	NA
2812							
	MJLIPHIC_05538	CDS	NA	5795059	5795433	+	NA
2813							
	MJLIPHIC_05539	CDS	COG0300	5795681	5796355	-	[R] Short-chain dehydrogenases of various substrate specificities
2814							
	MJLIPHIC_05540	CDS	NA	5796496	5797728	+	NA
2815							
	MJLIPHIC_05541	CDS	COG0583	5797558	5798490	-	[K] Transcriptional regulator
2816							
	MJLIPHIC_05542	CDS	COG1028	5798619	5799317	+	[IQR] Dehydrogenases with different specificities (related to
	MJLIPHIC_05543	CDS	ROG0232	5799304	5799669	+	NA
2817							
	MJLIPHIC_05544	CDS	COG2141	5799650	5800642	-	[C] Coenzyme F420-dependent N5,N10-methylene tetrahydromethanopterin reductase and
2818							
	MJLIPHIC_05545	CDS	ROG5840	5800752	5801177	+	NA
2819							
	MJLIPHIC_05546	CDS	COG1309	5801244	5801789	-	[K] Transcriptional regulator
2820							
	MJLIPHIC_05547	CDS	COG0654	5801864	5803072	+	[HC] 2-polyprenyl-6-methoxyphenol hydroxylase and related FAD-dependent oxidoreductases
2821							
	MJLIPHIC_05548	CDS	COG1250	5803163	5805325	-	[I] 3-hydroxyacyl-CoA dehydrogenase
	MJLIPHIC_05549	CDS	COG0183	5805343	5806554	-	[I] Acetyl-CoA acetyltransferase
2822							
	MJLIPHIC_05550	CDS	NA	5806701	5806889	-	NA
2823							
	MJLIPHIC_05551	CDS	COG0251	5807129	5807560	-	[J] Putative translation initiation inhibitor, yjgF family
2824							
	MJLIPHIC_05552	CDS	COG1846	5807649	5808116	+	[K] Transcriptional regulators
	MJLIPHIC_05553	CDS	COG0436	5808140	5809312	+	[E] Aspartate/tyrosine/aromatic aminotransferase
	MJLIPHIC_05554	CDS	ROG3217	5809320	5810558	+	NA
	MJLIPHIC_05555	CDS	COG4266	5810572	5811534	+	[F] Allantoicase
	MJLIPHIC_05556	CDS	NA	5811531	5812430	+	NA
2825							
	MJLIPHIC_05557	CDS	COG4126	5812505	5813212	-	[E] Hydantoin racemase
	MJLIPHIC_05558	CDS	COG1953	5813209	5814771	-	[FH] Cytosine/uracil/thiamine/allantoin permeases
	MJLIPHIC_05559	CDS	COG1802	5814846	5815541	-	[K] Transcriptional regulators
	MJLIPHIC_05560	CDS	COG2141	5815587	5816501	-	[C] Coenzyme F420-dependent N5,N10-methylene tetrahydromethanopterin reductase and
	MJLIPHIC_05561	CDS	COG0589	5816547	5817425	-	[T] Universal stress protein UspA and related
	MJLIPHIC_05562	CDS	COG1804	5817499	5818581	-	[C] Predicted acyl-CoA transferases/carnitine dehydratase
2826							
	MJLIPHIC_05563	CDS	COG3961	5818600	5820267	+	[GHR] Pyruvate decarboxylase and related thiamine pyrophosphate-requiring
2827							
	MJLIPHIC_05564	CDS	ROG1353	5820278	5820718	-	NA
2828							
	MJLIPHIC_05565	CDS	ROG0449	5820829	5821098	+	NA
2829							
	MJLIPHIC_05566	CDS	COG3320	5821317	5824838	+	[Q] Putative dehydrogenase domain of multifunctional non-ribosomal
2830							
	MJLIPHIC_05567	CDS	NA	5824860	5825129	-	NA
	MJLIPHIC_05568	CDS	COG1434	5825153	5825764	-	[S] Uncharacterized conserved protein
	MJLIPHIC_05569	CDS	NA	5825819	5826817	-	NA
	MJLIPHIC_05570	CDS	COG1752	5826814	5827605	-	[R] Predicted esterase of the alpha-beta hydrolase
	MJLIPHIC_05571	CDS	ROG5531	5827650	5827940	-	NA
	MJLIPHIC_05572	CDS	ROG1585	5827940	5828140	-	NA
	MJLIPHIC_05573	CDS	ROG5002	5828137	5828853	-	NA
	MJLIPHIC_05574	CDS	NA	5828870	5829322	-	NA
2831							
	MJLIPHIC_05575	CDS	NA	5829490	5830131	-	NA
2832							
	MJLIPHIC_05576	CDS	ROG3980	5830307	5830537	-	NA
	MJLIPHIC_05577	CDS	NA	5830537	5830830	-	NA
2833							
	MJLIPHIC_05578	CDS	COG4186	5831541	5832110	+	[R] Predicted phosphoesterase or phosphohydrolase
2834							
	MJLIPHIC_05579	CDS	ROG4729	5832107	5832586	-	NA
2835							
	MJLIPHIC_05580	CDS	COG0582	5832749	5833963	+	[L] Integrase
2836							
	MJLIPHIC_05581	tRNA	NA	5834093	5834167	-	NA
	MJLIPHIC_05582	tRNA	NA	5834209	5834285	-	NA
	MJLIPHIC_05583	tRNA	NA	5834321	5834394	-	NA
2837							
	MJLIPHIC_05584	tRNA	NA	5834573	5834646	+	NA
2838							
	MJLIPHIC_05585	CDS	NA	5834677	5835828	-	NA
2839							
	MJLIPHIC_05586	CDS	COG1167	5836135	5837526	+	[KE] Transcriptional regulators containing a DNA-binding HTH
2840							

	MJLIPHIC_05587	CDS	COG1917	5837604	5838014	+	[S] Uncharacterized conserved protein, contains double-stranded beta-helix
	MJLIPHIC_05588	CDS	COG0604	5838020	5838943	+	[CR] NADPH:quinone reductase and related Zn-dependent oxidoreductases
	MJLIPHIC_05589	CDS	COG0596	5838945	5839619	+	[R] Predicted hydrolases or acyltransferases (alpha/beta hydrolase
	MJLIPHIC_05590	CDS	ROG0115	5839631	5840230	+	NA
2841							
	MJLIPHIC_05591	CDS	COG2346	5840238	5840603	-	[R] Truncated hemoglobins
2842							
	MJLIPHIC_05592	CDS	COG3184	5840791	5841132	+	[S] Uncharacterized protein conserved in bacteria
2843							
	MJLIPHIC_05593	CDS	COG3662	5841139	5842122	-	[S] Uncharacterized protein conserved in bacteria
2844							
	MJLIPHIC_05594	CDS	COG0155	5842231	5842815	+	[P] Sulfite reductase, beta subunit (hemoprotein)
2845							
	MJLIPHIC_05595	CDS	COG4954	5842827	5843261	-	[S] Uncharacterized protein conserved in bacteria
	MJLIPHIC_05596	CDS	COG4954	5843430	5843864	-	[S] Uncharacterized protein conserved in bacteria
2846							
	MJLIPHIC_05597	CDS	COG3211	5843975	5844388	-	[R] Predicted phosphatase
2847							
	MJLIPHIC_05598	CDS	ROG1001	5844594	5845598	+	NA
	MJLIPHIC_05599	CDS	COG0042	5845595	5846764	+	[J] tRNA-dihydrouridine synthase
2848							
	MJLIPHIC_05600	CDS	COG1316	5846991	5849369	+	[K] Transcriptional regulator
	MJLIPHIC_05601	CDS	COG0704	5849446	5850114	+	[P] Phosphate uptake regulator
2849							
	MJLIPHIC_05602	CDS	COG0640	5850116	5850694	-	[K] Predicted transcriptional regulators
2850							
	MJLIPHIC_05603	CDS	COG0604	5850730	5851704	+	[CR] NADPH:quinone reductase and related Zn-dependent oxidoreductases
2851							
	MJLIPHIC_05604	CDS	COG1117	5851717	5852493	-	[P] ABC-type phosphate transport system, ATPase component
	MJLIPHIC_05605	CDS	COG0581	5852502	5853428	-	[P] ABC-type phosphate transport system, permease component
	MJLIPHIC_05606	CDS	COG0573	5853425	5854480	-	[P] ABC-type phosphate transport system, permease component
	MJLIPHIC_05607	CDS	COG0226	5854529	5855650	-	[P] ABC-type phosphate transport system, periplasmic component
2852							
	MJLIPHIC_05608	CDS	COG0454	5855793	5856710	-	[KR] Histone acetyltransferase HPA2 and related acetyltransferases
	MJLIPHIC_05609	CDS	COG0745	5856707	5857492	-	[TK] Response regulators consisting of a CheY-like
2853							
	MJLIPHIC_05610	CDS	ROG1093	5857658	5858473	+	NA
	MJLIPHIC_05611	CDS	COG0526	5858482	5858934	+	[OC] Thiol-disulfide isomerase and thioredoxins
2854							
	MJLIPHIC_05612	CDS	ROG2244	5859148	5859627	+	NA
	MJLIPHIC_05613	CDS	COG2897	5859664	5860497	+	[P] Rhodanese-related sulfurtransferase
	MJLIPHIC_05614	CDS	ROG0959	5860499	5860801	+	NA
2855							
	MJLIPHIC_05615	CDS	NA	5860943	5861041	+	NA
	MJLIPHIC_05616	CDS	ROG0284	5861038	5861694	+	NA
	MJLIPHIC_05617	CDS	ROG4412	5861703	5861984	+	NA
2856							
	MJLIPHIC_05618	CDS	COG0115	5862693	5863580	-	[EH] Branched-chain amino acid aminotransferase/4-amino-4-deoxychorismate lyase
2857							
	MJLIPHIC_05619	CDS	COG0354	5863663	5864742	+	[R] Predicted aminomethyltransferase related to GcvT
2858							
	MJLIPHIC_05620	CDS	ROG8289	5865007	5865180	+	NA
2859							
	MJLIPHIC_05621	CDS	COG0150	5865211	5866293	-	[F] Phosphoribosylaminoimidazole (AIR) synthetase
2860							
	MJLIPHIC_05622	CDS	COG0702	5866483	5867736	+	[MG] Predicted nucleoside-diphosphate-sugar epimerases
2861							
	MJLIPHIC_05623	CDS	COG0034	5867744	5869279	-	[F] Glutamine phosphoribosylpyrophosphate amidotransferase
2862							
	MJLIPHIC_05624	CDS	ROG0288	5869361	5870734	-	NA
2863							
	MJLIPHIC_05625	CDS	COG1028	5870893	5871624	+	[IQR] Dehydrogenases with different specificities (related to
2864							
	MJLIPHIC_05626	CDS	NA	5871708	5872067	+	NA
	MJLIPHIC_05627	CDS	NA	5872064	5872759	+	NA
2865							
	MJLIPHIC_05628	CDS	COG1135	5873134	5874081	+	[P] ABC-type metal ion transport system, ATPase
	MJLIPHIC_05629	CDS	COG2011	5874133	5874816	+	[P] ABC-type metal ion transport system, permease
	MJLIPHIC_05630	CDS	COG1464	5874832	5875731	+	[P] ABC-type metal ion transport system, periplasmic
	MJLIPHIC_05631	CDS	COG2141	5875728	5877137	+	[C] Coenzyme F420-dependent N5,N10-methylene tetrahydromethanopterin reductase and
2866							
	MJLIPHIC_05632	CDS	COG1309	5877168	5877815	-	[K] Transcriptional regulator
2867							
	MJLIPHIC_05633	CDS	COG1028	5877928	5878758	+	[IQR] Dehydrogenases with different specificities (related to
	MJLIPHIC_05634	CDS	COG1028	5878755	5879558	+	[IQR] Dehydrogenases with different specificities (related to
	MJLIPHIC_05635	CDS	COG0599	5879581	5880393	+	[S] Uncharacterized homolog of gamma-carboxymuconolactone decarboxylase subunit
	MJLIPHIC_05636	CDS	COG1012	5880390	5881853	+	[C] NAD-dependent aldehyde dehydrogenases
2868							
	MJLIPHIC_05637	CDS	ROG6081	5882145	5883410	+	NA
2869							
	MJLIPHIC_05638	CDS	ROG0203	5883420	5883806	-	NA
2870							
	MJLIPHIC_05639	CDS	COG1463	5883823	5885037	+	[Q] ABC-type transport system involved in resistance
	MJLIPHIC_05640	CDS	ROG0988	5885077	5885499	+	NA
	MJLIPHIC_05641	CDS	NA	5885520	5885951	+	NA
	MJLIPHIC_05642	CDS	NA	5885944	5887236	+	NA
2871							
	MJLIPHIC_05643	CDS	COG1266	5887175	5887831	-	[R] Predicted metal-dependent membrane protease
	MJLIPHIC_05644	CDS	COG4425	5887831	5889561	-	[S] Predicted membrane protein
	MJLIPHIC_05645	CDS	COG0046	5889558	5891855	-	[F] Phosphoribosylformylglycinamidine (FGAM) synthase, synthetase domain
	MJLIPHIC_05646	CDS	ROG1113	5891852	5893060	-	NA
2872							
	MJLIPHIC_05647	CDS	COG0028	5893122	5894912	+	[EH] Thiamine pyrophosphate-requiring enzymes [acetolactate synthase, pyruvate
2873							
	MJLIPHIC_05648	CDS	ROG0697	5894923	5895273	-	NA
	MJLIPHIC_05649	CDS	COG1362	5895289	5896554	-	[E] Aspartyl aminopeptidase
2874							
	MJLIPHIC_05650	CDS	COG2837	5896604	5897635	+	[P] Predicted iron-dependent peroxidase
	MJLIPHIC_05651	CDS	COG1659	5897635	5898432	+	[S] Uncharacterized protein, linocin/CFP29 homolog
2875							

MJLIPHIC_05652 CDS COG0047 5898597 5899271 - [F] Phosphoribosylformylglycinamidine (FGAM) synthase, glutamine amidotransferase domain
MJLIPHIC_05653 CDS COG1828 5899268 5899507 - [F] Phosphoribosylformylglycinamidine (FGAM) synthase, PurS component
MJLIPHIC_05654 CDS NA 5899518 5899883 - NA
2876
MJLIPHIC_05655 CDS COG2220 5900049 5900858 + [R] Predicted Zn-dependent hydrolases of the beta-lactamase
2877
MJLIPHIC_05656 CDS COG3573 5900871 5902514 - [R] Predicted oxidoreductase
MJLIPHIC_05657 CDS COG3233 5902536 5903252 - [R] Predicted deacetylase
2878
MJLIPHIC_05658 CDS COG0386 5903317 5903802 - [O] Glutathione peroxidase
MJLIPHIC_05659 CDS COG1309 5903822 5904367 - [K] Transcriptional regulator
2879
MJLIPHIC_05660 CDS COG4270 5904456 5904905 + [S] Predicted membrane protein
2880
MJLIPHIC_05661 CDS COG1770 5904831 5906987 - [E] Protease II
MJLIPHIC_05662 CDS COG0152 5906984 5907883 - [F] Phosphoribosylaminoimidazolesuccinocarboxamide (SAICAR) synthase
2881
MJLIPHIC_05663 CDS ROG2724 5907914 5908537 + NA
2882
MJLIPHIC_05664 CDS ROG3094 5908629 5909528 + NA
2883
MJLIPHIC_05665 CDS ROG0549 5909473 5910063 - NA
MJLIPHIC_05666 CDS NA 5910084 5910959 - NA
MJLIPHIC_05667 CDS COG2124 5910956 5912161 - [Q] Cytochrome P450
MJLIPHIC_05668 CDS COG0015 5912178 5913602 - [F] Adenylosuccinate lyase
2884
MJLIPHIC_05669 CDS COG4328 5913636 5915339 + [S] Uncharacterized protein conserved in bacteria
2885
MJLIPHIC_05670 CDS COG0531 5915336 5916760 - [E] Amino acid transporters
MJLIPHIC_05671 CDS COG3226 5916780 5917409 - [S] Uncharacterized protein conserved in bacteria
2886
MJLIPHIC_05672 CDS COG0627 5917517 5918410 + [R] Predicted esterase
2887
MJLIPHIC_05673 CDS COG0151 5918416 5919597 - [F] Phosphoribosylamine-glycine ligase
2888
MJLIPHIC_05674 CDS COG2124 5919792 5921018 + [Q] Cytochrome P450
MJLIPHIC_05675 CDS COG1309 5921046 5921669 + [K] Transcriptional regulator
2889
MJLIPHIC_05676 CDS ROG5992 5921730 5922956 - NA
MJLIPHIC_05677 CDS COG0599 5922946 5923401 - [S] Uncharacterized homolog of gamma-carboxymuconolactone decarboxylase subunit
MJLIPHIC_05678 CDS COG2084 5923404 5924258 - [I] 3-hydroxyisobutyrate dehydrogenase and related beta-hydroxyacid dehydrogenases
MJLIPHIC_05679 CDS COG1028 5924255 5925019 - [IQR] Dehydrogenases with different specificities (related to
MJLIPHIC_05680 CDS COG1012 5925031 5926500 - [C] NAD-dependent aldehyde dehydrogenases
2890
MJLIPHIC_05681 CDS COG1309 5926701 5927285 + [K] Transcriptional regulator
MJLIPHIC_05682 CDS COG2124 5927282 5928484 + [Q] Cytochrome P450
MJLIPHIC_05683 CDS COG4221 5928485 5929312 + [R] Short-chain alcohol dehydrogenase of unknown specificity
MJLIPHIC_05684 CDS COG2124 5929309 5930664 + [Q] Cytochrome P450
MJLIPHIC_05685 CDS COG1141 5930669 5930872 + [C] Ferredoxin
MJLIPHIC_05686 CDS ROG2779 5930884 5931423 + NA
2891
MJLIPHIC_05687 CDS COG1062 5931523 5932650 + [C] Zn-dependent alcohol dehydrogenases, class III
MJLIPHIC_05688 CDS ROG5736 5932661 5933098 + NA
MJLIPHIC_05689 CDS COG1028 5933124 5933951 + [IQR] Dehydrogenases with different specificities (related to
2892
MJLIPHIC_05690 CDS NA 5933938 5934270 - NA
2893
MJLIPHIC_05691 CDS COG0537 5934342 5934770 + [FGR] Diadenosine tetraphosphate (Ap4A) hydrolase and other
2894
MJLIPHIC_05692 CDS COG0642 5934730 5936157 - [T] Signal transduction histidine kinase
MJLIPHIC_05693 CDS COG0745 5936199 5936918 - [TK] Response regulators consisting of a CheY-like
2895
MJLIPHIC_05694 CDS NA 5937058 5937876 + NA
MJLIPHIC_05695 tRNA NA 5937904 5937978 + NA
2896
MJLIPHIC_05696 CDS ROG3286 5938050 5938424 + NA
2897
MJLIPHIC_05697 CDS NA 5938421 5938576 - NA
MJLIPHIC_05698 CDS ROG2976 5938617 5939756 - NA
2898
MJLIPHIC_05699 CDS ROG5614 5939853 5940551 - NA
2899
MJLIPHIC_05700 CDS COG2173 5940642 5941298 - [M] D-alanyl-D-alanine dipeptidase
2900
MJLIPHIC_05701 CDS COG1529 5941399 5943753 + [C] Aerobic-type carbon monoxide dehydrogenase, large subunit
MJLIPHIC_05702 CDS COG3427 5943756 5944466 + [S] Uncharacterized conserved protein
MJLIPHIC_05703 CDS COG1319 5944463 5945350 + [C] Aerobic-type carbon monoxide dehydrogenase, middle subunit
MJLIPHIC_05704 CDS COG2080 5945343 5945855 + [C] Aerobic-type carbon monoxide dehydrogenase, small subunit
MJLIPHIC_05705 CDS COG2084 5945888 5946787 + [I] 3-hydroxyisobutyrate dehydrogenase and related beta-hydroxyacid dehydrogenases
2901
MJLIPHIC_05706 CDS COG1028 5946791 5947642 - [IQR] Dehydrogenases with different specificities (related to
MJLIPHIC_05707 CDS COG0447 5947663 5948580 - [H] Dihydroxynaphthoic acid synthase
2902
MJLIPHIC_05708 CDS NA 5948845 5949216 + NA
2903
MJLIPHIC_05709 CDS NA 5949354 5949644 + NA
2904
MJLIPHIC_05710 CDS NA 5949761 5950150 + NA
MJLIPHIC_05711 CDS NA 5950199 5950363 + NA
MJLIPHIC_05712 CDS COG0380 5950364 5951875 + [G] Trehalose-6-phosphate synthase
2905
MJLIPHIC_05713 CDS COG3335 5951872 5952933 - [L] Transposase and inactivated derivatives
MJLIPHIC_05714 CDS ROG3802 5952977 5953462 - NA
MJLIPHIC_05715 CDS ROG5477 5953462 5954163 - NA
MJLIPHIC_05716 CDS COG1463 5954163 5955857 - [Q] ABC-type transport system involved in resistance
MJLIPHIC_05717 CDS COG1463 5955858 5957069 - [Q] ABC-type transport system involved in resistance
MJLIPHIC_05718 CDS COG1463 5957066 5958430 - [Q] ABC-type transport system involved in resistance
MJLIPHIC_05719 CDS COG1463 5958427 5959485 - [Q] ABC-type transport system involved in resistance
MJLIPHIC_05720 CDS COG1463 5959478 5960512 - [Q] ABC-type transport system involved in resistance

	MJLIPHIC_05721	CDS	COG1463	5960513	5961715	-	[Q] ABC-type transport system involved in resistance
	MJLIPHIC_05722	CDS	COG0767	5961722	5962564	-	[Q] ABC-type transport system involved in resistance
	MJLIPHIC_05723	CDS	COG0767	5962620	5963384	-	[Q] ABC-type transport system involved in resistance
2906							
	MJLIPHIC_05724	CDS	COG1028	5963654	5964604	-	[IQR] Dehydrogenases with different specificities (related to
	MJLIPHIC_05725	CDS	COG1141	5964647	5964838	-	[C] Ferredoxin
2907							
	MJLIPHIC_05726	CDS	COG1960	5965003	5966181	+	[I] Acyl-CoA dehydrogenases
	MJLIPHIC_05727	CDS	COG1960	5966201	5967298	+	[I] Acyl-CoA dehydrogenases
	MJLIPHIC_05728	CDS	COG0318	5967301	5968845	+	[IQ] Acyl-CoA synthetases (AMP-forming)/AMP-acid ligases II
	MJLIPHIC_05729	CDS	COG0702	5968857	5969831	+	[MG] Predicted nucleoside-diphosphate-sugar epimerases
2908							
	MJLIPHIC_05730	CDS	ROG4388	5969960	5970586	+	NA
2909							
	MJLIPHIC_05731	CDS	COG2764	5970523	5970918	-	[S] Uncharacterized protein conserved in bacteria
2910							
	MJLIPHIC_05732	CDS	COG1012	5971046	5972599	+	[C] NAD-dependent aldehyde dehydrogenases
2911							
	MJLIPHIC_05733	CDS	COG2070	5972603	5973724	-	[R] Dioxygenases related to 2-nitropropane dioxygenase
	MJLIPHIC_05734	CDS	COG0318	5973735	5975390	-	[IQ] Acyl-CoA synthetases (AMP-forming)/AMP-acid ligases II
2912							
	MJLIPHIC_05735	CDS	COG1024	5975459	5976259	+	[I] Enoyl-CoA hydratase/carnithine racemase
2913							
	MJLIPHIC_05736	CDS	ROG3094	5976871	5977719	+	NA
2914							
	MJLIPHIC_05737	CDS	COG2124	5977791	5978996	-	[Q] Cytochrome P450
	MJLIPHIC_05738	CDS	ROG3481	5978993	5979709	-	NA
	MJLIPHIC_05739	CDS	NA	5979753	5980784	-	NA
2915							
	MJLIPHIC_05740	CDS	COG1545	5980944	5981846	+	[R] Predicted nucleic-acid-binding protein containing a Zn-ribbon
	MJLIPHIC_05741	CDS	COG0183	5981856	5982929	+	[I] Acetyl-CoA acetyltransferase
	MJLIPHIC_05742	CDS	COG1214	5982929	5984107	+	[O] Inactive homolog of metal-dependent proteases, putative
2916							
	MJLIPHIC_05743	CDS	NA	5984266	5984898	+	NA
2917							
	MJLIPHIC_05744	CDS	COG4638	5985003	5986154	+	[PR] Phenylpropionate dioxygenase and related ring-hydroxylating dioxygenases,
	MJLIPHIC_05745	CDS	ROG3284	5986154	5986630	+	NA
2918							
	MJLIPHIC_05746	CDS	COG5337	5986627	5988120	-	[M] Spore coat assembly protein
	MJLIPHIC_05747	CDS	ROG0533	5988156	5988833	-	NA
	MJLIPHIC_05748	CDS	ROG0694	5988883	5989782	-	NA
2919							
	MJLIPHIC_05749	CDS	ROG0225	5989939	5991081	-	NA
	MJLIPHIC_05750	CDS	COG1028	5991078	5991860	-	[IQR] Dehydrogenases with different specificities (related to
	MJLIPHIC_05751	CDS	ROG1995	5991863	5992990	-	NA
2920							
	MJLIPHIC_05752	CDS	COG1414	5993104	5994012	+	[K] Transcriptional regulator
2921							
	MJLIPHIC_05753	CDS	NA	5994025	5994372	-	NA
2922							
	MJLIPHIC_05754	CDS	COG0514	5994502	5996577	-	[L] Superfamily II DNA helicase
2923							
	MJLIPHIC_05755	CDS	NA	5996684	5997067	-	NA
2924							
	MJLIPHIC_05756	CDS	COG0119	5997153	5998202	-	[E] Isopropylmalate/homocitrate/citramalate synthases
	MJLIPHIC_05757	CDS	COG4569	5998199	5999122	-	[Q] Acetaldehyde dehydrogenase (acetylating)
	MJLIPHIC_05758	CDS	COG3971	5999161	5999946	-	[Q] 2-keto-4-pentenoate hydratase
2925							
	MJLIPHIC_05759	CDS	COG1053	6000044	6001720	+	[C] Succinate dehydrogenase/fumarate reductase, flavoprotein subunit
	MJLIPHIC_05760	CDS	ROG0252	6001722	6002582	+	NA
2926							
	MJLIPHIC_05761	CDS	COG0318	6002532	6005153	-	[IQ] Acyl-CoA synthetases (AMP-forming)/AMP-acid ligases II
2927							
	MJLIPHIC_05762	CDS	COG2303	6005369	6006970	+	[E] Choline dehydrogenase and related flavoproteins
	MJLIPHIC_05763	CDS	COG1434	6007005	6007583	+	[S] Uncharacterized conserved protein
	MJLIPHIC_05764	CDS	COG1045	6007618	6008229	+	[E] Serine acetyltransferase
2928							
	MJLIPHIC_05765	CDS	NA	6008195	6009514	-	NA
	MJLIPHIC_05766	CDS	COG0463	6009511	6010416	-	[M] Glycosyltransferases involved in cell wall biogenesis
	MJLIPHIC_05767	CDS	NA	6010413	6011615	-	NA
2929							
	MJLIPHIC_05768	CDS	COG0110	6011799	6012347	+	[R] Acetyltransferase (isoleucine patch superfamily)
	MJLIPHIC_05769	CDS	COG0438	6012352	6013476	+	[M] Glycosyltransferase
	MJLIPHIC_05770	CDS	NA	6013473	6014132	+	NA
	MJLIPHIC_05771	CDS	ROG0972	6014129	6014881	+	NA
	MJLIPHIC_05772	CDS	COG3944	6014878	6015528	+	[M] Capsular polysaccharide biosynthesis protein
	MJLIPHIC_05773	CDS	COG3944	6015525	6016190	+	[M] Capsular polysaccharide biosynthesis protein
2930							
	MJLIPHIC_05774	CDS	COG0451	6016202	6017053	-	[MG] Nucleoside-diphosphate-sugar epimerases
	MJLIPHIC_05775	CDS	COG1004	6017397	6018602	-	[M] Predicted UDP-glucose 6-dehydrogenase
2931							
	MJLIPHIC_05776	CDS	NA	6019278	6020018	+	NA
	MJLIPHIC_05777	CDS	COG3307	6020020	6021429	+	[M] Lipid A core - O-antigen ligase
2932							
	MJLIPHIC_05778	CDS	COG5653	6021392	6022501	-	[M] Protein involved in cellulose biosynthesis (CelD)
	MJLIPHIC_05779	CDS	COG1216	6022498	6023439	-	[R] Predicted glycosyltransferases
	MJLIPHIC_05780	CDS	COG0726	6023439	6024206	-	[G] Predicted xylanase/chitin deacetylase
	MJLIPHIC_05781	CDS	ROG0655	6024203	6026239	-	NA
2933							
	MJLIPHIC_05782	CDS	COG0438	6026548	6027144	-	[M] Glycosyltransferase
2934							
	MJLIPHIC_05783	CDS	NA	6027361	6028110	+	NA
2935							
	MJLIPHIC_05784	CDS	NA	6028304	6029836	+	NA
2936							
	MJLIPHIC_05785	CDS	COG1028	6029863	6030690	-	[IQR] Dehydrogenases with different specificities (related to
	MJLIPHIC_05786	CDS	COG0644	6030680	6032278	-	[C] Dehydrogenases (flavoproteins)
2937							
	MJLIPHIC_05787	CDS	COG2244	6032735	6034267	-	[R] Membrane protein involved in the export
2938							

MJLIPHIC_05788 CDS ROG5646 6034371 6035561 - NA
2939
MJLIPHIC_05789 CDS COG0001 6035857 6037233 + [H] Glutamate-1-semialdehyde aminotransferase
2940
MJLIPHIC_05790 CDS ROG6974 6037322 6037984 + NA
2941
MJLIPHIC_05791 CDS NA 6038034 6038726 - NA
2942
MJLIPHIC_05792 CDS NA 6038751 6038999 + NA
2943
MJLIPHIC_05793 CDS NA 6039559 6040632 - NA
MJLIPHIC_05794 CDS NA 6040629 6041009 - NA
2944
MJLIPHIC_05795 CDS COG0451 6041185 6042213 - [MG] Nucleoside-diphosphate-sugar epimerases
MJLIPHIC_05796 CDS COG2120 6042243 6042911 - [S] Uncharacterized proteins, LmbE homologs
MJLIPHIC_05797 CDS COG1208 6042908 6043708 - [MJ] Nucleoside-diphosphate-sugar pyrophosphorylase involved in lipopolysaccharide biosynthesis/translation
MJLIPHIC_05798 CDS COG0500 6043705 6044961 - [QR] SAM-dependent methyltransferases
MJLIPHIC_05799 CDS COG5017 6044958 6046010 - [S] Uncharacterized conserved protein
MJLIPHIC_05800 CDS COG1004 6046007 6047368 - [M] Predicted UDP-glucose 6-dehydrogenase
MJLIPHIC_05801 CDS COG1209 6047365 6048243 - [M] dTDP-glucose pyrophosphorylase
MJLIPHIC_05802 CDS COG2148 6048253 6049809 - [M] Sugar transferases involved in lipopolysaccharide synthesis
MJLIPHIC_05803 CDS COG0451 6049806 6050765 - [MG] Nucleoside-diphosphate-sugar epimerases
MJLIPHIC_05804 CDS COG0449 6050802 6052589 - [M] Glucosamine 6-phosphate synthetase, contains amidotransferase and
2945
MJLIPHIC_05805 CDS COG2197 6052909 6053781 - [TK] Response regulator containing a CheY-like receiver
2946
MJLIPHIC_05806 CDS NA 6054384 6055199 - NA
MJLIPHIC_05807 CDS NA 6055221 6056327 - NA
2947
MJLIPHIC_05808 CDS NA 6056400 6057563 - NA
MJLIPHIC_05809 CDS ROG2306 6057560 6057958 - NA
MJLIPHIC_05810 CDS COG1545 6057955 6058920 - [R] Predicted nucleic-acid-binding protein containing a Zn-ribbon
MJLIPHIC_05811 CDS COG1960 6058917 6060080 - [I] Acyl-CoA dehydrogenases
MJLIPHIC_05812 CDS COG1960 6060080 6061084 - [I] Acyl-CoA dehydrogenases
MJLIPHIC_05813 CDS COG2124 6061103 6062386 - [Q] Cytochrome P450
2948
MJLIPHIC_05814 CDS COG0183 6062510 6063673 + [I] Acetyl-CoA acetyltransferase
MJLIPHIC_05815 CDS NA 6063676 6064056 + NA
MJLIPHIC_05816 CDS ROG1229 6064064 6064543 + NA
2949
MJLIPHIC_05817 CDS COG1028 6064621 6065526 - [IQR] Dehydrogenases with different specificities (related to
MJLIPHIC_05818 CDS COG1028 6065542 6066315 - [IQR] Dehydrogenases with different specificities (related to
2950
MJLIPHIC_05819 CDS COG1024 6066386 6067141 + [I] Enoyl-CoA hydratase/carnithine racemase
MJLIPHIC_05820 CDS COG1788 6067138 6068025 + [I] Acyl CoA:acetate/3-ketoacid CoA transferase, alpha subunit
MJLIPHIC_05821 CDS COG2057 6068025 6068771 + [I] Acyl CoA:acetate/3-ketoacid CoA transferase, beta subunit
MJLIPHIC_05822 CDS COG2070 6068780 6069844 + [R] Dioxygenases related to 2-nitropropane dioxygenase
MJLIPHIC_05823 CDS ROG4316 6069888 6070526 + NA
MJLIPHIC_05824 CDS ROG1498 6070523 6071269 + NA
2951
MJLIPHIC_05825 CDS NA 6071277 6071762 - NA
2952
MJLIPHIC_05826 CDS COG0053 6071829 6072254 - [P] Predicted Co/Zn/Cd cation transporters
2953
MJLIPHIC_05827 CDS COG0183 6072343 6073491 - [I] Acetyl-CoA acetyltransferase
MJLIPHIC_05828 CDS COG1309 6073484 6074101 - [K] Transcriptional regulator
2954
MJLIPHIC_05829 CDS COG1289 6074329 6074934 + [S] Predicted membrane protein
2955
MJLIPHIC_05830 CDS COG1028 6075060 6075848 - [IQR] Dehydrogenases with different specificities (related to
MJLIPHIC_05831 CDS COG1960 6075845 6077023 - [I] Acyl-CoA dehydrogenases
2956
MJLIPHIC_05832 CDS COG0318 6077072 6078415 + [IQ] Acyl-CoA synthetases (AMP-forming)/AMP-acid ligases II
MJLIPHIC_05833 CDS COG0318 6078412 6078624 + [IQ] Acyl-CoA synthetases (AMP-forming)/AMP-acid ligases II
MJLIPHIC_05834 CDS COG1960 6078624 6079766 + [I] Acyl-CoA dehydrogenases
MJLIPHIC_05835 CDS COG1960 6079763 6080719 + [I] Acyl-CoA dehydrogenases
MJLIPHIC_05836 CDS COG1960 6080716 6081651 + [I] Acyl-CoA dehydrogenases
MJLIPHIC_05837 CDS COG0436 6081678 6082838 + [E] Aspartate/tyrosine/aromatic aminotransferase
2957
MJLIPHIC_05838 CDS COG4214 6082847 6084166 - [G] ABC-type xylose transport system, permease component
MJLIPHIC_05839 CDS COG1129 6084175 6084954 - [G] ABC-type sugar transport system, ATPase component
MJLIPHIC_05840 CDS COG4213 6084958 6086037 - [G] ABC-type xylose transport system, periplasmic component
MJLIPHIC_05841 CDS COG2115 6086090 6087313 - [G] Xylose isomerase
2958
MJLIPHIC_05842 CDS COG1940 6087420 6088601 + [KG] Transcriptional regulator/sugar kinase
2959
MJLIPHIC_05843 CDS COG3547 6088633 6089832 - [L] Transposase and inactivated derivatives
2960
MJLIPHIC_05844 CDS ROG2733 6090126 6091412 + NA
2961
MJLIPHIC_05845 CDS COG0365 6091393 6093327 - [I] Acyl-coenzyme A synthetases/AMP-(fatty) acid ligases
MJLIPHIC_05846 CDS COG1028 6093324 6094070 - [IQR] Dehydrogenases with different specificities (related to
MJLIPHIC_05847 CDS COG0477 6094067 6095395 - [GEPR] Permeases of the major facilitator superfamily
2962
MJLIPHIC_05848 CDS COG0583 6095508 6096458 + [K] Transcriptional regulator
MJLIPHIC_05849 CDS COG2267 6096471 6097322 + [I] Lysophospholipase
2963
MJLIPHIC_05850 CDS COG2124 6097336 6098625 - [Q] Cytochrome P450
2964
MJLIPHIC_05851 CDS COG1309 6098766 6099374 + [K] Transcriptional regulator
2965
MJLIPHIC_05852 CDS COG1028 6099367 6100206 - [IQR] Dehydrogenases with different specificities (related to
2966
MJLIPHIC_05853 CDS NA 6100369 6101811 - NA
2967
MJLIPHIC_05854 CDS COG1853 6102080 6102649 - [R] Conserved protein/domain typically associated with flavoprotein
MJLIPHIC_05855 CDS COG0346 6102657 6103556 - [E] Lactoylglutathione lyase and related lyases
MJLIPHIC_05856 CDS COG0596 6103557 6104435 - [R] Predicted hydrolases or acyltransferases (alpha/beta hydrolase
MJLIPHIC_05857 CDS COG1960 6104435 6105625 - [I] Acyl-CoA dehydrogenases

2968							
	MJLIPHIC_05858	CDS	COG1018	6105785	6106846	+	[C] Flavodoxin reductases (ferredoxin-NADPH reductases) family 1
	MJLIPHIC_05859	CDS	NA	6106889	6107464	+	NA
2969							
	MJLIPHIC_05860	CDS	COG1960	6107545	6109689	-	[I] Acyl-CoA dehydrogenases
2970							
	MJLIPHIC_05861	CDS	COG1309	6109886	6110566	+	[K] Transcriptional regulator
	MJLIPHIC_05862	CDS	COG1877	6110570	6111319	+	[G] Trehalose-6-phosphatase
2971							
	MJLIPHIC_05863	CDS	COG1609	6111320	6112420	-	[K] Transcriptional regulators
	MJLIPHIC_05864	CDS	COG1108	6112469	6113341	-	[P] ABC-type Mn2+/Zn2+ transport systems, permease components
	MJLIPHIC_05865	CDS	COG1121	6113341	6114120	-	[P] ABC-type Mn/Zn transport systems, ATPase component
	MJLIPHIC_05866	CDS	COG0803	6114107	6115114	-	[P] ABC-type metal ion transport system, periplasmic
2972							
	MJLIPHIC_05867	CDS	COG0523	6115172	6116308	+	[R] Putative GTPases (G3E family)
2973							
	MJLIPHIC_05868	CDS	ROG0380	6116358	6117524	-	NA
	MJLIPHIC_05869	CDS	COG0803	6117544	6118446	-	[P] ABC-type metal ion transport system, periplasmic
	MJLIPHIC_05870	CDS	ROG7297	6118443	6119627	-	NA
	MJLIPHIC_05871	CDS	COG1108	6119624	6120448	-	[P] ABC-type Mn2+/Zn2+ transport systems, permease components
2974							
	MJLIPHIC_05872	CDS	COG1121	6120497	6121207	+	[P] ABC-type Mn/Zn transport systems, ATPase component
	MJLIPHIC_05873	CDS	COG0778	6121209	6121799	+	[C] Nitroreductase
	MJLIPHIC_05874	CDS	COG1540	6121823	6122608	+	[R] Uncharacterized proteins, homologs of lactam utilization
2975							
	MJLIPHIC_05875	CDS	NA	6122623	6123009	-	NA
2976							
	MJLIPHIC_05876	CDS	COG3547	6123276	6124349	-	[L] Transposase and inactivated derivatives
2977							
	MJLIPHIC_05877	CDS	NA	6124760	6125368	+	NA
2978							
	MJLIPHIC_05878	CDS	COG2217	6125473	6127572	-	[P] Cation transport ATPase
	MJLIPHIC_05879	CDS	NA	6127628	6127912	-	NA
2979							
	MJLIPHIC_05880	CDS	COG0701	6128041	6129036	+	[R] Predicted permeases
	MJLIPHIC_05881	CDS	COG3689	6129033	6129749	+	[S] Predicted membrane protein
2980							
	MJLIPHIC_05882	CDS	COG1120	6129763	6130554	-	[PH] ABC-type cobalamin/Fe3+-siderophores transport systems, ATPase components
	MJLIPHIC_05883	CDS	COG0609	6130551	6131585	-	[P] ABC-type Fe3+-siderophore transport system, permease component
	MJLIPHIC_05884	CDS	COG0614	6131586	6132566	-	[P] ABC-type Fe3+-hydroxamate transport system, periplasmic component
2981							
	MJLIPHIC_05885	CDS	COG0238	6132746	6133000	-	[J] Ribosomal protein S18
	MJLIPHIC_05886	CDS	COG0199	6133000	6133305	-	[J] Ribosomal protein S14
	MJLIPHIC_05887	CDS	COG0267	6133305	6133469	-	[J] Ribosomal protein L33
	MJLIPHIC_05888	CDS	COG0227	6133469	6133705	-	[J] Ribosomal protein L28
2982							
	MJLIPHIC_05889	CDS	COG0523	6133779	6134993	+	[R] Putative GTPases (G3E family)
	MJLIPHIC_05890	CDS	COG0254	6134990	6135238	+	[J] Ribosomal protein L31
	MJLIPHIC_05891	CDS	ROG0058	6135261	6136205	+	NA
	MJLIPHIC_05892	CDS	COG1055	6136154	6137437	+	[P] Na+/H+ antiporter NhaD and related arsenite
2983							
	MJLIPHIC_05893	CDS	COG0566	6137444	6138388	-	[J] rRNA methylases
	MJLIPHIC_05894	CDS	COG0215	6138388	6139635	-	[J] Cysteinyl-tRNA synthetase
	MJLIPHIC_05895	CDS	COG0245	6139851	6140342	-	[I] 2C-methyl-D-erythritol 2,4-cyclodiphosphate synthase
	MJLIPHIC_05896	CDS	COG1211	6140339	6141007	-	[I] 4-diphosphocytidyl-2-methyl-D-erithritol synthase
	MJLIPHIC_05897	CDS	COG1329	6141027	6141515	-	[K] Transcriptional regulators, similar to M. xanthus
2984							
	MJLIPHIC_05898	CDS	ROG4184	6141800	6142360	+	NA
	MJLIPHIC_05899	CDS	COG1066	6142417	6143817	+	[O] Predicted ATP-dependent serine protease
	MJLIPHIC_05900	CDS	COG1623	6143853	6144971	+	[R] Predicted nucleic-acid-binding protein (contains the HHH
2985							
	MJLIPHIC_05901	CDS	ROG0995	6144973	6145770	-	NA
	MJLIPHIC_05902	CDS	COG0288	6145828	6146448	-	[P] Carbonic anhydrase
2986							
	MJLIPHIC_05903	CDS	COG1194	6146474	6147355	+	[L] A/G-specific DNA glycosylase
2987							
	MJLIPHIC_05904	CDS	COG4977	6147363	6147995	-	[K] Transcriptional regulator containing an amidase domain
2988							
	MJLIPHIC_05905	CDS	NA	6148447	6148701	+	NA
2989							
	MJLIPHIC_05906	CDS	COG0596	6148672	6149445	-	[R] Predicted hydrolases or acyltransferases (alpha/beta hydrolase
2990							
	MJLIPHIC_05907	CDS	COG2329	6149485	6149802	+	[R] Uncharacterized enzyme involved in biosynthesis of
	MJLIPHIC_05908	CDS	COG2367	6149846	6151165	+	[V] Beta-lactamase class A
2991							
	MJLIPHIC_05909	CDS	COG0406	6151170	6151718	-	[G] Fructose-2,6-bisphosphatase
	MJLIPHIC_05910	CDS	ROG0896	6151715	6152521	-	NA
	MJLIPHIC_05911	CDS	ROG2078	6152546	6152758	-	NA
2992							
	MJLIPHIC_05912	CDS	COG0542	6152971	6155517	-	[O] ATPases with chaperone activity, ATP-binding subunit
2993							
	MJLIPHIC_05913	CDS	ROG3474	6155786	6156130	-	NA
2994							
	MJLIPHIC_05914	CDS	COG1190	6156246	6157769	-	[J] Lysyl-tRNA synthetase (class II)
2995							
	MJLIPHIC_05915	CDS	COG3616	6157793	6159031	+	[E] Predicted amino acid aldolase or racemase
2996							
	MJLIPHIC_05916	CDS	COG1521	6159032	6159847	-	[K] Putative transcriptional regulator, homolog of Bvg
	MJLIPHIC_05917	CDS	COG0414	6159923	6160867	-	[H] Panthothenate synthetase
	MJLIPHIC_05918	CDS	COG5495	6160864	6161811	-	[S] Uncharacterized conserved protein
2997							
	MJLIPHIC_05919	CDS	ROG6007	6161892	6163403	-	NA
2998							
	MJLIPHIC_05920	CDS	ROG0812	6163589	6164065	-	NA
	MJLIPHIC_05921	CDS	COG0801	6164065	6164601	-	[H] 7,8-dihydro-6-hydroxymethylpterin-pyrophosphokinase
	MJLIPHIC_05922	CDS	COG1539	6164598	6164969	-	[H] Dihydroneopterin aldolase
	MJLIPHIC_05923	CDS	COG0294	6164962	6165819	-	[H] Dihydropteroate synthase and related enzymes
	MJLIPHIC_05924	CDS	COG0302	6165816	6166427	-	[H] GTP cyclohydrolase I
	MJLIPHIC_05925	CDS	COG0465	6166447	6168759	-	[O] ATP-dependent Zn proteases
2999							

	MJLIPHIC_05926	CDS	COG0596	6168944	6169927	+	[R] Predicted hydrolases or acyltransferases (alpha/beta hydrolase
	MJLIPHIC_05927	CDS	COG2141	6169927	6171114	+	[C] Coenzyme F420-dependent N5,N10-methylene tetrahydromethanopterin reductase and
	MJLIPHIC_05928	CDS	COG1063	6171114	6172130	+	[ER] Threonine dehydrogenase and related Zn-dependent dehydrogenases
	MJLIPHIC_05929	CDS	COG2968	6172152	6172883	+	[S] Uncharacterized conserved protein
3000							
	MJLIPHIC_05930	CDS	COG0634	6172888	6173523	-	[F] Hypoxanthine-guanine phosphoribosyltransferase
	MJLIPHIC_05931	CDS	COG0037	6173520	6174449	-	[D] Predicted ATPase of the PP-loop superfamily
	MJLIPHIC_05932	CDS	COG5282	6174467	6175540	-	[S] Uncharacterized conserved protein
	MJLIPHIC_05933	CDS	COG2027	6175537	6176919	-	[M] D-alanyl-D-alanine carboxypeptidase (penicillin-binding protein 4)
3001							
	MJLIPHIC_05934	CDS	COG0221	6177063	6177551	+	[C] Inorganic pyrophosphatase
3002							
	MJLIPHIC_05935	CDS	COG3195	6177618	6178142	+	[S] Uncharacterized protein conserved in bacteria
3003							
	MJLIPHIC_05936	CDS	COG1052	6178118	6179122	-	[CHR] Lactate dehydrogenase and related dehydrogenases
	MJLIPHIC_05937	CDS	COG4948	6179128	6180477	-	[MR] L-alanine-DL-glutamate epimerase and related enzymes of
	MJLIPHIC_05938	CDS	COG1414	6180503	6181279	-	[K] Transcriptional regulator
	MJLIPHIC_05939	CDS	NA	6181305	6182027	-	NA
	MJLIPHIC_05940	CDS	COG4608	6182024	6183022	-	[E] ABC-type oligopeptide transport system, ATPase component
	MJLIPHIC_05941	CDS	COG1123	6183015	6184046	-	[R] ATPase components of various ABC-type transport
	MJLIPHIC_05942	CDS	COG1173	6184043	6185029	-	[EP] ABC-type dipeptide/oligopeptide/nickel transport systems, permease components
	MJLIPHIC_05943	CDS	COG0601	6185038	6185967	-	[EP] ABC-type dipeptide/oligopeptide/nickel transport systems, permease components
	MJLIPHIC_05944	CDS	COG0747	6185995	6187557	-	[E] ABC-type dipeptide transport system, periplasmic component
3004							
	MJLIPHIC_05945	CDS	COG1052	6187851	6188864	+	[CHR] Lactate dehydrogenase and related dehydrogenases
3005							
	MJLIPHIC_05946	CDS	COG0583	6188858	6189787	-	[K] Transcriptional regulator
3006							
	MJLIPHIC_05947	CDS	COG1366	6190044	6190403	+	[T] Anti-anti-sigma regulatory factor (antagonist of anti-sigma
	MJLIPHIC_05948	CDS	COG2172	6190437	6190883	+	[T] Anti-sigma regulatory factor (Ser/Thr protein kinase)
3007							
	MJLIPHIC_05949	CDS	COG0784	6190869	6191315	-	[T] FOG: CheY-like receiver
	MJLIPHIC_05950	CDS	COG4251	6191312	6192889	-	[T] Bacteriophytochrome (light-regulated signal transduction histidine kinase)
	MJLIPHIC_05951	CDS	COG2208	6192886	6194094	-	[TK] Serine phosphatase RsbU, regulator of sigma
3008							
	MJLIPHIC_05952	CDS	COG4948	6194325	6195596	-	[MR] L-alanine-DL-glutamate epimerase and related enzymes of
	MJLIPHIC_05953	CDS	COG0329	6195607	6196530	-	[EM] Dihydrodipicolinate synthase/N-acetylneuraminate lyase
	MJLIPHIC_05954	CDS	COG1012	6196561	6198150	-	[C] NAD-dependent aldehyde dehydrogenases
3009							
	MJLIPHIC_05955	CDS	NA	6198302	6198706	-	NA
3010							
	MJLIPHIC_05956	CDS	COG0861	6198808	6199776	-	[P] Membrane protein TerC, possibly involved in
3011							
	MJLIPHIC_05957	CDS	COG1020	6200005	6203940	+	[Q] Non-ribosomal peptide synthetase modules and related
	MJLIPHIC_05958	CDS	COG0308	6203937	6205274	+	[E] Aminopeptidase N
	MJLIPHIC_05959	CDS	ROG5013	6205271	6206551	+	NA
3012							
	MJLIPHIC_05960	CDS	COG1309	6206541	6207134	-	[K] Transcriptional regulator
	MJLIPHIC_05961	CDS	COG3653	6207136	6208917	-	[Q] N-acyl-D-aspartate/D-glutamate deacylase
3013							
	MJLIPHIC_05962	CDS	COG0451	6208979	6209872	-	[MG] Nucleoside-diphosphate-sugar epimerases
3014							
	MJLIPHIC_05963	CDS	ROG2129	6209977	6211740	+	NA
3015							
	MJLIPHIC_05964	CDS	COG0400	6211742	6213211	-	[R] Predicted esterase
3016							
	MJLIPHIC_05965	CDS	NA	6213394	6214221	+	NA
3017							
	MJLIPHIC_05966	CDS	ROG0006	6214302	6215708	+	NA
3018							
	MJLIPHIC_05967	CDS	NA	6216168	6216974	+	NA
3019							
	MJLIPHIC_05968	CDS	COG3464	6217472	6218383	+	[L] Transposase and inactivated derivatives
3020							
	MJLIPHIC_05969	CDS	ROG0016	6218391	6219143	-	NA
3021							
	MJLIPHIC_05970	CDS	COG0599	6219667	6220032	+	[S] Uncharacterized homolog of gamma-carboxymuconolactone decarboxylase subunit
	MJLIPHIC_05971	CDS	COG0596	6220029	6220904	+	[R] Predicted hydrolases or acyltransferases (alpha/beta hydrolase
3022							
	MJLIPHIC_05972	tRNA	NA	6220990	6221063	-	NA
3023							
	MJLIPHIC_05973	CDS	COG2812	6221127	6222335	-	[L] DNA polymerase III, gamma/tau subunits
3024							
	MJLIPHIC_05974	CDS	COG2114	6222417	6224108	+	[T] Adenylate cyclase, family 3 (some proteins
3025							
	MJLIPHIC_05975	CDS	COG3464	6224577	6225488	+	[L] Transposase and inactivated derivatives
3026							
	MJLIPHIC_05976	CDS	ROG0016	6225496	6226248	-	NA
3027							
	MJLIPHIC_05977	CDS	COG0550	6226360	6229119	-	[L] Topoisomerase IA
3028							
	MJLIPHIC_05978	CDS	ROG4682	6229274	6229867	-	NA
3029							
	MJLIPHIC_05979	CDS	COG1278	6229963	6230166	-	[K] Cold shock proteins
3030							
	MJLIPHIC_05980	CDS	COG1205	6230468	6232798	+	[R] Distinct helicase family with a unique
3031							
	MJLIPHIC_05981	CDS	COG3464	6233481	6234392	+	[L] Transposase and inactivated derivatives
3032							
	MJLIPHIC_05982	CDS	ROG0016	6234400	6235152	-	NA
3033							
	MJLIPHIC_05983	CDS	NA	6235338	6236363	+	NA
3034							
	MJLIPHIC_05984	CDS	NA	6236448	6236774	-	NA
	MJLIPHIC_05985	CDS	NA	6236785	6237051	-	NA
	MJLIPHIC_05986	CDS	ROG3712	6237108	6237317	-	NA
	MJLIPHIC_05987	CDS	COG2064	6237351	6237920	-	[NU] Flp pilus assembly protein TadC
	MJLIPHIC_05988	CDS	COG4965	6237917	6238591	-	[U] Flp pilus assembly protein TadB
	MJLIPHIC_05989	CDS	COG4962	6238696	6239853	-	[U] Flp pilus assembly protein, ATPase CpaF
	MJLIPHIC_05990	CDS	COG0455	6239850	6240938	-	[D] ATPases involved in chromosome partitioning

3035							
	MJLIPHIC_05991	CDS	COG0560	6241372	6242253	+	[E] Phosphoserine phosphatase
3036							
	MJLIPHIC_05992	CDS	COG1414	6242692	6243474	-	[K] Transcriptional regulator
3037							
	MJLIPHIC_05993	CDS	COG3734	6243533	6244549	+	[G] 2-keto-3-deoxy-galactonokinase
	MJLIPHIC_05994	CDS	COG0800	6244546	6245172	+	[G] 2-keto-3-deoxy-6-phosphogluconate aldolase
	MJLIPHIC_05995	CDS	COG4948	6245169	6246317	+	[MR] L-alanine-DL-glutamate epimerase and related enzymes of
	MJLIPHIC_05996	CDS	COG2271	6246323	6247654	+	[G] Sugar phosphate permease
3038							
	MJLIPHIC_05997	CDS	ROG1157	6247656	6248411	-	NA
3039							
	MJLIPHIC_05998	CDS	COG0365	6248539	6250494	+	[I] Acyl-coenzyme A synthetases/AMP-(fatty) acid ligases
3040							
	MJLIPHIC_05999	CDS	ROG2168	6250558	6251157	-	NA
3041							
	MJLIPHIC_06000	CDS	ROG1387	6251411	6251920	+	NA
	MJLIPHIC_06001	CDS	COG0596	6251920	6252870	+	[R] Predicted hydrolases or acyltransferases (alpha/beta hydrolase
3042							
	MJLIPHIC_06002	CDS	ROG0187	6252860	6254050	-	NA
	MJLIPHIC_06003	CDS	COG0494	6254050	6254838	-	[LR] NTP pyrophosphohydrolases including oxidative damage repair
	MJLIPHIC_06004	CDS	COG0526	6254850	6255497	-	[OC] Thiol-disulfide isomerase and thioredoxins
	MJLIPHIC_06005	CDS	COG0177	6255512	6256312	-	[L] Predicted EndoIII-related endonuclease
3043							
	MJLIPHIC_06006	CDS	NA	6256441	6256734	+	NA
3044							
	MJLIPHIC_06007	CDS	COG0664	6256824	6257498	+	[T] cAMP-binding proteins - catabolite gene activator
3045							
	MJLIPHIC_06008	CDS	COG0491	6257583	6258377	-	[R] Zn-dependent hydrolases, including glyoxylases
	MJLIPHIC_06009	CDS	COG0251	6258374	6258850	-	[J] Putative translation initiation inhibitor, yjgF family
	MJLIPHIC_06010	CDS	ROG0483	6258847	6259008	-	NA
3046							
	MJLIPHIC_06011	CDS	COG0003	6259056	6260090	+	[P] Oxyanion-translocating ATPase
	MJLIPHIC_06012	CDS	COG0003	6260087	6261217	+	[P] Oxyanion-translocating ATPase
3047							
	MJLIPHIC_06013	CDS	COG2367	6261214	6262032	-	[V] Beta-lactamase class A
	MJLIPHIC_06014	CDS	COG1113	6262025	6263446	-	[E] Gamma-aminobutyrate permease and related permeases
	MJLIPHIC_06015	CDS	COG0019	6263460	6264839	-	[E] Diaminopimelate decarboxylase
3048							
	MJLIPHIC_06016	CDS	COG2508	6264963	6266543	-	[TQ] Regulator of polyketide synthase expression
3049							
	MJLIPHIC_06017	CDS	ROG0386	6266743	6267123	-	NA
3050							
	MJLIPHIC_06018	CDS	COG0744	6267396	6269849	+	[M] Membrane carboxypeptidase (penicillin-binding protein)
3051							
	MJLIPHIC_06019	CDS	COG1408	6269949	6270935	+	[R] Predicted phosphohydrolases
	MJLIPHIC_06020	CDS	COG0031	6270944	6272041	+	[E] Cysteine synthase
3052							
	MJLIPHIC_06021	CDS	NA	6271945	6272169	-	NA
3053							
	MJLIPHIC_06022	tRNA	NA	6272367	6272441	+	NA
3054							
	MJLIPHIC_06023	CDS	NA	6272573	6272785	+	NA
3055							
	MJLIPHIC_06024	CDS	COG0515	6272798	6273946	-	[RTKL] Serine/threonine protein kinase
3056							
	MJLIPHIC_06025	CDS	ROG4322	6274084	6274581	+	NA
	MJLIPHIC_06026	CDS	COG1946	6274619	6275416	+	[I] Acyl-CoA thioesterase
3057							
	MJLIPHIC_06027	CDS	NA	6275532	6275699	+	NA
	MJLIPHIC_06028	CDS	ROG3648	6275739	6276398	+	NA
3058							
	MJLIPHIC_06029	CDS	NA	6276404	6276556	-	NA
3059							
	MJLIPHIC_06030	CDS	ROG3432	6276681	6277265	+	NA
3060							
	MJLIPHIC_06031	CDS	COG3546	6277327	6278220	-	[P] Mn-containing catalase
3061							
	MJLIPHIC_06032	CDS	COG1610	6278296	6278760	-	[S] Uncharacterized conserved protein
	MJLIPHIC_06033	CDS	COG2968	6278771	6279496	-	[S] Uncharacterized conserved protein
3062							
	MJLIPHIC_06034	CDS	ROG0538	6279513	6280592	+	NA
	MJLIPHIC_06035	CDS	ROG1840	6280594	6281244	+	NA
	MJLIPHIC_06036	CDS	ROG1458	6281241	6282347	+	NA
	MJLIPHIC_06037	CDS	COG0714	6282344	6283315	+	[R] MoxR-like ATPases
	MJLIPHIC_06038	CDS	COG1721	6283315	6284637	+	[R] Uncharacterized conserved protein (some members contain
3063							
	MJLIPHIC_06039	CDS	COG1300	6284642	6285574	-	[S] Uncharacterized membrane protein
3064							
	MJLIPHIC_06040	CDS	COG1714	6285605	6286591	+	[S] Predicted membrane protein/domain
	MJLIPHIC_06041	CDS	COG1309	6286641	6287210	+	[K] Transcriptional regulator
	MJLIPHIC_06042	CDS	COG0477	6287282	6288796	+	[GEPR] Permeases of the major facilitator superfamily
3065							
	MJLIPHIC_06043	CDS	COG3670	6288786	6290105	-	[Q] Lignostilbene-alpha,beta-dioxygenase and related enzymes
3066							
	MJLIPHIC_06044	CDS	COG1695	6290203	6290715	+	[K] Predicted transcriptional regulators
	MJLIPHIC_06045	CDS	COG1695	6290798	6291538	+	[K] Predicted transcriptional regulators
	MJLIPHIC_06046	CDS	NA	6291559	6292239	+	NA
3067							
	MJLIPHIC_06047	CDS	COG0554	6292243	6293805	-	[C] Glycerol kinase
3068							
	MJLIPHIC_06048	CDS	COG0500	6293807	6294562	+	[QR] SAM-dependent methyltransferases
3069							
	MJLIPHIC_06049	CDS	COG3936	6294559	6295710	-	[G] Protein involved in polysaccharide intercellular adhesin
	MJLIPHIC_06050	CDS	COG0753	6295741	6297402	-	[P] Catalase
3070							
	MJLIPHIC_06051	CDS	COG2220	6297491	6298297	-	[R] Predicted Zn-dependent hydrolases of the beta-lactamase
3071							
	MJLIPHIC_06052	CDS	ROG0182	6298361	6299845	+	NA
	MJLIPHIC_06053	CDS	COG0500	6299865	6300536	+	[QR] SAM-dependent methyltransferases

	MJLIPHIC_06054	CDS	COG0500	6300742	6301623	+	[QR] SAM-dependent methyltransferases
3072							
	MJLIPHIC_06055	CDS	COG2197	6301620	6302270	-	[TK] Response regulator containing a CheY-like receiver
	MJLIPHIC_06056	CDS	COG4585	6302267	6303418	-	[T] Signal transduction histidine kinase
3073							
	MJLIPHIC_06057	CDS	COG1454	6303617	6304828	-	[C] Alcohol dehydrogenase, class IV
3074							
	MJLIPHIC_06058	CDS	COG3552	6304953	6306446	-	[R] Protein containing von Willebrand factor type
	MJLIPHIC_06059	CDS	COG0464	6306443	6307501	-	[O] ATPases of the AAA+ class
	MJLIPHIC_06060	CDS	COG1454	6307532	6308803	-	[C] Alcohol dehydrogenase, class IV
3075							
	MJLIPHIC_06061	CDS	COG3558	6308975	6309448	-	[S] Uncharacterized protein conserved in bacteria
3076							
	MJLIPHIC_06062	CDS	COG1309	6309530	6310090	+	[K] Transcriptional regulator
3077							
	MJLIPHIC_06063	CDS	COG2814	6310092	6311297	-	[G] Arabinose efflux permease
3078							
	MJLIPHIC_06064	CDS	COG0520	6311487	6312599	-	[E] Selenocysteine lyase
	MJLIPHIC_06065	CDS	COG4301	6312599	6313564	-	[S] Uncharacterized conserved protein
	MJLIPHIC_06066	CDS	COG0121	6313576	6314259	-	[R] Predicted glutamine amidotransferase
	MJLIPHIC_06067	CDS	COG1262	6314259	6315545	-	[S] Uncharacterized conserved protein
	MJLIPHIC_06068	CDS	COG3572	6315542	6316813	-	[H] Gamma-glutamylcysteine synthetase
3079							
	MJLIPHIC_06069	CDS	NA	6316930	6317583	-	NA
	MJLIPHIC_06070	CDS	NA	6317589	6318089	-	NA
3080							
	MJLIPHIC_06071	CDS	COG0735	6318227	6318562	-	[P] Fe2+/Zn2+ uptake regulation proteins
3081							
	MJLIPHIC_06072	CDS	NA	6318744	6319214	-	NA
3082							
	MJLIPHIC_06073	CDS	ROG5359	6319355	6320356	-	NA
3083							
	MJLIPHIC_06074	CDS	COG0136	6320473	6321513	-	[E] Aspartate-semialdehyde dehydrogenase
	MJLIPHIC_06075	CDS	COG0527	6321515	6322780	-	[E] Aspartokinases
3084							
	MJLIPHIC_06076	CDS	COG0778	6322815	6323498	+	[C] Nitroreductase
3085							
	MJLIPHIC_06077	CDS	COG0004	6323614	6324864	+	[P] Ammonia permease
	MJLIPHIC_06078	CDS	COG0174	6324998	6326302	+	[E] Glutamine synthetase
	MJLIPHIC_06079	CDS	COG0067	6326334	6327203	+	[E] Glutamate synthase domain 1
	MJLIPHIC_06080	CDS	COG2218	6327191	6327886	+	[C] Formylmethanofuran dehydrogenase subunit C
	MJLIPHIC_06081	CDS	COG0069	6327886	6329226	+	[E] Glutamate synthase domain 2
	MJLIPHIC_06082	CDS	COG0665	6329223	6330410	+	[E] Glycine/D-amino acid oxidases (deaminating)
	MJLIPHIC_06083	CDS	COG1396	6330437	6331087	+	[K] Predicted transcriptional regulators
3086							
	MJLIPHIC_06084	CDS	ROG0365	6331220	6332059	+	NA
	MJLIPHIC_06085	CDS	ROG0342	6332056	6332766	+	NA
	MJLIPHIC_06086	CDS	ROG0365	6332770	6333120	+	NA
	MJLIPHIC_06087	CDS	COG2239	6333151	6334521	+	[P] Mg/Co/Ni transporter MgtE (contains CBS domain)
3087							
	MJLIPHIC_06088	CDS	COG0119	6334688	6336496	+	[E] Isopropylmalate/homocitrate/citramalate synthases
3088							
	MJLIPHIC_06089	CDS	COG2902	6336680	6339892	+	[E] NAD-specific glutamate dehydrogenase
3089							
	MJLIPHIC_06090	CDS	ROG3910	6340016	6340402	+	NA
3090							
	MJLIPHIC_06091	CDS	COG0847	6340409	6341389	-	[L] DNA polymerase III, epsilon subunit and
3091							
	MJLIPHIC_06092	CDS	COG0769	6341496	6342719	+	[M] UDP-N-acetylmuramyl tripeptide synthase
	MJLIPHIC_06093	CDS	COG3442	6342716	6343435	+	[R] Predicted glutamine amidotransferase
3092							
	MJLIPHIC_06094	CDS	COG0491	6343526	6344236	-	[R] Zn-dependent hydrolases, including glyoxylases
3093							
	MJLIPHIC_06095	CDS	COG0353	6344309	6344920	-	[L] Recombinational DNA repair protein (RecF pathway)
	MJLIPHIC_06096	CDS	COG0718	6344931	6345239	-	[S] Uncharacterized protein conserved in bacteria
3094							
	MJLIPHIC_06097	CDS	COG0860	6345388	6346182	+	[M] N-acetylmuramoyl-L-alanine amidase
3095							
	MJLIPHIC_06098	CDS	ROG2488	6346190	6346627	-	NA
3096							
	MJLIPHIC_06099	CDS	NA	6346723	6348117	+	NA
	MJLIPHIC_06100	CDS	COG2230	6348114	6349427	+	[M] Cyclopropane fatty acid synthase and related
3097							
	MJLIPHIC_06101	CDS	COG2812	6349463	6351460	-	[L] DNA polymerase III, gamma/tau subunits
	MJLIPHIC_06102	CDS	COG1167	6351487	6352773	-	[KE] Transcriptional regulators containing a DNA-binding HTH
3098							
	MJLIPHIC_06103	tRNA	NA	6352992	6353078	+	NA
3099							
	MJLIPHIC_06104	CDS	ROG0195	6353155	6354036	+	NA
	MJLIPHIC_06105	CDS	COG0265	6354081	6355103	+	[O] Trypsin-like serine proteases, typically periplasmic, contain
	MJLIPHIC_06106	CDS	COG2508	6355142	6356653	+	[TQ] Regulator of polyketide synthase expression
3100							
	MJLIPHIC_06107	CDS	COG0665	6356801	6358051	+	[E] Glycine/D-amino acid oxidases (deaminating)
3101							
	MJLIPHIC_06108	CDS	COG0782	6358172	6358645	+	[K] Transcription elongation factor
	MJLIPHIC_06109	CDS	COG1309	6358686	6359258	+	[K] Transcriptional regulator
3102							
	MJLIPHIC_06110	CDS	COG1804	6359488	6360657	+	[C] Predicted acyl-CoA transferases/carnitine dehydratase
	MJLIPHIC_06111	CDS	COG0477	6360654	6362012	+	[GEPR] Permeases of the major facilitator superfamily
	MJLIPHIC_06112	CDS	COG1063	6362009	6363139	+	[ER] Threonine dehydrogenase and related Zn-dependent dehydrogenases
	MJLIPHIC_06113	CDS	COG1012	6363136	6364671	+	[C] NAD-dependent aldehyde dehydrogenases
	MJLIPHIC_06114	CDS	COG2301	6364671	6365567	+	[G] Citrate lyase beta subunit
	MJLIPHIC_06115	CDS	COG1052	6365564	6366517	+	[CHR] Lactate dehydrogenase and related dehydrogenases
	MJLIPHIC_06116	CDS	COG1802	6366547	6367221	+	[K] Transcriptional regulators
3103							
	MJLIPHIC_06117	CDS	COG3285	6367237	6368286	-	[L] Predicted eukaryotic-type DNA primase
	MJLIPHIC_06118	CDS	COG1793	6368286	6369341	-	[L] ATP-dependent DNA ligase
3104							
	MJLIPHIC_06119	CDS	COG1793	6369392	6370456	+	[L] ATP-dependent DNA ligase
3105							

	MJLIPHIC_06120	CDS	COG2272	6370453	6371685	-	[I] Carboxylesterase type B
3106							
	MJLIPHIC_06121	CDS	NA	6371770	6372138	+	NA
	MJLIPHIC_06122	CDS	COG0008	6372152	6373030	+	[J] Glutamyl- and glutaminyl-tRNA synthetases
3107							
	MJLIPHIC_06123	CDS	COG0765	6373123	6374550	+	[E] ABC-type amino acid transport system, permease
	MJLIPHIC_06124	CDS	COG1126	6374547	6375320	+	[E] ABC-type polar amino acid transport system,
3108							
	MJLIPHIC_06125	CDS	COG0427	6375427	6376752	-	[C] Acetyl-CoA hydrolase
	MJLIPHIC_06126	CDS	COG1748	6376779	6377996	-	[E] Saccharopine dehydrogenase and related proteins
	MJLIPHIC_06127	CDS	COG2124	6378073	6379317	-	[Q] Cytochrome P450
	MJLIPHIC_06128	CDS	COG0343	6379351	6380595	-	[J] Queuine/archaeosine tRNA-ribosyltransferase
3109							
	MJLIPHIC_06129	CDS	ROG1065	6380676	6381155	+	NA
3110							
	MJLIPHIC_06130	CDS	COG0596	6381142	6381987	-	[R] Predicted hydrolases or acyltransferases (alpha/beta hydrolase
3111							
	MJLIPHIC_06131	CDS	ROG2096	6382186	6382716	+	NA
3112							
	MJLIPHIC_06132	CDS	COG2755	6382820	6383470	+	[E] Lysophospholipase L1 and related esterases
	MJLIPHIC_06133	CDS	COG0768	6383481	6385259	+	[M] Cell division protein FtsI/penicillin-binding protein 2
3113							
	MJLIPHIC_06134	CDS	ROG4508	6385224	6386960	-	NA
	MJLIPHIC_06135	CDS	COG4910	6386957	6387301	-	[Q] Propanediol dehydratase, small subunit
	MJLIPHIC_06136	CDS	COG4909	6387298	6389571	-	[Q] Propanediol dehydratase, large subunit
	MJLIPHIC_06137	CDS	ROG5027	6389612	6390985	-	NA
3114							
	MJLIPHIC_06138	CDS	NA	6391100	6391510	+	NA
	MJLIPHIC_06139	CDS	ROG5051	6391470	6393257	+	NA
	MJLIPHIC_06140	CDS	ROG1229	6393250	6393789	+	NA
3115							
	MJLIPHIC_06141	tRNA	NA	6393822	6393912	-	NA
3116							
	MJLIPHIC_06142	CDS	COG0590	6394144	6394593	-	[FJ] Cytosine/adenosine deaminases
	MJLIPHIC_06143	CDS	ROG1142	6394590	6395096	-	NA
3117							
	MJLIPHIC_06144	CDS	COG0287	6395196	6396140	+	[E] Prephenate dehydrogenase
3118							
	MJLIPHIC_06145	CDS	COG3554	6396103	6396705	-	[S] Uncharacterized protein conserved in bacteria
	MJLIPHIC_06146	CDS	COG1174	6396702	6397508	-	[E] ABC-type proline/glycine betaine transport systems, permease
	MJLIPHIC_06147	CDS	COG1174	6397505	6398149	-	[E] ABC-type proline/glycine betaine transport systems, permease
	MJLIPHIC_06148	CDS	COG1125	6398149	6399255	-	[E] ABC-type proline/glycine betaine transport systems, ATPase
	MJLIPHIC_06149	CDS	COG1732	6399266	6399955	-	[M] Periplasmic glycine betaine/choline-binding (lipo)protein of an
3119							
	MJLIPHIC_06150	CDS	COG5416	6400335	6400700	+	[S] Uncharacterized integral membrane protein
3120							
	MJLIPHIC_06151	CDS	COG3173	6400727	6401740	-	[R] Predicted aminoglycoside phosphotransferase
	MJLIPHIC_06152	CDS	COG0406	6401750	6402358	-	[G] Fructose-2,6-bisphosphatase
3121							
	MJLIPHIC_06153	CDS	COG1028	6402432	6403187	+	[IQR] Dehydrogenases with different specificities (related to
3122							
	MJLIPHIC_06154	CDS	COG2015	6403184	6405064	-	[Q] Alkyl sulfatase and related hydrolases
	MJLIPHIC_06155	CDS	COG2084	6405078	6405956	-	[I] 3-hydroxyisobutyrate dehydrogenase and related beta-hydroxyacid dehydrogenases
	MJLIPHIC_06156	CDS	COG2084	6405953	6406783	-	[I] 3-hydroxyisobutyrate dehydrogenase and related beta-hydroxyacid dehydrogenases
3123							
	MJLIPHIC_06157	CDS	COG1733	6406956	6407252	+	[K] Predicted transcriptional regulators
3124							
	MJLIPHIC_06158	CDS	COG0622	6407249	6407746	-	[R] Predicted phosphoesterase
3125							
	MJLIPHIC_06159	CDS	ROG4104	6407962	6408258	+	NA
3126							
	MJLIPHIC_06160	CDS	NA	6408351	6408779	+	NA
3127							
	MJLIPHIC_06161	CDS	NA	6408784	6409452	-	NA
3128							
	MJLIPHIC_06162	CDS	NA	6409714	6410292	+	NA
3129							
	MJLIPHIC_06163	tRNA	NA	6410365	6410438	-	NA
	MJLIPHIC_06164	tRNA	NA	6410476	6410567	-	NA
3130							
	MJLIPHIC_06165	CDS	COG0079	6410678	6411739	+	[E] Histidinol-phosphate/aromatic aminotransferase and cobyric acid decarboxylase
	MJLIPHIC_06166	CDS	ROG1622	6411736	6412209	+	NA
	MJLIPHIC_06167	CDS	COG1024	6412212	6413027	+	[I] Enoyl-CoA hydratase/carnithine racemase
3131							
	MJLIPHIC_06168	CDS	ROG5614	6413230	6413949	+	NA
3132							
	MJLIPHIC_06169	CDS	NA	6414231	6414500	+	NA
3133							
	MJLIPHIC_06170	CDS	COG2207	6414574	6415320	-	[K] AraC-type DNA-binding domain-containing proteins
3134							
	MJLIPHIC_06171	CDS	NA	6415683	6416591	+	NA
	MJLIPHIC_06172	CDS	ROG2168	6416588	6417490	+	NA
3135							
	MJLIPHIC_06173	tRNA	NA	6417689	6417776	-	NA
	MJLIPHIC_06174	CDS	COG1846	6417795	6418307	-	[K] Transcriptional regulators
3136							
	MJLIPHIC_06175	CDS	COG0604	6418375	6419352	+	[CR] NADPH:quinone reductase and related Zn-dependent oxidoreductases
3137							
	MJLIPHIC_06176	CDS	COG0520	6419371	6420567	-	[E] Selenocysteine lyase
3138							
	MJLIPHIC_06177	CDS	ROG1041	6421207	6421734	+	NA
	MJLIPHIC_06178	CDS	COG1134	6421742	6422551	+	[GM] ABC-type polysaccharide/polyol phosphate transport system, ATPase
	MJLIPHIC_06179	CDS	COG1216	6422538	6423446	+	[R] Predicted glycosyltransferases
	MJLIPHIC_06180	CDS	COG1682	6423443	6424273	+	[GM] ABC-type polysaccharide/polyol phosphate export systems, permease
3139							
	MJLIPHIC_06181	CDS	ROG1141	6424261	6424665	-	NA
3140							
	MJLIPHIC_06182	CDS	COG2128	6424775	6425236	-	[S] Uncharacterized conserved protein
3141							
	MJLIPHIC_06183	CDS	COG1167	6425285	6426694	+	[KE] Transcriptional regulators containing a DNA-binding HTH

	MJLIPHIC_06184	CDS	COG2246	6426713	6427120	+	[S] Predicted membrane protein
3142							
	MJLIPHIC_06185	CDS	COG3246	6427130	6428068	-	[S] Uncharacterized conserved protein
	MJLIPHIC_06186	CDS	COG1893	6428080	6429102	-	[H] Ketopantoate reductase
	MJLIPHIC_06187	CDS	NA	6429099	6430376	-	NA
3143							
	MJLIPHIC_06188	CDS	COG1609	6430444	6431502	-	[K] Transcriptional regulators
3144							
	MJLIPHIC_06189	CDS	COG3386	6431722	6432624	+	[G] Gluconolactonase
	MJLIPHIC_06190	CDS	NA	6432650	6433981	+	NA
3145							
	MJLIPHIC_06191	CDS	NA	6434002	6434412	-	NA
3146							
	MJLIPHIC_06192	CDS	ROG1748	6434939	6436762	-	NA
3147							
	MJLIPHIC_06193	CDS	COG0277	6436944	6438350	+	[C] FAD/FMN-containing dehydrogenases
	MJLIPHIC_06194	CDS	COG0735	6438415	6438876	+	[P] Fe2+/Zn2+ uptake regulation proteins
	MJLIPHIC_06195	CDS	COG0376	6438913	6441132	+	[P] Catalase (peroxidase I)
3148							
	MJLIPHIC_06196	CDS	COG0300	6441262	6442026	+	[R] Short-chain dehydrogenases of various substrate specificities
	MJLIPHIC_06197	CDS	ROG2066	6442026	6443906	+	NA
	MJLIPHIC_06198	CDS	ROG7926	6443896	6447153	+	NA
3149							
	MJLIPHIC_06199	CDS	ROG2152	6447287	6450529	+	NA
	MJLIPHIC_06200	CDS	ROG2152	6450493	6453774	+	NA
3150							
	MJLIPHIC_06201	CDS	NA	6453920	6455158	+	NA
3151							
	MJLIPHIC_06202	CDS	COG4799	6455190	6456743	-	[I] Acetyl-CoA carboxylase, carboxyltransferase component (subunits alpha
	MJLIPHIC_06203	CDS	COG3321	6456740	6462265	-	[Q] Polyketide synthase modules and related proteins
	MJLIPHIC_06204	CDS	COG0318	6462325	6464217	-	[IQ] Acyl-CoA synthetases (AMP-forming)/AMP-acid ligases II
3152							
	MJLIPHIC_06205	CDS	ROG2666	6464462	6465475	-	NA
	MJLIPHIC_06206	CDS	NA	6465494	6465871	-	NA
3153							
	MJLIPHIC_06207	CDS	COG0627	6466034	6466936	-	[R] Predicted esterase
	MJLIPHIC_06208	CDS	COG0627	6467091	6468098	-	[R] Predicted esterase
	MJLIPHIC_06209	CDS	COG0627	6468235	6469248	-	[R] Predicted esterase
3154							
	MJLIPHIC_06210	CDS	ROG2752	6469392	6471431	-	NA
	MJLIPHIC_06211	CDS	ROG5249	6471409	6472365	-	NA
	MJLIPHIC_06212	CDS	COG0671	6472402	6472944	-	[I] Membrane-associated phospholipid phosphatase
	MJLIPHIC_06213	CDS	COG1216	6472941	6474881	-	[R] Predicted glycosyltransferases
	MJLIPHIC_06214	CDS	COG0562	6474878	6476116	-	[M] UDP-galactopyranose mutase
3155							
	MJLIPHIC_06215	CDS	NA	6476344	6477261	+	NA
3156							
	MJLIPHIC_06216	CDS	COG5479	6477408	6479018	+	[M] Uncharacterized protein potentially involved in peptidoglycan
3157							
	MJLIPHIC_06217	CDS	COG0561	6479076	6479900	-	[R] Predicted hydrolases of the HAD superfamily
	MJLIPHIC_06218	CDS	COG0204	6479897	6480658	-	[I] 1-acyl-sn-glycerol-3-phosphate acyltransferase
	MJLIPHIC_06219	CDS	COG0204	6480665	6481447	-	[I] 1-acyl-sn-glycerol-3-phosphate acyltransferase
3158							
	MJLIPHIC_06220	CDS	COG2146	6481598	6483154	+	[PR] Ferredoxin subunits of nitrite reductase and
	MJLIPHIC_06221	CDS	ROG5838	6483151	6483483	+	NA
3159							
	MJLIPHIC_06222	CDS	ROG1743	6483487	6484305	-	NA
	MJLIPHIC_06223	CDS	COG0172	6484302	6485555	-	[J] Seryl-tRNA synthetase
3160							
	MJLIPHIC_06224	CDS	ROG2161	6485721	6487163	+	NA
	MJLIPHIC_06225	CDS	COG3824	6487179	6487517	+	[S] Uncharacterized protein conserved in bacteria
	MJLIPHIC_06226	CDS	NA	6487514	6487774	+	NA
3161							
	MJLIPHIC_06227	CDS	COG0406	6487771	6488457	-	[G] Fructose-2,6-bisphosphatase
	MJLIPHIC_06228	CDS	COG0077	6488454	6489410	-	[E] Prephenate dehydratase
3162							
	MJLIPHIC_06229	CDS	ROG2264	6489515	6490300	+	NA
	MJLIPHIC_06230	CDS	COG1266	6490338	6491120	+	[R] Predicted metal-dependent membrane protease
3163							
	MJLIPHIC_06231	CDS	NA	6491229	6491417	-	NA
3164							
	MJLIPHIC_06232	CDS	COG1316	6491564	6492484	+	[K] Transcriptional regulator
3165							
	MJLIPHIC_06233	CDS	COG1528	6492624	6493169	+	[P] Ferritin-like protein
3166							
	MJLIPHIC_06234	CDS	COG0584	6493183	6494028	-	[C] Glycerophosphoryl diester phosphodiesterase
	MJLIPHIC_06235	CDS	ROG5066	6494033	6495190	-	NA
	MJLIPHIC_06236	CDS	COG0607	6495219	6495560	-	[P] Rhodanese-related sulfurtransferase
3167							
	MJLIPHIC_06237	CDS	NA	6496032	6496190	+	NA
3168							
	MJLIPHIC_06238	CDS	COG0605	6496331	6496954	+	[P] Superoxide dismutase
3169							
	MJLIPHIC_06239	CDS	ROG7973	6497167	6497700	+	NA
3170							
	MJLIPHIC_06240	CDS	ROG8528	6498318	6500012	-	NA
	MJLIPHIC_06241	CDS	ROG6441	6500009	6500500	-	NA
3171							
	MJLIPHIC_06242	CDS	NA	6500573	6501679	-	NA
3172							
	MJLIPHIC_06243	CDS	ROG2041	6501891	6502547	+	NA
	MJLIPHIC_06244	CDS	NA	6502555	6503088	+	NA
	MJLIPHIC_06245	CDS	COG2372	6503125	6503676	+	[R] Uncharacterized protein, homolog of Cu resistance
	MJLIPHIC_06246	CDS	COG3336	6503652	6504617	+	[S] Predicted membrane protein
3173							
	MJLIPHIC_06247	CDS	NA	6504708	6505196	+	NA
	MJLIPHIC_06248	CDS	COG0684	6505226	6505705	+	[H] Demethylmenaquinone methyltransferase
3174							
	MJLIPHIC_06249	CDS	COG2072	6505769	6507247	-	[P] Predicted flavoprotein involved in K+ transport
3175							

	MJLIPHIC_06250	CDS	COG1309	6507325	6507978	+	[K] Transcriptional regulator
	MJLIPHIC_06251	CDS	COG0389	6508015	6509226	+	[L] Nucleotidyltransferase/DNA polymerase involved in DNA repair
3176							
	MJLIPHIC_06252	CDS	ROG7387	6509223	6509603	-	NA
3177							
	MJLIPHIC_06253	CDS	COG4298	6509624	6509908	+	[S] Uncharacterized protein conserved in bacteria
3178							
	MJLIPHIC_06254	CDS	COG1387	6509909	6510913	-	[ER] Histidinol phosphatase and related hydrolases of
3179							
	MJLIPHIC_06255	CDS	NA	6511011	6512072	+	NA
	MJLIPHIC_06256	CDS	NA	6512085	6512408	+	NA
	MJLIPHIC_06257	CDS	NA	6512438	6512932	+	NA
	MJLIPHIC_06258	CDS	COG1566	6512987	6514384	+	[V] Multidrug resistance efflux pump
3180							
	MJLIPHIC_06259	CDS	COG0640	6514667	6515059	+	[K] Predicted transcriptional regulators
	MJLIPHIC_06260	CDS	COG0659	6515056	6516696	+	[P] Sulfate permease and related transporters (MFS
3181							
	MJLIPHIC_06261	CDS	COG1012	6516693	6518039	-	[C] NAD-dependent aldehyde dehydrogenases
3182							
	MJLIPHIC_06262	CDS	NA	6518247	6519053	+	NA
3183							
	MJLIPHIC_06263	CDS	NA	6519063	6519647	-	NA
3184							
	MJLIPHIC_06264	CDS	NA	6519757	6519897	-	NA
3185							
	MJLIPHIC_06265	CDS	ROG3735	6520139	6520669	+	NA
3186							
	MJLIPHIC_06266	CDS	COG0493	6520673	6522139	-	[ER] NADPH-dependent glutamate synthase beta chain and
	MJLIPHIC_06267	CDS	COG0069	6522132	6526754	-	[E] Glutamate synthase domain 2
3187							
	MJLIPHIC_06268	CDS	COG3464	6527579	6528490	+	[L] Transposase and inactivated derivatives
3188							
	MJLIPHIC_06269	CDS	ROG0016	6528498	6529250	-	NA
3189							
	MJLIPHIC_06270	CDS	COG2050	6529689	6530489	+	[Q] Uncharacterized protein, possibly involved in aromatic
	MJLIPHIC_06271	CDS	COG0038	6530521	6531897	+	[P] Chloride channel protein EriC
	MJLIPHIC_06272	CDS	COG0580	6532006	6532779	+	[G] Glycerol uptake facilitator and related permeases
3190							
	MJLIPHIC_06273	CDS	COG0783	6532853	6533404	-	[P] DNA-binding ferritin-like protein (oxidative damage protectant)
	MJLIPHIC_06274	CDS	COG0122	6533466	6534350	-	[L] 3-methyladenine DNA glycosylase/8-oxoguanine DNA glycosylase
	MJLIPHIC_06275	CDS	COG2343	6534409	6534792	-	[S] Uncharacterized protein conserved in bacteria
3191							
	MJLIPHIC_06276	CDS	NA	6535030	6535236	+	NA
3192							
	MJLIPHIC_06277	CDS	COG0665	6535287	6536726	-	[E] Glycine/D-amino acid oxidases (deaminating)
3193							
	MJLIPHIC_06278	CDS	NA	6536834	6537058	+	NA
3194							
	MJLIPHIC_06279	CDS	COG3315	6537143	6537961	-	[Q] O-Methyltransferase involved in polyketide biosynthesis
3195							
	MJLIPHIC_06280	CDS	COG0702	6538024	6539073	-	[MG] Predicted nucleoside-diphosphate-sugar epimerases
	MJLIPHIC_06281	CDS	ROG4458	6539043	6539543	-	NA
	MJLIPHIC_06282	CDS	ROG1639	6539586	6539978	-	NA
3196							
	MJLIPHIC_06283	CDS	COG0225	6540054	6540569	+	[O] Peptide methionine sulfoxide reductase
3197							
	MJLIPHIC_06284	CDS	COG2124	6540573	6541916	-	[Q] Cytochrome P450
3198							
	MJLIPHIC_06285	CDS	COG1309	6542006	6542638	+	[K] Transcriptional regulator
3199							
	MJLIPHIC_06286	CDS	COG1309	6542615	6543169	-	[K] Transcriptional regulator
3200							
	MJLIPHIC_06287	CDS	ROG1108	6543238	6543909	+	NA
3201							
	MJLIPHIC_06288	CDS	ROG1556	6543906	6544952	-	NA
	MJLIPHIC_06289	CDS	COG0500	6544983	6545705	-	[QR] SAM-dependent methyltransferases
	MJLIPHIC_06290	CDS	COG0438	6545699	6546940	-	[M] Glycosyltransferase
3202							
	MJLIPHIC_06291	CDS	ROG7155	6547061	6547516	-	NA
	MJLIPHIC_06292	CDS	COG1902	6547545	6548636	-	[C] NADH:flavin oxidoreductases, Old Yellow Enzyme family
	MJLIPHIC_06293	CDS	COG2159	6548602	6549669	-	[R] Predicted metal-dependent hydrolase of the TIM-barrel
	MJLIPHIC_06294	CDS	ROG1294	6549677	6550114	-	NA
	MJLIPHIC_06295	CDS	ROG6417	6550150	6551340	-	NA
	MJLIPHIC_06296	CDS	ROG2472	6551337	6552680	-	NA
3203							
	MJLIPHIC_06297	CDS	COG1012	6552753	6554252	-	[C] NAD-dependent aldehyde dehydrogenases
3204							
	MJLIPHIC_06298	CDS	COG2508	6554429	6556201	+	[TQ] Regulator of polyketide synthase expression
3205							
	MJLIPHIC_06299	CDS	COG0715	6556375	6557424	+	[P] ABC-type nitrate/sulfonate/bicarbonate transport systems, periplasmic components
	MJLIPHIC_06300	CDS	COG0600	6557421	6558251	+	[P] ABC-type nitrate/sulfonate/bicarbonate transport system, permease component
	MJLIPHIC_06301	CDS	COG0600	6558248	6559027	+	[P] ABC-type nitrate/sulfonate/bicarbonate transport system, permease component
	MJLIPHIC_06302	CDS	COG1116	6559046	6559807	+	[P] ABC-type nitrate/sulfonate/bicarbonate transport system, ATPase component
3206							
	MJLIPHIC_06303	CDS	NA	6559895	6560428	-	NA
3207							
	MJLIPHIC_06304	CDS	NA	6560543	6560836	+	NA
3208							
	MJLIPHIC_06305	CDS	COG2343	6560975	6561772	+	[S] Uncharacterized protein conserved in bacteria
3209							
	MJLIPHIC_06306	CDS	ROG6192	6561852	6563714	+	NA
	MJLIPHIC_06307	CDS	NA	6563711	6563923	+	NA
	MJLIPHIC_06308	CDS	ROG5410	6563923	6564363	+	NA
	MJLIPHIC_06309	CDS	ROG3120	6564360	6565652	+	NA
3210							
	MJLIPHIC_06310	CDS	COG1309	6565649	6566215	-	[K] Transcriptional regulator
3211							
	MJLIPHIC_06311	CDS	COG0778	6566306	6567010	+	[C] Nitroreductase
	MJLIPHIC_06312	CDS	COG1335	6567028	6567579	+	[Q] Amidases related to nicotinamidase

3212							
	MJLIPHIC_06313	CDS	NA	6567435	6567734	-	NA
3213							
	MJLIPHIC_06314	CDS	COG1523	6567749	6569893	+	[G] Type II secretory pathway, pullulanase PulA
3214							
	MJLIPHIC_06315	CDS	COG1846	6570157	6570633	+	[K] Transcriptional regulators
	MJLIPHIC_06316	CDS	COG1131	6570630	6571499	+	[V] ABC-type multidrug transport system, ATPase component
	MJLIPHIC_06317	CDS	COG0842	6571489	6572292	+	[V] ABC-type multidrug transport system, permease component
	MJLIPHIC_06318	CDS	COG1960	6572351	6573508	+	[I] Acyl-CoA dehydrogenases
	MJLIPHIC_06319	CDS	COG1960	6573517	6574626	+	[I] Acyl-CoA dehydrogenases
3215							
	MJLIPHIC_06320	CDS	COG2814	6574647	6576638	-	[G] Arabinose efflux permease
	MJLIPHIC_06321	CDS	COG3281	6576690	6578015	-	[G] Uncharacterized protein, probably involved in trehalose
	MJLIPHIC_06322	CDS	COG0366	6578012	6579793	-	[G] Glycosidases
3216							
	MJLIPHIC_06323	CDS	ROG6392	6580061	6580456	+	NA
3217							
	MJLIPHIC_06324	CDS	ROG2264	6580577	6581377	+	NA
	MJLIPHIC_06325	CDS	COG0461	6581422	6582000	+	[F] Orotate phosphoribosyltransferase
3218							
	MJLIPHIC_06326	CDS	COG3842	6582005	6583069	-	[E] ABC-type spermidine/putrescine transport systems, ATPase components
	MJLIPHIC_06327	CDS	COG1178	6583066	6583887	-	[P] ABC-type Fe3+ transport system, permease component
	MJLIPHIC_06328	CDS	COG4132	6583884	6584744	-	[R] ABC-type uncharacterized transport system, permease component
	MJLIPHIC_06329	CDS	COG1840	6584753	6585904	-	[P] ABC-type Fe3+ transport system, periplasmic component
3219							
	MJLIPHIC_06330	CDS	NA	6586134	6586640	+	NA
	MJLIPHIC_06331	CDS	ROG2650	6586660	6587088	+	NA
3220							
	MJLIPHIC_06332	CDS	COG1309	6587093	6587659	-	[K] Transcriptional regulator
3221							
	MJLIPHIC_06333	CDS	NA	6587895	6588647	+	NA
	MJLIPHIC_06334	CDS	NA	6588644	6589393	+	NA
	MJLIPHIC_06335	CDS	COG1845	6589413	6589988	+	[C] Heme/copper-type cytochrome/quinol oxidase, subunit 3
	MJLIPHIC_06336	CDS	NA	6589990	6590283	+	NA
	MJLIPHIC_06337	CDS	COG1309	6590305	6590910	+	[K] Transcriptional regulator
3222							
	MJLIPHIC_06338	CDS	COG3208	6590911	6591846	-	[Q] Predicted thioesterase involved in non-ribosomal peptide
3223							
	MJLIPHIC_06339	CDS	COG0480	6592036	6594231	+	[J] Translation elongation factors (GTPases)
3224							
	MJLIPHIC_06340	CDS	ROG3446	6594233	6594799	-	NA
3225							
	MJLIPHIC_06341	CDS	COG1309	6594900	6595475	+	[K] Transcriptional regulator
	MJLIPHIC_06342	CDS	COG1028	6595503	6596210	+	[IQR] Dehydrogenases with different specificities (related to
3226							
	MJLIPHIC_06343	CDS	COG1463	6596379	6597620	+	[Q] ABC-type transport system involved in resistance
3227							
	MJLIPHIC_06344	CDS	COG5006	6597610	6598509	-	[R] Predicted permease, DMT superfamily
	MJLIPHIC_06345	CDS	ROG3479	6598543	6598893	-	NA
	MJLIPHIC_06346	CDS	ROG6619	6598890	6599987	-	NA
	MJLIPHIC_06347	CDS	COG2208	6599984	6600511	-	[TK] Serine phosphatase RsbU, regulator of sigma
	MJLIPHIC_06348	CDS	COG2208	6600484	6601626	-	[TK] Serine phosphatase RsbU, regulator of sigma
	MJLIPHIC_06349	CDS	NA	6601673	6601969	-	NA
3228							
	MJLIPHIC_06350	CDS	COG0406	6602127	6602834	-	[G] Fructose-2,6-bisphosphatase
	MJLIPHIC_06351	CDS	COG2207	6602836	6603669	-	[K] AraC-type DNA-binding domain-containing proteins
3229							
	MJLIPHIC_06352	CDS	COG1099	6603696	6604958	+	[R] Predicted metal-dependent hydrolases with the TIM-barrel
3230							
	MJLIPHIC_06353	CDS	COG0723	6604992	6605408	-	[C] Rieske Fe-S protein
	MJLIPHIC_06354	CDS	NA	6605424	6606014	-	NA
	MJLIPHIC_06355	CDS	NA	6606004	6606516	-	NA
3231							
	MJLIPHIC_06356	CDS	NA	6606582	6607028	-	NA
3232							
	MJLIPHIC_06357	CDS	COG2274	6607155	6608885	-	[V] ABC-type bacteriocin/lantibiotic exporters, contain an N-terminal
	MJLIPHIC_06358	CDS	COG1132	6608882	6611464	-	[V] ABC-type multidrug transport system, ATPase and
	MJLIPHIC_06359	CDS	ROG6516	6611464	6612174	-	NA
3233							
	MJLIPHIC_06360	CDS	COG1309	6612275	6612889	+	[K] Transcriptional regulator
	MJLIPHIC_06361	CDS	COG1024	6612886	6613542	+	[I] Enoyl-CoA hydratase/carnithine racemase
3234							
	MJLIPHIC_06362	CDS	COG3967	6613539	6614441	-	[M] Short-chain dehydrogenase involved in D-alanine esterification
	MJLIPHIC_06363	CDS	NA	6614451	6614747	-	NA
3235							
	MJLIPHIC_06364	CDS	COG1545	6614824	6615246	-	[R] Predicted nucleic-acid-binding protein containing a Zn-ribbon
	MJLIPHIC_06365	CDS	NA	6615249	6616469	-	NA
	MJLIPHIC_06366	CDS	COG1960	6616478	6618736	-	[I] Acyl-CoA dehydrogenases
	MJLIPHIC_06367	CDS	COG0702	6618733	6619743	-	[MG] Predicted nucleoside-diphosphate-sugar epimerases
3236							
	MJLIPHIC_06368	CDS	COG1309	6619839	6620408	+	[K] Transcriptional regulator
3237							
	MJLIPHIC_06369	CDS	COG1670	6620411	6621079	-	[J] Acetyltransferases, including N-acetylases of ribosomal proteins
	MJLIPHIC_06370	CDS	NA	6621103	6621258	-	NA
3238							
	MJLIPHIC_06371	CDS	COG2197	6621461	6622585	+	[TK] Response regulator containing a CheY-like receiver
3239							
	MJLIPHIC_06372	CDS	COG2837	6622524	6623711	-	[P] Predicted iron-dependent peroxidase
	MJLIPHIC_06373	CDS	COG2847	6623722	6624216	-	[S] Uncharacterized protein conserved in bacteria
	MJLIPHIC_06374	CDS	COG3182	6624209	6625501	-	[S] Uncharacterized iron-regulated membrane protein
3240							
	MJLIPHIC_06375	CDS	NA	6625702	6626244	-	NA
3241							
	MJLIPHIC_06376	CDS	COG0500	6626348	6626989	+	[QR] SAM-dependent methyltransferases
3242							
	MJLIPHIC_06377	CDS	ROG4954	6626995	6628662	-	NA
3243							
	MJLIPHIC_06378	CDS	ROG0284	6628925	6629410	+	NA
	MJLIPHIC_06379	CDS	COG1680	6629407	6630636	+	[V] Beta-lactamase class C and other penicillin
3244							

MJLIPHIC_06380 CDS ROG1053 6630646 6631086 - NA
3245
MJLIPHIC_06381 CDS COG4799 6631138 6632682 + [I] Acetyl-CoA carboxylase, carboxyltransferase component (subunits alpha
3246
MJLIPHIC_06382 CDS ROG7742 6633239 6633637 + NA
3247
MJLIPHIC_06383 CDS COG1733 6633752 6634201 + [K] Predicted transcriptional regulators
3248
MJLIPHIC_06384 CDS COG0300 6634284 6634952 + [R] Short-chain dehydrogenases of various substrate specificities
3249
MJLIPHIC_06385 CDS COG0627 6635047 6636249 + [R] Predicted esterase
3250
MJLIPHIC_06386 CDS COG0446 6636246 6637361 - [R] Uncharacterized NAD(FAD)-dependent dehydrogenases
3251
MJLIPHIC_06387 CDS COG1960 6637418 6638608 + [I] Acyl-CoA dehydrogenases
MJLIPHIC_06388 CDS COG1960 6638601 6639872 + [I] Acyl-CoA dehydrogenases
MJLIPHIC_06389 CDS COG0596 6639920 6640762 + [R] Predicted hydrolases or acyltransferases (alpha/beta hydrolase
MJLIPHIC_06390 CDS COG0179 6640759 6641664 + [Q] 2-keto-4-pentenoate hydratase/2-oxohepta-3-ene-1,7-dioic acid hydratase
(catechol pathway)
3252
MJLIPHIC_06391 CDS COG1643 6641661 6645653 - [L] HrpA-like helicases
MJLIPHIC_06392 CDS COG3335 6645663 6646724 - [L] Transposase and inactivated derivatives
3253
MJLIPHIC_06393 CDS ROG2444 6646800 6647195 + NA
MJLIPHIC_06394 CDS COG0300 6647198 6648052 + [R] Short-chain dehydrogenases of various substrate specificities
3254
MJLIPHIC_06395 CDS COG2964 6648028 6649758 - [S] Uncharacterized protein conserved in bacteria
MJLIPHIC_06396 CDS COG1126 6649755 6650522 - [E] ABC-type polar amino acid transport system,
MJLIPHIC_06397 CDS COG0765 6650515 6651408 - [E] ABC-type amino acid transport system, permease
MJLIPHIC_06398 CDS COG0834 6651395 6652369 - [ET] ABC-type amino acid transport/signal transduction systems,
3255
MJLIPHIC_06399 CDS NA 6652518 6653411 - NA
3256
MJLIPHIC_06400 CDS COG1073 6653552 6654313 + [R] Hydrolases of the alpha/beta superfamily
MJLIPHIC_06401 CDS COG0054 6654335 6654787 + [H] Riboflavin synthase beta-chain
3257
MJLIPHIC_06402 CDS NA 6654886 6655731 + NA
MJLIPHIC_06403 CDS COG2771 6655742 6656026 + [K] DNA-binding HTH domain-containing proteins
3258
MJLIPHIC_06404 CDS COG2801 6656133 6657125 + [L] Transposase and inactivated derivatives
3259
MJLIPHIC_06405 CDS NA 6657133 6657654 - NA
3260
MJLIPHIC_06406 CDS NA 6657780 6658817 - NA
MJLIPHIC_06407 CDS ROG0232 6658820 6659215 - NA
3261
MJLIPHIC_06408 CDS COG1695 6659352 6659954 + [K] Predicted transcriptional regulators
3262
MJLIPHIC_06409 CDS COG1309 6659918 6660517 - [K] Transcriptional regulator
MJLIPHIC_06410 CDS NA 6660569 6661243 - NA
MJLIPHIC_06411 CDS ROG0039 6661240 6662100 - NA
MJLIPHIC_06412 CDS NA 6662097 6663047 - NA
MJLIPHIC_06413 CDS ROG1601 6663038 6663502 - NA
MJLIPHIC_06414 CDS COG1721 6663492 6664352 - [R] Uncharacterized conserved protein (some members contain
MJLIPHIC_06415 CDS COG0714 6664363 6665397 - [R] MoxR-like ATPases
MJLIPHIC_06416 CDS NA 6665394 6666278 - NA
3263
MJLIPHIC_06417 CDS COG1408 6666377 6667543 - [R] Predicted phosphohydrolases
MJLIPHIC_06418 CDS ROG7307 6667571 6668248 - NA
MJLIPHIC_06419 CDS COG1063 6668245 6669426 - [ER] Threonine dehydrogenase and related Zn-dependent dehydrogenases
3264
MJLIPHIC_06420 CDS NA 6669781 6669990 - NA
3265
MJLIPHIC_06421 CDS NA 6670255 6670968 - NA
3266
MJLIPHIC_06422 CDS COG4581 6671013 6672065 + [L] Superfamily II RNA helicase
3267
MJLIPHIC_06423 CDS COG1051 6672072 6672791 - [F] ADP-ribose pyrophosphatase
MJLIPHIC_06424 CDS ROG2976 6672830 6673444 - NA
MJLIPHIC_06425 CDS NA 6673441 6674166 - NA
MJLIPHIC_06426 CDS NA 6674114 6674437 - NA
3268
MJLIPHIC_06427 CDS COG2141 6674557 6675408 - [C] Coenzyme F420-dependent N5,N10-methylene tetrahydromethanopterin reductase and
3269
MJLIPHIC_06428 CDS COG2124 6675471 6676661 + [Q] Cytochrome P450
3270
MJLIPHIC_06429 CDS COG1028 6676667 6677446 - [IQR] Dehydrogenases with different specificities (related to
MJLIPHIC_06430 CDS ROG2963 6677450 6678007 - NA
MJLIPHIC_06431 CDS COG2514 6678040 6678462 - [R] Predicted ring-cleavage extradiol dioxygenase
MJLIPHIC_06432 CDS COG1028 6678469 6679242 - [IQR] Dehydrogenases with different specificities (related to
3271
MJLIPHIC_06433 CDS COG0778 6679388 6680059 + [C] Nitroreductase
MJLIPHIC_06434 CDS COG3226 6680121 6680732 + [S] Uncharacterized protein conserved in bacteria
MJLIPHIC_06435 CDS COG3324 6680779 6681129 + [R] Predicted enzyme related to lactoylglutathione lyase
3272
MJLIPHIC_06436 CDS COG0331 6681119 6682024 - [I] (acyl-carrier-protein) S-malonyltransferase
MJLIPHIC_06437 CDS ROG7028 6682015 6682620 - NA
MJLIPHIC_06438 CDS ROG0511 6682617 6684233 - NA
MJLIPHIC_06439 CDS ROG0835 6684230 6684538 - NA
MJLIPHIC_06440 CDS COG1767 6684526 6685389 - [H] Triphosphoribosyl-dephospho-CoA synthetase
MJLIPHIC_06441 CDS ROG0487 6685394 6685996 - NA
3273
MJLIPHIC_06442 CDS COG0605 6686470 6687090 - [P] Superoxide dismutase
3274
MJLIPHIC_06443 CDS NA 6687242 6687499 + NA
3275
MJLIPHIC_06444 CDS COG0620 6687551 6689866 - [E] Methionine synthase II (cobalamin-independent)
3276
MJLIPHIC_06445 CDS COG1802 6690221 6690898 - [K] Transcriptional regulators
3277

	MJLIPHIC_06446	CDS	COG2141	6690967	6692328	+	[C] Coenzyme F420-dependent N5,N10-methylene tetrahydromethanopterin reductase and
	MJLIPHIC_06447	CDS	COG1804	6692329	6693528	+	[C] Predicted acyl-CoA transferases/carnitine dehydratase
	MJLIPHIC_06448	CDS	COG2301	6693546	6694457	+	[G] Citrate lyase beta subunit
3278							
	MJLIPHIC_06449	CDS	COG3800	6694441	6695859	-	[R] Predicted transcriptional regulator
3279							
	MJLIPHIC_06450	CDS	COG2079	6695977	6697482	+	[R] Uncharacterized protein involved in propionate catabolism
	MJLIPHIC_06451	CDS	COG2513	6697479	6698396	+	[G] PEP phosphonomutase and related enzymes
	MJLIPHIC_06452	CDS	COG0372	6698393	6699523	+	[C] Citrate synthase
	MJLIPHIC_06453	CDS	COG1038	6699516	6702911	+	[C] Pyruvate carboxylase
3280							
	MJLIPHIC_06454	CDS	NA	6702915	6703805	-	NA
3281							
	MJLIPHIC_06455	CDS	ROG4192	6704714	6705130	-	NA
	MJLIPHIC_06456	CDS	ROG0295	6705093	6707267	-	NA
	MJLIPHIC_06457	CDS	ROG4782	6707264	6708133	-	NA
3282							
	MJLIPHIC_06458	CDS	ROG8500	6708371	6709774	+	NA
3283							
	MJLIPHIC_06459	CDS	ROG4782	6709869	6710714	-	NA
	MJLIPHIC_06460	CDS	ROG3079	6710711	6712219	-	NA
	MJLIPHIC_06461	CDS	ROG0487	6712209	6713342	-	NA
	MJLIPHIC_06462	CDS	COG0596	6713339	6714169	-	[R] Predicted hydrolases or acyltransferases (alpha/beta hydrolase
3284							
	MJLIPHIC_06463	CDS	COG1457	6714430	6715866	+	[F] Purine-cytosine permease and related proteins
3285							
	MJLIPHIC_06464	CDS	NA	6715856	6715960	-	NA
3286							
	MJLIPHIC_06465	CDS	COG3315	6716067	6716909	+	[Q] O-Methyltransferase involved in polyketide biosynthesis
3287							
	MJLIPHIC_06466	CDS	COG1028	6717035	6717841	+	[IQR] Dehydrogenases with different specificities (related to
3288							
	MJLIPHIC_06467	CDS	COG0500	6717845	6718618	-	[QR] SAM-dependent methyltransferases
	MJLIPHIC_06468	CDS	ROG0969	6718615	6719640	-	NA
	MJLIPHIC_06469	CDS	ROG4575	6719637	6719912	-	NA
	MJLIPHIC_06470	CDS	NA	6719967	6720425	-	NA
	MJLIPHIC_06471	CDS	COG4329	6720444	6720974	-	[S] Predicted membrane protein
3289							
	MJLIPHIC_06472	CDS	COG1840	6721204	6722343	+	[P] ABC-type Fe3+ transport system, periplasmic component
	MJLIPHIC_06473	CDS	COG4132	6722340	6723293	+	[R] ABC-type uncharacterized transport system, permease component
	MJLIPHIC_06474	CDS	COG1178	6723290	6724114	+	[P] ABC-type Fe3+ transport system, permease component
	MJLIPHIC_06475	CDS	COG3842	6724124	6725167	+	[E] ABC-type spermidine/putrescine transport systems, ATPase components
	MJLIPHIC_06476	CDS	COG1524	6725164	6726183	+	[R] Uncharacterized proteins of the AP superfamily
3290							
	MJLIPHIC_06477	CDS	COG0154	6726242	6727711	+	[J] Asp-tRNAAsn/Glu-tRNAGln amidotransferase A subunit and related
3291							
	MJLIPHIC_06478	CDS	NA	6727730	6728623	-	NA
3292							
	MJLIPHIC_06479	CDS	ROG7907	6728888	6729547	+	NA
3293							
	MJLIPHIC_06480	CDS	NA	6729674	6730159	+	NA
3294							
	MJLIPHIC_06481	CDS	COG0583	6730109	6730969	-	[K] Transcriptional regulator
3295							
	MJLIPHIC_06482	CDS	COG1237	6731106	6732014	+	[R] Metal-dependent hydrolases of the beta-lactamase superfamily
3296							
	MJLIPHIC_06483	CDS	COG3059	6732021	6732491	-	[S] Predicted membrane protein
	MJLIPHIC_06484	CDS	ROG0463	6732488	6732724	-	NA
	MJLIPHIC_06485	CDS	COG1595	6732721	6733344	-	[K] DNA-directed RNA polymerase specialized sigma subunit,
3297							
	MJLIPHIC_06486	CDS	ROG2786	6733480	6733965	+	NA
	MJLIPHIC_06487	CDS	ROG1085	6733962	6735056	+	NA
3298							
	MJLIPHIC_06488	CDS	NA	6734977	6735234	-	NA
3299							
	MJLIPHIC_06489	CDS	COG0160	6735247	6736599	+	[E] 4-aminobutyrate aminotransferase and related aminotransferases
	MJLIPHIC_06490	CDS	COG1960	6736619	6737812	+	[I] Acyl-CoA dehydrogenases
	MJLIPHIC_06491	CDS	COG1012	6737809	6739272	+	[C] NAD-dependent aldehyde dehydrogenases
	MJLIPHIC_06492	CDS	COG1804	6739269	6740372	+	[C] Predicted acyl-CoA transferases/carnitine dehydratase
3300							
	MJLIPHIC_06493	CDS	COG2508	6740365	6741876	-	[TQ] Regulator of polyketide synthase expression
	MJLIPHIC_06494	CDS	COG2071	6741873	6742682	-	[R] Predicted glutamine amidotransferases
	MJLIPHIC_06495	CDS	COG2071	6742679	6743392	-	[R] Predicted glutamine amidotransferases
	MJLIPHIC_06496	CDS	COG0834	6743389	6744108	-	[ET] ABC-type amino acid transport/signal transduction systems,
	MJLIPHIC_06497	CDS	COG0174	6744105	6745490	-	[E] Glutamine synthetase
	MJLIPHIC_06498	CDS	ROG5258	6745496	6745684	-	NA
	MJLIPHIC_06499	CDS	COG2124	6745730	6746932	-	[Q] Cytochrome P450
3301							
	MJLIPHIC_06500	CDS	COG3464	6747617	6748528	+	[L] Transposase and inactivated derivatives
3302							
	MJLIPHIC_06501	CDS	ROG0016	6748536	6749237	-	NA
	MJLIPHIC_06502	CDS	NA	6749195	6750064	-	NA
	MJLIPHIC_06503	CDS	COG2186	6750061	6750798	-	[K] Transcriptional regulators
3303							
	MJLIPHIC_06504	CDS	COG0531	6750984	6752480	+	[E] Amino acid transporters
	MJLIPHIC_06505	CDS	COG1012	6752526	6753908	+	[C] NAD-dependent aldehyde dehydrogenases
	MJLIPHIC_06506	CDS	COG2141	6753954	6754784	+	[C] Coenzyme F420-dependent N5,N10-methylene tetrahydromethanopterin reductase and
	MJLIPHIC_06507	CDS	COG1414	6754873	6755619	+	[K] Transcriptional regulator
3304							
	MJLIPHIC_06508	CDS	COG0169	6755616	6756428	-	[E] Shikimate 5-dehydrogenase
3305							
	MJLIPHIC_06509	CDS	COG0596	6756549	6757454	+	[R] Predicted hydrolases or acyltransferases (alpha/beta hydrolase
	MJLIPHIC_06510	CDS	COG1028	6757445	6758197	+	[IQR] Dehydrogenases with different specificities (related to
	MJLIPHIC_06511	CDS	COG0596	6758217	6759065	+	[R] Predicted hydrolases or acyltransferases (alpha/beta hydrolase
3306							
	MJLIPHIC_06512	CDS	ROG2472	6759120	6760487	-	NA
	MJLIPHIC_06513	CDS	COG0371	6760497	6761582	-	[C] Glycerol dehydrogenase and related enzymes
	MJLIPHIC_06514	CDS	COG3485	6761579	6762454	-	[Q] Protocatechuate 3,4-dioxygenase beta subunit
	MJLIPHIC_06515	CDS	ROG2425	6762451	6763068	-	NA
3307							

	MJLIPHIC_06516	CDS	COG1082	6763271	6764110	+	[G] Sugar phosphate isomerases/epimerases
	MJLIPHIC_06517	CDS	COG1082	6764137	6764958	+	[G] Sugar phosphate isomerases/epimerases
	MJLIPHIC_06518	CDS	COG0673	6764976	6766046	+	[R] Predicted dehydrogenases and related proteins
3308							
	MJLIPHIC_06519	CDS	NA	6766137	6767504	+	NA
	MJLIPHIC_06520	CDS	COG0596	6767501	6768358	+	[R] Predicted hydrolases or acyltransferases (alpha/beta hydrolase
3309							
	MJLIPHIC_06521	CDS	COG0596	6768359	6769222	-	[R] Predicted hydrolases or acyltransferases (alpha/beta hydrolase
3310							
	MJLIPHIC_06522	CDS	COG3662	6769511	6770410	+	[S] Uncharacterized protein conserved in bacteria
3311							
	MJLIPHIC_06523	CDS	ROG4798	6770504	6771022	-	NA
	MJLIPHIC_06524	CDS	COG0619	6771009	6771791	-	[P] ABC-type cobalt transport system, permease component
	MJLIPHIC_06525	CDS	COG1122	6771788	6773263	-	[P] ABC-type cobalt transport system, ATPase component
	MJLIPHIC_06526	CDS	COG4721	6773260	6773889	-	[S] Predicted membrane protein
3312							
	MJLIPHIC_06527	CDS	COG0531	6774109	6775455	+	[E] Amino acid transporters
	MJLIPHIC_06528	CDS	ROG1086	6775459	6775947	+	NA
3313							
	MJLIPHIC_06529	CDS	COG0667	6776043	6776948	+	[C] Predicted oxidoreductases (related to aryl-alcohol dehydrogenases)
3314							
	MJLIPHIC_06530	CDS	ROG1633	6776891	6777760	-	NA
3315							
	MJLIPHIC_06531	CDS	COG1309	6777839	6778417	+	[K] Transcriptional regulator
3316							
	MJLIPHIC_06532	CDS	ROG4626	6778536	6779126	+	NA
3317							
	MJLIPHIC_06533	CDS	COG0388	6779127	6779912	-	[R] Predicted amidohydrolase
	MJLIPHIC_06534	CDS	COG2141	6779938	6781251	-	[C] Coenzyme F420-dependent N5,N10-methylene tetrahydromethanopterin reductase and
	MJLIPHIC_06535	CDS	COG0531	6781284	6782720	-	[E] Amino acid transporters
	MJLIPHIC_06536	CDS	COG1609	6782849	6783850	-	[K] Transcriptional regulators
3318							
	MJLIPHIC_06537	CDS	NA	6784163	6785038	+	NA
3319							
	MJLIPHIC_06538	CDS	COG1802	6785045	6785728	-	[K] Transcriptional regulators
3320							
	MJLIPHIC_06539	CDS	NA	6785888	6786334	+	NA
3321							
	MJLIPHIC_06540	CDS	COG2515	6786544	6787548	+	[E] 1-aminocyclopropane-1-carboxylate deaminase
3322							
	MJLIPHIC_06541	CDS	ROG3614	6787549	6788229	-	NA
	MJLIPHIC_06542	CDS	ROG6462	6788255	6788845	-	NA
3323							
	MJLIPHIC_06543	CDS	COG2514	6788929	6789396	+	[R] Predicted ring-cleavage extradiol dioxygenase
	MJLIPHIC_06544	CDS	COG3576	6789407	6790261	+	[R] Predicted flavin-nucleotide-binding protein structurally related to
	MJLIPHIC_06545	CDS	COG2188	6790305	6791048	+	[K] Transcriptional regulators
3324							
	MJLIPHIC_06546	CDS	COG0667	6791052	6792182	-	[C] Predicted oxidoreductases (related to aryl-alcohol dehydrogenases)
3325							
	MJLIPHIC_06547	CDS	COG0604	6792281	6793198	+	[CR] NADPH:quinone reductase and related Zn-dependent oxidoreductases
3326							
	MJLIPHIC_06548	CDS	COG0488	6793199	6794866	-	[R] ATPase components of ABC transporters with
	MJLIPHIC_06549	CDS	ROG2178	6794909	6795121	-	NA
	MJLIPHIC_06550	CDS	ROG3869	6795153	6795908	-	NA
3327							
	MJLIPHIC_06551	CDS	NA	6796078	6796293	+	NA
3328							
	MJLIPHIC_06552	CDS	COG5297	6796297	6797232	-	[G] Cellobiohydrolase A (1,4-beta-cellobiosidase A)
3329							
	MJLIPHIC_06553	CDS	COG1028	6797368	6798090	+	[IQR] Dehydrogenases with different specificities (related to
3330							
	MJLIPHIC_06554	CDS	ROG0252	6798433	6799461	+	NA
	MJLIPHIC_06555	CDS	NA	6799518	6800441	+	NA
3331							
	MJLIPHIC_06556	CDS	COG0554	6800466	6801983	-	[C] Glycerol kinase
	MJLIPHIC_06557	CDS	COG1414	6801980	6802756	-	[K] Transcriptional regulator
3332							
	MJLIPHIC_06558	CDS	COG0580	6802924	6803739	+	[G] Glycerol uptake facilitator and related permeases
	MJLIPHIC_06559	CDS	COG0554	6803763	6805280	+	[C] Glycerol kinase
	MJLIPHIC_06560	CDS	COG0578	6805277	6806983	+	[C] Glycerol-3-phosphate dehydrogenase
3333							
	MJLIPHIC_06561	CDS	COG3832	6806973	6807458	-	[S] Uncharacterized conserved protein
	MJLIPHIC_06562	CDS	COG0640	6807460	6807807	-	[K] Predicted transcriptional regulators
	MJLIPHIC_06563	CDS	COG0596	6807853	6808677	-	[R] Predicted hydrolases or acyltransferases (alpha/beta hydrolase
3334							
	MJLIPHIC_06564	CDS	COG1309	6808750	6809331	+	[K] Transcriptional regulator
3335							
	MJLIPHIC_06565	CDS	ROG2436	6809341	6810183	-	NA
	MJLIPHIC_06566	CDS	COG1131	6810183	6811019	-	[V] ABC-type multidrug transport system, ATPase component
	MJLIPHIC_06567	CDS	COG3321	6811016	6816490	-	[Q] Polyketide synthase modules and related proteins
3336							
	MJLIPHIC_06568	CDS	ROG0277	6816577	6818328	-	NA
	MJLIPHIC_06569	CDS	COG0475	6818359	6819639	-	[P] Kef-type K+ transport systems, membrane components
3337							
	MJLIPHIC_06570	CDS	COG3631	6819891	6820289	+	[R] Ketosteroid isomerase-related protein
	MJLIPHIC_06571	CDS	ROG5709	6820316	6820858	+	NA
3338							
	MJLIPHIC_06572	CDS	COG2021	6820932	6821960	+	[E] Homoserine acetyltransferase
3339							
	MJLIPHIC_06573	CDS	COG1804	6822076	6823278	+	[C] Predicted acyl-CoA transferases/carnitine dehydratase
	MJLIPHIC_06574	CDS	COG1024	6823288	6824070	+	[I] Enoyl-CoA hydratase/carnithine racemase
	MJLIPHIC_06575	CDS	COG0477	6824092	6825447	+	[GEPR] Permeases of the major facilitator superfamily
	MJLIPHIC_06576	CDS	COG0583	6825472	6826359	+	[K] Transcriptional regulator
	MJLIPHIC_06577	CDS	COG1024	6826399	6827178	+	[I] Enoyl-CoA hydratase/carnithine racemase
3340							
	MJLIPHIC_06578	CDS	NA	6827190	6827630	-	NA
	MJLIPHIC_06579	CDS	ROG2017	6827676	6828323	-	NA
3341							
	MJLIPHIC_06580	CDS	COG0384	6828361	6829233	+	[R] Predicted epimerase, PhzC/PhzF homolog
3342							

	MJLIPHIC_06581	CDS	COG2267	6829246	6830718	-	[I] Lysophospholipase
3343							
	MJLIPHIC_06582	CDS	NA	6831167	6832159	-	NA
	MJLIPHIC_06583	CDS	COG1680	6832219	6833313	-	[V] Beta-lactamase class C and other penicillin
3344							
	MJLIPHIC_06584	CDS	NA	6833391	6833990	+	NA
3345							
	MJLIPHIC_06585	CDS	COG0149	6833969	6834760	-	[G] Triosephosphate isomerase
	MJLIPHIC_06586	CDS	COG0698	6834753	6835211	-	[G] Ribose 5-phosphate isomerase RpiB
	MJLIPHIC_06587	CDS	COG2376	6835256	6836971	-	[G] Dihydroxyacetone kinase
	MJLIPHIC_06588	CDS	COG1802	6837064	6837840	-	[K] Transcriptional regulators
3346							
	MJLIPHIC_06589	CDS	COG1082	6838029	6838949	+	[G] Sugar phosphate isomerases/epimerases
	MJLIPHIC_06590	CDS	COG1250	6838975	6839937	+	[I] 3-hydroxyacyl-CoA dehydrogenase
	MJLIPHIC_06591	CDS	COG2610	6840005	6841405	+	[GE] H+/gluconate symporter and related permeases
3347							
	MJLIPHIC_06592	CDS	ROG1030	6841426	6842814	-	NA
3348							
	MJLIPHIC_06593	CDS	ROG1633	6842901	6843767	-	NA
3349							
	MJLIPHIC_06594	CDS	COG1024	6843947	6844789	+	[I] Enoyl-CoA hydratase/carnithine racemase
	MJLIPHIC_06595	CDS	COG3631	6844782	6845168	+	[R] Ketosteroid isomerase-related protein
	MJLIPHIC_06596	CDS	COG0599	6845184	6845597	+	[S] Uncharacterized homolog of gamma-carboxymuconolactone decarboxylase subunit
	MJLIPHIC_06597	CDS	COG0667	6845594	6846700	+	[C] Predicted oxidoreductases (related to aryl-alcohol dehydrogenases)
3350							
	MJLIPHIC_06598	CDS	COG1609	6846555	6847544	-	[K] Transcriptional regulators
	MJLIPHIC_06599	CDS	COG1072	6847550	6848200	-	[H] Panthothenate kinase
	MJLIPHIC_06600	CDS	COG0524	6848197	6849126	-	[G] Sugar kinases, ribokinase family
	MJLIPHIC_06601	CDS	COG1129	6849104	6849895	-	[G] ABC-type sugar transport system, ATPase component
	MJLIPHIC_06602	CDS	COG1172	6849892	6850911	-	[G] Ribose/xylose/arabinose/galactoside ABC-type transport systems, permease components
	MJLIPHIC_06603	CDS	COG1879	6850913	6851911	-	[G] ABC-type sugar transport system, periplasmic component
3351							
	MJLIPHIC_06604	CDS	COG1680	6852036	6853556	+	[V] Beta-lactamase class C and other penicillin
3352							
	MJLIPHIC_06605	CDS	COG0178	6853636	6856167	-	[L] Excinuclease ATPase subunit
	MJLIPHIC_06606	CDS	COG1309	6856188	6856793	-	[K] Transcriptional regulator
3353							
	MJLIPHIC_06607	CDS	NA	6856899	6858059	+	NA
	MJLIPHIC_06608	CDS	COG5271_4X	6858052	6858855	+	NA
	MJLIPHIC_06609	CDS	COG4548	6858852	6860519	+	[P] Nitric oxide reductase activation protein
3354							
	MJLIPHIC_06610	CDS	ROG1030	6860658	6861881	-	NA
3355							
	MJLIPHIC_06611	CDS	COG0477	6862029	6862958	-	[GEPR] Permeases of the major facilitator superfamily
	MJLIPHIC_06612	CDS	COG2159	6863010	6863963	-	[R] Predicted metal-dependent hydrolase of the TIM-barrel
3356							
	MJLIPHIC_06613	CDS	COG1846	6864033	6864503	+	[K] Transcriptional regulators
	MJLIPHIC_06614	CDS	ROG5315	6864557	6865342	+	NA
3357							
	MJLIPHIC_06615	CDS	COG0243	6865435	6867597	+	[C] Anaerobic dehydrogenases, typically selenocysteine-containing
3358							
	MJLIPHIC_06616	CDS	COG1595	6867601	6868491	-	[K] DNA-directed RNA polymerase specialized sigma subunit,
	MJLIPHIC_06617	CDS	COG1252	6868472	6869734	-	[C] NADH dehydrogenase, FAD-containing subunit
3359							
	MJLIPHIC_06618	CDS	NA	6869763	6870128	+	NA
	MJLIPHIC_06619	CDS	COG1105	6870182	6870715	+	[G] Fructose-1-phosphate kinase and related fructose-6-phosphate kinase
	MJLIPHIC_06620	CDS	COG0496	6870741	6871520	+	[R] Predicted acid phosphatase
3360							
	MJLIPHIC_06621	CDS	ROG3702	6871529	6872626	-	NA
3361							
	MJLIPHIC_06622	CDS	ROG1505	6872926	6873837	+	NA
3362							
	MJLIPHIC_06623	CDS	COG1028	6873845	6874579	-	[IQR] Dehydrogenases with different specificities (related to
3363							
	MJLIPHIC_06624	CDS	COG1846	6874761	6875189	-	[K] Transcriptional regulators
3364							
	MJLIPHIC_06625	CDS	COG1266	6875319	6876185	+	[R] Predicted metal-dependent membrane protease
3365							
	MJLIPHIC_06626	CDS	COG1620	6876287	6877969	+	[C] L-lactate permease
3366							
	MJLIPHIC_06627	CDS	NA	6877979	6878077	-	NA
	MJLIPHIC_06628	CDS	COG2771	6878097	6880823	-	[K] DNA-binding HTH domain-containing proteins
3367							
	MJLIPHIC_06629	CDS	COG1733	6880933	6881337	+	[K] Predicted transcriptional regulators
	MJLIPHIC_06630	CDS	NA	6881399	6882127	+	NA
3368							
	MJLIPHIC_06631	CDS	COG0596	6882131	6882982	-	[R] Predicted hydrolases or acyltransferases (alpha/beta hydrolase
	MJLIPHIC_06632	CDS	ROG1436	6883001	6883579	-	NA
3369							
	MJLIPHIC_06633	CDS	COG1064	6883736	6884752	+	[R] Zn-dependent alcohol dehydrogenases
	MJLIPHIC_06634	CDS	COG1064	6884787	6885815	+	[R] Zn-dependent alcohol dehydrogenases
3370							
	MJLIPHIC_06635	CDS	COG3239	6885835	6887229	-	[I] Fatty acid desaturase
	MJLIPHIC_06636	CDS	COG1018	6887256	6887666	-	[C] Flavodoxin reductases (ferredoxin-NADPH reductases) family 1
3371							
	MJLIPHIC_06637	CDS	COG0596	6887818	6888333	+	[R] Predicted hydrolases or acyltransferases (alpha/beta hydrolase
3372							
	MJLIPHIC_06638	CDS	COG0596	6888294	6889229	-	[R] Predicted hydrolases or acyltransferases (alpha/beta hydrolase
3373							
	MJLIPHIC_06639	CDS	COG4977	6889400	6890398	+	[K] Transcriptional regulator containing an amidase domain
3374							
	MJLIPHIC_06640	CDS	COG0583	6890416	6891297	-	[K] Transcriptional regulator
3375							
	MJLIPHIC_06641	CDS	COG0160	6891408	6892730	+	[E] 4-aminobutyrate aminotransferase and related aminotransferases
3376							
	MJLIPHIC_06642	CDS	COG0451	6892736	6893554	-	[MG] Nucleoside-diphosphate-sugar epimerases
	MJLIPHIC_06643	CDS	COG1638	6893571	6894623	-	[G] TRAP-type C4-dicarboxylate transport system, periplasmic component
	MJLIPHIC_06644	CDS	COG1593	6894636	6895931	-	[G] TRAP-type C4-dicarboxylate transport system, large permease
	MJLIPHIC_06645	CDS	COG3090	6895944	6896453	-	[G] TRAP-type C4-dicarboxylate transport system, small permease

MJLIPHIC_06646 CDS ROG3164 6896461 6897615 - NA
3377
MJLIPHIC_06647 CDS ROG1744 6897798 6899045 - NA
MJLIPHIC_06648 CDS COG0583 6899091 6900023 - [K] Transcriptional regulator
3378
MJLIPHIC_06649 CDS COG0596 6900106 6900822 + [R] Predicted hydrolases or acyltransferases (alpha/beta hydrolase
MJLIPHIC_06650 CDS COG0657 6900819 6901784 + [I] Esterase/lipase
3379
MJLIPHIC_06651 CDS COG1881 6901769 6902308 - [R] Phospholipid-binding protein
MJLIPHIC_06652 CDS COG0066 6902305 6902853 - [E] 3-isopropylmalate dehydratase small subunit
MJLIPHIC_06653 CDS COG0065 6902850 6904118 - [E] 3-isopropylmalate dehydratase large subunit
MJLIPHIC_06654 CDS COG2513 6904120 6905010 - [G] PEP phosphonomutase and related enzymes
MJLIPHIC_06655 CDS COG2079 6905010 6906443 - [R] Uncharacterized protein involved in propionate catabolism
3380
MJLIPHIC_06656 CDS COG1414 6906505 6907275 + [K] Transcriptional regulator
3381
MJLIPHIC_06657 CDS COG0596 6907277 6908395 - [R] Predicted hydrolases or acyltransferases (alpha/beta hydrolase
MJLIPHIC_06658 CDS COG4221 6908449 6909201 - [R] Short-chain alcohol dehydrogenase of unknown specificity
3382
MJLIPHIC_06659 CDS COG3631 6909291 6909740 - [R] Ketosteroid isomerase-related protein
3383
MJLIPHIC_06660 CDS COG1396 6909812 6910366 - [K] Predicted transcriptional regulators
3384
MJLIPHIC_06661 CDS COG1309 6910524 6911108 + [K] Transcriptional regulator
MJLIPHIC_06662 CDS COG0235 6911105 6911872 + [G] Ribulose-5-phosphate 4-epimerase and related epimerases and
3385
MJLIPHIC_06663 CDS COG1414 6912063 6912704 - [K] Transcriptional regulator
3386
MJLIPHIC_06664 CDS COG0747 6912953 6914365 + [E] ABC-type dipeptide transport system, periplasmic component
MJLIPHIC_06665 CDS COG0601 6914358 6915356 + [EP] ABC-type dipeptide/oligopeptide/nickel transport systems, permease components
MJLIPHIC_06666 CDS COG1173 6915353 6916180 + [EP] ABC-type dipeptide/oligopeptide/nickel transport systems, permease components
MJLIPHIC_06667 CDS COG1123 6916180 6916989 + [R] ATPase components of various ABC-type transport
MJLIPHIC_06668 CDS COG1123 6916986 6917819 + [R] ATPase components of various ABC-type transport
3387
MJLIPHIC_06669 CDS COG1940 6917828 6918790 - [KG] Transcriptional regulator/sugar kinase
3388
MJLIPHIC_06670 CDS COG1402 6918823 6919647 + [R] Uncharacterized protein, putative amidase
MJLIPHIC_06671 CDS COG4948 6919644 6920771 + [MR] L-alanine-DL-glutamate epimerase and related enzymes of
3389
MJLIPHIC_06672 CDS COG1680 6920945 6921817 + [V] Beta-lactamase class C and other penicillin
3390
MJLIPHIC_06673 CDS COG1024 6921842 6922234 - [I] Enoyl-CoA hydratase/carnithine racemase
MJLIPHIC_06674 CDS COG1012 6922554 6924056 - [C] NAD-dependent aldehyde dehydrogenases
MJLIPHIC_06675 CDS COG0251 6924099 6925229 - [J] Putative translation initiation inhibitor, yjgF family
3391
MJLIPHIC_06676 CDS COG0410 6925321 6926043 - [E] ABC-type branched-chain amino acid transport systems,
MJLIPHIC_06677 CDS COG0411 6926040 6926819 - [E] ABC-type branched-chain amino acid transport systems,
MJLIPHIC_06678 CDS COG4177 6926816 6927790 - [E] ABC-type branched-chain amino acid transport system,
MJLIPHIC_06679 CDS COG0559 6927790 6928653 - [E] Branched-chain amino acid ABC-type transport system,
MJLIPHIC_06680 CDS COG0683 6928729 6929985 - [E] ABC-type branched-chain amino acid transport systems,
MJLIPHIC_06681 CDS COG1802 6930093 6930737 - [K] Transcriptional regulators
3392
MJLIPHIC_06682 CDS COG1028 6930866 6931108 + [IQR] Dehydrogenases with different specificities (related to
3393
MJLIPHIC_06683 CDS COG0583 6931229 6932158 - [K] Transcriptional regulator
3394
MJLIPHIC_06684 CDS COG2085 6932415 6932957 + [R] Predicted dinucleotide-binding enzymes
3395
MJLIPHIC_06685 CDS NA 6933023 6933847 + NA
3396
MJLIPHIC_06686 CDS COG1228 6934366 6935613 + [Q] Imidazolonepropionase and related amidohydrolases
MJLIPHIC_06687 CDS NA 6935634 6935837 + NA
3397
MJLIPHIC_06688 CDS COG2801 6935806 6936627 - [L] Transposase and inactivated derivatives
MJLIPHIC_06689 CDS COG2963 6936660 6936968 - [L] Transposase and inactivated derivatives
3398
MJLIPHIC_06690 CDS COG1359 6937052 6937345 + [S] Uncharacterized conserved protein
3399
MJLIPHIC_06691 CDS COG0305 6937984 6941067 - [L] Replicative DNA helicase
3400
MJLIPHIC_06692 CDS COG0359 6941588 6942043 - [J] Ribosomal protein L9
MJLIPHIC_06693 CDS COG0238 6942062 6942316 - [J] Ribosomal protein S18
MJLIPHIC_06694 CDS COG0629 6942354 6942851 - [L] Single-stranded DNA-binding protein
MJLIPHIC_06695 CDS COG0360 6942924 6943214 - [J] Ribosomal protein S6
3401
MJLIPHIC_06696 CDS NA 6943394 6943984 + NA
3402
MJLIPHIC_06697 CDS COG5650 6943954 6945618 - [S] Predicted integral membrane protein
MJLIPHIC_06698 CDS COG0744 6945615 6948098 - [M] Membrane carboxypeptidase (penicillin-binding protein)
MJLIPHIC_06699 CDS ROG0974 6948091 6948501 - NA
3403
MJLIPHIC_06700 CDS ROG2484 6948613 6949461 + NA
3404
MJLIPHIC_06701 CDS COG1695 6949605 6950150 + [K] Predicted transcriptional regulators
MJLIPHIC_06702 CDS COG1260 6950228 6951319 + [I] Myo-inositol-1-phosphate synthase
MJLIPHIC_06703 CDS COG0596 6951334 6952305 + [R] Predicted hydrolases or acyltransferases (alpha/beta hydrolase
MJLIPHIC_06704 CDS NA 6952349 6953122 + NA
3405
MJLIPHIC_06705 CDS COG1802 6953210 6953875 + [K] Transcriptional regulators
MJLIPHIC_06706 CDS COG0765 6953922 6955694 + [E] ABC-type amino acid transport system, permease
MJLIPHIC_06707 CDS COG1126 6955694 6956431 + [E] ABC-type polar amino acid transport system,
MJLIPHIC_06708 CDS COG1846 6956496 6956996 + [K] Transcriptional regulators
3406
MJLIPHIC_06709 CDS ROG3162 6957005 6958357 - NA
3407
MJLIPHIC_06710 CDS COG4221 6958400 6959065 + [R] Short-chain alcohol dehydrogenase of unknown specificity
3408
MJLIPHIC_06711 CDS COG0495 6959072 6961933 - [J] Leucyl-tRNA synthetase
3409
MJLIPHIC_06712 CDS NA 6962147 6963091 + NA

```
3410
        MJLIPHIC_06713  CDS     ROG7637 6963161 6963529 +       NA
3411
        MJLIPHIC_06714  CDS     COG1678 6963530 6964135 -       [K] Putative transcriptional regulator
3412
        MJLIPHIC_06715  CDS     NA      6964271 6965557 +       NA
        MJLIPHIC_06716  CDS     ROG1303 6965572 6966345 +       NA
3413
        MJLIPHIC_06717  CDS     NA      6966439 6966936 -       NA
        MJLIPHIC_06718  CDS     COG0617 6966955 6968397 -       [J] tRNA nucleotidyltransferase/poly(A) polymerase
3414
        MJLIPHIC_06719  CDS     COG1051 6968461 6969375 +       [F] ADP-ribose pyrophosphatase
        MJLIPHIC_06720  CDS     ROG1037 6969357 6971798 +       NA
        MJLIPHIC_06721  CDS     COG0728 6971795 6975445 +       [R] Uncharacterized membrane protein, putative virulence factor
        MJLIPHIC_06722  CDS     COG1595 6975546 6976172 +       [K] DNA-directed RNA polymerase specialized sigma subunit,
        MJLIPHIC_06723  CDS     ROG6028 6976117 6976860 +       NA
3415
        MJLIPHIC_06724  CDS     COG0492 6976965 6977900 +       [O] Thioredoxin reductase
        MJLIPHIC_06725  CDS     COG0526 6977934 6978266 +       [OC] Thiol-disulfide isomerase and thioredoxins
3416
        MJLIPHIC_06726  CDS     COG3409 6978405 6979595 +       [M] Putative peptidoglycan-binding domain-containing protein
3417
        MJLIPHIC_06727  CDS     ROG0891 6979641 6980393 -       NA
3418
        MJLIPHIC_06728  CDS     COG1475 6980632 6981687 -       [K] Predicted transcriptional regulators
        MJLIPHIC_06729  CDS     COG1192 6981747 6982718 -       [D] ATPases involved in chromosome partitioning
        MJLIPHIC_06730  CDS     COG0357 6982721 6983437 -       [M] Predicted S-adenosylmethionine-dependent methyltransferase involved in
bacterial
        MJLIPHIC_06731  CDS     COG1847 6983492 6984043 -       [R] Predicted RNA-binding protein
        MJLIPHIC_06732  CDS     COG0706 6984079 6985182 -       [U] Preprotein translocase subunit YidC
        MJLIPHIC_06733  CDS     COG0759 6985175 6985522 -       [S] Uncharacterized conserved protein
        MJLIPHIC_06734  CDS     COG0594 6985519 6985911 -       [J] RNase P protein component
        MJLIPHIC_06735  CDS     ROG1730 6985943 6986086 -       NA
3419
        MJLIPHIC_06736  CDS     NA      6986600 6988114 +       NA
```
